# Selective skeletal editing of polycyclic arenes using organophotoredox dearomative functionalization

**DOI:** 10.1038/s41467-022-32201-7

**Published:** 2022-08-05

**Authors:** Peng Ji, Cassondra C. Davies, Feng Gao, Jing Chen, Xiang Meng, Kendall N. Houk, Shuming Chen, Wei Wang

**Affiliations:** 1 Departments of Pharmacology and Toxicology and Chemistry and Biochemistry, University of Arizona, Tucson, AZ 85721-0207 USA; 2 Department of Chemistry and Biochemistry, Oberlin College, Oberlin, OH 44074 USA; 3 Department of Chemistry and Biochemistry, University of California, Los Angeles, CA 90095-1569 USA

**Keywords:** Synthetic chemistry methodology, Photocatalysis

## Abstract

Reactions that lead to destruction of aromatic ring systems often require harsh conditions and, thus, take place with poor selectivities. Selective partial dearomatization of fused arenes is even more challenging but can be a strategic approach to creating versatile, complex polycyclic frameworks. Herein we describe a general organophotoredox approach for the chemo- and regioselective dearomatization of structurally diverse polycyclic aromatics, including quinolines, isoquinolines, quinoxalines, naphthalenes, anthracenes and phenanthrenes. The success of the method for chemoselective oxidative rupture of aromatic moieties relies on precise manipulation of the electronic nature of the fused polycyclic arenes. Mechanistic studies show that the addition of a hydrogen atom transfer (HAT) agent helps favor the dearomatization pathway over the more thermodynamically downhill aromatization pathway. We show that this strategy can be applied to rapid synthesis of biologically valued targets and late-stage skeletal remodeling en route to complex structures.

## Abstract (web summary)

The synthesis of polycyclic structures bearing partially saturated (hetero)arenes is important as many of these structures have biological relevance. Here the authors develop a one-step method for selective dearomative functionalization of feedstock (hetero)arenes via organophotocatalysis.

## Introduction

Polycyclic scaffolds bearing partially dearomatized fused arenes are commonly encountered in natural products, pharmaceuticals and bioactive molecules^1–5^. While these molecular frameworks lead to great structural diversity and intriguing biological properties, they engender synthetic challenges as their assembly often requires prefunctionalized substrates and multi-step sequences. Direct dearomatization of fused arenes constitutes an efficient approach for the construction of polycyclic scaffolds due to its high atom and step economy^1–5^. However, selective dearomatization and functionalization of fused arenes is difficult because harsh conditions are often required to disrupt aromaticity, leading to poor selectivities. As a result, dearomative functionalization of fused arenes has been largely limited to activated arenes such as indoles^6–15^ and naphthols^16–20^.

**Fig. 1:**
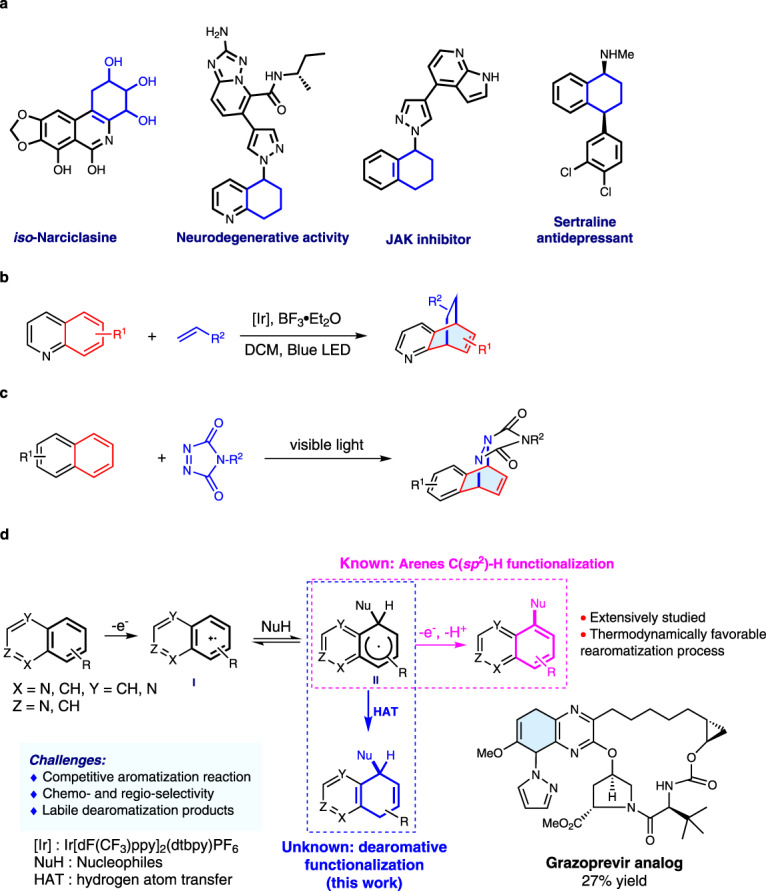
Methods for selective dearomative functionalization of fused arenes. **a** Examples of partially dearomatic natural products and bioactive compounds. **b** Selective dearomatization of quinolines. **c** Selective dearomatization of naphthalenes. **d** Selective dearomatization of structurally diverse fused arenes (this work).

Issues of chemo- and regioselectivity also add to the difficulty of dearomative functionalizations of unactivated fused arenes such as quinolines and naphthalenes. Selective dearomatization of pyridine moieties in fused arenes is feasible^21–25^ owing to the electron deficient nature of pyridine and assistance provided by Lewis acid complexation. Selective dearomatization of phenyl moieties in fused arenes, on the other hand, is significantly more challenging. To our knowledge, only one example has been described in a recent report by Brown, Houk and Glorius involving a photochemical [4 + 2] cycloaddition between quinolines and alkenes (Fig. 1b)^26^ enabled by energy transfer and Lewis acid activation. A similar [4 + 2] cycloaddition strategy initiated by energy transfer was also employed in the dearomatization of naphthalene by Sarlah et al. (Fig. 1c)^27–32^.

We wondered if a photoredox strategy could be used to selectively dearomatize the phenyl moieties in quinolines, isoquinolines, quinoxalines, naphthalenes and other fused arenes. In reported photoredox processes, radicals add selectively to the pyridine ring in quinolines^33,34^, and indiscriminately to the phenyl rings in naphthalenes. In contrast to Sarlah’s [4 + 2] cycloaddition strategy, in which regioselectivity is governed primarily by steric effects^27,32^, we envisaged taking advantage of the relative electron richness of the phenyl ring to achieve selective dearomative functionalization (Fig. 1d). Photocatalytically promoted single electron transfer (SET) oxidation of quinolines would produce radical cation **I**, in which the positive charge is mostly localized on the phenyl ring. We anticipated nucleophiles to add to intermediate **I** selectively to form radical **II**. Direct arene C(sp^2^)-H functionalization with nucleophiles under photoredox catalysis or electrophotochemical conditions have been elegantly leveraged by Nicewicz^35–38^, Lambert^39^, Hu^40^, and Wickens^41^ to generate aromatic functionalized products. We postulated that it would be possible to direct the reaction toward the less thermodynamically stable dearomatized products by capturing radical **II** with a sufficiently activated hydrogen atom transfer (HAT) agent.

In this work, we describe the successful development of a general chemo- and regioselective method for the dearomative functionalization of diverse fused arenes using this strategy.

## Results and discussion

### Reaction model design

In order to undergo selective photoredox promoted dearomative functionalization, fused arenes must have oxidation potentials that enable them to be oxidized by excited states of photocatalysts (PCs) through SET. The oxidation potential of unsubstituted quinoline (*E*^*ox*^ = 2.23 V vs SCE, Supplementary Fig. 2) suggests that its oxidation would be very difficult using common PCs. However, introduction of an OMe group onto the phenyl moiety of quinoline lowers the oxidation potential sufficiently (e.g., 6-methoxyquinoline **1a**, *E*^*ox*^ = 1.83 V vs SCE, Table 1 and Supplementary Fig. 2), making it possible to participate in thermodynamically driven SETs with excited states of conventional organophotoredox catalysts such as acridinium and triphenylpyrylium (TPT) salts that have excited state reduction potentials (E^*red^) greater than 2.0 V (Table 1).

**Table 1:** Exploration and optimization of reaction conditions


Entry	Variation from the “Standard Conditions”^a^	Yield^b^ (%)
1	None	81, 76^c^ (<5 of **4a**)^b^
2	**Mes-Acr1** instead of **Mes-Acr2**	75
3	**Mes-Acr3** instead of **Mes-Acr2**	67
4	**DCA** instead of **Mes-Acr2**	34
5	**TPT** instead of **Mes-Acr2**	18
6	**4CzIPN** instead of **Mes-Acr2**	trace
7	DCM (0.5 M) with 2,6-lutidine (0.2 equiv)	20
8	DCM (0.2 M) with 2,6-lutidine (0.2 equiv)	49
9	DCM (0.1 M) with 2,6-lutidine (0.2 equiv)	63
10	DCM (0.05 M) with 2,6-lutidine (0.2 equiv)	76
11	DCM (0.025 M) with 2,6-lutidine (0.2 equiv)	74
12	without Ph_3_SiSH	53 (23 of **4a**)^b^
13	without **Mes-Acr2**	trace
14	without light	trace
15	390 nm light	40
16	O_2_ instead of N_2_	trace (56 of **4a**)^b^

^a^Standard conditions: unless otherwise specified, a mixture of 6-methoxyquinoline **1a** (0.3 mmol, 0.05 M), pyrazole **1b** (0.2 mmol), Ph_3_SiSH (0.04 mmol), and **Mes-Acr2** (0.005 mmol) in DCM under a N_2_ atmosphere at room temperature was irradiated with 40 W Kessil blue LEDs for 48 h.

^b^Yield determined by ^1^H NMR spectroscopy using 1,3,5-trimethoxybenzene as an internal standard.

^c^Isolated yield.

### Reaction optimization

We assessed the viability of the proposed dearomative functionalization protocol by reacting 6-methoxyquinoline **1a** with pyrazole **2a** as a nucleophile under irradiation with blue LEDs in DCM solution containing HAT agents and commercially available organic photosensitizers (Table 1). The desired dearomatization product **3a** is generated in the highest yield using 1.5 equiv of **1a**, 1.0 equiv of **2a** (0.05 M), 2.5 mol% *N-*phenylmeso-acridinium tetrafluoroborate (**Mes-Acr2**), and 0.2 equiv of the HAT agent. Nucleophile addition occurred exclusively at the 5-position. The efficiency of the reaction is dependent on the HAT agent (Supplementary Table 1). Among the 17 different thiols and benzeneselenols screened, Ph_3_SiSH was found to be the superior HAT agent, leading to regioselective formation of **3a** in 81% yield (Table 1, entry 1). While photocatalysts such as **TPT** (E*^red^ = 2.55 V vs SCE, 18%, entry 4) with excited state reduction potentials higher than the oxidation potential of **1a** were found to promote this reaction, **Mes-Acr2** (2.5 mol%, E*^red^ = 2.2 V vs SCE) was identified as the best photocatalyst (**3a** formed 81% ^1^H NMR yield, entry 1). Solvent has a significant effect on the reaction (Supplementary Table 2). Furthermore, substrate ratio (Supplementary Table 3) and concentration have a marked impact on the yield (entries 7–11) with low concentrations of **1a** being more beneficial (entries 7 vs 10). The absence of 2,6-lutidine does not affect the reaction outcome (entries 1 vs 10) as quinoline **1a** can serve as the base. Lastly, control experiments confirm that the HAT agent, light and photocatalyst are all required for selective dearomatization. In particular, the HAT agent is critical for minimizing the formation of the aromatization product **4a** (entries 1 vs 12).

#### Dearomatization of quinolines, isoquinolines, and quinoxalines

**Fig. 2:**
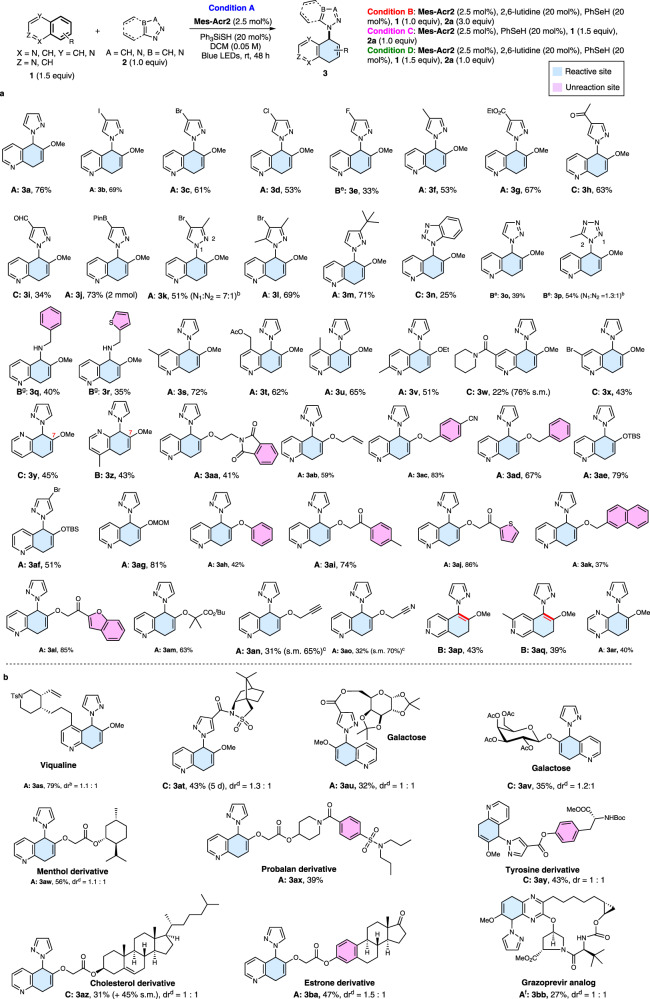
Scope of photoredox dearomative functionalization of quinolines, isoquinolines, and quinoxalines with azoles. **a** Scope of quinolines and quinoxalines with azoles. **b** Late-stage functionalization. ^a^Reaction conditions: unless otherwise specified, see footnote a of Table 1 and supplementary information; ^b^Ratio of isomers was determined by using ^1^H NMR analysis of the crude reaction mixture; ^c^s.m.: starting materials; ^d^dr value was determined by ^1^H NMR analysis of the crude reaction mixture; ^e^Condition B without 2,6-lutidine; ^f^Condition A using 3,6-di-*tert*-butyl-9-mesityl-10-phenylacridin-10-ium tetrafluoroborate as photocatalyst for 5 d. ^g^Condition B but using amines (2.0 equiv) and 3,6-di-*tert*-butyl-9-mesityl-10-phenylacridin-10-ium tetrafluoroborate as photocatalyst for 2 d.

Having established optimal conditions for the photoredox promoted dearomative functionalization reaction, we next explored the scope with fused arenes as substrates and azoles as nucleophiles (Fig. 2a). Our results show that the reaction proceeds with high chemo- and regioselectivity for a wide range of quinolines and azoles. Both electron-donating (**3f**, **3m**) and electron-withdrawing groups are tolerated on the pyrazole. Halogenated pyrazoles reacted with 6-methoxyquinoline with Ph_3_SiSH as the HAT agent (condition A giving **3b**–**3d**). For 5-fluoropyrazole, benzeneselenol, which is 10-fold more reactive than thiols in HAT^42,43^, is required along with 2,6-lutidine (condition B) to yield **3e**. Other electron-deficient pyrazoles also reacted in the presence of benzeneselenol to produce **3h** and **3i** (condition C, without 2,6-lutidine). Notably, the dearomatization method can be applied to a pyrazole boronate, which generates **3j** (73%) containing a useful handle for subsequent coupling reactions. Di- and trisubstituted pyrazoles also reacted smoothly to form **3k** (51%) and **3l** (69%), as did other common azoles including benzotriazole (**3n**), 1,2,3-triazoles (**3o**), and tetrazole (**3p**) under condition B or C. Alkyl amines are also good nucleophiles for the dearomatization of quinolines, generating **3q** (40%) and **3r** (35%).

6-Alkoxy-substituted quinolines are particularly effective substrates for the dearomative functionalization reaction, producing diverse substituted 5,8-dihydroquinolines as products (Fig. 2a). A wide range of alkoxy substituents, including those containing methyl/ethyl (**3s**, **3u**, **3v**, and **3z**), ester (**3t, 3am**), bromo (**3x**), amide (**3w**), phthalimide (**3aa**), alkene (**3ab**), benzyl (**3ac**, **3ad**), TBS (**3ae, 3af**), MOM (**3ag**), phenyl (**3ah**), ketone (**3ai**), alkyne (**3an**), nitrile (**3ac**, **3ao**), thiophene (**3aj**) and benzofuran (**3al**) moieties, are well tolerated. Moreover, the process promotes selective dearomatization and functionalization of quinoline ring systems even when other arene moieties including benzene (**3q**, **3aa**, **3ac**, **3ad**, **3ah, 3ai**), thiophene (**3r**, **3aj**) and benzofuran (**3al**) are present. Notably, the protocol enables highly selective dearomatization of a quinoline (*E*^*ox*^ = 1.86 V vs SCE) over a naphathalene (*E*^*ox*^ = 1.92 V vs SCE) ring (**3ak**) despite their similar oxidation potentials. In addition to controlling chemoselectivity, 6-alkyloxy groups also direct site selective formation of 5-nucleophile-substituted-5,8-dihydroquinolines. Furthermore, dearomatization of 7-methoxyquinolines is also highly regioselective generating only 8-substituted 5,8-dihydroquinoline products (**3y**, **3z**). Isoquinolines can participate in the process (**3ap, 3aq**). However, regioisomeric 7,8-dihydroisoquinolines are formed. Finally, quinoxalines are also viable substrate for the dearomatization process (**3ar**).

Owing to the mild conditions required, the organophotoredox-promoted dearomative functionalization process is applicable to late-stage functionalizations of complex pharmaceutically relevant quinoline structures including viqualine (**3as**), Oppolzer’s camphorsultam (**3at**), galactose (**3au**, **3av**), menthol (**3aw**), probalan (**3ax**), tyrosine (**3ay**), cholesterol (**3az**), and estrone (**3ba**) groups (Fig. 2b). It is particularly impressive that a grazoprevir analog (**3bb**) containing various functional groups was selectively dearomatized utilizing this process. These results underscore the mildness, high chemo- and regioselectivity, and practicality of this protocol.

#### Dearomatization of fused arenes

**Fig. 3:**
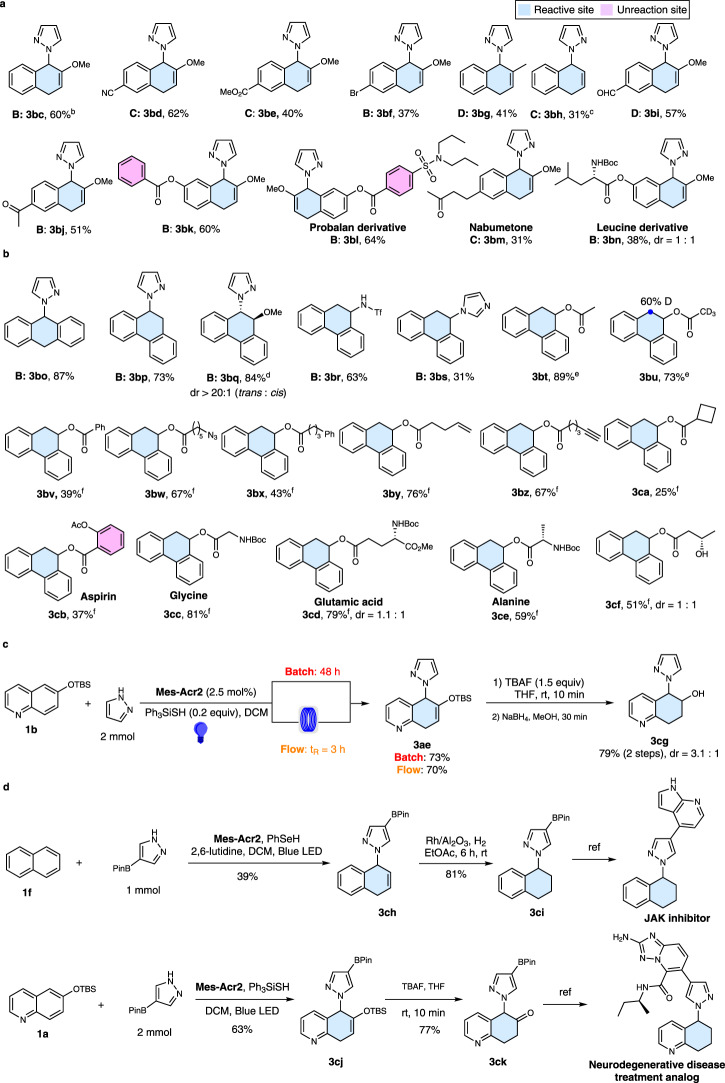
Dearomatization of fused arenes. **a** Scope of naphathalenes. **b** Scope of anthracenes and phenanathrenes. **c** Large scale synthesis. **d** Application in synthesis of valuable targets. ^a^Reaction condition: unless specified, see footnote a of Table 1 and the ESI; ^b^Ratio of 6-methoxynaphthalene to pyrazole: 1: 2; ^c^Ratio of naphthalene to pyrazole is 2:1; ^d^Reaction time: 4 d; ^e^Condition B using 10.0 equiv of acid; ^f^Condition B using 5.0 equiv of acid.

Encouraged by the success of the photoredox reaction of quinolines, we explored its utility for the selective dearomatization of fused non-heteroaromatics. The results show that selective dearomatization of fused arenes is more challenging. As with quinolines, electron-donating substituents can be used to lower the oxidation potentials and enhance reactivity. A 2-methoxy substituent in naphthalene, for instance, lowers the oxidation potential to 1.8 V (vs SCE, supplementary Fig. 2) and enables selective oxidation by the excited state of photocatalyst **Mes-Acr2**. Selective dearomatization and C-1 functionalization of 2-methoxynaphthalene by pyrazole using condition B led to **3bc** (Fig. 3a). The efficiency of this process is also dependent on the base and the HAT agent, as are shown in **3bd**, **3be**, and **3bm** (condition C), **3bf, 3bj, 3bk, 3bl**, and **3bn** (condition B) and **3bi** (condition D). The dearomatization strategy can be applied to methyl substituted naphthalene (**3bg**). Finally, unsubstituted naphthalene (**3bh**) also participates in the process to produce the 1-substituted product regioselectively.

UV-mediated photochemcial dearomative functionalization of naphthalene and phenanthrene with excess amines and cyanide (10–25 equiv) were reported by Yasuda and Pac in 1970s and 80s^44–47^. However, these classic methods showed very limited substrate scopes and poor functional group tolerance due to the harsh activation method. Furthermore, it is challenging to employ these methods for dearomatization of fused heteroaromatic systems such as quinoline, isoquinoline, and quinaxoline because they cannot be activated by UV light. In contrast, our mild, visible-light-mediated photoredox reaction can be applied to the dearomative functionalization of polycyclic aromatic hydrocarbons with a broad substrate scope, in addition to highly-valued fused heteroaromatics. High yields with azoles, which are challenging nucleophiles in Yasuda and Pac’s UV method, are obtained for simple anthracene (**3bo**, 87%) and phenanthrene (**3** **bp**, 73%) (Fig. 3b). Notably, 6-methoxyphenanthrene reacts to form the *trans* substitution product **3bq** (84%). Moreover, nucleophiles other than azoles, such as trifluoromethanesulfonamide (**3br**) and imidazole (**3bs**), can also participate in the process. Furthermore, carboxylic acids, which are often used as radical precursors in photoredox reactions^48^, serve as effective nucleophiles for dearomative functionalization of phenanthrenes. These reactions proceed in moderate to high yields (**3bt**–**3cf**, 25–89%) and display broad functional group tolerance including phenyl (**3bv**, **3bx**), azide (**3bw**), alkenyl (**3by**), alkynyl (**3bz**), cyclobutyl (**3ca**), and hydroxyl acid groups (**3cf**). Due to their weaker nucleophilicity and steric effects, benzoic acid (**3cb**) and secondary carboxylic acids (**3ca**) are less effective nucleophiles for this process. In addition, the reaction can be utilized for the direct modification of biologically relevant structures such as aspirin (**3cb**) and amino acids (**3cc–3ce**).

### Synthetic applications

Studies with the silyloxyquinoline **1b** show that the synthetic protocol can be scaled up without loss of yield (Fig. 3c). Moreover, the reaction can be adapted for use in a flow system, an emerging technology in organic synthesis^49^. Using the flow system, the reaction time is reduced dramatically to 3 h without compromising the yield. The product silylenol ether **3ae** can be converted to the corresponding alcohol **3cg**. The preparative power of this method was also demonstrated by its use in the cost-effective synthesis of important biologically active targets, including a JAK inhibitor (Fig. 3d)^50^. Our synthetic route starts from naphthalene ($0.04/g) instead of the more expensive 1-bromo-1,2,3,4-tetrahydronaphthalene ($370/g) used in the earlier preparative pathway^50^. Dearomatization of naphthalene with pyrazol-4-ylboronic acid pinacol ester produces key intermediate **3ch**, which is then reduced using Rh/Al_2_O_3_ catalyzed hydrogenation to form **3ci**. The JAK inhibitor is then readily prepared from **3ci** using reported procedures^50^. For synthesis of the compound with neurodegenerative activity shown in Fig. 3d, our protocol employed 6-hydroxyquinoline ($1.6/g) as starting material, which is much cheaper than the previously used precursor, 5-hydroxy-5,6,7,8-tetrahydroquinoline ($416/g)^51^. These studies clearly demonstrate the synthetic value of the our dearomative functionalization protocol, which provides direct and efficient access to polycyclic partially dearomatized frameworks that were previously inaccessible or required strenuous synthetic efforts.

#### Mechanistic studies

**Fig. 4:**
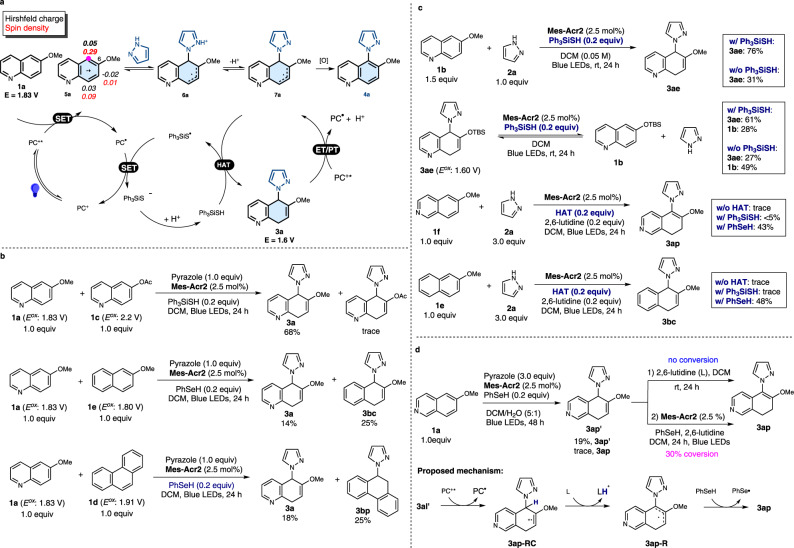
Mechanistic studies. **a** Proposed catalytic cycle. **b** Chemoselectivity studies. **c** Study of roles of HAT reagent. **d** Study of regioselectivity of isoquinoline engaged reaction.

To gain insight into the reaction mechanism, we conducted additional experimental and computational studies. The excited state PC^+^* Mes-Acr2* possesses strong oxidizing power (*E*^red^* = 2.2 V) and can oxidize the 6-methoxyquinoline (**1a**) (*E*^ox^ = 1.83 V vs SCE, supplementary Fig. 2) to give radical cation **5a** (Fig. 4a and Stern-Volmer quenching experiments, supplementary Figs. 3 and 4). The oxidation potential of the fused arene substrate is crucial for the success of this process, as demonstrated by the high reactivity of **1a** in contrast to the lack of reactivity of unsubstituted quinoline (*E*^*ox*^ = 2.23 V vs SCE, Supplementary Fig. 2). In addition, when a mixture of **1a** and 6-acetoxyquinoline (**1c**) (*E*^ox^ = 2.2 V vs SCE, supplementary Fig. 2) is subjected to the reaction conditions, only **1a** undergoes selective dearomatization to form **3a**, indicating that electron-withdrawing substituents inhibit the reaction. In contrast, **1a** and 2-methoxynaphthalene (**1e**, *E*^ox^ = 1.8 V vs SCE, Supplementary Fig. 2), which have similar oxidation potentials, both react under the same conditions to produce the corresponding dearomatized products **3a** and **3bc** (Fig. 4b). Likewise, phenanthrene **1d** (*E*^*ox*^ = 1.91 V vs SCE, Supplementary Fig. 2) is an effective substrate for this process (Fig. 4b). Notably, the pyrazole nucleophile (*E*^*ox*^ = 2.15 V vs SCE, Supplementary Fig. 2) is not oxidized by Mes-Acr2*. This is supported by luminescence quenching study of **Mes-Acr2** with varying concentrations of 6-methoxyquinoline **1a** and pyrazole **2a** (Supplementary Fig. 4).

**Fig. 5:**
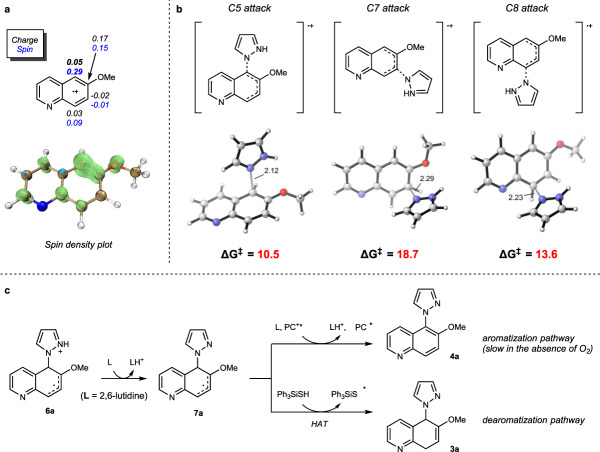
Computational investigations. **a** Calculated Hirshfeld charge and spin densities of radical cation **5a** at the ωB97X-D/def2-TZVPP, SMD (CH_2_Cl_2_)//ωB97X-D/def2-SVP level of theory. **b** Calculated C–N forming transition states for the reaction between **5a** and pyrazole at the ωB97X-D/def2-TZVPP, SMD (CH_2_Cl_2_)//ωB97X-D/def2-SVP level of theory. Free energies are in kcal/mol, and interatomic distances are in Å. **c** Possible fates of radical cation **6a**.

Following the oxidation of **1a** through SET, pyrazole reacts with the electrophilic radical cation **5a** to form the radical cation **6a** (Fig. 4a). Calculated Hirshfeld charge and spin densities (Fig. 5a; see Supplementary Fig. 6 for calculated charge and spin densities for other substrates) indicate that C5 has the highest spin density (bold face) in **5a** and is therefore the preferred site for nucleophilic addition. The calculated free energies of the key transition states also support the observation that pyrazole attack at the C5 position is kinetically favored over C8 or C7 by at least 3 kcal/mol (Fig. 5b).

Subsequent exothermic deprotonation of **6a** by 2,6-lutidine gives a neutral radical **7a** (Fig. 5c). Calculated free energies suggest that sequential SET oxidation and deprotonation of **7a** to form the aromatic substitution product **4a** is more thermodynamically favorable (−62.5 kcal/mol from **7a** with the excited state PC^+^* Mes-Acr2* as the oxidant, Figs. 5c and S7). This is reflected in the outcomes of processes leading to fully aromatic products developed by Nicewicz^35–38^, Lambert^39^, Hu^40^ and Wickens^41^. However, the aromatization pathway requires an oxidant (such as the excited-state photocatalyst or oxygen) to be present in sustained concentrations. Because this pathway does not regenerate the photocatalyst, the aromatization process has been previously noted to be slow in the absence of oxygen, despite its favorable thermodynamics.

Given the apparent hurdles in the aromatization pathway, we reasoned that the presence of a reactive HAT agent (e.g., Ph_3_SiSH) could intercept **7a** and redirect the course of the reaction toward the dearomatized product **3a** rather than the more stable aromatic product **4a** (Fig. 5c). We elected to employ thiol and selenol HAT agents that have weak S-H and Se-H bonds (S-H: 82 kcal/mol, Se-H: 73 kcal/mol) and correspondingly high H-atom transfer rates (PhSH: K_20_ = 9.0 × 10^7 ^M^−1^S^−1^ and PhSeH: K_20_ = 1.3 × 10^9 ^M^−1^S^−1^)^42,43,52^. Indeed, the addition of the HAT agent had a marked effect on the yield of the dearomatized product. Control experiments showed that in the absence of the HAT agent Ph_3_SiSH, the yield of the dearomatized product **3ae** dropped from 76% to 31% (Fig. 4c). The effect of the HAT agent is particularly noticeable in reactions of isoquinolines and naphthalenes with pyrazole. Only when PhSeH is present do these processes produce the desired products **3ap** and **3bc** (Fig. 4c). In these cases, the HAT process might have been further facilitated by the polarity match^48^ between the protic S-H/Se-H and the nucleophilic carbon radical **7a**. Calculations on the HAT step also confirm that PhSeH decreases the barrier of the HAT step by about 9 kcal/mol relative to Ph_3_SiSH (Supplementary Fig. 7). After the HAT step, the Ph_3_SiS^•^ radical is reduced to the Ph_3_SiS^−^ anion by the PC^•^ **Mes-Acr2** radical. Protonation of the Ph_3_SiS^−^ anion regenerates both the HAT agent Ph_3_SiSH and the ground-state photocatalyst **Mes-Acr2** (Fig. 4a). Further computational efforts are underway in our laboratories to elucidate the full mechanistic picture (see discussion accompanying Supplementary Fig. 8).

In addition to promoting the formation of the dearomatized product **3a**, the HAT agent Ph_3_SiSH also retards the conversion of **3a** (*E*^*ox*^ = 1.60 V vs SCE, Supplementary Fig. 2) back to the starting material **1a** via PC^+^*(Mes-Acr2*)-mediated oxidation under the standard reaction conditions (Fig. 4c). Specifically, preliminary experiments show that the photoinduced reaction of **3a** in the presence of Ph_3_SiSH leads to 61% recovery of **3ae** and formation of 28% of **1b**. In contrast, in the absence of the HAT agent, **1b** was produced in 49% yield.

It is also worth noting that the dearomatization pathway selectively produced the 5,8-dihydroquinoline regioisomer **3a**. We did not observe the formation of the 7,8-dihydroquinoline **3a’**, which was calculated to be 3.8 kcal/mol less stable than **3a** due to the steric repulsion between the alkene substituents (Supplementary Fig. 8). However, reactions between isoquinolines and pyrazole yielded the regioisomeric 7,8-dihydroisoquinolines. Our experiments suggest that the reaction initially forms 5,8-dihydroisoquinoline **3ap’**, which is then converted to the more stable 7,8-dihydroisoquinoline **3ap** (Fig. 4d). The 5,8-dihydroisoquinoline **3ap’** was obtained in the presence of H_2_O. Two possible pathways could lead to 5,8-dihydroisoquinoline **3ap**. The deprotonation/C=C isomerization pathway was ruled out as we did not observe the formation of **3ap** when **3ap’** was treated with 2,6-lutidine, a base used in the dearomatization process. In contrast, **3ap** was formed under the photoredox reaction conditions through SET oxidation of the C=C bond, deprotonation and HAT processes. This pathway was also supported by calculated reaction energies (Supplementary Scheme 1).

In this work, we develop a conceptually unique organophotoredox catalytic dearomative functionalization strategy for the selective disruption of aromaticity in fused arenes. A reactive HAT agent is utilized to help direct the reaction toward the dearomatized product over the thermodynamically more favorable aromatizatized product. The preparative power of the protocol is demonstrated by applications to structurally diverse fused arenes including quinolines, isoquinolines, quinoxalines, naphthalenes, anthracenes and phenanthrenes. The method can also be applied to the synthesis and late-stage skeletal editing of complex pharmaceutically valued structures. We anticipate that this process will enable facile access to a wide range of synthetically versatile frameworks and accelerate the construction of new molecular architectures for drug discovery.

## Methods

### Representative procedures for the dearomatization of heteroarenes

To an oven-dried 20 mL-Schlenk tube equipped with a stir bar, was added pyrazole (0.2 mmol), quinoline derivatives (0.3 mmol), Ph_3_SiSH (0.04 mmol), and **Mes-Acr2** (0.005 mmol). The tube was evacuated and back-filled with N_2_ for three times, then sealed with rubber stopper and parafilm. Subsequently, the degassed dichloromethane (4 mL) was added. The reaction was irradiated by the two 40 W Kessil Blue LEDs, cooling by the electronic fan. After the completion of reactions (usually 48 h), the resulted solution was purified by flash column chromatography on silica gel eluting with hexane/ethyl acetate or DCM/ethyl acetate in indicated ratio.

### Representative procedures for the dearomatization of arenes

To an oven-dried 20 mL-Schlenk tube equipped with a stir bar, was added pyrazole (0.6 mmol), naphthalene derivatives (0.2 mmol), PhSeH (0.04 mmol), **Mes-Acr2** (0.005 mmol), 2,6-lutidine (0.04 mmol) as the base, will be added. The tube was evacuated and back-filled with N_2_ for three times, then sealed with rubber stopper and parafilm. Subsequently, the degassed dichloromethane (4 mL) was added. The reaction was irradiated by the two 40 W Kessil Blue LEDs, cooling by the electronic fan. After the completion of reactions, the resulted solution was purified by flash column chromatography on silica gel eluting with hexane/ethyl acetate or DCM/ethyl acetate in the indicated ratio.

## Supplementary information

Supplementary Information

Description of Additional Supplementary Files

Supplementary Data 1

## Data Availability

Experimental procedures and characterization data are given in the Supplementary Information. The authors declare that all other data supporting the findings of this study are available within the article and its Supplementary Information files. For Cartesian coordinates, see Supplementary Data 1 file. Further data can be obtained from the corresponding author upon request.
