# Supplementary material for: Selective skeletal editing of polycyclic arenes using organophotoredox dearomative functionalization
Source: Nat Commun. 2022 Aug 5;13:4565. doi: 10.1038/s41467-022-32201-7 (PMC9355940; doi:10.1038/s41467-022-32201-7)
Supplement: Supplementary file 1 — Supplementary Information [file 41467_2022_32201_MOESM1_ESM.pdf]

Supplementary Information

Selective Skeletal Editing of Polycyclic Arenes Using Organophotoredox Dearomative Functionalization

Peng Ji,[1] Cassondra C. Davies,[2] Feng Gao,[1] Jing Chen,[1] Xiang Meng,[1] Kendall N. Houk,[3]* Shuming Chen[2]* and Wei Wang[1]*

[1]*Departments of Pharmacology and Toxicology and Chemistry and Biochemistry, University of Arizona, Tucson, AZ 85721-0207, USA*
[2]*Department of Chemistry and Biochemistry, Oberlin College, Oberlin, OH 44074, USA*
[3]*Department of Chemistry and Biochemistry, University of California, Los Angeles, CA 90095-1569, USA*

List of Contents

1. Supplementary Methods:

1.1 General Information ---S2

1.2 Experiment Procedures and Optimization of Reaction Conditions -----------------------S2

1.3 Reaction setup and Cyclic Voltammetry Measurements -----------------------------------S6

1.4 Substrate Synthesis ---S9

1.5 Large Scale of Synthesis of Compound **3cc** and **3ce** -------------------------------------S17

1.6 DFT Calculations---S20

1.7 Compounds Characterization Data --S25

2. Supplementary Figure --S61

3. Supplementary References---S161

1. Supplementary Methods

1.1 General Information

All commercially available reagents were purchased from Sigma Aldrich, Ark Pharm Inc., Matrix Chemical, Combi-Blocks, AKSci, Alfa Aesar, Acros, Ambeed or TCI America, and used as received unless otherwise noted. Merck 60 silica gel was used for chromatography, and Whatman silica gel plates with a fluorescence F254 indicator were used for thin-layer chromatography (TLC) analysis. **^{1}H** and **^{13}C NMR** spectra were recorded on Bruker Avance 500 MHz or Varian 400 MHz. Chemical shifts in **^{1}H NMR** spectra are reported in parts permillion (ppm) relative to residual chloroform (7.26 ppm) or dimethyl sulfoxide (2.50 ppm) as internal standards. ^{1}H NMR data are reported as follows: chemical shift (δ ppm), multiplicity (s = singlet, d = doublet, quint = quintet, sext = sextet, m = multiplet, br = broad), coupling constant in Hertz (Hz) and hydrogen numbers based on integration intensities. **^{13}C NMR** chemical shifts are reported in ppm relative to the central peak of $CDCl_3$ (77.16 ppm) or DMSO-d_6 (39.52 ppm) as internal standards. High-resolution mass spectrometry was performed in Analytical and Biological Mass Spectrometry Center. Both blue LED strip and 40 W Kessil Blue LEDs were purchased from Amazon. Cyclic voltammetry was performed at 25 °C on a CH Instrument CHI604xD electrochemical analyzer using a glassy carbon working electrode, a platinum wire counter electrode, and the Ag/AgCl reference electrode calibrated using ferrocene redox couple (4.8 V below vacuum). HPLC analyses with chiral stationary phase were carried out using a SHIMADZU HPLC system with SPD-20A detector. Samples are purified by flash column chromatography on Teledyne ISCO CombiFlash NextGen300+.

1.2. General Procedures and Optimization of Reaction Conditions

1.2.1 <u>General procedure for Condition A</u>

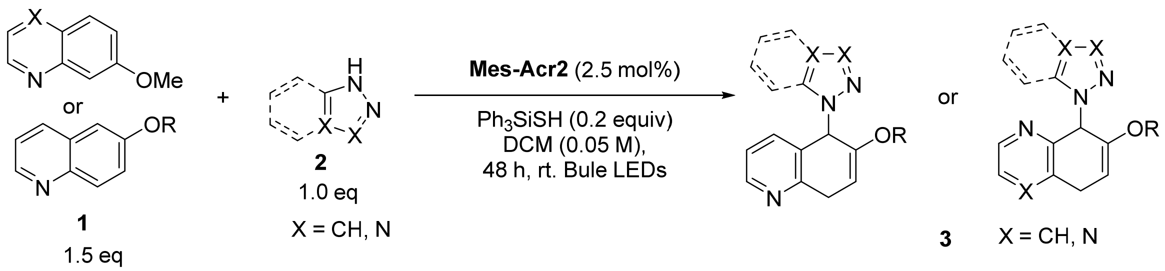

To an oven-dried 20 mL-Schlenk tube equipped with a stir bar, was added pyrazole (0.2 mmol), quinoline derivatives (0.3 mmol), Ph_3SiSH (0.04 mmol), and **Mes-Acr2** (0.005 mmol). The tube was evacuated and back-filled with N_2 for three times, then sealed with rubber stopper and parafilm. Subsequently, the degassed dichloromethane (4 mL) was added. The reaction was irradiated by the two 40 W Kessil Blue LEDs, cooling by the electronic fan (Figure S1). After the completion of reactions (usually 48 h), the resulted solution was purified by flash column chromatography on silica gel eluting with hexane/ethyl acetate or DCM/ethyl acetate in indicated ratio.

1.2.2 General procedure for Condition B/C/D

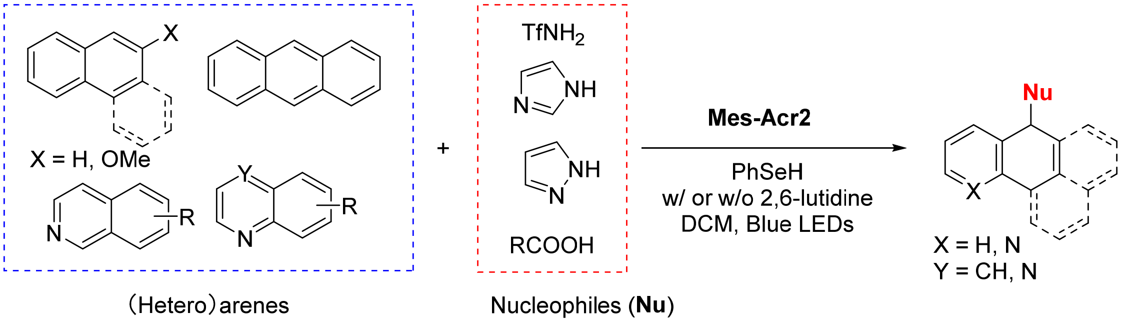

Condition B: To an oven-dried 20 mL-Schlenk tube equipped with a stir bar, was added pyrazole (0.6 mmol), quinoline derivatives (0.2 mmol), PhSeH (0.04 mmol), **Mes-Acr2** (0.005 mmol), 2,6-lutidine (0.04 mmol) as the base, will be added. The tube was evacuated and back-filled with N_2 for three times, then sealed with rubber stopper and parafilm. Subsequently, the degassed dichloromethane (4 mL) was added. The reaction was irradiated by the two 40 W Kessil Blue LEDs, cooling by the electronic fan (Figure S1). After the completion of reactions, the resulted solution was purified by flash column chromatography on silica gel eluting with hexane/ethyl acetate or DCM/ethyl acetate in the indicated ratio.

Condition C: Similar to condition B, a mixture of the quinoline derivatives (0.3 mmol), pyrazole (0.2 mmol), PhSeH (0.04 mmol), and **Mes-Acr2** (0.005 mmol) in DCM (0.05 M) was irradiated with 40 W Kessil blue LEDs in a N_2 atmosphere at room temperature for 48 h.

Condition D: Similar to condition B, a mixture of the quinoline derivatives (0.3 mmol), pyrazole (0.2 mmol), PhSeH (0.04 mmol), **Mes-Acr2** (0.005 mmol), and 2,6-lutidine (0.04 mmol) as the base in DCM (0.05 M) was irradiated with 40 W Kessil blue LEDs in a N_2 atmosphere at room temperature for 48 h.

1.2.3 Optimization of reaction conditions:

Supplementary Table 1. Effect of different hydrogen donors on reaction efficiency

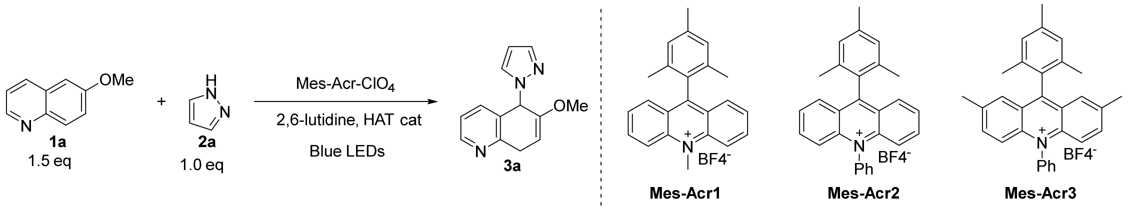

PC (5 mol%)	Base (20 mol%)	HAT cat (20 mol%)	Solvent (0.2 M)	Time	Yield[a] (%)
**Mes-Acr1**	2,6-lutidine	PhSH	DCE	24 h	37
**Mes-Acr1**	2,6-lutidine	PhSeH	DCE	24 h	40
**Mes-Acr1**	2,6-lutidine	TRIP-SH	DCE	24 h	30
**Mes-Acr1**	2,6-lutidine	*i*Pr_3SiSH	DCE	24 h	39, 44 (48 h)
**Mes-Acr-1**	2,6-lutidine	4-MeOPhSH	DCE	24 h	16
**Mes-Acr1**	2,6-lutidine	Ph_3SiSH	DCE	24 h	40, 45 (48 h)
**Mes-Acr1**	2,6-lutidine	4-NO_2PhSH	DCE	24 h	41
**Mes-Acr1**	2,6-lutidine	5F-PhSH	DCE	24 h	26
**Mes-Acr1**	2,6-lutidine	CPA-SH	DCE	24 h	12
**Mes-Acr1**	2,6-lutidine	Adam-SH	DCE	24 h	34, 44 (48 h)
**Mes-Acr1**	2,6-lutidine	PhCOSH	DCE	24 h	31
**Mes-Acr1**	2,6-lutidine	5OAcGlu-SH	DCE	24 h	38
**Mes-Acr1**	2,6-lutidine	Methyl Thioglycolate	DCE	24 h	35
**Mes-Acr1**	2,6-lutidine	tBuSH	DCE	24 h	34
**Mes-Acr1**	2,6-lutidine	Cysteine	DCE	24 h	37
**Mes-Acr1**	2,6-lutidine	Ph_3SiSH	DCE	24 h	33[b]
**Mes-Acr1**	2,6-lutidine	Methyl thiosalicylate	DCE	24 h	37

[a]The yields of **3a** are determined by ^1H NMR using 1,3,5-trimethoxybenzne as internal reference. [b]The concentration is 0.5 M.

Supplementary Table 2. Effect of different solvent on the reaction efficiency

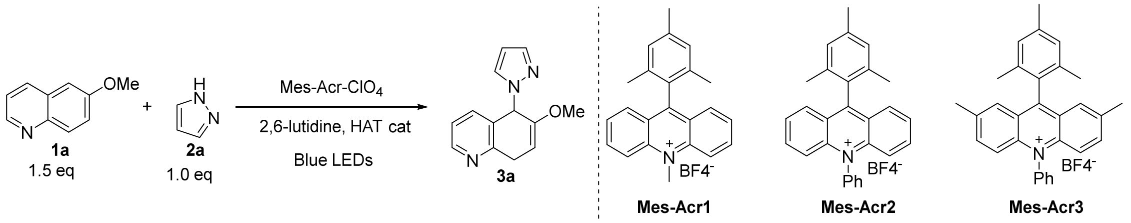

PC (2.5 mol%)	Base (20 mol%)	HAT cat (20 mol%)	Solvent (0.2 M)	Time	Yield[a] (%)
**Mes-Acr1**	2,6-lutidine	Ph_3SiSH	$PhCF_3$	48 h	35
**Mes-Acr1**	2,6-lutidine	Ph_3SiSH	$CHCl_3$	48 h	44
**Mes-Acr1**	2,6-lutidine	Ph_3SiSH	ACN	48 h	5
**Mes-Acr1**	2,6-lutidine	Ph_3SiSH	DMF	48 h	trace
**Mes-Acr1**	2,6-lutidine	Ph_3SiSH	DCE/H_2O (5:1, v/v)	48 h	37

[a]The yields of **3a** are determined by ^{1}H NMR using 1,3,5-trimethoxybenzne as internal reference.

Supplementary Table 3. Ratio of quinoline/pyrazole on the reaction efficiency.

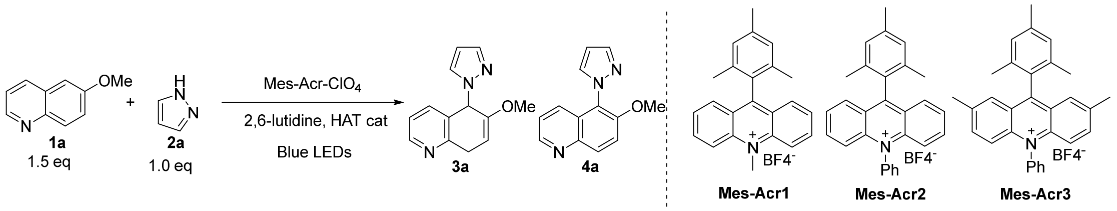

PC (2.5 mol%)	Base (20 mol%)	HAT cat (20 mol%)	Solvent	Time	Yield[a] (%)	Ratio of 1a:2a
**Mes-Acr1**	-	Ph_3SiSH	DCM (0.025 M)	48 h	85	5:1
**Mes-Acr1**	-	Ph_3SiSH	DCM (0.05 M)	48 h	82	2.5 : 1
**Mes-Acr1**	-	Ph_3SiSH	DCM (0.05 M)	48 h	72	2 : 1
**Mes-Acr1**	-	Ph_3SiSH	DCM (0.05 M)	48 h	75	1.5 : 1
**Mes-Acr2**	-	Ph_3SiSH	DCM (0.05 M)	48 h	81, 76[b] (<5 of **4a**)	1.5 : 1
**Mes-Acr3**	-	Ph_3SiSH	DCM (0.05 M)	48 h	67	1.5 : 1
**Mes-Acr2**	-	Ph_3SiSH	DCM (0.05 M)	48 h	48	1 : 2
**Mes-Acr1**	2,6-lutidine	Ph_3SiSH	DCE (0.025 M)	48 h	41	2.5:1

[a]The yields of **3a** are determined by crude ^{1}H NMR using 1,3,5-trimethoxybenzne as internal reference. [b]The yield is isolated yield.

Supplementary Table 4. Different HAT donor effect on the reaction efficacy under optimal conditions.

PC (2.5 mol%)	Base (20 mol%)	HAT cat (20 mol%)	Solvent	Time	Yield[a] (%)	Ratio of 1a:2a
**Mes-Acr2**	-	Ph_3SiSH	DCM (0.05 M)	48 h	81, 76[b] (<5 of **4a**)	1.5 : 1
**Mes-Acr2**	-	PhSeH	DCM (0.05 M)	48 h	62	1.5 : 1
**Mes-Acr2**	-	4-NO_2PhSH	DCM (0.05 M)	48 h	62	1.5 : 1
**Mes-Acr2**	-	2-aminoPhSH	DCM (0.05 M)	48 h	45	1.5 : 1
**Mes-Acr2**	-	PhSH	DCM (0.05 M)	48 h	70	1.5 : 1
**Mes-Acr2**	-	TRIPSH	DCM (0.05 M)	48 h	64	1.5 : 1
**Mes-Acr2**	-	*i*Pr_3SiSH	DCM (0.05 M)	48 h	74	1.5 : 1
**Mes-Acr2**	-	Adam-SH	DCM (0.05 M)	48 h	77	1.5 : 1
**Mes-Acr2**	-	PhCOSH	DCM (0.05 M)	48 h	13	1.5 : 1
**Mes-Acr2**	-	5-AcOGluSH	DCM (0.05 M)	48 h	76	1.5 : 1
**Mes-Acr2**	-	Cysteine	DCM (0.05 M)	48 h	72	1.5 : 1
**Mes-Acr2**	-	4MeOPhSH	DCM (0.05 M)	48 h	63	1.5 : 1
**Mes-Acr2**	-	Methyl Thioglycolate	DCM (0.05 M)	48 h	73	1.5 : 1
**Mes-Acr2**	-	Methyl thiosalicylate	DCM (0.05 M)	48 h	74	1.5 : 1

[a]The yields of **3a** are determined by ^{1}H NMR using 1,3,5-trimethoxybenzne as internal reference. [b]The yield is isolated yield.

1.3. Reaction setup and measurement of redox potential

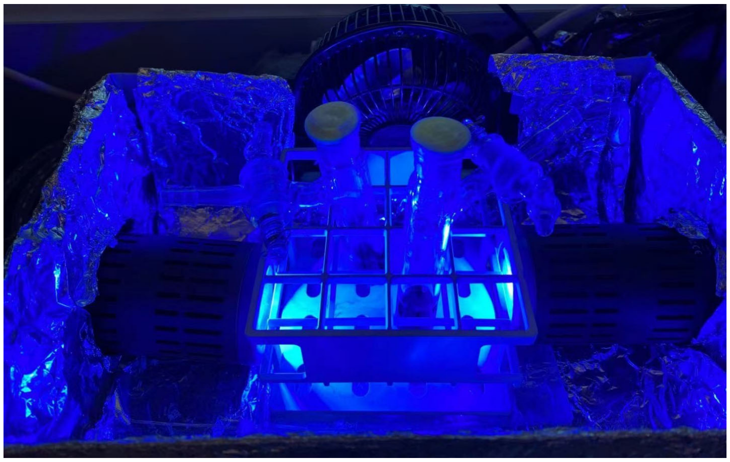

Supplementary Figure 1. Reaction Setup.

Procedure for measurement of the redox potential:

Voltammetric measurements were recorded on a CH Instruments: Model 600E Series Electrochemical Analyzer using a standard three electrodes setup in dry and degassed MeCN (10 mL), with ferrocene as an internal reference ($E^0_{1/2}$ = + 0.40 V vs SCE) and nBu$_4$NPF$_6$ as the electrolyte (0.10 mmol). Cyclic voltammograms were recorded at a scan rate of 0.2 V/s.

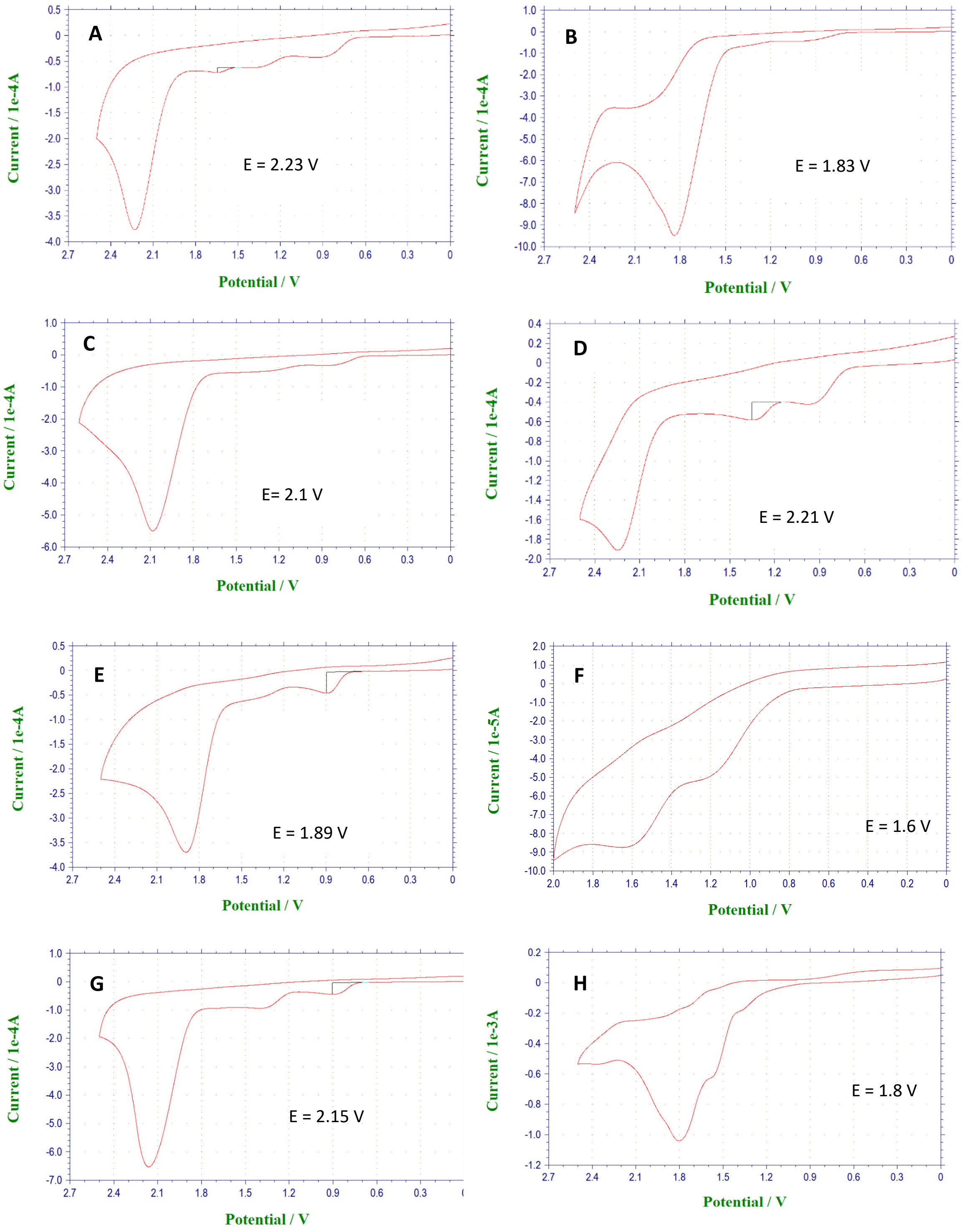

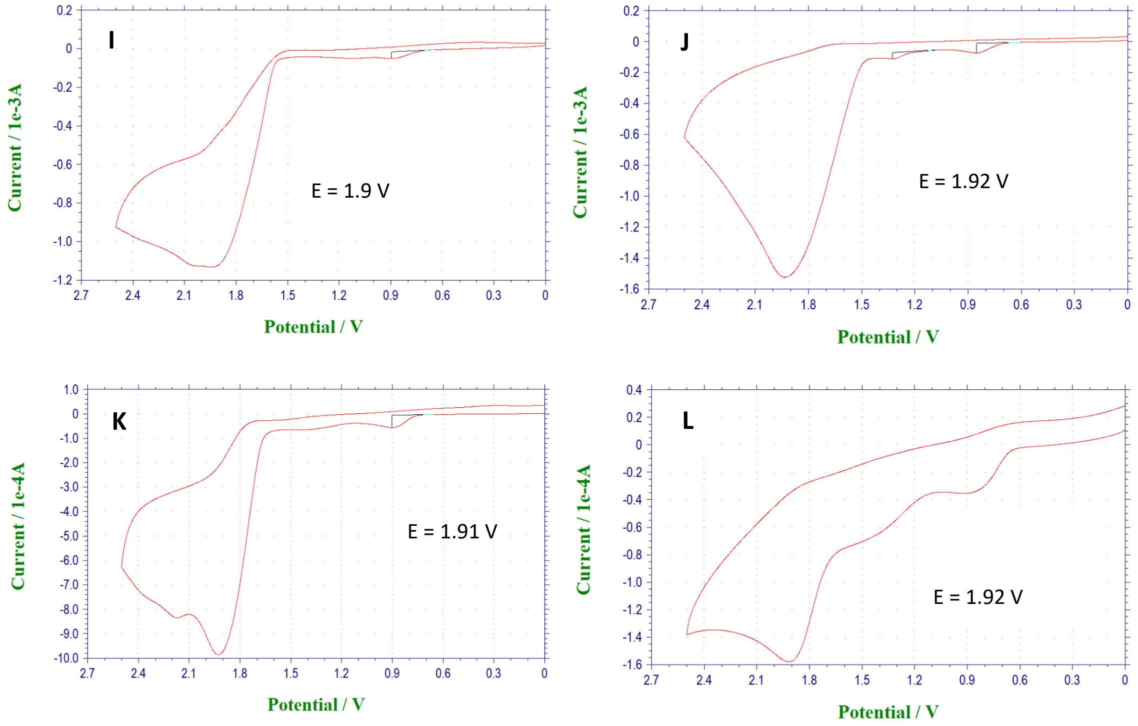

Supplemenatry Figure 2. The cyclic voltammogram (CV) of (hetero)arenes and pyrazole vs SCE in dry acetonitrile at 0.1 V/s. The results indicated that the (hetero)arenes are more easy to be oxidized than the pyrazole. **A**, CV of quinoline. **B**, CV of 6-methoxyquinoline. **C**, CV of 6-methylquinoline. **D**, CV of 6-acetyloxyquinoline. **E**, CV of 6-OTBS-quinoline. **F**, CV of 6-((*t*-butyldimethylsilyl)oxy)-5-(1H-pyrazol-1-yl)-5,8-dihydroquinoline. **G**, CV of pyrazole. **H**, CV of 2-methoxynaphthalene. **I**, CV of naphthalene. **J**, CV of 2-methylnaphthalene. **K**, CV of phenanthrene. **L**, CV of 6-methoxyisoquinoline.

Stern-Volmer Quenching Experiments

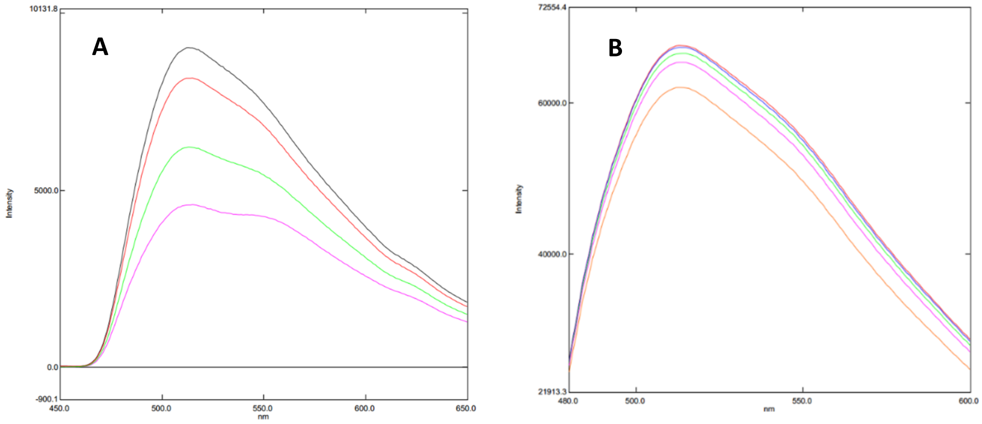

Supplementary Figure 3. Emission Curve of Quenching experiment. Stern-Volmer quenching experiments were conducted on a Shimadzu Fluorescence Spectrophotometer. Stern-Volmer luminescence quenching experiments were run with freshly prepared solutions

of 0.02 mM **Mes-Acr2** and the appropriate amount of quencher in dry DCM at room temperature. The solutions were irradiated at 450 nm, and the emission spectra of the samples were collected. **A**, quinoline and **Mes-Acr2**. **B**, pyrazole and **Mes-Acr2**.

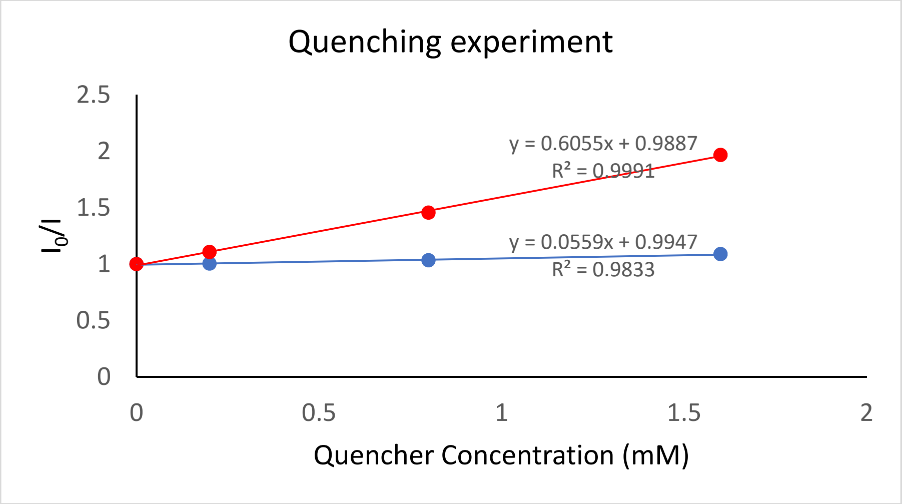

Supplementary Figure 4. Luminescence quenching of **Mes-Acr2** with varying concentrations of 6-methoxyquinoline **1a** (red) and pyrazole **2a** (blue) in DCM. The results indicated that the **Mes-Acr2** will be quenched by the quinoline first.

1.4. Substrates Synthesis

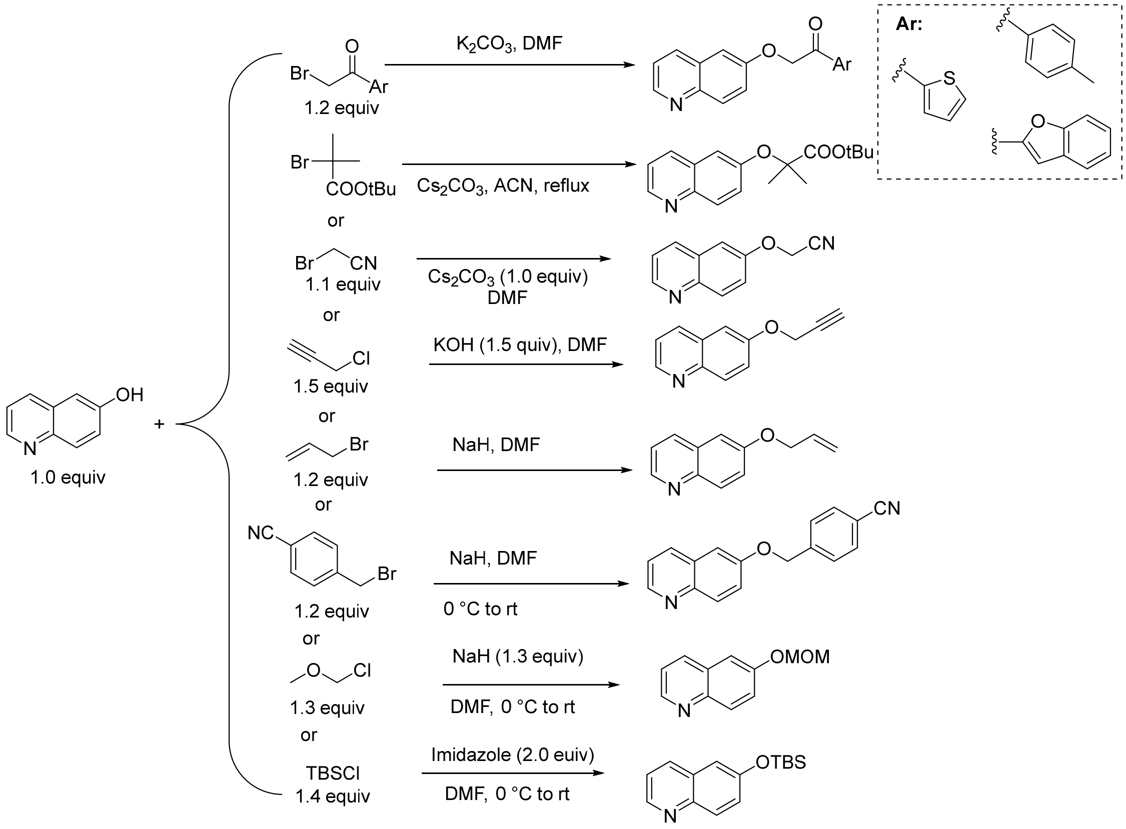

General procedure: The mixture of 6-hydroxyquinoline (1.0 equiv), the diverse halogenated chemicals (1.2 equiv), and base (K_2CO_3/Cs_2CO_3/NaH/KOH/imidazole (1.0-2.0 equiv)) were dissolved in DMF or acetonitrile at room temperature overnight. The mixture was quenched by H_2O and then extracted by ethyl acetate. The obtained organic phase was removed to give crude product, which was purified using hexane/ethyl acetate as eluent.

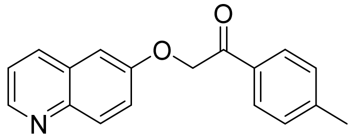

2-(quinolin-6-yloxy)-1-(p-tolyl)ethan-1-one:
^{1}H NMR (500 MHz, $CDCl_3$) δ 8.78 (dd, *J* = 4.4, 1.7 Hz, 1H), 8.02 (t, *J* = 8.1 Hz, 2H), 7.98 – 7.81 (m, 2H), 7.49 (dd, *J* = 9.3, 2.8 Hz, 1H), 7.41 – 7.29 (m, 3H), 7.06 (d, *J* = 2.8 Hz, 1H), 5.38 (s, 2H), 2.45 (s, 3H).

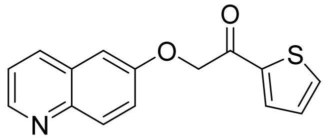

2-(quinolin-6-yloxy)-1-(thiophen-2-yl)ethan-1-one:
^{1}H NMR (500 MHz, $CDCl_3$) δ 8.79 (dd, *J* = 4.2, 1.8 Hz, 1H), 8.23 – 7.91 (m, 3H), 7.73 (d, *J* = 4.9 Hz, 1H), 7.50 (dt, *J* = 9.2, 2.3 Hz, 1H), 7.35 (dd, *J* = 8.4, 4.2 Hz, 1H), 7.18 (t, *J* = 4.4 Hz, 1H), 7.09 (d, *J* = 2.8 Hz, 1H), 5.23 (d, *J* = 1.8 Hz, 2H).

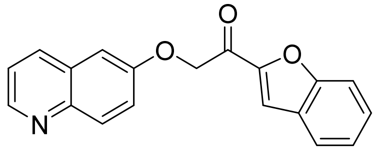

1-(benzofuran-2-yl)-2-(quinolin-6-yloxy)ethan-1-one:
^{1}H NMR (500 MHz, $CDCl_3$) δ 8.79 (dd, *J* = 4.3, 1.7 Hz, 1H), 8.34 – 7.88 (m, 2H), 7.85 – 7.70 (m, 2H), 7.62 (dq, *J* = 8.5, 0.9 Hz, 1H), 7.58 – 7.47 (m, 2H), 7.43 – 7.28 (m, 2H), 7.13 (d, *J* = 2.9 Hz, 1H), 5.38 (s, 2H).

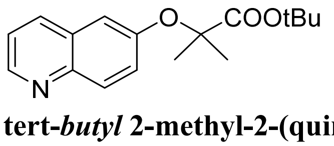

tert-*butyl* 2-methyl-2-(quinolin-6-yloxy)propanoate:
^{1}H NMR (500 MHz, $CDCl_3$) δ 8.77 (dd, *J* = 4.3, 1.7 Hz, 1H), 8.08 – 7.73 (m, 2H), 7.45 – 7.29 (m, 2H), 7.02 (d, *J* = 2.7 Hz, 1H), 1.66 (s, 6H), 1.41 (s, 9H).

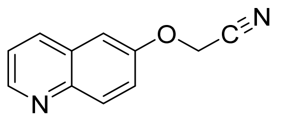

2-(quinolin-6-yloxy)acetonitrile:

^{1}H NMR (500 MHz, CDCl3) δ 9.03 – 8.71 (m, 1H), 8.09 (dd, *J* = 11.3, 8.7 Hz, 2H), 7.50 – 7.32 (m, 2H), 7.20 (d, *J* = 2.8 Hz, 1H), 4.90 (d, *J* = 1.3 Hz, 2H).

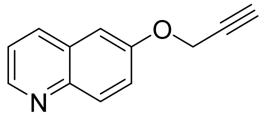

6-(prop-2-yn-1-yloxy)quinoline:

^{1}H NMR (500 MHz, CDCl3) δ 8.80 (dd, *J* = 4.3, 1.7 Hz, 1H), 8.25 – 7.80 (m, 2H), 7.39 (ddd, *J* = 25.5, 8.7, 3.5 Hz, 2H), 7.20 (d, *J* = 2.8 Hz, 1H), 4.83 (d, *J* = 2.4 Hz, 2H), 2.57 (s, 1H).

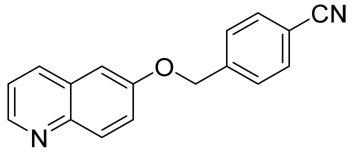

4-((quinolin-6-yloxy)methyl)benzonitrile:

^{1}H NMR (400 MHz, CDCl3) δ 8.79 (dd, *J* = 4.4, 1.6 Hz, 1H), 8.28 – 7.99 (m, 2H), 7.77 – 7.64 (m, 2H), 7.60 (d, *J* = 8.0 Hz, 2H), 7.48 (dd, *J* = 9.2, 2.8 Hz, 1H), 7.40 (dd, *J* = 8.3, 4.3 Hz, 1H), 7.13 (d, *J* = 2.8 Hz, 1H), 5.26 (s, 2H).

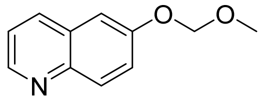

6-(methoxymethoxy)quinoline:

^{1}H NMR (400 MHz, CDCl3) δ 8.78 (dd, *J* = 4.4, 1.8 Hz, 1H), 8.07 (d, *J* = 6.8 Hz, 2H), 7.55 – 7.41 (m, 1H), 7.39 – 7.31 (m, 2H), 5.30 (d, *J* = 1.3 Hz, 2H), 3.53 (d, *J* = 1.7 Hz, 3H).

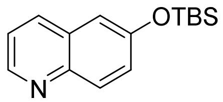

6-((*tert*-butyldimethylsilyl)oxy)quinoline:

^{1}H NMR (400 MHz, CDCl3) δ 8.77 (dd, *J* = 4.3, 1.6 Hz, 1H), 8.20 – 7.91 (m, 2H), 7.34 (ddd, *J* = 16.4, 8.7, 3.4 Hz, 2H), 7.15 (d, *J* = 2.7 Hz, 1H), 1.03 (s, 9H), 0.27 (s, 6H).

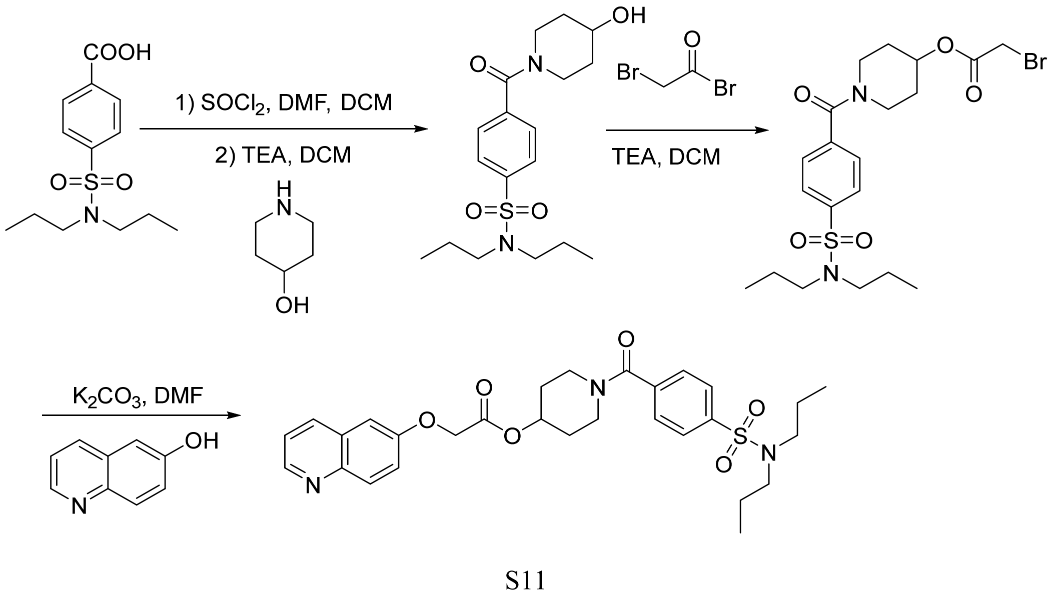

Step 1: The probenecid (0.5 g) was dissolved in dry DCM (10 mL), and cooled to 0 °C, after that, the catalytic DMF, $SOCl_2$ (0.25 mL) were added at 0 °C, respectively. Stirred at 0 °C for 15 min. And then it was stirred at room temperature overnight. After completion, remove the solvent and use directly without purification. The obtained reagent (500 mg), piperidin-4-ol (0.2 g), TEA (0.35 mL), and DMAP (10 mg) were mixed in DCM (10 mL) at room temperature. After completion, water (10 mL) was added and extracted by ethyl acetate. The crude product was purified by dichloromethane/ethyl acetate (5:1) and dichloromethane/methanol (10:1).

Step 2: The probenecid derivative (0.527 g) was dissolved in dry DCM (10 mL), and cooled to 0 °C, after that, the DMAP (18 mg), TEA (300 μL), bromoacetyl bromide (0.15 mL) were added at 0 °C, respectively. Stirred at 0 °C for 15 min. And then it was stirred at room temperature for 20 min. H_2O (10 mL) was added and extracted with DCM (10 mL × 3), combined the organic phase and evaporated in vacuum. The obtained crude will be purified using silica-gel by dichloromethane/methanol (10:1) as eluent.

Step 3: The obtained probenecid derivative (600 mg) from above was dissolved in DMF (10 mL), and then quinolin-6-ol (195 mg) and K_2CO_3 (260 mg) were added and stirred at rt overnight. After completion, the H_2O (10 mL) was added and extracted with Ethyl acetate (10 mL × 3) and combinate the organic phase, dried by 5 g $MgSO_4$ and remove solvent and purify using silica-gel by dichloromethane/ethyl acetate (1:1) to give the desired product.

1-(4-(*N*,*N*-dipropylsulfamoyl)benzoyl)piperidin-4-yl 2-(quinolin-6-yloxy)acetate:

^{1}H NMR (500 MHz, $CDCl_3$) δ 8.82 (dd, J = 4.3, 1.7 Hz, 1H), 8.18 – 7.97 (m, 2H), 7.91 – 7.77 (m, 2H), 7.46 (d, J = 8.6 Hz, 3H), 7.38 (dd, J = 8.3, 4.2 Hz, 1H), 7.03 (d, J = 2.9 Hz, 1H), 5.20 (dt, J = 7.4, 3.7 Hz, 1H), 4.79 (s, 2H), 3.91 (s, 1H), 3.64 (s, 1H), 3.36 (d, J = 17.6 Hz, 1H), 3.22 (s, 1H), 3.13 – 2.99 (m, 4H), 1.92 (d, J = 92.1 Hz, 3H), 1.56 (q, J = 7.5 Hz, 4H), 1.27 (dd, J = 9.5, 5.0 Hz, 1H), 0.87 (t, J = 7.4 Hz, 6H).

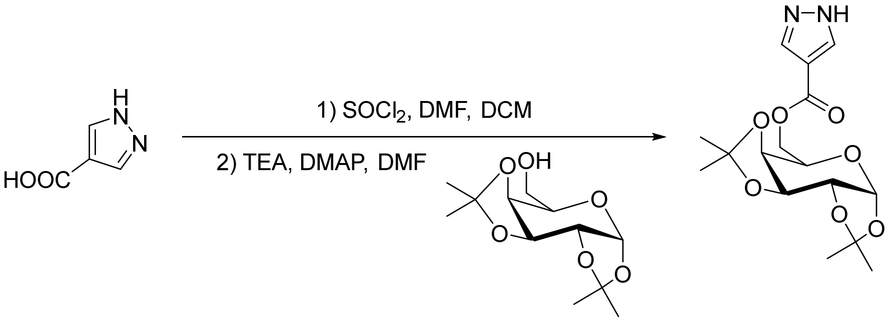

Procedure: The 1*H*-pyrazole-4-carboxylic acid (300mg) was dissolved in catalytic DMF and $SOCl_2$ (8 mL) at 0 ºC, and heated to 75 ºC overnight. After completion, removed the solvent and used directly without purification. The obtained compound was dissolved in DMF (10 mL), and then galactose (600 mg), TEA (0.481 mL), DMAP (28 mg) were added and stirred at room temperature for 4 days. After completion, the DMF was removed directly by vacuum and purify by silica-gel using hexane/ethyl acetate (1:1) to give the desired product (474 mg, 50%).

((3a*R*,5*R*,5a*S*,8a*S*,8b*R*)-2,2,7,7-tetramethyltetrahydro-5*H*-bis([1,3]dioxolo)[4,5-b:4',5'-d]pyran-5-yl)methyl 1*H*-pyrazole-4-carboxylate:

^{1}H NMR (500 MHz, $CDCl_3$) δ 8.06 (s, 2H), 5.56 (d, *J* = 5.0 Hz, 1H), 4.65 (dd, *J* = 7.9, 2.5 Hz, 1H), 4.49 (dd, *J* = 11.6, 4.4 Hz, 1H), 4.42 – 4.27 (m, 3H), 4.22 – 4.13 (m, 1H), 1.49 (d, *J* = 19.9 Hz, 6H), 1.34 (d, *J* = 9.5 Hz, 6H).

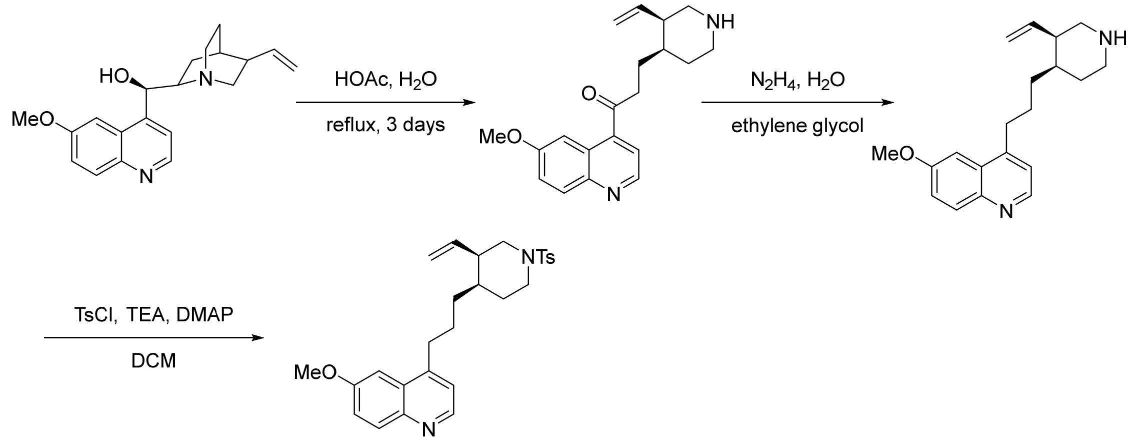

Step 1[1] to **step 2**: According to the reported literature[2], [3*R*,4*R*]-3-Ethenyl-4-[3-(6-methoxyquinolin-4-yl)propyl]piperidine was prepared with the similar yield.

Step 3: [3*R*,4*R*]-3-Ethenyl-4-[3-(6-methoxyquinolin-4-yl)propyl]piperidine (0.5 mg) was dissolved in dry DCM (30 mL), and then TEA (0.675 mL), DMAP (197 mg), 4-toluenesulfonyl chloride (338 mg) were added subsequently and stirred at room temperature overnight. After the completion, H_2O (10 mL) was added and extracted by DCM (10 mL × 3). The organic phase was removed to give the crude product. The crude product was purified by hexane/ethyl acetate (1:4) to deliver the pure white solid (620 mg, 83%).

6-Methoxy-4-(3-((3*R*,4*R*)-1-tosyl-3-vinylpiperidin-4-yl)propyl)quinoline:

^{1}H NMR (500 MHz, $CDCl_3$) δ 8.64 (d, *J* = 4.4 Hz, 1H), 8.00 (d, *J* = 9.2 Hz, 1H), 7.62 (d, *J* = 8.0 Hz, 2H), 7.43 – 7.28 (m, 3H), 7.15 (dd, *J* = 21.9, 3.6 Hz, 2H), 6.00 (dt, *J* = 17.1, 9.7 Hz, 1H), 5.22 – 5.05 (m, 2H), 3.92 (s, 3H), 3.72 (d, *J* = 11.6 Hz, 1H), 3.63 (d, *J* = 11.4 Hz, 1H), 2.95 (t, *J* = 7.6 Hz, 2H), 2.43 (s, 3H), 2.39 – 2.26 (m, 2H), 2.17 (s, 1H), 1.84 – 1.63 (m, 2H), 1.57 – 1.49 (m, 1H), 1.38 (q, *J* = 9.1, 7.0 Hz, 2H), 1.32 – 1.20 (m, 2H).

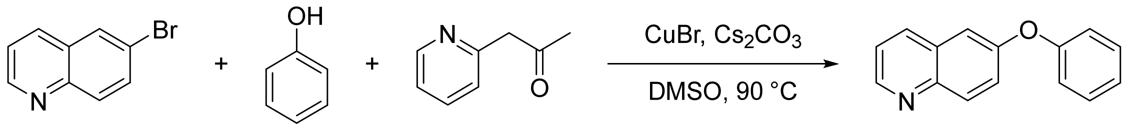

Procedure:[3] A sealed tube equipped with a Teflon valve was charged with a magnetic stir bar, CuBr (14 mg, 0.10mmol, 10 mol %), Cs_2CO_3 (650 mg, 2 mmol) and phenol. The tube was evacuated and backfilled with argon (this procedure was repeated three times). Under a counter flow of argon, 1.2 mmol phenols (if liquid), 1 mmol aryl halide (if liquid), DMSO (1.5 mL) and 1-(pyridin-2-yl)propan-2-one (27 mg, 0.2 mmol, 20 mol %) were added by syringe. The tube was evacuated and backfilled with argon again (this procedure was repeated three times) and sealed. The reaction mixture was heated to the indicated temperature (90 °C) overnight. After cooling to room temperature, the mixture was diluted

with ethyl acetate (10 ml), passed through a fritted glass filter to remove the inorganic salts and the solvent was removed under vacuum. The residue was purified by column chromatography on silica-gel using hexane/ethyl acetate (2:1) and the product was dried under high vacuum for at least 1 h.

6-Phenoxyquinoline:

^{1}H NMR (500 MHz, $CDCl_3$) δ 8.90 – 8.73 (m, 1H), 8.10 (d, *J* = 9.1 Hz, 1H), 8.01 (dd, *J* = 8.4, 1.5 Hz, 1H), 7.50 (dd, *J* = 9.1, 2.7 Hz, 1H), 7.38 (ddd, *J* = 14.6, 8.5, 5.7 Hz, 3H), 7.23 (d, *J* = 2.7 Hz, 1H), 7.22 – 7.16 (m, 1H), 7.14 – 7.06 (m, 2H).

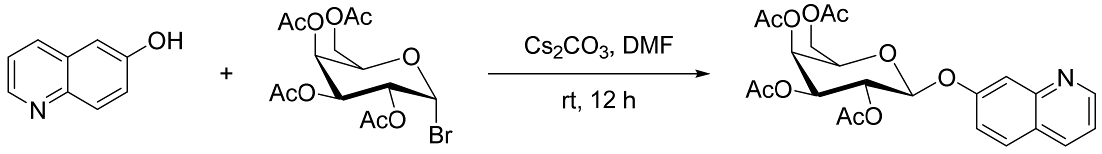

Procedure:[4] The (2*R*,3*S*,4*S*,5*R*,6*R*)-2-(acetoxymethyl)-6-bromotetrahydro-2*H*-pyran-3,4,5-triyl triacetate (1.0 g), 6-hydroxyquinoline (0.35 g) were dissolved in DMF (15 mL). Afterwards, the Cs_2CO_3 (1.0 g) was added and stirred at room temperature overnight. After completion, 0.1 M HCl was added and then extracted by ethyl acetate (10 mL × 3) and washed by 2 M NaOH. After combination of organic phase, removed the solvent to give the crude product, which was purified by DCM : EA (1:1) to deliver the solid (728 mg, 63%).

(2*R*,3*S*,4*S*,5*R*,6*S*)-2-(acetoxymethyl)-6-(quinolin-7-yloxy)tetrahydro-2*H*-pyran-3,4,5-triyl triacetate:

^{1}H NMR (500 MHz, $CDCl_3$) δ 8.84 (dd, *J* = 4.2, 1.8 Hz, 1H), 8.17 – 7.97 (m, 2H), 7.41 (ddd, *J* = 16.7, 8.8, 3.4 Hz, 2H), 7.32 (d, *J* = 2.7 Hz, 1H), 5.57 (dd, *J* = 10.4, 7.9 Hz, 1H), 5.50 (d, *J* = 3.4 Hz, 1H), 5.29 – 5.19 (m, 1H), 5.16 (dd, *J* = 10.4, 3.4 Hz, 1H), 4.28 (dd, *J* = 11.0, 6.8 Hz, 1H), 4.23 – 4.08 (m, 2H), 2.21 (s, 3H), 2.09 (s, 3H), 2.07 (s, 3H), 2.03 (s, 3H).

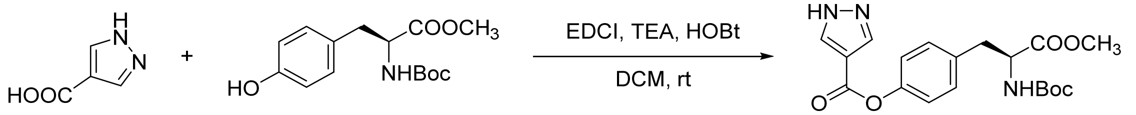

Procedure:[5] The 1*H*-pyrazole-4-carboxylic acid (230 mg, 2.0 mmol) was dissolved in DCM (10 mL), and then tyrosine derivative (600 mg, 2.0 mmol), DCC (503 mg), TAE (425 uL). The mixture was stirred at room temperature overnight. After the completion, the H_2O (10 mL) was added and extracted by ethyl acetate (10 ml × 3), combine the organic phase and evaporate to give the crude product, which was purified by flash chromatography (Hexane/ethyl acetate = 1 : 1) to give white solid (356 mg, 51%).

(*S*)-4-(2-((*tert*-butoxycarbonyl)amino)-3-methoxy-3-oxopropyl)phenyl 1*H*-pyrazole-4-carboxylate:

^{1}H NMR (400 MHz, $CDCl_3$) δ 8.15 (s, 2H), 7.16 (d, *J* = 8.5 Hz, 2H), 7.09 (d, *J* = 8.6 Hz, 2H), 5.20 (d, *J* = 8.5 Hz, 1H), 4.59 (d, *J* = 7.4 Hz, 1H), 3.73 (d, *J* = 3.6 Hz, 3H), 3.27 – 2.92 (m, 2H), 1.42 (s, 9H).

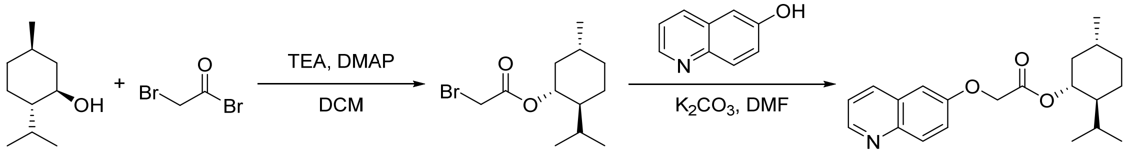

Step 1:[6] The L-menthol (0.5 g) was dissolved in dry DCM (15 mL), and cooled to 0 °C, after that, the DMAP (39 mg), TEA (670 µL), bromoacetyl bromide (0.42 mL) were added at 0 °C, respectively. Stirred at 0 °C for 15 min. And then it was stirred at room temperature for 20 min. H_2O (10 mL) was added and extracted with DCM (10 mL × 3), combined the organic phase and evaporated in vacuum. The obtained crude will be purified using silica-gel by hexane/dichloromethane (2:1) as eluent.

Step 2:[6] The L-menthol derivative (500 mg) from above was dissolved in DMF (10 mL), and then quinolin-6-ol (261 mg) and K_2CO_3 (500 mg) were added and stirred at rt overnight. After completion, the H_2O (10 mL) was added and extracted with Ethyl acetate (10 mL × 3) and combinate the organic phase, dried by 5 g $MgSO_4$ and remove solvent and purify using silica-gel by hexane/ethyl acetate (2:1) to give the desired product.

(1*R*,2*S*,5*R*)-2-isopropyl-5-methylcyclohexyl 2-(quinolin-6-yloxy)acetate:

^{1}H NMR (500 MHz, $CDCl_3$) δ 8.79 (dd, *J* = 4.2, 1.7 Hz, 1H), 8.23 – 7.83 (m, 2H), 7.46 (dd, *J* = 9.2, 2.8 Hz, 1H), 7.36 (dd, *J* = 8.3, 4.2 Hz, 1H), 7.01 (d, *J* = 2.8 Hz, 1H), 4.82 (td, *J* = 10.9, 4.4 Hz, 1H), 4.74 (d, *J* = 1.0 Hz, 2H), 1.66 (dddd, *J* = 18.7, 15.2, 9.4, 5.6 Hz, 4H), 1.49 (tdd, *J* = 12.0, 6.5, 3.4 Hz, 1H), 1.38 (ddt, *J* = 14.4, 11.0, 3.2 Hz, 1H), 1.11 – 0.96 (m, 3H), 0.90 (d, *J* = 6.6 Hz, 3H), 0.88 – 0.83 (m, 1H), 0.78 (d, *J* = 7.0 Hz, 3H), 0.67 (d, *J* = 7.0 Hz, 3H).

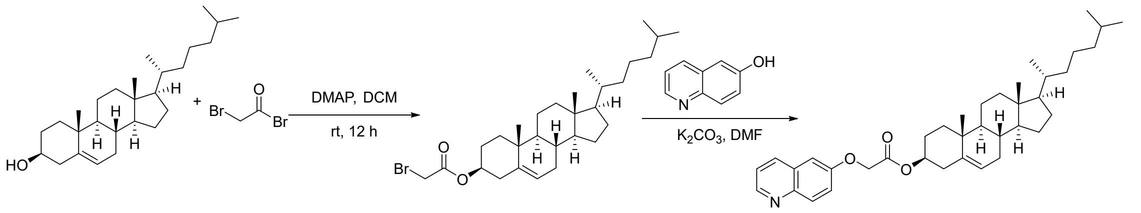

Step 1:[6] The cholesterol (1.0 g) was dissolved in dry DCM, and cooled to 0 °C, after that, the DMAP (33 mg), bromoacetyl bromide (0.28 mL) were added at 0 °C, respectively. Stirred at 0 °C for 15 min. And then it was stirred at room temperature for 20 min. H_2O (10 mL) was added and extracted with DCM (10 mL × 3), combined the organic phase and evaporated in vacuum. The obtained crude will be purified using silica-gel by hexane/ethyl acetate (50:1) as eluent.

Step 2:[6] The cholesterol derivative (500 mg) from above was dissolved in DMF (10 mL), and then quinolin-6-ol (162 mg) and K_2CO_3 (280 mg) were added and stirred at rt overnight. After completion, the H_2O (10 mL) was added and extracted with Ethyl acetate (10 mL × 3) and combinate the organic phase, dried by 5 g $MgSO_4$ and remove solvent and purify using silica-gel by hexane/DCM/ethyl acetate (5:4:1) to give the desired product.

(3*S*,8*S*,9*S*,10*R*,13*R*,14*S*,17*R*)-10,13-dimethyl-17-((*R*)-6-methylheptan-2-yl)-2,3,4,7,8,9,10,11,12,13,14,15,16,17-tetradecahydro-1*H*-cyclopenta[a]phenanthren-3-yl 2-(quinolin-6-yloxy)acetate:

^{1}H NMR (400 MHz, $CDCl_3$) δ 8.80 (dd, *J* = 4.3, 1.6 Hz, 1H), 8.04 (d, *J* = 9.1 Hz, 2H), 7.47 (dd, *J* = 9.2, 2.8 Hz, 1H), 7.37 (dd, *J* = 8.3, 4.2 Hz, 1H), 7.03 (d, *J* = 2.8 Hz, 1H), 5.38 (d, *J* = 5.0 Hz, 1H), 4.89 – 4.75 (m, 1H), 4.73 (s, 2H), 2.37 (d, *J* = 8.1 Hz, 2H), 2.06 – 1.93 (m, 2H), 1.93 – 1.78 (m, 3H), 1.70 – 1.42 (m, 7H), 1.41 – 1.30 (m, 3H), 1.29 – 1.24 (m, 1H), 1.14 (ddd, *J* = 19.2, 14.1, 8.3 Hz, 7H), 1.07 – 0.95 (m, 6H), 0.92 (d, *J* = 6.5 Hz, 3H), 0.87 (dd, *J* = 6.6, 1.8 Hz, 6H), 0.68 (s, 3H).

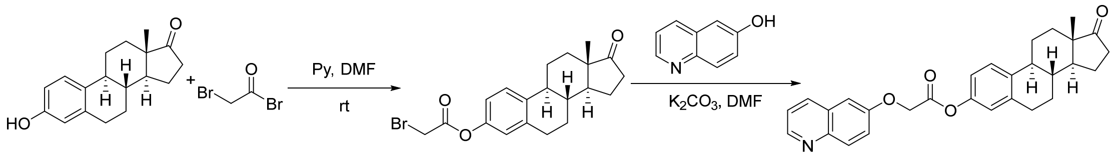

Step 1:[6] The 1,3,5(10)-estratrien-3-ol-17-one (500 mg) was dissolved in dry DMF, and cooled to 0 °C, after that, the pyridine (0.149 mL), bromoacetyl bromide (0.161 mL) were added at 0 °C, respectively. Stirred at 0 °C for 15 min. And then it was stirred at room temperature for 20 min. H_2O (10 mL) was added and extracted with DCM (3 × 10 mL), combined the organic phase and evaporated in vacuum. The obtained crude will be purified using silica-gel by hexane/dichloromethane/ethyl acetate (6:3:1) as eluent.

Step 2:[6] The estrone derivative (0.175 mg, 0.45 mmol) from above was dissolved in DMF (5 mL), and then quinolin-6-ol (68 mg, 0.45 mmol) and K_2CO_3 (123 mg, 0.9 mmol) were added and stirred at rt overnight. The H_2O (5 mL) was added and extracted with ethyl acetate (5 mL × 3) and combinate the organic phase, dried by 5 g $MgSO_4$ and remove solvent and purify using silica-gel by hexane/ethyl acetate (1:2) to give the desired product.

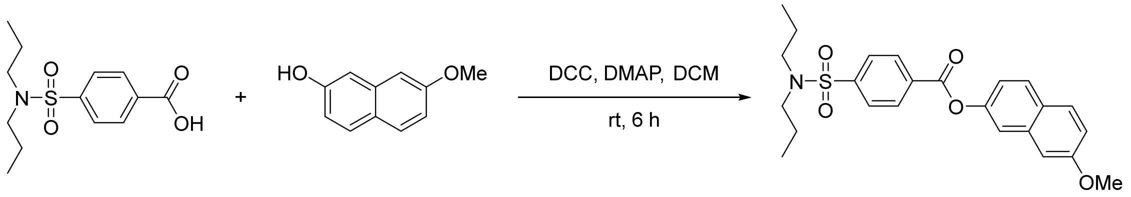

Procedure:

To the 100 mL round bottom flask, the probenecid (500 mg), 7-methoxynaphthalen-2-ol (305 mg), DCC (550 mg), and DMAP (20 mg) were added in 20 mL DCM at room temperature and stirred about 6 hours. After the completion of the reaction, the solution was filtrated by the celite and remove the solvent. The crude product was purified directly by flash chromatograph using the mixture of hexane/ethyl acetate (5:1) to provide the white solid (330 mg, 42%).

^{1}H NMR (500 MHz, $CDCl_3$) δ 8.48 (d, *J* = 8.2 Hz, 2H), 8.08 (d, *J* = 8.2 Hz, 2H), 7.95 (d, *J* = 8.8 Hz, 1H), 7.88 (d, *J* = 8.9 Hz, 1H), 7.71 (d, *J* = 2.3 Hz, 1H), 7.30 (ddd, *J* = 20.6, 8.9, 2.4 Hz, 2H), 7.24 (d, *J* = 2.5 Hz, 1H), 4.04 (s, 3H), 3.39 – 3.09 (m, 4H), 1.83 – 1.54 (m, 4H), 1.01 (t, *J* = 7.4 Hz, 6H).

^{13}C NMR (126 MHz, $CDCl_3$) δ 164.16, 158.48, 149.06, 145.06, 135.29, 133.06, 130.95, 129.49, 129.46, 127.32, 127.24, 118.97, 118.44, 117.72, 105.84, 55.47, 50.07, 22.07, 11.30.

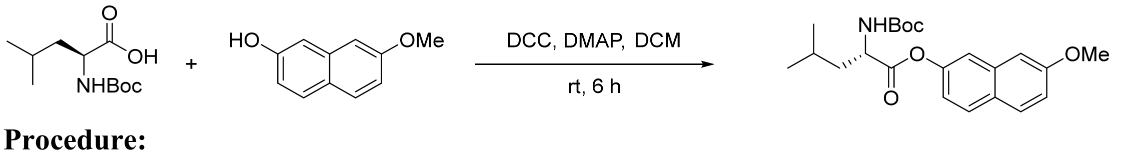

Procedure:

To the 100 mL round bottom flask, the leucine (560 mg), 7-methoxynaphthalen-2-ol (350 mg), DCC (600 mg), and DMAP (20 mg) were added in 20 mL DCM at room temperature and stirred about 6 hours. After the completion of the reaction, the solution was filtrated by the celite and remove the solvent. The crude product was purified directly by flash

chromatograph using the mixture of hexane/ethyl acetate (3:1) to provide the white solid (521 mg, 67%).

^{1}H NMR (500 MHz, $CDCl_3$) δ 7.92 (d, *J* = 8.8 Hz, 1H), 7.88 (d, *J* = 8.9 Hz, 1H), 7.61 (d, *J* = 2.3 Hz, 1H), 7.28 (dd, *J* = 8.9, 2.5 Hz, 1H), 7.26 – 7.17 (m, 2H), 5.15 (d, *J* = 8.7 Hz, 1H), 4.74 (q, *J* = 5.3, 4.8 Hz, 1H), 4.06 (s, 3H), 2.12 – 1.95 (m, 2H), 1.91 – 1.81 (m, 1H), 1.74 (s, 1H), 1.63 (s, 9H), 1.20 (d, *J* = 6.2 Hz, 6H).

^{13}C NMR (126 MHz, $CDCl_3$) δ 172.76, 158.64, 155.90, 149.24, 135.50, 129.67, 129.60, 127.42, 119.09, 118.71, 117.82, 106.05, 80.48, 55.72, 52.80, 42.14, 28.74, 25.37, 23.34, 22.36.

1.5. Large scale synthesis:

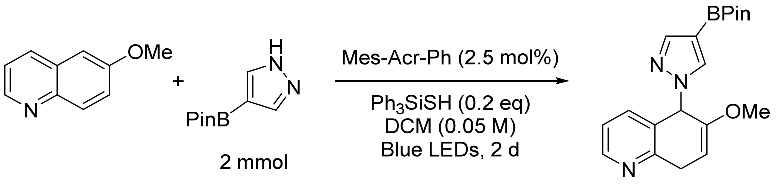

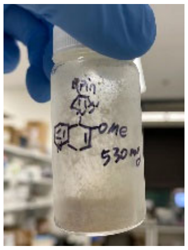

To an oven-dried 20 mL-Schlenk tube equipped with a stir bar, was added 4-(4,4,5,5-tetramethyl-1,3,2-dioxaborolan-2-yl)-1*H*-pyrazole (420 mg, 2.0 mmol), 6-methoxyquinoline (480 mg, 3 mmol), Ph_3SiSH (240 mg, 0.4 mmol), and **Mes-Acr2** (20 mg, 0.05 mmol). The tube was evacuated and back-filled with N_2 for three times, then sealed with rubber stopper and parafilm. Subsequently, the degassed dichloromethane (40 mL) was added. The reaction was irradiated by the two 40 W Kessil Blue LEDs cooling by the electronic fan for 48 hours. After the completion of reactions, the resulted solution was purified by flash column chromatography on silica gel eluting with hexane/ethyl acetate (5:1) in proper ratio. The desired product will be obtained as the grey solid (530 mg, 73%).

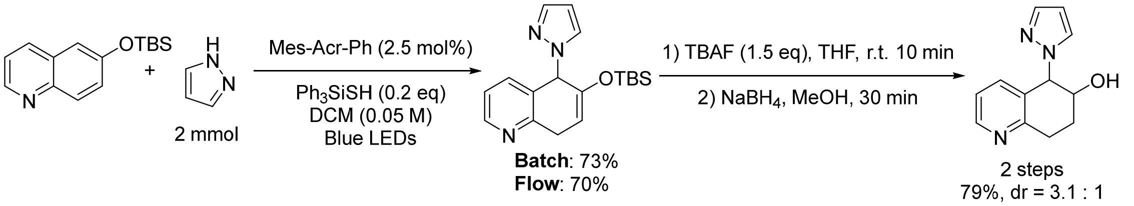

Step 1:

Batch method: To an oven-dried 100 mL-Schlenk tube equipped with a stir bar, was added pyrazole (2 mmol), quinoline derivatives (3 mmol), Ph_3SiSH (0.4 mmol), and **Mes-Acr2** (0.05 mmol). The tube was evacuated and back-filled with N_2 for three times, then sealed with rubber stopper and parafilm. Subsequently, the degassed dichloromethane (40 mL) was added. The reaction was irradiated by the two 40 W Kessil Blue LEDs. After the completion of reactions, the resulted solution was purified by flash column chromatography on silica gel eluting with hexane/ethyl acetate (5:1) to deliver the brown solid (490 mg, 73%).

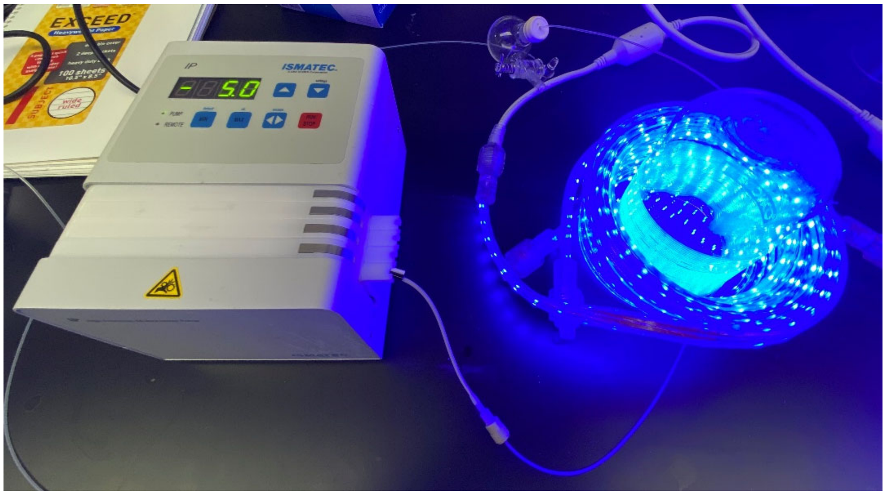

Supplementary Figure 5. Reaction setup using flow system under the irradiation of Blue LEDs

Flow method: To an oven-dried 100 mL-Schlenk tube equipped with a stir bar, was added pyrazole (2 mmol), quinoline derivatives (3 mmol), Ph_3SiSH (0.4 mmol), and **Mes-Acr2** (0.05 mmol). The tube was evacuated and back-filled with N_2 for three times, then sealed with rubber stopper and parafilm. Subsequently, the degassed dichloromethane (40 mL) was added. The solution was then pumped through the photoreactor at a flow rate to achieve a setting residence time of 3 h and collected in the flask under the N_2 atmosphere. The reaction rate is 2.2 mL/h, the tube length 13 m, tube volume is 6.5 mL, internal diameter is 0.6 mm. The flow setup is single-pass condition. After the completion of reactions, the resulted solution was purified by flash column chromatography on silica gel eluting with hexane/ethyl acetate (5:1) to deliver the brown solid (470 mg, 70%)

Step 2: The 6-((*tert*-butyldimethylsilyl)oxy)-5-(1*H*-pyrazol-1-yl)-5,8-dihydroquinoline (1.0 mmol) was dissolved in 5 mL THF, and then the 2.0 M TBAF was added subsequently and stirred at room temperature for 10 min. After completion of the reaction, remove the solvent and purify directly using short pad of silica-gel to get the crude product. The obtained product was dissolved in the methanol (5 mL) and $NaBH_4$ (2.0 mmol) was added slowly. 30 mins later, directly remove the solvent and purify by the flash chromatography using DCM/MeOH to get the colorless oil **3cc** (170 mg, 79%) with the dr value 3.1 : 1.

5-(1*H*-pyrazol-1-yl)-5,6,7,8-tetrahydroquinolin-6-ol (3cg-1) (with lower polarity):

^{1}H NMR (500 MHz, $CDCl_3$) δ 8.42 (dd, *J* = 4.7, 1.7 Hz, 1H), 7.57 (d, *J* = 2.0 Hz, 1H), 7.40 (d, *J* = 2.4 Hz, 1H), 7.03 (dd, *J* = 7.8, 4.7 Hz, 1H), 6.94 (d, *J* = 8.0 Hz, 1H), 6.31 (t, *J* = 2.2 Hz, 1H), 5.47 (d, *J* = 3.2 Hz, 1H), 4.90 (s, 1H), 4.41 (dd, *J* = 6.5, 3.2 Hz, 1H), 3.31 (ddd, *J* = 18.2, 9.1, 6.6 Hz, 1H), 2.96 (ddd, *J* = 18.1, 6.7, 4.8 Hz, 1H), 2.40 – 1.70 (m, 2H).

^{13}C NMR (126 MHz, $CDCl_3$) δ 157.11, 149.07, 140.38, 136.05, 131.12, 128.81, 121.59, 105.64, 68.04, 63.32, 28.31, 27.21.

5-(1*H*-pyrazol-1-yl)-5,6,7,8-tetrahydroquinolin-6-ol (**3cg-2**) (with higher polarity):

^{1}H NMR (500 MHz, $CDCl_3$) δ 8.45 (d, *J* = 4.7 Hz, 1H), 7.61 (d, *J* = 1.8 Hz, 1H), 7.48 (d, *J* = 2.3 Hz, 1H), 7.06 (dd, *J* = 8.0, 4.6 Hz, 1H), 7.02 – 6.94 (m, 1H), 6.35 (d, *J* = 2.5 Hz, 1H),

5.33 (d, J = 8.5 Hz, 1H), 4.40 (ddd, J = 11.7, 8.4, 3.6 Hz, 1H), 3.18 (dd, J = 8.7, 4.6 Hz, 2H), 2.78 (s, 1H), 2.33 (dq, J = 13.1, 4.3 Hz, 1H), 2.14 – 1.96 (m, 1H).
^{13}C NMR (126 MHz, $CDCl_3$) δ 156.27, 149.19, 140.77, 135.93, 130.40, 129.42, 121.82, 106.19, 70.94, 68.04, 30.80, 29.19.
HRMS (ESI+): calcd for $C_{12}H_{13}N_3O$ $[M+K]^+$ 254.0690, found 254.0703.

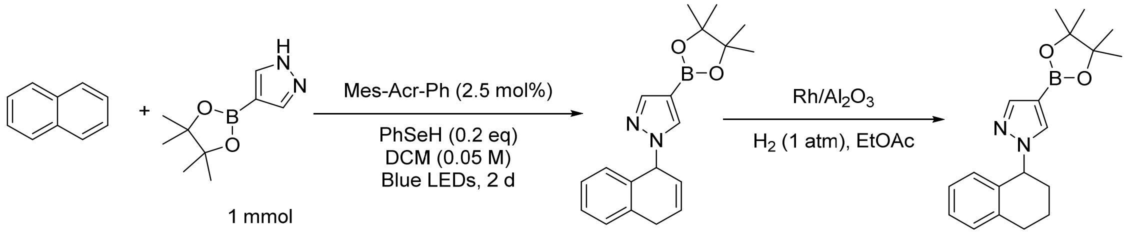

Procedure:
Step 1: To an oven-dried 20 mL-Schlenk tube equipped with a stir bar, was added 4-(4,4,5,5-tetramethyl-1,3,2-dioxaborolan-2-yl)-1*H*-pyrazole (200 mg, 1.0 mmol), naphthalene (270 mg, 2.0 mmol), and **Mes-Acr2** (10 mg, 0.025 mmol). The tube was evacuated and back-filled with N_2 for three times, then sealed with rubber stopper and parafilm. Subsequently, the degassed dichloromethane (20 mL) was added. Afterwards, the PhSeH (25 uL, 0.2 mmol), 2,6-lutidine (25 uL, 0.2 mmol), was added using the syringe. The reaction was irradiated by the two 40 W Kessil Blue LEDs cooling by the electronic fan. After the completion of reactions, the resulted solution was purified by flash column chromatography on silica gel eluting with hexane/ethyl acetate (5:1) in proper ratio. The desired product will be obtained as the colorless oil (125 mg, 39%).
Step 2: Once the dearomatized product was obtained, to the 10 mL round bottom flask, the 5 mL ethyl acetate was added, followed by the addition of Rd/Al_2O_3 (5%, 40 mg). The flask was evacuated and back-filled with H_2 for 2 times. The reaction was stirred at room temperature for 6 hours. After the completion of reaction, the solution was filtered and then purified directly using silica gel by the flash chromatograph (hexane : dichloromethane: ethyl acetate = 8 : 1 : 1) to give the desired product **3ce** as the white solid (103 mg, 81%).
1-(1,2,3,4-tetrahydronaphthalen-1-yl)-4-(4,4,5,5-tetramethyl-1,3,2-dioxaborolan-2-yl)-1*H*-pyrazole (3ci):
^{1}H NMR (500 MHz, $CDCl_3$) δ 7.84 (s, 1H), 7.41 (s, 1H), 7.22 (td, J = 7.4, 1.4 Hz, 1H), 7.18 – 7.05 (m, 2H), 6.96 (d, J = 7.6 Hz, 1H), 5.59 (t, J = 5.5 Hz, 1H), 3.01 – 2.71 (m, 2H), 2.33 (dtd, J = 13.2, 6.9, 3.5 Hz, 1H), 2.24 – 2.10 (m, 1H), 1.94 – 1.66 (m, 2H), 1.29 (d, J = 2.1 Hz, 12H).
^{13}C NMR (126 MHz, $CDCl_3$) δ 145.55, 138.04, 135.88, 133.85, 129.64, 129.49, 128.20, 126.51, 83.34, 60.25, 30.82, 29.13, 24.91, 24.89, 19.34.
HRMS (ESI+): calcd for $C_{19}H_{25}BN_2O_2$ $[M+H]^+$ 325.2085, found 325.2081.

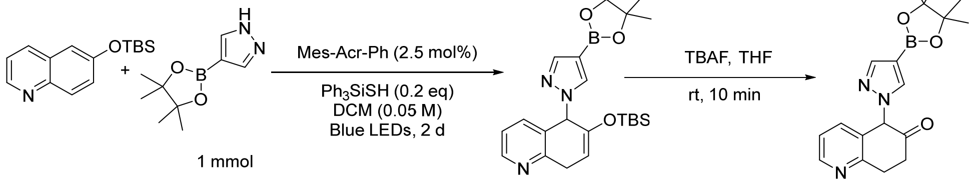

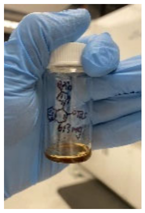

Procedure:

Step 1: To an oven-dried 20 mL-Schlenk tube equipped with a stir bar, was added 4-(4,4,5,5-tetramethyl-1,3,2-dioxaborolan-2-yl)-1*H*-pyrazole (420 mg, 2.0 mmol), 6-((*tert*-butyldimethylsilyl)oxy)quinoline (800 mg, 3.0 mmol), Ph_3SiSH (240 mg, 0.4 mmol), and **Mes-Acr2** (20 mg, 0.05 mmol). The tube was evacuated and back-filled with N_2 for three times, then sealed with rubber stopper and parafilm. Subsequently, the degassed dichloromethane (40 mL) was added. The reaction was irradiated by the two 40 W Kessil Blue LEDs cooling by the electronic fan for 48 hours. After the completion of reactions, the resulted solution was purified by flash column chromatography on silica gel eluting with hexane/ethyl acetate (5:1) in proper ratio. The desired product will be obtained as the grey solid (613 mg, 63%).

Step 2: The obtained dearomatized product (100 mg, 0.22 mmol) was dissolved in the 5 mL THF, followed by the addition of 1.0 M TBAF (0.35 mL, 0.35 mmol), The reaction was stirred at rt for 10 min until all the starting materials was consumed. The resulting crude was purified by the silical gel eluting with hexane/ethyl acetate (2:1). The desired product is the mixture of ketone and enolate with ratio 4:1 based on ^{1}H NMR.

6-((tert-butyldimethylsilyl)oxy)-5-(4-(4,4,5,5-tetramethyl-1,3,2-dioxaborolan-2-yl)-1H-pyrazol-1-yl)-5,8-dihydroquinoline (3cj):

^{1}H NMR (500 MHz, $CDCl_3$) δ 8.27 (d, J = 4.8 Hz, 1H), 7.55 (s, 1H), 7.49 (s, 1H), 7.27 (d, J = 7.9 Hz, 1H), 6.88 (dd, J = 8.3, 4.5 Hz, 1H), 5.72 (s, 1H), 5.16 (s, 1H), 3.93 – 3.54 (m, 1H), 3.54 – 3.38 (m, 1H), 1.07 (s, 12H), 0.57 (s, 9H), -0.05 (s, 3H), -0.22 (s, 3H).

^{13}C NMR (126 MHz, $CDCl_3$) δ 153.49, 149.01, 145.27, 144.79, 136.55, 135.56, 128.71, 121.88, 104.30, 83.25, 62.00, 36.74, 32.14, 25.40, 24.73, 24.67, 17.78.

HRMS (ESI+): calcd for $C_{24}H_{36}BN_3O_3Si$ $[M+H]^+$ 454.2492, found 454.2489.

5-(4-(4,4,5,5-tetramethyl-1,3,2-dioxaborolan-2-yl)-1*H*-pyrazol-1-yl)-7,8-dihydroquinolin-6(5*H*)-one (3ck):

^{1}H NMR (500 MHz, $CDCl_3$) δ 8.93 – 8.34 (m, 1H), 8.30 – 7.92 (m, 2H), 7.68 – 7.08 (m, 2.2H), 6.24 (s, 0.8H), 4.28 – 3.26 (m, 2H), 3.24 – 2.73 (m, 2H), 1.80 – 1.08 (m, 12H).

^{13}C NMR (126 MHz, $CDCl_3$) δ 202.07, 154.89 (d, J = 242.2 Hz), 150.46 (d, J = 231.3 Hz), 147.43 – 143.22 (m), 137.70, 135.60 (d, J = 92.8 Hz), 129.17, 127.49, 122.22 (d, J = 126.5 Hz), 83.73 (d, J = 24.3 Hz), 68.08, 36.20, 31.07, 24.97 (d, J = 5.2 Hz).

HRMS (ESI+): calcd for $C_{18}H_{22}BN_3O_3$ $[M+H]^+$ 340.1827, found 340.1826.

1.6. DFT calculations

1.6.1. Computational Methods

All calculations were performed with Gaussian 16.[7] Molecular geometries were optimized using the ωB97X-D[8] functional using the def2-SVP basis set. Frequency calculations were performed at the same level of theory as that used for geometry optimization to characterize the stationary points as either minima (no imaginary frequencies) or saddle points (one imaginary frequency) on the potential energy surface. Thermal contributions to free energies were calculated from the vibrational frequencies using the quasi-rigid rotor-harmonic oscillator (RRHO) approach proposed by Grimme.[9] Intrinsic Reaction Coordinate (IRC) calculations were performed to confirm the saddle points as real transition states connecting the expected reactants and products. Single point energies were calculated with the ωB97X-D

functional using the def2-TZVPP basis set for all atoms. Solvation effects were incorporated using the SMD[10] model in single-point energy calculations with dichloromethane as the solvent. Molecular visualizations were created using CYLview.[11] Conformational searches were performed with the MMFF force field as implemented in Spartan '20[12] to ensure that the lowest-energy conformers are presented in the manuscript. Spin density plots were created using Multiwfn[13] and VMD.[14]

1.6.2. Calculated Charges and Spin Densities

Supplementary Figure 6 shows the calculated Hirshfeld charges and spin densities for radical cation intermediates. Bolded numbers indicate the carbons with the highest spin densities within the molecule.

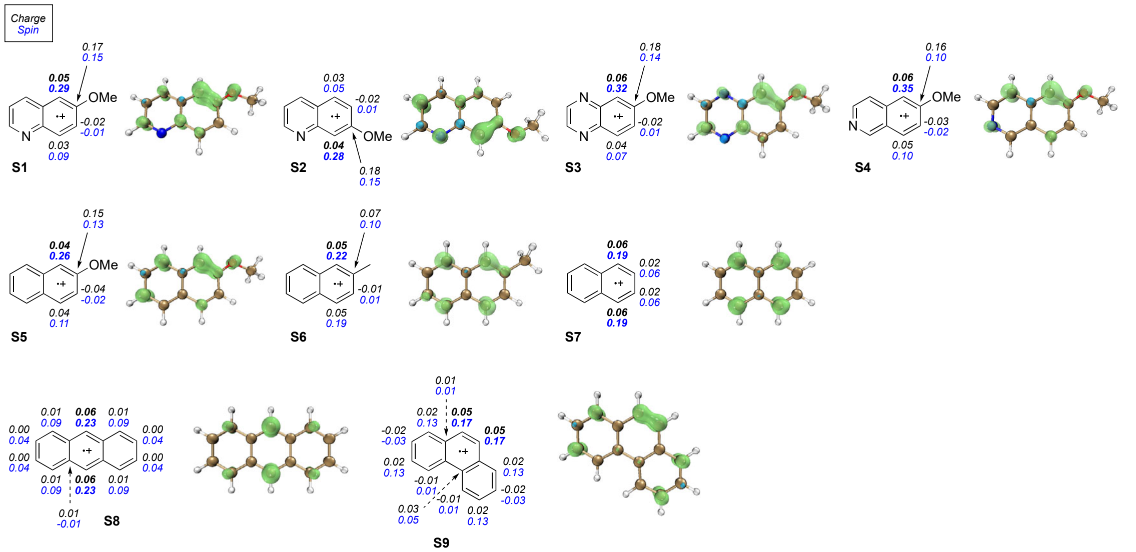

Supplementary Figure 6. Calculated Hirshfeld charges and spin densities at the ωB97X-D/def2-TZVPP, SMD (CH_2Cl_2)//ωB97X-D/def2-SVP level of theory. Spin density plots are shown next to the structures.

1.6.3. Hydrogen Atom Transfer Transition States

Supplementary Figure 7 shows the calculated hydrogen atom transfer (HAT) transition states *en route* to dearomatized products. Our results show that HAT from PhSeH proceeds with kinetic barriers about 9 kcal/mol lower than HAT from Ph_3SiSH, consistent with the higher H-atom transfer rates of PhSeH.

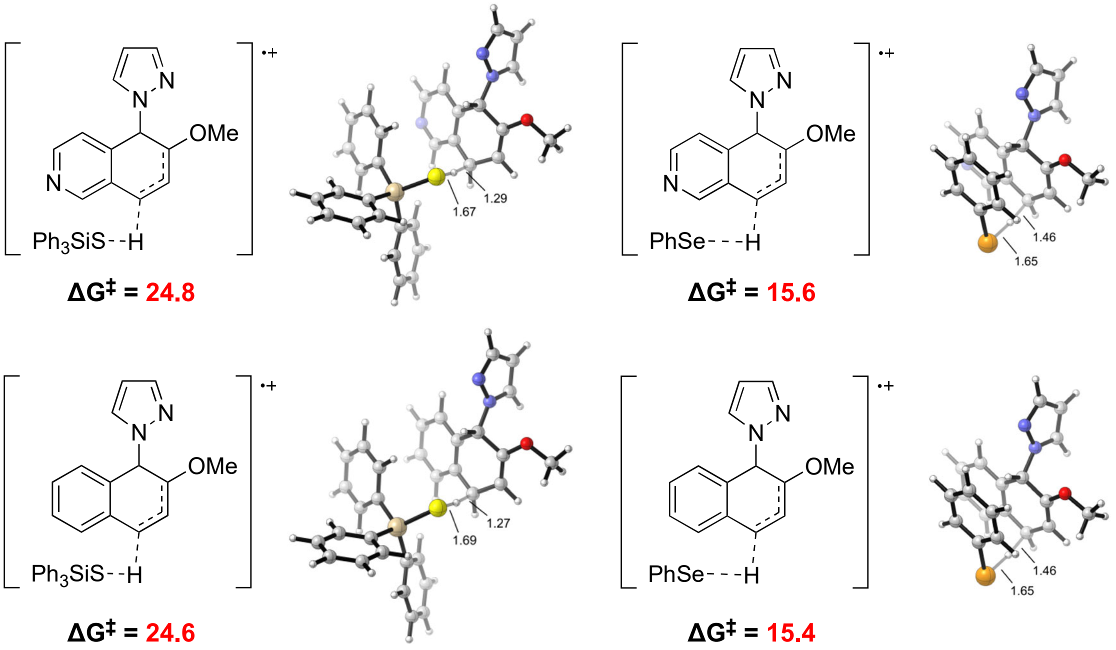

Supplementary Figure 7. Calculated hydrogen atom transfer (HAT) transition states involving Ph_3SiSH or PhSeH and corresponding activation free energies (kcal/mol). Activation free energies were calculated with respect to isolated reactants. Interatomic distances are in ångströms.

1.6.4. Reaction Free Energy Diagram

The calculated free energy diagram of the reaction between **1a** and pyrazole is shown in Supplementary Figure 8. As discussed in the manuscript, radical **7a** can either undergo highly exergonic aromatization to **4a** in the presence of an oxidant and a base, or it can undergo dearomatization to yield **3a**. While the kinetic barriers of the aromatization pathway are difficult to determine due to concentration effects (among other complicating factors), we calculated the free energy barrier of the HAT step *en route* to the dearomatized product **3a**. With Ph_3SiSH as the HAT agent, the HAT barrier was calculated to be 24.0 kcal/mol. An alternative pathway for the formation of **3a** is through reduction of radical **7a** by SET from $PC^{\bullet}$ to form anion **8a**, which then undergoes protonation to form **3a**. Calculations suggest that the two possible pathways for generating **3a** may be energetically comparable, which is in good agreement with the observation that **3a** is generated also in the absence of the HAT agent (albeit in lower yields). While the HAT step leading to the formation of **3a** is endergonic, the cycle becomes overall exergonic (relative to the excited-state photocatalyst and **1a**) upon regeneration of the HAT agent, the ground-state photocatalyst and the base.

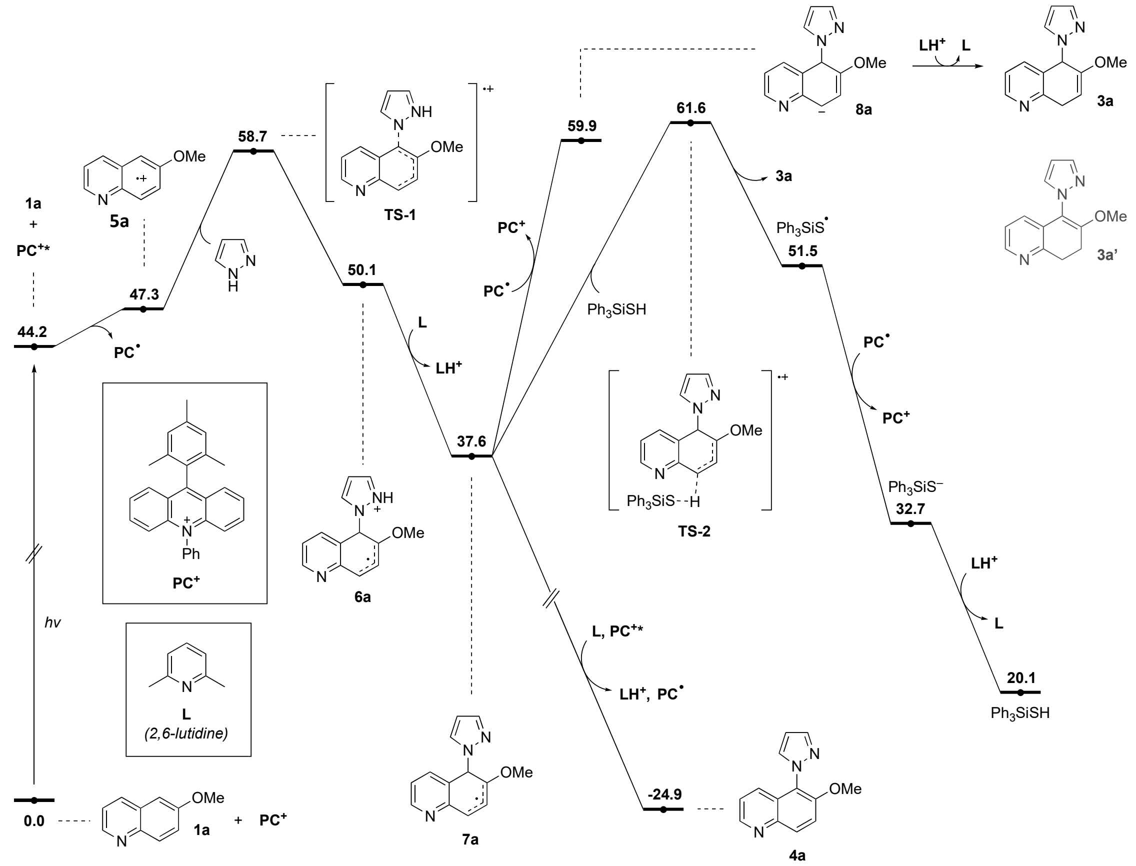

Supplementary Figure 8. Calculated free energy diagram (in kcal/mol) at the ωB97X-D/def2-TZVPP, SMD (CH_2Cl_2)//ωB97X-D/def2-SVP level of theory.

1.6.5. Isomerization of Dearomatized Product from 6-Methoxyisoquinoline

Two possible mechanistic pathways and calculated relative free energies (kcal/mol) for the isomerization of the dearomatized product **I** from 6-methoxyisoquinoline. Our calculations indicate that the deprotonation/reprotonation pathway proceeds through an anionic intermediate **V** that is prohibitively high in energy (35.8 kcal/mol). The SET/deprotonation/HAT is therefore the more likely pathway for the isomerization.

SET/Deprotonation/HAT Pathway

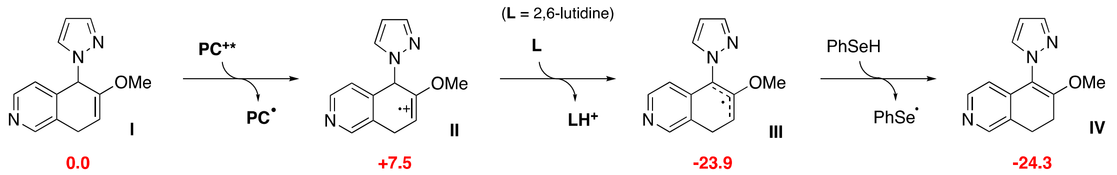

Deprotonation/Reprotonation Pathway

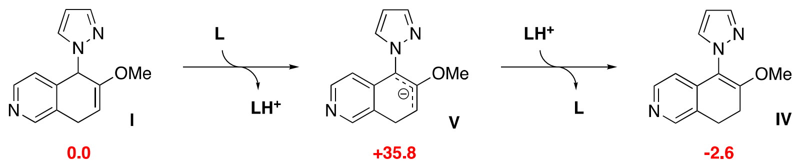

1.6.6. Calculated Energies

Structure	ΔG [ωB97X-D/def2-SVP]	E [ωB97X-D/6-def2-TZVPP, SMD (CH_2Cl_2)]	G (ΔG+E)
**S1**	0.133814	-516.260166	-516.126352
**S2**	0.133735	-516.258280	-516.124545
**S3**	0.121460	-532.285207	-532.163747
**S4**	0.133608	-516.255574	-516.121966
**S5**	0.146141	-500.230177	-500.084036
**S6**	0.140562	-425.006055	-424.865493
**S7**	0.116242	-385.680560	-385.564318
**S8**	0.160625	-539.339615	-539.178990
**S9**	0.067824	-667.347788	-667.279964
**PC^+**	0.389407	-1136.114133	-1135.724726
**PC^{*+}**	0.383100	-1136.037333	-1135.654233
**PC•**	0.385587	-1136.252721	-1135.867134
**L**	0.111573	-326.944211	-326.832638
**LH^+**	0.125191	-327.406124	-327.280933
**Ph_3SiSH**	0.238447	-1383.347166	-1383.108719
**Ph_3SiSH^-**	0.229656	-1382.870050	-1382.640394
**Ph_3SiSH•**	0.229101	-1382.697089	-1382.467988
**pyrazole**	0.046206	-226.223573	-226.177367
**6-methoxyquinoline**	0.135706	-516.479898	-516.344192
**TS-1**	0.200647	-742.486178	-742.285531
**6a**	0.204248	-742.503607	-742.299359
**7a**	0.190515	-742.061416	-741.870901
**8a**	0.188977	-742.166805	-741.977828
**4a**	0.181780	-741.491102	-741.309322
**3a**	0.204296	-742.693753	-742.489457
**3a’**	0.203169	-742.686505	-742.483336
**I**	0.204154	-742.691367	-742.487213
**II**	0.201842	-742.464163	-742.262321
**III**	0.189531	-742.053572	-741.864041
**IV**	0.202960	-742.694276	-742.491316
**V**	0.188786	-742.170691	-741.981905

Supplementary Table 5. Calculated energies in Hartrees.

1.7. Compound Characterization Data

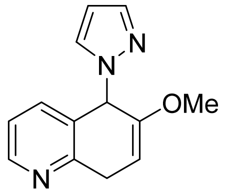

6-methoxy-5-(1*H*-pyrazol-1-yl)-5,8-dihydroquinoline (3a): The title compound was prepared according to the general procedure A. The crude residue was purified by column chromatography on silica gel with an eluent of hexanes/ethyl acetate (2:1) to furnish the pure compound as a brown solid in 76% yield.

^{1}H NMR (500 MHz, $CDCl_3$) δ 8.51 (dd, *J* = 4.7, 1.8 Hz, 1H), 7.55 (dd, *J* = 7.9, 1.7 Hz, 1H), 7.52 (dd, *J* = 1.9, 0.7 Hz, 1H), 7.35 (dd, *J* = 2.3, 0.7 Hz, 1H), 7.20 – 6.98 (m, 1H), 6.25 (t, *J* = 2.1 Hz, 1H), 6.04 (t, *J* = 3.9 Hz, 1H), 5.28 (dd, *J* = 4.6, 3.2 Hz, 1H), 3.89 (dt, *J* = 22.4, 3.7 Hz, 1H), 3.72 (dt, *J* = 22.4, 4.2 Hz, 1H), 3.62 (s, 3H).

^{13}C NMR (126 MHz, $CDCl_3$) δ 154.20, 150.01, 149.30, 139.54, 136.56, 128.58, 127.90, 121.99, 106.27, 95.98, 61.02, 55.04, 31.95.

HRMS (ESI+): calcd for $C_{13}H_{13}N_3O$ $[M+H]^+$ 228.1131, found 228.1129.

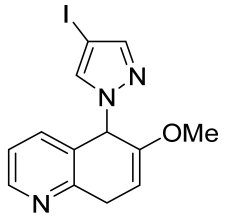

5-(4-iodo-1*H*-pyrazol-1-yl)-6-methoxy-5,8-dihydroquinoline (3b): The title compound was prepared according to the general procedure A. The crude residue was purified by column chromatography on silica gel with an eluent of hexanes/ethyl acetate (2:1) to furnish the pure compound as a brown solid in 69% yield.

^{1}H NMR (500 MHz, $CDCl_3$) δ 8.52 (d, *J* = 4.6 Hz, 1H), 7.56 (dd, *J* = 7.8, 1.7 Hz, 1H), 7.49 (s, 1H), 7.37 (s, 1H), 7.14 (dd, *J* = 7.9, 4.7 Hz, 1H), 6.00 (t, *J* = 3.8 Hz, 1H), 5.28 (dd, *J* = 4.6, 3.1 Hz, 1H), 3.87 (dt, *J* = 22.6, 3.6 Hz, 1H), 3.72 (dt, *J* = 22.6, 4.2 Hz, 1H), 3.61 (s, 3H).

^{13}C NMR (126 MHz, $CDCl_3$) δ 154.17, 149.50, 149.36, 144.60, 136.83, 132.38, 128.12, 122.17, 96.43, 61.66, 57.23, 55.14, 31.81.

HRMS (ESI+): calcd for $C_{13}H_{12}IN_3O$ $[M+H]^+$ 354.0098, found 354.0093.

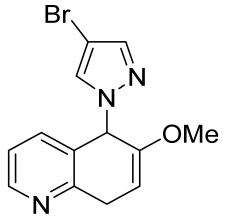

5-(4-bromo-1*H*-pyrazol-1-yl)-6-methoxy-5,8-dihydroquinoline (3c): The title compound was prepared according to the general procedure A. The crude residue was purified by

column chromatography on silica gel with an eluent of hexanes/ethyl acetate (2:1) to furnish the pure compound as a brown solid in 61% yield.
^{1}H NMR (500 MHz, $CDCl_3$) δ 8.53 (d, *J* = 4.6 Hz, 1H), 7.56 (dd, *J* = 8.0, 1.7 Hz, 1H), 7.45 (d, *J* = 0.7 Hz, 1H), 7.34 (d, *J* = 0.7 Hz, 1H), 7.15 (dd, *J* = 7.9, 4.6 Hz, 1H), 5.97 (t, *J* = 3.9 Hz, 1H), 5.29 (dd, *J* = 4.6, 3.1 Hz, 1H), 3.87 (dt, *J* = 22.5, 3.6 Hz, 1H), 3.72 (dt, *J* = 22.6, 4.1 Hz, 1H), 3.62 (s, 3H).
^{13}C NMR (126 MHz, $CDCl_3$) δ 154.23, 149.56, 149.49, 140.11, 136.71, 128.11, 127.99, 122.15, 96.50, 94.05, 61.94, 55.15, 31.89.
HRMS (ESI+): calcd for $C_{13}H_{12}BrN_3O$ $[M+H]^+$ 306.0237, found 306.0232.

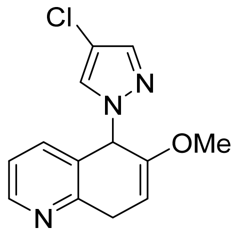

5-(4-chloro-1*H*-pyrazol-1-yl)-6-methoxy-5,8-dihydroquinoline (3d): The title compound was prepared according to the general procedure A. The crude residue was purified by column chromatography on silica gel with an eluent of hexanes/ethyl acetate (2:1) to furnish the pure compound as a brown solid in 53% yield.
^{1}H NMR (500 MHz, $CDCl_3$) δ 8.54 (d, *J* = 3.3 Hz, 1H), 7.61 (dd, *J* = 7.9, 1.8 Hz, 1H), 7.42 (s, 1H), 7.31 (s, 1H), 7.19 (dd, *J* = 7.9, 4.7 Hz, 1H), 5.95 (t, *J* = 3.9 Hz, 1H), 5.29 (dd, *J* = 4.6, 3.1 Hz, 1H), 3.89 (dt, *J* = 22.7, 3.7 Hz, 1H), 3.75 (dt, *J* = 22.6, 4.2 Hz, 1H), 3.63 (s, 3H).
^{13}C NMR (126 MHz, $CDCl_3$) δ 153.99, 149.45, 149.05, 138.08, 137.21, 128.31, 125.99, 122.31, 110.93, 96.30, 61.85, 55.18, 31.62.
HRMS (ESI+): calcd for $C_{13}H_{12}ClN_3O$ $[M+H]^+$ 262.0742, found 262.0739.

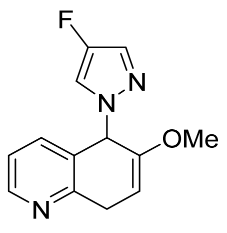

5-(4-fluoro-1*H*-pyrazol-1-yl)-6-methoxy-5,8-dihydroquinoline (3e): The title compound was prepared according to the general procedure A. The crude residue was purified by column chromatography on silica gel with an eluent of hexanes/ethyl acetate (2:1) to furnish the pure compound as a brown solid in 33% yield.
^{1}H NMR (500 MHz, $CDCl_3$) δ 8.77 – 8.35 (m, 1H), 7.56 (dd, *J* = 7.9, 1.7 Hz, 1H), 7.33 (dd, *J* = 4.4, 0.8 Hz, 1H), 7.22 – 7.07 (m, 2H), 5.89 (t, *J* = 4.1 Hz, 1H), 5.42 – 4.99 (m, 1H), 3.86 (dt, *J* = 22.5, 3.7 Hz, 1H), 3.71 (dt, *J* = 22.5, 4.2 Hz, 1H), 3.63 (s, 3H).
^{13}C NMR (126 MHz, $CDCl_3$) δ 154.18, 151.21, 149.62, 149.24, 136.57, 128.06, 126.60, 126.50, 122.13, 113.97, 113.76, 96.36, 62.32, 55.14, 31.93.
HRMS (ESI+): calcd for $C_{13}H_{12}FN_3O$ $[M+H]^+$ 246.1037, found 246.1035.

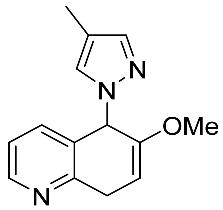

6-methoxy-5-(4-methyl-1*H*-pyrazol-1-yl)-5,8-dihydroquinoline (3f): The title compound was prepared according to the general procedure A. The crude residue was purified by column chromatography on silica gel with an eluent of hexanes/ethyl acetate (2:1) to furnish the pure compound as a brown solid in 53% yield.

^{1}H NMR (500 MHz, $CDCl_3$) δ 8.65 – 8.43 (m, 1H), 7.56 (dd, *J* = 8.0, 1.7 Hz, 1H), 7.30 (s, 1H), 7.12 (dd, *J* = 7.9, 4.7 Hz, 1H), 7.08 (s, 1H), 5.94 (t, *J* = 3.9 Hz, 1H), 5.25 (dd, *J* = 4.6, 3.1 Hz, 1H), 3.87 (dt, *J* = 22.4, 3.7 Hz, 1H), 3.71 (dt, *J* = 22.5, 4.2 Hz, 1H), 3.62 (s, 3H), 2.01 (s, 3H).

^{13}C NMR (126 MHz, $CDCl_3$) δ 154.06, 150.16, 149.09, 140.01, 136.71, 128.91, 126.52, 122.06, 116.96, 95.81, 60.91, 55.09, 31.86, 9.12.

HRMS (ESI+): calcd for $C_{14}H_{15}N_3O$ $[M+H]^+$ 242.1288, found 242.1284.

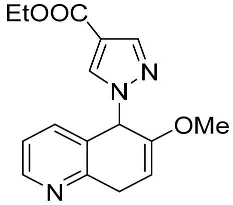

Ethyl 1-(6-methoxy-5,8-dihydroquinolin-5-yl)-1*H*-pyrazole-4-carboxylate (3g): The title compound was prepared according to the general procedure A. The crude residue was purified by column chromatography on silica gel with an eluent of hexanes/ethyl acetate (2:1) to furnish the pure compound as a brown solid in 67% yield.

^{1}H NMR (400 MHz, $CDCl_3$) δ 8.54 (s, 1H), 7.87 (d, *J* = 20.2 Hz, 2H), 7.59 (dd, *J* = 8.0, 1.6 Hz, 1H), 7.16 (dd, *J* = 7.9, 4.7 Hz, 1H), 6.02 (t, *J* = 3.9 Hz, 1H), 5.32 (dd, *J* = 4.6, 3.1 Hz, 1H), 4.26 (q, *J* = 7.1 Hz, 2H), 3.91 (dt, *J* = 22.6, 3.7 Hz, 1H), 3.80 – 3.68 (m, 1H), 3.63 (s, 3H), 1.32 (t, *J* = 7.1 Hz, 3H).

^{13}C NMR (101 MHz, $CDCl_3$) δ 163.09, 154.28, 149.52, 149.25, 141.22, 136.83, 131.53, 127.89, 122.21, 115.94, 96.71, 61.74, 60.37, 55.18, 31.85, 14.52.

HRMS (ESI+): calcd for $C_{16}H_{17}N_3O_3$ $[M+H]^+$ 300.1343, found 300.1339.

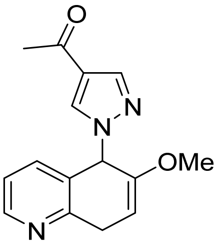

1-(1-(6-methoxy-5,8-dihydroquinolin-5-yl)-1*H*-pyrazol-4-yl)ethan-1-one (3h): The title compound was prepared according to the general procedure C. The crude residue was

purified by column chromatography on silica gel with an eluent of hexanes/ethyl acetate (2:1) to furnish the pure compound as a brown solid in 43% yield.

^{1}H NMR (500 MHz, $CDCl_3$) δ 8.52 (dd, *J* = 4.8, 1.7 Hz, 1H), 7.85 (d, *J* = 14.5 Hz, 2H), 7.56 (dd, *J* = 7.9, 1.7 Hz, 1H), 7.13 (dd, *J* = 8.0, 4.7 Hz, 1H), 6.01 (t, *J* = 3.9 Hz, 1H), 5.56 – 5.15 (m, 1H), 3.87 (dt, *J* = 22.6, 3.7 Hz, 1H), 3.71 (dt, *J* = 22.6, 4.2 Hz, 1H), 3.60 (s, 3H), 2.37 (s, 3H).

^{13}C NMR (126 MHz, $CDCl_3$) δ 192.24, 154.30, 149.66, 149.06, 140.59, 136.61, 130.59, 127.57, 124.77, 122.11, 96.84, 61.79, 55.13, 31.83, 27.96.

HRMS (ESI+): calcd for $C_{15}H_{15}N_3O_2$ $[M+H]^+$ 270.1237, found 270.1234.

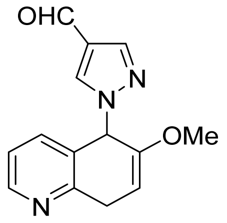

1-(6-methoxy-5,8-dihydroquinolin-5-yl)-1*H*-pyrazole-4-carbaldehyde (3i): The title compound was prepared according to the general procedure C. The crude residue was purified by column chromatography on silica gel with an eluent of hexanes/ethyl acetate (2:1) to furnish the pure compound as a brown solid in 34% yield.

^{1}H NMR (400 MHz, $CDCl_3$) δ 9.80 (s, 1H), 8.54 (dd, *J* = 4.7, 1.6 Hz, 1H), 7.92 (d, *J* = 15.4 Hz, 2H), 7.59 (dd, *J* = 7.9, 1.7 Hz, 1H), 7.16 (dd, *J* = 7.9, 4.7 Hz, 1H), 6.04 (s, 1H), 5.34 (dd, *J* = 4.6, 3.2 Hz, 1H), 3.94 – 3.82 (m, 1H), 3.73 (dt, *J* = 22.7, 4.2 Hz, 1H), 3.63 (s, 3H).

^{13}C NMR (101 MHz, $CDCl_3$) δ 184.20, 154.37, 149.73, 148.97, 140.96, 136.72, 131.56, 127.44, 124.81, 122.18, 97.04, 61.96, 55.18, 31.83.

HRMS (ESI+): calcd for $C_{14}H_{13}N_3O_2$ $[M+H]^+$ 256.1081, found 256.1078.

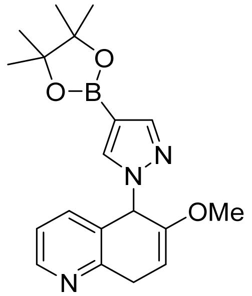

6-methoxy-5-(4-(4,4,5,5-tetramethyl-1,3,2-dioxaborolan-2-yl)-1*H*-pyrazol-1-yl)-5,8-dihydroquinoline (3j): The title compound was prepared according to the general procedure A. The crude residue was purified by column chromatography on silica gel with an eluent of hexanes/ethyl acetate (2:1) to furnish the pure compound as a brown solid in 51% yield.

^{1}H NMR (500 MHz, $CDCl_3$) δ 8.50 (dd, *J* = 4.7, 1.7 Hz, 1H), 7.78 (s, 1H), 7.64 (s, 1H), 7.56 (dd, *J* = 7.9, 1.7 Hz, 1H), 7.11 (dd, *J* = 7.9, 4.7 Hz, 1H), 6.05 (t, *J* = 3.9 Hz, 1H), 5.27 (dd, *J* = 4.6, 3.1 Hz, 1H), 3.87 (dt, *J* = 22.4, 3.7 Hz, 1H), 3.71 (dt, *J* = 22.5, 4.2 Hz, 1H), 3.61 (s, 3H), 1.28 (d, *J* = 2.6 Hz, 12H).

^{13}C NMR (126 MHz, $CDCl_3$) δ 154.23, 149.79, 149.37, 145.62, 136.74, 134.80, 128.46, 122.06, 96.24, 83.46, 61.03, 55.10, 31.96, 24.94, 24.91.
HRMS (ESI+): calcd for $C_{19}H_{24}BN_3O_3$ $[M+H]^+$ 354.1984, found 354.1978.

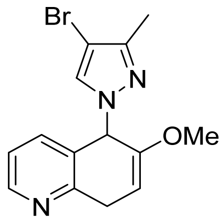

5-(4-bromo-3-methyl-1*H*-pyrazol-1-yl)-6-methoxy-5,8-dihydroquinoline (3k): The title compound was prepared according to the general procedure A. The crude residue was purified by column chromatography on silica gel with an eluent of hexanes/ethyl acetate (2:1) to furnish the pure compound as a brown solid in 51% yield.
^{1}H NMR (500 MHz, $CDCl_3$) δ 8.51 (d, *J* = 4.7 Hz, 1H), 7.58 (d, *J* = 7.9 Hz, 1H), 7.41 (s, 0.18H), 7.21 (s, 0.85H), 7.13 (dd, *J* = 8.1, 4.7 Hz, 1H), 6.10 (s, 0.17H), 5.89 (t, *J* = 4.0 Hz, 0.85H), 5.26 (t, *J* = 3.9 Hz, 0.89H), 5.19 (d, *J* = 3.4 Hz, 0.18H), 3.84 (dt, *J* = 22.5, 3.7 Hz, 1H), 3.70 (dt, *J* = 22.5, 4.3 Hz, 1H), 3.61 (s, 2.55H), 3.59 (s, 0.56H), 2.21 (s, 2.73H), 2.10 (s, 0.53H).
^{13}C NMR (126 MHz, $CDCl_3$) δ 154.12 (d, *J* = 41.4 Hz), 149.37 (d, *J* = 59.8 Hz), 149.24 (d, *J* = 8.8 Hz), 147.38, 138.23 (d, *J* = 234.3 Hz), 136.53 (d, *J* = 65.3 Hz), 128.26 (d, *J* = 28.0 Hz), 122.15 (d, *J* = 5.5 Hz), 95.83 (d, *J* = 108.9 Hz), 94.65 (d, *J* = 62.0 Hz), 60.86 (d, *J* = 173.5 Hz), 55.06 (d, *J* = 11.4 Hz), 31.79, 10.97 (d, *J* = 295.0 Hz).
HRMS (ESI+): calcd for $C_{14}H_{14}BrN_3O$ $[M+H]^+$ 320.0393, found 320.0389.

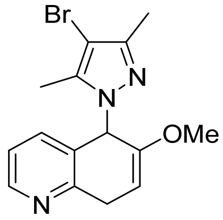

5-(4-bromo-3,5-dimethyl-1H-pyrazol-1-yl)-6-methoxy-5,8-dihydroquinoline (3l): The title compound was prepared according to the general procedure A. The crude residue was purified by column chromatography on silica gel with an eluent of hexanes/ethyl acetate (2:1) to furnish the pure compound as a brown solid in 69% yield.
^{1}H NMR (500 MHz, $CDCl_3$) δ 8.60 – 8.37 (m, 1H), 7.27 (d, *J* = 7.9 Hz, 1H), 7.21 – 7.03 (m, 1H), 6.06 (s, 1H), 5.15 (s, 1H), 3.88 – 3.65 (m, 2H), 3.58 (s, 3H), 2.18 (s, 3H), 1.95 (s, 3H).
^{13}C NMR (126 MHz, $CDCl_3$) δ 154.02, 149.26, 149.19, 146.23, 137.36, 136.19, 128.20, 122.16, 95.61, 95.16, 60.25, 55.04, 31.97, 12.62, 10.26.
HRMS (ESI+): calcd for $C_{15}H_{16}BrN_3O$ $[M+H]^+$ 334.0550, found 334.0548.

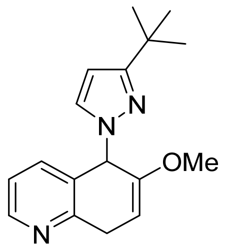

5-(3-(tert-butyl)-1H-pyrazol-1-yl)-6-methoxy-5,8-dihydroquinoline (3m): The title compound was prepared according to the general procedure A. The crude residue was purified by column chromatography on silica gel with an eluent of hexanes/ethyl acetate (2:1) to furnish the pure compound as a brown solid in 71% yield.

^{1}H NMR (500 MHz, $CDCl_3$) δ 8.63 (d, J = 4.7 Hz, 1H), 7.84 (d, J = 7.9 Hz, 1H), 7.26 (d, J = 2.2 Hz, 2H), 6.22 (d, J = 2.3 Hz, 1H), 6.18 (s, 1H), 5.43 (d, J = 4.2 Hz, 1H), 4.03 – 3.93 (m, 1H), 3.91 – 3.80 (m, 1H), 3.77 (s, 3H), 1.47 (d, J = 2.1 Hz, 9H).

^{13}C NMR (126 MHz, $CDCl_3$) δ 161.51, 153.70, 150.16, 148.97, 136.73, 129.28, 126.60, 121.87, 102.84, 96.12, 60.58, 54.96, 32.11, 31.89, 30.73.

HRMS (ESI+): calcd for $C_{17}H_{21}N_3O$ $[M+H]^+$ 284.1757, found 284.1755.

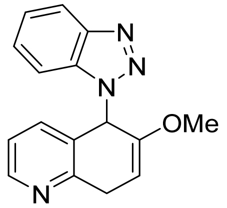

5-(1*H*-benzo[*d*][1,2,3]triazol-1-yl)-6-methoxy-5,8-dihydroquinoline (3n): The title compound was prepared according to the general procedure C. The crude residue was purified by column chromatography on silica gel with an eluent of hexanes/ethyl acetate (2:1) to furnish the pure compound as a brown solid in 25% yield.

^{1}H NMR (500 MHz, $CDCl_3$) δ 8.54 (dd, J = 4.7, 1.7 Hz, 1H), 8.19 – 7.89 (m, 1H), 7.43 (dd, J = 8.0, 1.7 Hz, 1H), 7.34 – 7.27 (m, 2H), 7.19 – 7.10 (m, 1H), 7.08 (dd, J = 8.0, 4.7 Hz, 1H), 6.85 (t, J = 4.5 Hz, 1H), 5.36 (t, J = 3.9 Hz, 1H), 4.12 – 3.98 (m, 1H), 3.95 – 3.83 (m, 1H), 3.53 (s, 3H).

^{13}C NMR (126 MHz, $CDCl_3$) δ 154.17, 149.92, 148.08, 146.80, 136.59, 131.59, 127.47, 126.80, 124.05, 122.40, 120.30, 109.96, 96.34, 58.88, 55.07, 32.14.

HRMS (ESI+): calcd for $C_{16}H_{14}N_4O$ $[M+H]^+$ 297.1240, found 297.1238.

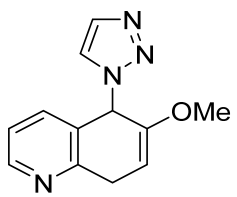

6-methoxy-5-(1*H*-1,2,3-triazol-1-yl)-5,8-dihydroquinoline (3o): The title compound was prepared according to the general procedure B. The crude residue was purified by column chromatography on silica gel with an eluent of hexanes/ethyl acetate (2:1) to furnish the pure compound as a brown solid in 39% yield.

^{1}H NMR (500 MHz, $CDCl_3$) δ 8.55 (dd, *J* = 4.6, 1.7 Hz, 1H), 7.66 (d, *J* = 1.1 Hz, 1H), 7.59 (dd, *J* = 7.9, 1.7 Hz, 1H), 7.38 (d, *J* = 1.0 Hz, 1H), 7.16 (dd, *J* = 7.9, 4.7 Hz, 1H), 6.46 (t, *J* = 3.9 Hz, 1H), 5.33 (dd, *J* = 4.5, 3.2 Hz, 1H), 3.89 (dt, *J* = 22.6, 3.7 Hz, 1H), 3.77 (dt, *J* = 22.6, 4.2 Hz, 1H), 3.61 (s, 3H).
^{13}C NMR (126 MHz, $CDCl_3$) δ 154.01, 149.79, 148.73, 136.79, 134.32, 127.11, 122.21, 121.47, 96.71, 59.56, 55.03, 31.75.
HRMS (ESI+): calcd for $C_{12}H_{12}N_4O$ $[M+H]^+$ 229.1084, found 229.1083.

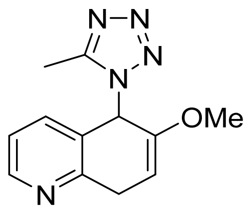

6-methoxy-5-(5-methyl-1*H*-tetrazol-1-yl)-5,8-dihydroquinoline (3p): The title compound was prepared according to the general procedure B. The crude residue was purified by column chromatography on silica gel with an eluent of hexanes/ethyl acetate (2:1) to furnish the pure compound as a brown solid in 51% yield.
^{1}H NMR (500 MHz, $(CD_3)_2CO$) δ 8.72 – 8.45 (m, 1H), 7.49 (dd, *J* = 8.0, 1.6 Hz, 1H), 7.41 – 7.08 (m, 1H), 6.60 (d, *J* = 4.2 Hz, 1H), 5.44 (t, *J* = 3.9 Hz, 1H), 3.85 (dt, *J* = 22.6, 3.9 Hz, 1H), 3.70 (dt, *J* = 22.6, 4.2 Hz, 1H), 3.61 (s, 3H), 2.49 (s, 3H).
^{13}C NMR (126 MHz, $(CD_3)_2CO$) δ 155.77, 152.80, 150.58, 148.88, 137.19, 126.92, 122.95, 97.68, 57.76, 55.41, 32.46, 9.03.
HRMS (ESI+): calcd for $C_{12}H_{13}N_5O$ $[M+H]^+$ 244.1193, found 244.1190.

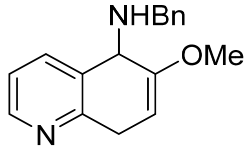

***N*-benzyl-6-methoxy-5,8-dihydroquinolin-5-amine (3q)**: The title compound was prepared according to the general procedure B but use amines (2.0 equiv) and 3,6-di-*tert*-butyl-9-mesityl-10-phenylacridin-10-ium tetrafluoroborate as photocatalyst. The crude residue was purified by column chromatography on silica gel with an eluent of hexanes/ethyl acetate (2:1) to furnish the pure compound as a brown solid in 40% yield.
^{1}H NMR (500 MHz, $CDCl_3$) δ 8.51 (dd, *J* = 4.7, 1.8 Hz, 1H), 7.95 (dd, *J* = 7.8, 1.8 Hz, 1H), 7.41 – 7.01 (m, 7H), 5.14 (t, *J* = 3.8 Hz, 1H), 4.60 (t, *J* = 4.0 Hz, 1H), 3.84 – 3.68 (m, 4H), 3.68 – 3.59 (m, 1H), 3.56 (d, *J* = 12.7 Hz, 1H), 3.27 (d, *J* = 12.7 Hz, 1H).
^{13}C NMR (126 MHz, $CDCl_3$) δ 155.59, 152.33, 148.29, 140.87, 137.26, 130.87, 128.36, 128.25, 126.89, 121.75, 93.98, 55.98, 54.60, 47.52, 32.23.
HRMS (ESI+): calcd for $C_{17}H_{18}N_2O$ $[M+H]^+$ 267.1492, found 267.1491.

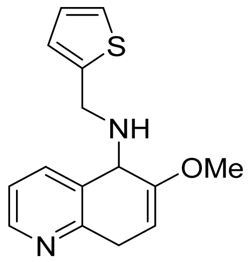

6-methoxy-N-(thiophen-2-ylmethyl)-5,8-dihydroquinolin-5-amine (3r): The title compound was prepared according to the general procedure B but use amines (2.0 equiv) and 3,6-di-*tert*-butyl-9-mesityl-10-phenylacridin-10-ium tetrafluoroborate as photocatalyst. The crude residue was purified by column chromatography on silica gel with an eluent of hexanes/ethyl acetate (2:1) to furnish the pure compound as a brown solid in 35% yield.
^{1}H NMR (500 MHz, $CDCl_3$) δ 8.47 (dd, *J* = 4.7, 1.8 Hz, 1H), 7.95 (dd, *J* = 7.9, 1.8 Hz, 1H), 7.19 (dd, *J* = 7.9, 4.7 Hz, 1H), 7.15 (dd, *J* = 5.1, 1.2 Hz, 1H), 6.89 (dd, *J* = 5.0, 3.5 Hz, 1H), 6.83 (d, *J* = 3.4 Hz, 1H), 5.11 (t, *J* = 3.8 Hz, 1H), 4.57 (t, *J* = 4.1 Hz, 1H), 3.82 (d, *J* = 13.7 Hz, 1H), 3.68 (s, 4H), 3.64 – 3.53 (m, 1H), 3.44 (d, *J* = 13.7 Hz, 1H).
^{13}C NMR (126 MHz, $CDCl_3$) δ 155.48, 152.31, 148.37, 144.85, 137.34, 130.59, 126.64, 124.43, 124.34, 121.82, 93.98, 55.74, 54.64, 42.62, 32.19.
HRMS (ESI+): calcd for $C_{15}H_{16}N_2OS$ $[M+H]^+$ 273.1056, found 273.1053.

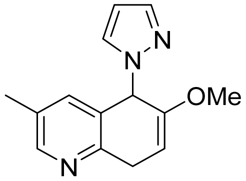

6-methoxy-3-methyl-5-(1*H*-pyrazol-1-yl)-5,8-dihydroquinoline (3s): The title compound was prepared according to the general procedure A. The crude residue was purified by column chromatography on silica gel with an eluent of hexanes/ethyl acetate (2:1) to furnish the pure compound as a brown solid in 72% yield.
^{1}H NMR (500 MHz, $CDCl_3$) δ 8.33 (d, *J* = 2.2 Hz, 1H), 7.51 (d, *J* = 1.8 Hz, 1H), 7.34 (d, *J* = 2.1 Hz, 1H), 7.33 (d, *J* = 2.3 Hz, 1H), 6.24 (t, *J* = 2.1 Hz, 1H), 5.99 (t, *J* = 3.9 Hz, 1H), 5.25 (dd, *J* = 4.6, 3.1 Hz, 1H), 3.83 (dt, *J* = 22.3, 3.7 Hz, 1H), 3.66 (dt, *J* = 22.3, 4.2 Hz, 1H), 3.59 (s, 3H), 2.23 (s, 3H).
^{13}C NMR (126 MHz, $CDCl_3$) δ 151.12, 150.08, 149.92, 139.47, 136.81, 131.56, 128.00, 127.85, 106.26, 96.10, 61.05, 55.04, 31.44, 18.09.
HRMS (ESI+): calcd for $C_{14}H_{15}N_3O$ $[M+H]^+$ 242.1288, found 242.1285.

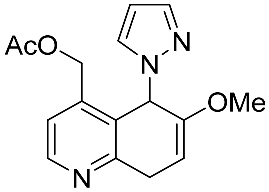

(6-methoxy-5-(1*H*-pyrazol-1-yl)-5,8-dihydroquinolin-4-yl)methyl acetate (3t): The title compound was prepared according to the general procedure A. The crude residue was

purified by column chromatography on silica gel with an eluent of hexanes/ethyl acetate (2:1) to furnish the pure compound as a brown solid in 62% yield.

^{1}H NMR (400 MHz, $CDCl_3$) δ 8.54 (d, *J* = 5.0 Hz, 1H), 7.47 (d, *J* = 1.8 Hz, 1H), 7.33 (d, *J* = 2.4 Hz, 1H), 7.19 (d, *J* = 4.7 Hz, 1H), 6.22 (t, *J* = 2.1 Hz, 1H), 6.17 (t, *J* = 2.9 Hz, 1H), 5.18 (dd, *J* = 5.0, 2.6 Hz, 1H), 5.13 (d, *J* = 14.2 Hz, 1H), 4.65 (d, *J* = 14.2 Hz, 1H), 4.01 – 3.90 (m, 1H), 3.73 (ddd, *J* = 22.5, 5.1, 2.5 Hz, 1H), 3.60 (s, 3H), 2.10 (s, 3H).

^{13}C NMR (101 MHz, $CDCl_3$) δ 170.36, 156.06, 150.88, 149.33, 145.10, 139.58, 128.71, 126.07, 121.05, 106.19, 94.74, 61.96, 58.74, 55.16, 32.22, 20.91.

HRMS (ESI+): calcd for $C_{16}H_{17}N_3O_3$ $[M+H]^+$ 300.1343, found 300.1340.

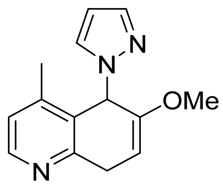

6-methoxy-4-methyl-5-(1*H*-pyrazol-1-yl)-5,8-dihydroquinoline (3u): The title compound was prepared according to the general procedure A. The crude residue was purified by column chromatography on silica gel with an eluent of hexanes/ethyl acetate (2:1) to furnish the pure compound as a brown solid in 62% yield.

^{1}H NMR (500 MHz, $CDCl_3$) δ 8.39 (d, *J* = 4.9 Hz, 1H), 7.46 (d, *J* = 1.8 Hz, 1H), 7.23 (d, *J* = 2.4 Hz, 1H), 6.97 (d, *J* = 4.9 Hz, 1H), 6.20 (t, *J* = 2.1 Hz, 1H), 6.06 (dd, *J* = 3.7, 2.5 Hz, 1H), 5.13 (dd, *J* = 5.2, 2.7 Hz, 1H), 3.91 (dt, *J* = 22.3, 3.2 Hz, 1H), 3.67 (ddd, *J* = 22.3, 5.2, 2.5 Hz, 1H), 3.58 (s, 3H), 2.09 (s, 3H).

^{13}C NMR (126 MHz, $CDCl_3$) δ 155.65, 151.41, 149.00, 147.68, 139.02, 128.40, 127.16, 124.09, 105.93, 94.74, 59.48, 55.09, 32.36, 18.82.

HRMS (ESI+): calcd for $C_{14}H_{15}N_3O$ $[M+H]^+$ 242.1288, found 242.1286.

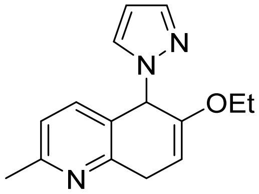

6-ethoxy-2-methyl-5-(1*H*-pyrazol-1-yl)-5,8-dihydroquinoline (3v): The title compound was prepared according to the general procedure A. The crude residue was purified by column chromatography on silica gel with an eluent of hexanes/ethyl acetate (2:1) to furnish the pure compound as a brown solid in 51% yield.

^{1}H NMR (500 MHz, $CDCl_3$) δ 7.49 (d, *J* = 1.8 Hz, 1H), 7.46 (d, *J* = 7.9 Hz, 1H), 7.37 (d, *J* = 2.2 Hz, 1H), 6.98 (d, *J* = 8.0 Hz, 1H), 6.22 (t, *J* = 2.1 Hz, 1H), 5.96 (t, *J* = 3.9 Hz, 1H), 5.23 (dd, *J* = 4.7, 3.1 Hz, 1H), 3.92 – 3.72 (m, 3H), 3.65 (dt, *J* = 22.3, 4.2 Hz, 1H), 2.52 (s, 3H), 1.21 (t, *J* = 7.0 Hz, 3H).

^{13}C NMR (126 MHz, $CDCl_3$) δ 157.94, 153.49, 149.28, 139.43, 136.99, 127.96, 125.79, 121.83, 105.96, 96.49, 63.11, 61.10, 32.05, 24.40, 14.50.

HRMS (ESI+): calcd for $C_{15}H_{17}N_3O$ $[M+H]^+$ 256.1444, found 256.1441.

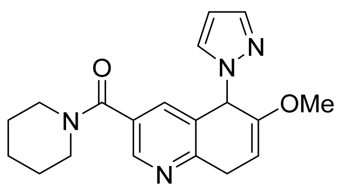

(6-methoxy-5-(1*H*-pyrazol-1-yl)-5,8-dihydroquinolin-3-yl)(piperidin-1-yl)methanone (3w): The title compound was prepared according to the general procedure C. The crude residue was purified by column chromatography on silica gel with an eluent of hexanes/ethyl acetate (2:1) to furnish the pure compound as a brown solid in 22% yield.

^{1}H NMR (400 MHz, $CDCl_3$) δ 8.57 (d, *J* = 2.1 Hz, 1H), 7.55 (d, *J* = 2.0 Hz, 1H), 7.50 (d, *J* = 1.8 Hz, 1H), 7.38 (d, *J* = 2.4 Hz, 1H), 6.25 (d, *J* = 2.1 Hz, 1H), 6.03 (t, *J* = 4.0 Hz, 1H), 5.28 (t, *J* = 3.9 Hz, 1H), 3.90 (d, *J* = 26.5 Hz, 1H), 3.83 – 3.68 (m, 1H), 3.63 (s, 4H), 3.21 (s, 2H), 1.54 (d, *J* = 90.0 Hz, 6H).

^{13}C NMR (101 MHz, $CDCl_3$) δ 167.29, 155.32, 149.78, 147.65, 139.91, 135.32, 130.44, 128.32, 128.29, 106.38, 95.86, 60.72, 55.16, 43.43, 31.92, 29.84, 24.56.

HRMS (ESI+): calcd for $C_{19}H_{22}N_4O_2$ $[M+H]^+$ 339.1816, found 339.1813.

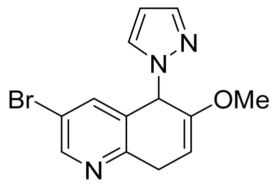

3-bromo-6-methoxy-5-(1*H*-pyrazol-1-yl)-5,8-dihydroquinoline (3x): The title compound was prepared according to the general procedure C. The crude residue was purified by column chromatography on silica gel with an eluent of hexanes/ethyl acetate (2:1) to furnish the pure compound as a brown solid in 43% yield.

^{1}H NMR (400 MHz, $CDCl_3$) δ 8.56 (d, *J* = 2.2 Hz, 1H), 7.69 (d, *J* = 2.2 Hz, 1H), 7.53 (d, *J* = 1.8 Hz, 1H), 7.37 (d, *J* = 2.3 Hz, 1H), 6.27 (t, *J* = 2.2 Hz, 1H), 5.99 (t, *J* = 4.0 Hz, 1H), 5.26 (dd, *J* = 4.6, 3.2 Hz, 1H), 3.84 (dt, *J* = 22.6, 3.7 Hz, 1H), 3.67 (dt, *J* = 22.6, 4.2 Hz, 1H), 3.62 (s, 3H).

^{13}C NMR (101 MHz, $CDCl_3$) δ 152.81, 150.34, 149.66, 140.03, 138.86, 130.27, 128.14, 118.47, 106.52, 95.84, 60.63, 55.19, 31.48.

HRMS (ESI+): calcd for $C_{13}H_{12}BrN_3O$ $[M+H]^+$ 306.0237, found 306.0233.

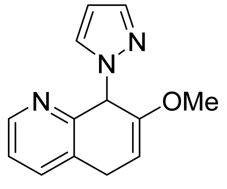

7-methoxy-8-(1*H*-pyrazol-1-yl)-5,8-dihydroquinoline (3y): The title compound was prepared according to the general procedure C. The crude residue was purified by column chromatography on silica gel with an eluent of hexanes/ethyl acetate (2:1) to furnish the pure compound as a brown solid in 45% yield.

^{1}H NMR (500 MHz, $CDCl_3$) δ 8.77 – 8.28 (m, 1H), 7.65 (d, *J* = 2.3 Hz, 1H), 7.61 – 7.52 (m, 1H), 7.49 (d, *J* = 1.9 Hz, 1H), 7.17 (dd, *J* = 7.8, 4.6 Hz, 1H), 6.23 (t, *J* = 2.1 Hz, 1H), 5.91 (t,

J = 3.7 Hz, 1H), 5.22 (dd, *J* = 4.9, 2.9 Hz, 1H), 3.91 – 3.74 (m, 1H), 3.61 (s, 3H), 3.52 (dt, *J* = 21.5, 4.0 Hz, 1H).
^{13}C NMR (126 MHz, $CDCl_3$) δ 151.65, 151.39, 148.38, 140.15, 136.28, 135.90, 130.28, 122.85, 105.09, 95.21, 62.97, 55.01, 28.15.
HRMS (ESI+): calcd for $C_{13}H_{13}N_3O$ $[M+H]^+$ 228.1131, found 228.1130.

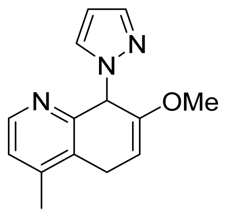

7-methoxy-4-methyl-8-(1*H*-pyrazol-1-yl)-5,8-dihydroquinoline (3z): The title compound was prepared according to the general procedure B. The crude residue was purified by column chromatography on silica gel with an eluent of hexanes/ethyl acetate (2:1) to furnish the pure compound as a brown solid in 43% yield.
^{1}H NMR (400 MHz, $CDCl_3$) δ 8.33 (d, *J* = 4.8 Hz, 1H), 7.66 (dd, *J* = 2.3, 0.7 Hz, 1H), 7.49 (d, *J* = 1.8 Hz, 1H), 7.02 (d, *J* = 4.8 Hz, 1H), 6.22 (t, *J* = 2.0 Hz, 1H), 5.92 (t, *J* = 3.6 Hz, 1H), 5.23 (dd, *J* = 4.8, 3.0 Hz, 1H), 3.73 – 3.63 (m, 1H), 3.62 (s, 3H), 3.44 (ddd, *J* = 21.6, 4.8, 3.3 Hz, 1H), 2.33 (s, 3H).
^{13}C NMR (101 MHz, $CDCl_3$) δ 151.29, 151.21, 148.12, 145.63, 140.17, 130.43, 129.28, 124.38, 104.98, 94.89, 63.08, 54.98, 26.19, 19.02.
HRMS (ESI+): calcd for $C_{14}H_{15}N_3O$ $[M+H]^+$ 242.1288, found 242.1286.

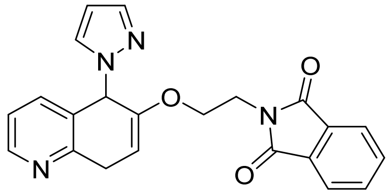

2-(2-((5-(1*H*-pyrazol-1-yl)-5,8-dihydroquinolin-6-yl)oxy)ethyl)isoindoline-1,3-dione (3aa): The title compound was prepared according to the general procedure A. The crude residue was purified by column chromatography on silica gel with an eluent of hexanes/ethyl acetate (2:1) to furnish the pure compound as a brown solid in 41% yield.
^{1}H NMR (500 MHz, $CDCl_3$) δ 8.49 (dd, *J* = 4.7, 1.7 Hz, 1H), 7.81 (dd, *J* = 5.4, 3.1 Hz, 2H), 7.72 (dd, *J* = 5.4, 3.1 Hz, 2H), 7.45 (dd, *J* = 7.9, 1.7 Hz, 1H), 7.31 (d, *J* = 2.3 Hz, 1H), 7.14 (d, *J* = 1.8 Hz, 1H), 7.09 (dd, *J* = 7.9, 4.7 Hz, 1H), 5.91 (t, *J* = 4.0 Hz, 1H), 5.83 (t, *J* = 2.1 Hz, 1H), 5.26 (dd, *J* = 4.6, 3.2 Hz, 1H), 4.13 – 3.93 (m, 3H), 3.91 – 3.78 (m, 2H), 3.67 (dt, *J* = 22.5, 4.2 Hz, 1H).
^{13}C NMR (126 MHz, $CDCl_3$) δ 168.07, 154.14, 149.22, 148.71, 139.52, 136.60, 134.00, 132.16, 128.60, 128.32, 123.41, 122.01, 105.52, 97.25, 64.22, 60.67, 36.92, 31.87.
HRMS (ESI+): calcd for $C_{22}H_{18}N_4O_3$ $[M+H]^+$ 387.1452, found 387.1447.

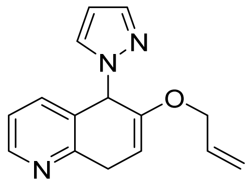

6-(allyloxy)-5-(1*H*-pyrazol-1-yl)-5,8-dihydroquinoline (3ab): The title compound was prepared according to the general procedure A. The crude residue was purified by column chromatography on silica gel with an eluent of hexanes/ethyl acetate (2:1) to furnish the pure compound as a yellow oil in 59% yield.

^{1}H NMR (500 MHz, $CDCl_3$) δ 8.50 (dd, *J* = 4.7, 1.6 Hz, 1H), 7.55 (dd, *J* = 7.9, 1.7 Hz, 1H), 7.50 (d, *J* = 1.8 Hz, 1H), 7.39 (d, *J* = 2.3 Hz, 1H), 7.12 (dd, *J* = 7.9, 4.7 Hz, 1H), 6.23 (t, *J* = 2.1 Hz, 1H), 6.03 (t, *J* = 4.0 Hz, 1H), 5.84 (ddt, *J* = 17.1, 10.4, 5.1 Hz, 1H), 5.33 – 5.25 (m, 1H), 5.24 – 5.10 (m, 2H), 4.33 (tdd, *J* = 13.1, 11.4, 5.1 Hz, 2H), 3.87 (dt, *J* = 22.4, 3.7 Hz, 1H), 3.70 (dt, *J* = 22.4, 4.2 Hz, 1H).

^{13}C NMR (126 MHz, $CDCl_3$) δ 154.24, 149.26, 148.69, 139.61, 136.62, 132.92, 128.62, 128.18, 122.00, 117.21, 106.10, 97.30, 68.22, 61.10, 32.00.

HRMS (ESI+): calcd for $C_{15}H_{15}N_3O$ $[M+H]^+$ 254.1288, found 254.1286.

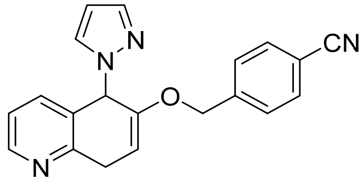

4-(((5-(1*H*-pyrazol-1-yl)-5,8-dihydroquinolin-6-yl)oxy)methyl)benzonitrile (3ac): The title compound was prepared according to the general procedure A. The crude residue was purified by column chromatography on silica gel with an eluent of hexanes/ethyl acetate (2:1) to furnish the pure compound as a brown solid in 83% yield.

^{1}H NMR (400 MHz, $CDCl_3$) δ 8.52 (d, *J* = 4.7 Hz, 1H), 7.65 – 7.48 (m, 4H), 7.39 (d, *J* = 2.4 Hz, 1H), 7.21 (d, *J* = 7.9 Hz, 2H), 7.14 (dd, *J* = 7.9, 4.7 Hz, 1H), 6.28 (t, *J* = 2.1 Hz, 1H), 6.11 (t, *J* = 4.1 Hz, 1H), 5.29 (t, *J* = 3.8 Hz, 1H), 4.92 (q, *J* = 13.1 Hz, 2H), 4.10 – 3.80 (m, 1H), 3.77 – 3.60 (m, 1H).

^{13}C NMR (126 MHz, $CDCl_3$) δ 153.83, 149.34, 148.58, 142.06, 139.77, 136.68, 132.36, 128.46, 128.27, 127.20, 122.17, 118.74, 111.65, 106.31, 98.01, 68.26, 60.87, 31.82.

HRMS (ESI+): calcd for $C_{20}H_{16}N_4O$ $[M+H]^+$ 329.1397, found 329.1395.

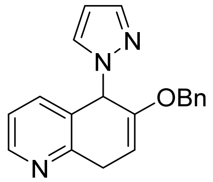

6-(benzyloxy)-5-(1*H*-pyrazol-1-yl)-5,8-dihydroquinoline (3ad): The title compound was prepared according to the general procedure A. The crude residue was purified by column chromatography on silica gel with an eluent of hexanes/ethyl acetate (2:1) to furnish the pure compound as a brown solid in 67% yield.

^{1}H NMR (400 MHz, $CDCl_3$) δ 8.54 (dd, *J* = 4.7, 1.7 Hz, 1H), 7.63 – 7.52 (m, 2H), 7.44 (d, *J* = 2.3 Hz, 1H), 7.37 – 7.25 (m, 3H), 7.22 – 7.09 (m, 3H), 6.30 (t, *J* = 2.1 Hz, 1H), 6.13 (t, *J* = 4.0 Hz, 1H), 5.37 (dd, *J* = 4.5, 3.2 Hz, 1H), 4.91 (q, *J* = 12.1 Hz, 2H), 3.92 (dt, *J* = 22.5, 3.7 Hz, 1H), 3.74 (dt, *J* = 22.5, 4.2 Hz, 1H).
^{13}C NMR (101 MHz, $CDCl_3$) δ 154.22, 149.28, 148.85, 139.66, 136.73, 136.62, 128.53, 128.51, 128.36, 127.86, 127.03, 121.99, 106.08, 97.72, 69.42, 61.09, 32.02.
HRMS (ESI+): calcd for $C_{19}H_{17}N_3O$ $[M+H]^+$ 304.1444, found 304.1443.

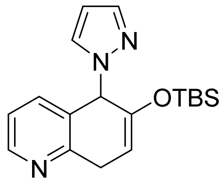

6-((*tert*-butyldimethylsilyl)oxy)-5-(1*H*-pyrazol-1-yl)-5,8-dihydroquinoline (3ae): The title compound was prepared according to the general procedure A. The crude residue was purified by column chromatography on silica gel with an eluent of hexanes/ethyl acetate (2:1) to furnish the pure compound as a brown solid in 79% yield.
^{1}H NMR (500 MHz, $CDCl_3$) δ 8.79 – 8.23 (m, 1H), 7.57 – 7.41 (m, 2H), 7.36 (d, *J* = 2.3 Hz, 1H), 7.10 (dd, *J* = 7.9, 4.7 Hz, 1H), 6.24 (t, *J* = 2.1 Hz, 1H), 5.92 (t, *J* = 4.6 Hz, 1H), 5.38 (t, *J* = 3.9 Hz, 1H), 3.84 (dt, *J* = 22.6, 4.0 Hz, 1H), 3.68 (dt, *J* = 22.6, 4.4 Hz, 1H), 0.78 (s, 9H), 0.16 (s, 3H), -0.01 (s, 3H).
^{13}C NMR (126 MHz, $CDCl_3$) δ 153.63, 149.14, 145.18, 139.35, 136.51, 129.14, 128.30, 121.99, 106.15, 104.29, 62.23, 32.37, 25.52, 17.93, -4.41, -4.98.
HRMS (ESI+): calcd for $C_{18}H_{25}N_3OSi$ $[M+H]^+$ 328.1840, found 328.1836.

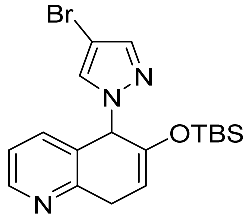

5-(4-bromo-1*H*-pyrazol-1-yl)-6-((tert-butyldimethylsilyl)oxy)-5,8-dihydroquinoline (3af): The title compound was prepared according to the general procedure A. The crude residue was purified by column chromatography on silica gel with an eluent of hexanes/ethyl acetate (2:1) to furnish the pure compound as a brown solid in % yield.
^{1}H NMR (500 MHz, $CDCl_3$) δ 8.51 (dd, *J* = 4.7, 1.7 Hz, 1H), 7.57 (s, 1H), 7.48 (dd, *J* = 8.0, 1.7 Hz, 1H), 7.44 (s, 1H), 7.37 (s, 1H), 7.13 (dd, *J* = 7.9, 4.6 Hz, 1H), 5.86 (t, *J* = 4.6 Hz, 1H), 5.38 (td, *J* = 3.8, 3.4, 0.9 Hz, 1H), 3.83 (dt, *J* = 22.7, 4.0 Hz, 1H), 3.68 (dt, *J* = 22.8, 4.4 Hz, 1H), 0.80 (s, 9H), 0.17 (s, 3H), 0.03 (s, 3H).
^{13}C NMR (126 MHz, $CDCl_3$) δ 153.65, 149.43, 144.65, 139.87, 136.57, 128.58, 128.39, 122.11, 104.65, 93.86, 63.10, 32.28, 25.47, 17.93, -4.36.
HRMS (ESI+): calcd for $C_{18}H_{24}BrN_3OSi$ $[M+K]^+$ 444.0504, found 444.0507.

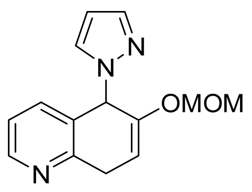

6-(methoxymethoxy)-5-(1*H*-pyrazol-1-yl)-5,8-dihydroquinoline (3ag): The title compound was prepared according to the general procedure A. The crude residue was purified by column chromatography on silica gel with an eluent of hexanes/ethyl acetate (2:1) to furnish the pure compound as a yellow oil in 81% yield.

^{1}H NMR (500 MHz, $CDCl_3$) δ 8.50 (d, *J* = 4.7 Hz, 1H), 7.54 (d, *J* = 7.9 Hz, 1H), 7.49 (d, *J* = 1.8 Hz, 1H), 7.36 (d, *J* = 2.3 Hz, 1H), 7.11 (dd, *J* = 7.9, 4.7 Hz, 1H), 6.23 (d, *J* = 2.2 Hz, 1H), 6.04 (t, *J* = 4.2 Hz, 1H), 5.60 (t, *J* = 3.9 Hz, 1H), 5.12 – 4.68 (m, 2H), 3.86 (dt, *J* = 22.6, 3.9 Hz, 1H), 3.69 (dt, *J* = 22.7, 4.3 Hz, 1H), 3.24 (s, 3H).

^{13}C NMR (126 MHz, $CDCl_3$) δ 153.84, 149.33, 147.01, 139.45, 136.60, 128.49, 127.83, 122.03, 106.35, 100.87, 93.73, 60.80, 56.04, 31.96.

HRMS (ESI+): calcd for $C_{14}H_{15}N_3O_2$ $[M+H]^+$ 258.1237, found 258.1236.

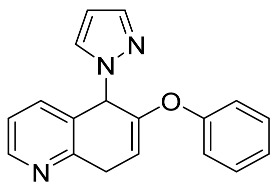

6-phenoxy-5-(1*H*-pyrazol-1-yl)-5,8-dihydroquinoline (3ah): The title compound was prepared according to the general procedure A. The crude residue was purified by column chromatography on silica gel with an eluent of hexanes/ethyl acetate (2:1) to furnish the pure compound as a brown solid in 42% yield.

^{1}H NMR (400 MHz, $CDCl_3$) δ 8.53 (dd, *J* = 4.7, 1.7 Hz, 1H), 7.58 (dd, *J* = 7.9, 1.7 Hz, 1H), 7.54 (d, *J* = 1.9 Hz, 1H), 7.49 (d, *J* = 2.4 Hz, 1H), 7.39 – 7.27 (m, 2H), 7.20 – 7.03 (m, 2H), 7.00 – 6.88 (m, 2H), 6.29 (t, *J* = 2.1 Hz, 1H), 6.22 (t, *J* = 4.3 Hz, 1H), 5.43 (t, *J* = 3.9 Hz, 1H), 4.08 – 3.76 (m, 1H), 3.69 (dt, *J* = 22.8, 4.3 Hz, 1H).

^{13}C NMR (101 MHz, $CDCl_3$) δ 155.06, 153.82, 149.52, 149.25, 139.96, 136.80, 129.84, 128.49, 128.46, 124.45, 122.18, 120.41, 106.41, 105.76, 60.19, 32.12.

HRMS (ESI+): calcd for $C_{18}H_{15}N_3O$ $[M+H]^+$ 290.1288, found 290.1285.

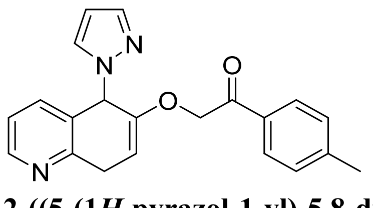

2-((5-(1*H*-pyrazol-1-yl)-5,8-dihydroquinolin-6-yl)oxy)-1-(p-tolyl)ethan-1-one (3ai): The title compound was prepared according to the general procedure A. The crude residue was purified by column chromatography on silica gel with an eluent of hexanes/ethyl acetate (2:1) to furnish the pure compound as a brown solid in 74% yield.

^{1}H NMR (500 MHz, $CDCl_3$) δ 8.49 (d, *J* = 4.8 Hz, 1H), 7.73 (d, *J* = 8.0 Hz, 2H), 7.61 – 7.42 (m, 3H), 7.20 (d, *J* = 7.9 Hz, 2H), 7.10 (dd, *J* = 7.9, 4.7 Hz, 1H), 6.23 (t, *J* = 2.1 Hz, 1H),

6.12 (t, J = 4.0 Hz, 1H), 5.23 (t, J = 3.9 Hz, 1H), 5.00 (d, J = 2.5 Hz, 2H), 3.84 (dt, J = 22.5, 3.8 Hz, 1H), 3.77 – 3.49 (m, 1H), 2.39 (s, 3H).
^{13}C NMR (126 MHz, $CDCl_3$) δ 194.10, 153.81, 149.31, 148.37, 144.86, 139.72, 136.53, 132.00, 129.53, 128.60, 128.39, 122.02, 106.16, 98.42, 70.61, 60.80, 31.87, 21.84.
HRMS (ESI+): calcd for $C_{21}H_{19}N_3O_2$ $[M+H]^+$ 346.1550, found 346.1546.

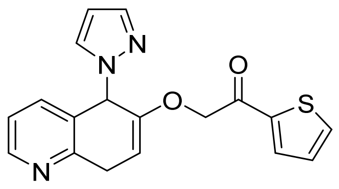

2-((5-(1*H*-pyrazol-1-yl)-5,8-dihydroquinolin-6-yl)oxy)-1-(thiophen-2-yl)ethan-1-one (3aj): The title compound was prepared according to the general procedure A. The crude residue was purified by column chromatography on silica gel with an eluent of hexanes/ethyl acetate (2:1) to furnish the pure compound as a brown solid in 86% yield.
^{1}H NMR (500 MHz, $CDCl_3$) δ 8.49 (dd, J = 4.7, 1.7 Hz, 1H), 7.63 (d, J = 4.9 Hz, 1H), 7.54 (d, J = 3.9 Hz, 1H), 7.51 (dd, J = 7.2, 1.8 Hz, 2H), 7.42 (d, J = 2.4 Hz, 1H), 7.10 (dd, J = 7.9, 4.7 Hz, 1H), 7.02 (t, J = 4.4 Hz, 1H), 6.24 (t, J = 2.1 Hz, 1H), 6.15 (t, J = 4.2 Hz, 1H), 5.29 (t, J = 3.9 Hz, 1H), 4.85 (d, J = 2.7 Hz, 2H), 4.06 – 3.74 (m, 1H), 3.67 (dt, J = 22.5, 4.3 Hz, 1H).
^{13}C NMR (126 MHz, $CDCl_3$) δ 187.97, 153.64, 149.41, 148.28, 140.65, 139.67, 136.44, 134.67, 133.57, 128.49, 128.42, 128.24, 122.06, 106.38, 98.60, 71.19, 60.76, 31.84.
HRMS (ESI+): calcd for $C_{18}H_{15}N_3O_2S$ $[M+H]^+$ 338.0958, found 338.0952.

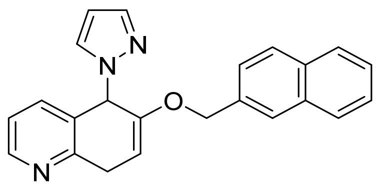

6-(naphthalen-2-ylmethoxy)-5-(1*H*-pyrazol-1-yl)-5,8-dihydroquinoline (3ak): The title compound was prepared according to the general procedure A. The crude residue was purified by column chromatography on silica gel with an eluent of hexanes/ethyl acetate (2:1) to furnish the pure compound as a brown solid in 37% yield.
^{1}H NMR (500 MHz, $CDCl_3$) δ 8.38 (d, J = 4.6 Hz, 1H), 7.62 (dt, J = 12.4, 6.5 Hz, 3H), 7.44 (t, J = 10.8 Hz, 3H), 7.36 – 7.21 (m, 3H), 7.19 – 7.04 (m, 1H), 7.01 (dd, J = 7.9, 4.7 Hz, 1H), 6.14 (s, 1H), 5.97 (d, J = 4.2 Hz, 1H), 5.20 (d, J = 4.0 Hz, 1H), 5.00 – 4.67 (m, 2H), 3.73 (dt, J = 22.3, 3.9 Hz, 1H), 3.56 (dt, J = 22.5, 4.3 Hz, 1H).
^{13}C NMR (126 MHz, $CDCl_3$) δ 153.97, 139.80 (2C), 137.22, 134.17, 133.31, 133.11, 128.92, 128.53, 128.36, 127.99, 127.82, 126.72, 126.32, 126.14, 125.89, 124.94, 122.23, 106.25, 97.63, 69.56, 61.03, 31.72.
HRMS (ESI+): calcd for $C_{23}H_{19}N_3O$ $[M+H]^+$ 354.1601, found 354.1597.

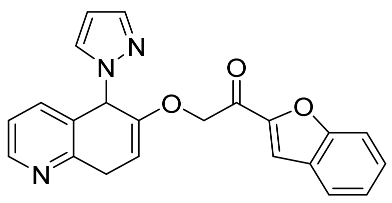

2-((5-(1*H*-pyrazol-1-yl)-5,8-dihydroquinolin-6-yl)oxy)-1-(benzofuran-2-yl)ethan-1-one (3al): The title compound was prepared according to the general procedure A. The crude residue was purified by column chromatography on silica gel with an eluent of hexanes/ethyl acetate (2:1) to furnish the pure compound as a brown solid in 85% yield.

^{1}H NMR (500 MHz, $CDCl_3$) δ 8.76 – 8.40 (m, 1H), 7.64 (d, *J* = 7.9 Hz, 1H), 7.60 – 7.52 (m, 3H), 7.50 – 7.40 (m, 2H), 7.36 – 7.22 (m, 2H), 7.13 (dd, *J* = 7.9, 4.7 Hz, 1H), 6.26 (t, *J* = 2.1 Hz, 1H), 6.19 (t, *J* = 4.1 Hz, 1H), 5.51 – 5.18 (m, 1H), 5.10 – 4.68 (m, 2H), 3.87 (dt, *J* = 22.5, 3.8 Hz, 1H), 3.70 (dt, *J* = 22.6, 4.2 Hz, 1H).

^{13}C NMR (126 MHz, $CDCl_3$) δ 185.42, 155.56, 153.43, 149.93, 149.16, 148.18, 139.68, 136.66, 128.93, 128.56, 128.30, 126.82, 124.07, 123.73, 122.13, 115.67, 112.46, 106.45, 98.44, 70.71, 60.68, 31.62.

HRMS (ESI+): calcd for $C_{22}H_{17}N_3O_3$ $[M+H]^+$ 372.1343, found 372.1339.

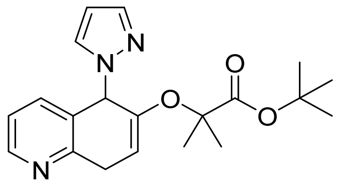

***tert*-butyl 2-((5-(1*H*-pyrazol-1-yl)-5,8-dihydroquinolin-6-yl)oxy)-2-methylpropanoate (3am):** The title compound was prepared according to the general procedure A. The crude residue was purified by column chromatography on silica gel with an eluent of hexanes/ethyl acetate (2:1) to furnish the pure compound as a brown solid in 63% yield.

^{1}H NMR (500 MHz, $CDCl_3$) δ 8.49 (dd, *J* = 4.7, 1.8 Hz, 1H), 7.57 (dd, *J* = 7.9, 1.7 Hz, 1H), 7.48 (d, *J* = 1.8 Hz, 1H), 7.44 (d, *J* = 2.2 Hz, 1H), 7.11 (dd, *J* = 7.9, 4.8 Hz, 1H), 6.21 (t, *J* = 2.0 Hz, 1H), 5.95 (t, *J* = 4.0 Hz, 1H), 5.08 (dd, *J* = 4.7, 3.1 Hz, 1H), 3.82 (dt, *J* = 22.4, 3.8 Hz, 1H), 3.64 (dt, *J* = 22.4, 4.2 Hz, 1H), 1.44 (s, 9H), 1.43 (s, 3H), 1.33 (s, 3H).

^{13}C NMR (126 MHz, $CDCl_3$) δ 173.05, 154.00, 149.22, 145.20, 139.42, 136.73, 128.71, 128.46, 121.97, 105.76, 100.96, 81.80, 78.97, 61.47, 32.06, 27.96, 25.77, 23.73.

HRMS (ESI+): calcd for $C_{20}H_{25}N_3O_3$ $[M+H]^+$ 356.1969, found 356.1963.

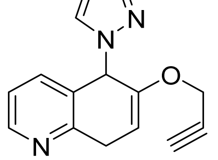

6-(prop-2-yn-1-yloxy)-5-(1*H*-pyrazol-1-yl)-5,8-dihydroquinoline (3an): The title compound was prepared according to the general procedure A. The crude residue was purified by column chromatography on silica gel with an eluent of hexanes/ethyl acetate (2:1) to furnish the pure compound as a brown solid in 31% yield.

^{1}H NMR (500 MHz, $CDCl_3$) δ 8.59 (d, *J* = 5.0 Hz, 1H), 7.73 (dd, *J* = 8.0, 1.7 Hz, 1H), 7.52 (d, *J* = 1.8 Hz, 1H), 7.42 (d, *J* = 2.4 Hz, 1H), 7.26 (s, 1H), 6.27 (t, *J* = 2.1 Hz, 1H), 6.07 (t, *J* = 3.9 Hz, 1H), 5.47 (dd, *J* = 4.6, 3.2 Hz, 1H), 4.52 (t, *J* = 2.1 Hz, 2H), 3.98 (dt, *J* = 22.7, 3.7 Hz, 1H), 3.84 (dt, *J* = 22.6, 4.2 Hz, 1H), 2.49 (t, *J* = 2.4 Hz, 1H).
^{13}C NMR (126 MHz, $CDCl_3$) δ 153.02, 147.63, 147.45, 139.99, 138.56, 129.68, 128.25, 122.72, 106.51, 98.24, 77.93, 75.94, 60.39, 55.42, 30.77.
HRMS (ESI+): calcd for $C_{15}H_{13}N_3O$ $[M+H]^+$ 252.1131, found 252.1129.

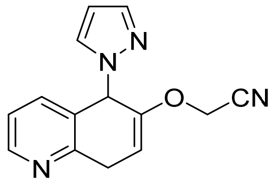

2-((5-(1*H*-pyrazol-1-yl)-5,8-dihydroquinolin-6-yl)oxy)acetonitrile (3ao): The title compound was prepared according to the general procedure A. The crude residue was purified by column chromatography on silica gel with an eluent of hexanes/ethyl acetate (2:1) to furnish the pure compound as a brown solid in 32% yield.
^{1}H NMR (500 MHz, $CDCl_3$) δ 8.54 (dd, *J* = 4.7, 1.7 Hz, 1H), 7.73 – 7.44 (m, 2H), 7.38 (d, *J* = 2.4 Hz, 1H), 7.16 – 7.08 (m, 1H), 6.28 (t, *J* = 2.1 Hz, 1H), 6.08 (t, *J* = 4.1 Hz, 1H), 5.51 (t, *J* = 3.9 Hz, 1H), 4.75 – 4.34 (m, 2H), 3.94 (dt, *J* = 22.6, 3.8 Hz, 1H), 3.79 (dt, *J* = 22.6, 4.3 Hz, 1H).
^{13}C NMR (126 MHz, $CDCl_3$) δ 153.08, 149.60, 147.39, 139.85, 136.43, 128.08, 127.82, 122.21, 114.41, 106.65, 100.31, 60.28, 52.47, 31.68.
HRMS (ESI+): calcd for $C_{14}H_{12}N_4O$ $[M+H]^+$ 253.1084, found 253.1080.

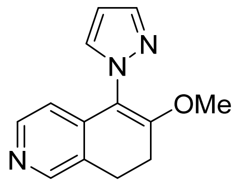

6-methoxy-5-(1*H*-pyrazol-1-yl)-7,8-dihydroisoquinoline (3ap): The title compound was prepared according to the general procedure B. The crude residue was purified by column chromatography on silica gel with an eluent of hexanes/ethyl acetate (1:2) with ammonium hydroxide as additive to furnish the pure compound as a brown solid in 43% yield.
^{1}H NMR (500 MHz, $CDCl_3$) δ 8.26 (d, *J* = 28.3 Hz, 2H), 7.74 (d, *J* = 1.9 Hz, 1H), 7.50 (d, *J* = 2.3 Hz, 1H), 6.43 (t, *J* = 2.2 Hz, 1H), 6.25 (d, *J* = 5.1 Hz, 1H), 3.61 (s, 3H), 3.01 (t, *J* = 8.2 Hz, 2H), 2.74 (dd, *J* = 8.9, 7.4 Hz, 2H).
^{13}C NMR (126 MHz, $CDCl_3$) δ 158.60, 148.60, 146.70, 142.73, 140.82, 132.94, 125.68, 115.51, 114.57, 106.33, 56.55, 24.91, 24.42.
HRMS (ESI+): calcd for $C_{13}H_{13}N_3O$ $[M+H]^+$ 228.1131, found 228.1130.

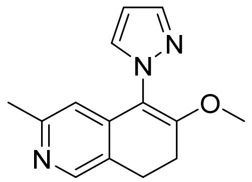

6-methoxy-3-methyl-5-(1*H*-pyrazol-1-yl)-7,8-dihydroisoquinoline (3aq): The title compound was prepared according to the general procedure B. The crude residue was purified by column chromatography on silica gel with an eluent of hexanes/ethyl acetate (1:1) with ammonium hydroxide as additive to furnish the pure compound as a brown solid in 39% yield.

^{1}H NMR (500 MHz, $CDCl_3$) δ 8.17 (s, 1H), 7.75 (d, *J* = 1.9 Hz, 1H), 7.49 (d, *J* = 2.3 Hz, 1H), 6.44 (t, *J* = 2.2 Hz, 1H), 6.11 (s, 1H), 3.60 (s, 3H), 2.97 (t, *J* = 8.1 Hz, 2H), 2.73 (t, *J* = 8.1 Hz, 2H), 2.37 (s, 3H).

^{13}C NMR (126 MHz, $CDCl_3$) δ 158.87, 157.03, 145.55, 143.28, 140.81, 132.93, 123.01, 115.02, 114.75, 106.28, 56.51, 24.92, 24.09, 24.01.

HRMS (ESI+): calcd for $C_{14}H_{15}N_3O$ $[M+H]^+$ 242.1288, found 242.1285.

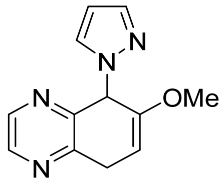

6-methoxy-5-(1*H*-pyrazol-1-yl)-5,8-dihydroquinoxaline (3ar): The title compound was prepared according to the general procedure A. The crude residue was purified by column chromatography on silica gel with an eluent of hexanes/ethyl acetate (2:1) to furnish the pure compound as a brown solid in 43% yield.

^{1}H NMR (500 MHz, $CDCl_3$) δ 8.48 (d, *J* = 2.5 Hz, 1H), 8.43 (d, *J* = 2.5 Hz, 1H), 7.63 (d, *J* = 2.4 Hz, 1H), 7.50 (d, *J* = 1.9 Hz, 1H), 6.25 (t, *J* = 2.0 Hz, 1H), 5.98 (t, *J* = 3.6 Hz, 1H), 5.28 (dd, *J* = 4.7, 3.0 Hz, 1H), 4.13 – 3.91 (m, 1H), 3.76 – 3.67 (m, 1H), 3.63 (s, 3H).

^{13}C NMR (126 MHz, $CDCl_3$) δ 150.85, 150.45, 147.21, 144.01, 143.17, 140.50, 130.29, 105.45, 95.32, 62.93, 55.21, 31.00.

HRMS (ESI+): calcd for $C_{14}H_{15}N_3O$ $[M+H]^+$ 242.1288, found 242.1285.

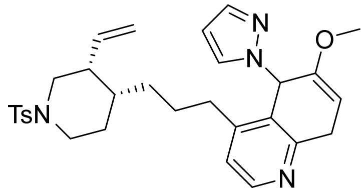

6-methoxy-5-(1*H*-pyrazol-1-yl)-4-(3-((3*R*,4*R*)-1-tosyl-3-vinylpiperidin-4-yl)propyl)-5,8-dihydroquinoline (3as): The title compound was prepared according to the general procedure A. The crude residue was purified by column chromatography on silica gel with an eluent of hexanes/ethyl acetate (2:1) to furnish the pure compound as a brown solid in 79% yield.

^{1}H NMR (500 MHz, $CDCl_3$) δ 8.42 (d, *J* = 5.0 Hz, 1H), 7.76 – 7.50 (m, 2H), 7.36 (d, *J* = 1.8 Hz, 1H), 7.31 (d, *J* = 8.0 Hz, 2H), 7.13 (dd, *J* = 10.3, 2.3 Hz, 1H), 6.95 (d, *J* = 5.0 Hz, 1H), 6.13 (dt, *J* = 4.0, 2.0 Hz, 1H), 6.08 (q, *J* = 2.8 Hz, 1H), 6.01 – 5.69 (m, 1H), 5.42 – 4.91 (m, 3H), 3.90 (dd, *J* = 22.3, 3.0 Hz, 1H), 3.78 – 3.61 (m, 2H), 3.56 (d, *J* = 1.4 Hz, 4H), 2.61 – 2.33 (m, 3H), 2.43 (s, 3H), 2.33 – 2.20 (m, 2H), 1.45 (dtt, *J* = 12.0, 8.8, 3.9 Hz, 2H), 1.36 – 0.80 (m, 6H).
^{13}C NMR (126 MHz, $CDCl_3$) δ 156.15, 151.71 (d, *J* = 6.5 Hz), 151.45 (d, *J* = 9.9 Hz), 149.10, 143.49, 138.84 (d, *J* = 7.0 Hz), 135.54 (d, *J* = 7.1 Hz), 133.23 (d, *J* = 1.6 Hz), 129.69, 127.94 (d, *J* = 13.6 Hz), 127.81, 126.39 (d, *J* = 9.3 Hz), 122.58 (d, *J* = 3.0 Hz), 117.57 (d, *J* = 3.1 Hz), 106.16 (d, *J* = 5.0 Hz), 94.59, 59.15, 55.14, 51.18 (d, *J* = 5.4 Hz), 46.42 (d, *J* = 3.4 Hz), 41.97 (d, *J* = 12.8 Hz), 37.86 (d, *J* = 6.6 Hz), 32.56, 32.37, 31.36 (d, *J* = 25.4 Hz), 27.29 (d, *J* = 14.5 Hz), 26.86 (d, *J* = 35.8 Hz), 21.65.
HRMS (ESI+): calcd for $C_{30}H_{36}N_4O_3S$ $[M+Na]^+$ 555.2400, found 555.2398.

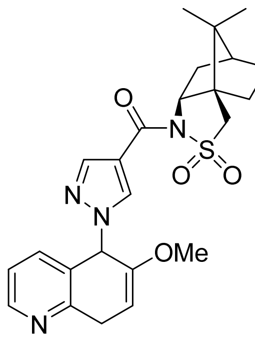

((3a*R*,6*S*,7a*S*)-8,8-dimethyl-2,2-dioxidotetrahydro-3*H*-3a,6-methanobenzo[*c*]isothiazol-1(4*H*)-yl)(1-(6-methoxy-5,8-dihydroquinolin-5-yl)-1*H*-pyrazol-4-yl)methanone (3at): The title compound was prepared according to the general procedure C. The crude residue was purified by column chromatography on silica gel with an eluent of hexanes/ethyl acetate (2:1) to furnish the pure compound as a brown solid in 43% yield.
^{1}H NMR (500 MHz, $CDCl_3$) δ 8.62 – 8.48 (m, 1H), 8.15 (d, *J* = 27.3 Hz, 1H), 8.07 (d, *J* = 21.0 Hz, 1H), 7.60 (ddd, *J* = 11.9, 7.9, 1.7 Hz, 1H), 7.16 (dd, *J* = 7.9, 4.7 Hz, 1H), 5.99 (t, *J* = 3.8 Hz, 1H), 5.40 – 5.26 (m, 1H), 4.12 (td, *J* = 7.2, 4.7 Hz, 1H), 3.89 (ddt, *J* = 22.4, 6.1, 3.6 Hz, 1H), 3.71 (dq, *J* = 22.4, 4.4 Hz, 1H), 3.62 (d, *J* = 3.2 Hz, 3H), 3.52 (dd, *J* = 13.6, 8.8 Hz, 1H), 3.43 (dd, *J* = 13.7, 10.5 Hz, 1H), 2.13 – 1.73 (m, 5H), 1.53 – 1.33 (m, 2H), 1.23 (d, *J* = 2.6 Hz, 3H), 0.99 (d, *J* = 4.3 Hz, 3H).
^{13}C NMR (126 MHz, $CDCl_3$) δ 161.75 (d, *J* = 1.3 Hz), 154.49 (d, *J* = 34.2 Hz), 149.55, 149.17 (d, *J* = 25.7 Hz), 142.17 (d, *J* = 18.7 Hz), 136.86 (d, *J* = 9.1 Hz), 132.94 (d, *J* = 7.7 Hz), 127.64 (d, *J* = 7.4 Hz), 122.14 (d, *J* = 14.0 Hz), 117.12 (d, *J* = 19.5 Hz), 96.88 (d, *J* = 14.9 Hz), 66.20, 61.76 (d, *J* = 17.5 Hz), 55.18, 53.76 (d, *J* = 6.4 Hz), 48.18 (d, *J* = 6.3 Hz), 47.90, 45.16 (d, *J* = 3.0 Hz), 38.62 (d, *J* = 3.5 Hz), 32.59 (d, *J* = 171.2 Hz), 29.42, 26.64, 21.31 (d, *J* = 10.0 Hz), 20.06.
HRMS (ESI+): calcd for $C_{24}H_{28}N_4O_4S$ $[M+H]^+$ 469.1904, found 469.1905.

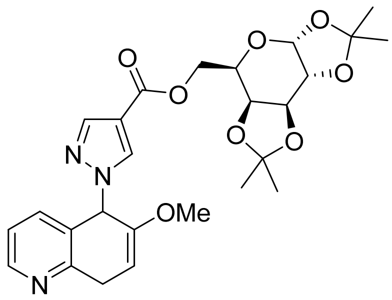

((3a*R*,5*R*,5a*S*,8a*S*,8b*R*)-2,2,7,7-tetramethyltetrahydro-5*H*-bis([1,3]dioxolo)[4,5-b:4',5'-*d*]pyran-5-yl)methyl 1-(6-methoxy-5,8-dihydroquinolin-5-yl)-1*H*-pyrazole-4-carboxylate (3au): The title compound was prepared according to the general procedure A. The crude residue was purified by column chromatography on silica gel with an eluent of hexanes/ethyl acetate (2:1) to furnish the pure compound as a brown solid in 32% yield.

^{1}H NMR (500 MHz, $CDCl_3$) δ 8.53 (dd, *J* = 4.8, 1.6 Hz, 1H), 7.89 (s, 1H), 7.86 (d, *J* = 1.2 Hz, 1H), 7.63 – 7.53 (m, 1H), 7.15 (dd, *J* = 7.9, 4.6 Hz, 1H), 6.00 (s, 1H), 5.52 (d, *J* = 5.0 Hz, 1H), 5.45 – 5.15 (m, 1H), 4.61 (dd, *J* = 7.9, 2.5 Hz, 1H), 4.40 (ddd, *J* = 11.6, 4.9, 1.2 Hz, 1H), 4.34 – 4.29 (m, 2H), 4.28 – 4.22 (m, 1H), 4.10 (dt, *J* = 7.3, 2.6 Hz, 1H), 3.88 (dt, *J* = 22.6, 3.6 Hz, 1H), 3.72 (dt, *J* = 22.6, 4.2 Hz, 1H), 3.61 (s, 3H), 1.47 (s, 3H), 1.44 (s, 3H), 1.32 (s, 3H), 1.30 (s, 3H).

^{13}C NMR (126 MHz, $CDCl_3$) δ 162.82, 154.26, 149.47, 149.17, 141.44 (d, *J* = 2.4 Hz), 136.95, 131.82 (d, *J* = 2.9 Hz), 122.23, 115.35, 109.37 (d, *J* = 106.5 Hz), 96.72 (d, *J* = 3.7 Hz), 96.44, 71.22, 70.82, 70.66, 66.24 (d, *J* = 2.2 Hz), 63.42 (d, *J* = 3.4 Hz), 61.73, 55.17, 51.52, 31.78, 29.84, 26.16, 26.10, 25.13, 24.62.

HRMS (ESI+): calcd for $C_{26}H_{31}N_3O_8$ $[M+H]^+$ 514.2184, found 514.2186.

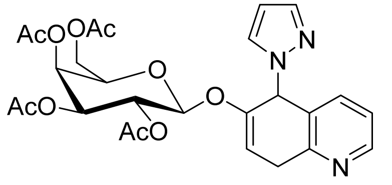

(2*S*,3*R*,4*S*,5*S*,6*R*)-2-((5-(1*H*-pyrazol-1-yl)-5,8-dihydroquinolin-6-yl)oxy)-6-(acetoxymethyl)tetrahydro-2*H*-pyran-3,4,5-triyl triacetate (3av): The title compound was prepared according to the general procedure C. The crude residue was purified by column chromatography on silica gel with an eluent of hexanes/ethyl acetate (2:1) to furnish the pure compound as a brown solid in 35% yield.

^{1}H NMR (500 MHz, $CDCl_3$) δ 8.57 – 8.35 (m, 1H), 7.68 – 7.36 (m, 3H), 7.12 (ddd, *J* = 10.3, 7.9, 4.7 Hz, 1H), 6.23 (d, *J* = 15.7 Hz, 1H), 5.98 (dt, *J* = 25.4, 4.1 Hz, 1H), 5.90 – 5.59 (m, 1H), 5.39 (ddd, *J* = 12.1, 3.5, 1.2 Hz, 1H), 5.26 (ddd, *J* = 26.9, 10.4, 7.9 Hz, 1H), 5.00 (ddd, *J* = 17.0, 10.4, 3.5 Hz, 1H), 4.91 (dd, *J* = 24.6, 8.0 Hz, 1H), 4.32 – 4.06 (m, 2H), 4.04 – 3.84 (m, 2H), 3.71 (ddt, *J* = 23.1, 19.3, 4.3 Hz, 1H), 2.14 (d, *J* = 8.2 Hz, 3H), 2.03 (d, *J* = 4.9 Hz, 1.7H), 1.96 (d, *J* = 5.9 Hz, 3H), 1.72 (s, 1.3H).

^{13}C NMR (126 MHz, $CDCl_3$) δ 170.29 (d, *J* = 1.9 Hz), 170.17 (d, *J* = 10.5 Hz), 170.05 (d, *J* = 9.3 Hz), 169.08 (d, *J* = 13.2 Hz), 153.35 (d, *J* = 74.5 Hz), 149.38 (d, *J* = 10.4 Hz), 147.14 (d, *J* = 14.6 Hz), 139.85 (d, *J* = 69.5 Hz), 136.37 (d, *J* = 48.4 Hz), 128.58 (d, *J* = 126.5 Hz),

128.18 (d, J = 56.5 Hz), 122.04 (d, J = 4.8 Hz), 106.96 (d, J = 146.9 Hz), 104.23 (d, J = 377.2 Hz), 98.62 (d, J = 131.2 Hz), 71.07 (d, J = 4.0 Hz), 70.71 (d, J = 2.5 Hz), 68.02 (d, J = 84.6 Hz), 66.76 (d, J = 10.7 Hz), 61.28 (d, J = 30.9 Hz), 60.22 (d, J = 59.1 Hz), 31.95 (d, J = 21.2 Hz), 20.79, 20.66, 20.63 (d, J = 2.8 Hz), 20.54 (d, J = 4.2 Hz).

HRMS (ESI+): calcd for $C_{26}H_{29}N_3O_{10}$ $[M+H]^+$ 544.1926, found 544.1923.

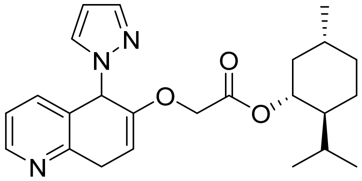

(1*R*,2*S*,5*R*)-2-isopropyl-5-methylcyclohexyl 2-((5-(1*H*-pyrazol-1-yl)-5,8-dihydroquinolin-6-yl)oxy)acetate (3aw): The title compound was prepared according to the general procedure A. The crude residue was purified by column chromatography on silica gel with an eluent of hexanes/ethyl acetate (2:1) to furnish the pure compound as a brown solid in 56% yield.

^{1}H NMR (400 MHz, $CDCl_3$) δ 8.52 (dd, J = 4.7, 1.7 Hz, 1H), 7.60 (dd, J = 7.9, 1.7 Hz, 1H), 7.51 (d, J = 1.8 Hz, 1H), 7.48 (t, J = 2.9 Hz, 1H), 7.15 (dd, J = 7.9, 4.7 Hz, 1H), 6.25 (t, J = 2.1 Hz, 1H), 6.11 (q, J = 4.1 Hz, 1H), 5.18 (td, J = 5.0, 3.2 Hz, 1H), 4.75 (dtd, J = 12.8, 10.9, 4.4 Hz, 1H), 4.42 (d, J = 11.0 Hz, 2H), 3.95 – 3.80 (m, 1H), 3.69 (dt, J = 22.5, 4.2 Hz, 1H), 2.12 – 1.91 (m, 1H), 1.78 (dtd, J = 23.6, 7.0, 2.7 Hz, 1H), 1.72 – 1.57 (m, 2H), 1.47 (dddt, J = 12.0, 9.5, 6.6, 3.2 Hz, 1H), 1.41 – 1.29 (m, 1H), 1.16 – 0.82 (m, 8H), 0.81 – 0.60 (m, 4H).

^{13}C NMR (101 MHz, $CDCl_3$) δ 168.11 (d, J = 17.0 Hz), 153.58 (d, J = 4.1 Hz), 148.98 (d, J = 4.3 Hz), 148.35 (d, J = 1.6 Hz), 139.78, 137.03 (d, J = 4.2 Hz), 128.67 (d, J = 5.3 Hz), 128.53 (d, J = 3.7 Hz), 122.21, 106.20 (d, J = 2.5 Hz), 97.93 (d, J = 4.3 Hz), 75.62 (d, J = 10.3 Hz), 65.01 (d, J = 7.8 Hz), 60.66 (d, J = 6.8 Hz), 46.97 (d, J = 3.9 Hz), 40.84 (d, J = 4.4 Hz), 34.20, 31.60, 31.47, 26.28 (d, J = 7.5 Hz), 23.34 (d, J = 6.0 Hz), 22.05, 20.79 (d, J = 12.3 Hz), 16.19 (d, J = 11.9 Hz).

HRMS (ESI+): calcd for $C_{24}H_{31}N_3O_3$ $[M+H]^+$ 410.2438, found 410.2438.

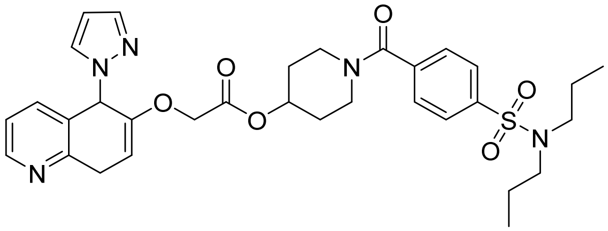

1-(4-(*N*,*N*-dipropylsulfamoyl)benzoyl)piperidin-4-yl 2-((5-(1*H*-pyrazol-1-yl)-5,8-dihydroquinolin-6-yl)oxy)acetate (3ax): The title compound was prepared according to the general procedure A. The crude residue was purified by column chromatography on silica gel with an eluent of hexanes/ethyl acetate (2:1) to furnish the pure compound as a brown solid in 39% yield.

^{1}H NMR (500 MHz, $CDCl_3$) δ 8.52 (dd, J = 4.8, 1.7 Hz, 1H), 8.05 – 7.77 (m, 2H), 7.64 – 7.32 (m, 5H), 7.14 (dd, J = 7.9, 4.7 Hz, 1H), 6.25 (t, J = 2.1 Hz, 1H), 6.11 (t, J = 4.0 Hz, 1H), 5.20 (dd, J = 4.6, 3.2 Hz, 1H), 5.12 (dt, J = 7.5, 3.7 Hz, 1H), 4.53 – 4.33 (m, 2H), 3.87 (dt, J

= 22.5, 3.8 Hz, 2H), 3.76 – 3.58 (m, 2H), 3.46 (s, 1H), 3.25 (s, 1H), 3.16 – 2.87 (m, 4H), 1.98 (s, 1H), 1.80 (s, 2H), 1.67 – 1.45 (m, 5H), 0.87 (t, *J* = 7.4 Hz, 6H).
^{13}C NMR (126 MHz, $CDCl_3$) δ 168.83, 167.58, 153.54, 149.31, 148.34, 141.51, 139.70, 139.48, 136.55, 128.45, 128.20, 127.44, 127.39, 122.11, 106.15, 98.00, 70.07, 64.82, 60.60, 50.14, 41.77 (d, *J* = 674.5 Hz), 31.69, 30.60 (d, *J* = 112.0 Hz), 22.10, 11.18.
HRMS (ESI+): calcd for $C_{32}H_{39}N_5O_6S$ $[M+H]^+$ 622.2694, found 622.2690.

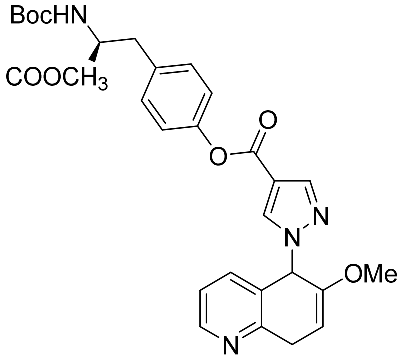

4-((*S*)-2-((tert-butoxycarbonyl)amino)-3-methoxy-3-oxopropyl)phenyl 1-(6-methoxy-5,8-dihydroquinolin-5-yl)-1*H*-pyrazole-4-carboxylate (3ay): The title compound was prepared according to the general procedure C. The crude residue was purified by column chromatography on silica gel with an eluent of hexanes/ethyl acetate (2:1) to furnish the pure compound as a brown solid in 43% yield.
^{1}H NMR (500 MHz, $CDCl_3$) δ 8.57 (s, 1H), 8.02 (d, *J* = 18.8 Hz, 2H), 7.63 (dd, *J* = 7.9, 1.6 Hz, 1H), 7.18 (q, *J* = 4.4 Hz, 1H), 7.14 (d, *J* = 8.2 Hz, 2H), 7.10 – 7.02 (m, 2H), 6.07 (t, *J* = 3.8 Hz, 1H), 5.35 (dd, *J* = 4.6, 3.1 Hz, 1H), 4.99 (d, *J* = 8.4 Hz, 1H), 4.58 (d, *J* = 7.4 Hz, 1H), 3.96 – 3.88 (m, 1H), 3.82 – 3.72 (m, 1H), 3.70 (s, 3H), 3.66 (s, 3H), 3.27 – 2.90 (m, 2H), 1.42 (s, 10H).
^{13}C NMR (126 MHz, $CDCl_3$) δ 172.36, 161.20, 155.22, 154.39, 149.77, 149.62, 149.09, 141.76, 136.70, 133.76, 132.31, 130.42, 122.19, 121.91, 116.33, 114.87, 97.01, 80.16, 61.97, 55.22, 54.51, 52.40, 37.87, 31.94, 28.44.
HRMS (ESI+): calcd for $C_{29}H_{32}N_4O_7$ $[M+H]^+$ 549.2344, found 549.2344.

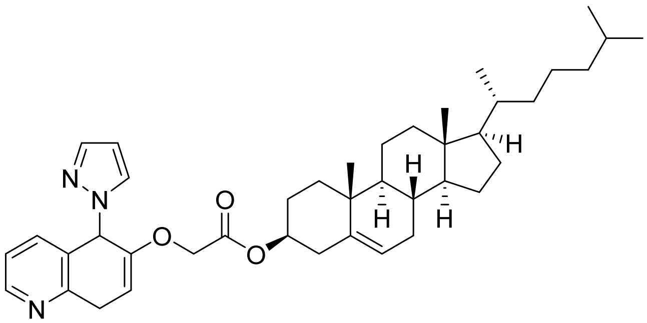

(3*S*,8*S*,9*S*,10*R*,13*R*,14*S*,17*R*)-10,13-dimethyl-17-((*R*)-6-methylheptan-2-yl)-2,3,4,7,8,9,10,11,12,13,14,15,16,17-tetradecahydro-1*H*-cyclopenta[*a*]phenanthren-3-yl 2-((5-(1*H*-pyrazol-1-yl)-5,8-dihydroquinolin-6-yl)oxy)acetate (3az): The title compound was prepared according to the general procedure C. The crude residue was purified by column

chromatography on silica gel with an eluent of hexanes/ethyl acetate (2:1) to furnish the pure compound as a white solid in 31% yield.

^{1}H NMR (500 MHz, $CDCl_3$) δ 8.51 (dd, *J* = 4.7, 1.7 Hz, 1H), 7.58 (dd, *J* = 8.0, 1.7 Hz, 1H), 7.55 – 7.48 (m, 2H), 7.13 (dd, *J* = 7.9, 4.7 Hz, 1H), 6.26 (t, *J* = 2.1 Hz, 1H), 6.12 (t, *J* = 4.0 Hz, 1H), 5.37 (dt, *J* = 5.3, 1.8 Hz, 1H), 5.20 (dd, *J* = 4.7, 3.2 Hz, 1H), 4.84 – 4.61 (m, 1H), 4.55 – 4.28 (m, 2H), 3.87 (dt, *J* = 22.4, 3.7 Hz, 1H), 3.69 (dt, *J* = 22.5, 4.3 Hz, 1H), 2.31 (t, *J* = 7.6 Hz, 2H), 1.99 (ddt, *J* = 23.3, 17.1, 4.4 Hz, 2H), 1.90 – 1.78 (m, 3H), 1.65 – 1.41 (m, 8H), 1.38 – 1.29 (m, 3H), 1.28 – 1.22 (m, 2H), 1.20 – 1.06 (m, 9H), 1.01 (s, 4H), 0.91 (d, *J* = 6.5 Hz, 3H), 0.87 (d, *J* = 2.4 Hz, 3H), 0.85 (d, *J* = 2.3 Hz, 3H), 0.67 (s, 3H).

^{13}C NMR (126 MHz, $CDCl_3$) δ 167.80, 153.73, 149.27, 148.27, 139.66, 139.19 (d, *J* = 2.4 Hz), 136.54, 128.46, 128.34, 123.09 (d, *J* = 2.8 Hz), 121.98, 106.05, 97.99, 75.15, 64.89, 60.66, 56.67, 56.14, 49.99, 42.31, 39.71, 39.52, 37.96 (d, *J* = 4.8 Hz), 36.88, 36.56, 36.18, 35.79, 31.89, 31.84, 31.79, 28.23, 28.02, 27.69 (d, *J* = 6.2 Hz), 24.28, 23.83, 22.83, 22.57, 21.03, 19.29, 18.72, 11.86.

HRMS (ESI+): calcd for $C_{41}H_{57}N_3O_3$ $[M+H]^+$ 640.4473, found 640.4471.

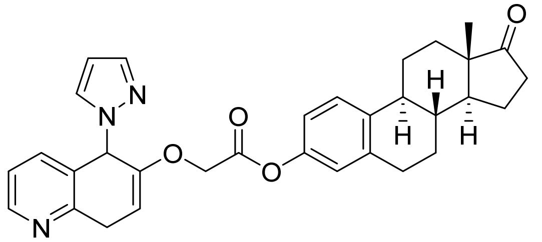

(8*R*,9*S*,13*S*,14*S*)-13-methyl-17-oxo-7,8,9,11,12,13,14,15,16,17-decahydro-6*H*-cyclopenta[*a*]phenanthren-3-yl 2-((5-(1*H*-pyrazol-1-yl)-5,8-dihydroquinolin-6-yl)oxy)acetate (3ba): The title compound was prepared according to the general procedure A. The crude residue was purified by column chromatography on silica gel with an eluent of hexanes/ethyl acetate (2:1) to furnish the pure compound as a brown solid in 47% yield.

^{1}H NMR (500 MHz, $CDCl_3$) δ 8.51 (dd, *J* = 4.7, 1.7 Hz, 1H), 7.59 (ddd, *J* = 8.1, 4.1, 1.7 Hz, 1H), 7.55 – 7.44 (m, 2H), 7.14 (ddd, *J* = 7.9, 4.7, 3.3 Hz, 1H), 6.97 – 6.72 (m, 1H), 6.25 (dt, *J* = 10.5, 2.1 Hz, 1H), 6.14 (dt, *J* = 19.0, 4.0 Hz, 1H), 5.46 – 5.18 (m, 1H), 4.76 – 4.57 (m, 1H), 4.52 – 4.34 (m, 1H), 4.21 (q, *J* = 7.1 Hz, 1H), 4.11 (q, *J* = 7.2 Hz, 0H), 3.89 (ddt, *J* = 22.5, 18.8, 3.7 Hz, 1H), 3.80 – 3.64 (m, 2H), 2.96 – 2.78 (m, 2H), 2.56 – 2.44 (m, 1H), 2.43 – 2.22 (m, 1H), 2.19 – 1.87 (m, 4H), 1.73 – 1.35 (m, 4H), 1.26 (q, *J* = 7.3 Hz, 3H), 0.90 (s, 2H).

^{13}C NMR (126 MHz, $CDCl_3$) δ 167.85 (d, *J* = 147.8 Hz), 153.61 (d, *J* = 19.2 Hz), 149.18 (d, *J* = 9.2 Hz), 148.29 (d, *J* = 3.7 Hz), 147.95, 139.80 (d, *J* = 2.5 Hz), 138.19 (d, *J* = 41.7 Hz), 136.89, 128.64 (d, *J* = 8.3 Hz), 128.58 (d, *J* = 2.8 Hz), 128.52, 126.66, 122.24, 122.19, 121.32, 118.48, 106.25 (d, *J* = 13.1 Hz), 98.83 (d, *J* = 100.7 Hz), 98.14 (d, *J* = 11.0 Hz), 64.75 (d, *J* = 9.7 Hz), 61.40, 60.64 (d, *J* = 4.7 Hz), 60.50, 50.51, 48.03, 44.24, 38.06, 35.94, 31.72, 31.69, 31.63, 29.50, 26.38, 25.85, 21.69, 14.28 (d, *J* = 6.9 Hz), 13.92.

HRMS (ESI+): calcd for $C_{32}H_{33}N_3O_4$ $[M+H]^+$ 524.2544, found 524.2540.

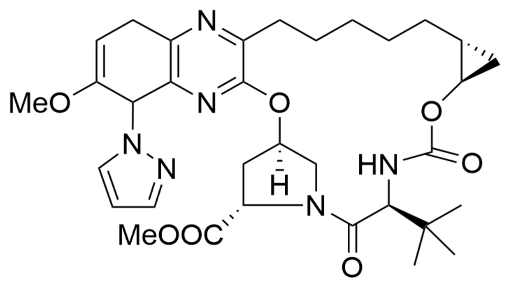

Methyl (1a*R*,5*S*,8*S*,10*R*,22a*R*)-5-(*tert*-butyl)-14-methoxy-3,6-dioxo-13-(1*H*-pyrazol-1-yl)-1,1a,3,4,5,6,9,10,13,16,18,19,20,21,22,22a-hexadecahydro-8*H*-7,10-methanocyclopropa[18,19][1,10]dioxa[3,6]diazacyclononadecino[11,12-b]quinoxaline-8-carboxylate (3bb): The title compound was prepared according to the general procedure A. The crude residue was purified by column chromatography on silica gel with an eluent of hexanes/ethyl acetate (2:1) to furnish the pure compound as a brown solid in 27% yield.

^{1}H NMR (500 MHz, $CDCl_3$) δ 7.51 (d, J = 1.8 Hz, 1H), 7.48 (d, J = 2.3 Hz, 1H), 6.24 (t, J = 2.1 Hz, 1H), 5.84 (t, J = 4.4 Hz, 1H), 5.46 (t, J = 4.0 Hz, 1H), 5.25 (dd, J = 4.6, 3.1 Hz, 1H), 5.18 (d, J = 9.9 Hz, 1H), 4.52 (dd, J = 10.6, 7.3 Hz, 1H), 4.30 (d, J = 10.0 Hz, 1H), 4.25 (dd, J = 11.8, 1.9 Hz, 1H), 3.89 – 3.82 (m, 1H), 3.82 – 3.78 (m, 1H), 3.77 (dt, J = 7.1, 2.9 Hz, 1H), 3.73 (s, 3H), 3.62 (s, 3H), 3.61 – 3.53 (m, 1H), 2.79 – 2.60 (m, 2H), 2.52 (ddd, J = 14.2, 7.6, 1.8 Hz, 1H), 2.22 – 2.09 (m, 1H), 1.81 – 1.56 (m, 4H), 1.46 (qd, J = 7.8, 7.0, 4.0 Hz, 3H), 1.04 (s, 9H), 0.89 (tdt, J = 7.1, 4.7, 2.3 Hz, 2H), 0.69 – 0.56 (m, 1H), 0.44 (dt, J = 7.1, 5.8 Hz, 1H).

^{13}C NMR (126 MHz, $CDCl_3$) δ 172.13, 171.27, 157.24, 154.98, 150.18, 148.46, 140.69, 139.78, 139.10, 129.61, 105.25, 95.57, 74.15, 62.59, 59.16, 57.91, 55.29, 55.08, 54.10, 52.25, 35.32, 34.92, 33.16, 30.60, 29.96, 29.17, 28.38, 28.18, 26.24, 18.77, 10.92.

HRMS (ESI+): calcd for $C_{33}H_{44}N_6O_7$ $[M+H]^+$ 637.3344, found 737.3335.

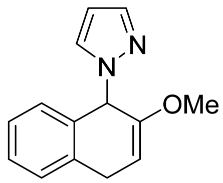

1-(2-methoxy-1,4-dihydronaphthalen-1-yl)-1*H*-pyrazole (3bc): The title compound was prepared according to the general procedure B, but use 2.0 equiv acid. The crude residue was purified by column chromatography on silica gel with an eluent of hexanes/ethyl acetate (5:1) to furnish the pure compound as a brown solid in 60% yield.

^{1}H NMR (400 MHz, $CDCl_3$) δ 7.55 (d, J = 1.8 Hz, 1H), 7.36 (d, J = 2.3 Hz, 1H), 7.32 – 7.24 (m, 3H), 7.24 – 7.14 (m, 1H), 6.27 (t, J = 2.2 Hz, 1H), 6.04 (t, J = 3.8 Hz, 1H), 5.27 (dd, J = 4.7, 3.1 Hz, 1H), 3.82 (dt, J = 21.5, 3.6 Hz, 1H), 3.65 (s, 3H), 3.63 – 3.54 (m, 1H).

^{13}C NMR (101 MHz, $CDCl_3$) δ 151.23, 139.19, 134.30, 133.37, 128.62, 128.15, 127.89, 127.78, 126.72, 105.88, 95.85, 61.12, 54.91, 29.04.

HRMS (ESI+): calcd for $C_{30}H_{36}N_4O_3S$ $[M+H]^+$ 227.1179, found 227.1181.

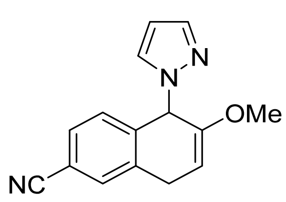

6-methoxy-5-(1*H*-pyrazol-1-yl)-5,8-dihydronaphthalene-2-carbonitrile (3bd): The title compound was prepared according to the general procedure C. The crude residue was purified by column chromatography on silica gel with an eluent of hexanes/ethyl acetate (5:1) to furnish the pure compound as a brown solid in 62% yield.

^{1}H NMR (500 MHz, $CDCl_3$) δ 7.43 (d, *J* = 1.6 Hz, 1H), 7.37 (d, *J* = 1.9 Hz, 1H), 7.30 (dd, *J* = 8.2, 1.7 Hz, 1H), 7.26 (d, *J* = 2.4 Hz, 1H), 7.18 (d, *J* = 8.1 Hz, 1H), 6.12 (t, *J* = 2.1 Hz, 1H), 5.84 (t, *J* = 4.0 Hz, 1H), 5.11 (dd, *J* = 4.7, 3.1 Hz, 1H), 3.66 (dt, *J* = 21.8, 3.6 Hz, 1H), 3.54 – 3.41 (m, 4H).

^{13}C NMR (126 MHz, $CDCl_3$) δ 150.35, 139.81, 138.11, 135.65, 132.13, 129.91, 129.48, 128.41, 118.58, 111.70, 106.18, 95.35, 60.36, 54.99, 28.68.

HRMS (ESI+): calcd for $C_{15}H_{13}N_3O$ $[M+H]^+$ 252.1131, found 252.1133.

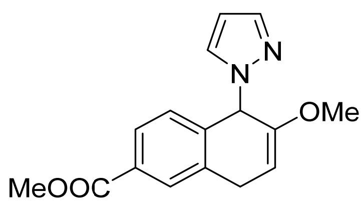

Methyl 6-methoxy-5-(1*H*-pyrazol-1-yl)-5,8-dihydronaphthalene-2-carboxylate (3be): The title compound was prepared according to the general procedure C. The crude residue was purified by column chromatography on silica gel with an eluent of hexanes/ethyl acetate (2:1) to furnish the pure compound as a brown solid in 40% yield.

^{1}H NMR (500 MHz, $CDCl_3$) δ 7.90 – 7.84 (m, 1H), 7.79 – 7.69 (m, 1H), 7.43 (dd, *J* = 1.8, 0.7 Hz, 1H), 7.26 (dd, *J* = 2.4, 0.8 Hz, 1H), 7.23 – 7.13 (m, 1H), 6.15 (t, *J* = 2.1 Hz, 1H), 5.92 (t, *J* = 3.9 Hz, 1H), 5.16 (dd, *J* = 4.7, 3.1 Hz, 1H), 3.81 (s, 3H), 3.73 (dt, *J* = 21.6, 3.4 Hz, 1H), 3.52 (s, 4H).

^{13}C NMR (126 MHz, $CDCl_3$) δ 166.87, 150.76, 139.54, 137.96, 134.53, 129.66, 129.57, 128.76, 128.12, 127.63, 106.07, 95.79, 60.76, 54.97, 52.27, 28.96.

HRMS (ESI+): calcd for $C_{16}H_{17}N_2O_3$ $[M+H]^+$ 285.1234, found 285.1231.

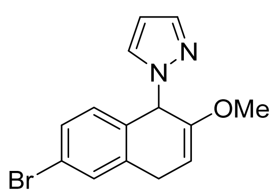

1-(6-bromo-2-methoxy-1,4-dihydronaphthalen-1-yl)-1*H*-pyrazole (3bf): The title compound was prepared according to the general procedure C. The crude residue was purified by column chromatography on silica gel with an eluent of hexanes/ethyl acetate (4:1) to furnish the pure compound as a brown solid in 37% yield.

^{1}H NMR (500 MHz, $CDCl_3$) δ 7.43 (d, *J* = 1.9 Hz, 1H), 7.33 (d, *J* = 2.1 Hz, 1H), 7.26 (d, *J* = 2.4 Hz, 1H), 7.21 (dd, *J* = 8.3, 2.0 Hz, 1H), 7.02 (d, *J* = 8.4 Hz, 1H), 6.16 (t, *J* = 2.1 Hz, 1H), 5.85 (t, *J* = 3.9 Hz, 1H), 5.11 (dd, *J* = 4.7, 3.2 Hz, 1H), 3.67 (dt, *J* = 21.7, 3.7 Hz, 1H), 3.52 (s, 3H), 3.45 (dt, *J* = 21.7, 4.3 Hz, 1H).
^{13}C NMR (126 MHz, $CDCl_3$) δ 150.87, 139.42, 136.49, 132.34, 130.92, 130.23, 129.91, 127.98, 121.82, 106.00, 95.35, 60.49, 54.93, 28.77.
HRMS (ESI+): calcd for $C_{14}H_{14}BrN_2O$ $[M+H]^+$ 305.0284, found 305.0279.

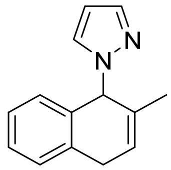

1-(2-methyl-1,4-dihydronaphthalen-1-yl)-1*H*-pyrazole (3bg): The title compound was prepared according to the general procedure D. The crude residue was purified by column chromatography on silica gel with an eluent of hexanes/ethyl acetate (2:1) to furnish the pure compound as a brown solid in 41% yield.
^{1}H NMR (500 MHz, $CDCl_3$) δ 7.56 (d, *J* = 1.8 Hz, 1H), 7.35 – 7.27 (m, 2H), 7.27 – 7.15 (m, 3H), 6.29 (t, *J* = 2.1 Hz, 1H), 6.04 (tt, *J* = 3.0, 1.4 Hz, 1H), 5.94 (t, *J* = 4.6 Hz, 1H), 3.88 – 3.59 (m, 1H), 3.58 – 3.38 (m, 1H), 1.72 (d, *J* = 2.1 Hz, 3H).
^{13}C NMR (126 MHz, $CDCl_3$) δ 138.82, 133.81, 133.63, 131.18, 128.49, 128.28, 127.73, 127.65, 126.80, 124.22, 106.28, 62.79, 30.05, 20.55.
HRMS (ESI+): calcd for $C_{14}H_{14}N_2$ $[M+H]^+$ 211.1230, found 211.1227.

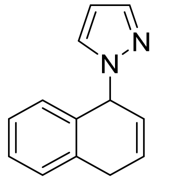

1-(1,4-dihydronaphthalen-1-yl)-1*H*-pyrazole (3bh): The title compound was prepared according to the general procedure C, but use 2.0 equiv naphthalene. The crude residue was purified by column chromatography on silica gel with an eluent of hexanes/ethyl acetate (2:1) to furnish the pure compound as a brown solid in 31% yield.
^{1}H NMR (500 MHz, $CDCl_3$) δ 7.57 (d, *J* = 1.8 Hz, 1H), 7.32 (d, *J* = 2.4 Hz, 1H), 7.30 – 7.23 (m, 2H), 7.23 – 7.12 (m, 2H), 6.53 – 6.24 (m, 2H), 6.22 – 5.96 (m, 2H), 3.79 – 3.58 (m, 1H), 3.56 – 3.39 (m, 1H).
^{13}C NMR (126 MHz, $CDCl_3$) δ 139.06, 133.60, 133.44, 128.55, 128.48, 128.22, 127.84, 127.82, 126.93, 125.18, 106.25, 58.63, 29.61.
HRMS (ESI+): calcd for $C_{30}H_{36}N_4O_3S$ $[M+H]^+$ 197.1073, found 197.1069.

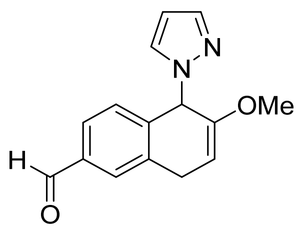

6-methoxy-5-(1*H*-pyrazol-1-yl)-5,8-dihydronaphthalene-2-carbaldehyde (3bi): The title compound was prepared according to the general procedure D. The crude residue was purified by column chromatography on silica gel with an eluent of hexanes/ethyl acetate (5:1) to furnish the pure compound as a brown solid in 57% yield.

^{1}H NMR (500 MHz, $CDCl_3$) δ 9.97 (d, *J* = 1.1 Hz, 1H), 7.77 (s, 1H), 7.67 (d, *J* = 8.0 Hz, 1H), 7.51 (d, *J* = 1.8 Hz, 1H), 7.45 – 7.34 (m, 2H), 6.24 (q, *J* = 1.7 Hz, 1H), 6.00 (t, *J* = 4.0 Hz, 1H), 5.26 (t, *J* = 3.9 Hz, 1H), 3.84 (dt, *J* = 21.8, 3.7 Hz, 1H), 3.65 (dt, *J* = 21.6, 4.2 Hz, 1H), 3.61 (s, 3H).

^{13}C NMR (126 MHz, $CDCl_3$) δ 192.06, 150.65, 139.74, 139.54, 135.75, 135.33, 129.85, 129.48, 128.31, 127.63, 106.14, 95.74, 60.80, 55.03, 28.95.

HRMS (ESI+): calcd for $C_{15}H_{15}N_2O_2$ $[M+H]^+$ 255.1128, found 255.1126.

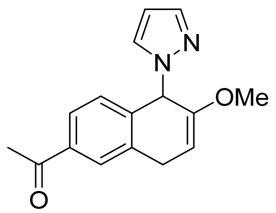

1-(6-methoxy-5-(1H-pyrazol-1-yl)-5,8-dihydronaphthalen-2-yl)ethan-1-one (3bj): The title compound was prepared according to the general procedure B, but used 1.5 equiv of pyrazole. The crude residue was purified by column chromatography on silica gel with an eluent of hexanes/ethyl acetate (2:1) to furnish the pure compound as a colorless oil in 51% yield.

^{1}H NMR (500 MHz, $CDCl_3$) δ 7.92 – 7.80 (m, 1H), 7.73 (dd, *J* = 8.1, 1.8 Hz, 1H), 7.51 (d, *J* = 1.8 Hz, 1H), 7.35 (d, *J* = 2.5 Hz, 1H), 7.30 (d, *J* = 8.1 Hz, 1H), 6.24 (t, *J* = 2.1 Hz, 1H), 5.99 (t, *J* = 3.9 Hz, 1H), 5.25 (dd, *J* = 4.7, 3.1 Hz, 1H), 3.82 (dt, *J* = 21.7, 3.6 Hz, 1H), 3.61 (s, 4H), 2.57 (s, 3H).

^{13}C NMR (126 MHz, $CDCl_3$) δ 197.95, 150.74, 139.64, 138.16, 136.48, 134.77, 128.96, 128.29, 128.22, 126.57, 106.10, 95.87, 60.75, 55.01, 29.08, 26.80.

HRMS (ESI+): calcd for $C_{16}H_{17}N_2O_2$ $[M+H]^+$ 269.1285, found 269.1281.

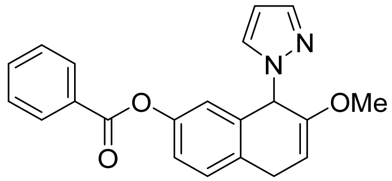

7-methoxy-8-(1*H*-pyrazol-1-yl)-5,8-dihydronaphthalen-2-yl benzoate (3bk): The title compound was prepared according to the general procedure B, but used 1.5 equiv of pyrazole.

The crude residue was purified by column chromatography on silica gel with an eluent of hexanes/ethyl acetate (5:1) to furnish the pure compound as a brown solid in 60% yield.
^{1}H NMR (500 MHz, $CDCl_3$) δ 8.44 – 8.27 (m, 2H), 7.80 (td, *J* = 7.5, 1.4 Hz, 1H), 7.70 (d, *J* = 1.9 Hz, 1H), 7.67 (t, *J* = 7.6 Hz, 2H), 7.55 (d, *J* = 2.4 Hz, 1H), 7.50 (d, *J* = 8.4 Hz, 1H), 7.32 (dd, *J* = 8.5, 2.4 Hz, 1H), 7.26 (d, *J* = 2.4 Hz, 1H), 6.43 (t, *J* = 1.9 Hz, 1H), 6.19 (t, *J* = 4.0 Hz, 1H), 5.42 (t, *J* = 3.9 Hz, 1H), 3.97 (dt, *J* = 21.7, 3.7 Hz, 1H), 3.79 (s, 4H).
^{13}C NMR (126 MHz, $CDCl_3$) δ 165.26, 150.80, 149.51, 139.50, 134.67, 133.72, 131.96, 130.28, 129.53, 129.33, 128.66, 128.13, 121.70, 121.29, 106.06, 95.84, 60.93, 54.99, 28.66.
HRMS (ESI+): calcd for $C_{21}H_{19}N_2O_3$ $[M+H]^+$ 347.1390, found 347.1388.

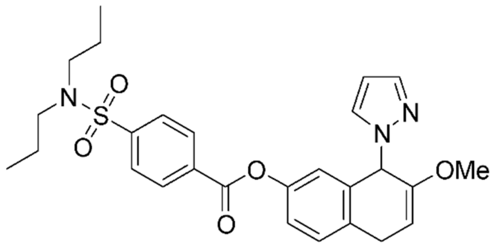

7-methoxy-8-(1*H*-pyrazol-1-yl)-5,8-dihydronaphthalen-2-yl 4-(*N*,*N*-dipropylsulfamoyl)benzoate (3bl): The title compound was prepared according to the general procedure B, but use 1.5 equiv of pyrazole. The crude residue was purified by column chromatography on silica gel with an eluent of hexanes/ethyl acetate (3:1) to furnish the pure compound as a brown solid in 64% yield.
^{1}H NMR (500 MHz, $CDCl_3$) δ 8.15 (d, *J* = 8.5 Hz, 2H), 7.96 – 7.64 (m, 2H), 7.41 (d, *J* = 1.8 Hz, 1H), 7.26 (d, *J* = 2.3 Hz, 1H), 7.22 (d, *J* = 8.5 Hz, 1H), 7.03 (dd, *J* = 8.4, 2.5 Hz, 1H), 6.98 (d, *J* = 2.5 Hz, 1H), 6.14 (t, *J* = 2.1 Hz, 1H), 5.90 (t, *J* = 3.9 Hz, 1H), 5.14 (dd, *J* = 4.7, 3.1 Hz, 1H), 3.68 (dt, *J* = 21.7, 3.7 Hz, 1H), 3.56 – 3.40 (m, 4H), 3.18 – 2.73 (m, 4H), 1.45 (q, *J* = 7.5 Hz, 4H), 0.77 (t, *J* = 7.4 Hz, 6H).
^{13}C NMR (126 MHz, $CDCl_3$) δ 163.87, 150.61, 149.12, 144.98, 139.44, 134.78, 132.77, 132.29, 130.85, 129.41, 128.04, 127.19, 121.37, 121.06, 106.07, 95.82, 60.81, 54.95, 49.95, 28.59, 21.96, 11.24.
HRMS (ESI+): calcd for $C_{27}H_{32}N_3O_5S$ $[M+H]^+$ 510.2057, found 510.2056.

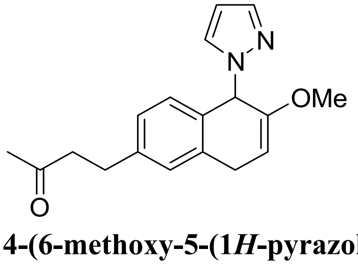

4-(6-methoxy-5-(1*H*-pyrazol-1-yl)-5,8-dihydronaphthalen-2-yl)butan-2-one (3bm): The title compound was prepared according to the general procedure C. The crude residue was purified by column chromatography on silica gel with an eluent of hexanes/ethyl acetate (2:1) to furnish the pure compound as a brown solid in 31% yield.
^{1}H NMR (500 MHz, $CDCl_3$) δ 7.64 (d, *J* = 1.8 Hz, 1H), 7.45 (d, *J* = 2.4 Hz, 1H), 7.27 (d, *J* = 7.9 Hz, 1H), 7.20 (d, *J* = 1.9 Hz, 1H), 7.12 (dd, *J* = 8.0, 1.9 Hz, 1H), 6.35 (t, *J* = 2.1 Hz, 1H), 6.08 (t, *J* = 3.8 Hz, 1H), 5.34 (dd, *J* = 4.7, 3.1 Hz, 1H), 3.86 (dt, *J* = 21.6, 3.5 Hz, 1H), 3.73 (s,

3H), 3.66 (dt, J = 21.5, 4.2 Hz, 1H), 2.99 (t, J = 7.6 Hz, 2H), 2.88 (d, J = 7.8 Hz, 2H), 2.27 (d, J = 2.3 Hz, 3H).
^{13}C NMR (126 MHz, $CDCl_3$) δ 207.86, 151.25, 140.58, 139.19, 134.41, 131.27, 128.74, 127.96, 127.93, 126.85, 105.81, 95.84, 60.90, 54.90, 45.05, 30.16, 29.36, 29.01.
HRMS (ESI+): calcd for $C_{18}H_{21}N_2O_2$ $[M+H]^+$ 297.1593, found 297.1595.

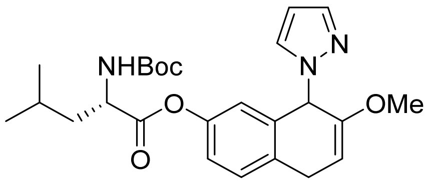

7-methoxy-8-(1*H*-pyrazol-1-yl)-5,8-dihydronaphthalen-2-yl (*tert*-butoxycarbonyl)-L-leucinate (3bn): The title compound was prepared according to the general procedure B, but use 1.5 equiv of pyrazole. The crude residue was purified by column chromatography on silica gel with an eluent of hexanes/ethyl acetate (3:1) to furnish the pure compound as a brown solid in 38% yield.
^{1}H NMR (500 MHz, $CDCl_3$) δ 7.48 (d, J = 1.8 Hz, 1H), 7.30 (dd, J = 5.4, 2.4 Hz, 1H), 7.27 – 7.17 (m, 1H), 6.98 (ddd, J = 11.1, 8.3, 2.4 Hz, 1H), 6.91 (dd, J = 4.2, 2.5 Hz, 1H), 6.21 (t, J = 2.1 Hz, 1H), 5.93 (t, J = 3.8 Hz, 1H), 5.18 (dd, J = 4.7, 3.1 Hz, 1H), 4.91 (d, J = 8.7 Hz, 1H), 4.44 (q, J = 5.6, 4.9 Hz, 1H), 3.72 (dt, J = 21.6, 3.7 Hz, 1H), 3.56 (s, 3H), 3.52 (dt, J = 21.7, 4.3 Hz, 1H), 1.73 (dtd, J = 16.3, 8.2, 2.7 Hz, 2H), 1.62 – 1.51 (m, 1H), 1.41 (d, J = 3.1 Hz, 10H), 0.95 (d, J = 6.2 Hz, 6H).
^{13}C NMR (126 MHz, $CDCl_3$) δ 172.19, 155.54, 150.75 (d, J = 5.0 Hz), 149.07, 139.43 (d, J = 5.6 Hz), 134.60, 132.11, 129.29, 128.04 (d, J = 10.9 Hz), 121.33 (d, J = 11.7 Hz), 120.91 (d, J = 11.5 Hz), 106.06 (d, J = 2.8 Hz), 95.75, 80.13, 60.83 (d, J = 4.5 Hz), 54.95, 52.38, 41.64, 28.59, 28.41, 24.97, 23.04 (d, J = 5.9 Hz), 21.89 (d, J = 8.8 Hz).
HRMS (ESI+): calcd for $C_{25}H_{33}N_3O_5$ $[M+H]^+$ 456.2493, found 456.2490.

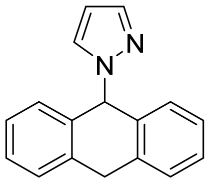

1-(9,10-dihydroanthracen-9-yl)-1*H*-pyrazole (3bo): The title compound was prepared according to the general procedure B. The crude residue was purified by column chromatography on silica gel with an eluent of hexanes/ethyl acetate (2:1) to furnish the pure compound as a brown solid in 87% yield.
^{1}H NMR (500 MHz, $CDCl_3$) δ 7.58 (d, J = 1.8 Hz, 1H), 7.39 (d, J = 7.5 Hz, 2H), 7.36 – 7.24 (m, 6H), 7.13 (d, J = 2.4 Hz, 1H), 6.58 (d, J = 1.8 Hz, 1H), 6.24 (t, J = 2.1 Hz, 1H), 4.23 (d, J = 19.1 Hz, 1H), 4.05 (dd, J = 19.1, 1.9 Hz, 1H).
^{13}C NMR (126 MHz, $CDCl_3$) δ 139.48, 136.19, 134.82, 128.42, 128.24, 128.20, 128.09, 126.98, 106.00, 63.66, 34.84.
HRMS (ESI+): calcd for $C_{17}H_{14}N_2$ $[M+H]^+$ 247.1230, found 247.1228.

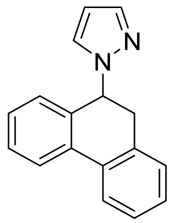

1-(9,10-dihydrophenanthren-9-yl)-1*H*-pyrazole (3bp): The title compound was prepared according to the general procedure B. The crude residue was purified by column chromatography on silica gel with an eluent of hexanes/ethyl acetate (2:1) to furnish the pure compound as a brown solid in 73% yield.

^{1}H NMR (500 MHz, $CDCl_3$) δ 8.06 (ddd, *J* = 25.3, 7.9, 1.2 Hz, 2H), 7.77 (d, *J* = 1.9 Hz, 1H), 7.67 (td, *J* = 7.6, 1.4 Hz, 1H), 7.57 (td, *J* = 7.4, 1.8 Hz, 1H), 7.51 (td, *J* = 7.6, 1.3 Hz, 1H), 7.49 – 7.39 (m, 2H), 7.26 (d, *J* = 2.4 Hz, 1H), 7.22 (dd, *J* = 7.6, 1.3 Hz, 1H), 6.35 (t, *J* = 2.1 Hz, 1H), 5.90 (dd, *J* = 7.4, 5.0 Hz, 1H), 3.88 (dd, *J* = 15.5, 7.4 Hz, 1H), 3.54 (dd, *J* = 15.4, 5.0 Hz, 1H).

^{13}C NMR (126 MHz, $CDCl_3$) δ 139.40, 134.61, 134.24, 133.40, 133.34, 129.32, 129.17, 128.65, 128.35, 128.33, 128.04, 127.78, 124.22, 123.84, 105.38, 59.93, 35.68.

HRMS (ESI+): calcd for $C_{17}H_{14}N_2$ $[M+H]^+$ 247.1230, found 247.1227.

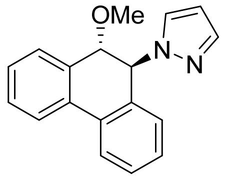

1-((9*S*,10*S*)-10-methoxy-9,10-dihydrophenanthren-9-yl)-1*H*-pyrazole (3bq): The title compound was prepared according to the general procedure B. The crude residue was purified by column chromatography on silica gel with an eluent of hexanes/ethyl acetate (2:1) to furnish the pure compound as a brown solid in 84% yield.

^{1}H NMR (500 MHz, $CDCl_3$) δ 7.86 (ddd, *J* = 7.9, 4.9, 1.2 Hz, 2H), 7.58 (d, *J* = 1.8 Hz, 1H), 7.55 (d, *J* = 2.4 Hz, 1H), 7.49 (td, *J* = 7.6, 1.5 Hz, 1H), 7.46 – 7.39 (m, 2H), 7.35 (td, *J* = 7.4, 1.2 Hz, 1H), 7.26 (td, *J* = 7.6, 1.2 Hz, 1H), 6.83 (d, *J* = 7.6 Hz, 1H), 6.28 (t, *J* = 2.1 Hz, 1H), 5.98 (d, *J* = 3.7 Hz, 1H), 4.53 (d, *J* = 3.6 Hz, 1H), 3.38 (s, 3H).

^{13}C NMR (126 MHz, $CDCl_3$) δ 138.71, 133.65, 133.20, 133.09, 133.03, 129.86, 129.63, 128.90, 128.62, 128.54, 127.97, 127.24, 124.62, 123.92, 106.22, 79.59, 63.81, 56.75.

HRMS (ESI+): calcd for $C_{18}H_{16}N_2O$ $[M+H]^+$ 277.1335, found 277.1334.

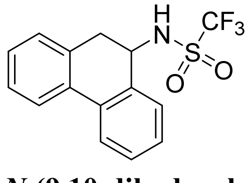

***N*-(9,10-dihydrophenanthren-9-yl)-1,1,1-trifluoromethanesulfonamide (3br)**: The title compound was prepared according to the general procedure B. The crude residue was purified by column chromatography on silica gel with an eluent of hexanes/ethyl acetate (2:1) to furnish the pure compound as a brown solid in 63% yield.

^{1}H NMR (400 MHz, $CDCl_3$) δ 7.99 – 7.75 (m, 2H), 7.59 – 7.28 (m, 6H), 5.00 (d, *J* = 8.8 Hz, 1H), 4.91 (dt, *J* = 8.8, 4.3 Hz, 1H), 3.22 (qd, *J* = 15.7, 4.2 Hz, 2H).
^{13}C NMR (101 MHz, $CDCl_3$) δ 133.62, 133.50, 133.05, 131.40, 129.99, 129.98, 128.76, 128.71, 128.42, 128.16, 124.59, 124.22, 119.69 (q, *J* = 320.8 Hz), 54.01, 36.67.
HRMS (ESI+): calcd for $C_{15}H_{12}F_3NO_2S$ $[M+H]^+$ 328.0614, found 328.0613.

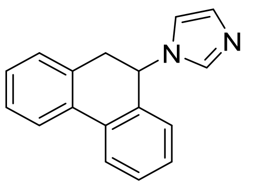

1-(9,10-dihydrophenanthren-9-yl)-1*H*-imidazole (3bs): The title compound was prepared according to the general procedure B. The crude residue was purified by column chromatography on silica gel with an eluent of hexanes/ethyl acetate (2:1) to furnish the pure compound as a yellow solid in 31% yield.
^{1}H NMR (500 MHz, $CDCl_3$) δ 8.09 (dd, *J* = 7.8, 1.2 Hz, 1H), 8.04 (dd, *J* = 7.8, 1.1 Hz, 1H), 7.77 (d, *J* = 1.9 Hz, 1H), 7.66 (td, *J* = 7.6, 1.4 Hz, 1H), 7.57 (td, *J* = 7.4, 1.8 Hz, 1H), 7.51 (td, *J* = 7.5, 1.3 Hz, 1H), 7.49 – 7.40 (m, 2H), 7.26 (d, *J* = 2.3 Hz, 1H), 7.22 (dd, *J* = 7.6, 1.3 Hz, 1H), 6.35 (t, *J* = 2.1 Hz, 1H), 5.89 (dd, *J* = 7.4, 5.0 Hz, 1H), 3.88 (dd, *J* = 15.4, 7.4 Hz, 1H), 3.54 (dd, *J* = 15.4, 5.0 Hz, 1H).
^{13}C NMR (126 MHz, $CDCl_3$) δ 139.40, 134.61, 134.22, 133.39, 133.33, 129.30, 129.15, 128.63, 128.33, 128.32, 128.01, 127.77, 124.21, 123.83, 105.36, 59.92, 35.67.
HRMS (ESI+): calcd for $C_{17}H_{14}N_2$ $[M+H]^+$ 247.1230, found 247.1229.

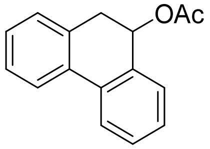

9,10-dihydrophenanthren-9-yl acetate (3bt): The title compound was prepared according to the general procedure B, but use 10 equiv acid. The crude residue was purified by column chromatography on silica gel with an eluent of hexanes/ethyl acetate (2:1) to furnish the pure compound as a brown solid in 89% yield.
^{1}H NMR (400 MHz, $CDCl_3$) δ 8.08 – 7.76 (m, 2H), 7.68 – 7.41 (m, 2H), 7.39 – 7.15 (m, 4H), 6.07 (t, *J* = 4.9 Hz, 1H), 3.18 (d, *J* = 4.9 Hz, 2H), 1.99 (s, 3H).
^{13}C NMR (101 MHz, $CDCl_3$) δ 170.89, 134.28, 133.60, 133.31, 133.03, 129.48, 129.26, 128.66, 128.09, 127.90, 127.53, 124.07, 123.73, 70.01, 34.56, 21.47.
HRMS (ESI+): calcd for $C_{14}H_{16}O_2$ $[M+H]^+$ 239.1067, found 239.1066.

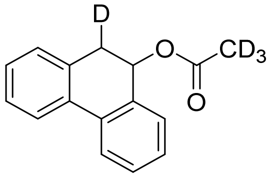

9,10-dihydrophenanthren-9-yl-10-d acetate-d3 (3bu): The title compound was prepared according to the general procedure B, but use 10 equiv acid. The crude residue was purified by column chromatography on silica gel with an eluent of hexanes/ethyl acetate (2:1) to furnish the pure compound as a brown solid in 73% yield.

^{1}H NMR (400 MHz, $CDCl_3$) δ 7.84 (dd, *J* = 7.8, 5.0 Hz, 2H), 7.54 – 7.42 (m, 2H), 7.41 – 7.15 (m, 4H), 6.08 (dd, *J* = 5.3, 4.0 Hz, 1H), 3.17 (dd, *J* = 12.3, 5.3 Hz, 1.4H).

^{13}C NMR (101 MHz, $CDCl_3$) δ 170.92, 134.27, 133.62 (d, *J* = 3.9 Hz), 133.33 (d, *J* = 2.9 Hz), 133.01 (d, *J* = 4.4 Hz), 129.47 (d, *J* = 2.5 Hz), 129.27, 128.73, 128.62 (d, *J* = 8.8 Hz), 127.99 (d, *J* = 18.6 Hz), 127.54 (d, *J* = 1.8 Hz), 124.07, 123.73, 69.96 (d, *J* = 1.9 Hz), 35.02 – 33.65 (m), 21.52 – 20.12 (m).

HRMS (ESI+): calcd for $C_{14}H_{12}D_4O_2$ $[M+H]^+$ 239.1067, found 239.1066.

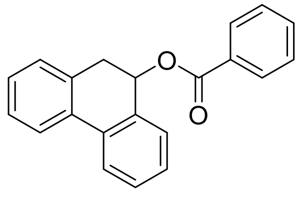

9,10-dihydrophenanthren-9-yl benzoate (3bv): The title compound was prepared according to the general procedure B, but use 5 equiv acid. The crude residue was purified by column chromatography on silica gel with an eluent of hexanes/ethyl acetate (2:1) to furnish the pure compound as a colorless oil in 39% yield.

^{1}H NMR (400 MHz, $CDCl_3$) δ 7.97 – 7.89 (m, 2H), 7.88 – 7.79 (m, 2H), 7.57 (dd, *J* = 7.6, 1.4 Hz, 1H), 7.53 – 7.42 (m, 2H), 7.40 – 7.21 (m, 6H), 6.32 (t, *J* = 5.3 Hz, 1H), 3.30 (d, *J* = 5.3 Hz, 2H).

^{13}C NMR (101 MHz, $CDCl_3$) δ 166.09, 134.25, 133.75, 133.32, 132.93, 130.34, 129.69, 129.29, 129.13, 128.28, 128.23, 127.99, 127.79, 127.43, 126.56, 123.99, 123.64, 70.58, 34.66.

HRMS (ESI+): calcd for $C_{21}H_{16}O_2$ $[M+H]^+$ 301.1223, found 301.1222.

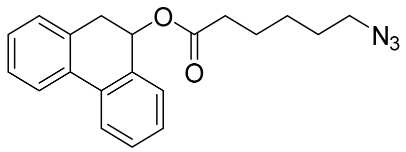

9,10-dihydrophenanthren-9-yl 6-azidohexanoate (3bw): The title compound was prepared according to the general procedure B, but use 5 equiv acid. The crude residue was purified by column chromatography on silica gel with an eluent of hexanes/ethyl acetate (2:1) to furnish the pure compound as a yellow oil in 67% yield.

^{1}H NMR (500 MHz, $CDCl_3$) δ 7.83 (t, *J* = 7.5 Hz, 2H), 7.56 – 7.41 (m, 2H), 7.39 – 7.20 (m, 4H), 6.06 (t, *J* = 4.8 Hz, 1H), 3.53 – 3.02 (m, 4H), 2.23 (t, *J* = 7.3 Hz, 2H), 1.91 – 1.40 (m, 4H), 1.36 – 1.03 (m, 2H).

^{13}C NMR (126 MHz, $CDCl_3$) δ 173.11, 134.18, 133.47, 133.21, 132.91, 129.40, 129.11, 128.57, 127.97, 127.78, 127.42, 123.97, 123.61, 69.88, 51.17, 34.46, 34.26, 28.46, 26.04, 24.44.

HRMS (ESI+): calcd for $C_{20}H_{21}N_3O_2$ $[M+H]^+$ 336.1707, found 336.1706.

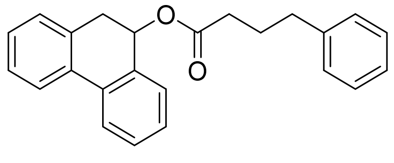

9,10-dihydrophenanthren-9-yl 4-phenylbutanoate (3bx): The title compound was prepared according to the general procedure B, but use 5 equiv acid. The crude residue was purified by column chromatography on silica gel with an eluent of hexanes/ethyl acetate (2:1) to furnish the pure compound as a brown solid in 43% yield.

^{1}H NMR (500 MHz, $CDCl_3$) δ 7.84 (t, *J* = 6.9 Hz, 2H), 7.63 – 7.40 (m, 2H), 7.39 – 7.19 (m, 6H), 7.15 (t, *J* = 7.3 Hz, 1H), 7.09 – 6.87 (m, 2H), 6.08 (t, *J* = 4.8 Hz, 1H), 3.17 (d, *J* = 4.9 Hz, 2H), 2.52 (t, *J* = 7.6 Hz, 2H), 2.23 (t, *J* = 7.4 Hz, 2H), 1.85 (p, *J* = 7.4 Hz, 2H).

^{13}C NMR (126 MHz, $CDCl_3$) δ 173.18, 141.35, 134.18, 133.51, 133.22, 132.92, 129.38, 129.14, 128.56, 128.50, 128.32, 127.99, 127.78, 127.41, 125.90, 123.96, 123.63, 69.83, 34.92, 34.49, 33.77, 26.59.

HRMS (ESI+): calcd for $C_{24}H_{22}O_2$ $[M+H]^+$ 343.1693, found 343.1694.

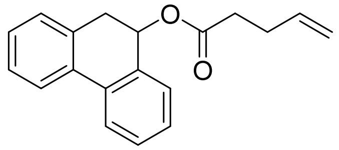

9,10-dihydrophenanthren-9-yl pent-4-enoate (3by): The title compound was prepared according to the general procedure B, but use 5 equiv acid. The crude residue was purified by column chromatography on silica gel with an eluent of hexanes/ethyl acetate (2:1) to furnish the pure compound as a yellow oil in 76% yield.

^{1}H NMR (500 MHz, $CDCl_3$) δ 7.83 (t, *J* = 7.2 Hz, 2H), 7.55 – 7.42 (m, 2H), 7.40 – 7.28 (m, 2H), 7.28 – 7.20 (m, 2H), 6.08 (t, *J* = 4.8 Hz, 1H), 5.86 – 5.57 (m, 1H), 5.08 – 4.70 (m, 2H), 3.17 (d, *J* = 4.7 Hz, 2H), 2.47 – 2.12 (m, 5H).

^{13}C NMR (126 MHz, $CDCl_3$) δ 172.83, 136.63, 134.28, 133.63, 133.31, 133.01, 129.44, 129.24, 128.56, 128.05, 127.87, 127.51, 124.05, 123.71, 115.58, 70.02, 34.58, 33.80, 28.95.

HRMS (ESI+): calcd for $C_{19}H_{18}O_2$ $[M+H]^+$ 279.1380, found 279.1380.

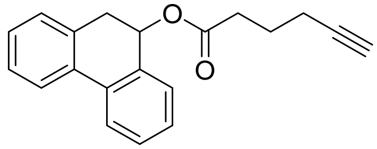

9,10-dihydrophenanthren-9-yl hex-5-ynoate (3bz): The title compound was prepared according to the general procedure B, but use 5 equiv acid. The crude residue was purified by column chromatography on silica gel with an eluent of hexanes/ethyl acetate (2:1) to furnish the pure compound as a yellow oil in 67% yield.

^{1}H NMR (500 MHz, $CDCl_3$) δ 7.83 (t, *J* = 7.5 Hz, 2H), 7.61 – 7.40 (m, 2H), 7.39 – 7.29 (m, 2H), 7.27 (q, *J* = 6.6, 6.0 Hz, 2H), 6.07 (td, *J* = 4.9, 1.7 Hz, 1H), 3.16 (dd, *J* = 5.0, 2.3 Hz, 2H), 2.37 (td, *J* = 7.4, 1.9 Hz, 2H), 2.16 (tt, *J* = 7.0, 2.4 Hz, 2H), 1.90 (q, *J* = 2.5 Hz, 1H), 1.76 (pd, *J* = 7.2, 2.0 Hz, 2H).

^{13}C NMR (126 MHz, $CDCl_3$) δ 172.85, 134.28, 133.60, 133.31, 132.97, 129.47, 129.22, 128.54, 128.09, 127.88, 127.53, 124.08, 123.73, 83.35, 70.09, 69.21, 34.59, 33.26, 23.67, 17.85.
HRMS (ESI+): calcd for $C_{20}H_{18}O_2$ $[M+H]^+$ 291.1380, found 291.1377.

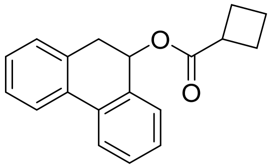

9,10-dihydrophenanthren-9-yl cyclobutanecarboxylate (3ca): The title compound was prepared according to the general procedure B, but use 5 equiv acid. The crude residue was purified by column chromatography on silica gel with an eluent of hexanes/ethyl acetate (2:1) to furnish the pure compound as a yellow oil in 25% yield.
^{1}H NMR (500 MHz, $CDCl_3$) δ 8.04 – 7.68 (m, 2H), 7.55 – 7.40 (m, 2H), 7.35 (dtd, *J* = 14.8, 7.1, 1.7 Hz, 2H), 7.31 – 7.21 (m, 2H), 6.07 (t, *J* = 5.3 Hz, 1H), 3.17 (t, *J* = 5.4 Hz, 2H), 3.12 – 3.00 (m, 1H), 2.23 (dp, *J* = 11.5, 8.7 Hz, 2H), 2.17 – 2.03 (m, 2H), 2.03 – 1.68 (m, 2H).
^{13}C NMR (126 MHz, $CDCl_3$) δ 175.35, 134.26, 134.04, 133.43, 133.13, 129.28, 129.20, 128.08, 128.04, 127.89, 127.51, 124.07, 123.73, 69.85, 38.36, 34.64, 25.40, 25.26, 18.51.
HRMS (ESI+): calcd for $C_{19}H_{18}O_2$ $[M+H]^+$ 279.1380, found 279.1380.

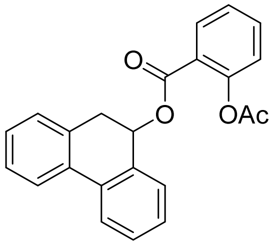

9,10-dihydrophenanthren-9-yl 2-acetoxybenzoate (3cb): The title compound was prepared according to the general procedure B, but use 3 equiv acid. The crude residue was purified by column chromatography on silica gel with an eluent of hexanes/ethyl acetate (2:1) to furnish the pure compound as a brown solid in 37% yield.
^{1}H NMR (400 MHz, $CDCl_3$) δ 7.86 (ddd, *J* = 7.7, 4.5, 2.7 Hz, 3H), 7.59 – 7.42 (m, 3H), 7.40 – 7.30 (m, 2H), 7.30 – 7.27 (m, 2H), 7.21 (td, *J* = 7.6, 1.2 Hz, 1H), 7.02 (dd, *J* = 8.0, 1.2 Hz, 1H), 6.30 (t, *J* = 5.1 Hz, 1H), 3.48 – 3.09 (m, 2H), 2.06 (s, 3H).
^{13}C NMR (101 MHz, $CDCl_3$) δ 169.72, 164.31, 150.72, 134.41, 133.94, 133.37, 133.29, 132.91, 132.19, 129.60, 129.41, 128.68, 128.22, 128.05, 127.62, 126.08, 124.05, 123.88, 123.73, 123.57, 70.91, 34.57, 20.76.
HRMS (ESI+): calcd for $C_{23}H_{18}O_4$ $[M+H]^+$ 359.1278, found 359.1276.

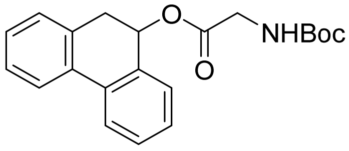

9,10-dihydrophenanthren-9-yl (tert-butoxycarbonyl)glycinate (3cc): The title compound was prepared according to the general procedure B, , but use 5 equiv acid. The crude residue was purified by column chromatography on silica gel with an eluent of hexanes/ethyl acetate (2:1) to furnish the pure compound as a yellow oil in 81% yield.

^{1}H NMR (500 MHz, $CDCl_3$) δ 7.83 (t, *J* = 8.2 Hz, 2H), 7.55 – 7.40 (m, 2H), 7.39 – 7.19 (m, 4H), 6.12 (t, *J* = 4.7 Hz, 1H), 4.94 (t, *J* = 5.9 Hz, 1H), 4.04 – 3.61 (m, 2H), 3.19 (d, *J* = 4.8 Hz, 2H), 1.42 (s, 9H).

^{13}C NMR (126 MHz, $CDCl_3$) δ 170.24, 155.76, 134.29, 133.14, 132.88, 132.61, 129.73, 129.28, 128.84, 128.19, 127.91, 127.62, 124.10, 123.74, 80.03, 71.19, 42.70, 34.49, 28.55.

HRMS (ESI+): calcd for $C_{21}H_{23}NO_4$ $[M+H]^+$ 354.1700, found 354.1696.

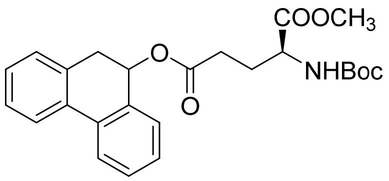

5-(9,10-dihydrophenanthren-9-yl) 1-methyl (tert-butoxycarbonyl)-L-glutamate (3cd): The title compound was prepared according to the general procedure B, but use 5 equiv acid. The crude residue was purified by column chromatography on silica gel with an eluent of hexanes/ethyl acetate (2:1) to furnish the pure compound as a yellow oil in 79% yield.

^{1}H NMR (500 MHz, $CDCl_3$) δ 7.83 (t, *J* = 7.7 Hz, 2H), 7.51 – 7.40 (m, 2H), 7.40 – 7.14 (m, 4H), 6.07 (q, *J* = 4.8 Hz, 1H), 5.10 – 4.95 (m, 1H), 4.28 (t, *J* = 7.3 Hz, 1H), 3.69 (d, *J* = 3.6 Hz, 3H), 3.17 (dd, *J* = 5.0, 2.8 Hz, 2H), 2.44 – 2.21 (m, 2H), 2.10 (s, 1H), 2.02 – 1.78 (m, 1H), 1.42 (s, 9H).

^{13}C NMR (126 MHz, $CDCl_3$) δ 172.82, 172.44 (d, *J* = 2.0 Hz), 155.71 (d, *J* = 69.6 Hz), 134.29 (d, *J* = 2.3 Hz), 133.42, 133.27 (d, *J* = 2.9 Hz), 132.92, 129.54, 129.26, 128.67 (d, *J* = 9.1 Hz), 128.12 (d, *J* = 2.6 Hz), 127.91 (d, *J* = 1.9 Hz), 127.55, 124.08, 123.73, 80.16, 70.35 (d, *J* = 10.8 Hz), 52.90, 52.51, 34.53 (d, *J* = 3.8 Hz), 30.56 (d, *J* = 4.7 Hz), 28.43, 28.16 (d, *J* = 95.0 Hz).

HRMS (ESI+): calcd for $C_{25}H_{29}NO_6$ $[M+H]^+$ 440.2068, found 440.2067.

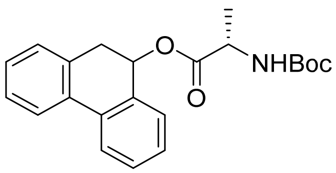

9,10-dihydrophenanthren-9-yl (*tert*-butoxycarbonyl)-L-alaninate (3ce): The title compound was prepared according to the general procedure B, but use 5 equiv acid. The crude residue was purified by column chromatography on silica gel with an eluent of hexanes/ethyl acetate (2:1) to furnish the pure compound as a brown solid in 59% yield.

^{1}H NMR (500 MHz, $CDCl_3$) δ 7.82 (td, *J* = 9.8, 8.7, 3.1 Hz, 2H), 7.57 – 7.39 (m, 2H), 7.38 – 7.06 (m, 4H), 6.07 (dt, *J* = 10.2, 5.1 Hz, 1H), 4.98 (dd, *J* = 19.1, 7.8 Hz, 1H), 4.40 – 4.04 (m, 1H), 3.17 (dd, *J* = 8.4, 5.3 Hz, 2H), 1.41 (s, 9H), 1.21 (t, *J* = 7.1 Hz, 3H).

^{13}C NMR (126 MHz, $CDCl_3$) δ 173.18, 155.22, 134.33, 134.22, 133.21, 132.61, 129.59 (d, *J* = 19.1 Hz), 129.21 (d, *J* = 20.1 Hz), 128.16 (d, *J* = 6.5 Hz), 127.92 (d, *J* = 14.8 Hz), 127.63

(d, J = 5.3 Hz), 124.11 (d, J = 4.6 Hz), 123.84, 123.70, 71.19, 49.49, 34.49 (d, J = 3.9 Hz), 28.57, 28.44, 18.92.
HRMS (ESI+): calcd for $C_{22}H_{25}NO_4$ $[M+H]^+$ 368.1856, found 368.1857.

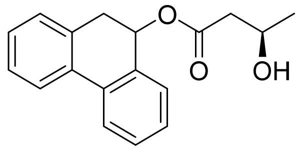

9,10-dihydrophenanthren-9-yl (3R)-3-hydroxybutanoate (3cf): The title compound was prepared according to the general procedure B, but use 5 equiv acid. The crude residue was purified by column chromatography on silica gel with an eluent of hexanes/ethyl acetate (2:1) to furnish the pure compound as a brown solid in 51% yield.
^{1}H NMR (500 MHz, $CDCl_3$) δ 7.84 (td, J = 7.3, 6.7, 1.4 Hz, 2H), 7.58 – 7.42 (m, 2H), 7.41 – 7.18 (m, 4H), 6.11 (t, J = 4.6 Hz, 1H), 4.09 (ddt, J = 9.4, 6.4, 3.3 Hz, 1H), 3.19 (dd, J = 5.0, 2.0 Hz, 2H), 2.77 (dd, J = 26.4, 3.8 Hz, 1H), 2.52 – 2.20 (m, 2H), 1.13 (dd, J = 6.3, 2.2 Hz, 3H).
^{13}C NMR (126 MHz, $CDCl_3$) δ 172.54 (d, J = 9.6 Hz), 134.33 (d, J = 3.6 Hz), 133.25 (d, J = 9.6 Hz), 133.15, 132.75 (d, J = 3.7 Hz), 129.73, 129.26 (d, J = 5.0 Hz), 128.82 (d, J = 16.6 Hz), 128.21 (d, J = 3.0 Hz), 127.97 (d, J = 6.0 Hz), 127.67 (d, J = 3.8 Hz), 124.17, 123.82 (d, J = 6.2 Hz), 70.51 (d, J = 6.5 Hz), 64.36, 43.16 (d, J = 9.2 Hz), 34.59 (d, J = 1.6 Hz), 22.41 (d, J = 2.0 Hz).
HRMS (ESI+): calcd for $C_{18}H_{18}O_3$ $[M+H]^+$ 283.1329, found 283.1325.

2. Supplementary Figures

2.1 Spectral Data of quinoline derivatives

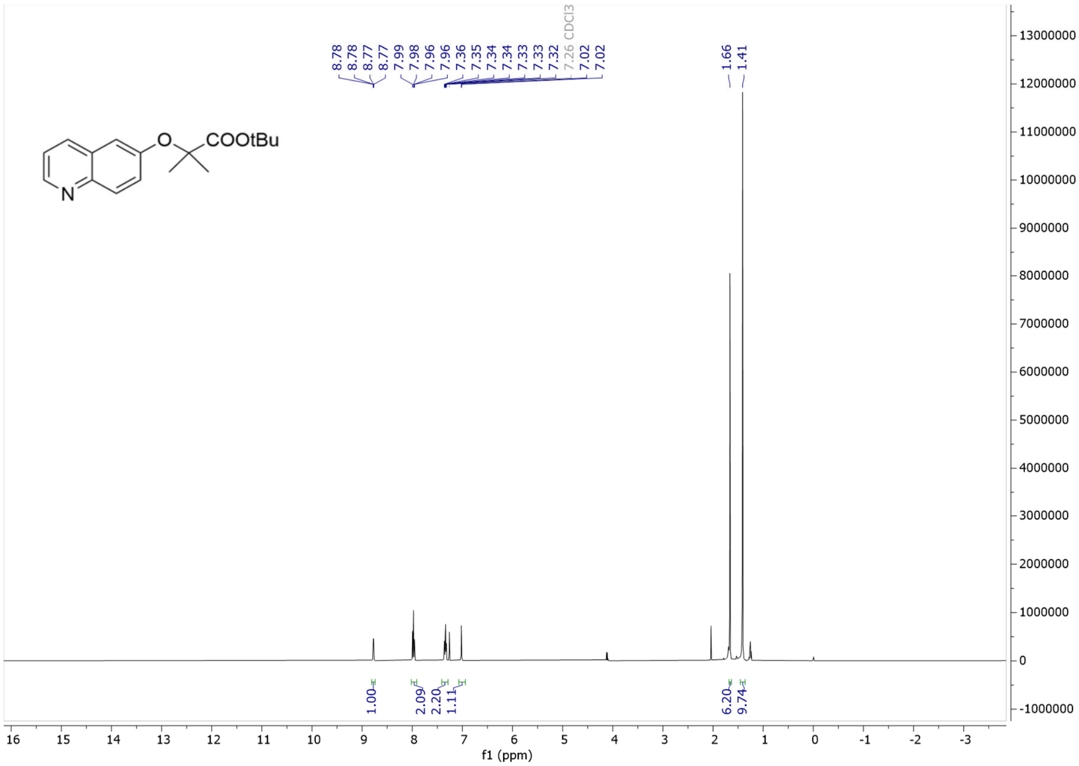

Supplementary Figure 9. ^{1}H NMR of **1am** (500 MHz, rt, $CDCl_3$).

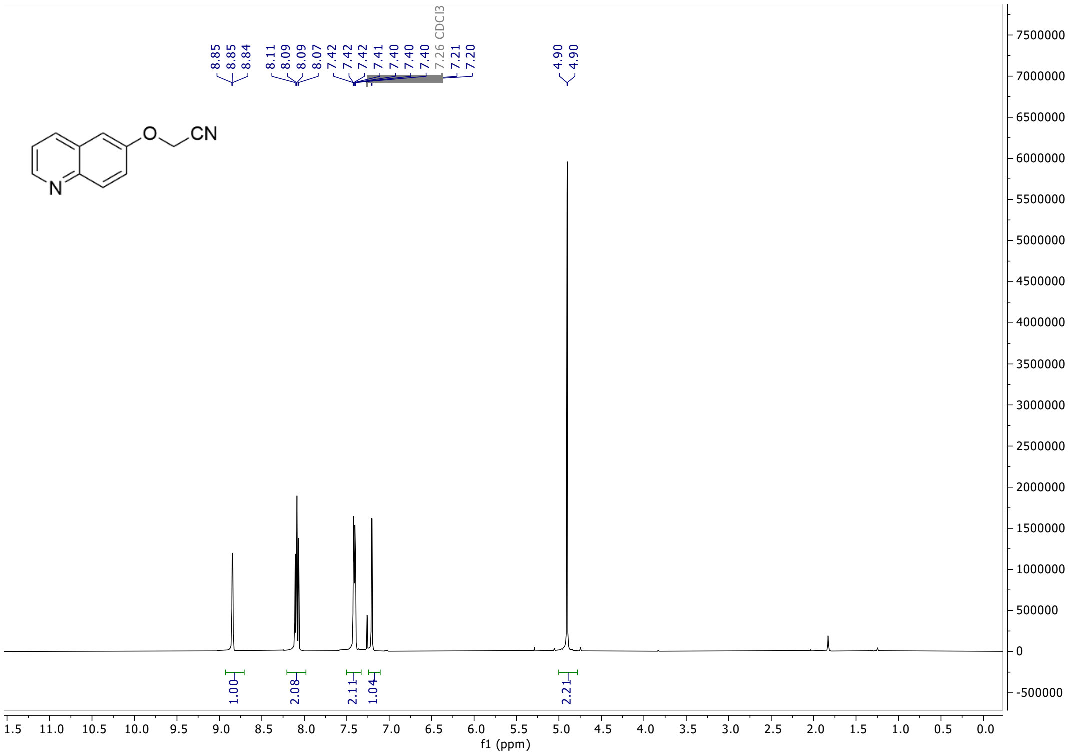

Supplementary Figure 10. ^{1}H NMR of **1ao** (500 MHz, rt, $CDCl_3$).

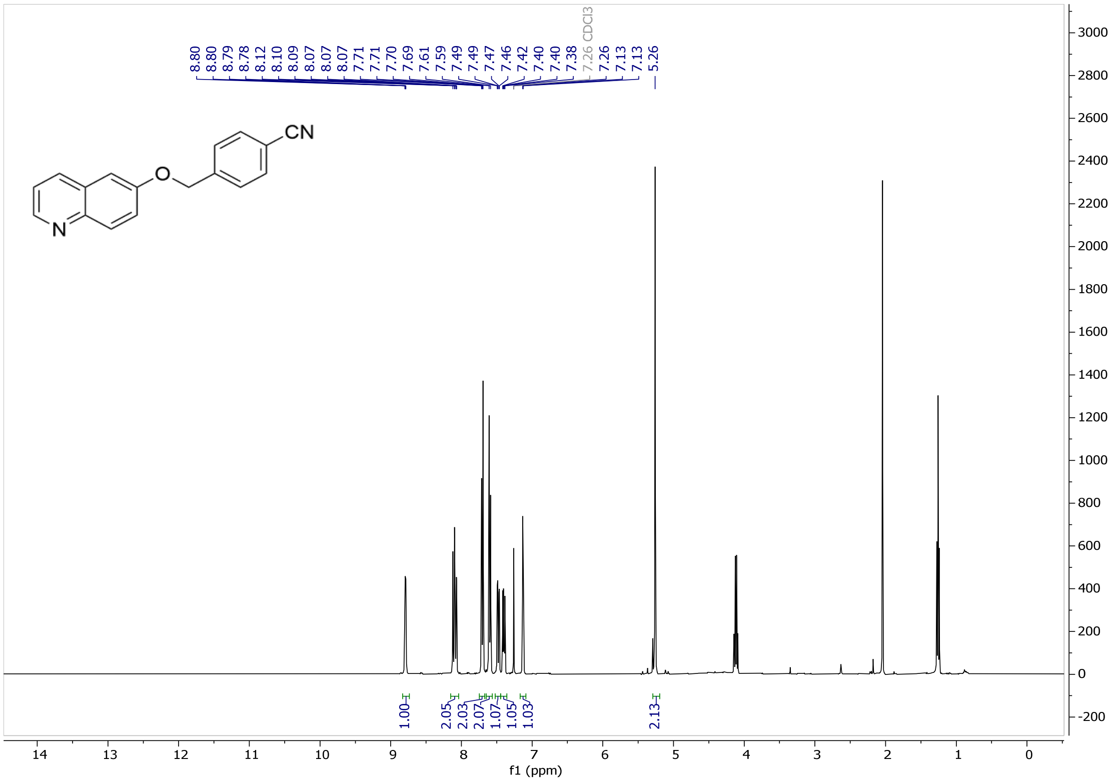

Supplementary Figure 11. ^{1}H NMR of **1ac** (500 MHz, rt, $CDCl_3$).

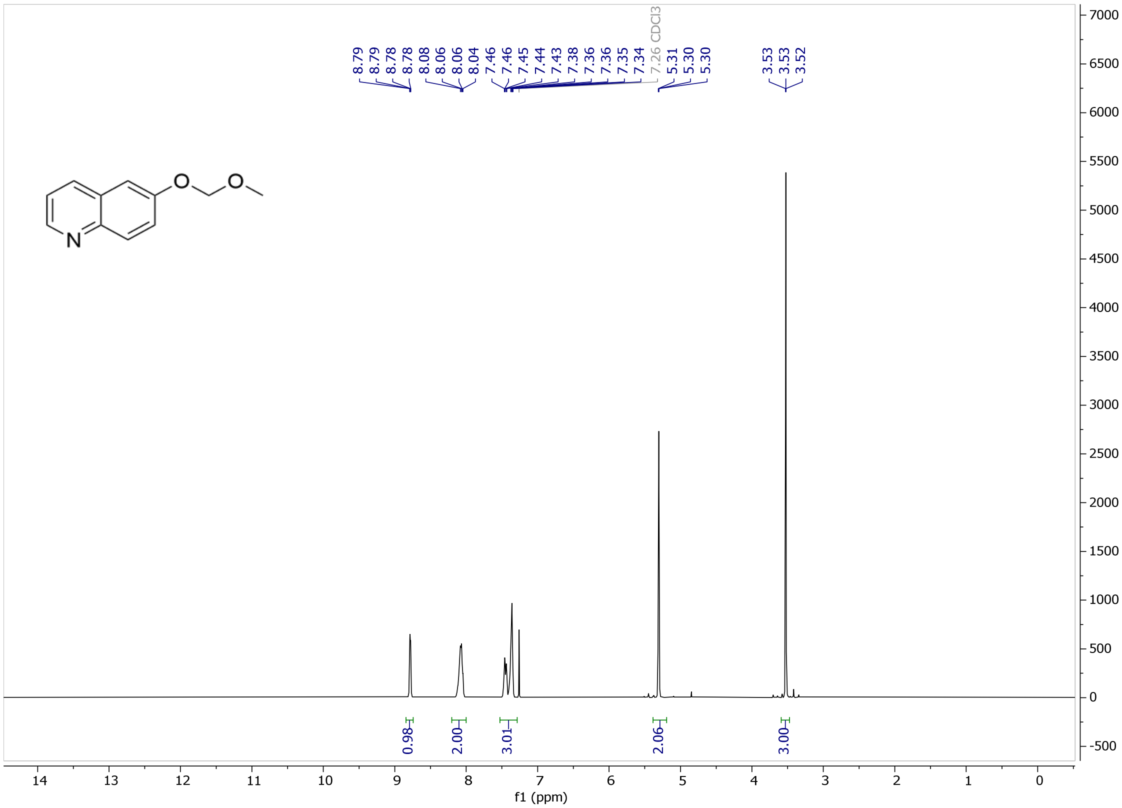

Supplementary Figure 12. ^{1}H NMR of **1ag** (500 MHz, rt, $CDCl_3$).

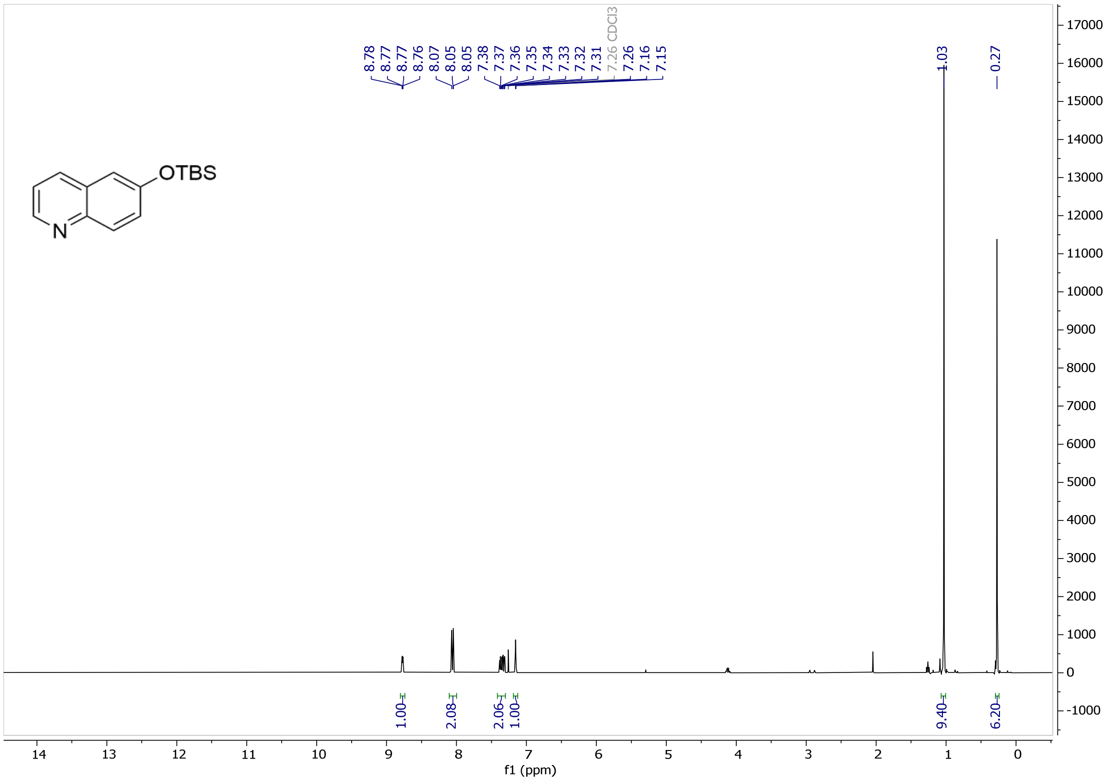

Supplementary Figure 13. ^{1}H NMR of **1ae** (500 MHz, rt, $CDCl_3$).

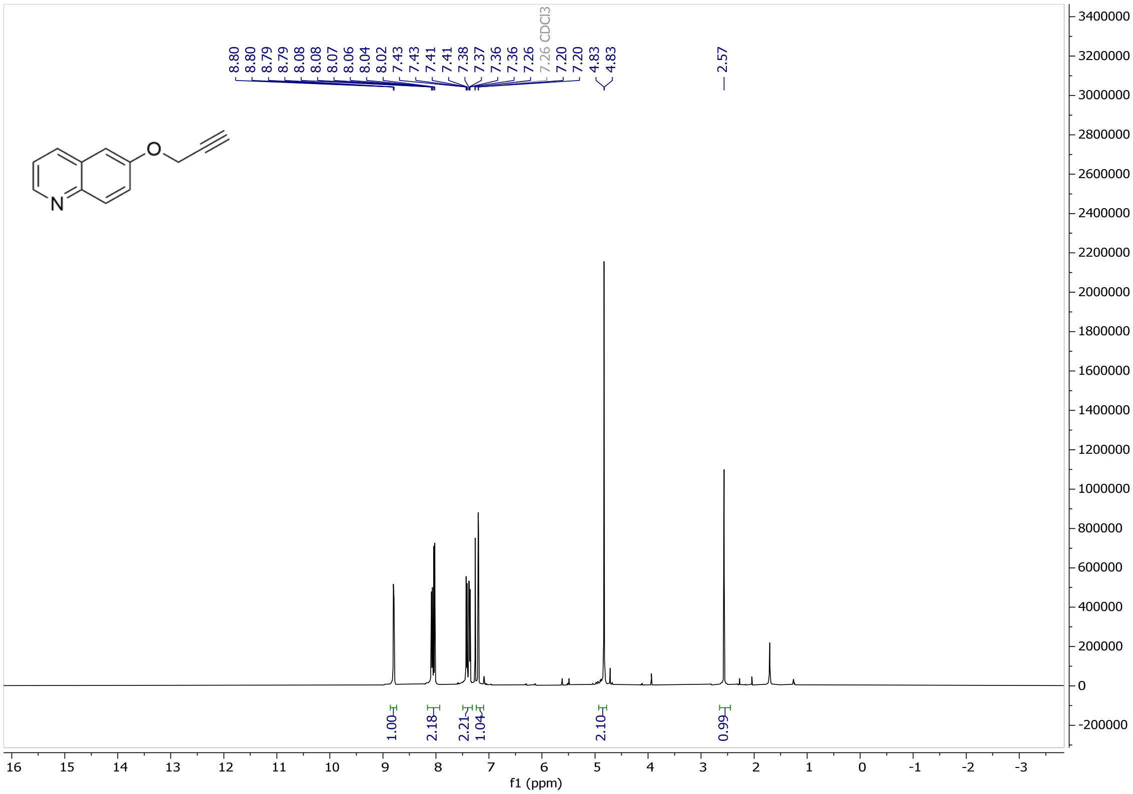

Supplementary Figure 14. ^{1}H NMR of **1an** (500 MHz, rt, $CDCl_3$).

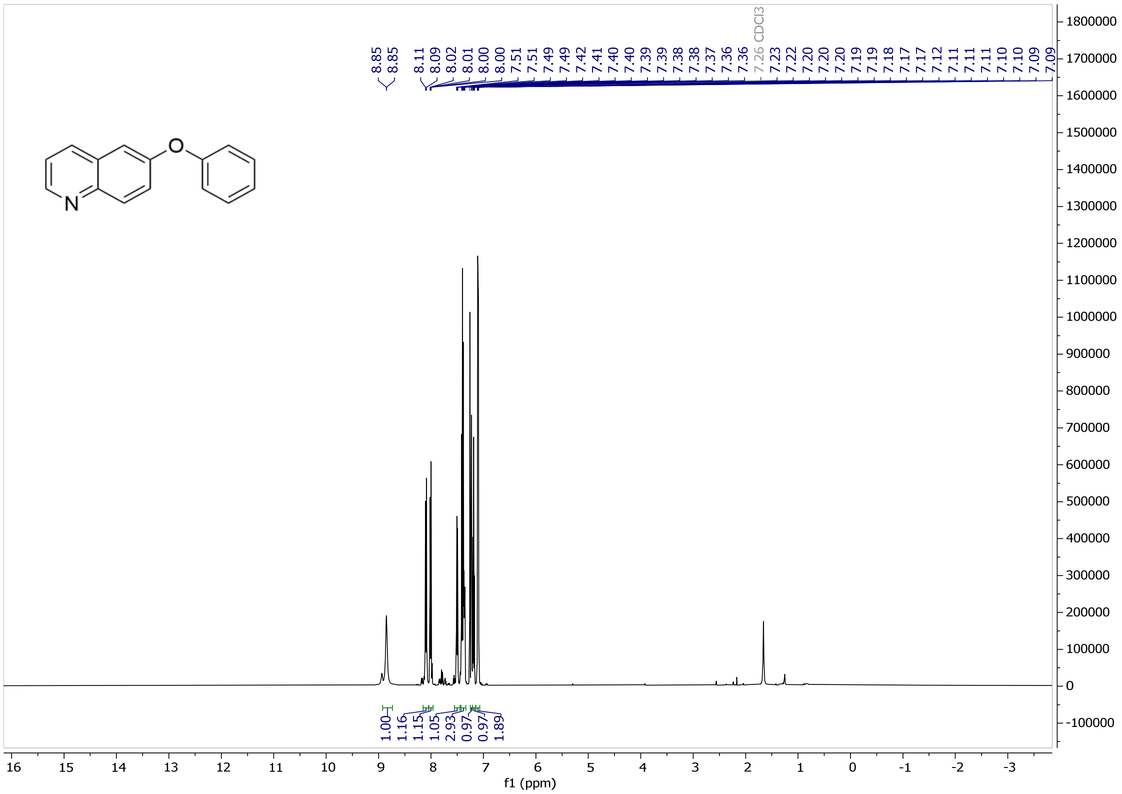

Supplementary Figure 15. ^{1}H NMR of **1ah** (500 MHz, rt, $CDCl_3$).

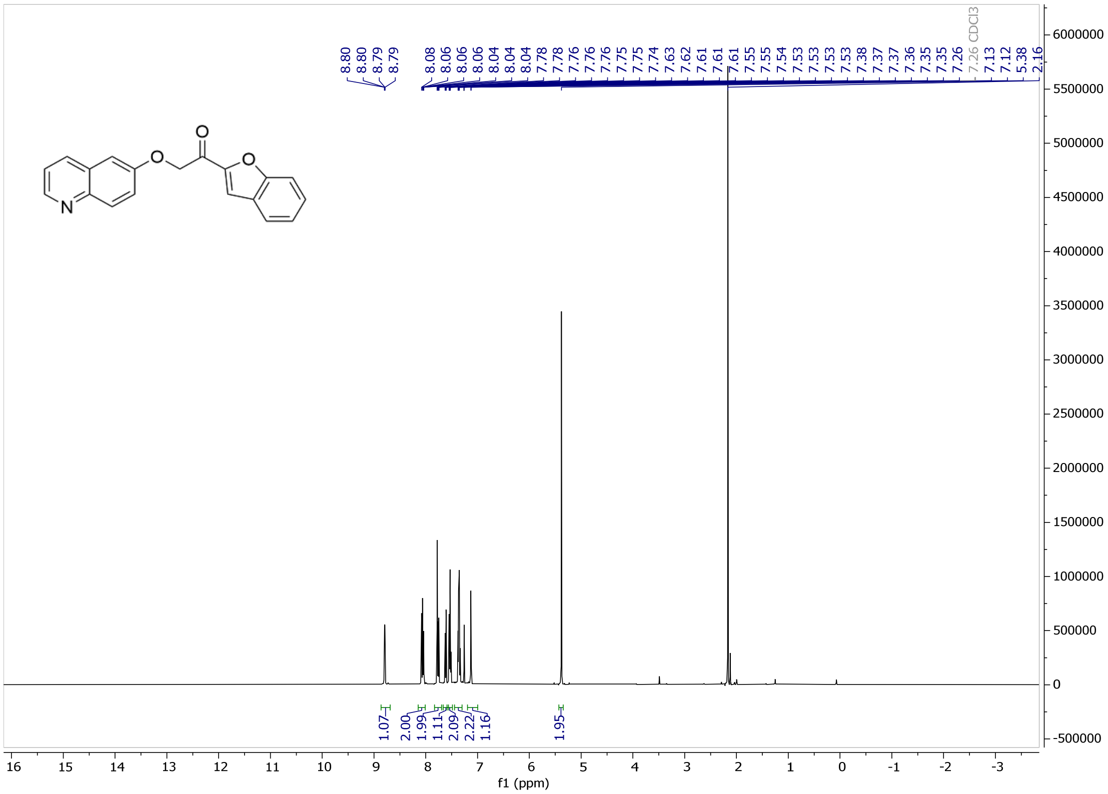

Supplementary Figure 16. ^{1}H NMR of **1al** (500 MHz, rt, $CDCl_3$).

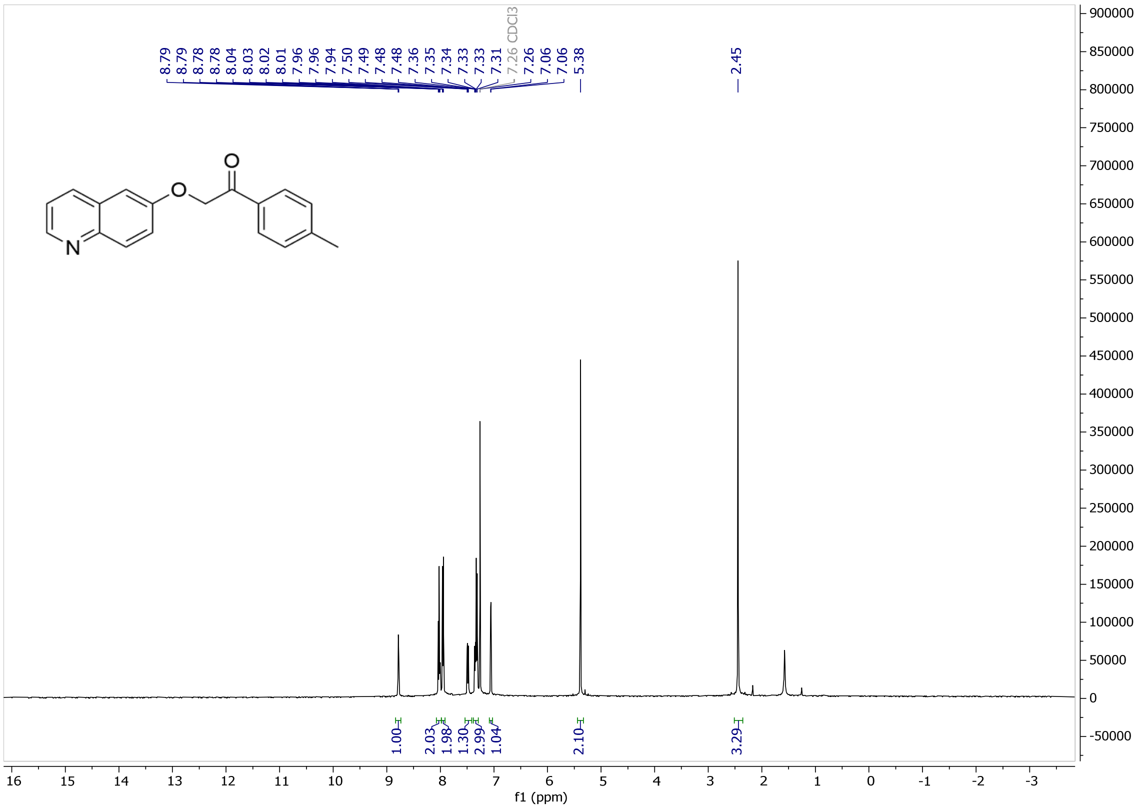

Supplementary Figure 17. ^{1}H NMR of **1ai** (500 MHz, rt, $CDCl_3$).

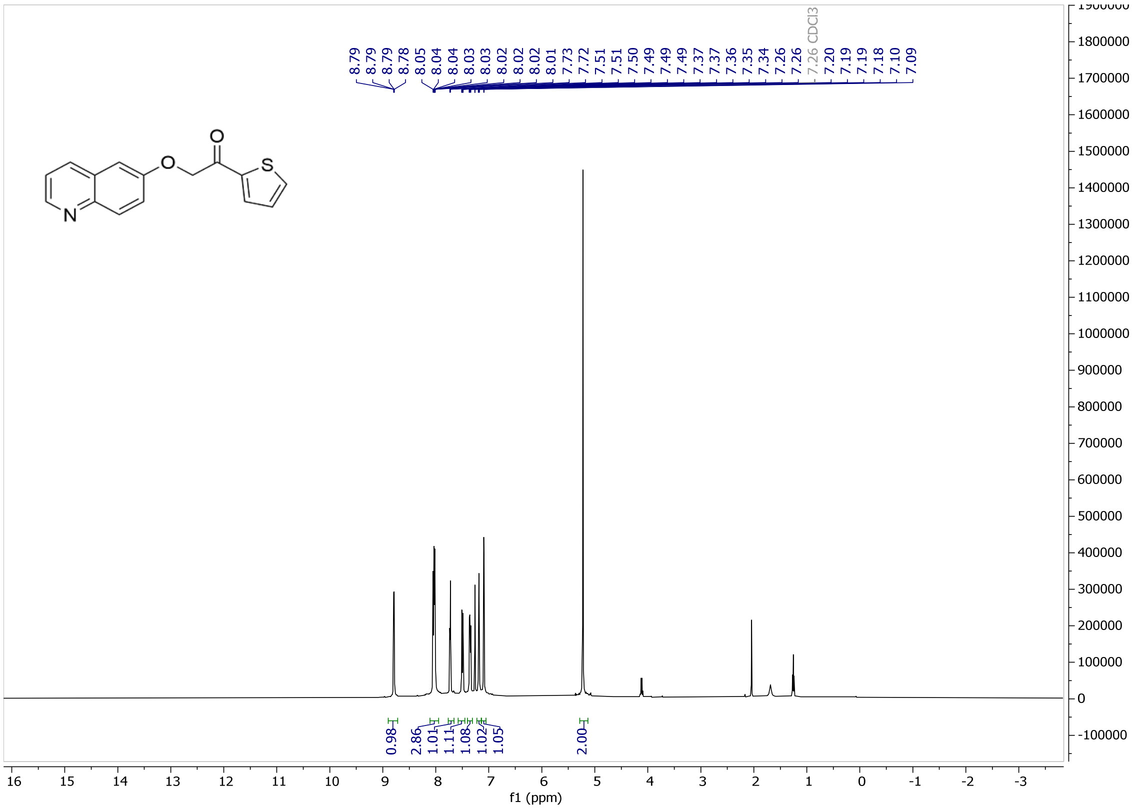

Supplementary Figure 18. ^{1}H NMR of **1aj** (500 MHz, rt, $CDCl_3$).

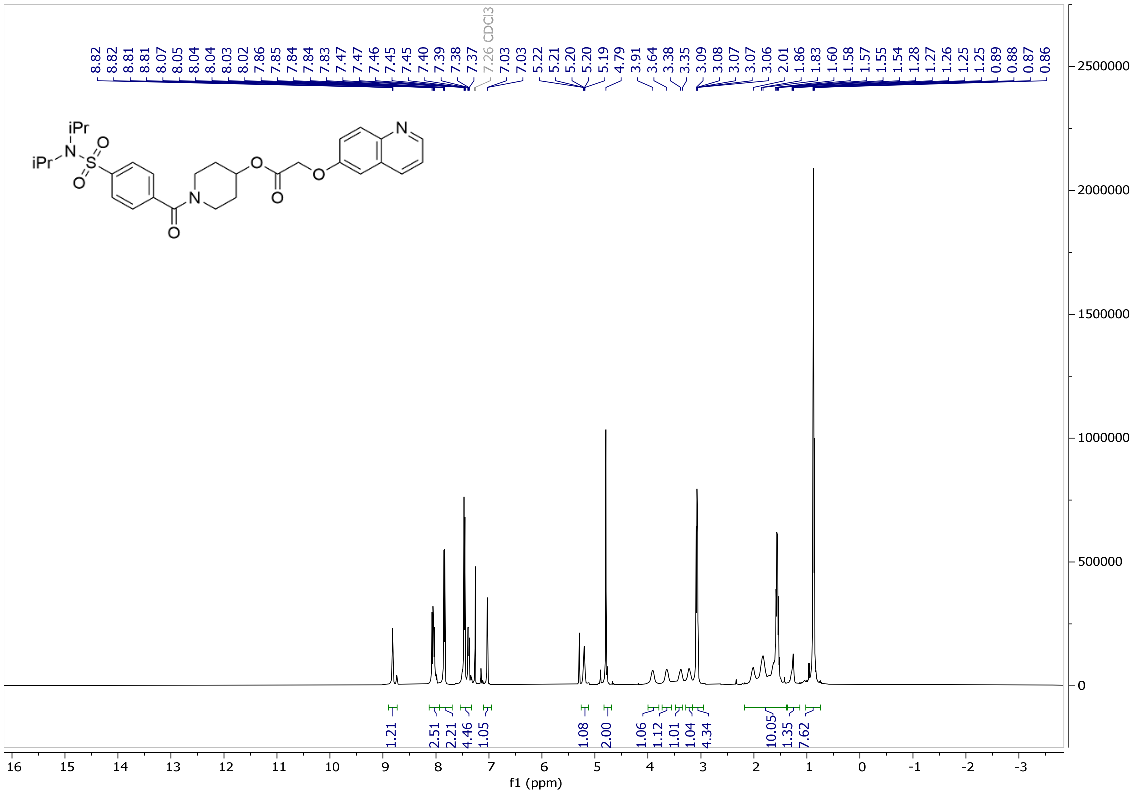

Supplementary Figure 19. ^{1}H NMR of **1ax** (500 MHz, rt, $CDCl_3$).

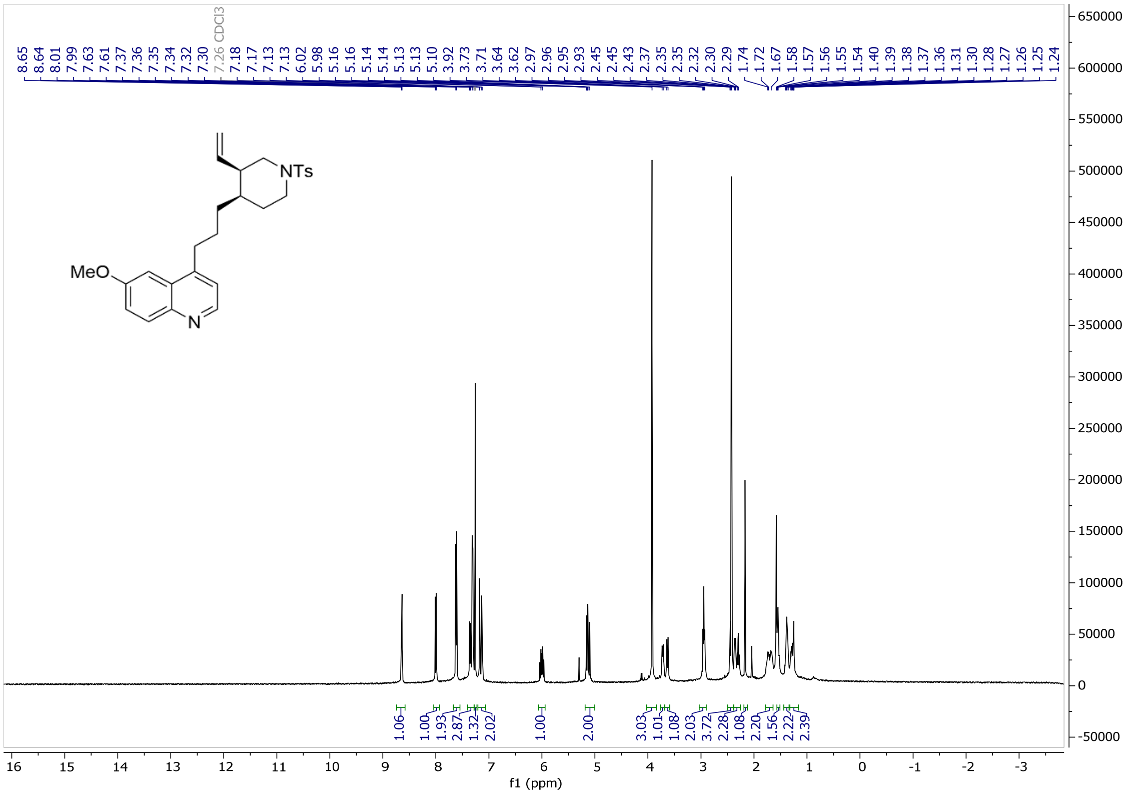

Supplementary Figure 20. ^{1}H NMR of **1as** (500 MHz, rt, $CDCl_3$).

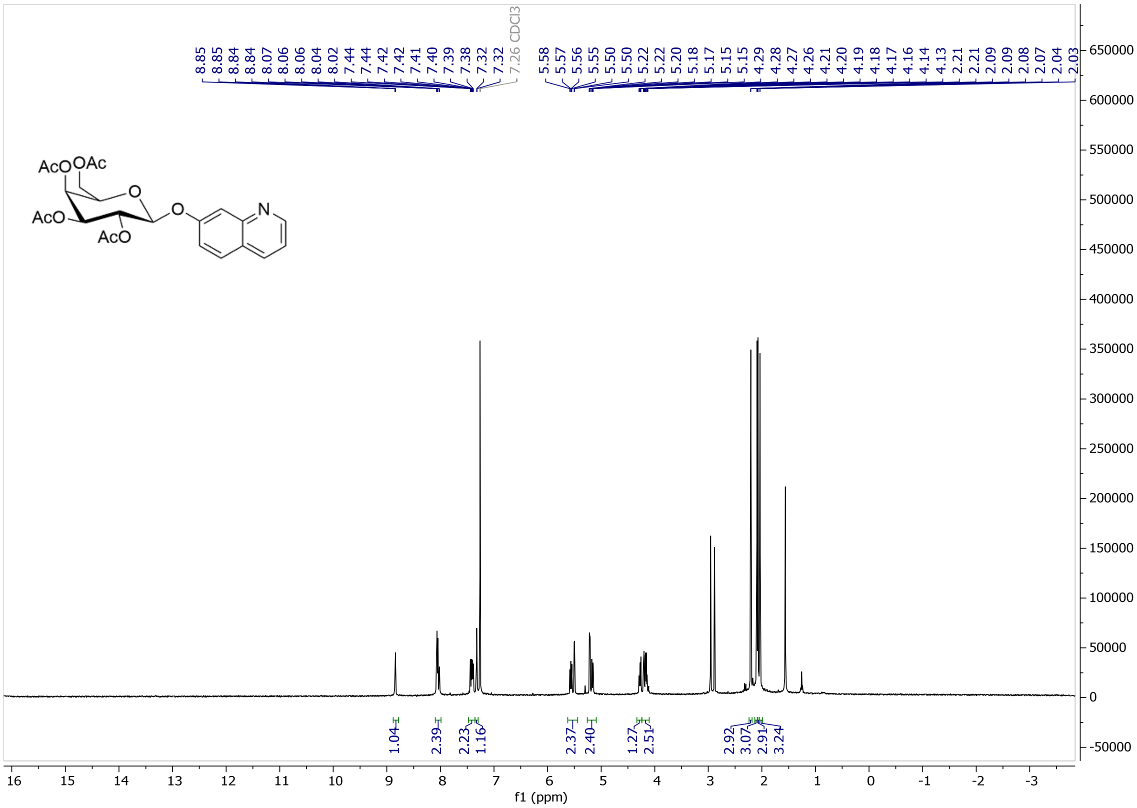

Supplementary Figure 21. ^{1}H NMR of **1av** (500 MHz, rt, $CDCl_3$).

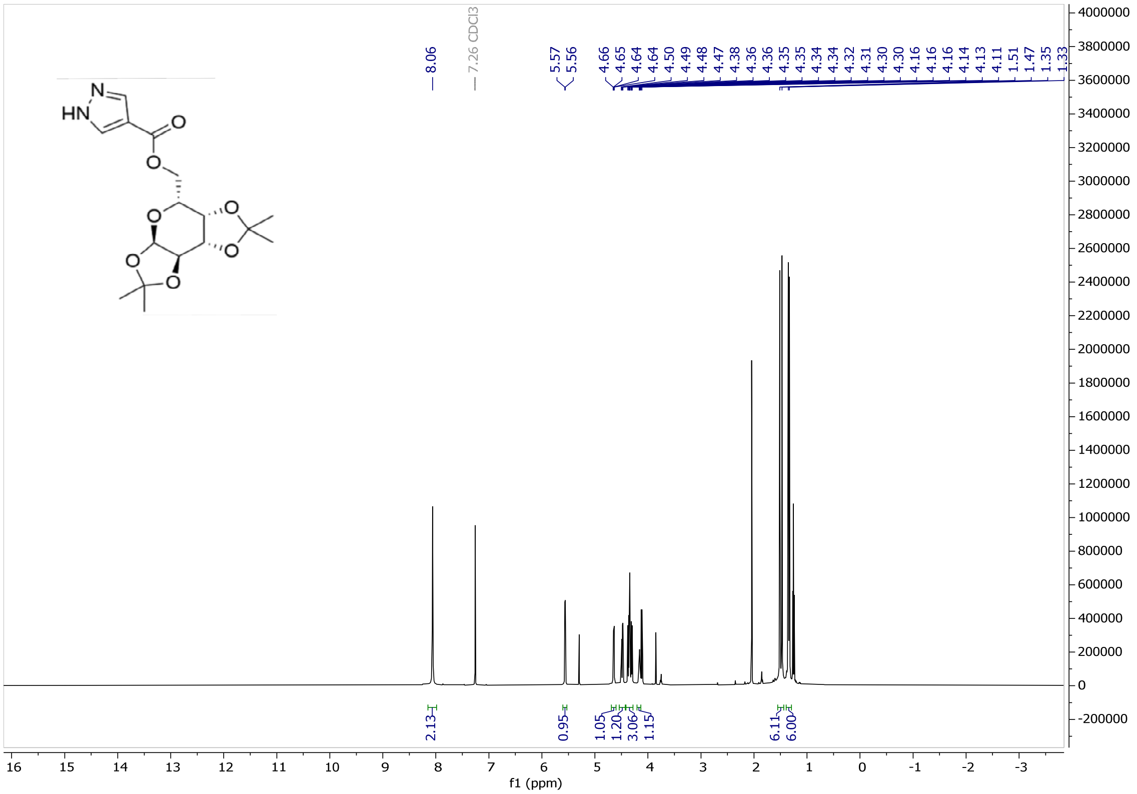

Supplementary Figure 22. ^{1}H NMR of **1au** (500 MHz, rt, $CDCl_3$).

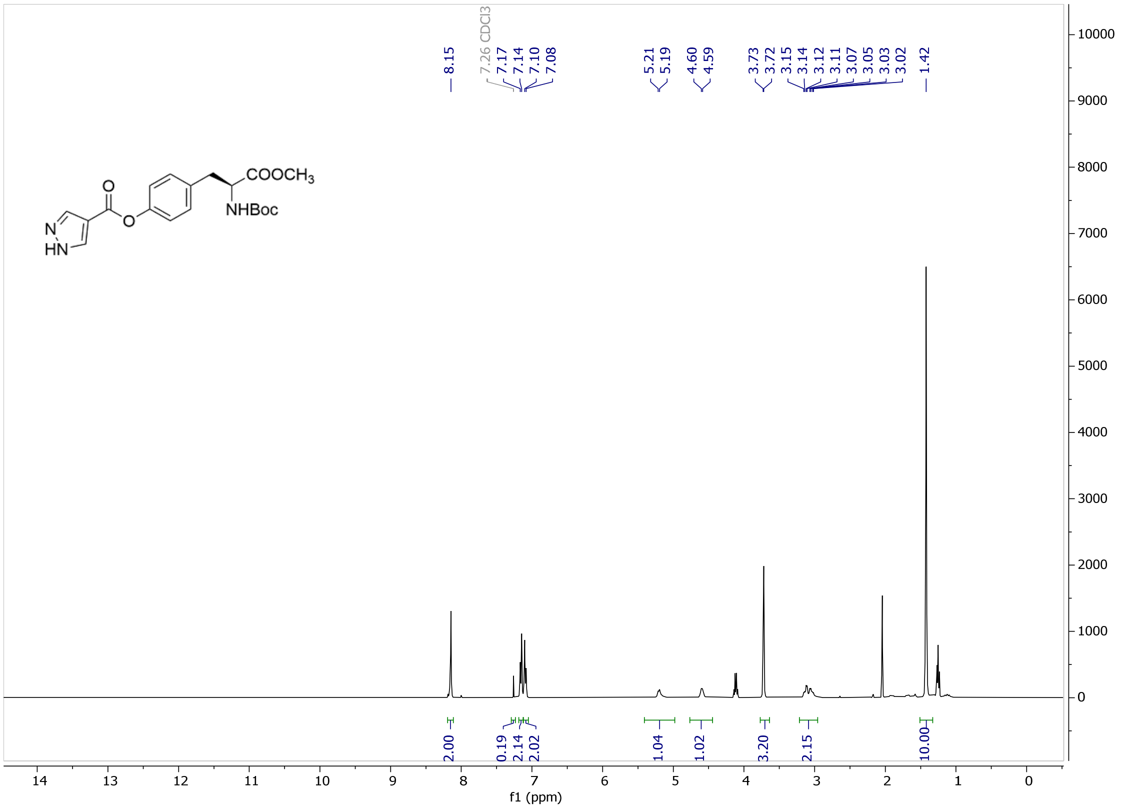

Supplementary Figure 23. ^{1}H NMR of **2ay** (500 MHz, rt, $CDCl_3$).

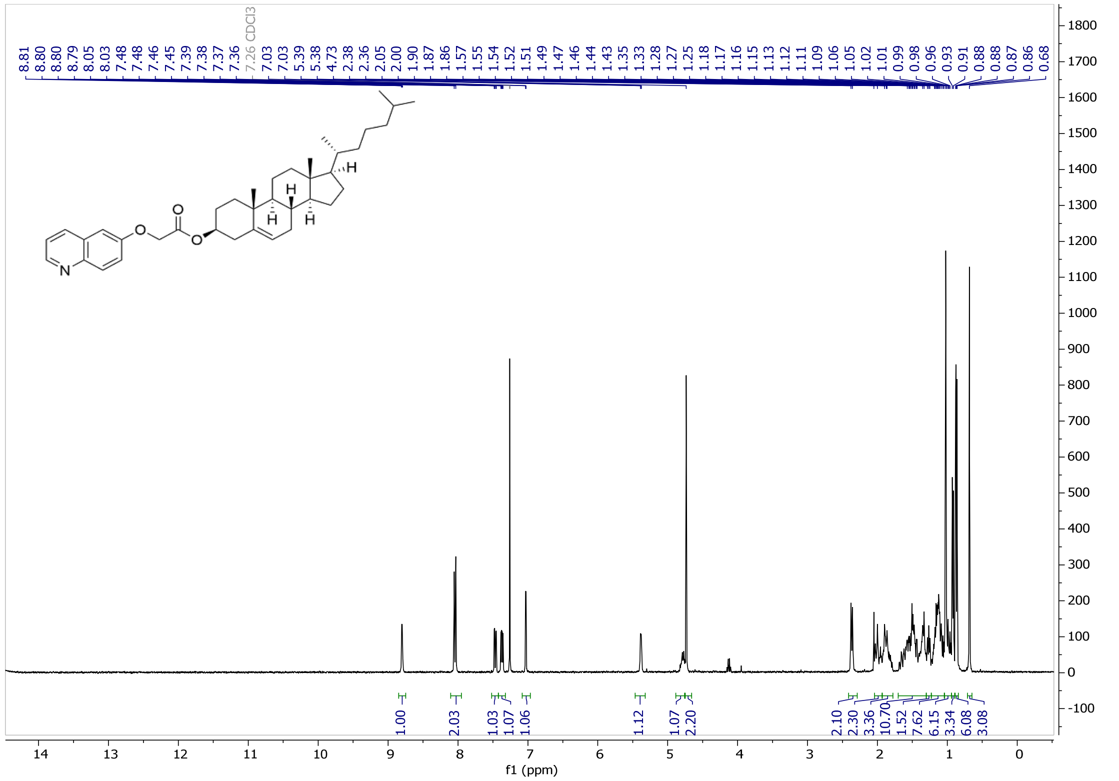

Supplementary Figure 24. ^{1}H NMR of **1az** (500 MHz, rt, $CDCl_3$).

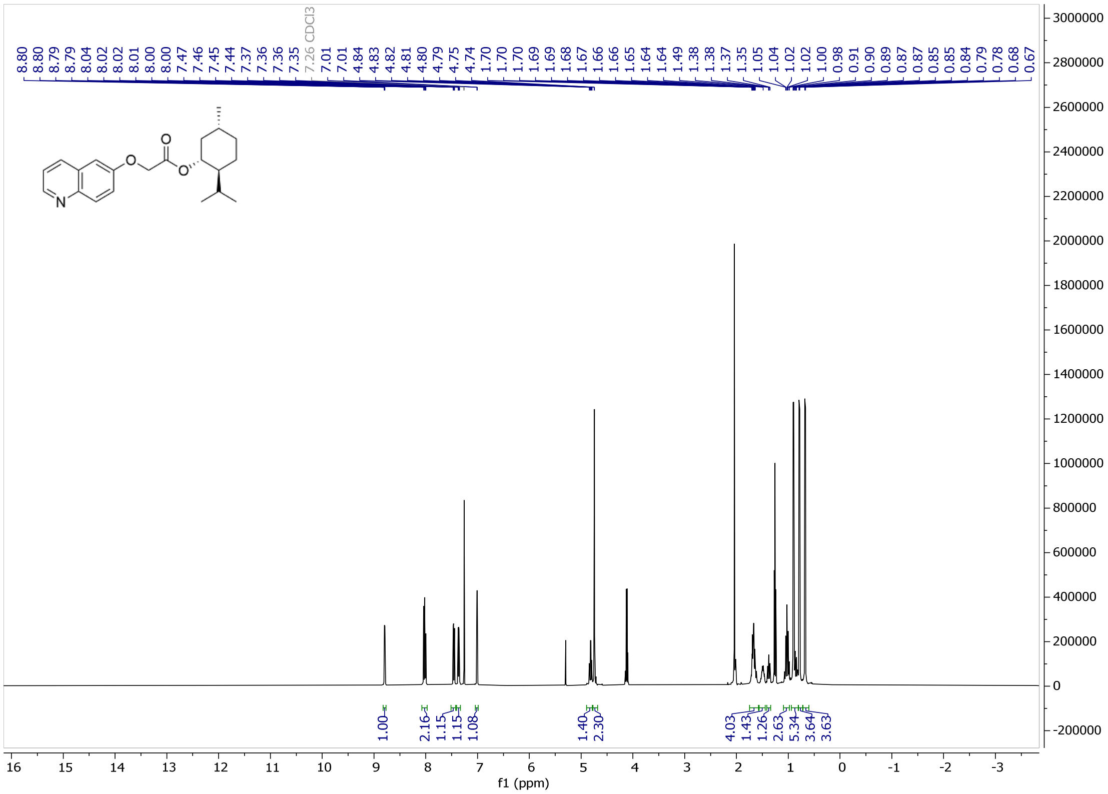

Supplementary Figure 25. ^{1}H NMR of **1aw** (500 MHz, rt, $CDCl_3$).

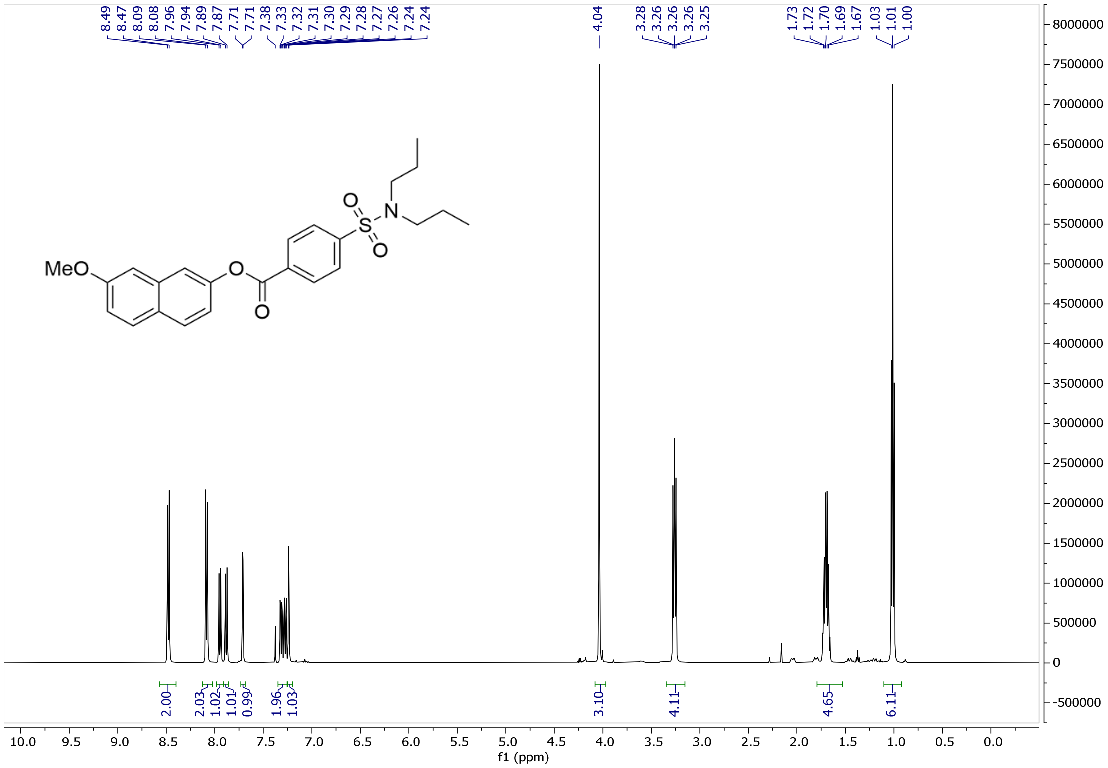

Supplementary Figure 26. ^{1}H NMR of **1bl** (500 MHz, rt, $CDCl_3$).

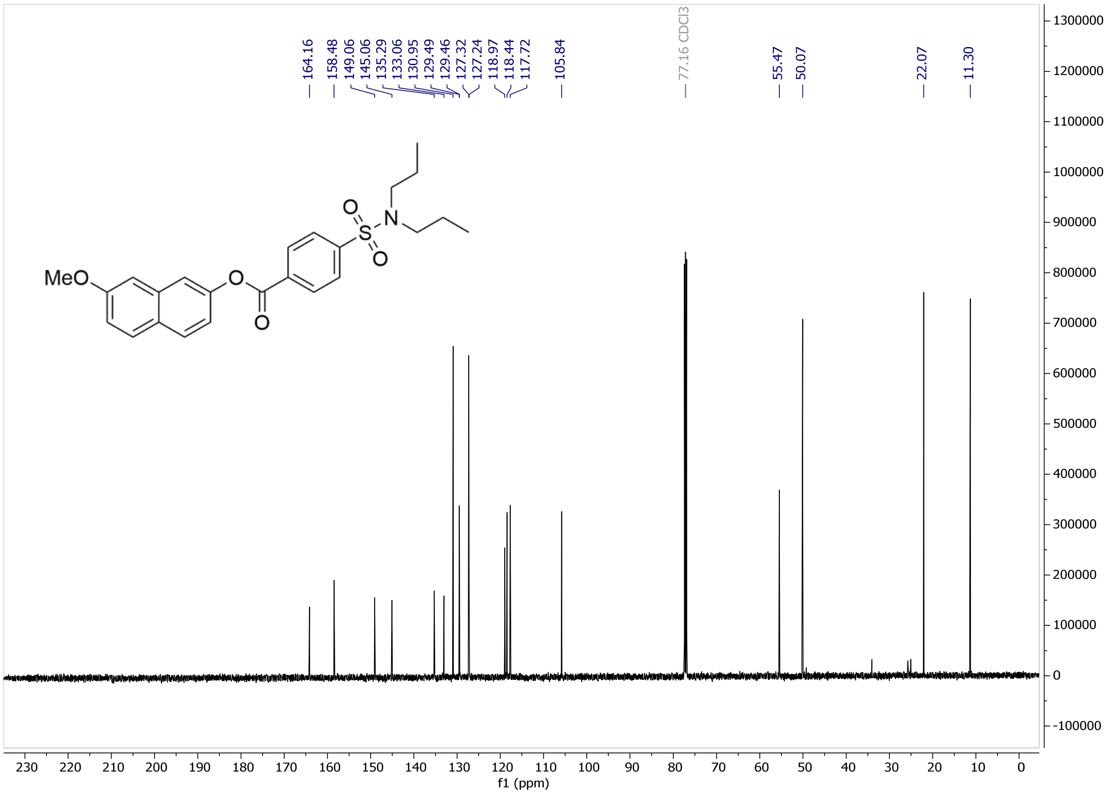

Supplementary Figure 27. ^{13}C NMR of **1bl** (500 MHz, rt, $CDCl_3$).

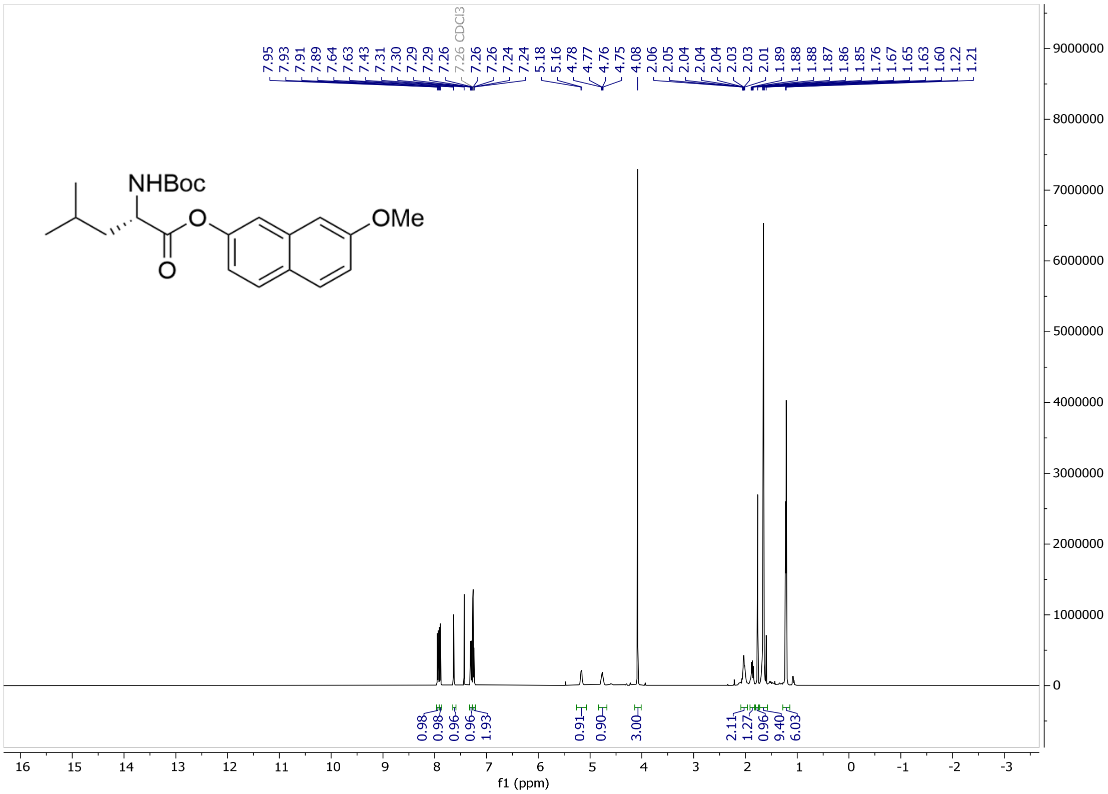

Supplementary Figure 28. ^{1}H NMR of **1bn** (500 MHz, rt, $CDCl_3$).

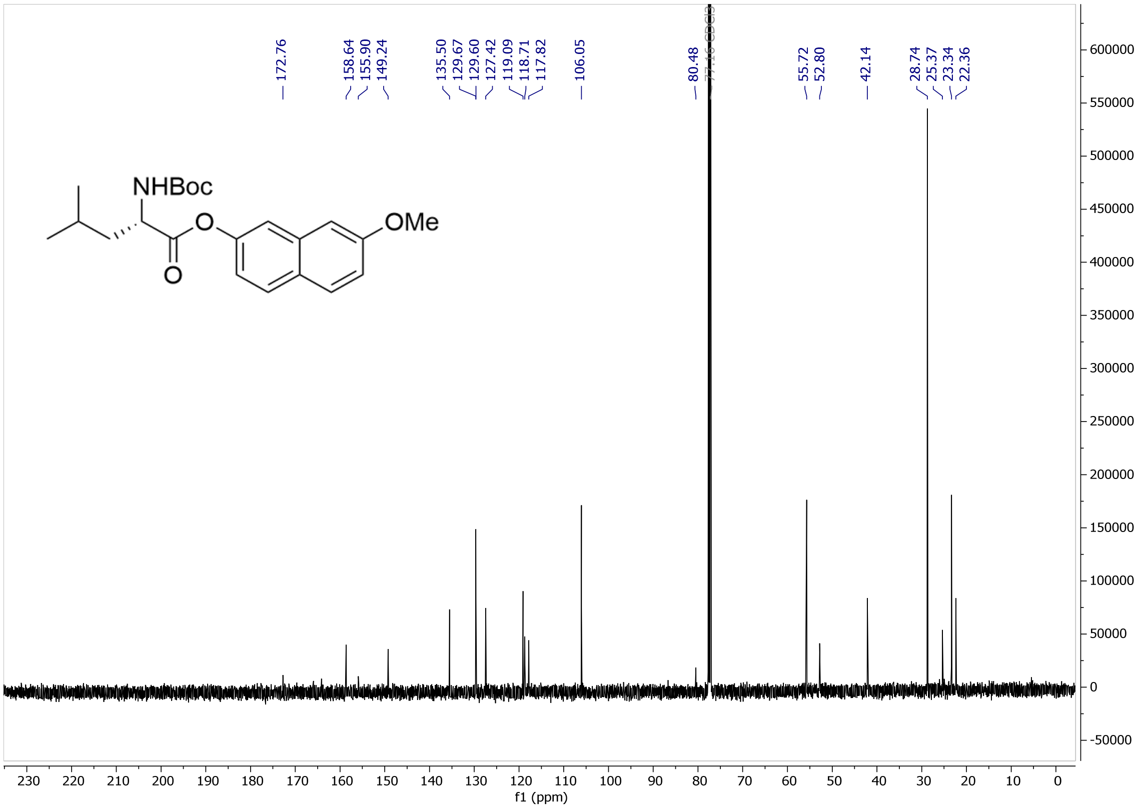

Supplementary Figure 29. ^{13}C NMR of **1bn** (500 MHz, rt, $CDCl_3$).

3.2 Spectral Data of dearomatized arenes

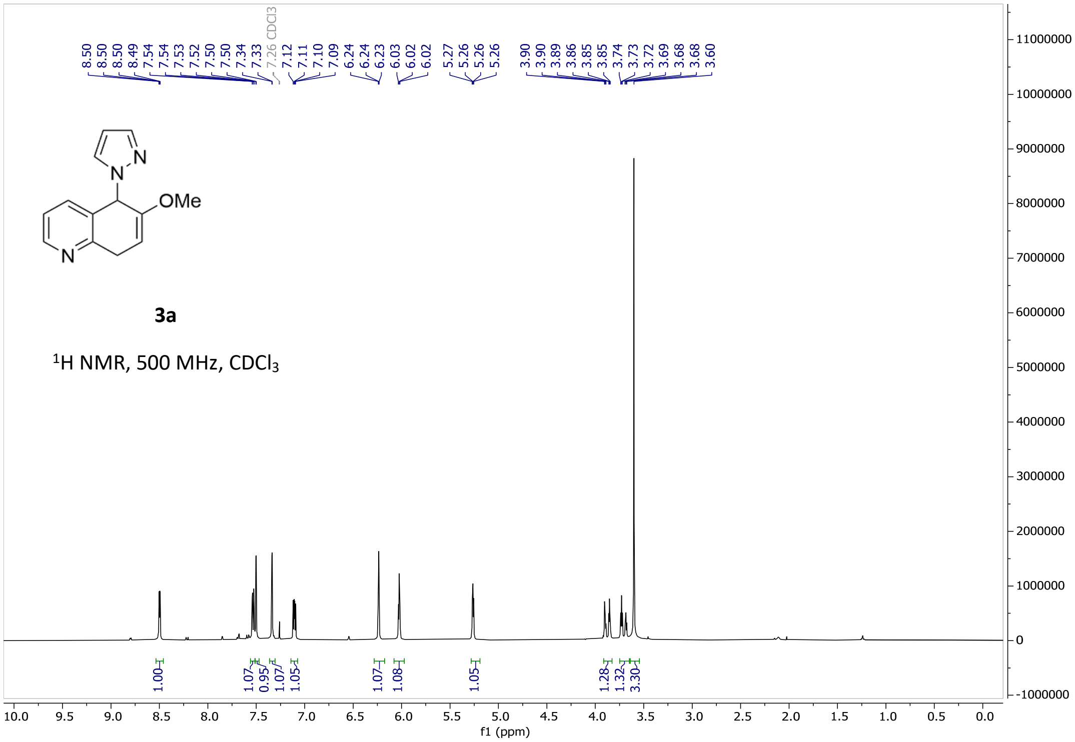

Supplementary Figure 30. ^{1}H NMR spectra of **3a** (500 MHz, rt, $CDCl_3$).

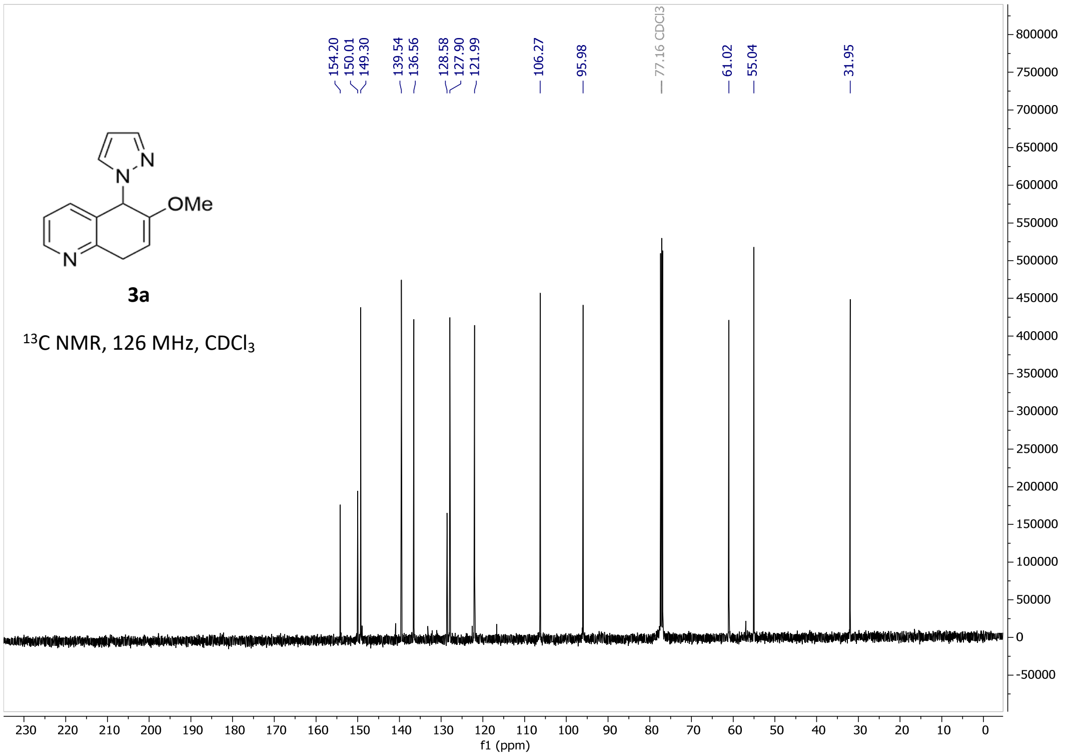

Supplementary Figure 31. ^{13}C NMR spectra of **3a** (126 MHz, rt, $CDCl_3$).

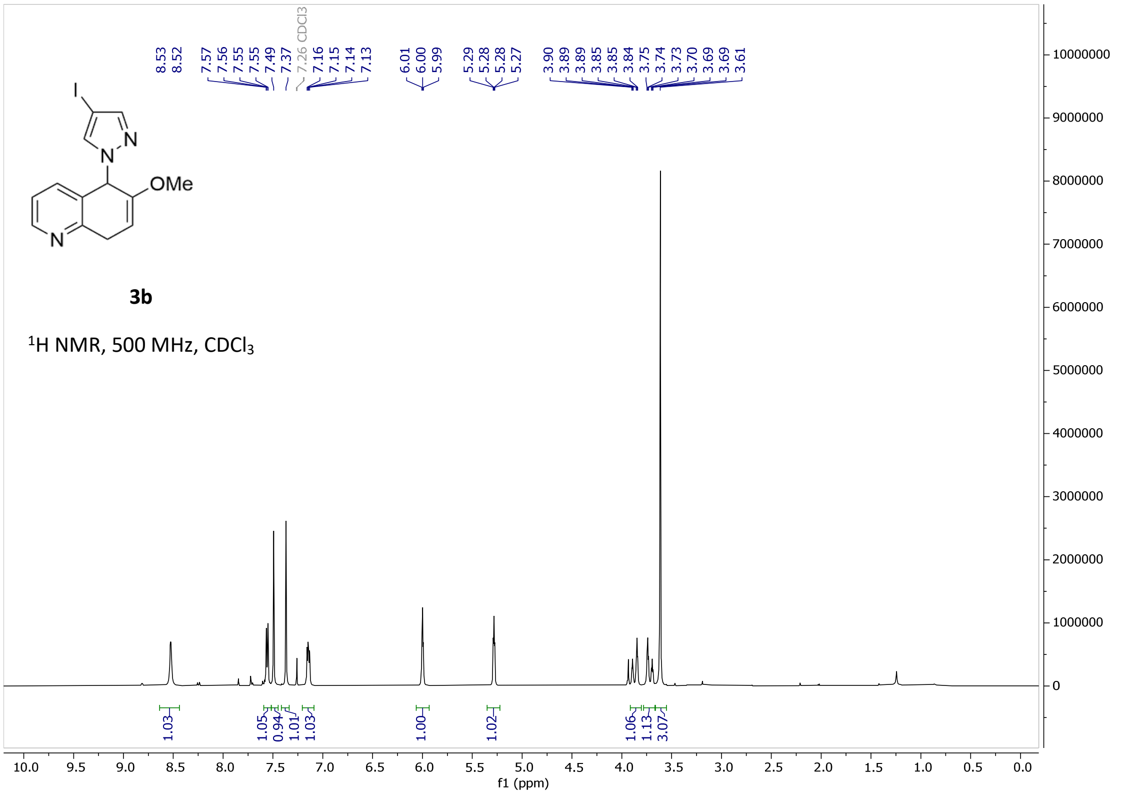

Supplementary Figure 32. ^{1}H NMR spectra of **3b** (500 MHz, rt, $CDCl_3$).

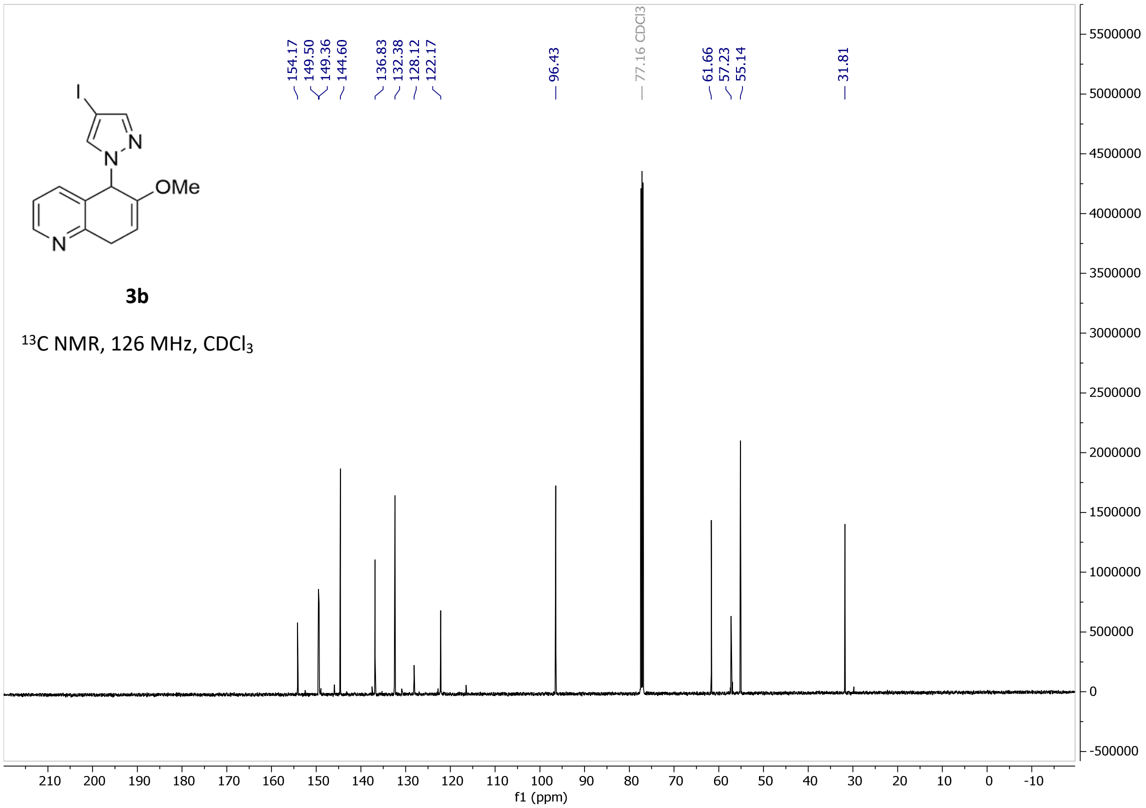

Supplementary Figure 33. ^{13}C NMR spectra of **3b** (126 MHz, rt, $CDCl_3$).

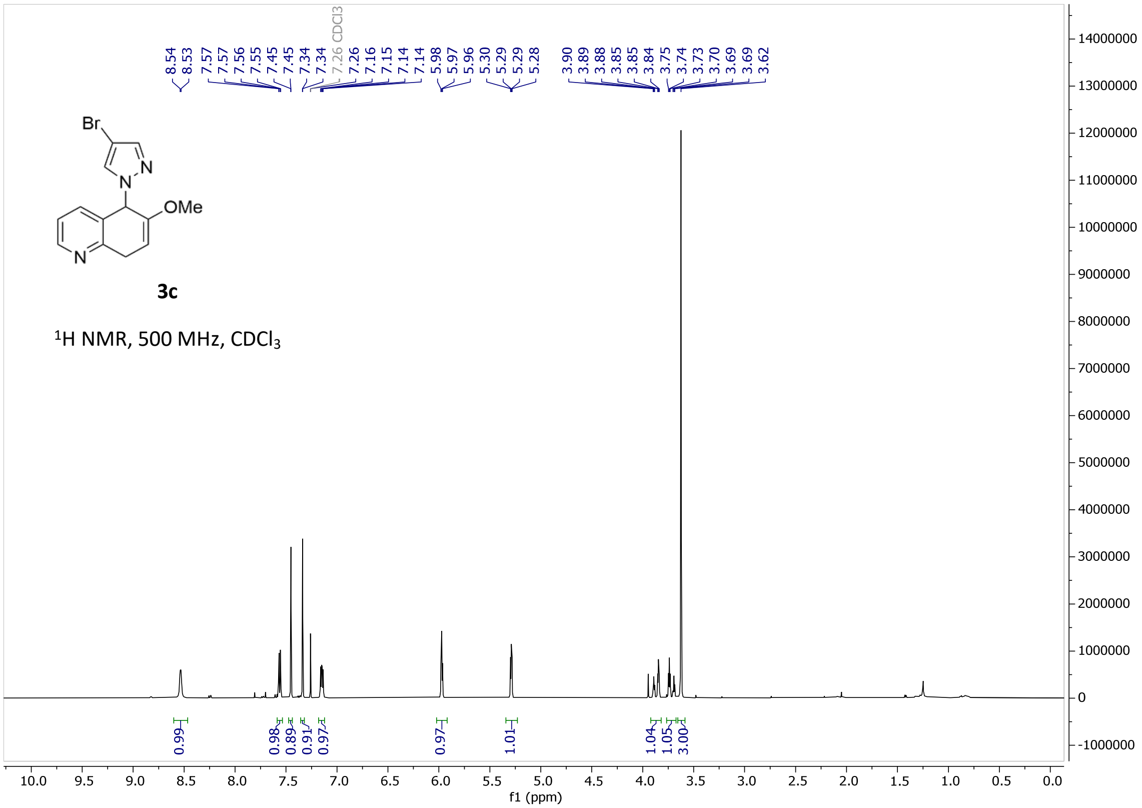

Supplementary Figure 34. ^{1}H NMR spectra of **3c** (500 MHz, rt, $CDCl_3$).

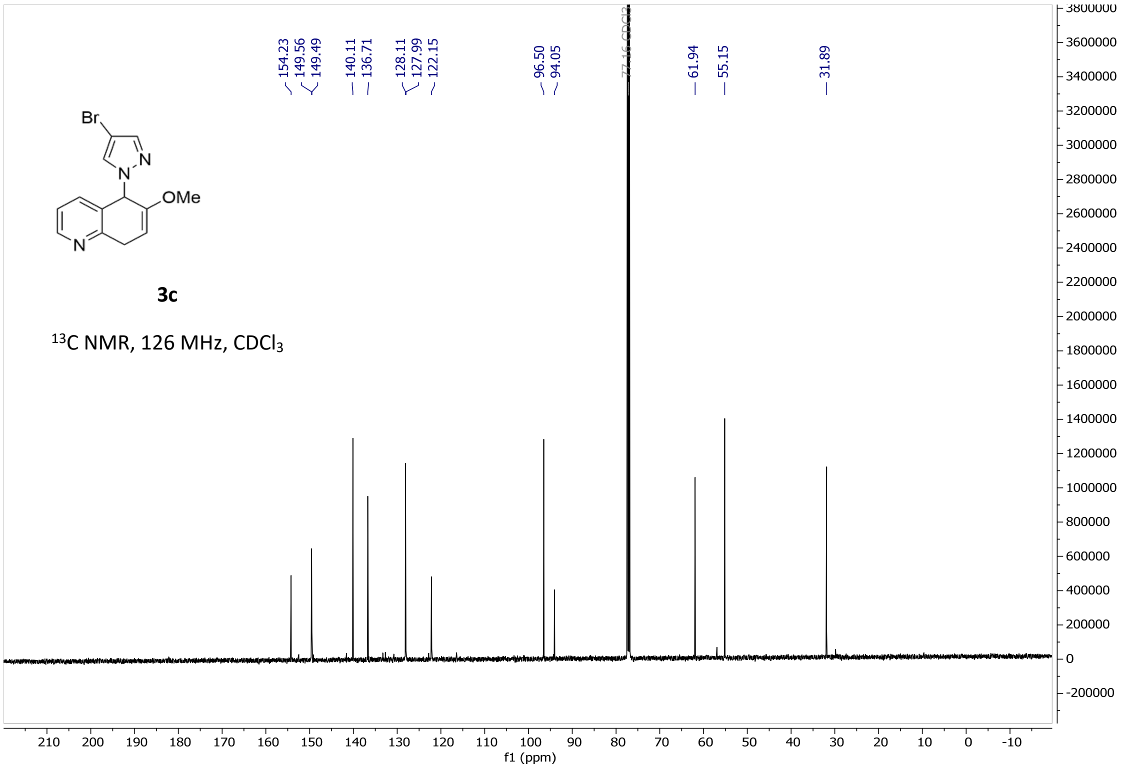

Supplementary Figure 35. ^{13}C NMR spectra of **3b** (126 MHz, rt, $CDCl_3$).

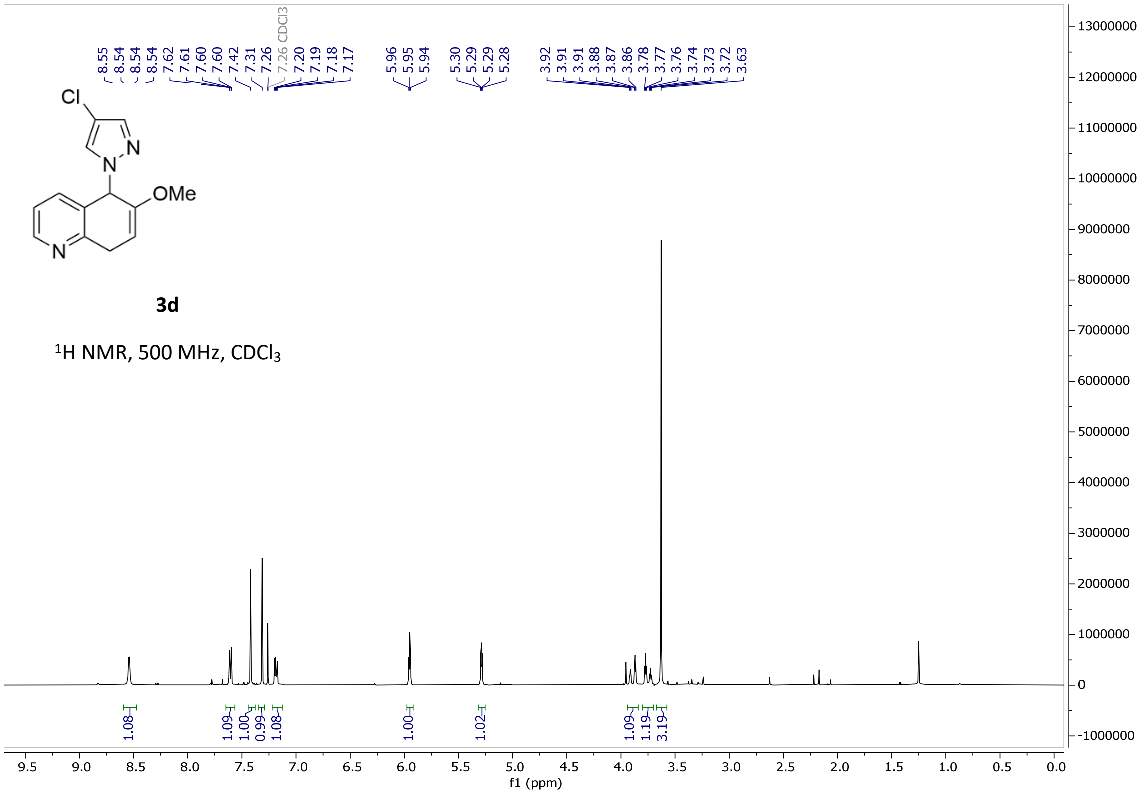

Supplementary Figure 36. ^{1}H NMR spectra of **3d** (500 MHz, rt, $CDCl_3$).

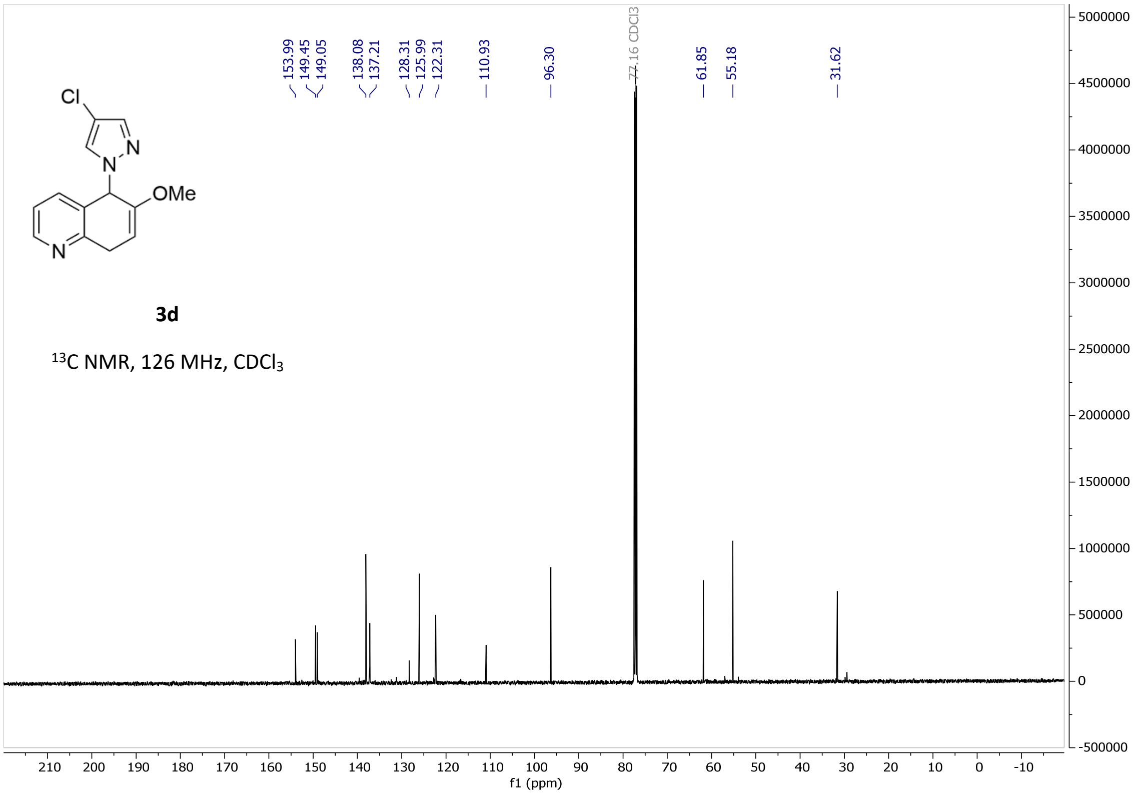

Supplementary Figure 37. ^{13}C NMR spectra of **3d** (126 MHz, rt, $CDCl_3$).

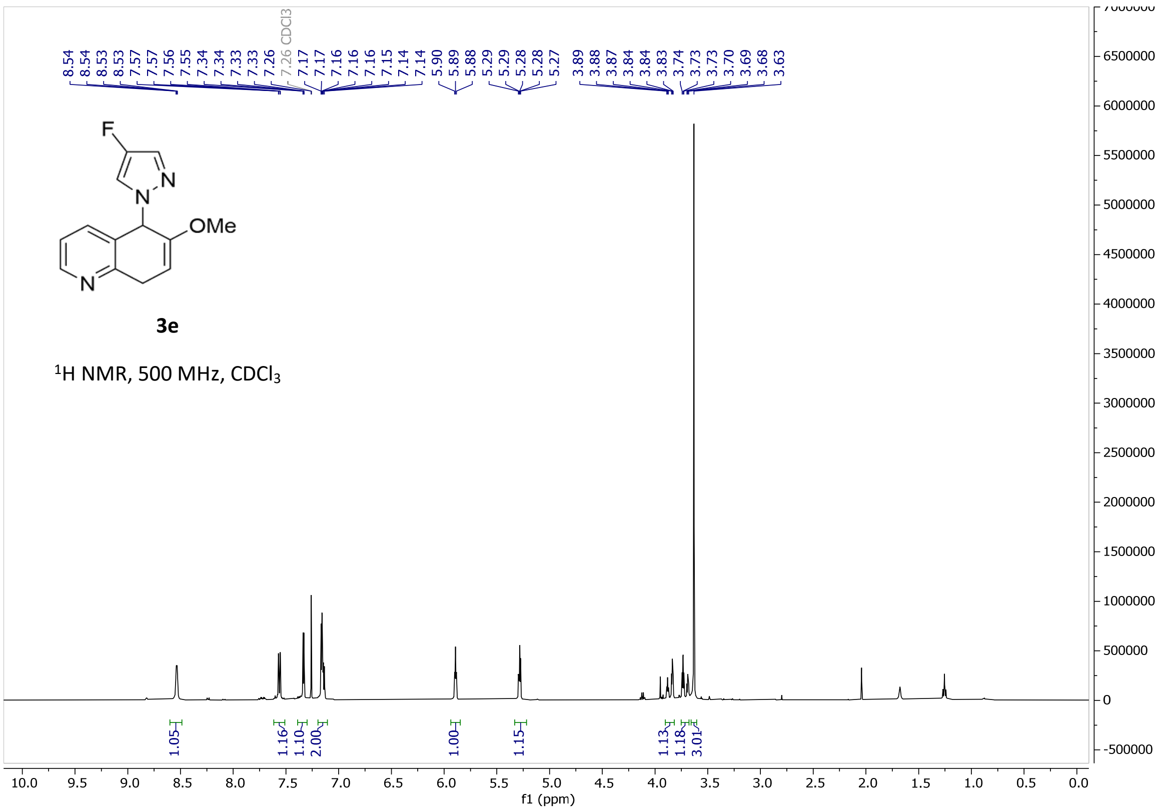

Supplementary Figure 38. ^{1}H NMR spectra of **3e** (500 MHz, rt, $CDCl_3$).

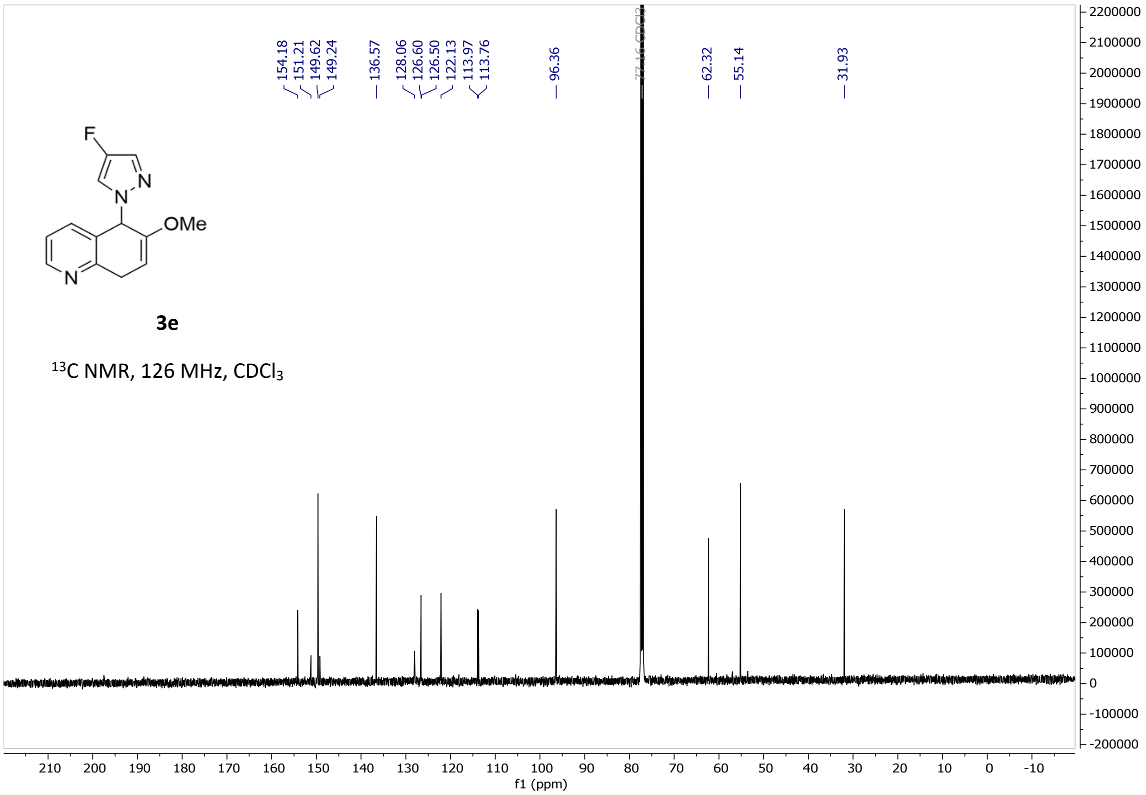

Supplementary Figure 39. ^{13}C NMR spectra of **3e** (126 MHz, rt, $CDCl_3$).

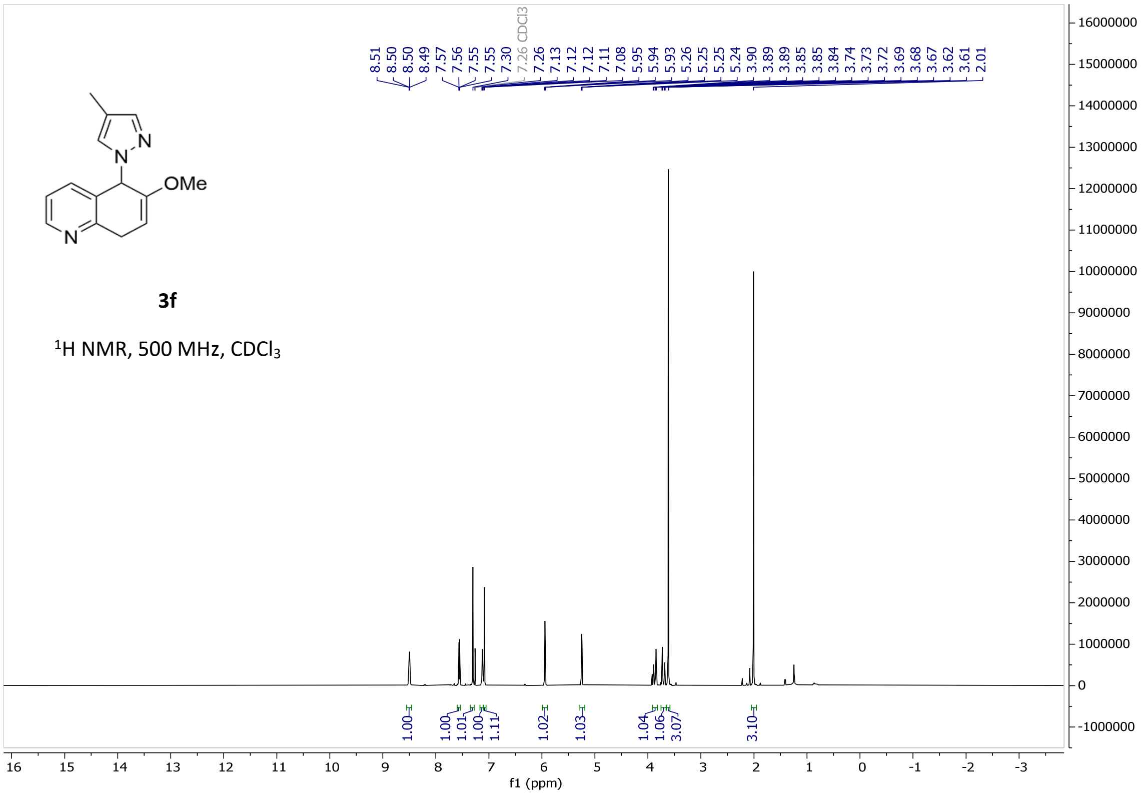

Supplementary Figure 40. ^{1}H NMR spectra of **3f** (500 MHz, rt, $CDCl_3$).

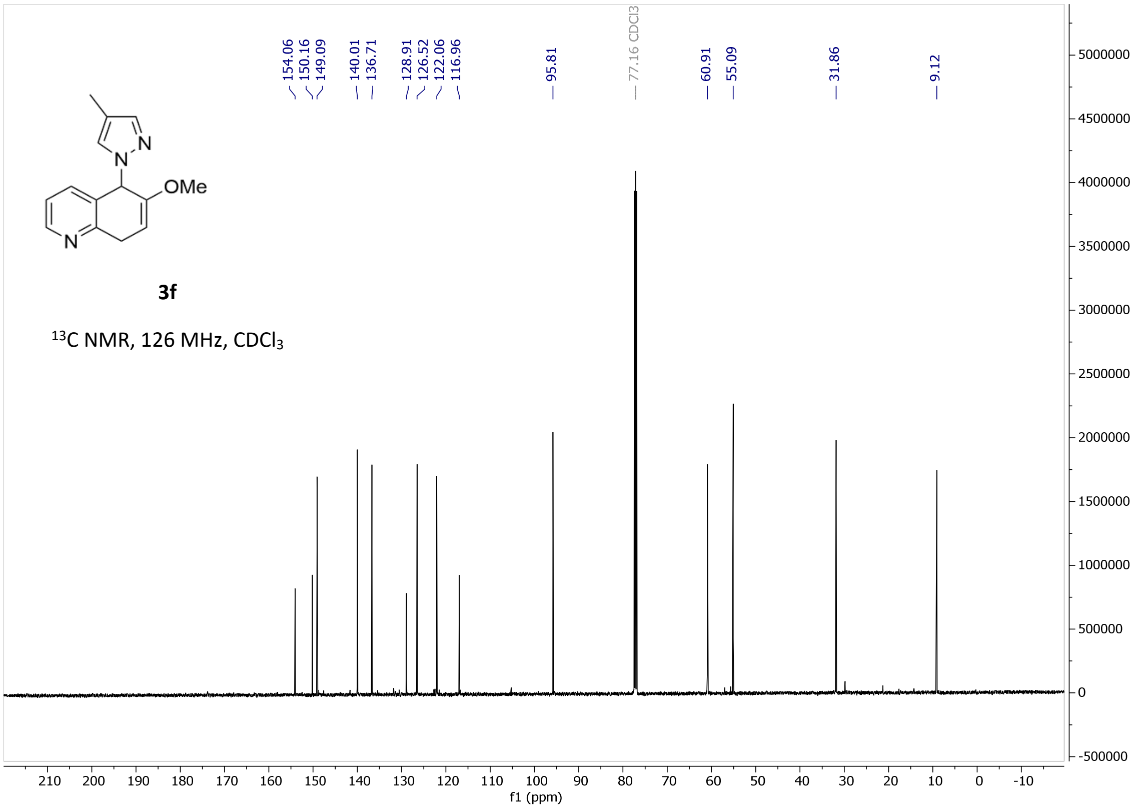

Supplementary Figure 41. ^{13}C NMR spectra of **3f** (126 MHz, rt, $CDCl_3$).

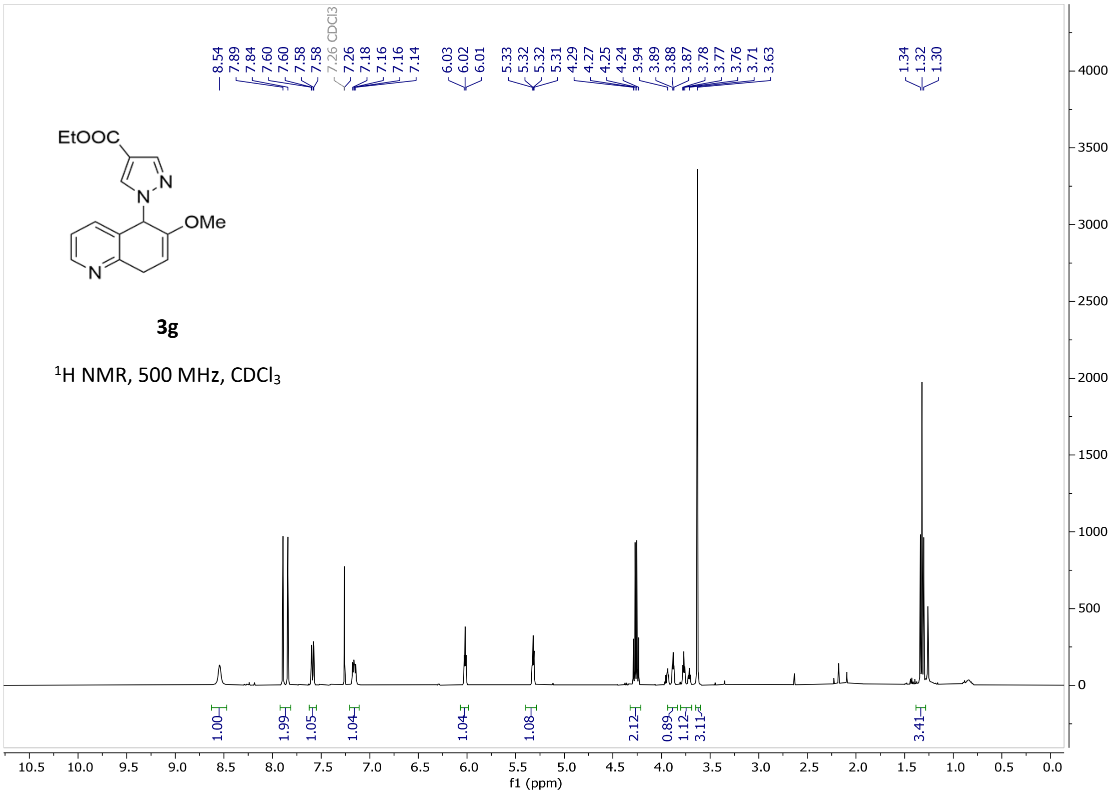

Supplementary Figure 42. ^{1}H NMR spectra of **3g** (500 MHz, rt, $CDCl_3$).

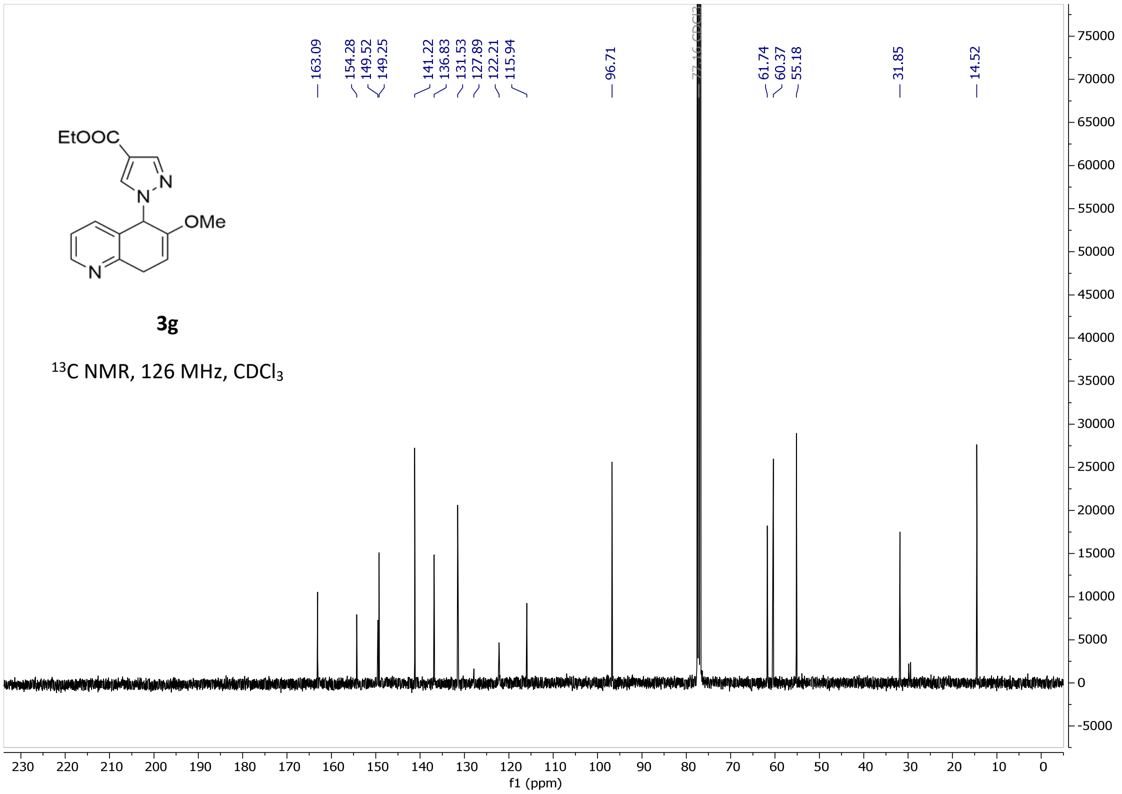

Supplementary Figure 43. ^{13}C NMR spectra of **3g** (126 MHz, rt, $CDCl_3$).

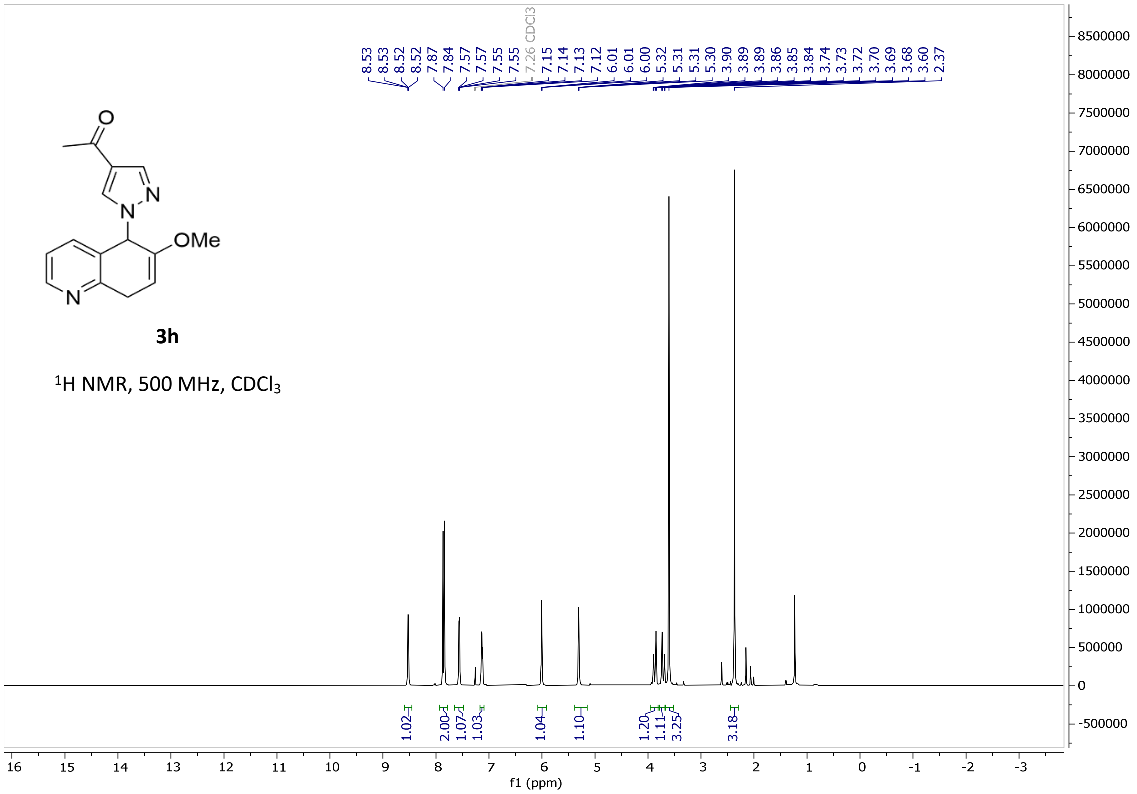

Supplementary Figure 44. ^{1}H NMR spectra of **3h** (500 MHz, rt, $CDCl_3$).

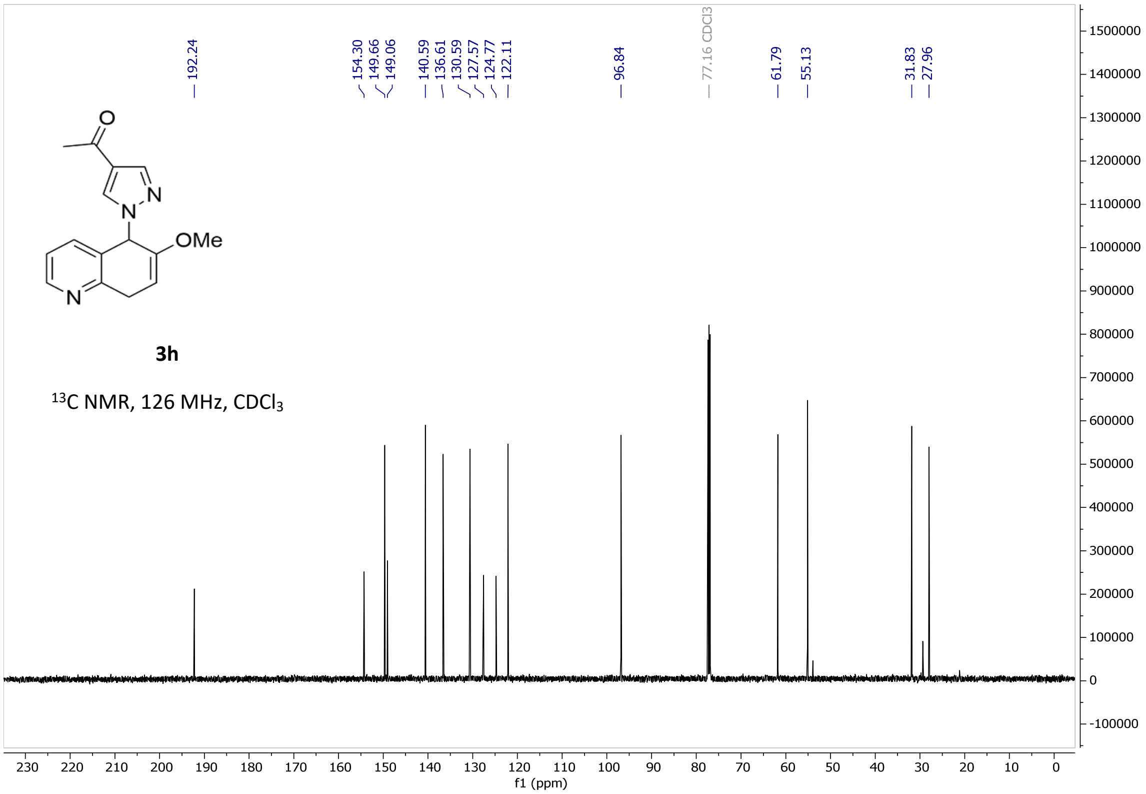

Supplementary Figure 45. ^{13}C NMR spectra of **3h** (126 MHz, rt, $CDCl_3$).

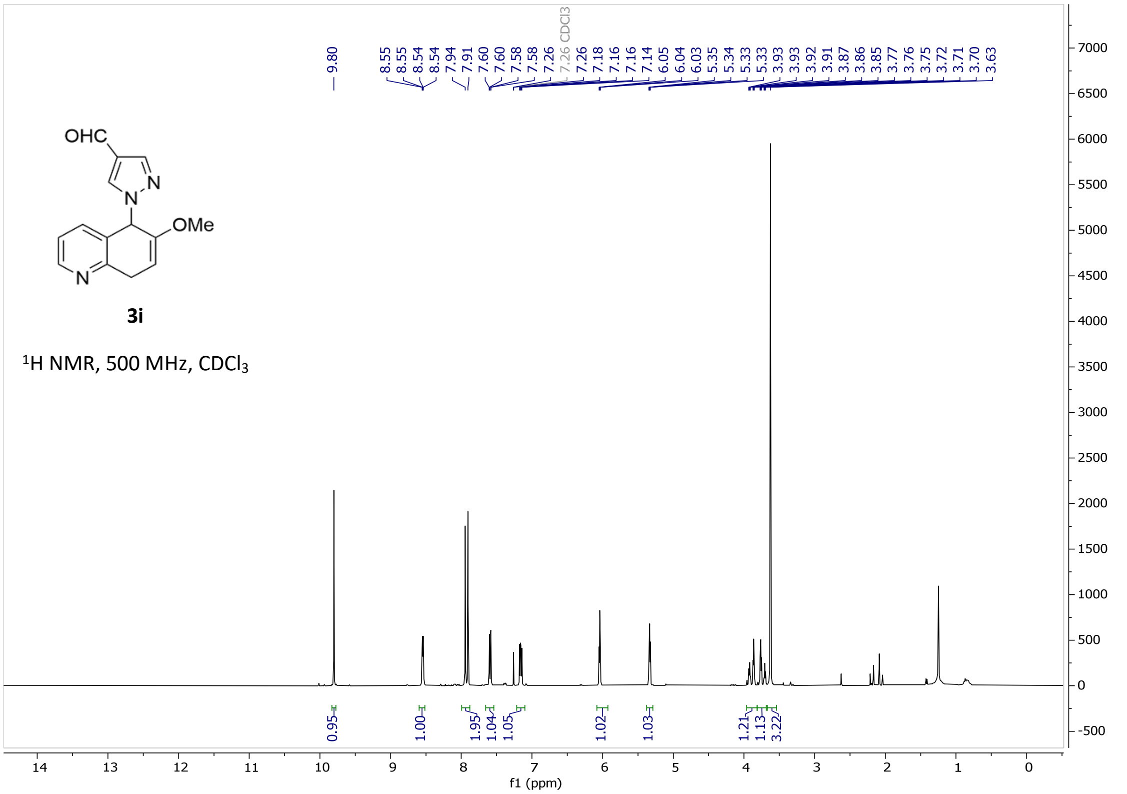

Supplementary Figure 46. ^{1}H NMR spectra of **3i** (500 MHz, rt, $CDCl_3$).

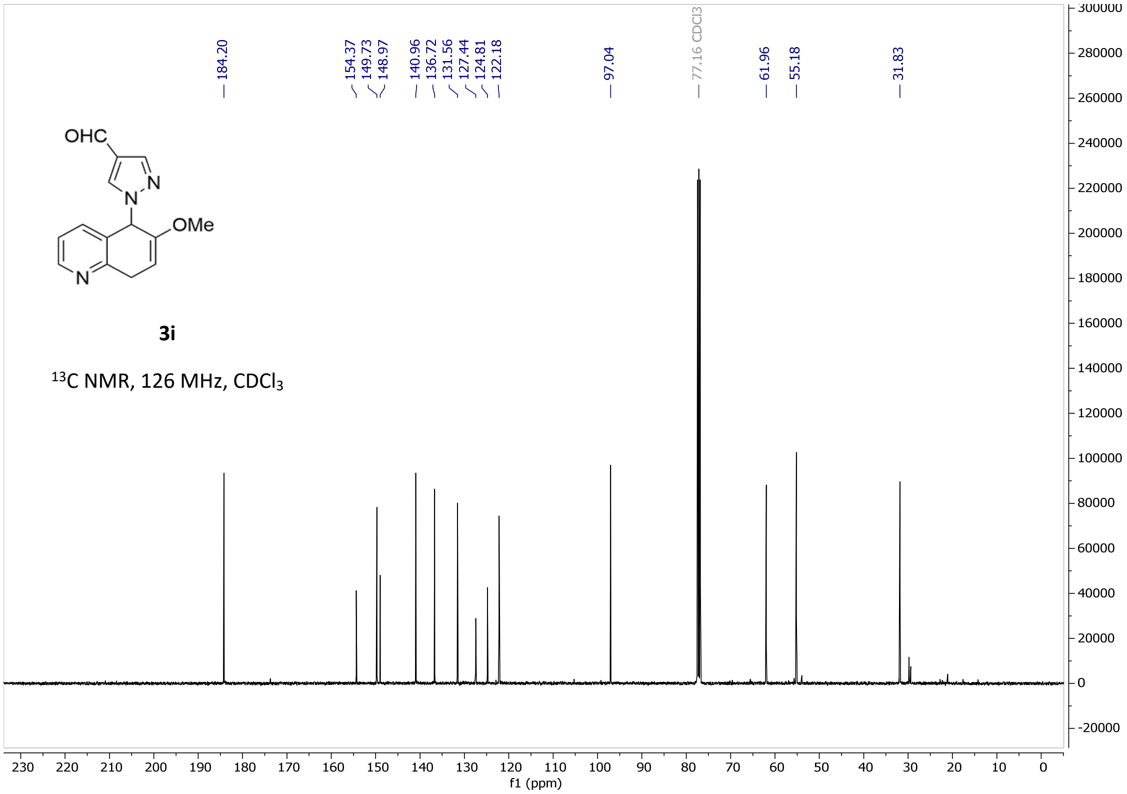

Supplementary Figure 47. ^{13}C NMR spectra of **3i** (126 MHz, rt, $CDCl_3$).

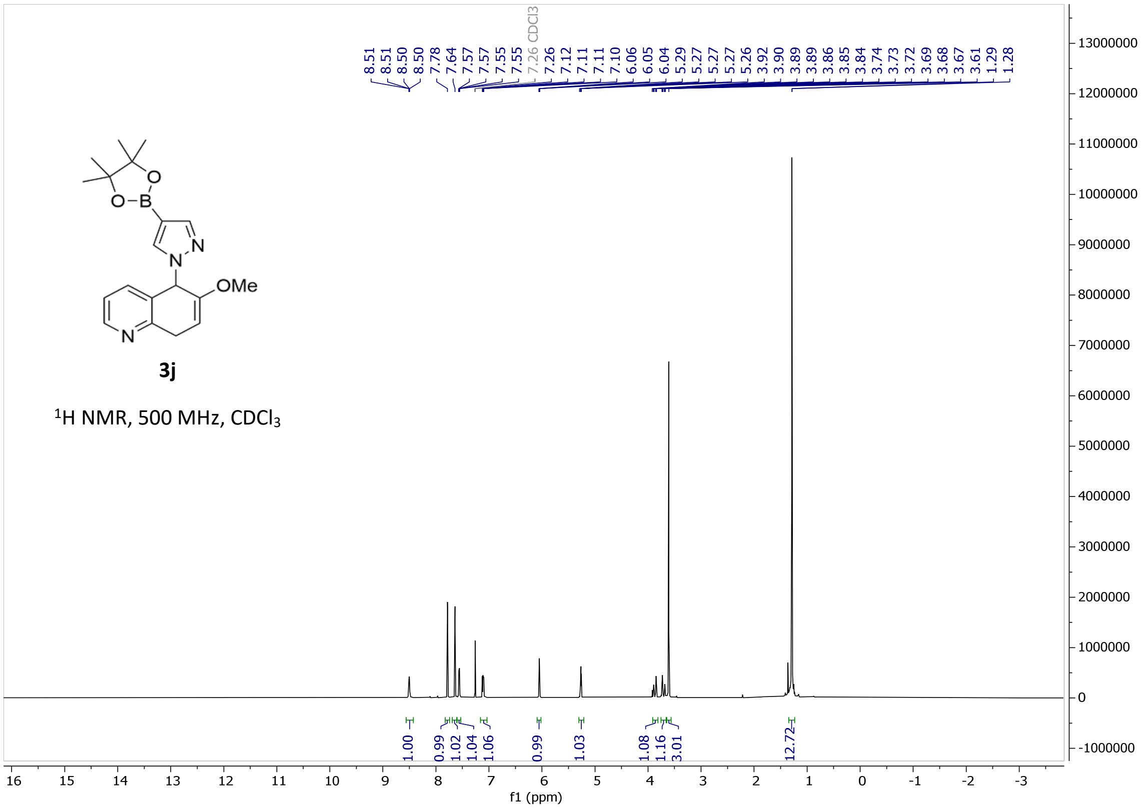

Supplementary Figure 48. ^{1}H NMR spectra of **3j** (500 MHz, rt, $CDCl_3$).

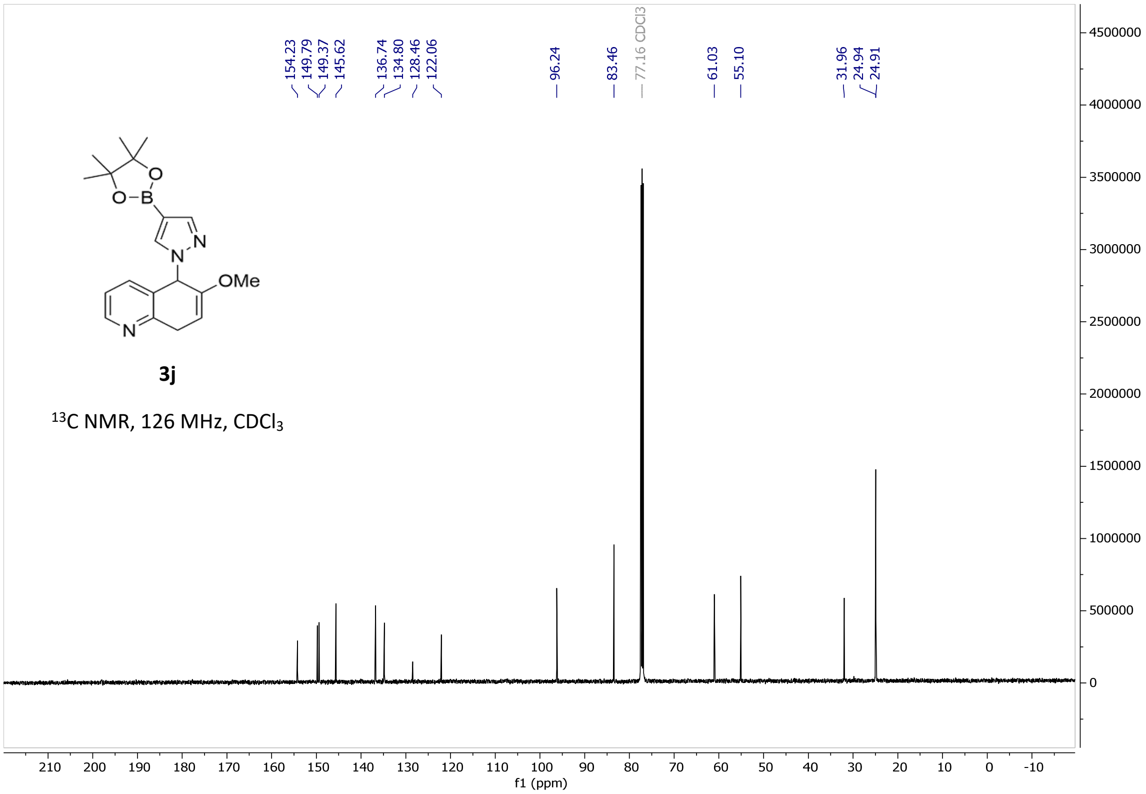

Supplementary Figure 49. ^{13}C NMR spectra of **3j** (126 MHz, rt, $CDCl_3$).

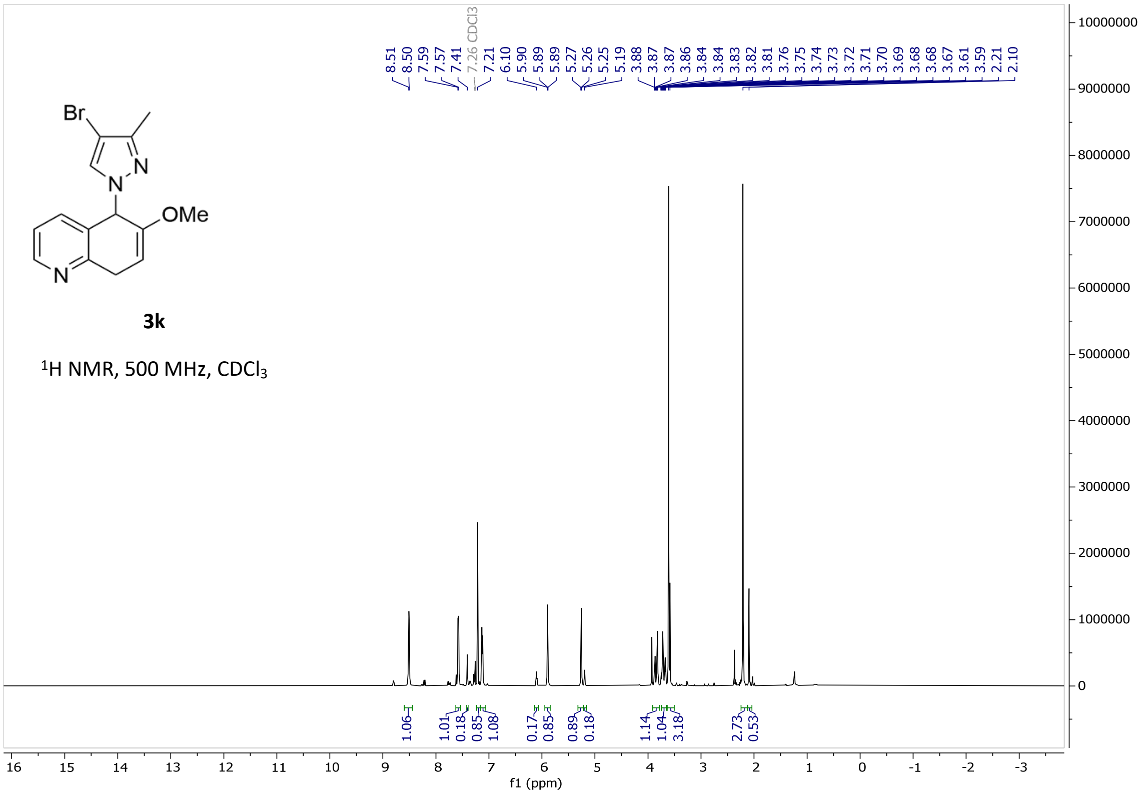

Supplementary Figure 50. ^{1}H NMR spectra of **3k** (500 MHz, rt, $CDCl_3$).

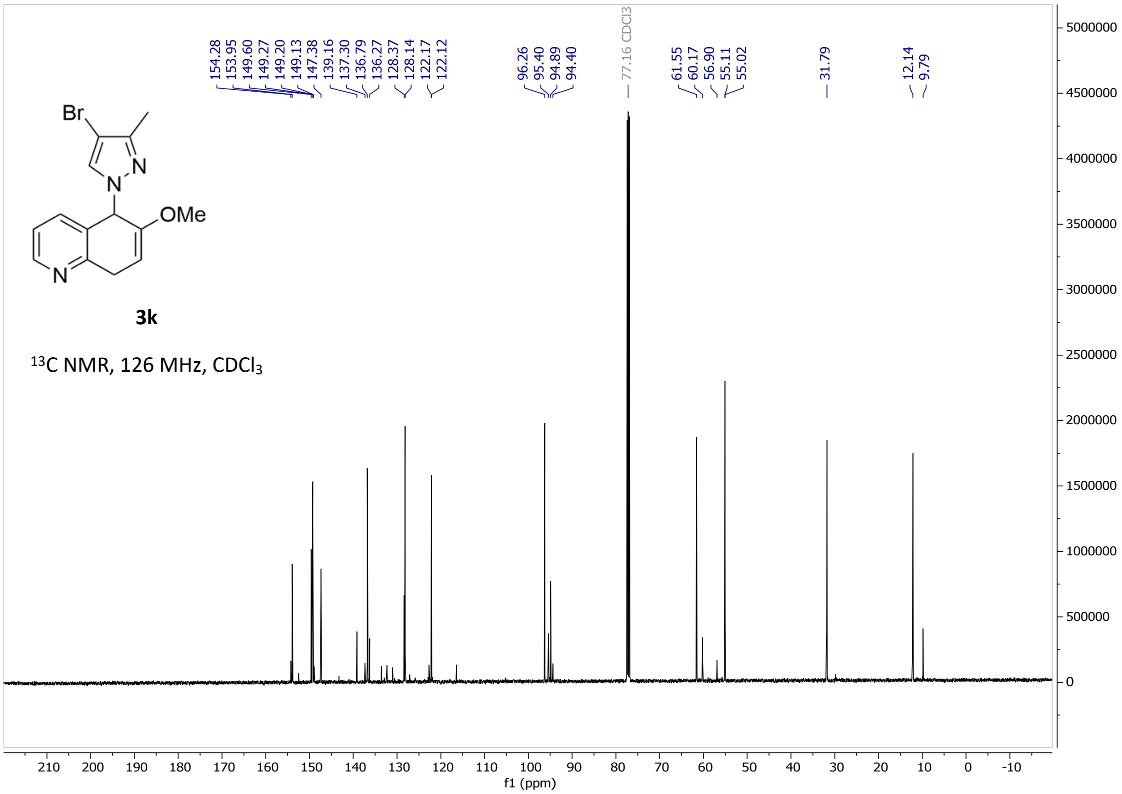

Supplementary Figure 51. ^{13}C NMR spectra of **3k** (126 MHz, rt, $CDCl_3$).

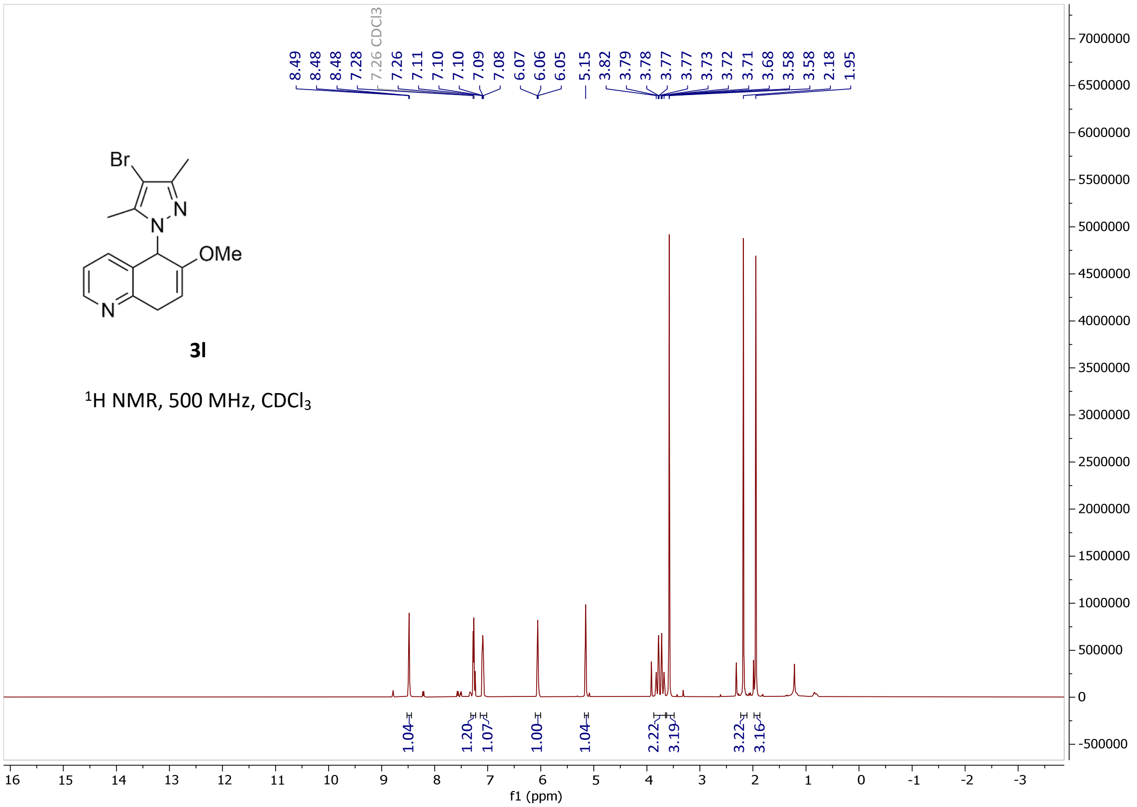

Supplementary Figure 52. ^{1}H NMR spectra of **3l** (500 MHz, rt, $CDCl_3$).

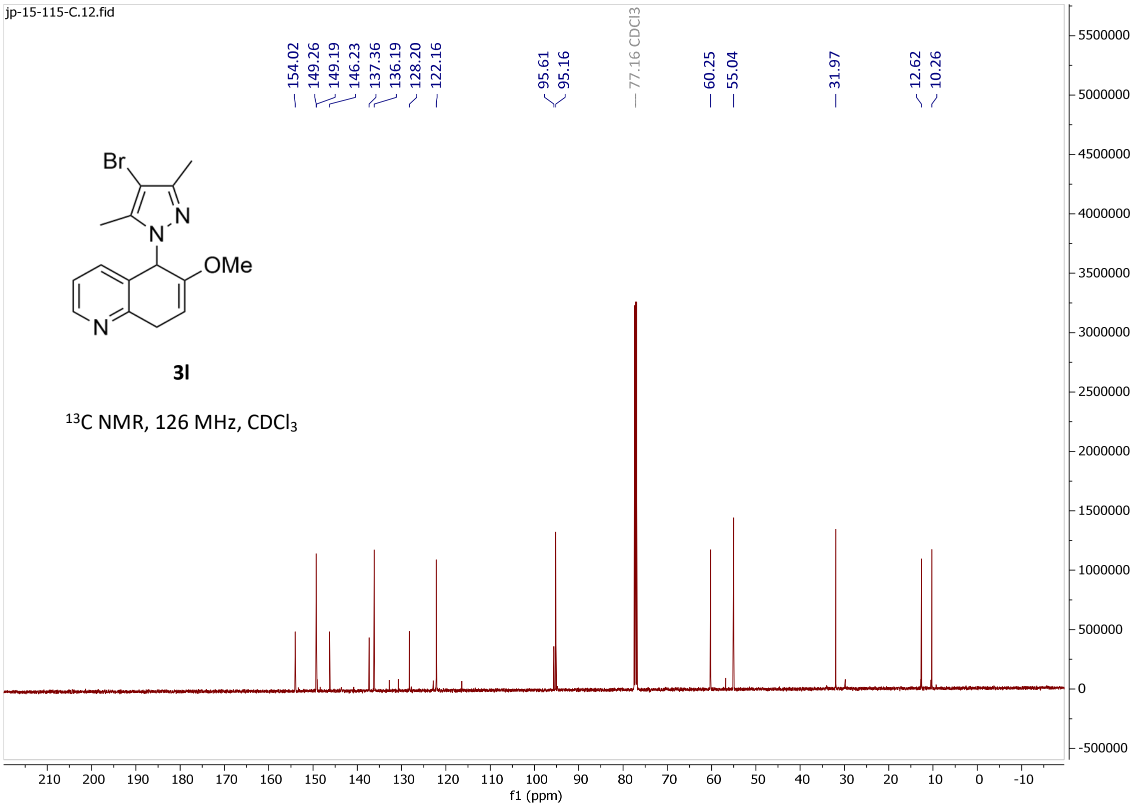

Supplementary Figure 53. ^{13}C NMR spectra of **3l** (126 MHz, rt, $CDCl_3$).

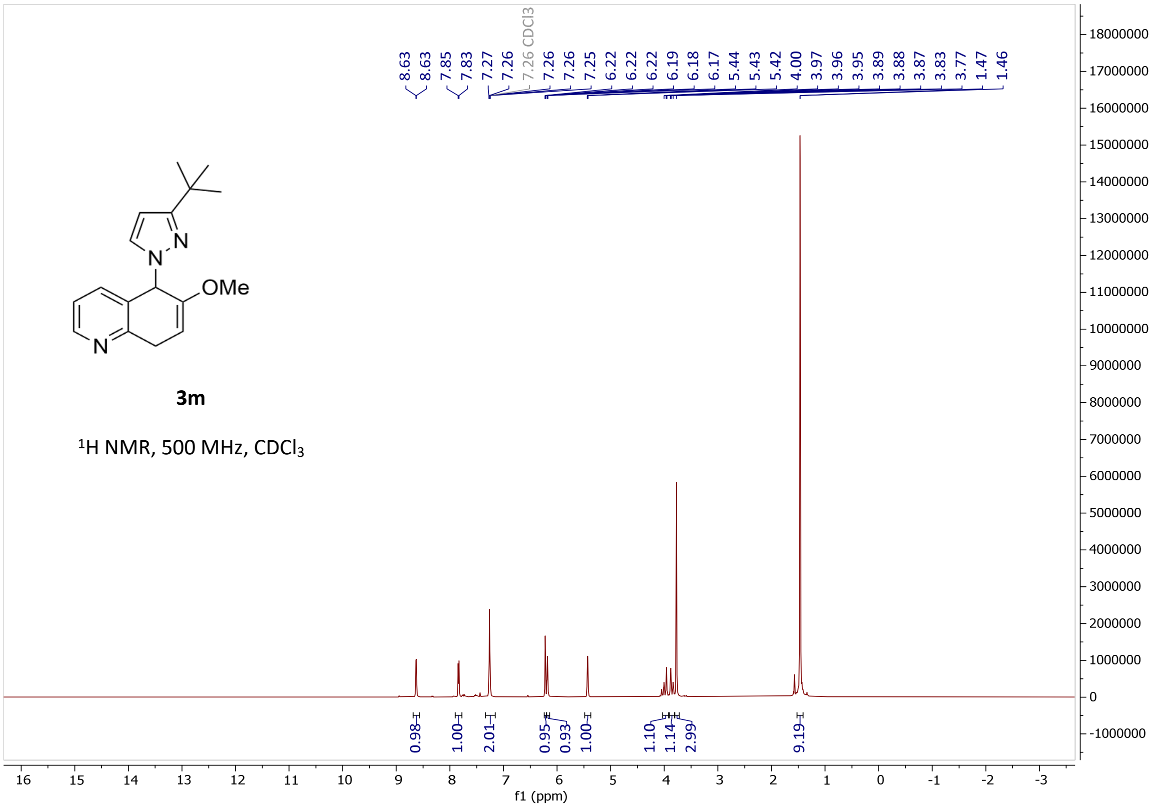

Supplementary Figure 54. ^{1}H NMR spectra of **3m** (500 MHz, rt, $CDCl_3$).

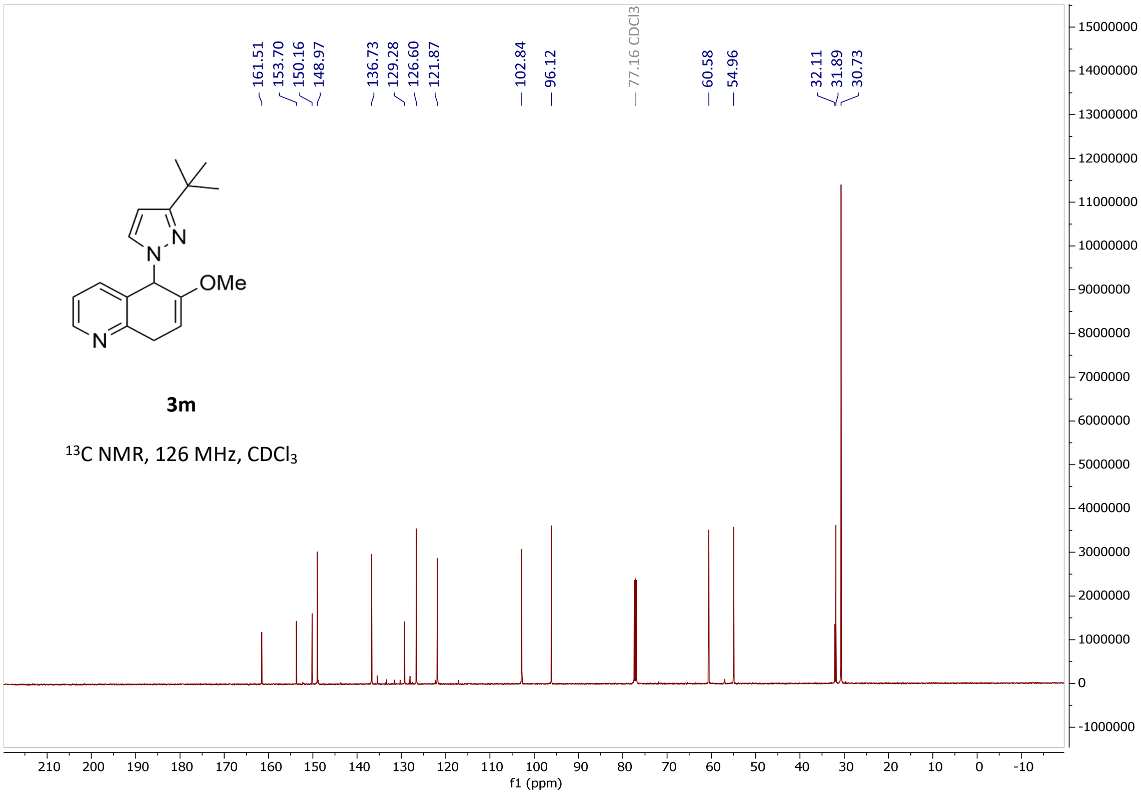

Supplementary Figure 55. ^{13}C NMR spectra of **3m** (126 MHz, rt, $CDCl_3$).

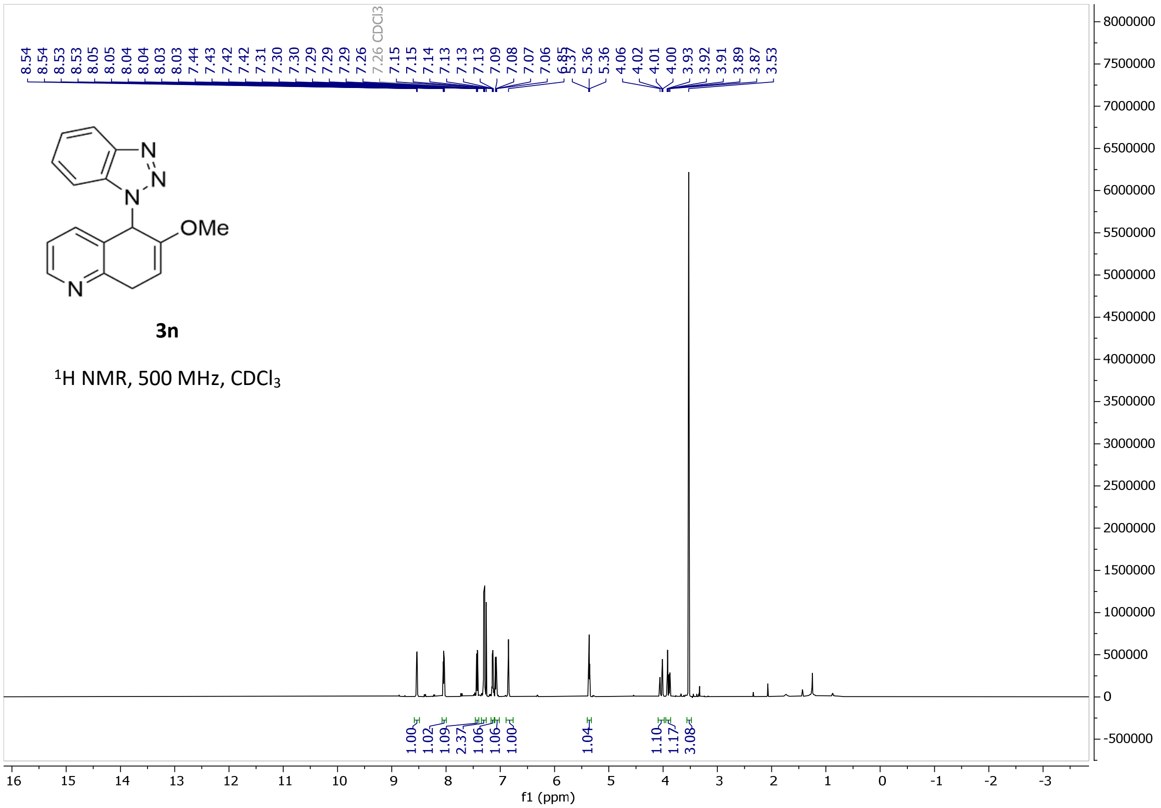

Supplementary Figure 56. ^{1}H NMR spectra of **3n** (500 MHz, rt, $CDCl_3$).

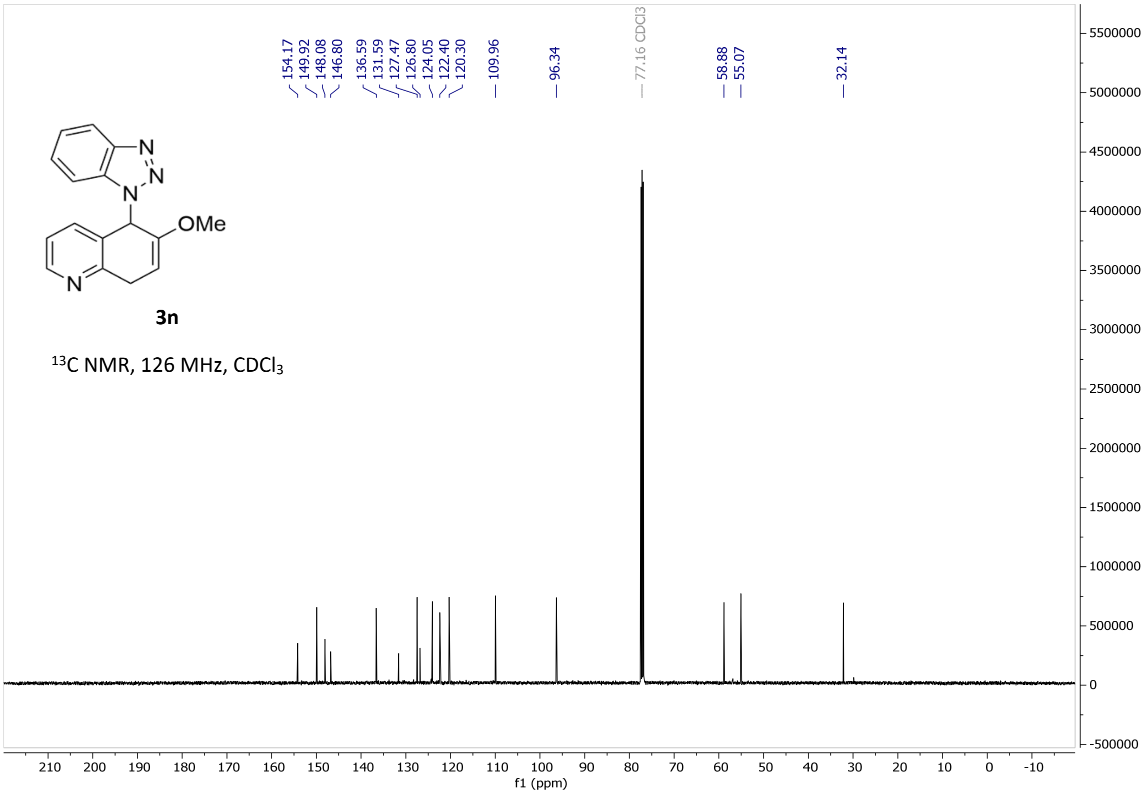

Supplementary Figure 57. ^{13}C NMR spectra of **3n** (126 MHz, rt, $CDCl_3$).

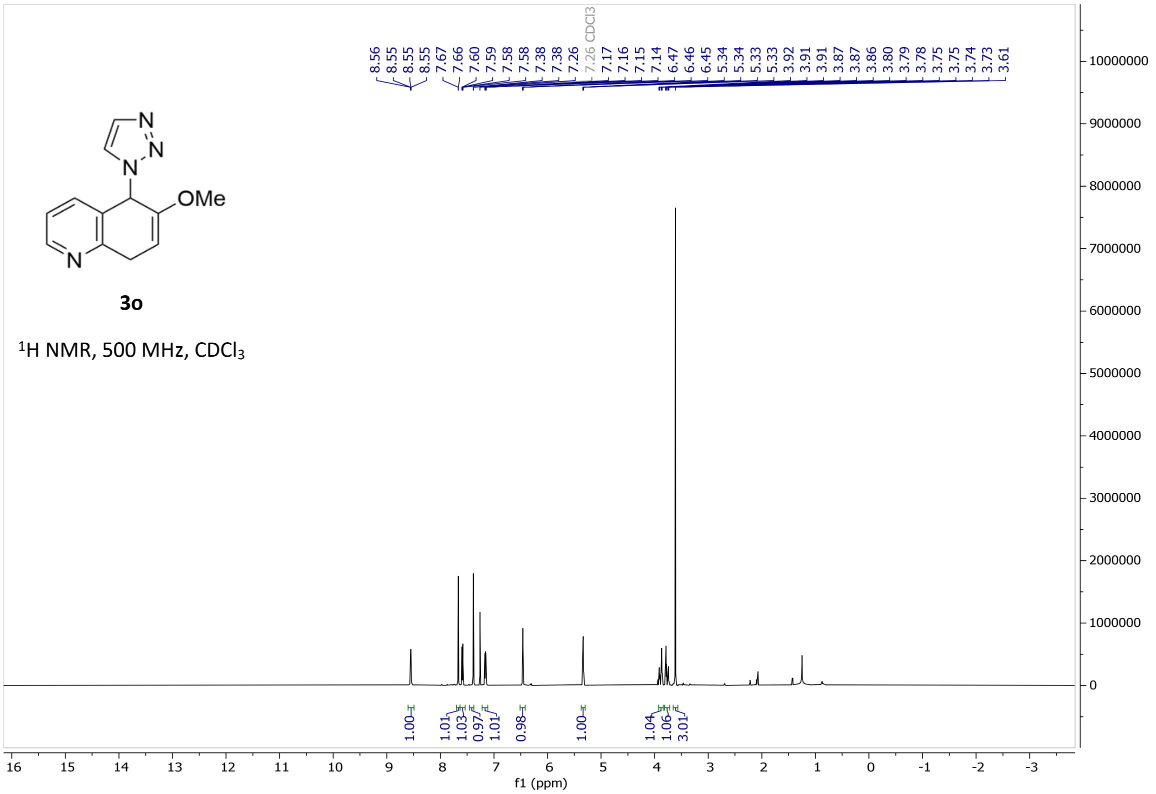

Supplementary Figure 58. ^{1}H NMR spectra of **3o** (500 MHz, rt, $CDCl_3$).

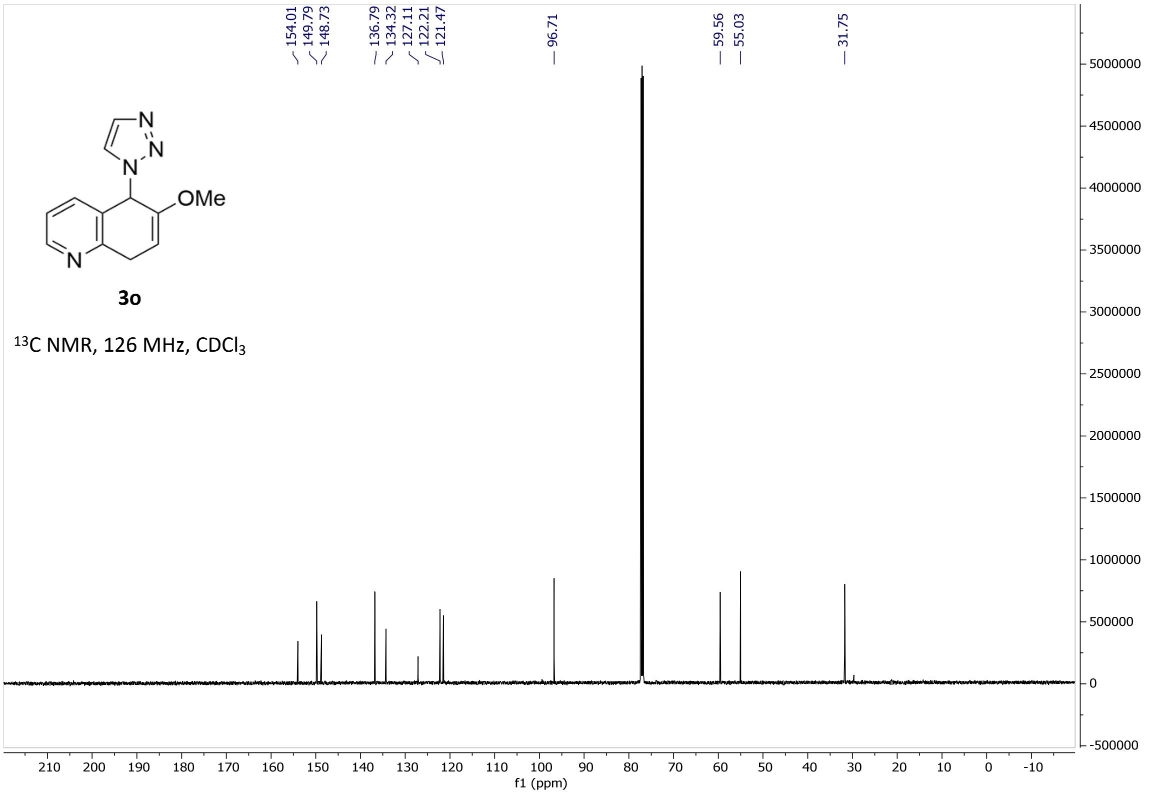

Supplementary Figure 59. ^{13}C NMR spectra of **3o** (126 MHz, rt, $CDCl_3$).

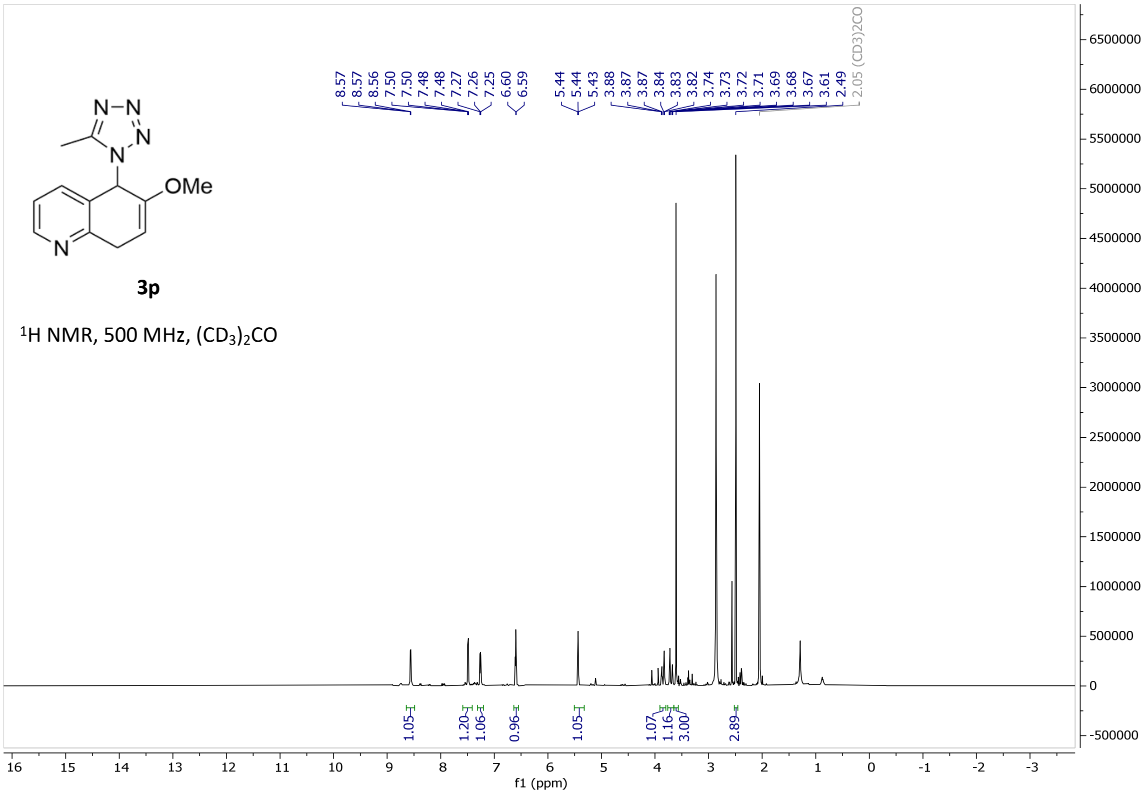

Supplementary Figure 60. ^{1}H NMR spectra of **3p** (500 MHz, rt, $(CD_3)_2CO$).

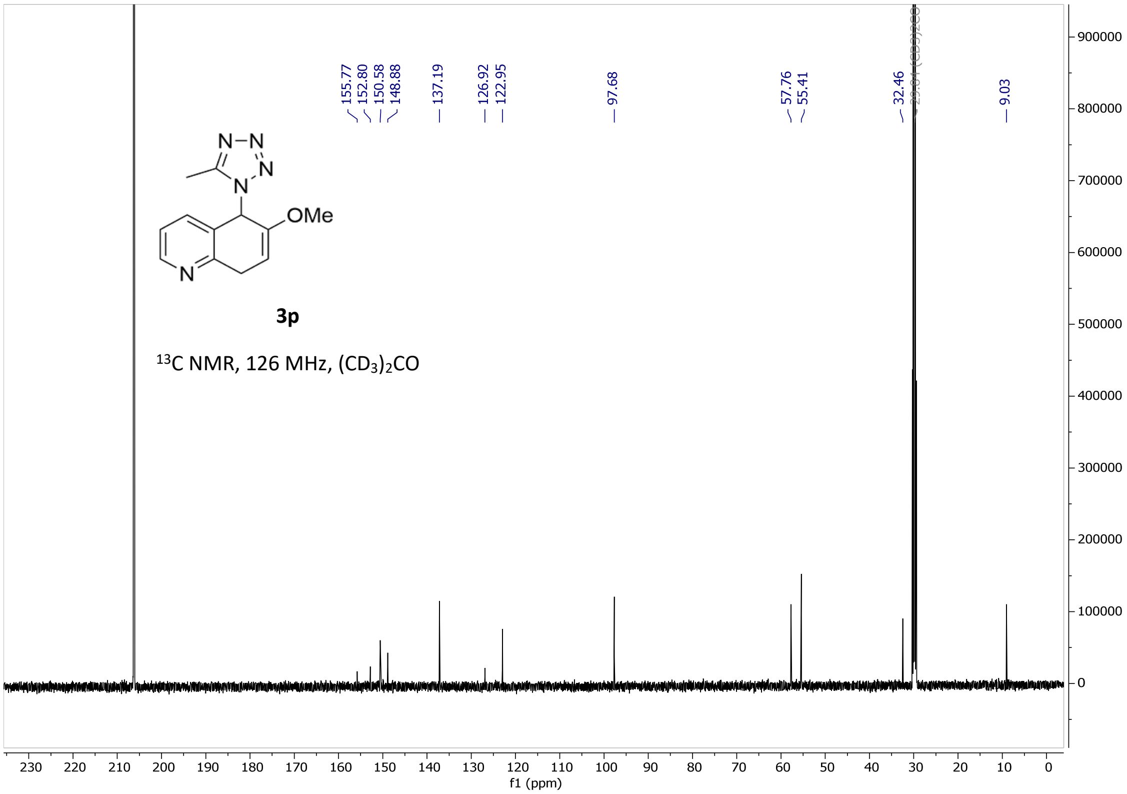

Supplementary Figure 61. ^{13}C NMR spectra of **3p** (126 MHz, rt, $(CD_3)_2CO$).

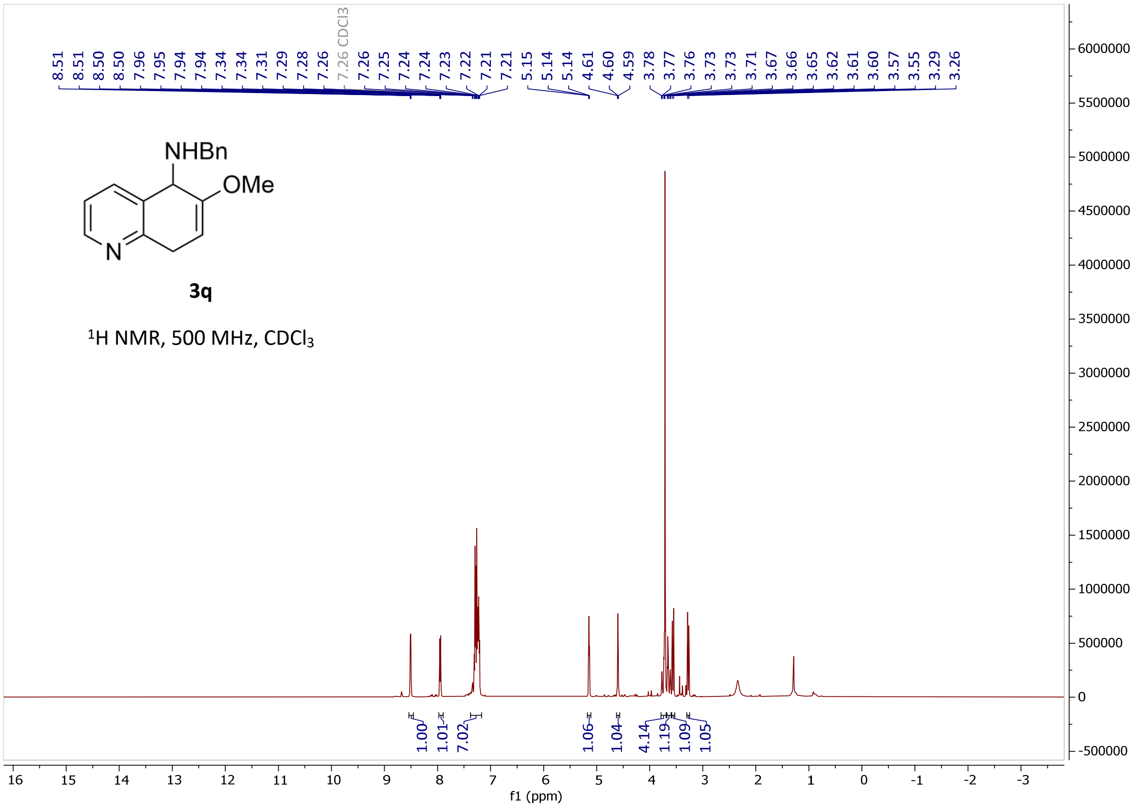

Supplementary Figure 62. ^{1}H NMR spectra of **3q** (500 MHz, rt, CDCl$_3$).

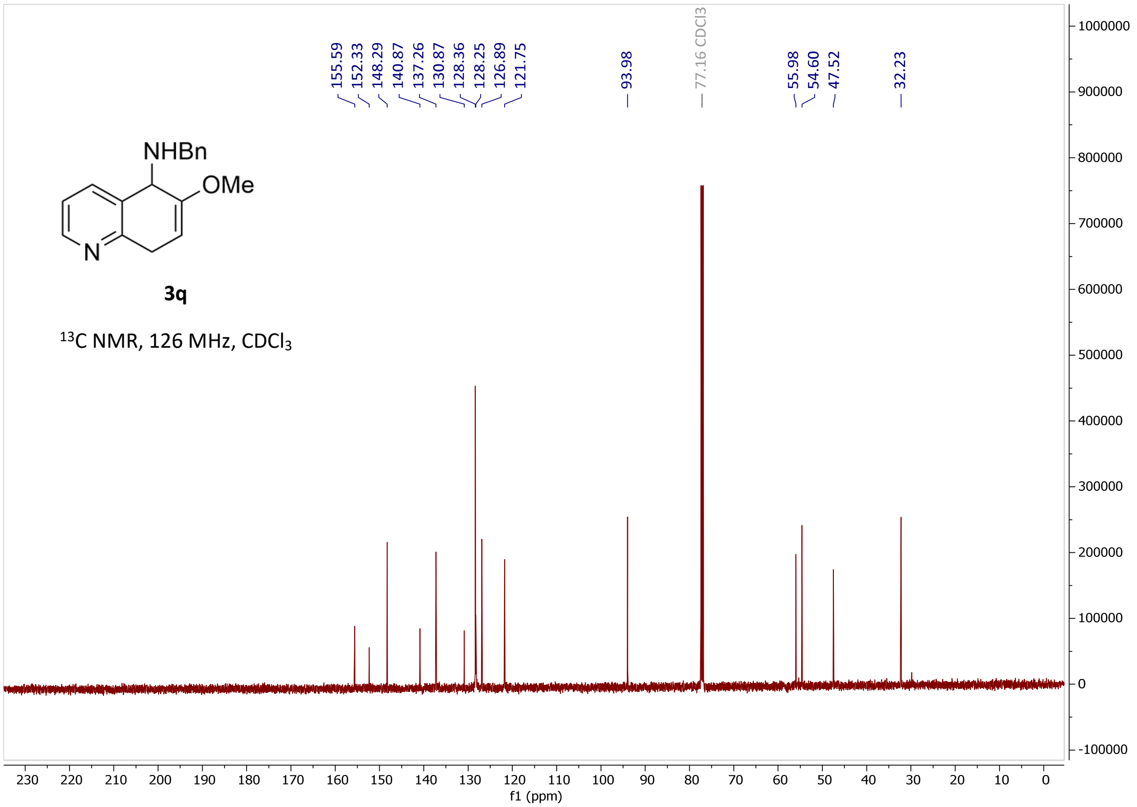

Supplementary Figure 63. ^{13}C NMR spectra of **3q** (126 MHz, rt, CDCl$_3$).

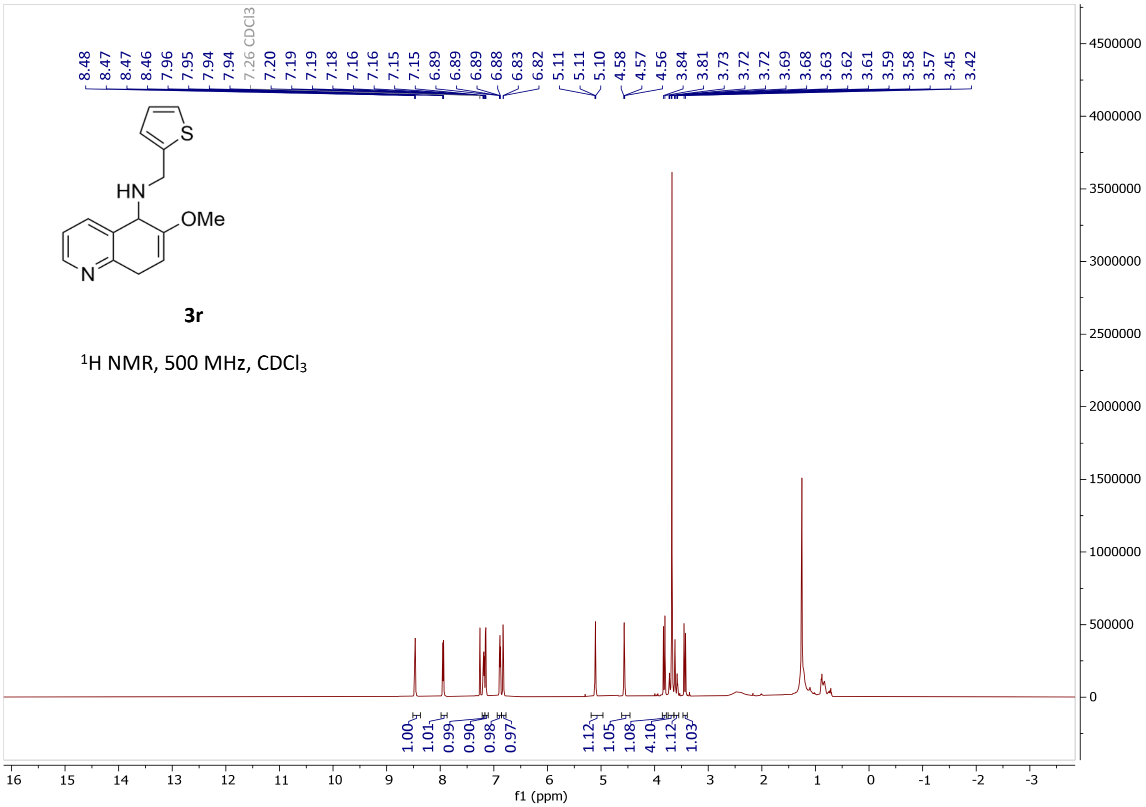

Supplementary Figure 64. ^{1}H NMR spectra of **3r** (500 MHz, rt, $CDCl_3$).

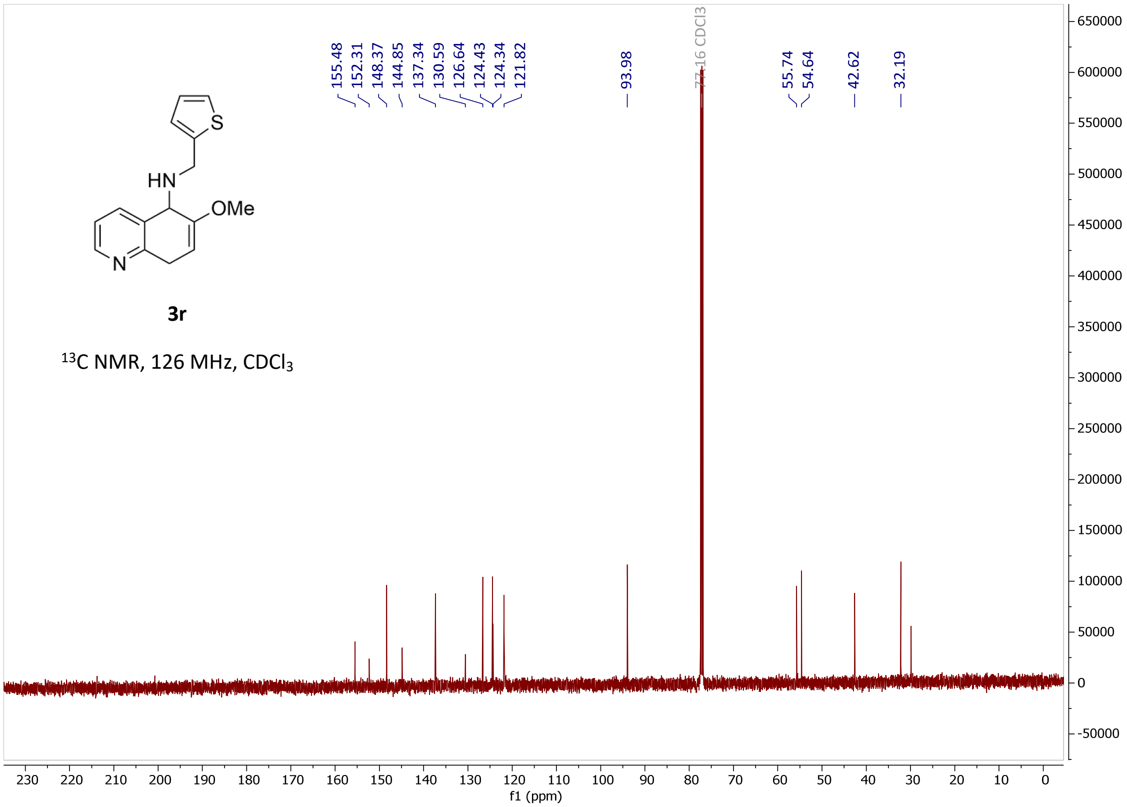

Supplementary Figure 65. ^{13}C NMR spectra of **3r** (126 MHz, rt, $CDCl_3$).

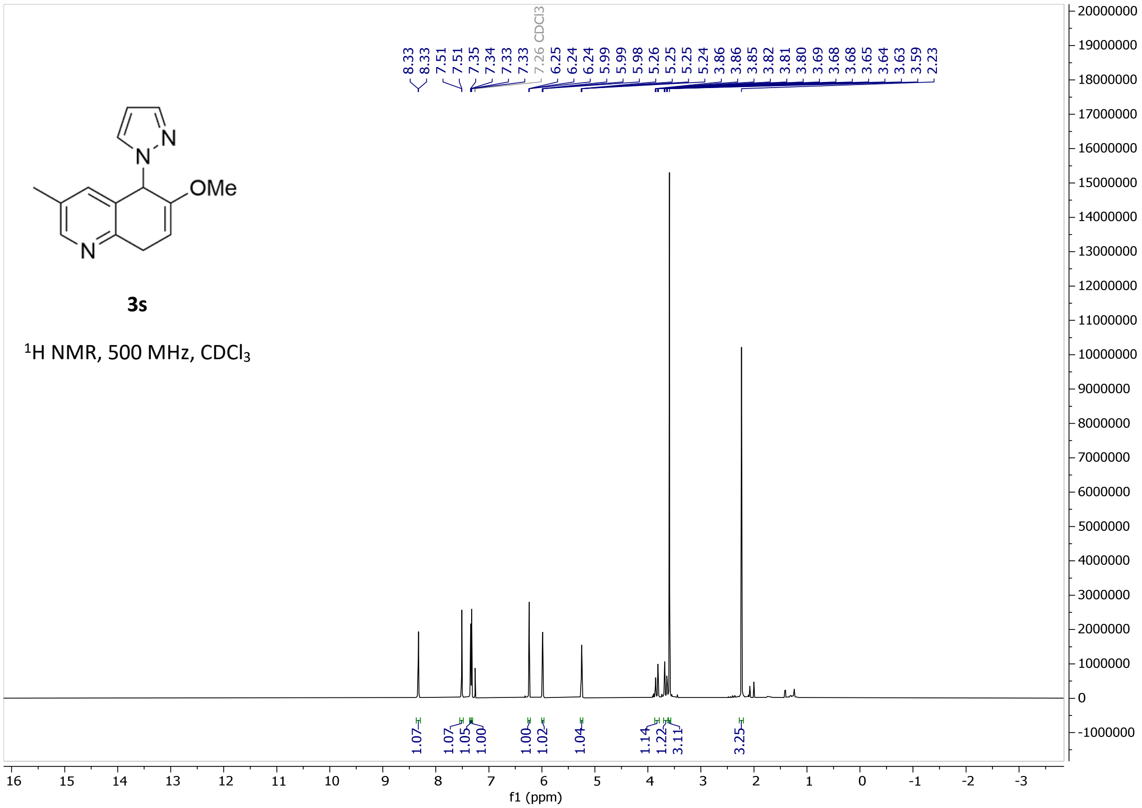

Supplementary Figure 66. ^{1}H NMR spectra of **3s** (500 MHz, rt, $CDCl_3$).

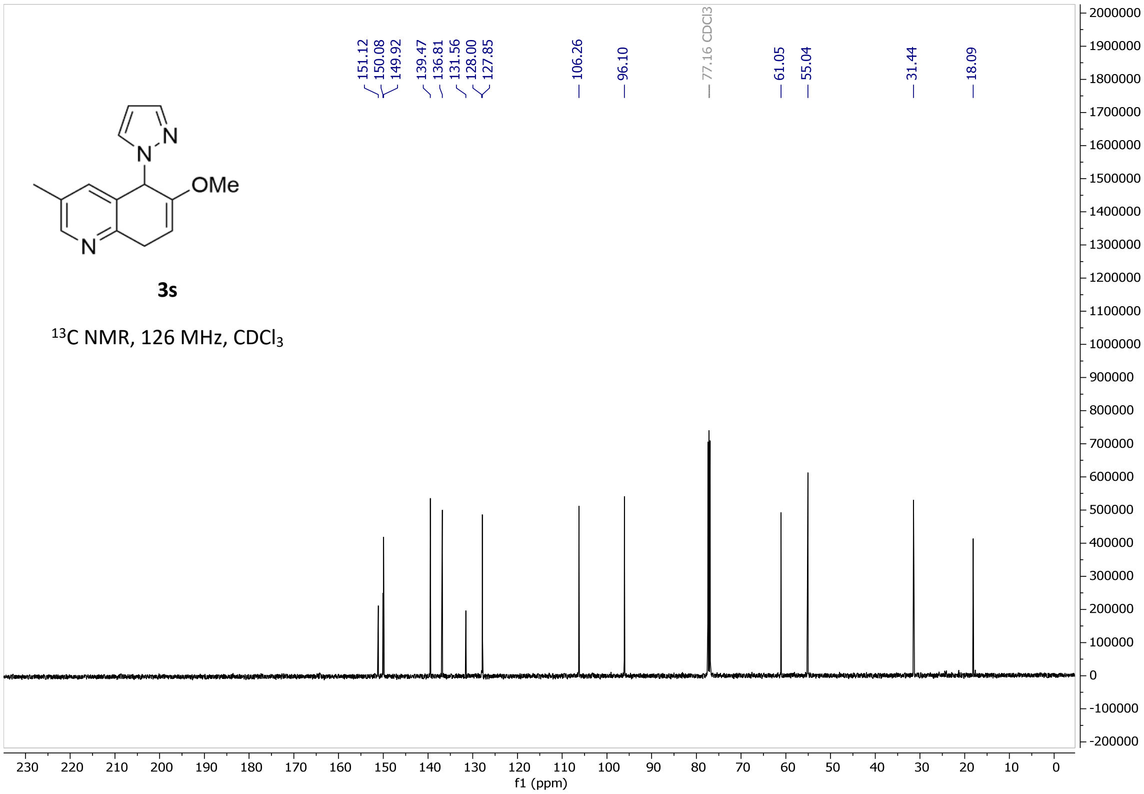

Supplementary Figure 67. ^{13}C NMR spectra of **3s** (126 MHz, rt, $CDCl_3$).

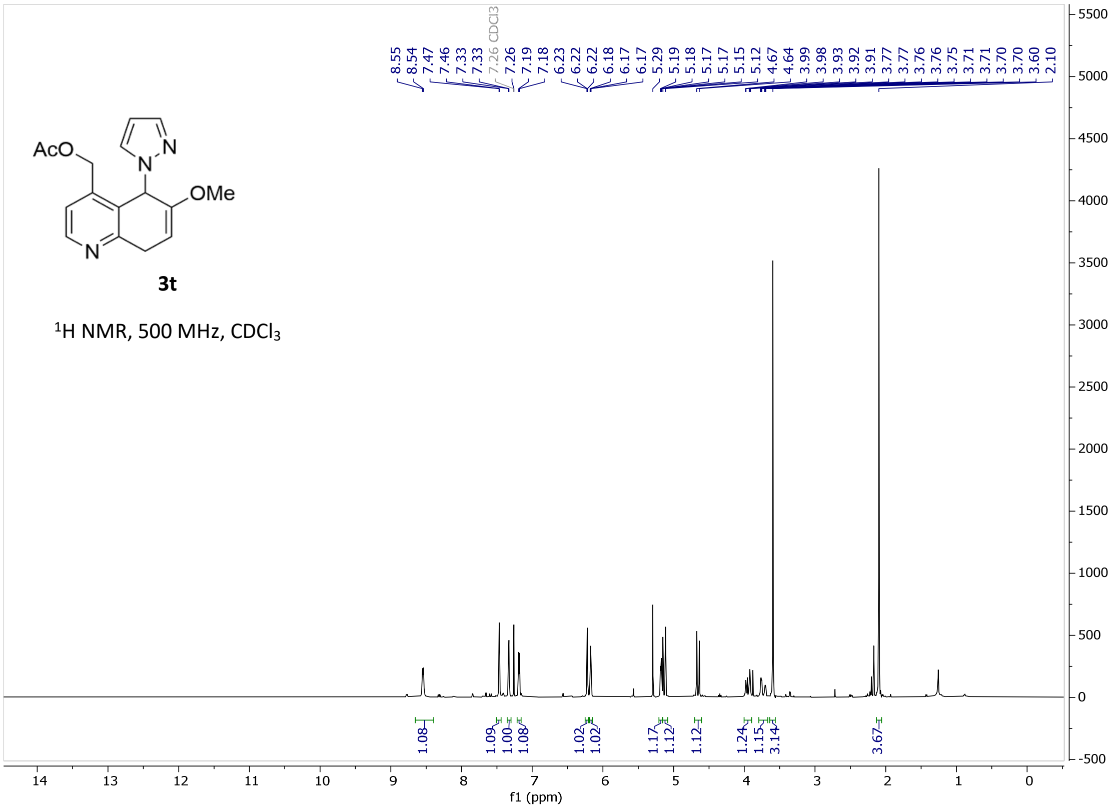

Supplementary Figure 68. ^{1}H NMR spectra of **3t** (500 MHz, rt, $CDCl_3$).

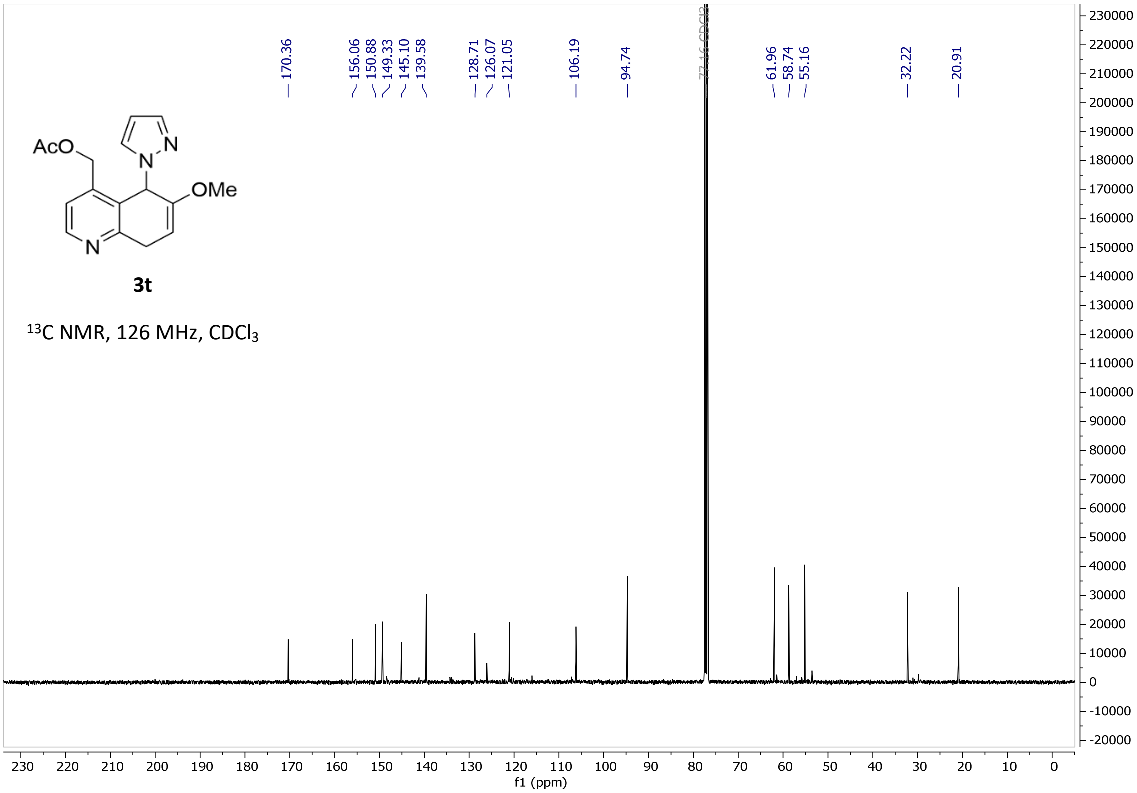

Supplementary Figure 69. ^{13}C NMR spectra of **3t** (126 MHz, rt, $CDCl_3$).

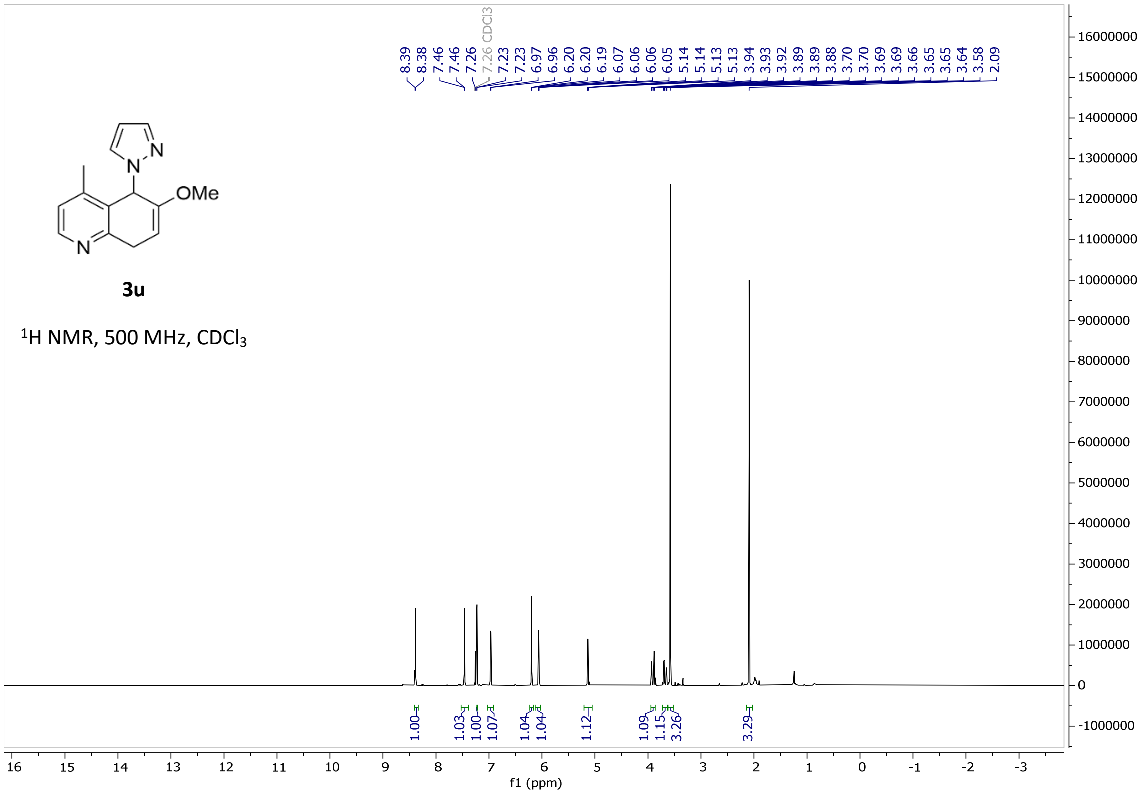

Supplementary Figure 70. ^{1}H NMR spectra of **3u** (500 MHz, rt, $CDCl_3$).

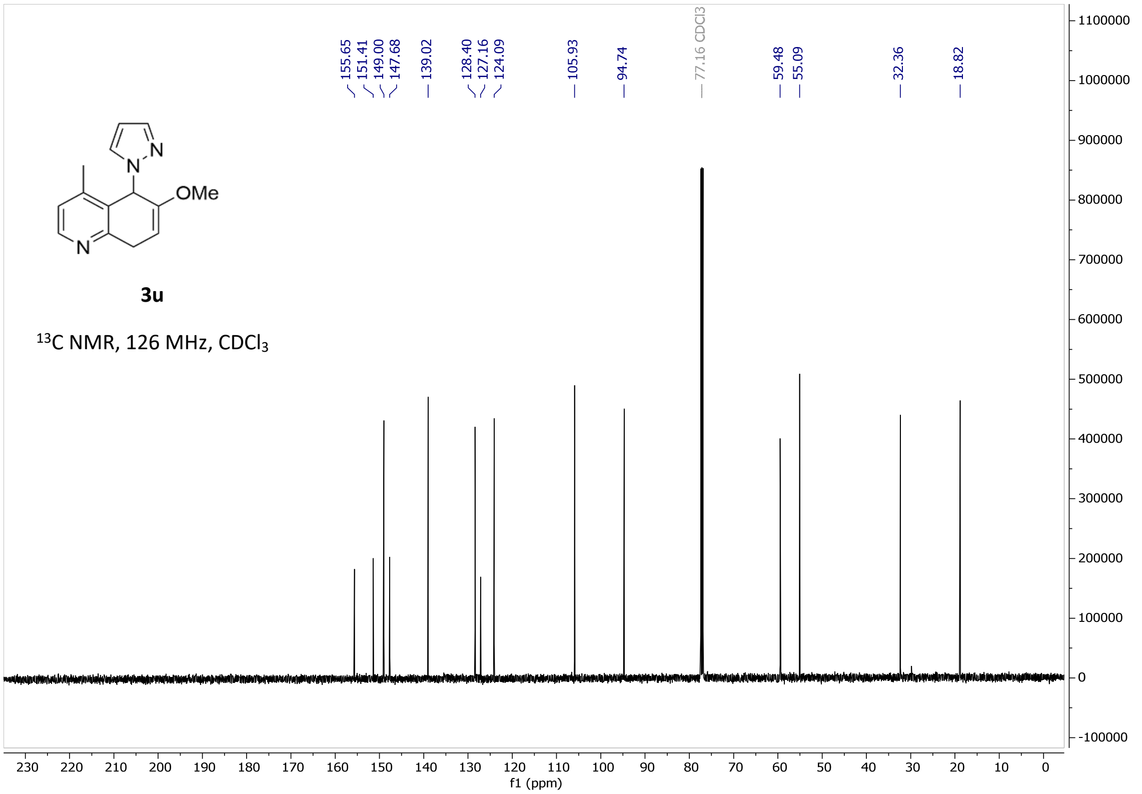

Supplementary Figure 71. ^{13}C NMR spectra of **3u** (126 MHz, rt, $CDCl_3$).

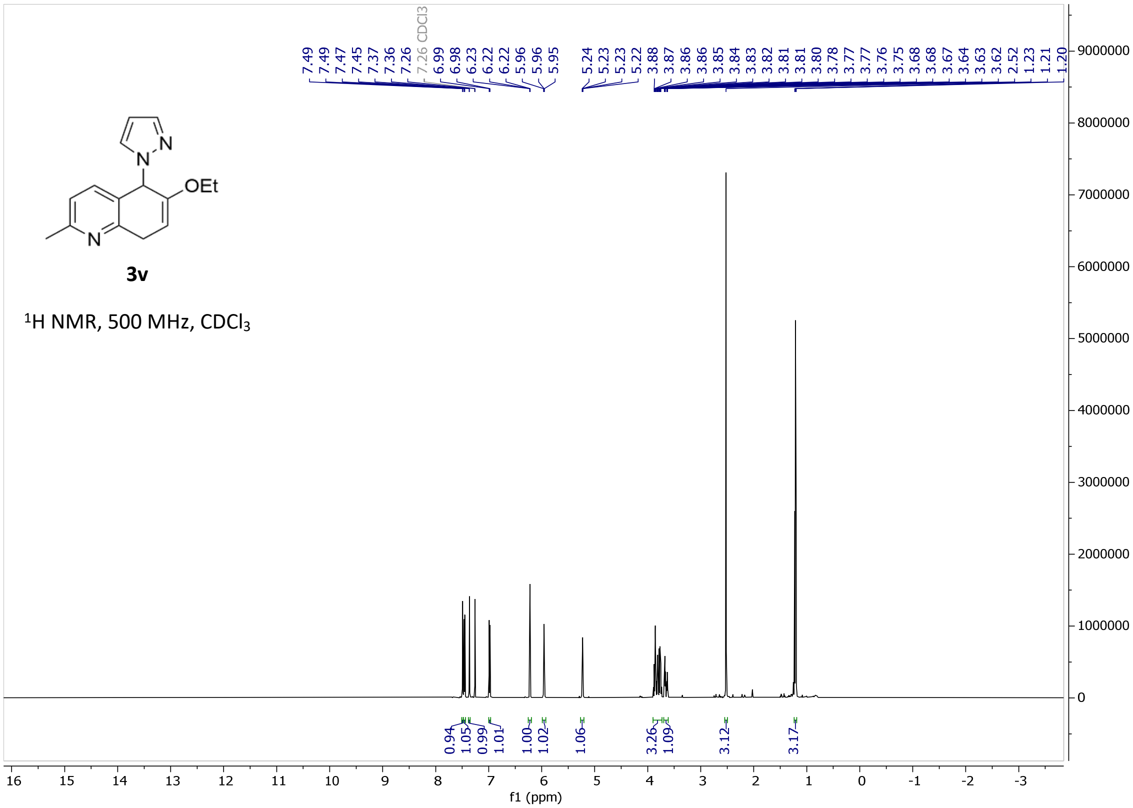

Supplementary Figure 72. ^{1}H NMR spectra of **3v** (500 MHz, rt, $CDCl_3$).

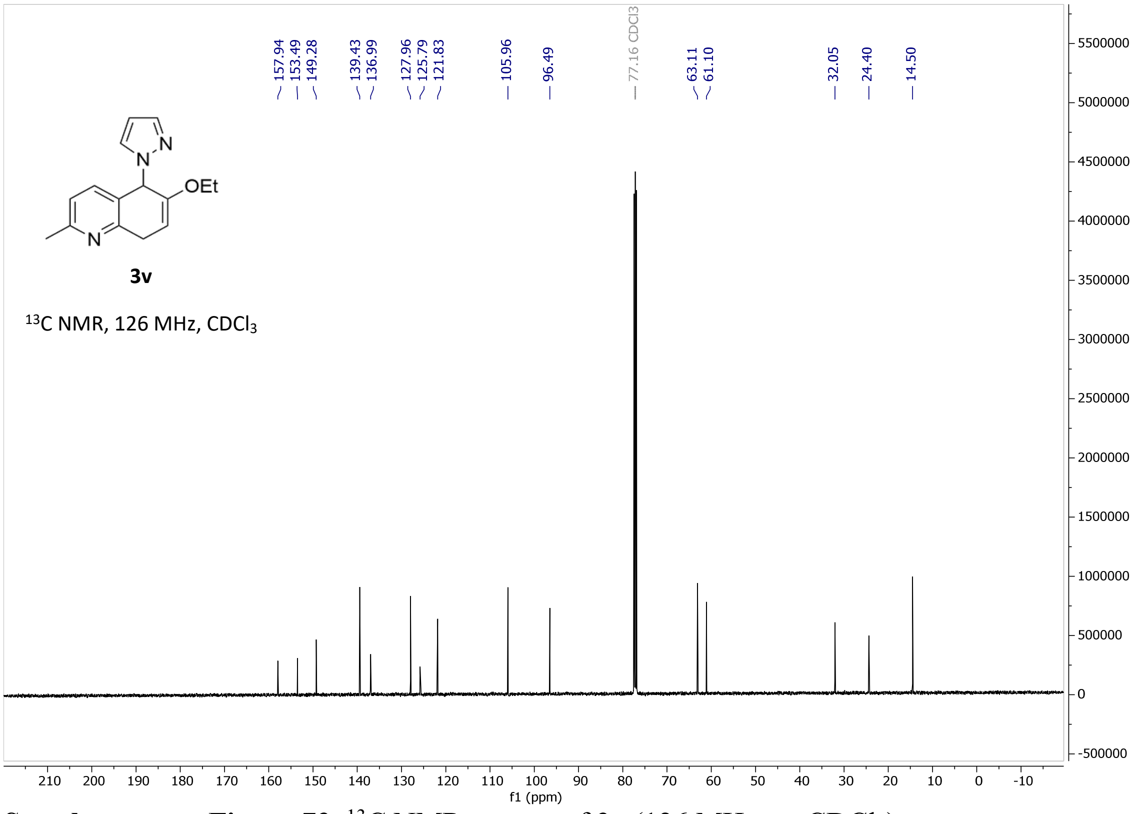

Supplementary Figure 73. ^{13}C NMR spectra of **3v** (126 MHz, rt, $CDCl_3$).

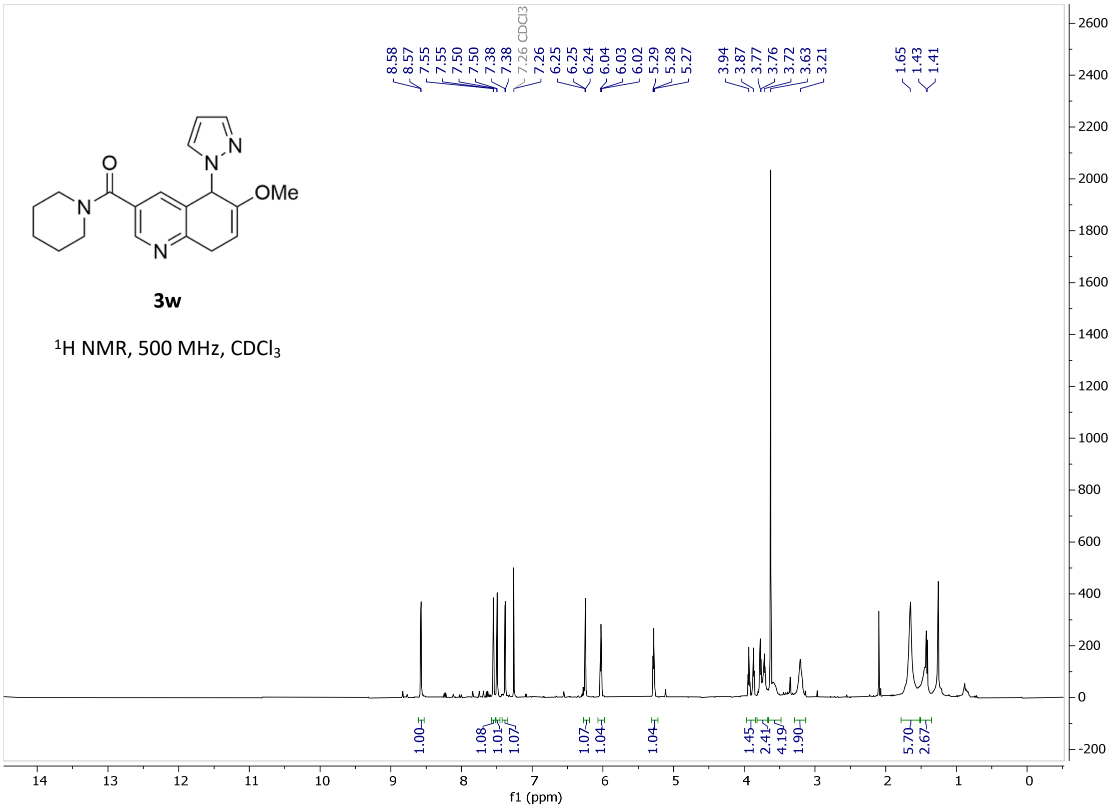

Supplementary Figure 74. ^{1}H NMR spectra of **3w** (500 MHz, rt, $CDCl_3$).

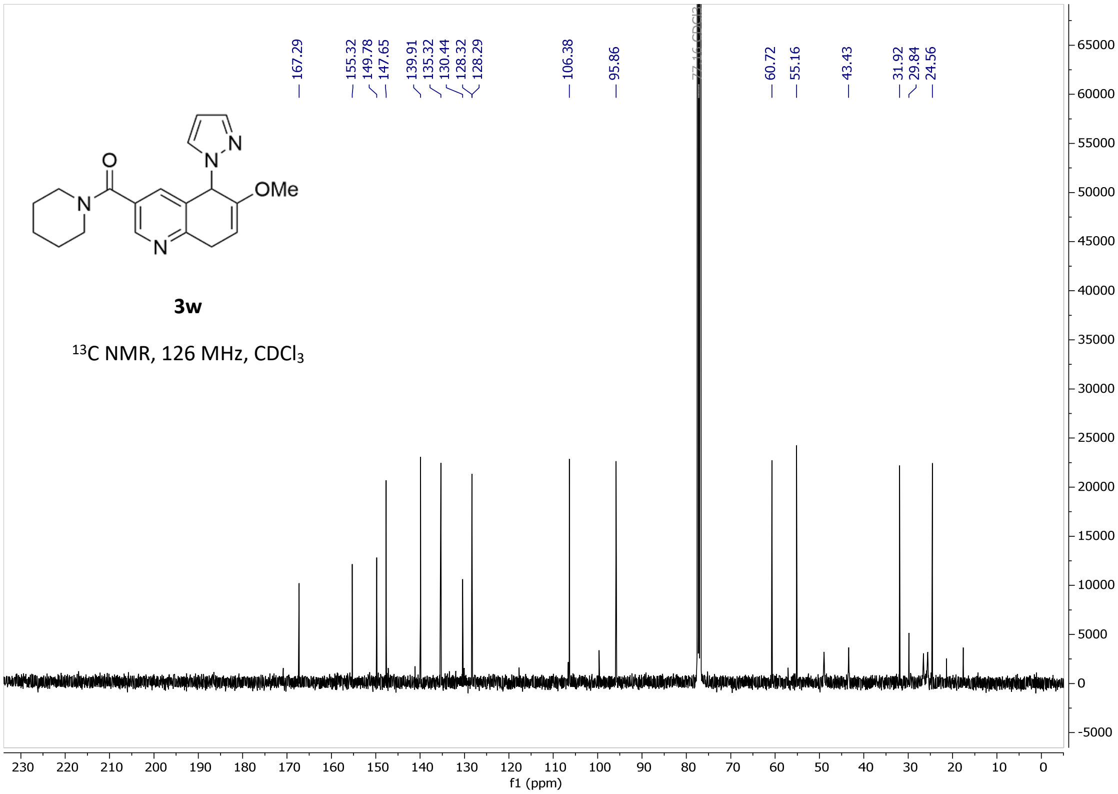

Supplementary Figure 75. ^{13}C NMR spectra of **3w** (126 MHz, rt, $CDCl_3$).

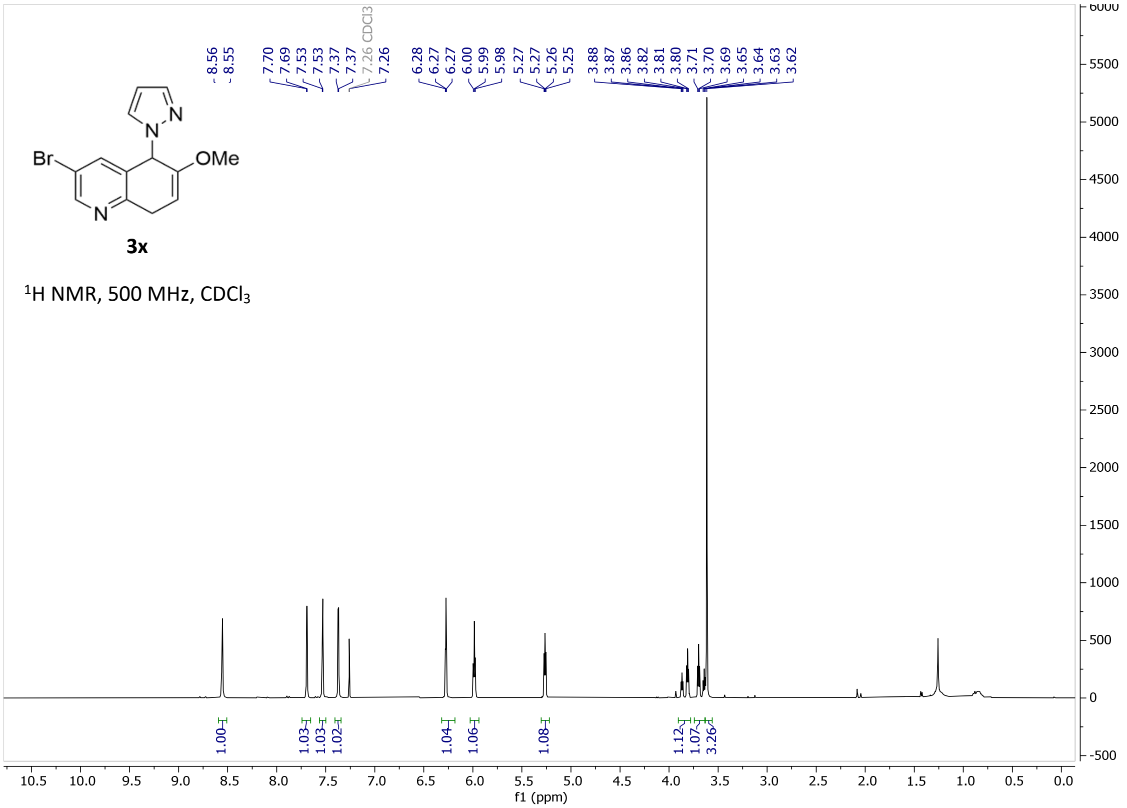

Supplementary Figure 76. ^{1}H NMR spectra of **3x** (500 MHz, rt, $CDCl_3$).

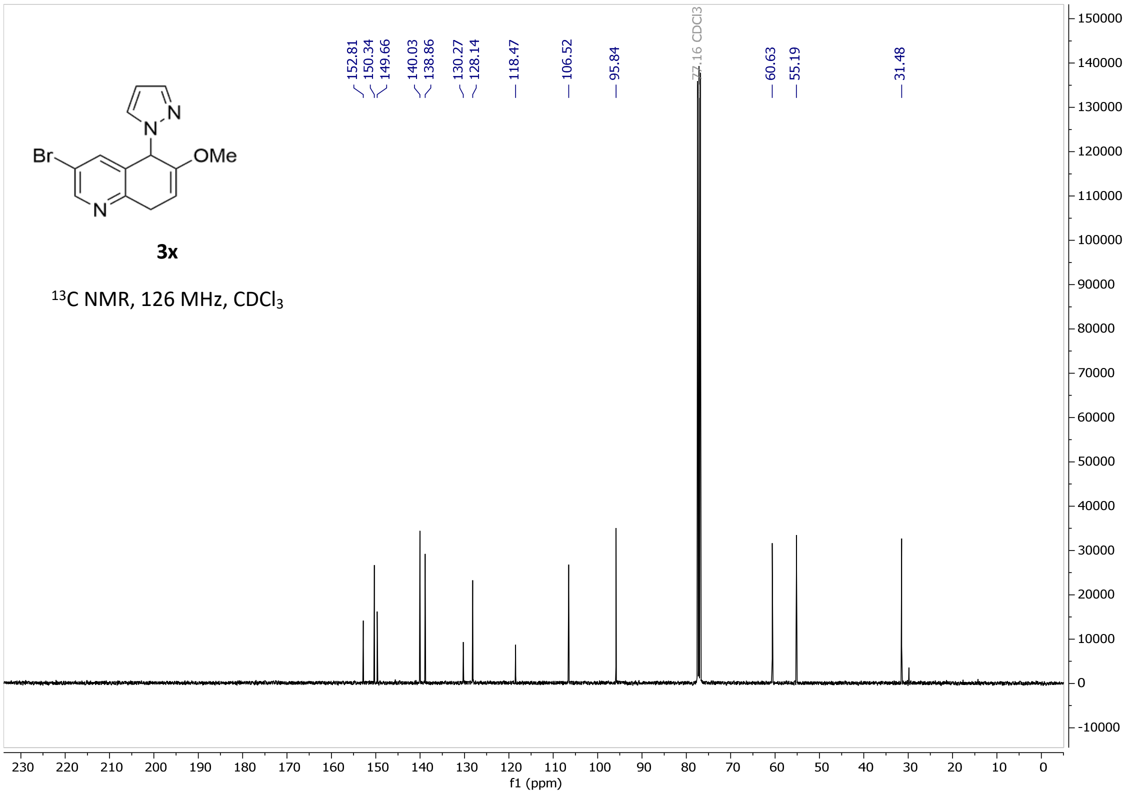

Supplementary Figure 77. ^{13}C NMR spectra of **3x** (126 MHz, rt, $CDCl_3$).

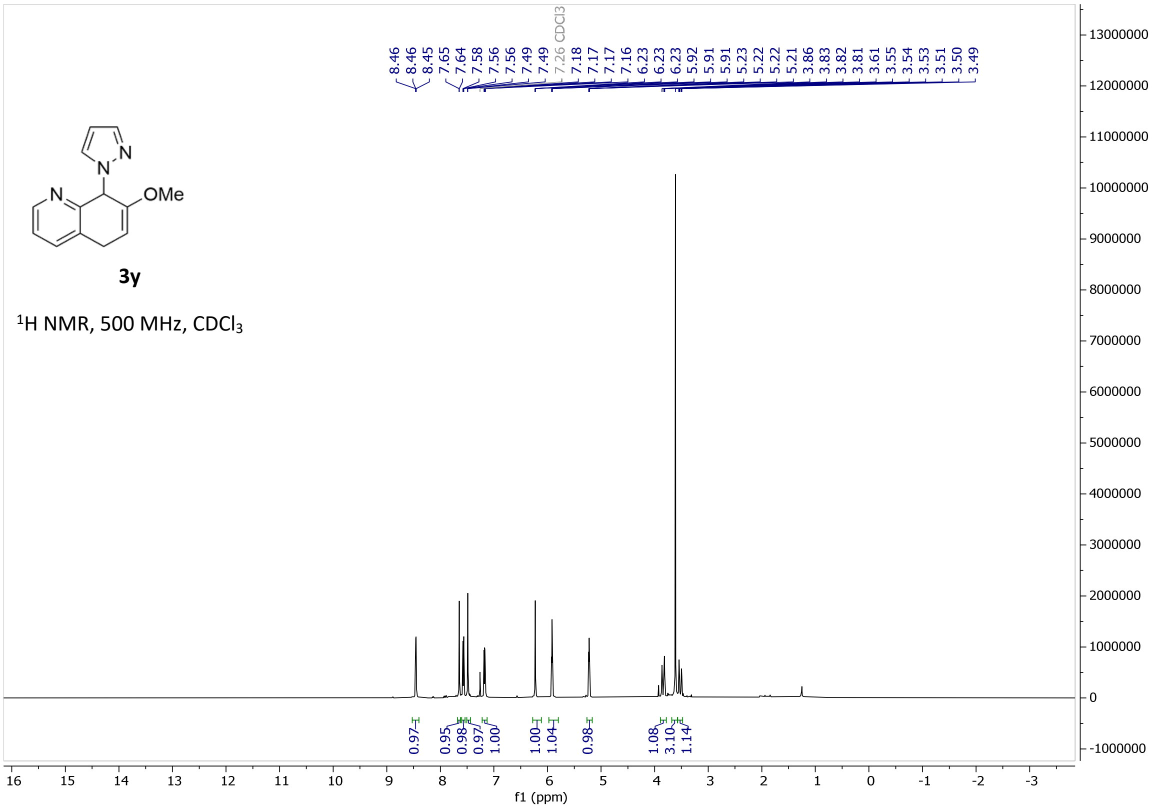

Supplementary Figure 78. ^{1}H NMR spectra of **3y** (500 MHz, rt, $CDCl_3$).

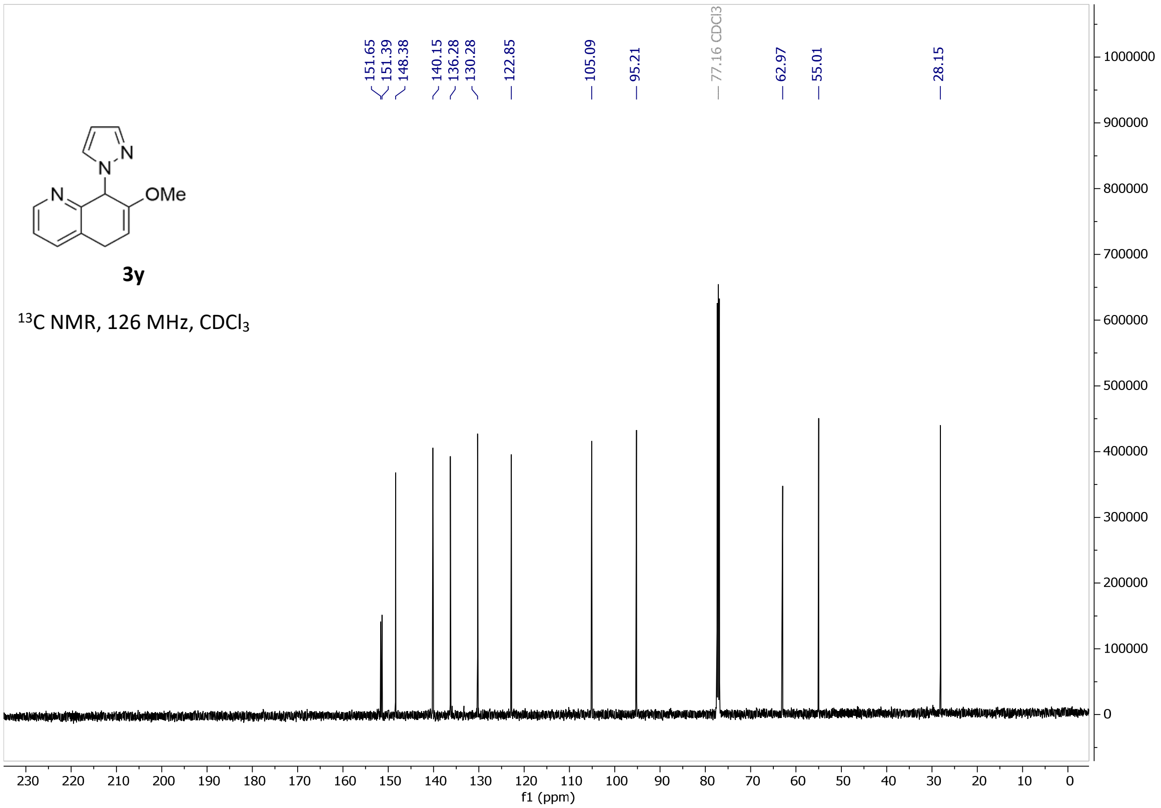

Supplementary Figure 79. ^{13}C NMR spectra of **3y** (126 MHz, rt, $CDCl_3$).

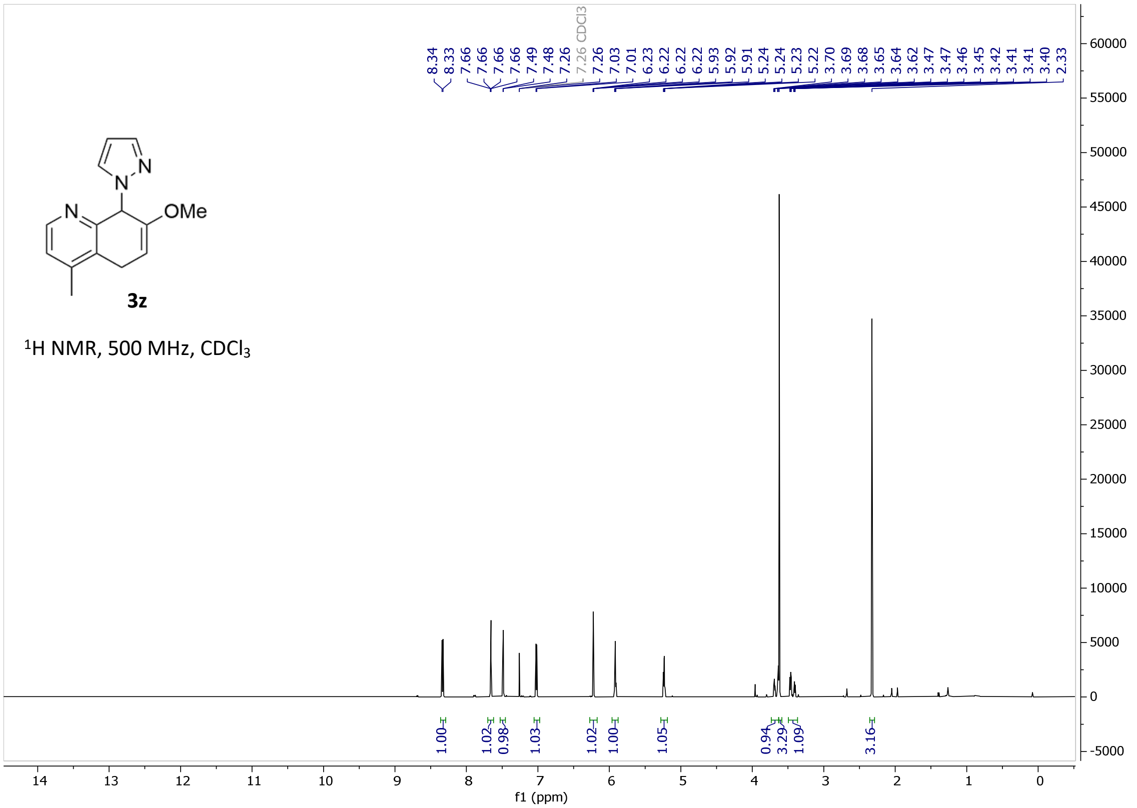

Supplementary Figure 80. ^{1}H NMR spectra of **3z** (500 MHz, rt, CDCl$_3$).

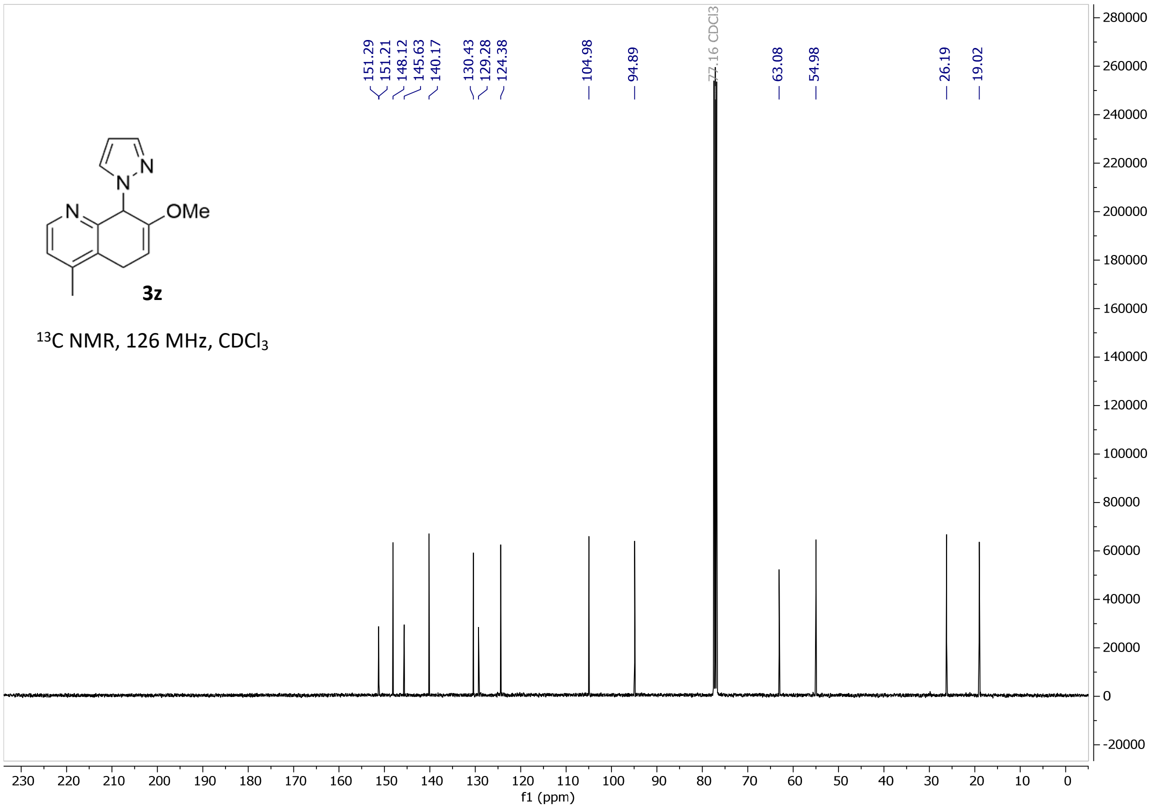

Supplementary Figure 81. ^{13}C NMR spectra of **3z** (126 MHz, rt, CDCl$_3$).

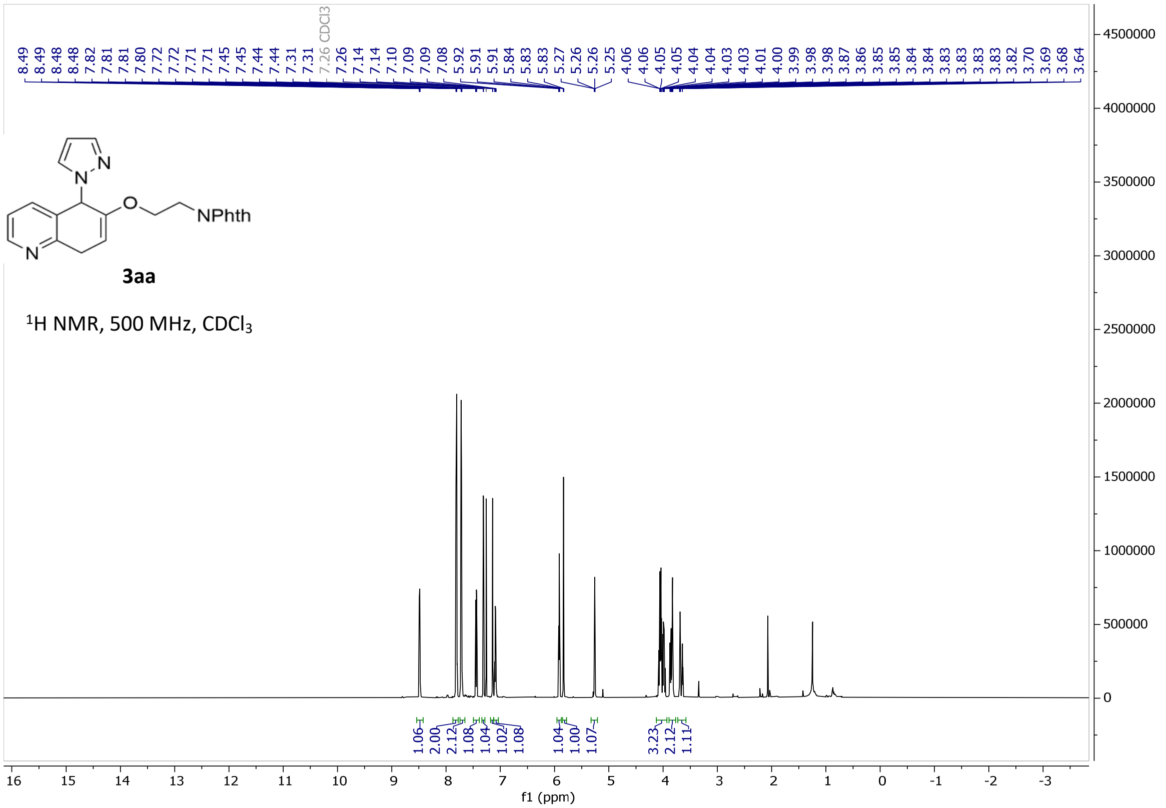

Supplementary Figure 82. ^{1}H NMR spectra of **3aa** (500 MHz, rt, CDCl$_3$).

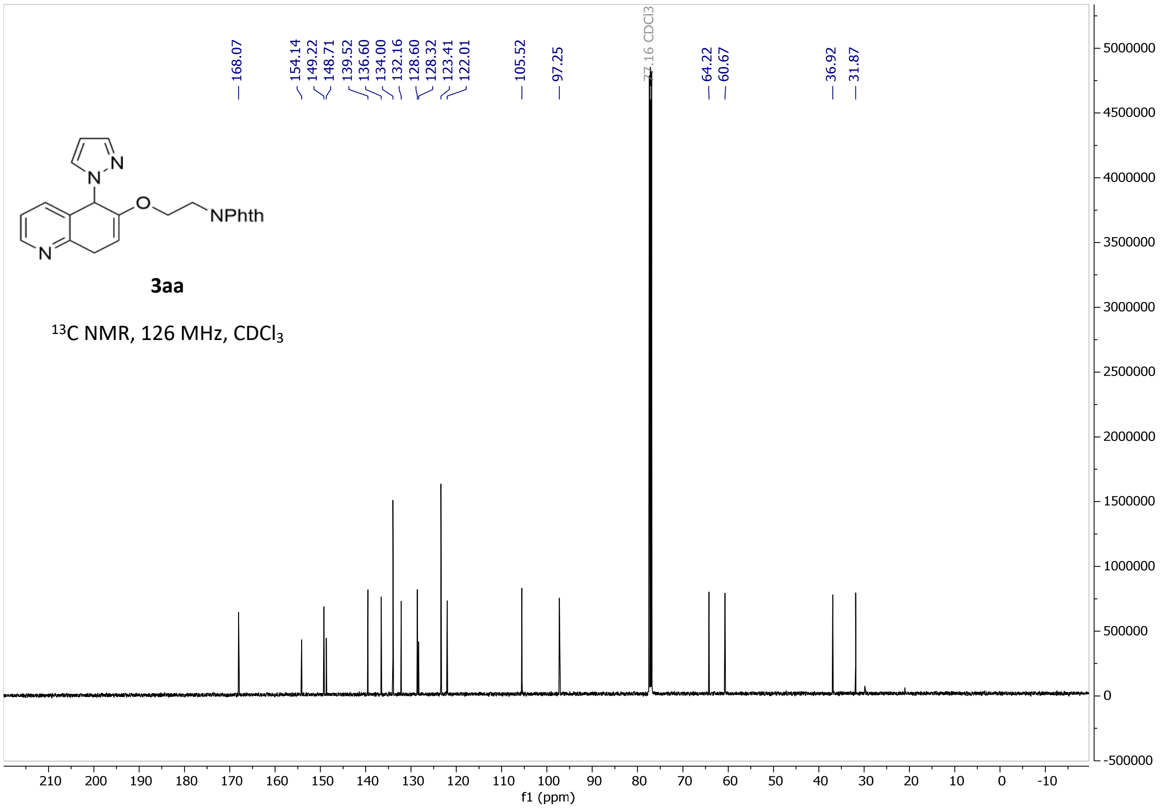

Supplementary Figure 83. ^{13}C NMR spectra of **3aa** (126 MHz, rt, CDCl$_3$).

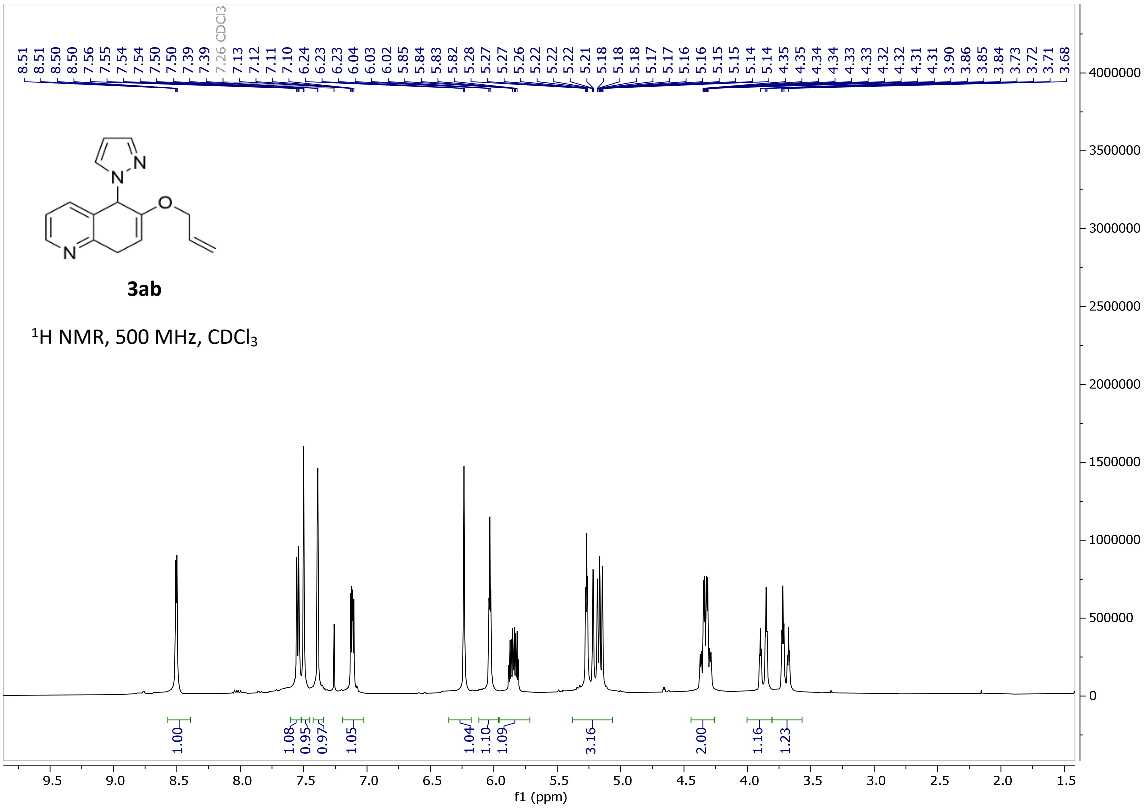

Supplementary Figure 84. ^{1}H NMR spectra of **3ab** (500 MHz, rt, $CDCl_3$).

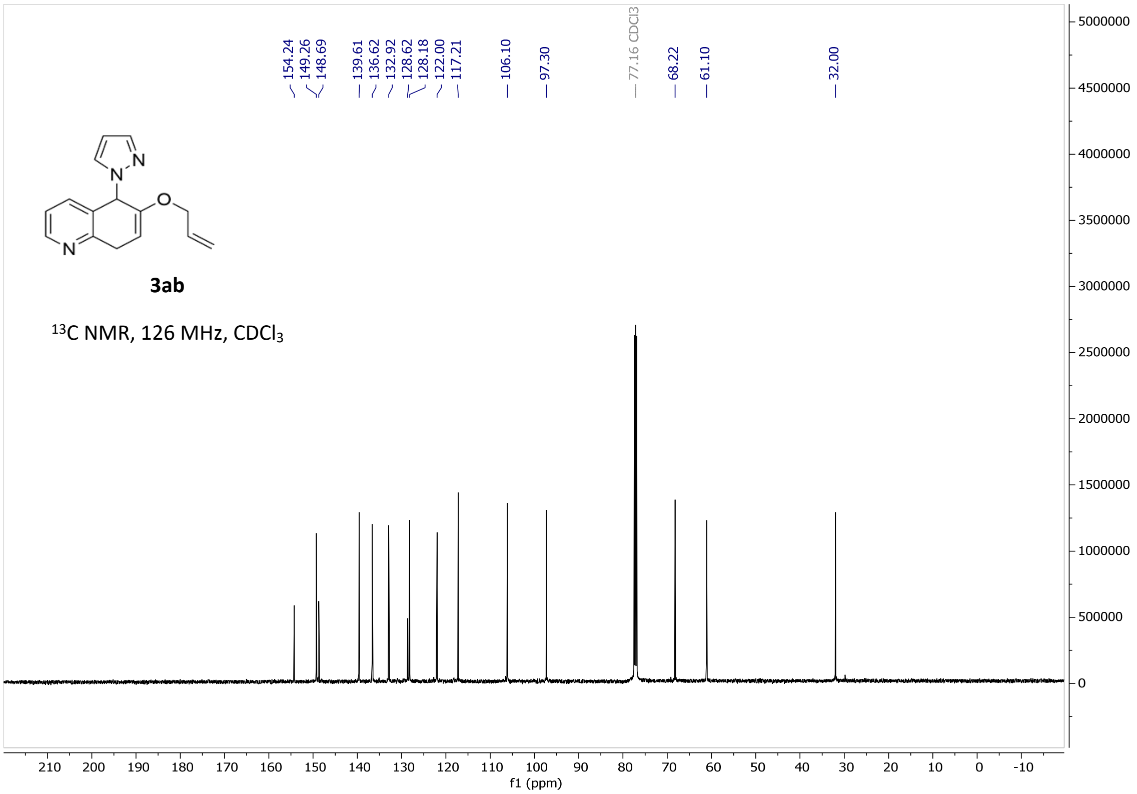

Supplementary Figure 85. ^{13}C NMR spectra of **3ab** (126 MHz, rt, $CDCl_3$).

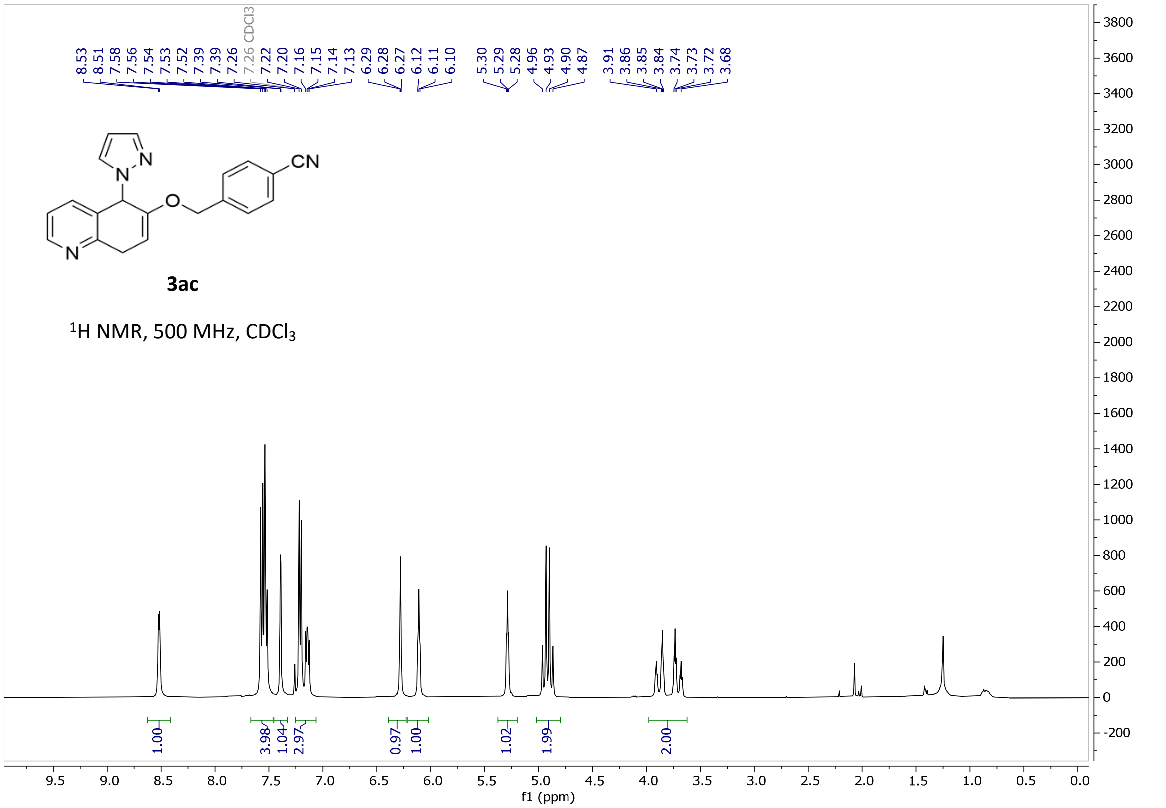

Supplementary Figure 86. ^{1}H NMR spectra of **3ac** (500 MHz, rt, $CDCl_3$).

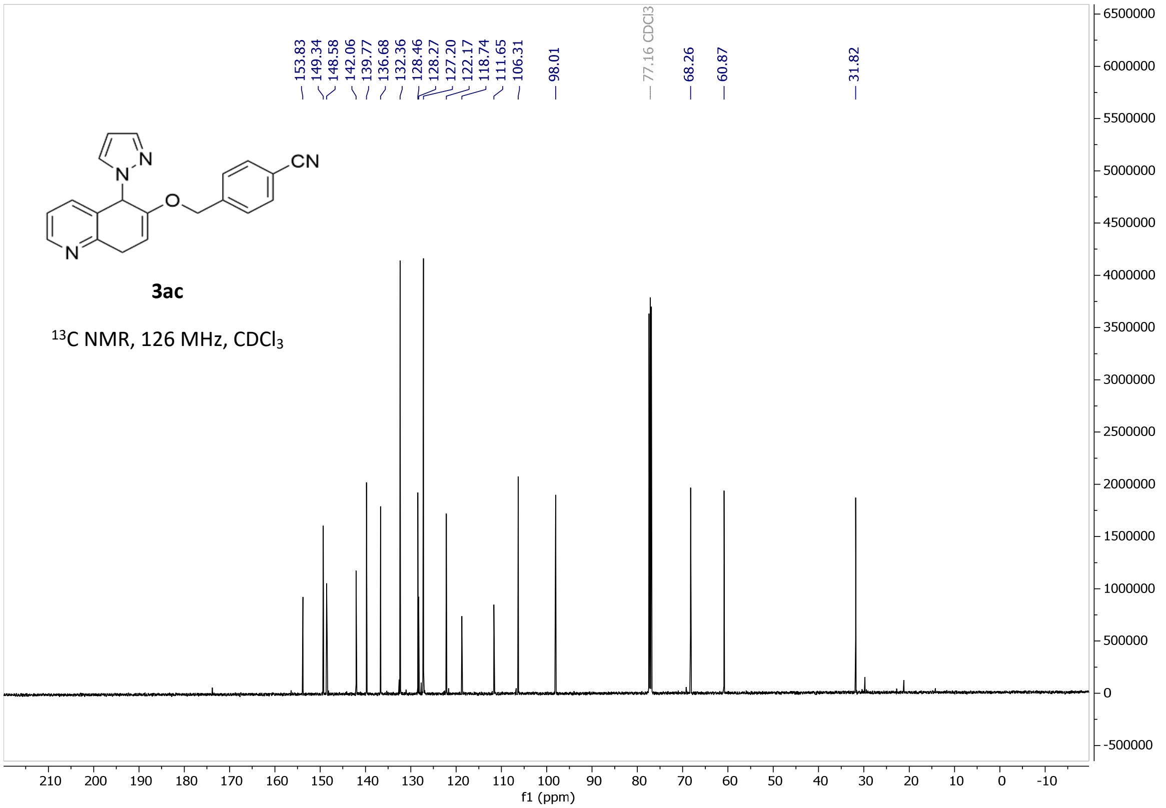

Supplementary Figure 87. ^{13}C NMR spectra of **3ac** (126 MHz, rt, $CDCl_3$).

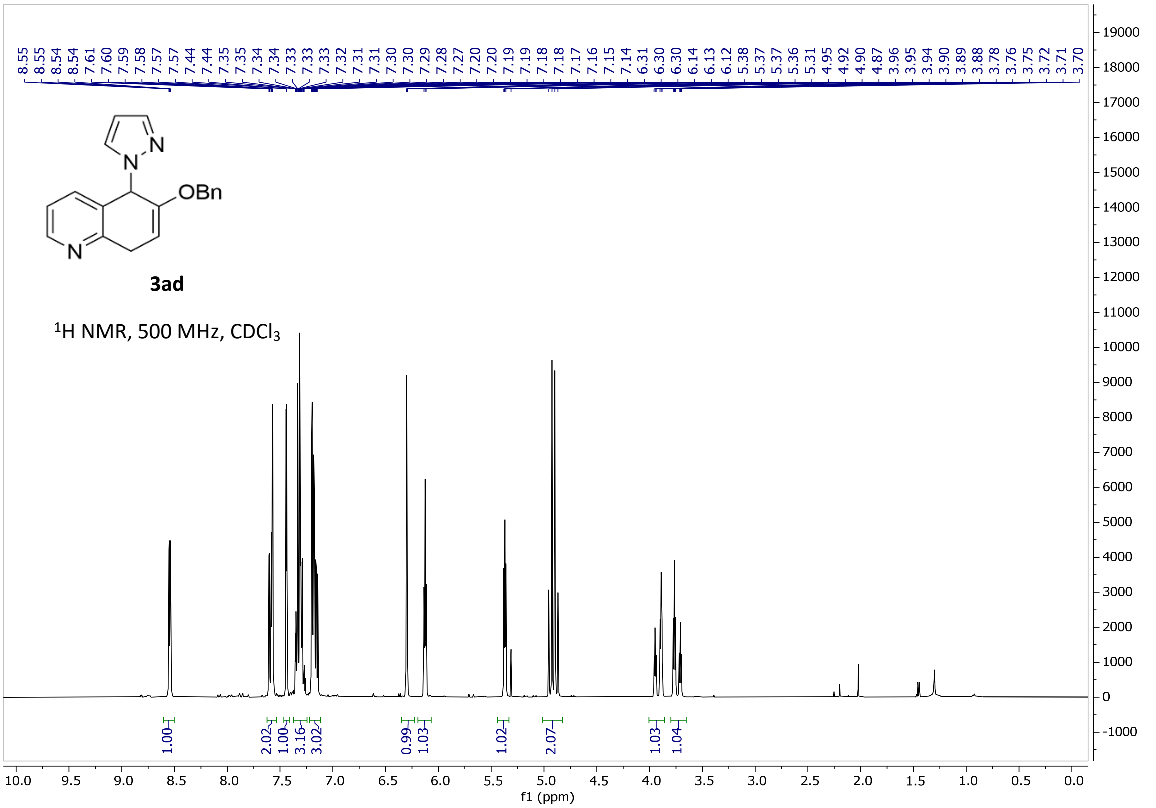

Supplementary Figure 88. ^{1}H NMR spectra of **3ad** (500 MHz, rt, CDCl$_3$).

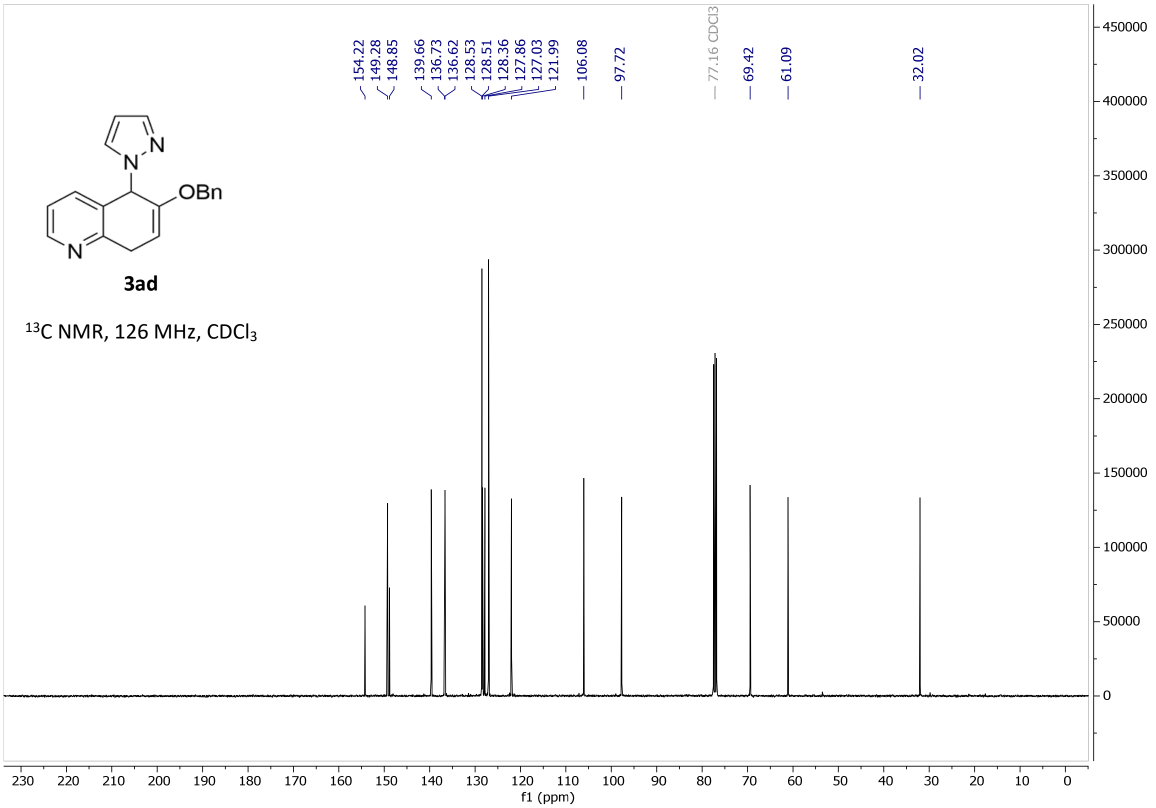

Supplementary Figure 89. ^{13}C NMR spectra of **3ad** (126 MHz, rt, CDCl$_3$).

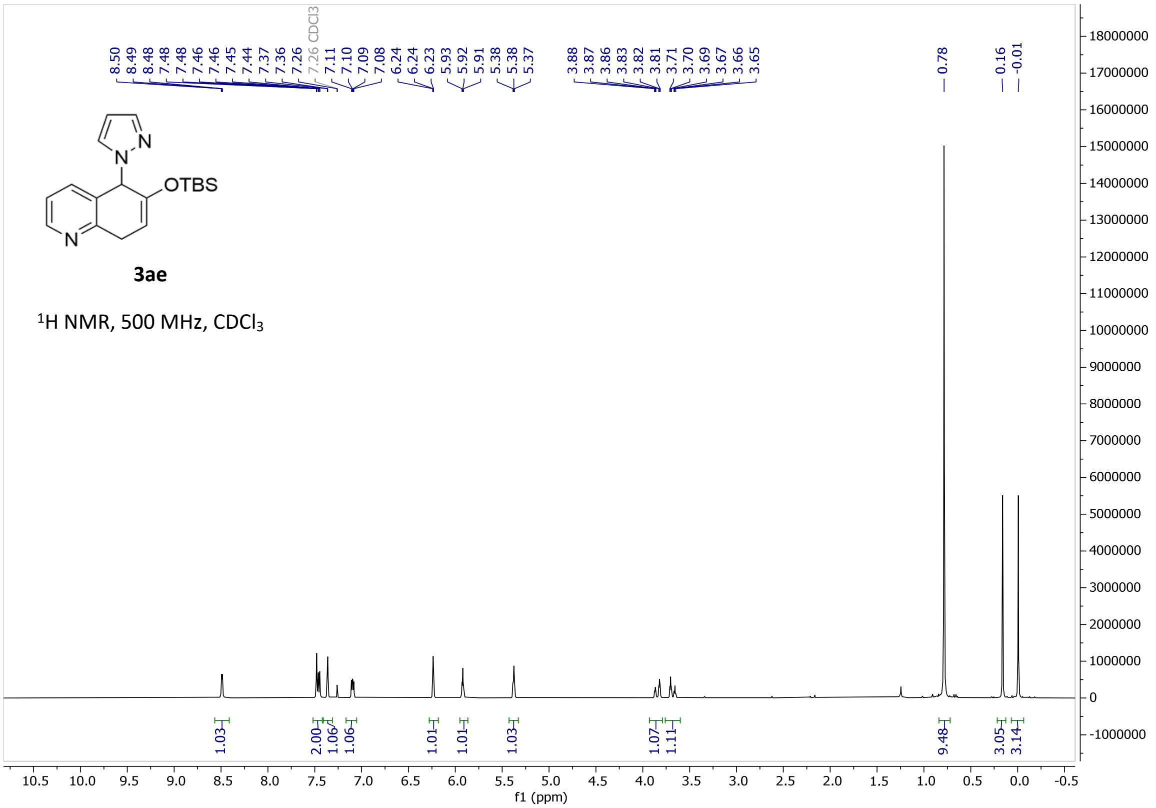

Supplementary Figure 90. ^{1}H NMR spectra of **3ae** (500 MHz, rt, CDCl$_3$).

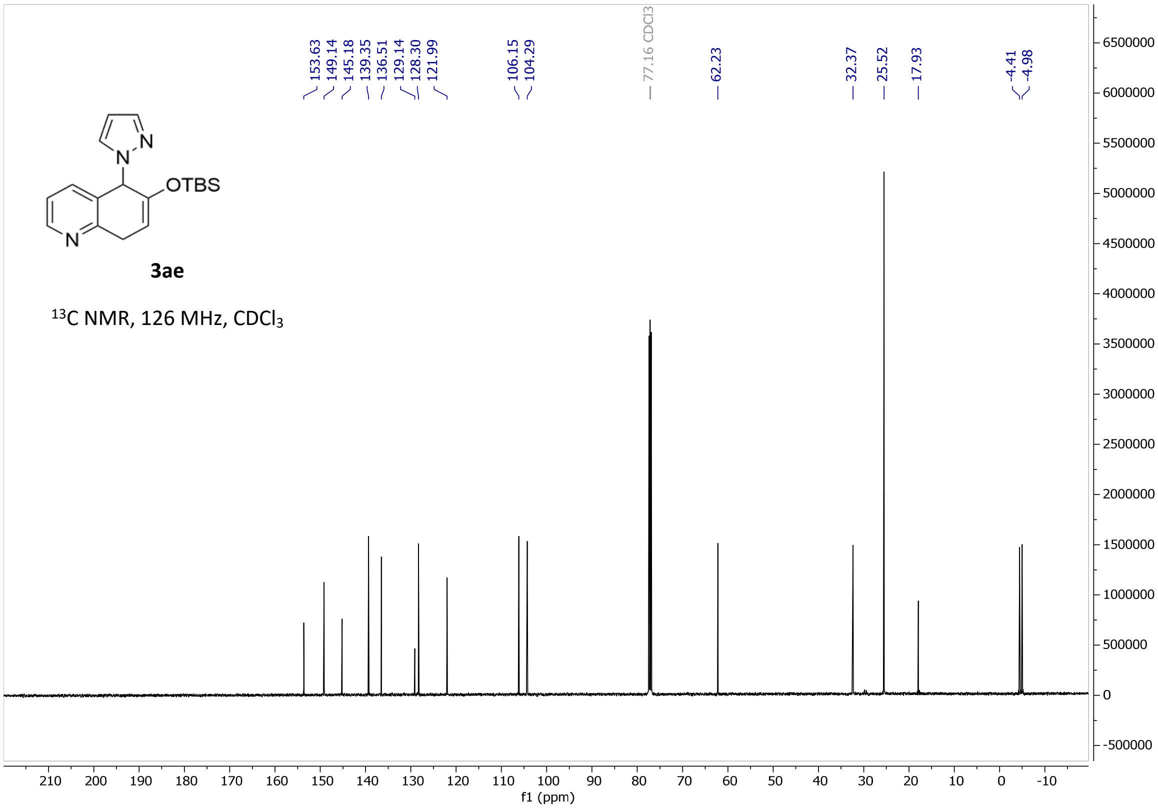

Supplementary Figure 91. ^{13}C NMR spectra of **3ae** (126 MHz, rt, CDCl$_3$).

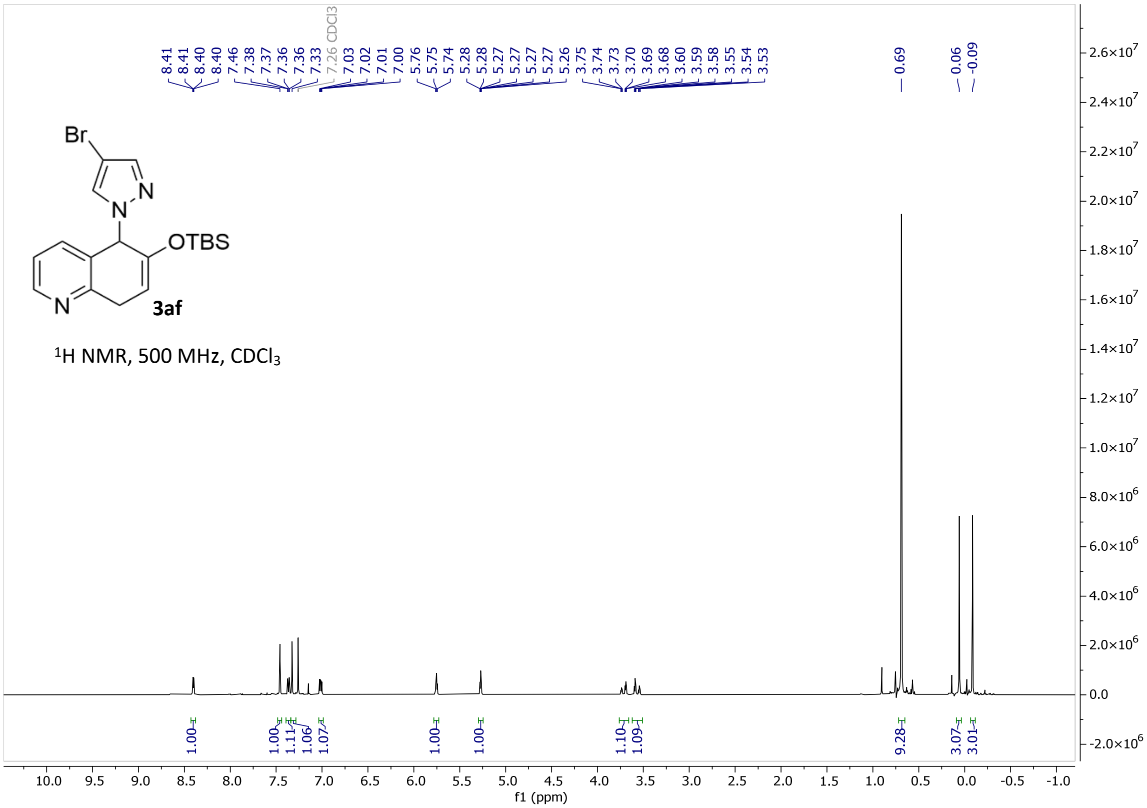

Supplementary Figure 92. ^{1}H NMR spectra of **3af** (500 MHz, rt, $CDCl_3$).

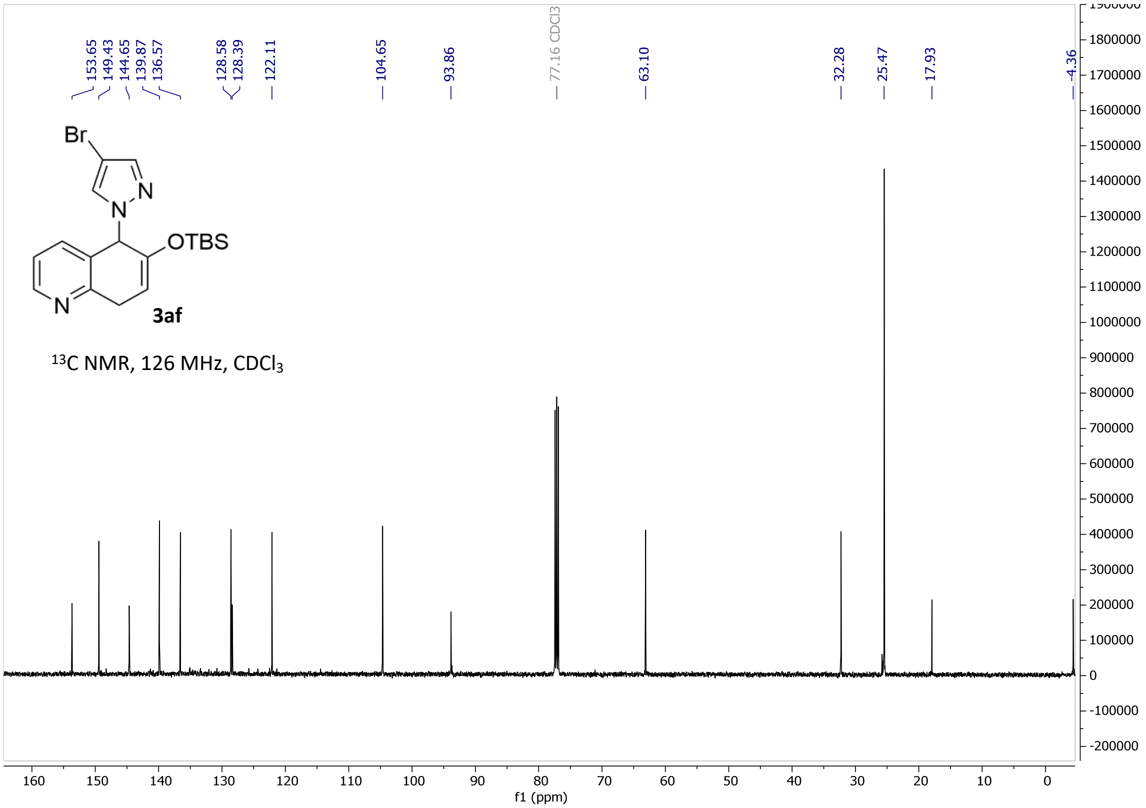

Supplementary Figure 93. ^{13}C NMR spectra of **3af** (126 MHz, rt, $CDCl_3$).

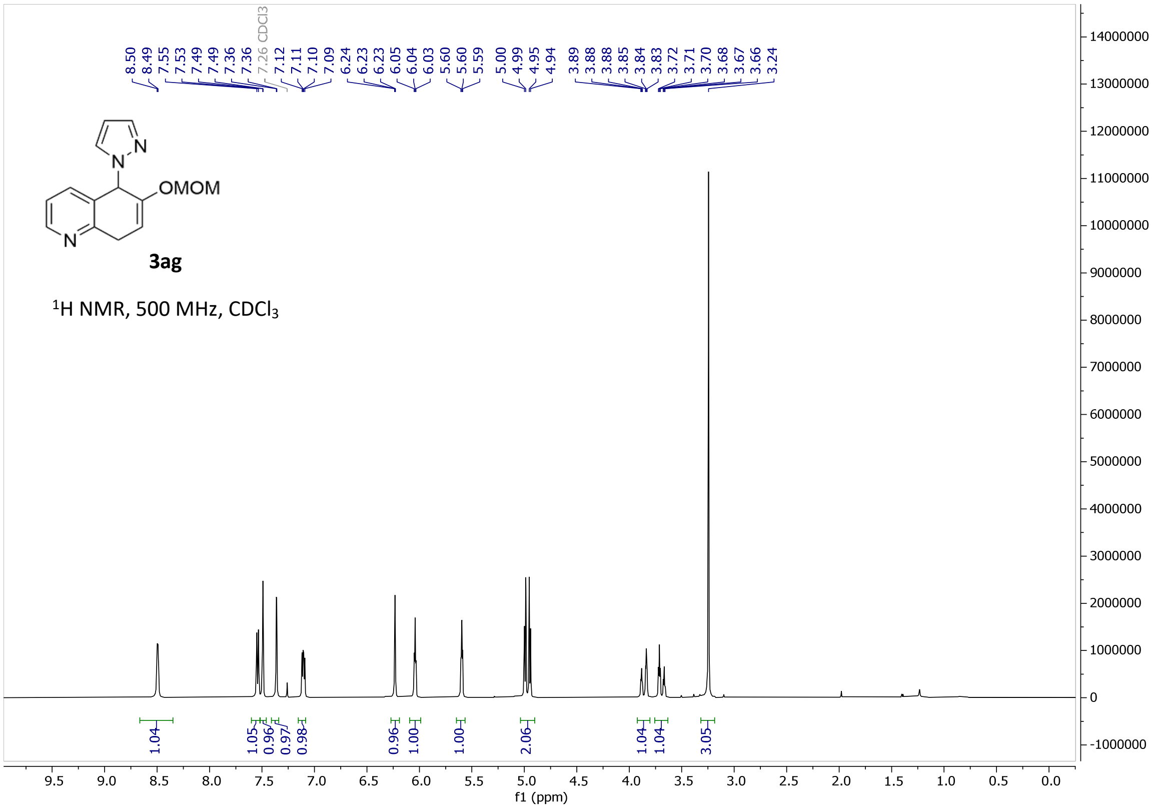

Supplementary Figure 94. ^{1}H NMR spectra of **3ag** (500 MHz, rt, $CDCl_3$).

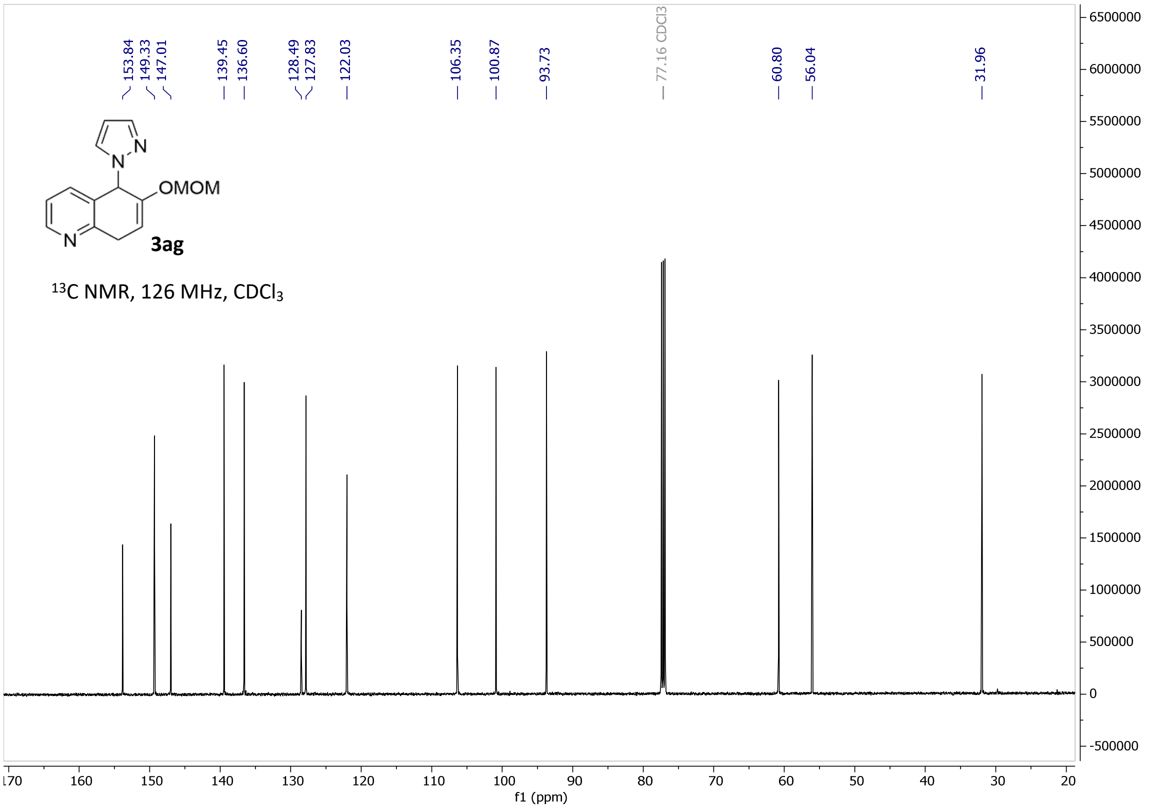

Supplementary Figure 95. ^{13}C NMR spectra of **3ag** (126 MHz, rt, $CDCl_3$).

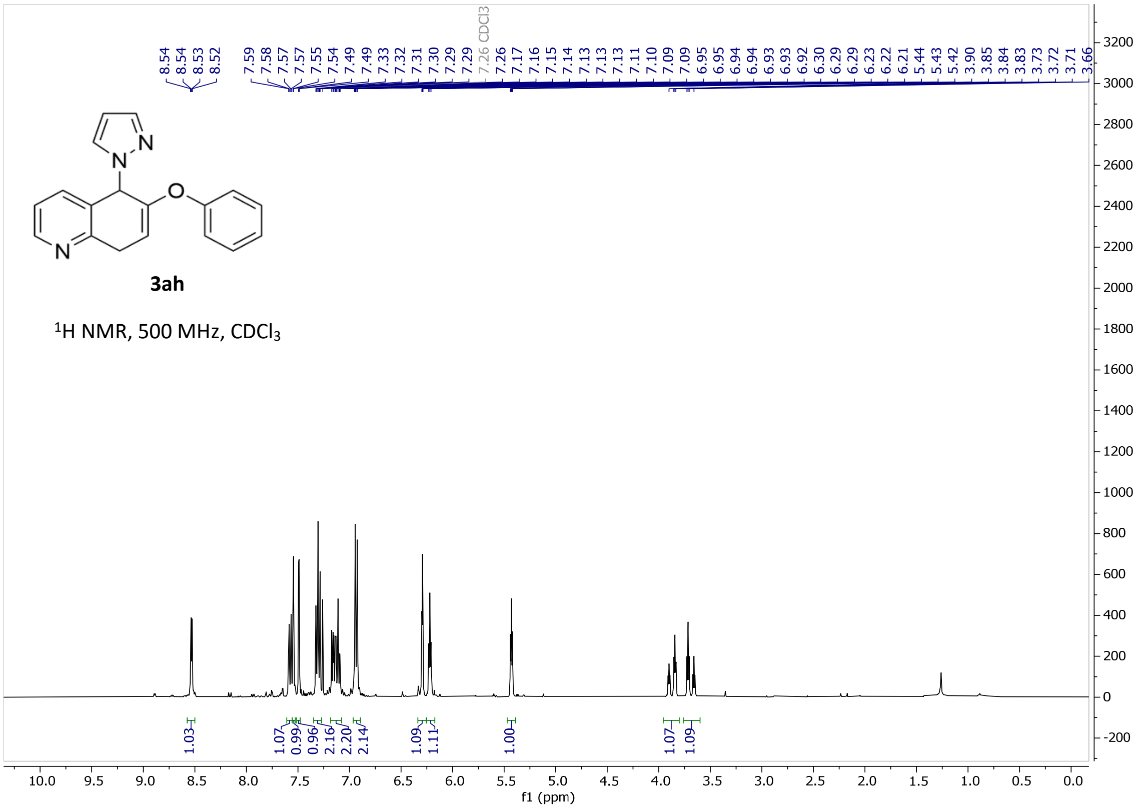

Supplementary Figure 96. ^{1}H NMR spectra of **3ah** (500 MHz, rt, $CDCl_3$).

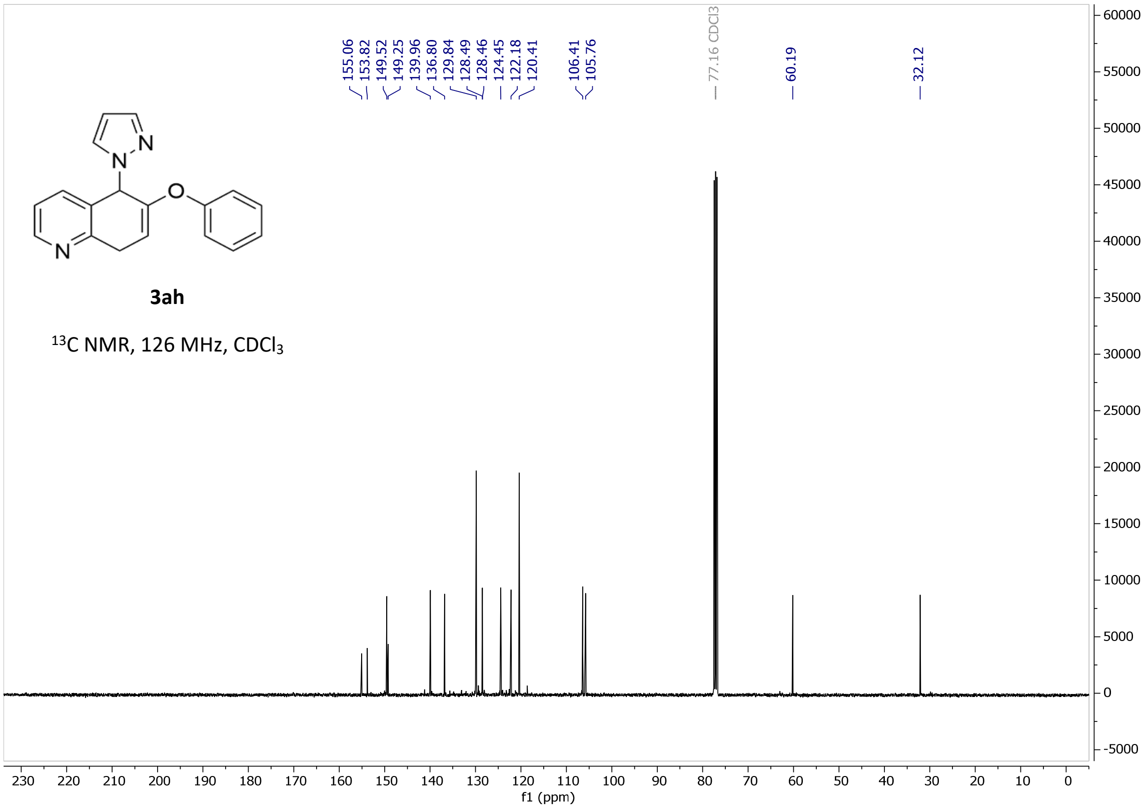

Supplementary Figure 97. ^{13}C NMR spectra of **3ah** (126 MHz, rt, $CDCl_3$).

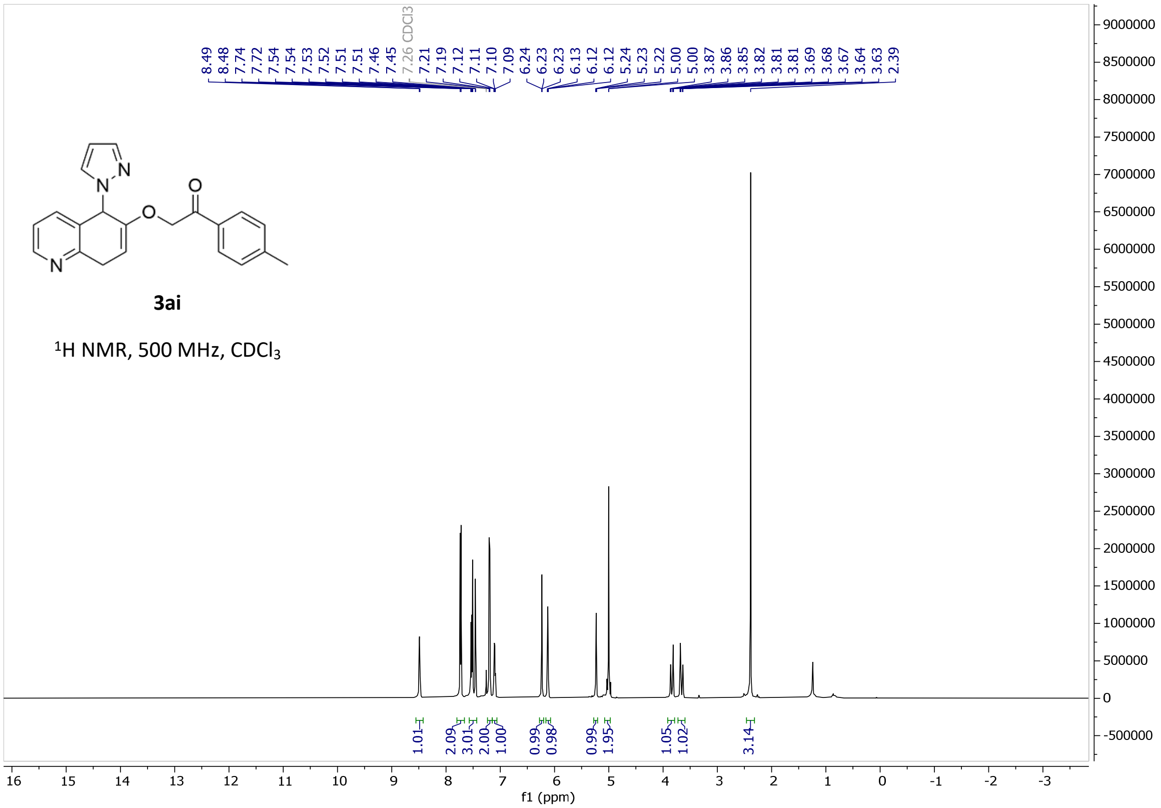

Supplementary Figure 98. ^{1}H NMR spectra of **3ai** (500 MHz, rt, $CDCl_3$).

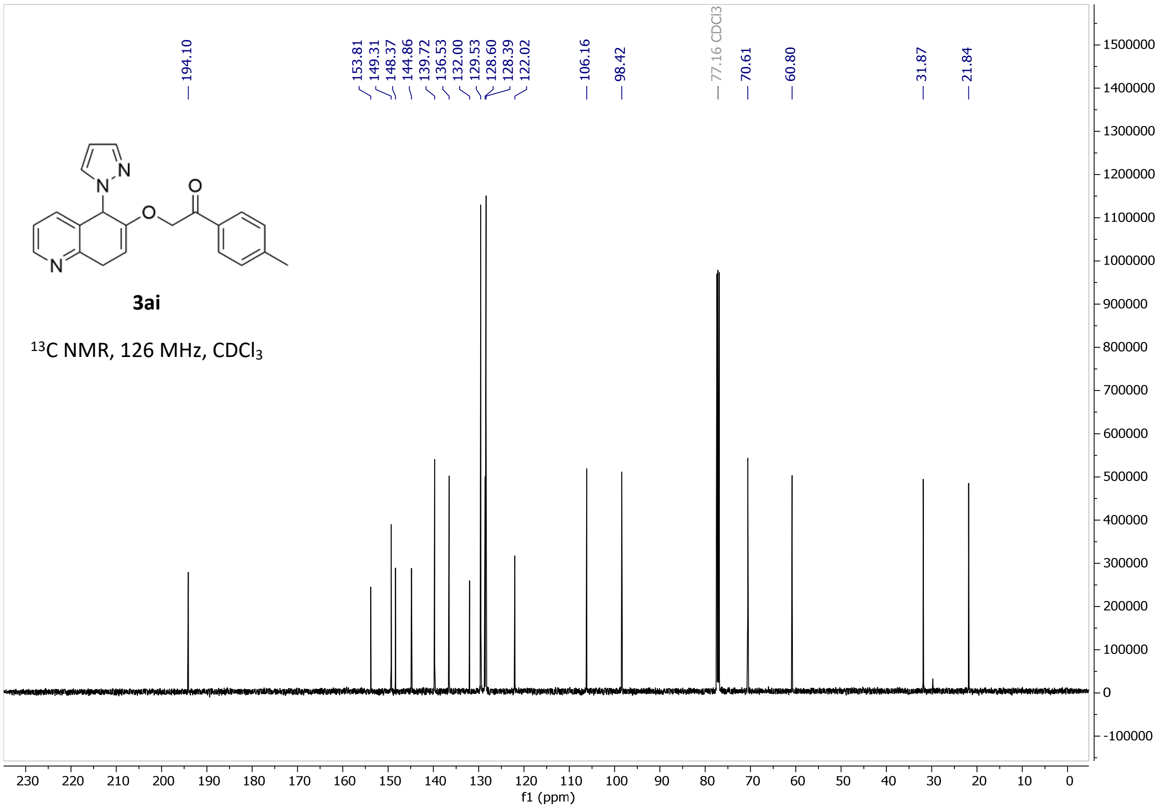

Supplementary Figure 99. ^{13}C NMR spectra of **3ai** (126 MHz, rt, $CDCl_3$).

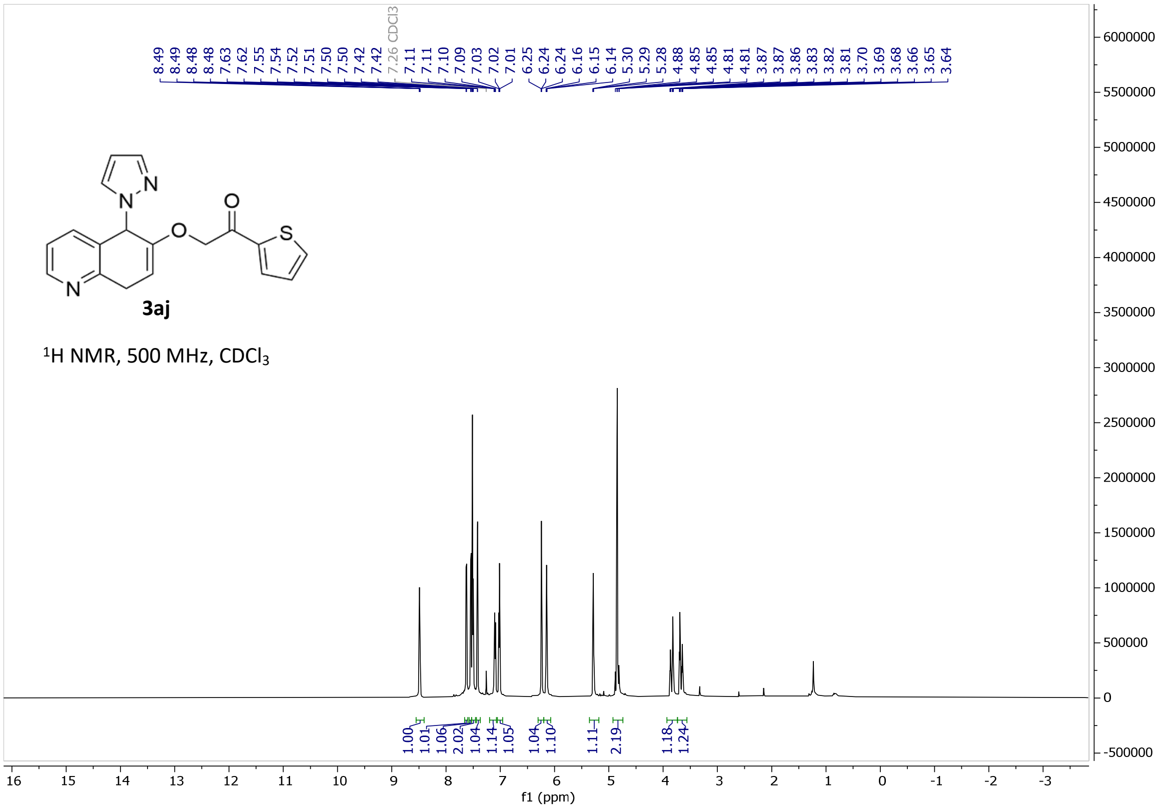

Supplementary Figure 100. ^{1}H NMR spectra of **3aj** (500 MHz, rt, $CDCl_3$).

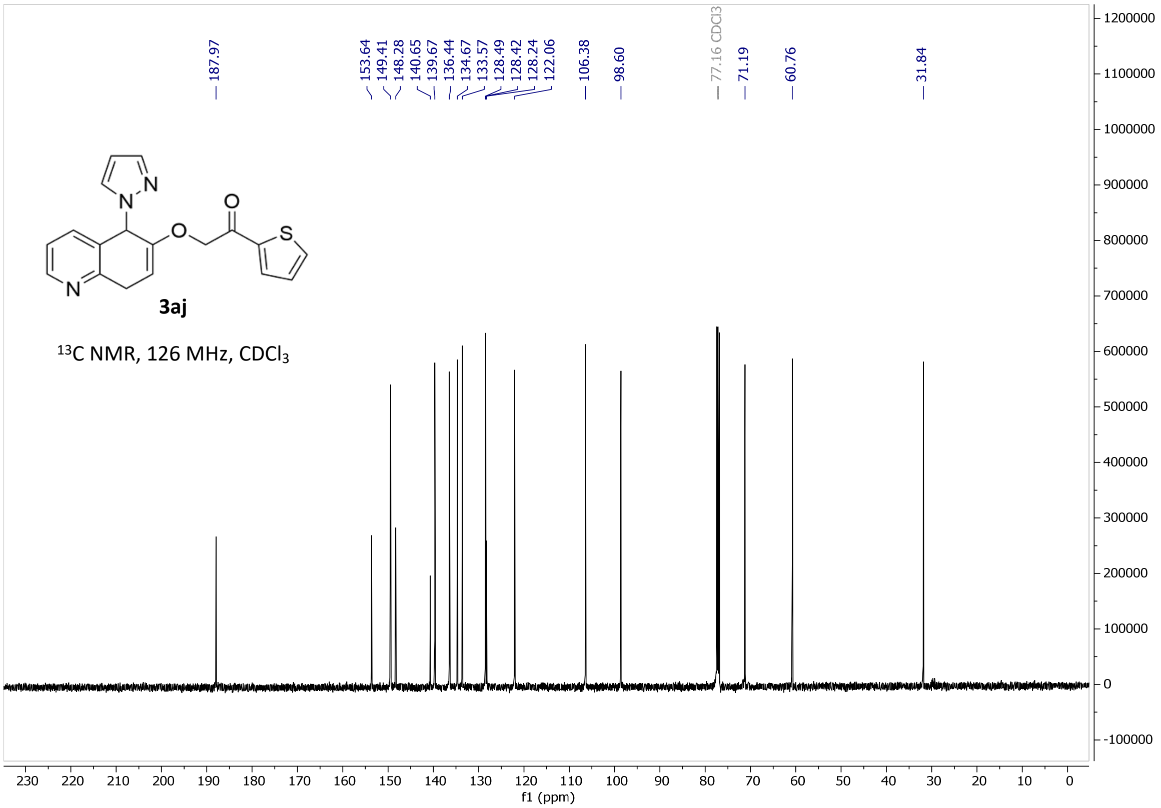

Supplementary Figure 101. ^{13}C NMR spectra of **3aj** (126 MHz, rt, $CDCl_3$).

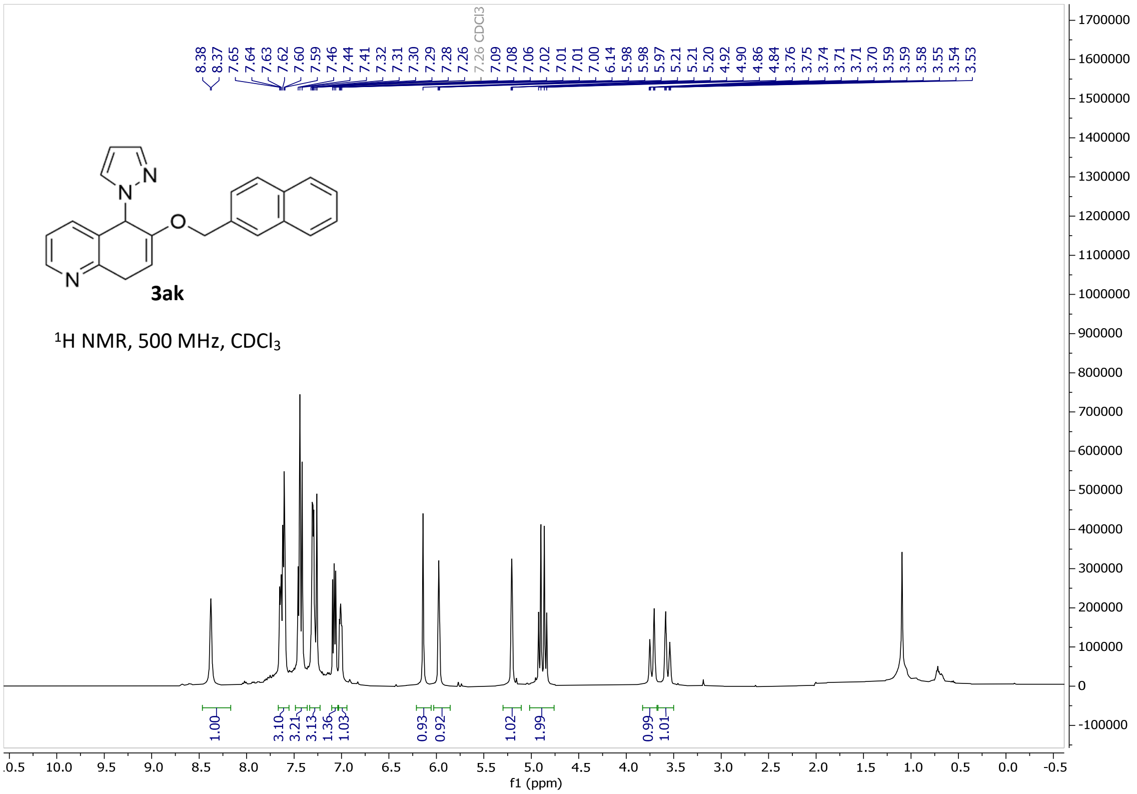

Supplementary Figure 102. ^{1}H NMR spectra of **3ak** (500 MHz, rt, $CDCl_3$).

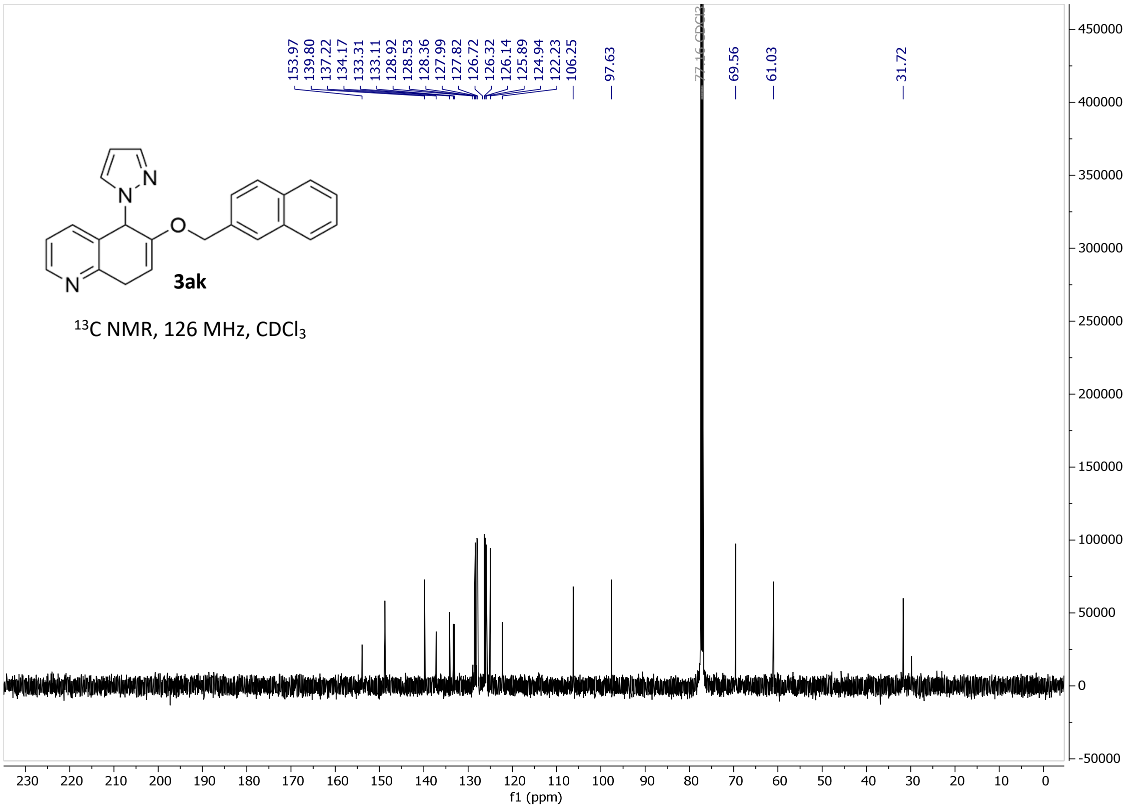

Supplementary Figure 103. ^{13}C NMR spectra of **3ak** (126 MHz, rt, $CDCl_3$).

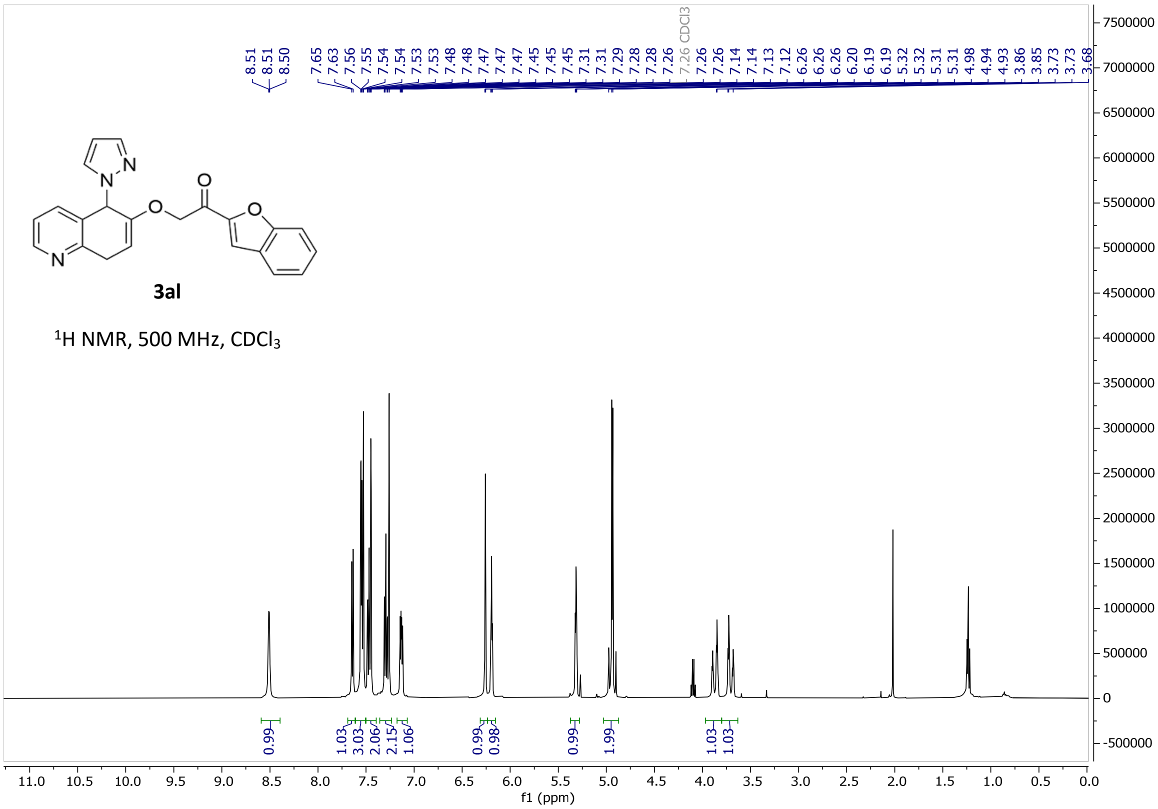

Supplementary Figure 104. ^{1}H NMR spectra of **3al** (500 MHz, rt, $CDCl_3$).

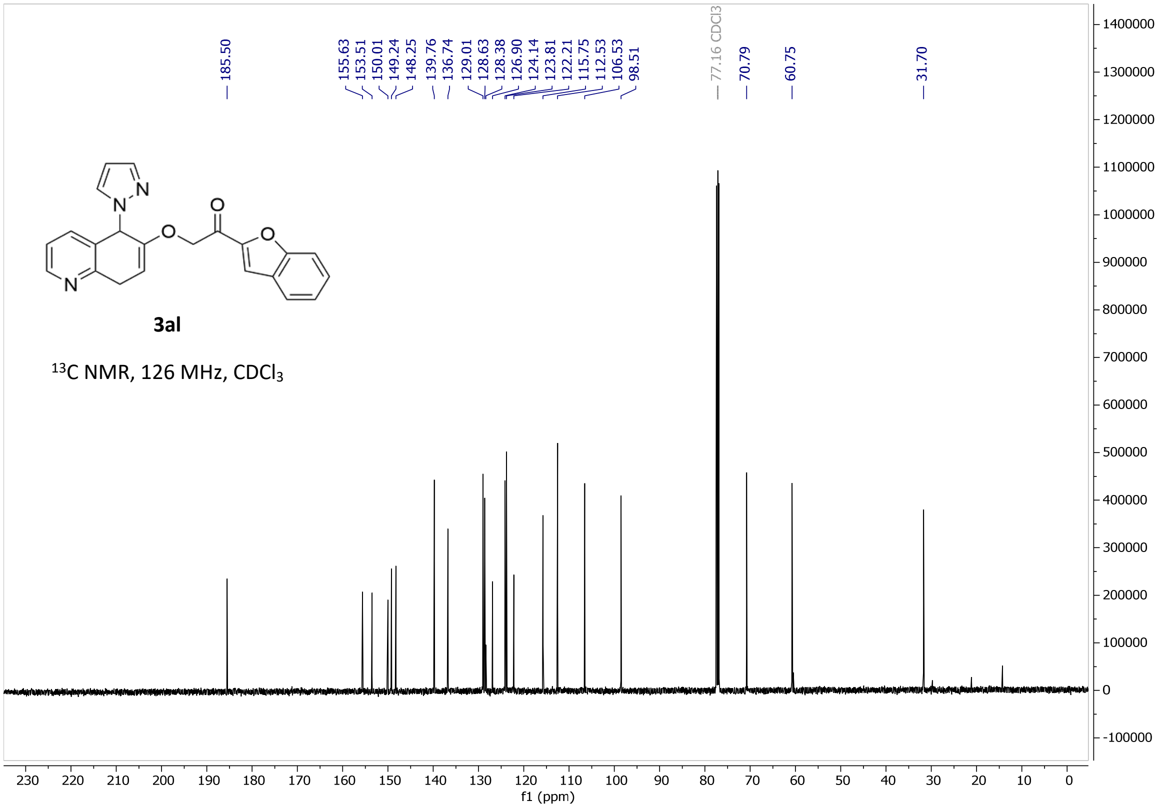

Supplementary Figure 105. ^{13}C NMR spectra of **3al** (126 MHz, rt, $CDCl_3$).

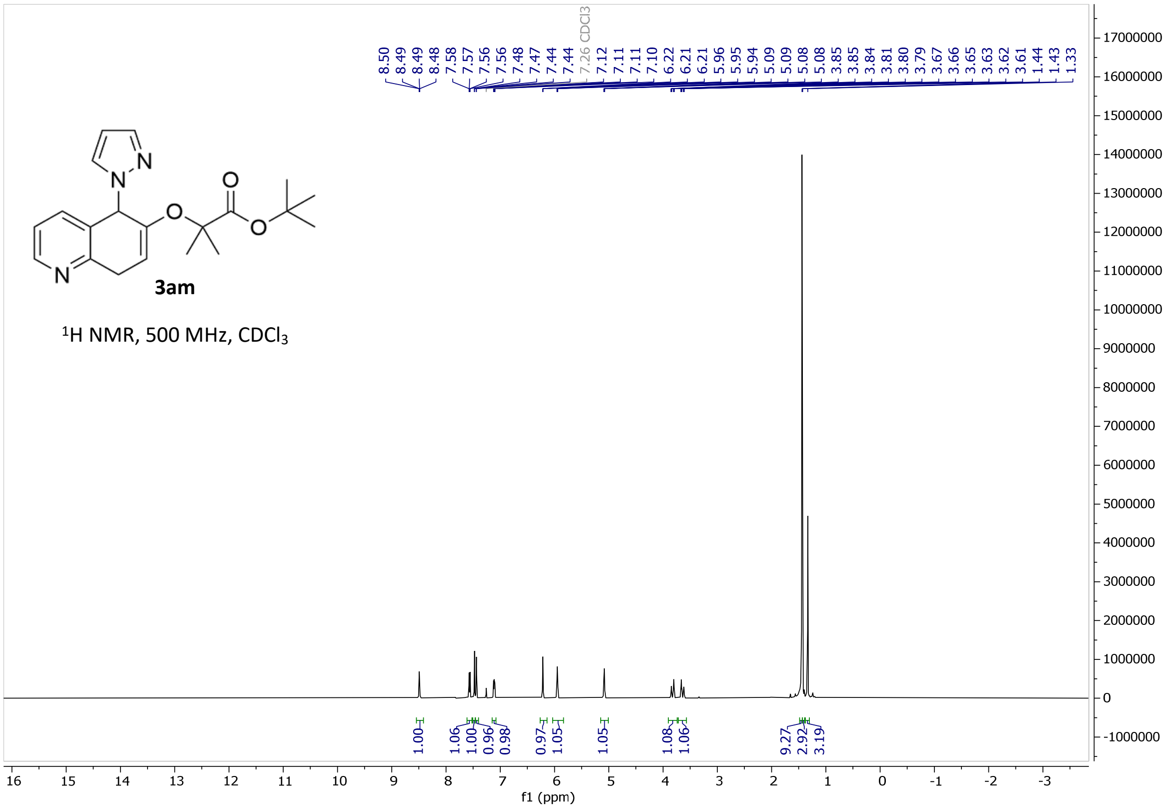

Supplementary Figure 106. ^{1}H NMR spectra of **3am** (500 MHz, rt, $CDCl_3$).

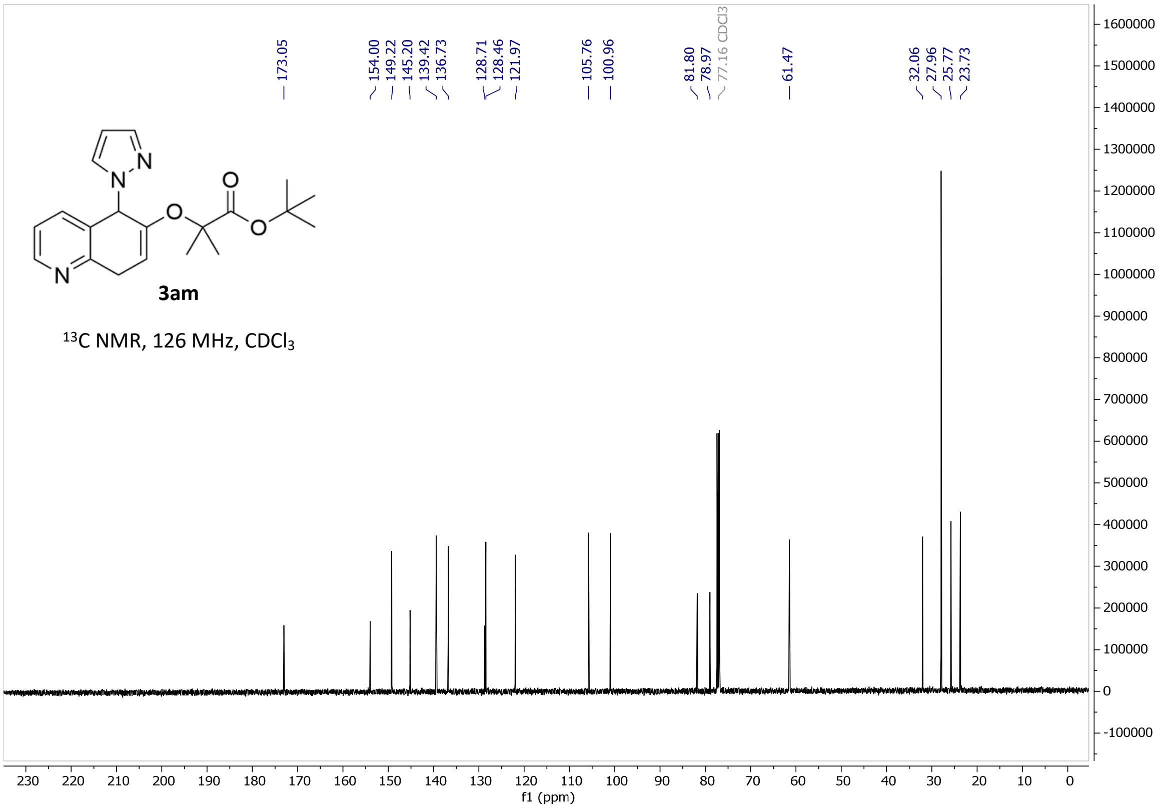

Supplementary Figure 107. ^{13}C NMR spectra of **3am** (126 MHz, rt, $CDCl_3$).

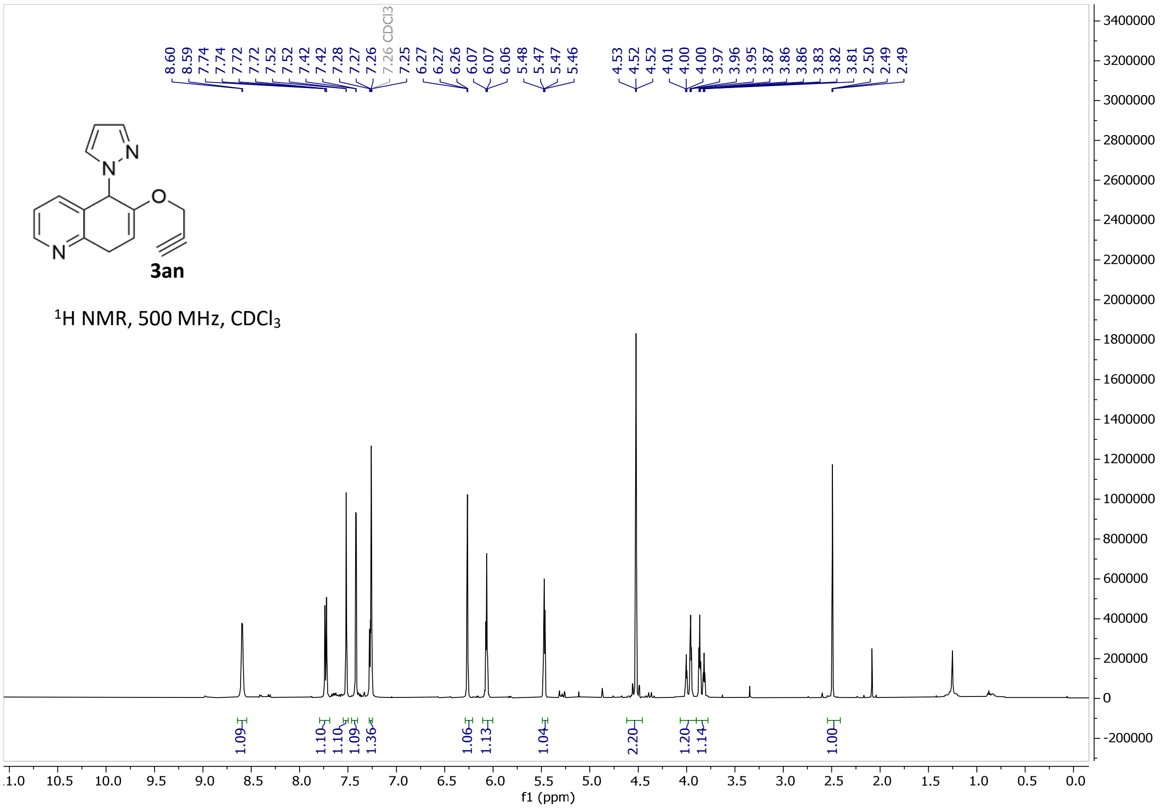

Supplementary Figure 108. ^{1}H NMR spectra of **3an** (500 MHz, rt, $CDCl_3$).

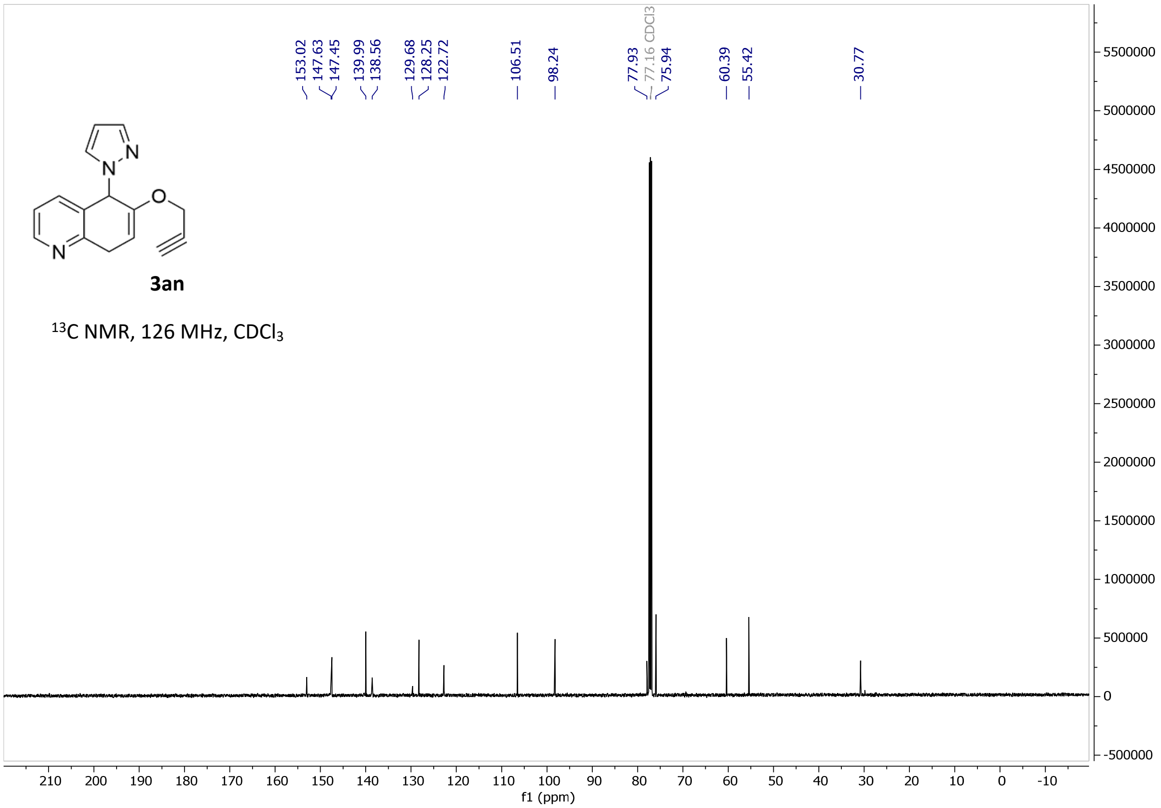

Supplementary Figure 109. ^{13}C NMR spectra of **3an** (126 MHz, rt, $CDCl_3$).

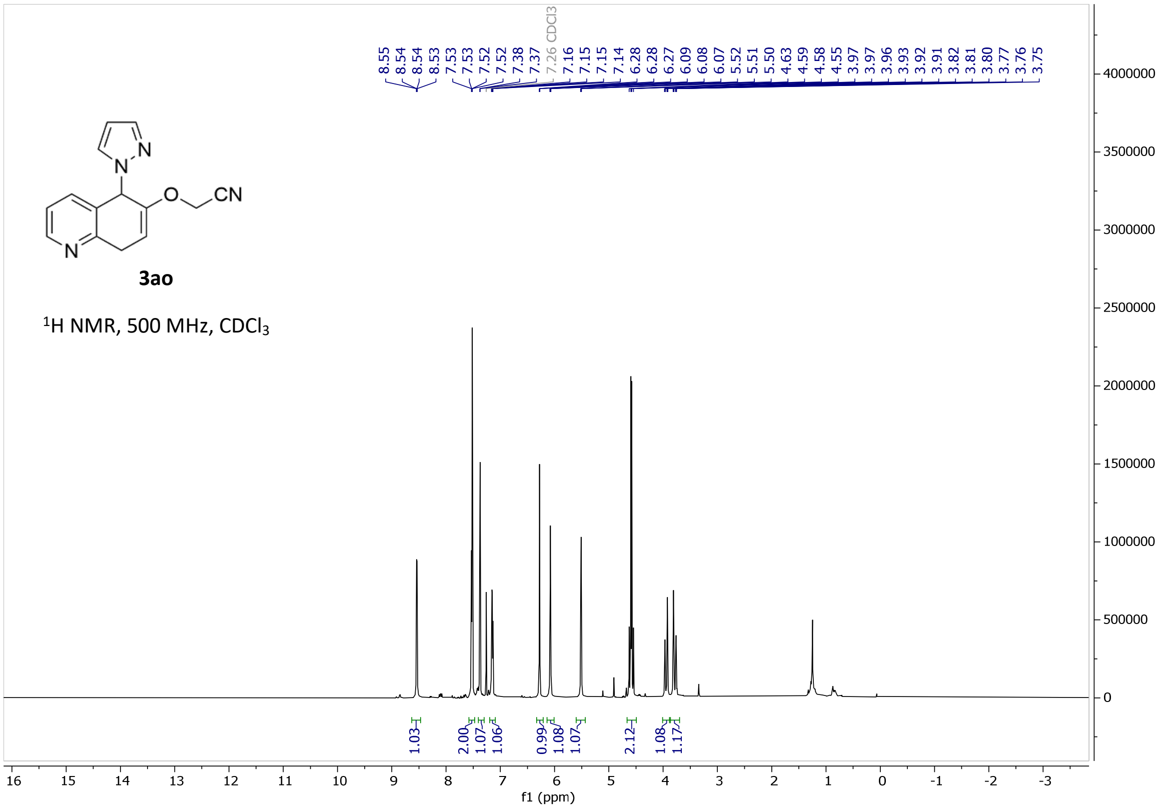

Supplementary Figure 110. ^{1}H NMR spectra of **3ao** (500 MHz, rt, $CDCl_3$).

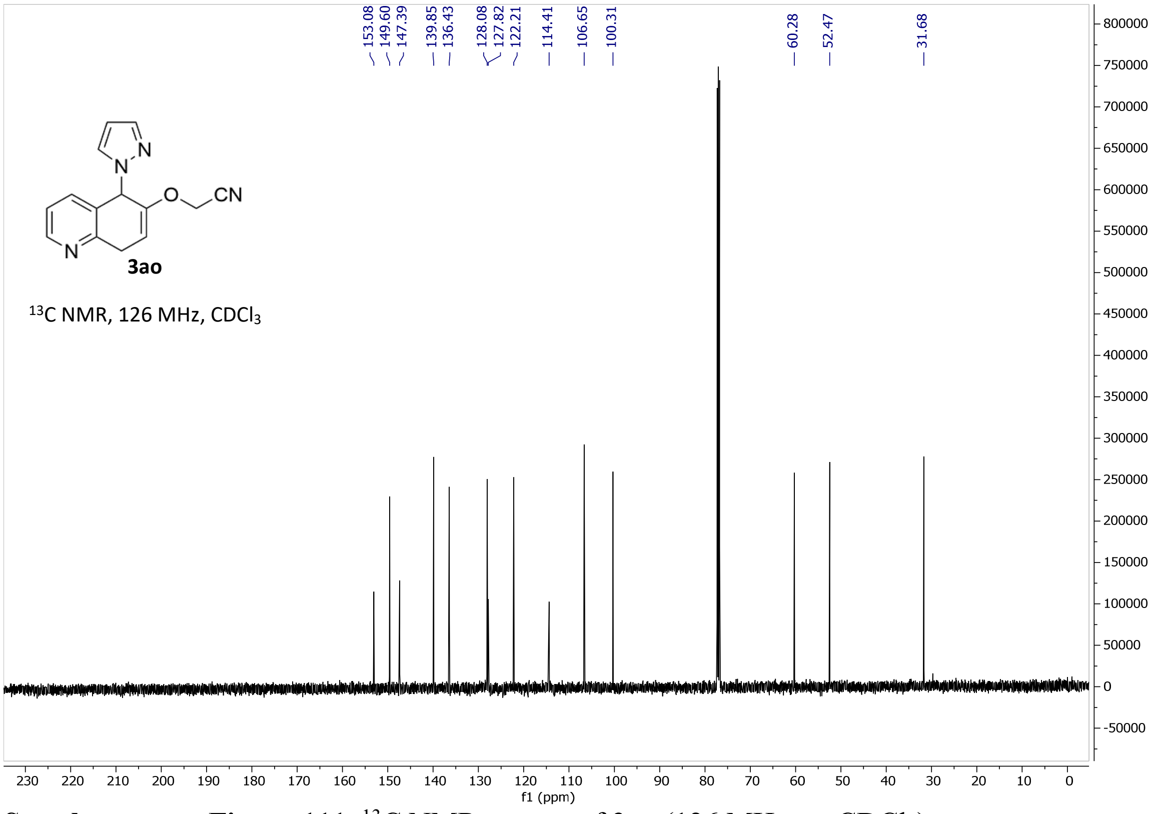

Supplementary Figure 111. ^{13}C NMR spectra of **3ao** (126 MHz, rt, $CDCl_3$).

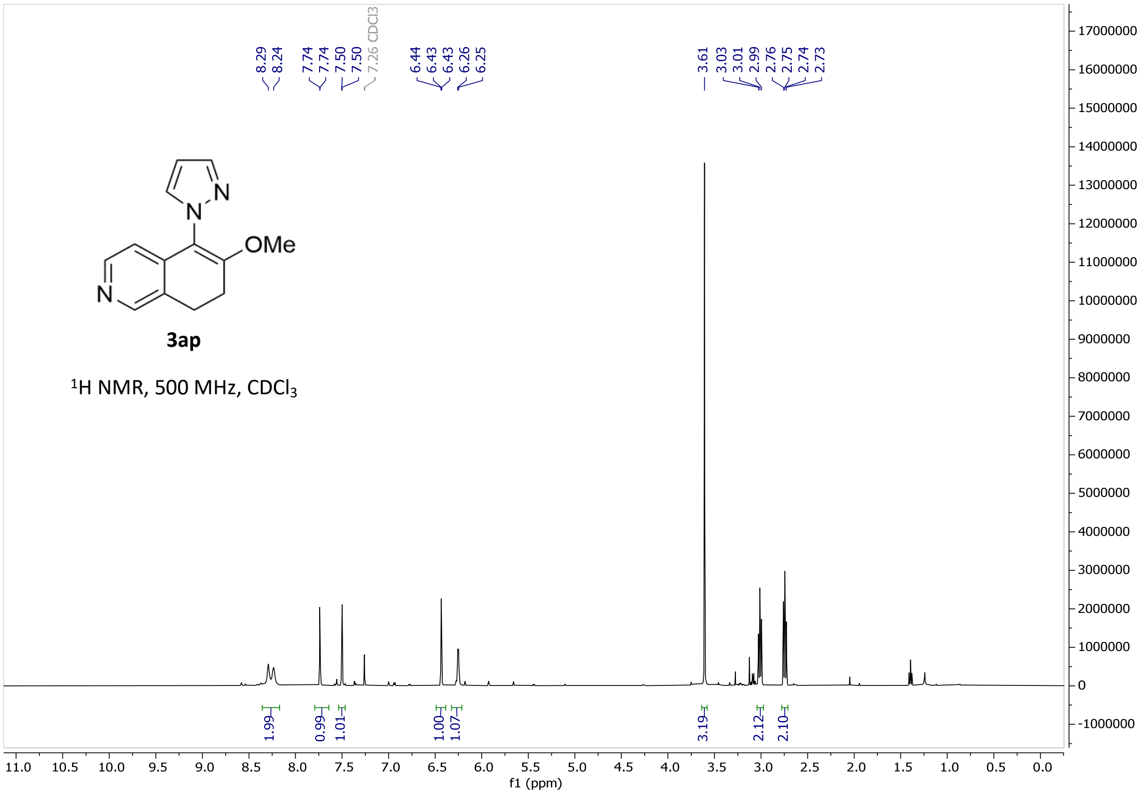

Supplementary Figure 112. ^{1}H NMR spectra of **3ap** (500 MHz, rt, $CDCl_3$).

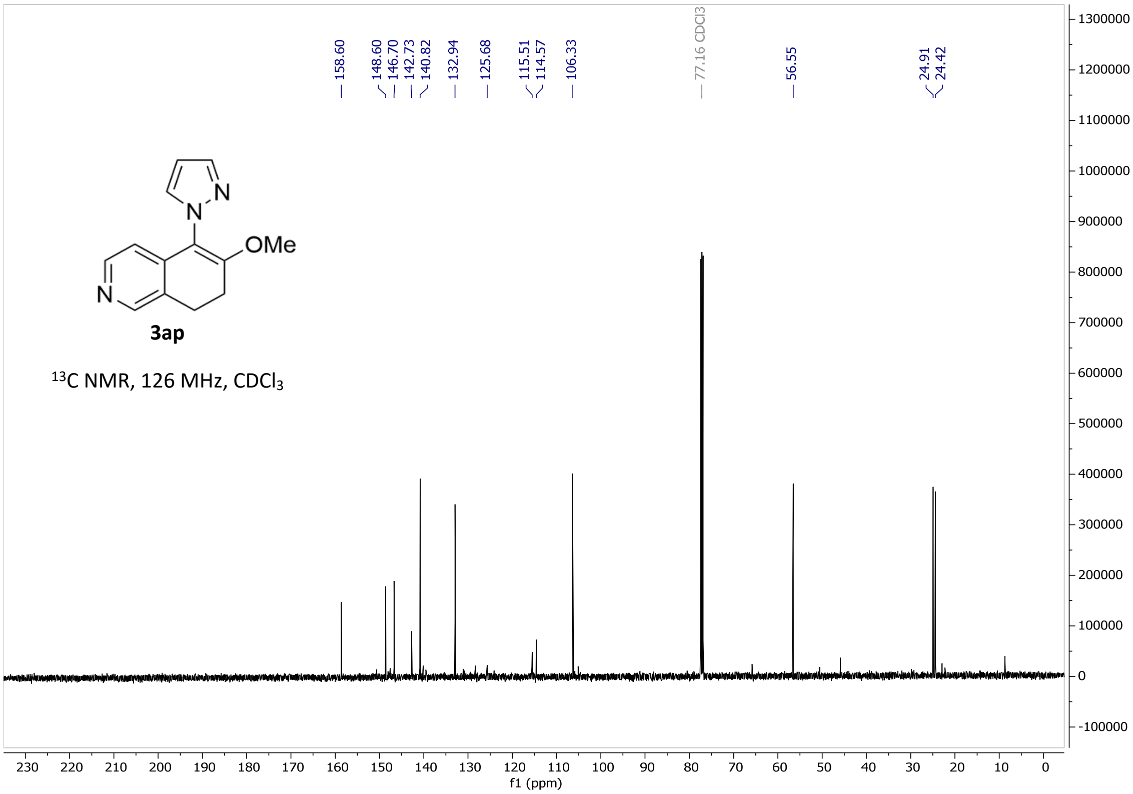

Supplementary Figure 113. ^{13}C NMR spectra of **3ap** (126 MHz, rt, $CDCl_3$).

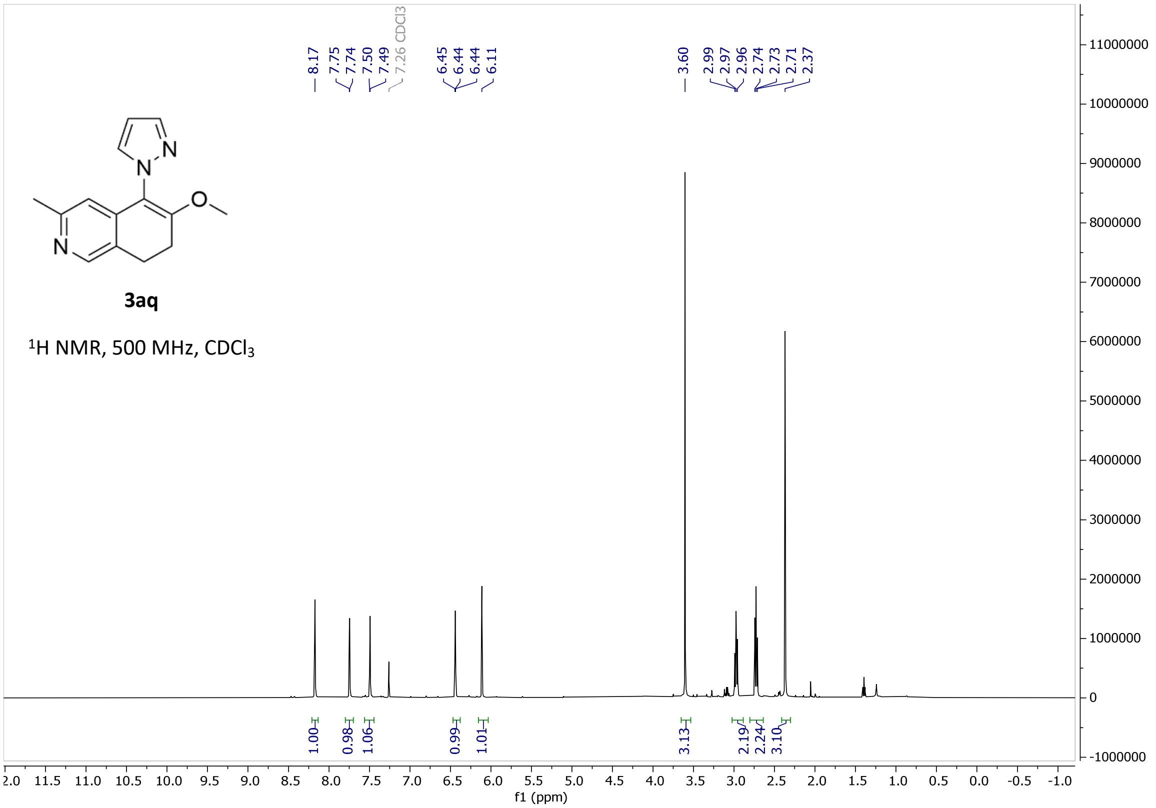

Supplementary Figure 114. ^{1}H NMR spectra of **3aq** (500 MHz, rt, $CDCl_3$).

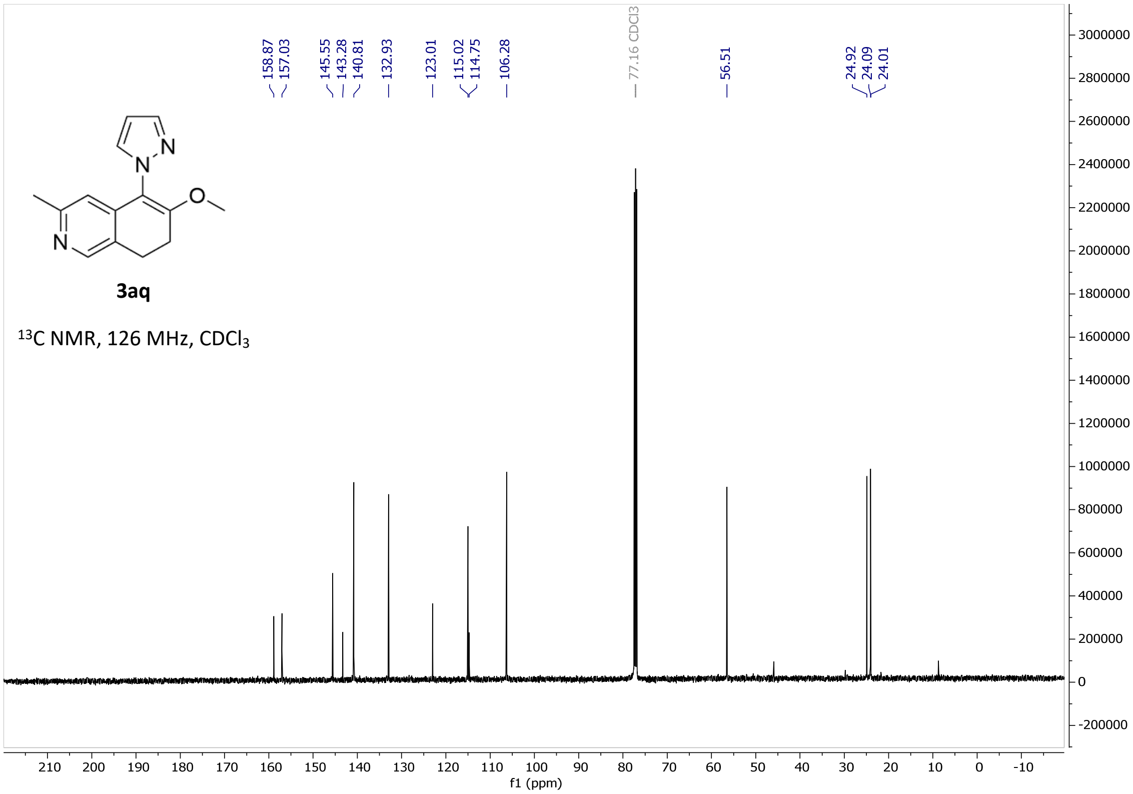

Supplementary Figure 115. ^{13}C NMR spectra of **3aq** (126 MHz, rt, $CDCl_3$).

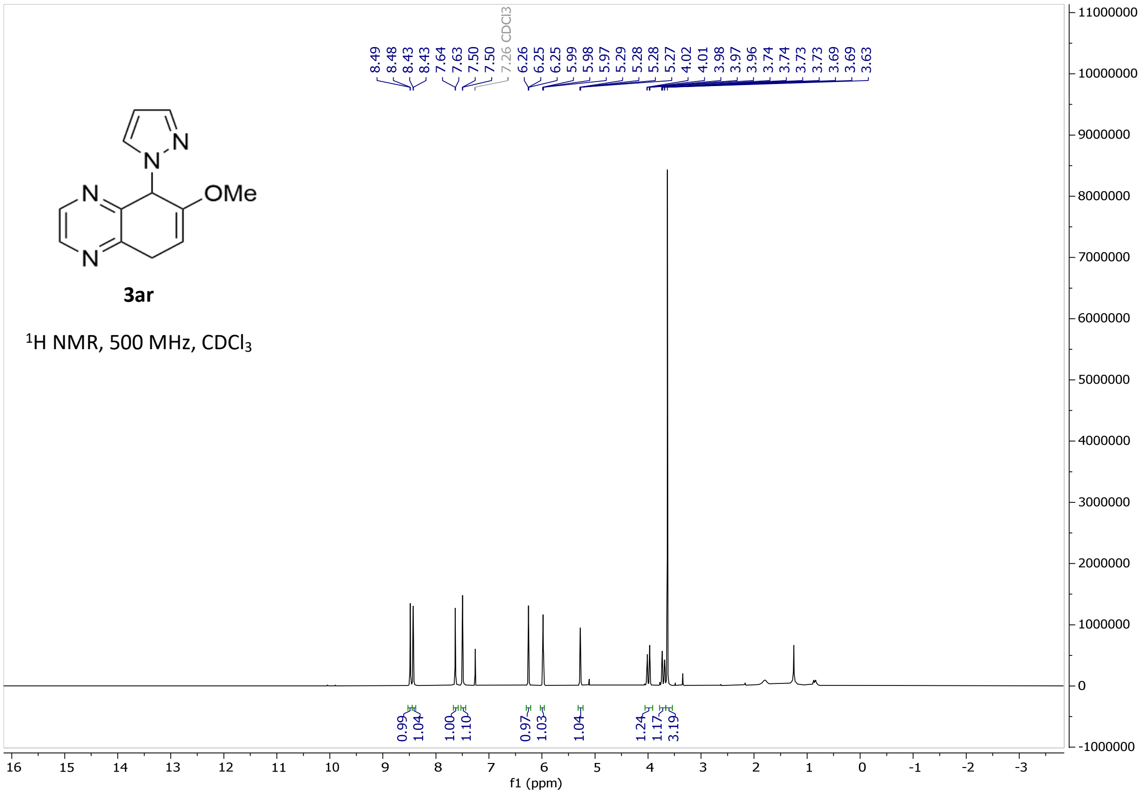

Supplementary Figure 116. ^{1}H NMR spectra of **3ar** (500 MHz, rt, $CDCl_3$).

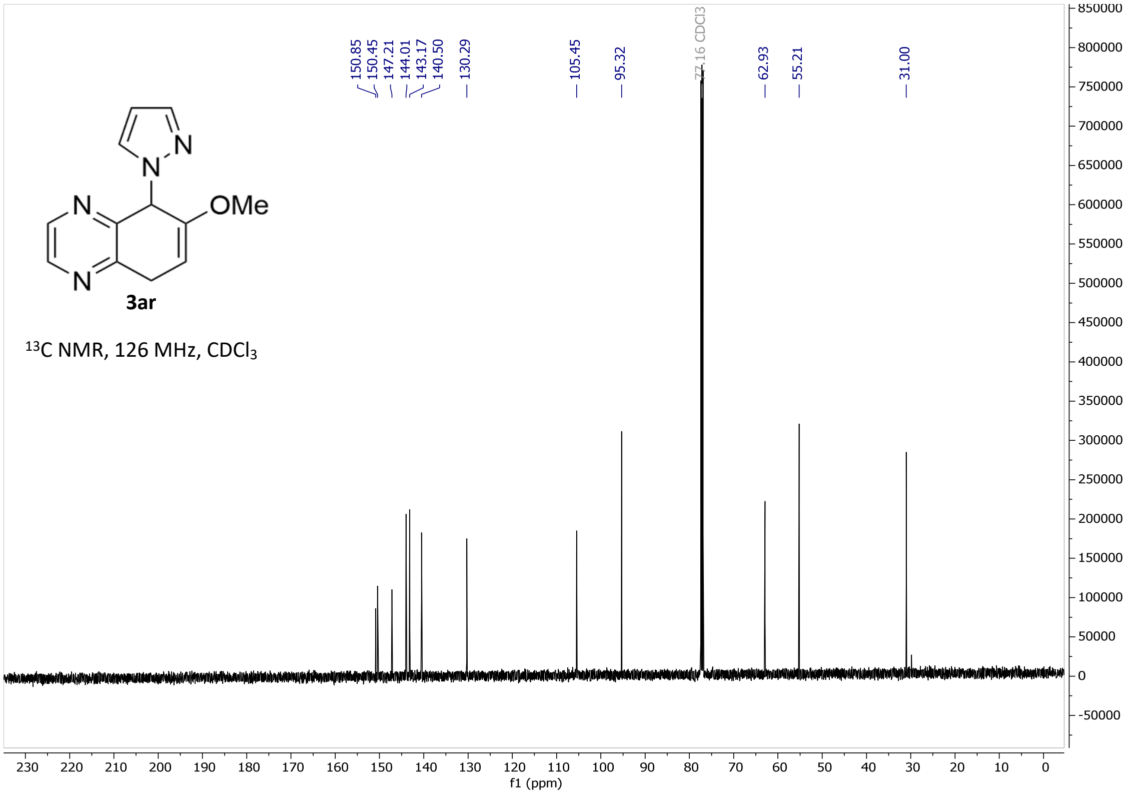

Supplementary Figure 117. ^{13}C NMR spectra of **3ar** (126 MHz, rt, $CDCl_3$).

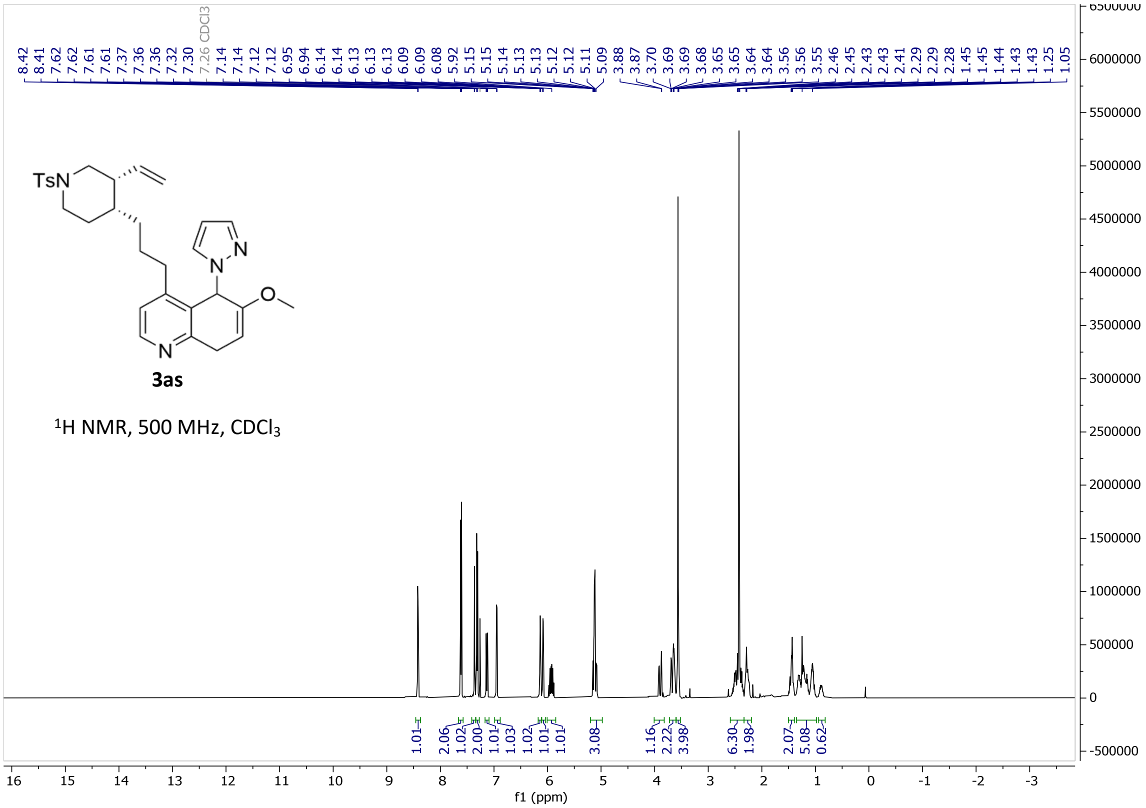

Supplementary Figure 118. ^{1}H NMR spectra of **3as** (500 MHz, rt, $CDCl_3$).

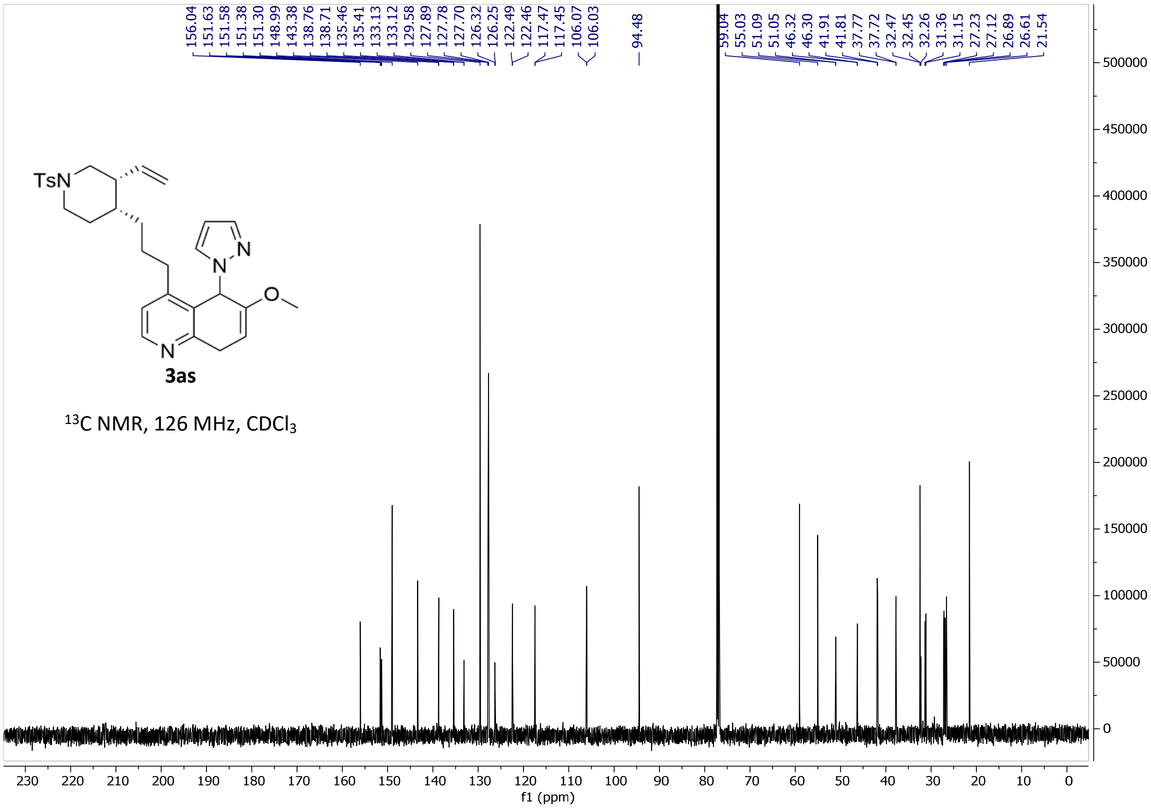

Supplementary Figure 119. ^{13}C NMR spectra of **3as** (126 MHz, rt, $CDCl_3$).

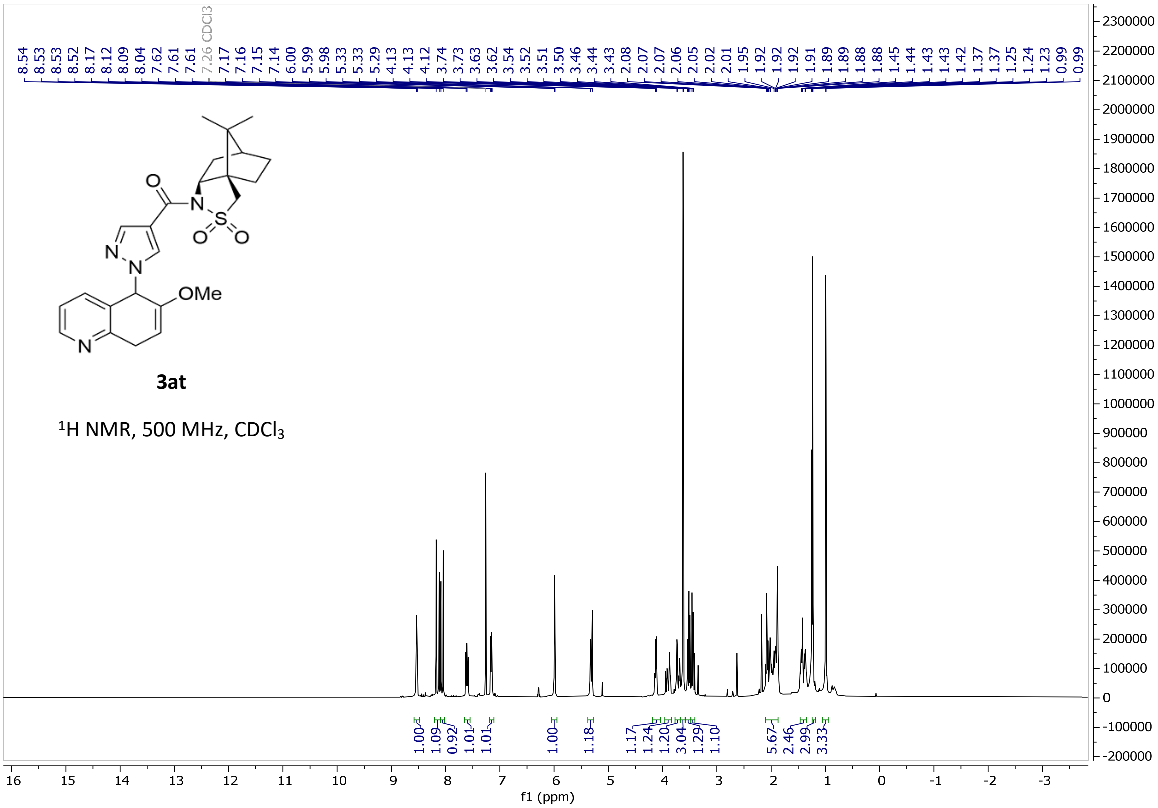

Supplementary Figure 120. ^{1}H NMR spectra of **3at** (500 MHz, rt, $CDCl_3$).

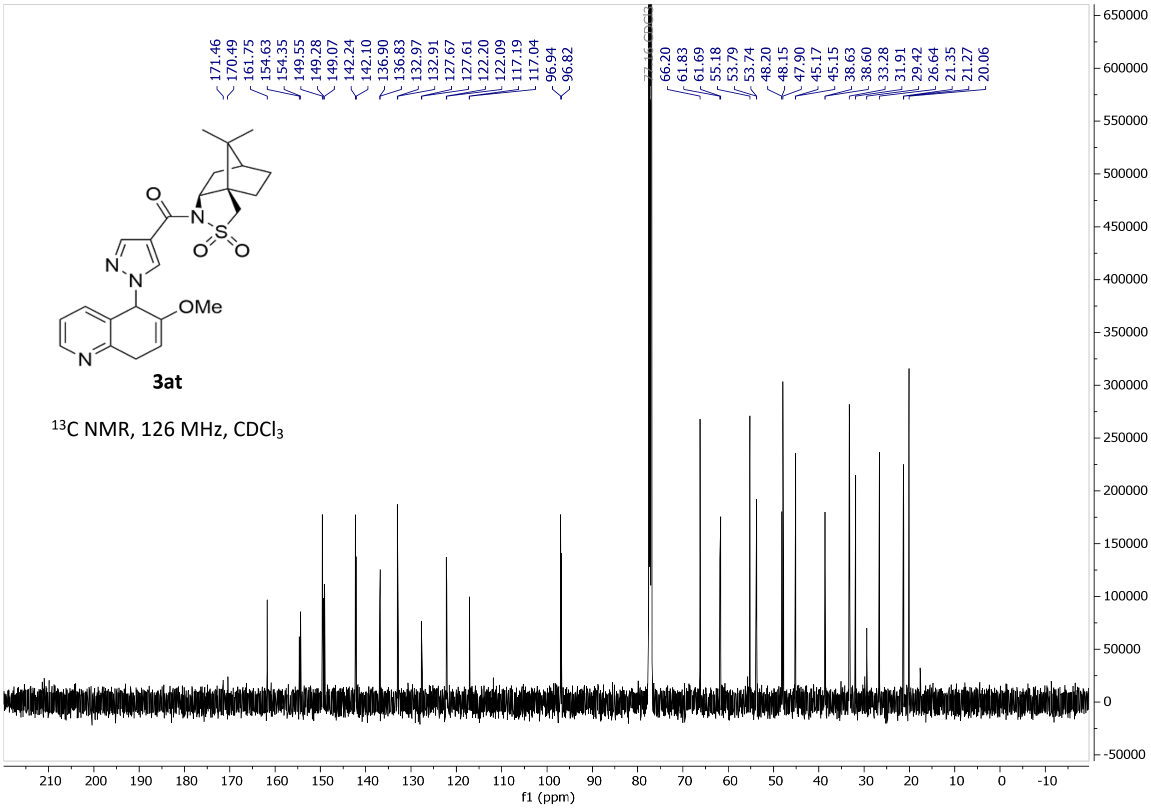

Supplementary Figure 121. ^{13}C NMR spectra of **3at** (126 MHz, rt, $CDCl_3$).

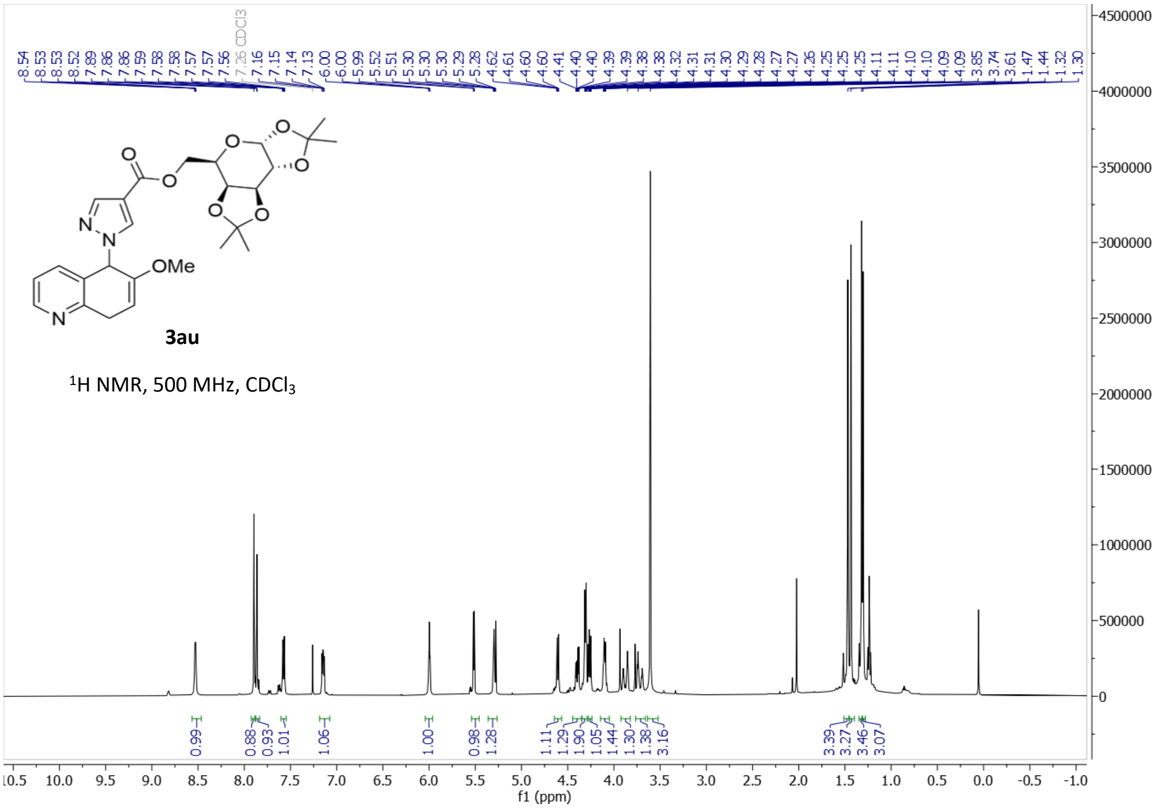

Supplementary Figure 122. ^{1}H NMR spectra of **3au** (500 MHz, rt, $CDCl_3$).

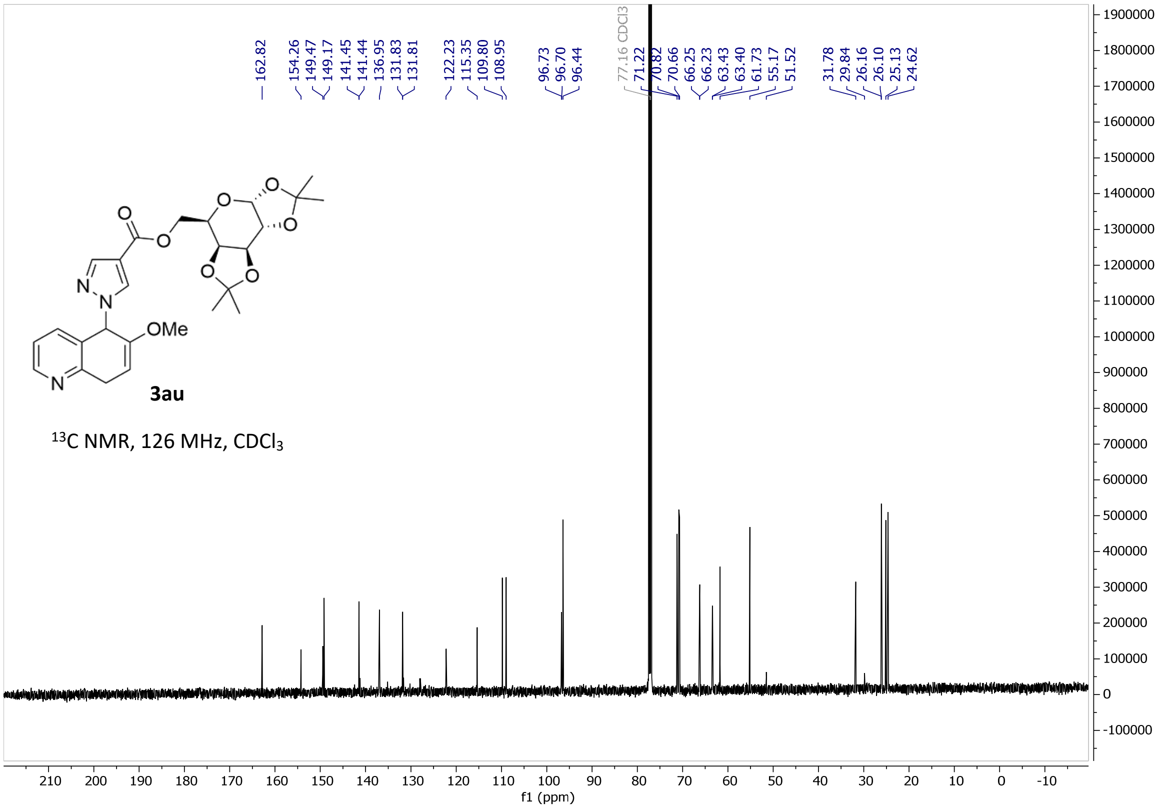

Supplementary Figure 123. ^{13}C NMR spectra of **3au** (126 MHz, rt, $CDCl_3$).

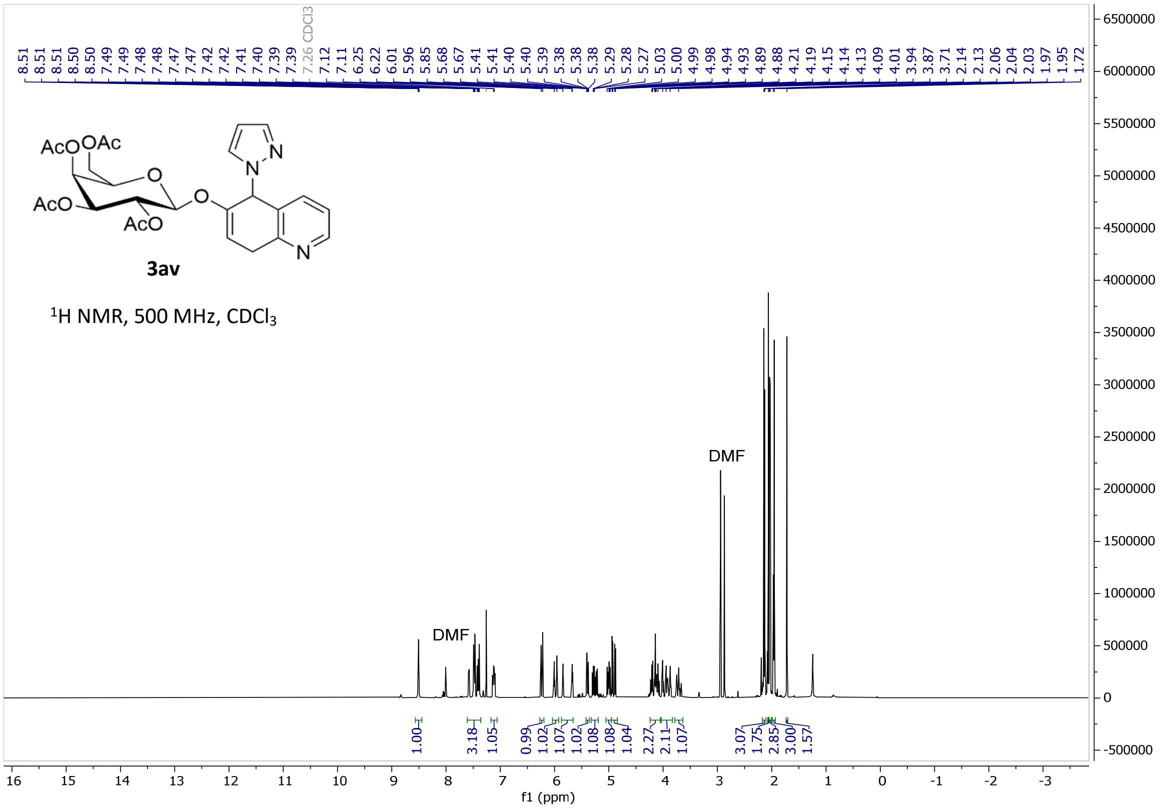

Supplementary Figure 124. ^{1}H NMR spectra of **3av** (500 MHz, rt, $CDCl_3$).

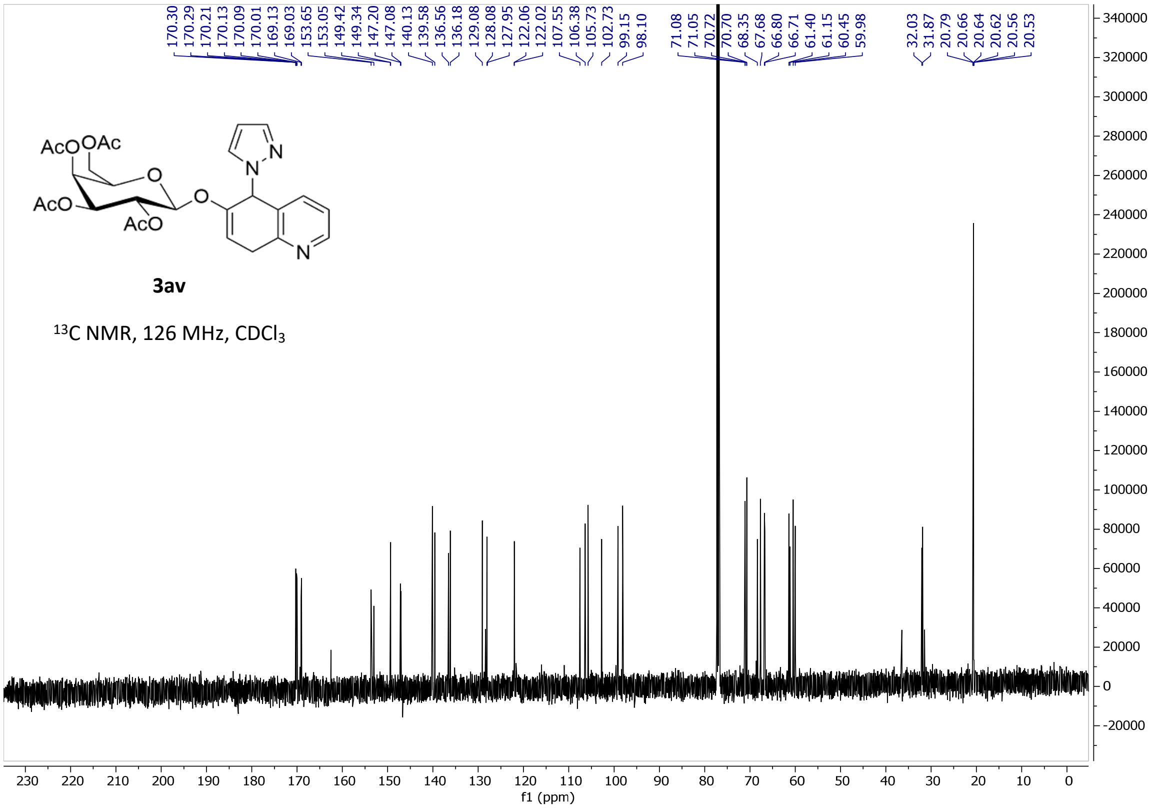

Supplementary Figure 125. ^{13}C NMR spectra of **3av** (126 MHz, rt, $CDCl_3$).

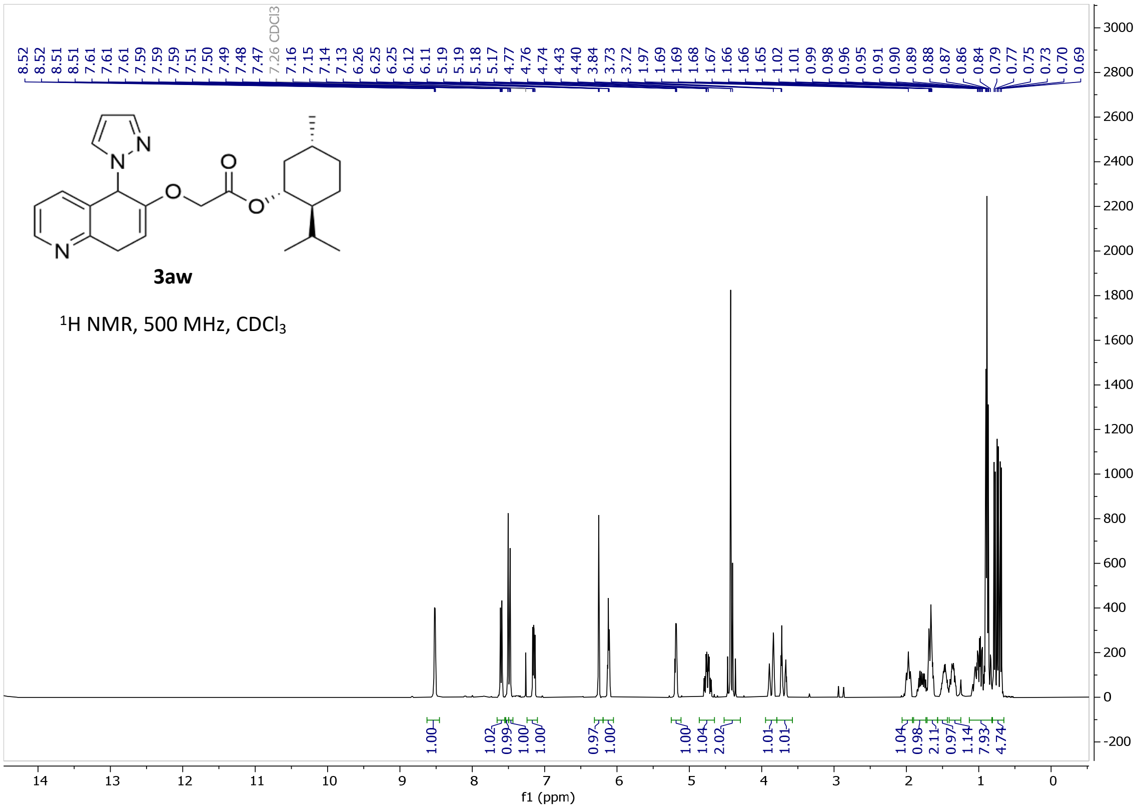

Supplementary Figure 126. ^{1}H NMR spectra of **3aw** (500 MHz, rt, $CDCl_3$).

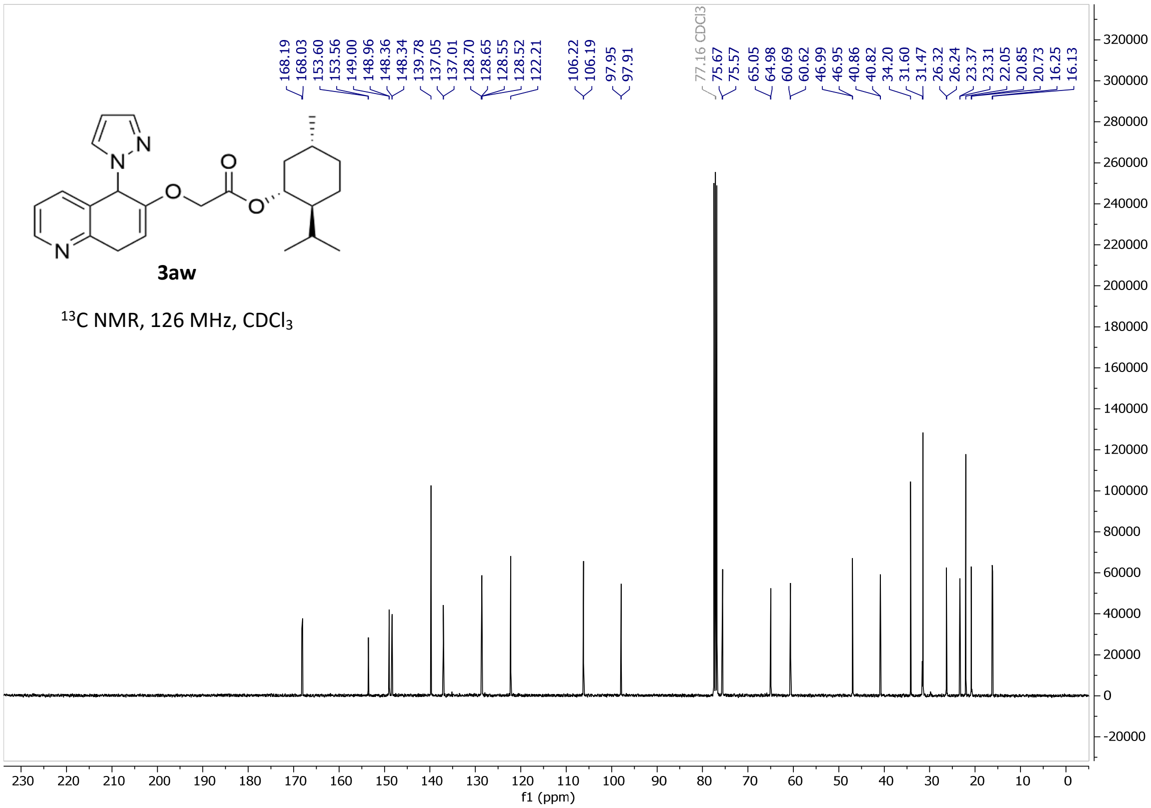

Supplementary Figure 127. ^{13}C NMR spectra of **3aw** (126 MHz, rt, $CDCl_3$).

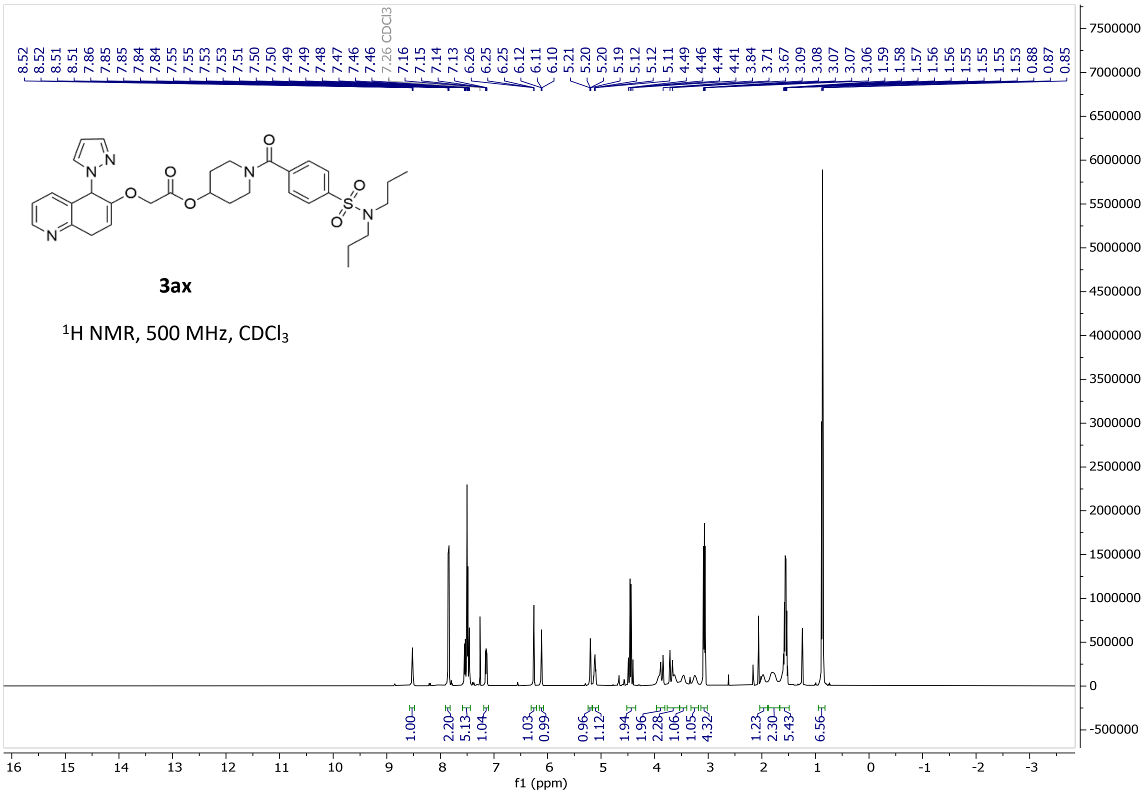

Supplementary Figure 128. ^{1}H NMR spectra of **3ax** (500 MHz, rt, $CDCl_3$).

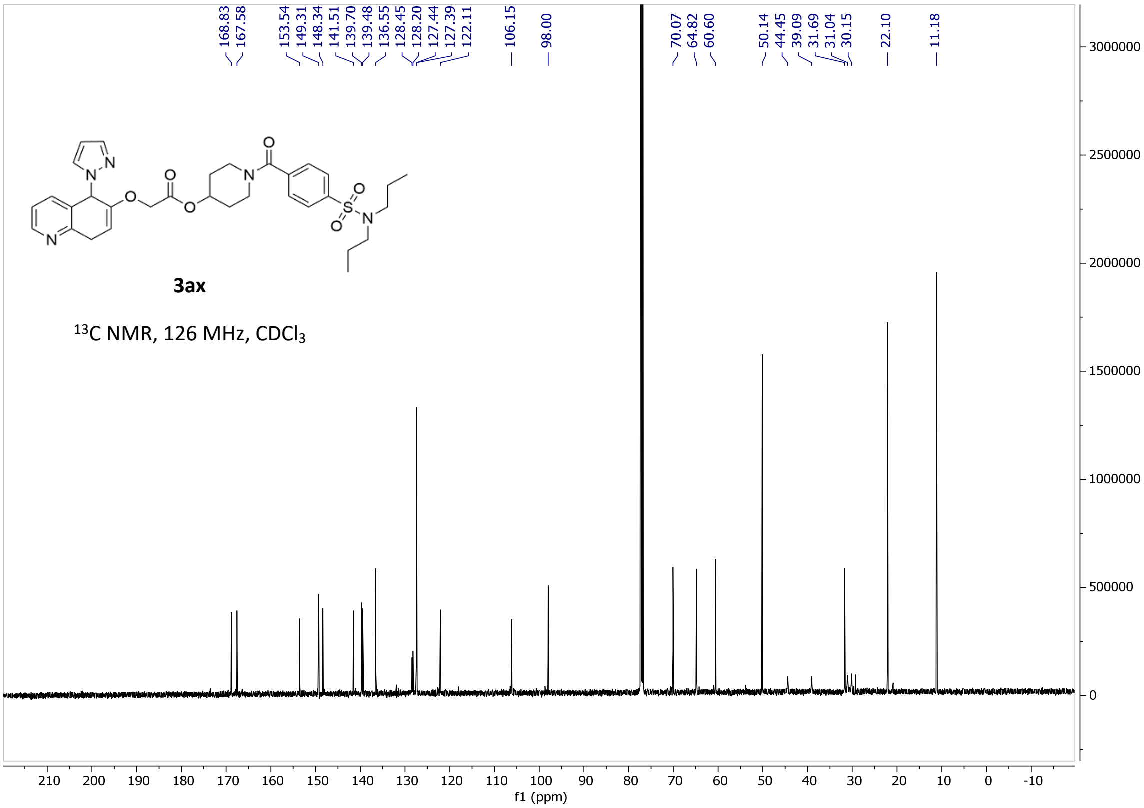

Supplementary Figure 129. ^{13}C NMR spectra of **3ax** (126 MHz, rt, $CDCl_3$).

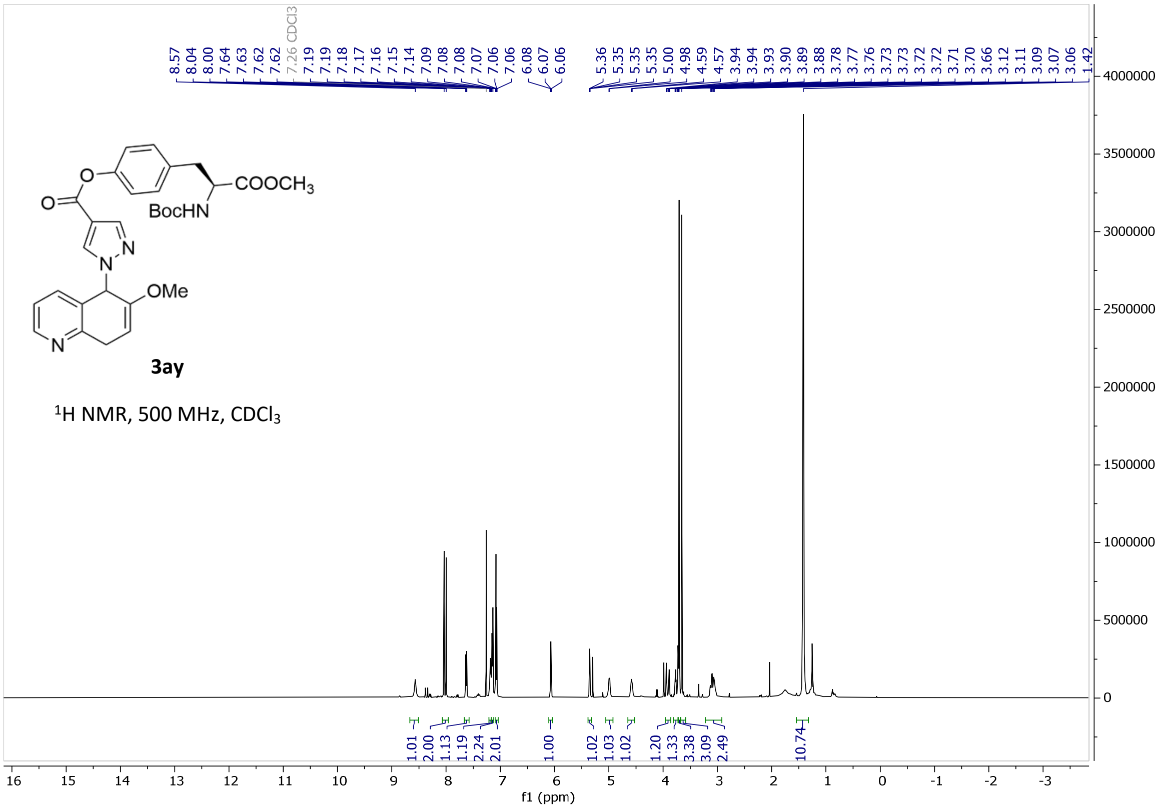

Supplementary Figure 130. ^{1}H NMR spectra of **3ay** (500 MHz, rt, $CDCl_3$).

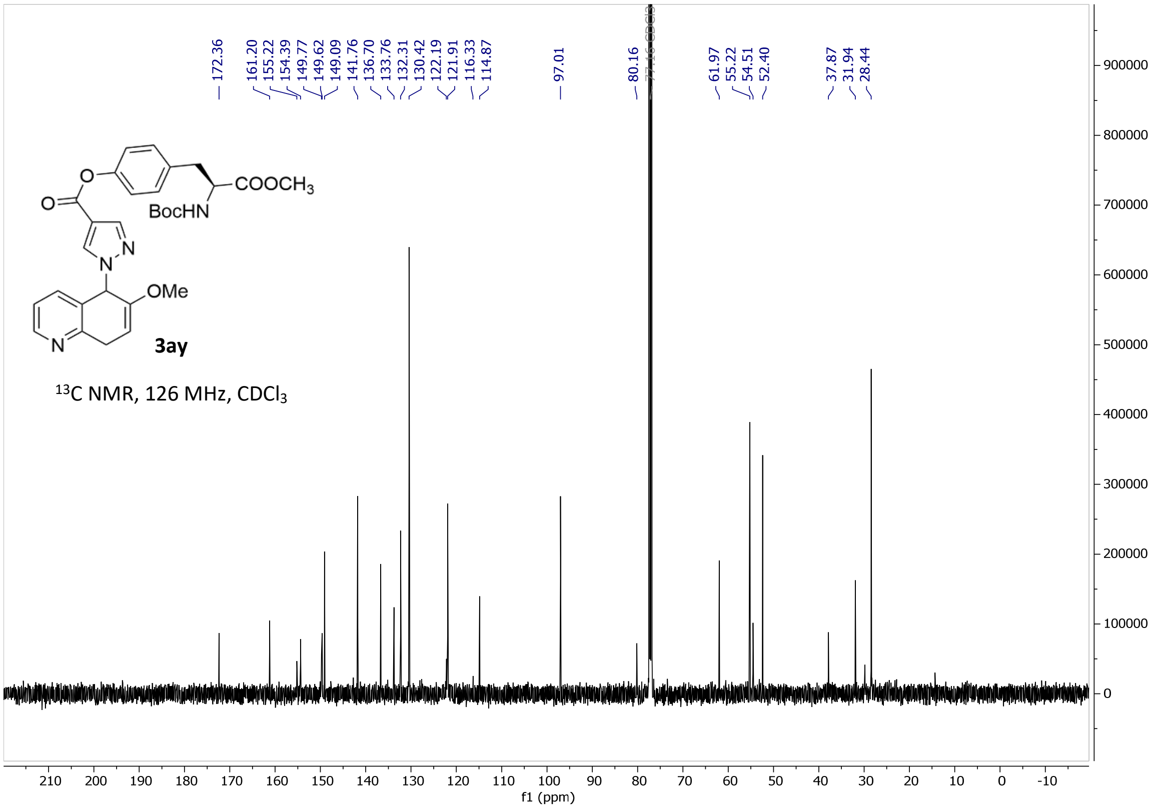

Supplementary Figure 131. ^{13}C NMR spectra of **3ay** (126 MHz, rt, $CDCl_3$).

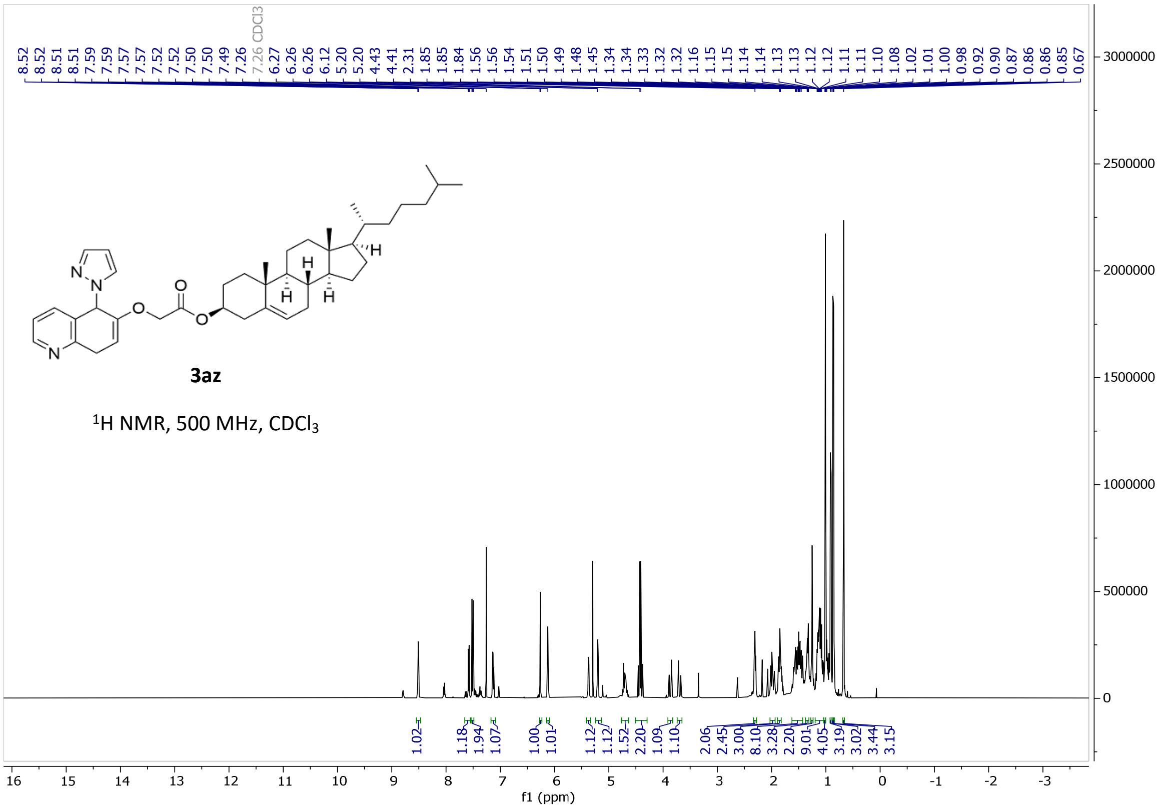

Supplementary Figure 132. ^{1}H NMR spectra of **3az** (500 MHz, rt, $CDCl_3$).

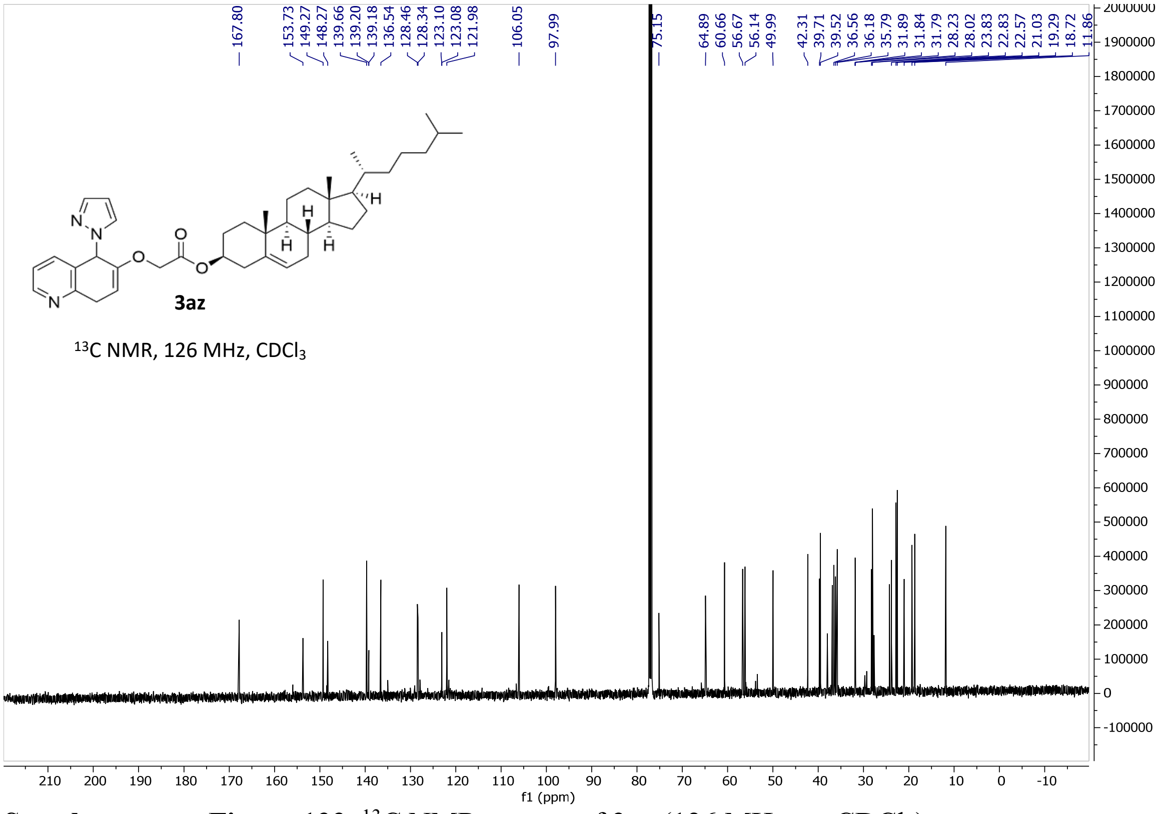

Supplementary Figure 133. ^{13}C NMR spectra of **3az** (126 MHz, rt, $CDCl_3$).

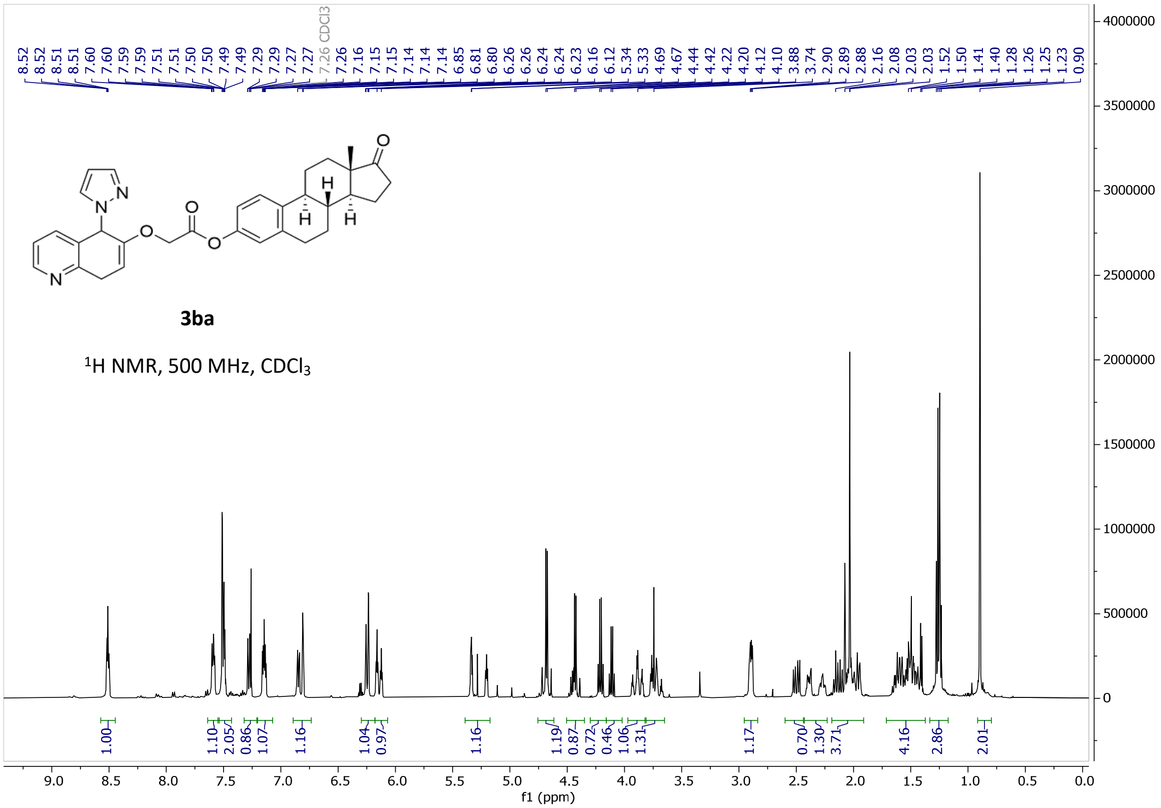

Supplementary Figure 134. ^{1}H NMR spectra of **3ba** (500 MHz, rt, $CDCl_3$).

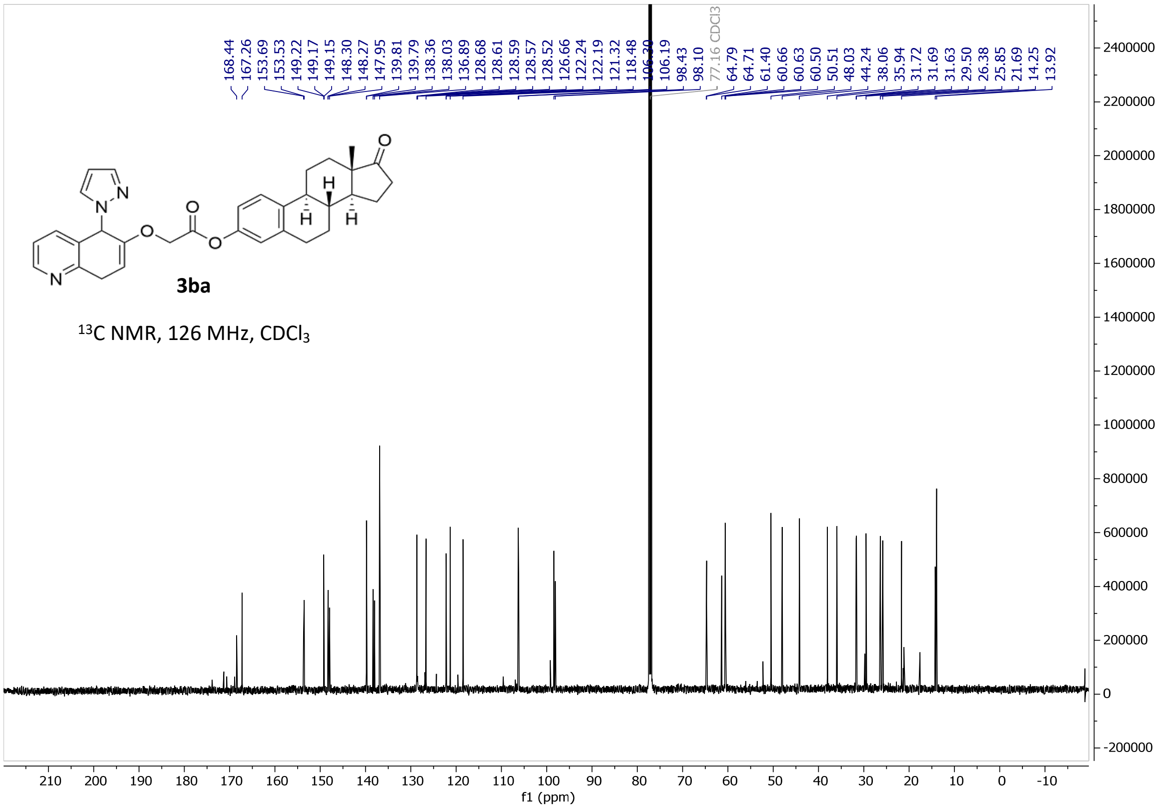

Supplementary Figure 135. ^{13}C NMR spectra of **3ba** (126 MHz, rt, $CDCl_3$).

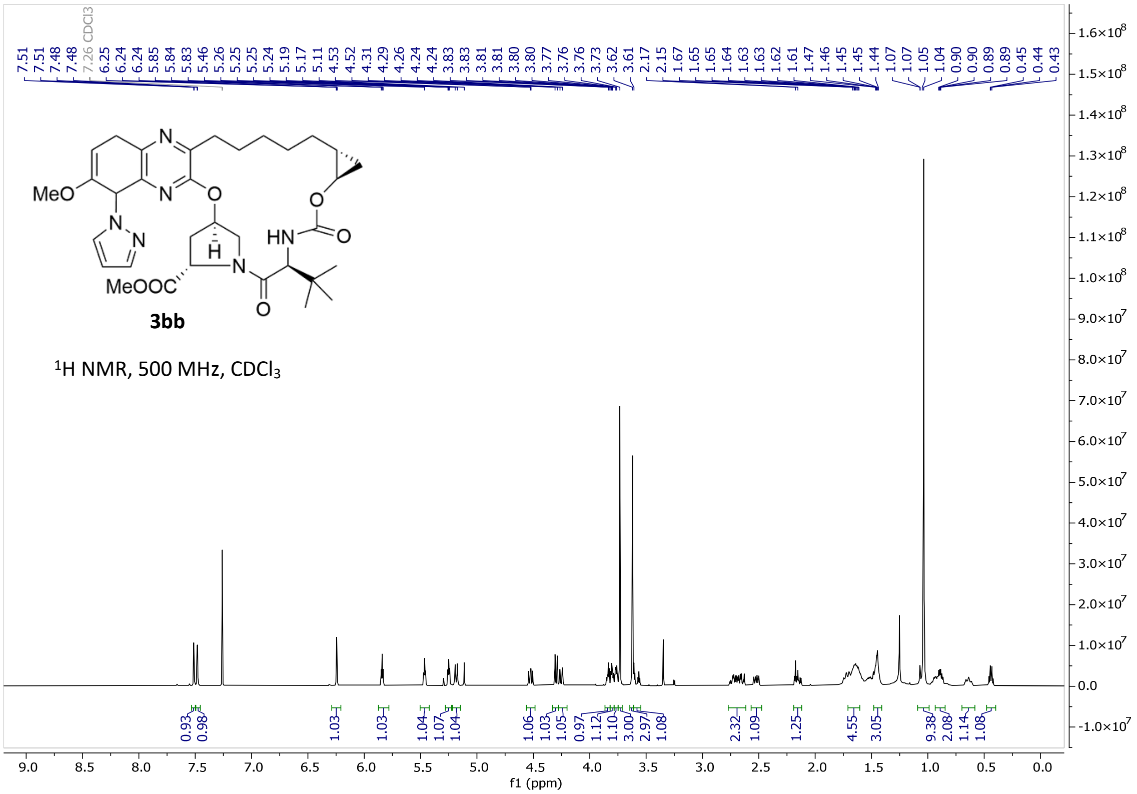

Supplementary Figure 136. ^{1}H NMR spectra of **3bb** (500 MHz, rt, $CDCl_3$).

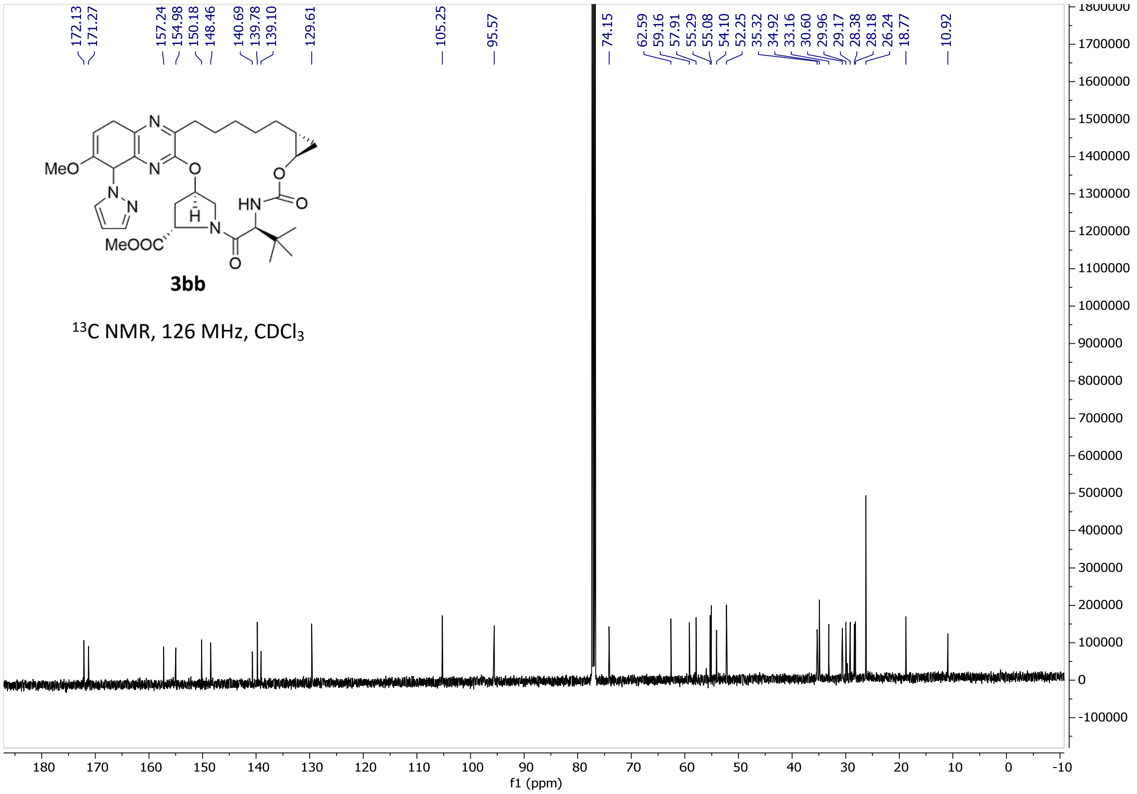

Supplementary Figure 137. ^{13}C NMR spectra of **3bb** (126 MHz, rt, $CDCl_3$).

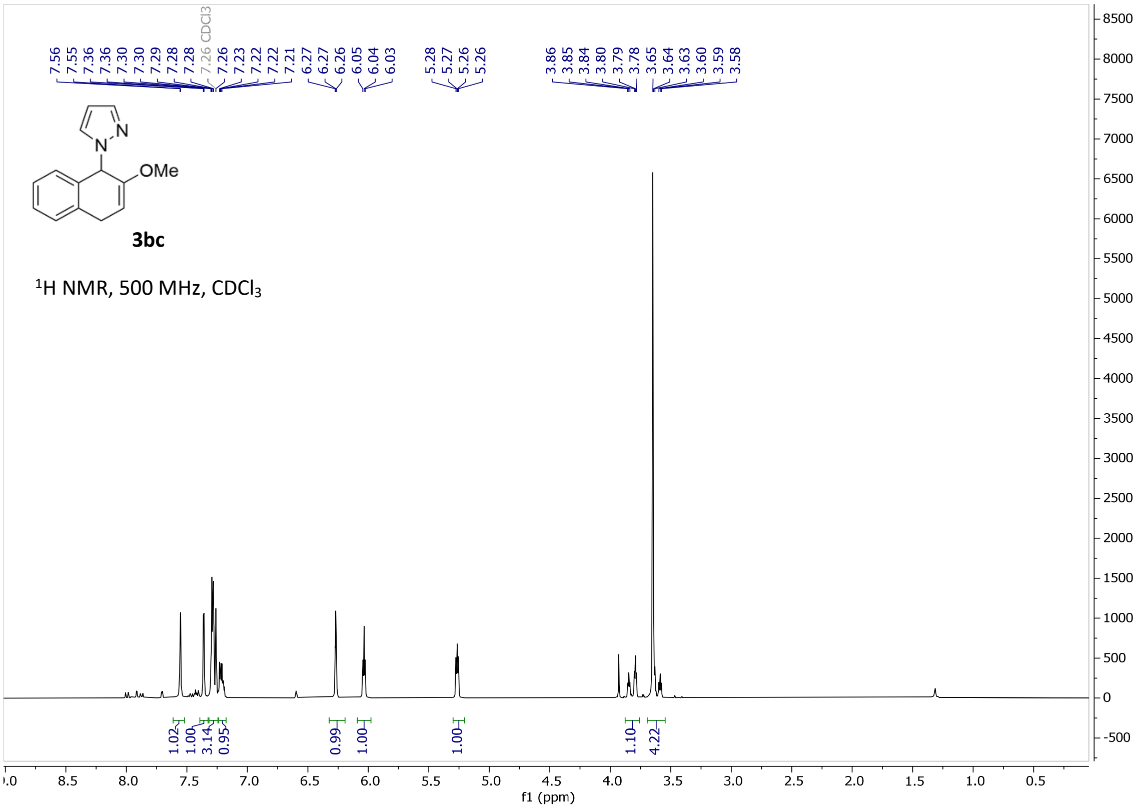

Supplementary Figure 138. ^{1}H NMR spectra of **3bc** (500 MHz, rt, CDCl$_3$).

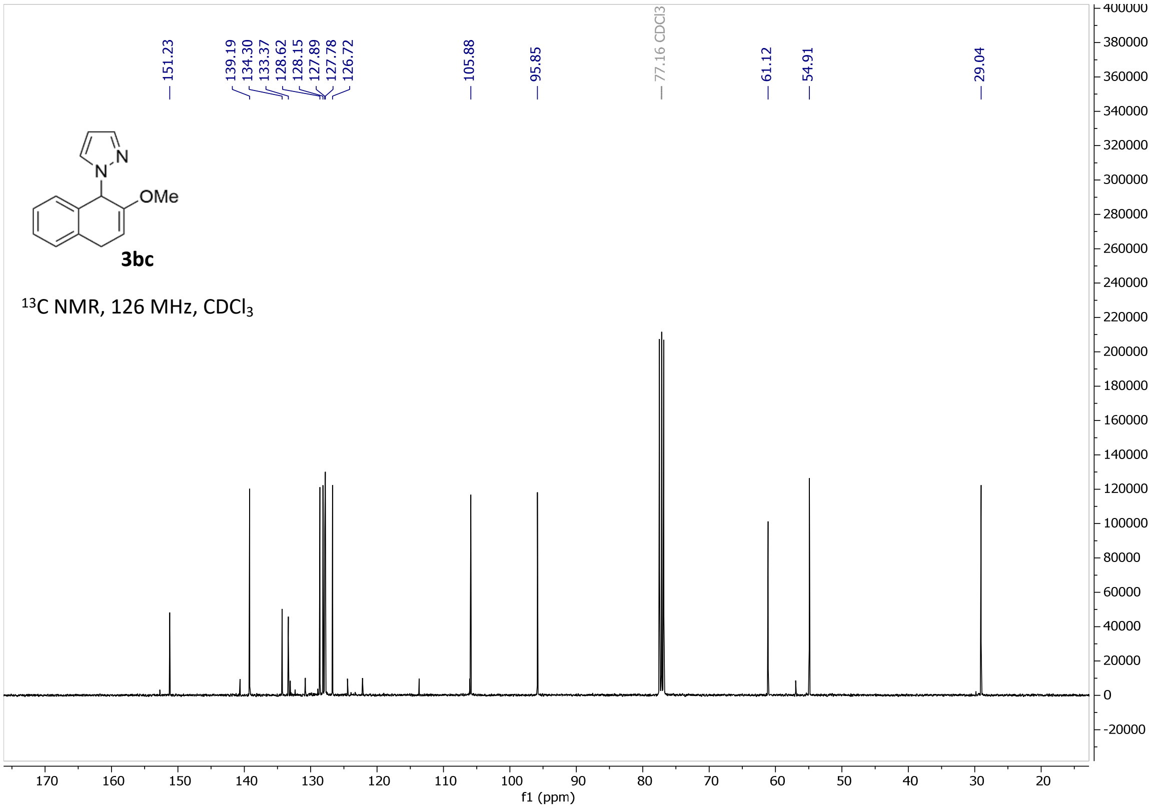

Supplementary Figure 139. ^{13}C NMR spectra of **3bc** (126 MHz, rt, CDCl$_3$).

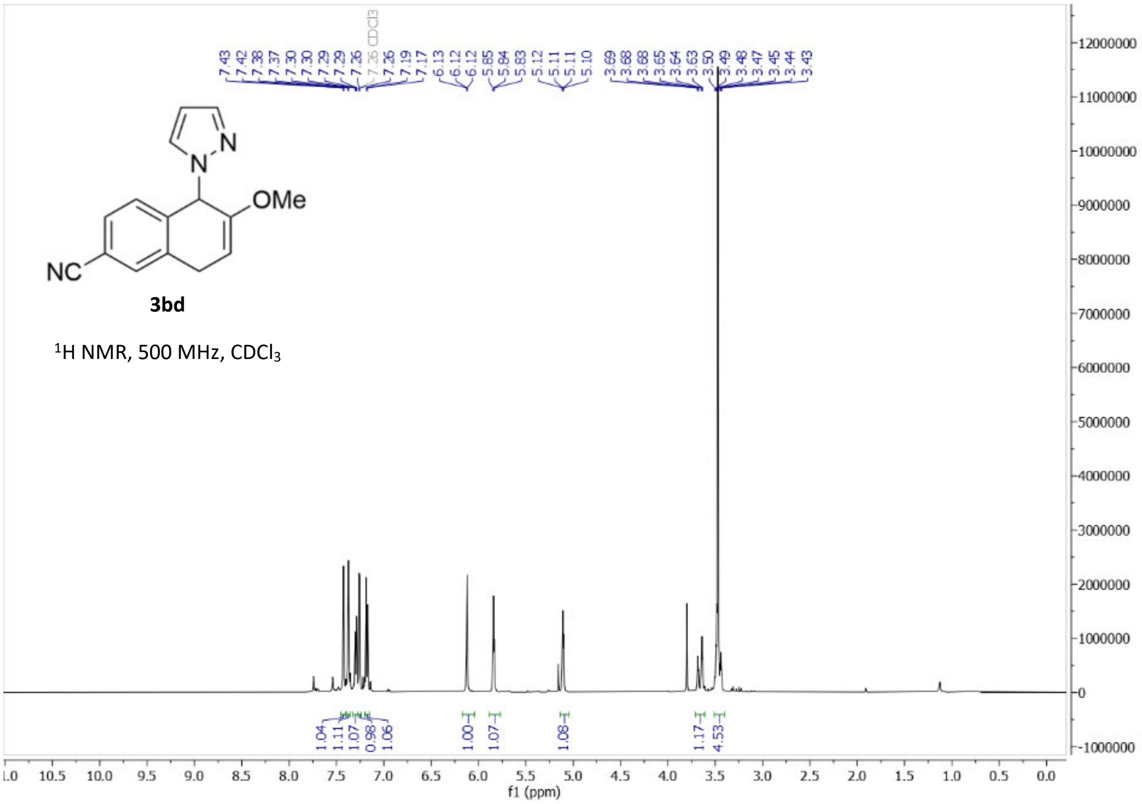

Supplementary Figure 140. ^1H NMR spectra of **3bd** (500 MHz, rt, $CDCl_3$).

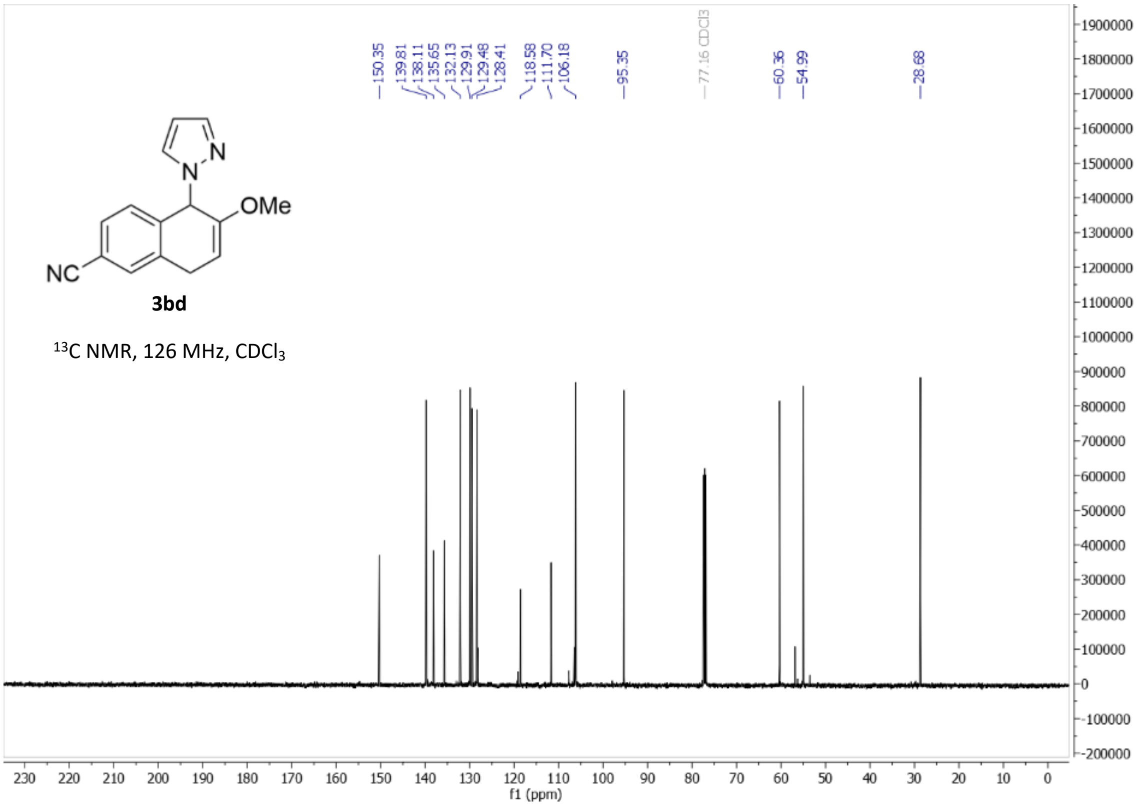

Supplementary Figure 141. ^{13}C NMR spectra of **3bd** (126 MHz, rt, $CDCl_3$).

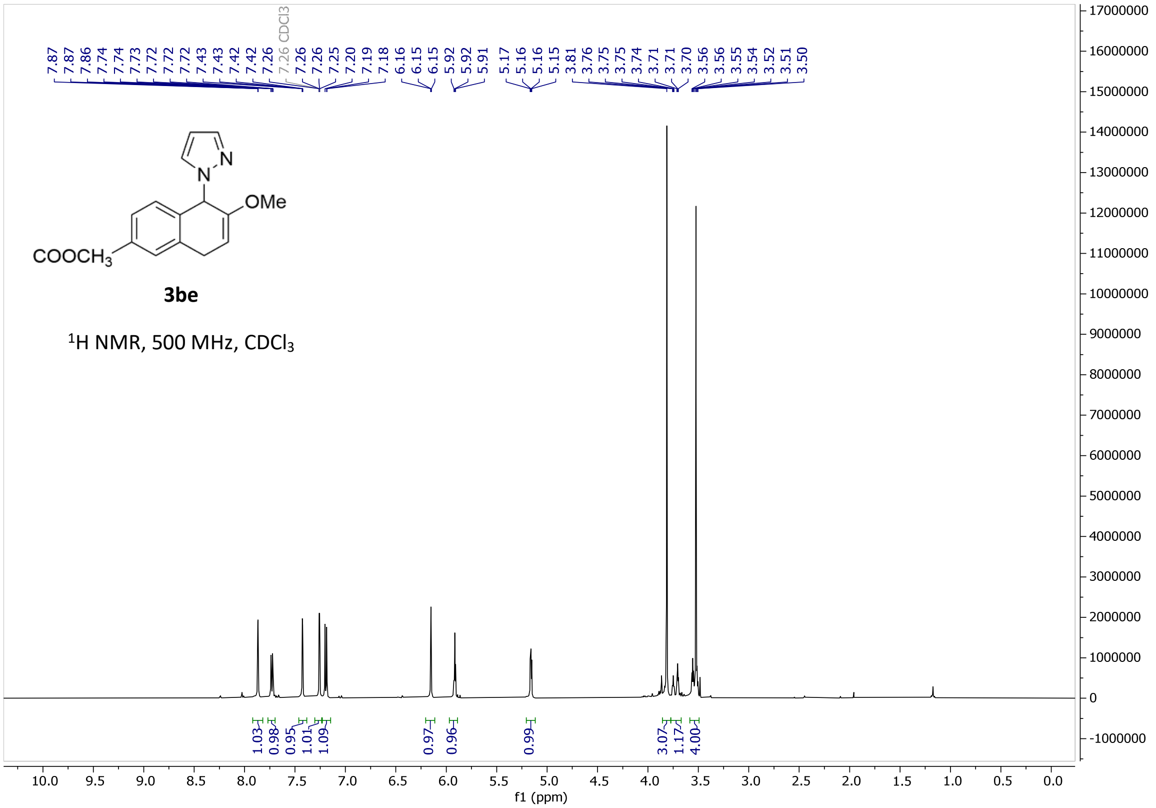

Supplementary Figure 142. ^{1}H NMR spectra of **3be** (500 MHz, rt, $CDCl_3$).

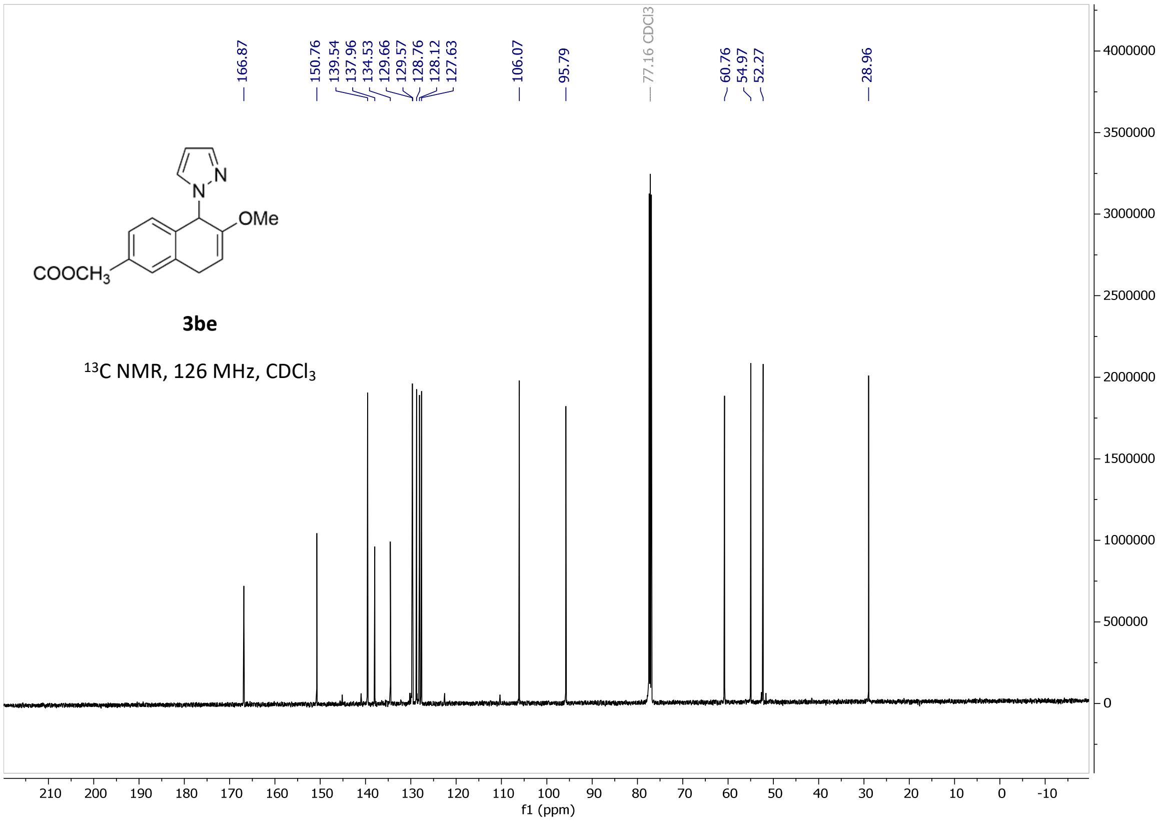

Supplementary Figure 143. ^{13}C NMR spectra of **3be** (126 MHz, rt, $CDCl_3$).

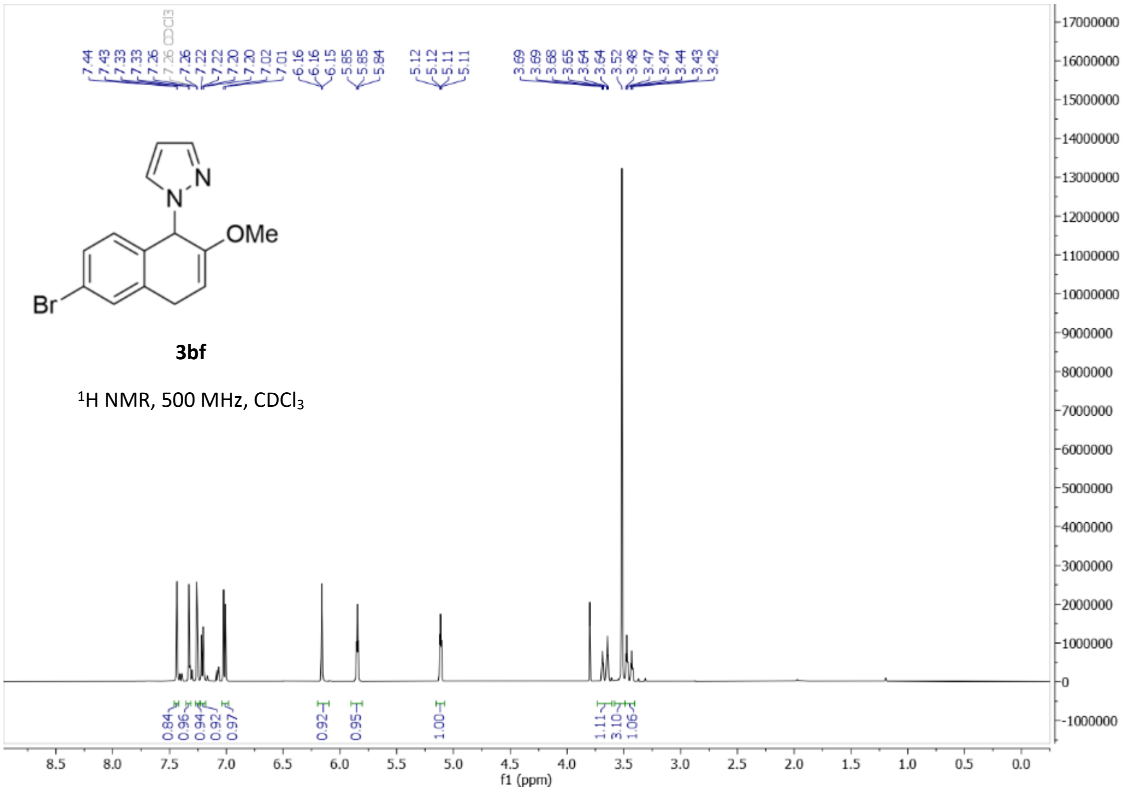

Supplementary Figure 144. ^{1}H NMR spectra of **3bf** (500 MHz, rt, $CDCl_3$).

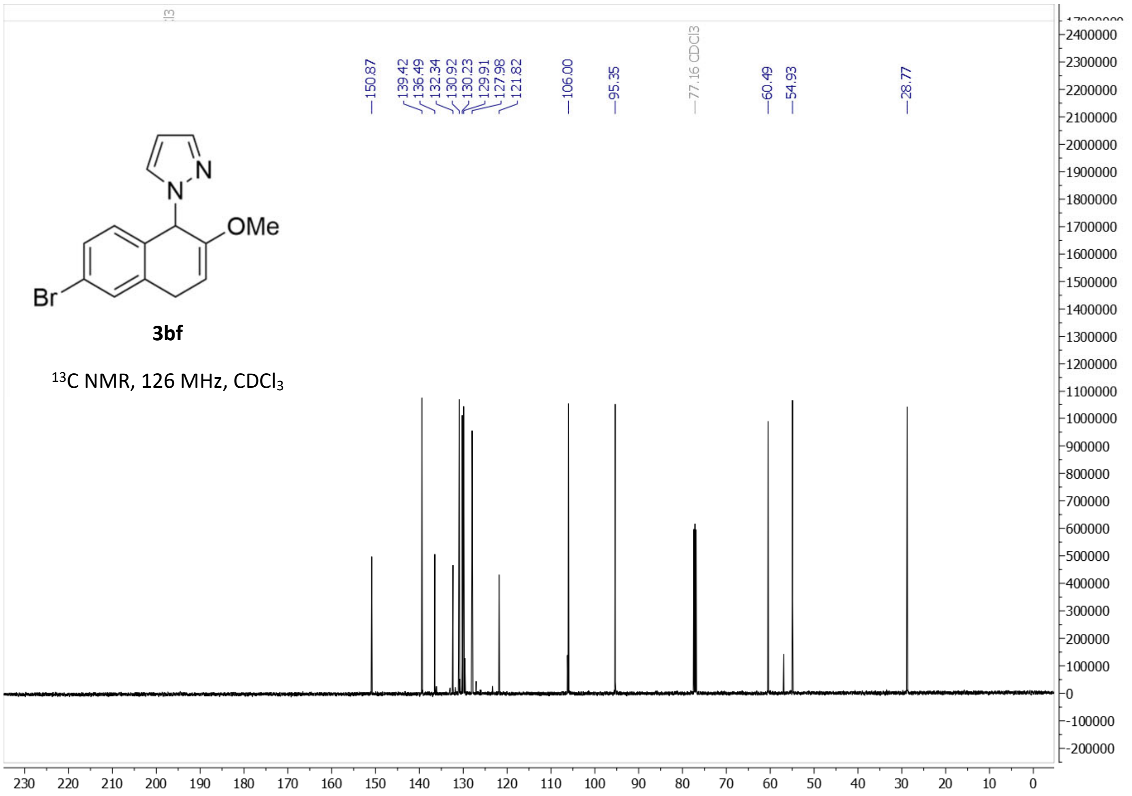

Supplementary Figure 145. ^{13}C NMR spectra of **3bf** (126 MHz, rt, $CDCl_3$).

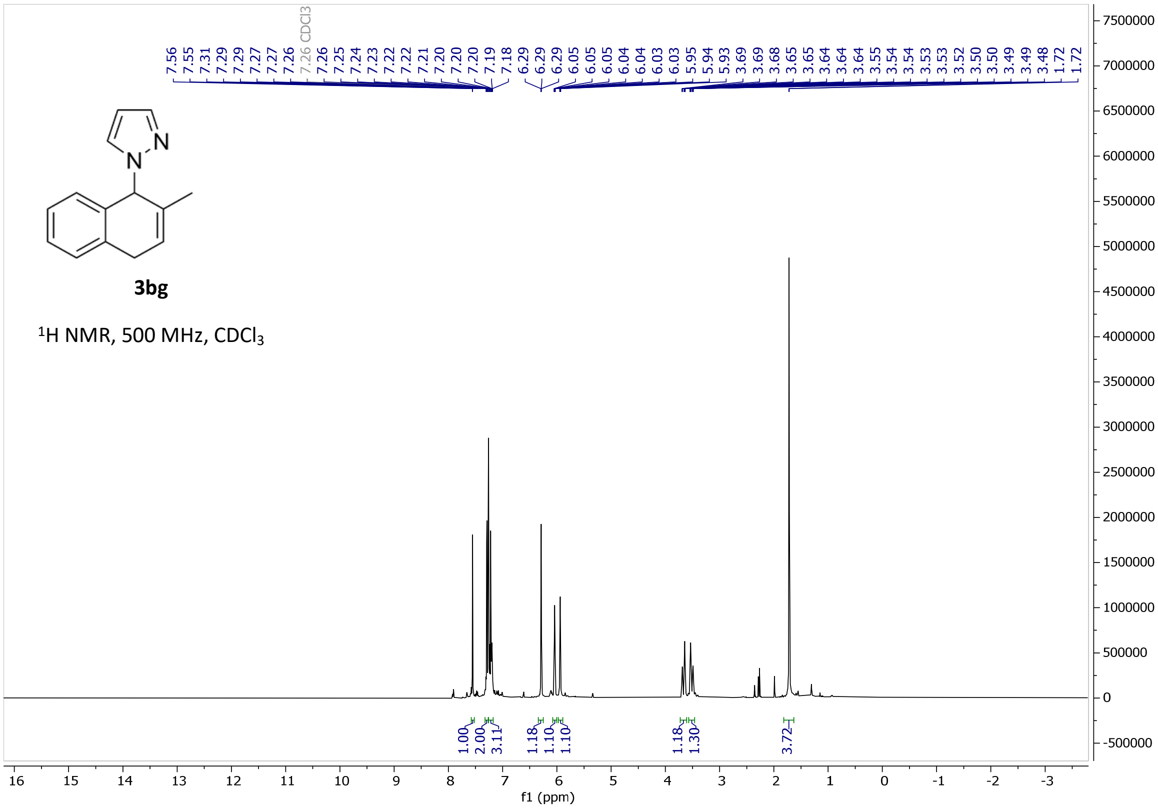

Supplementary Figure 146. ^{1}H NMR spectra of **3bg** (500 MHz, rt, CDCl$_3$).

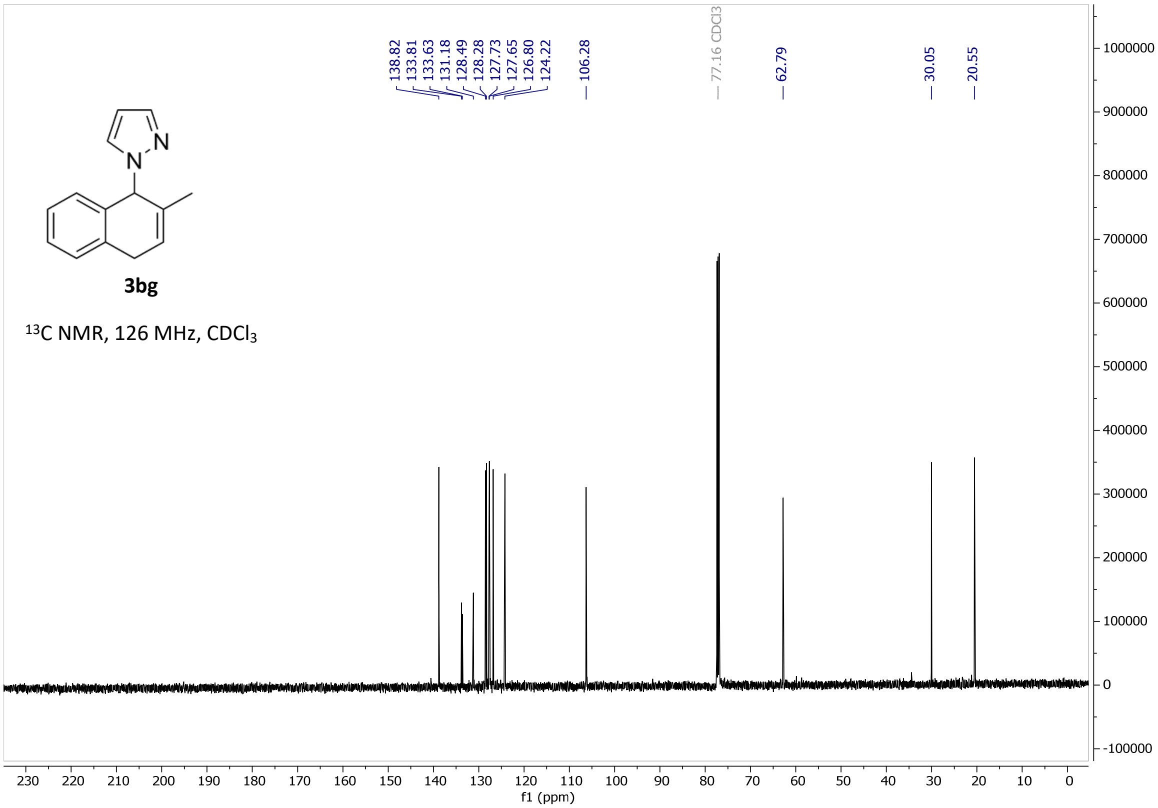

Supplementary Figure 147. ^{13}C NMR spectra of **3bg** (126 MHz, rt, CDCl$_3$).

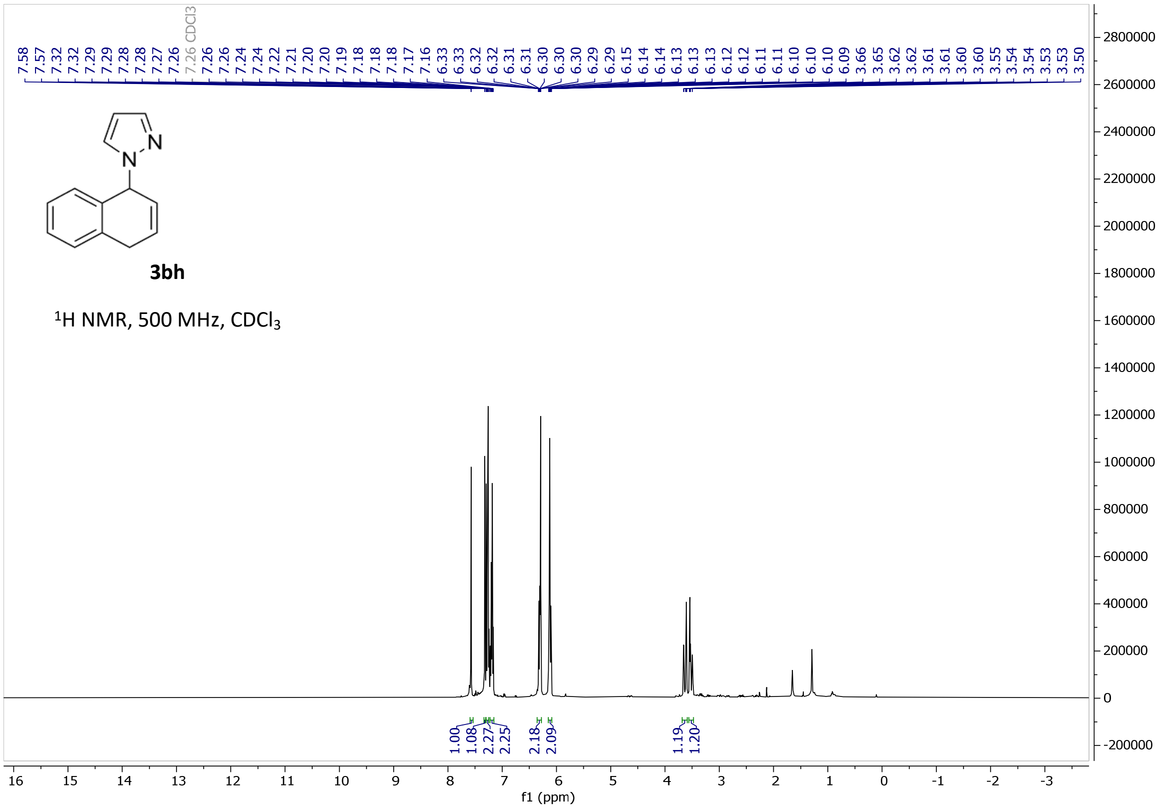

Supplementary Figure 148. ^{1}H NMR spectra of **3bh** (500 MHz, rt, $CDCl_3$).

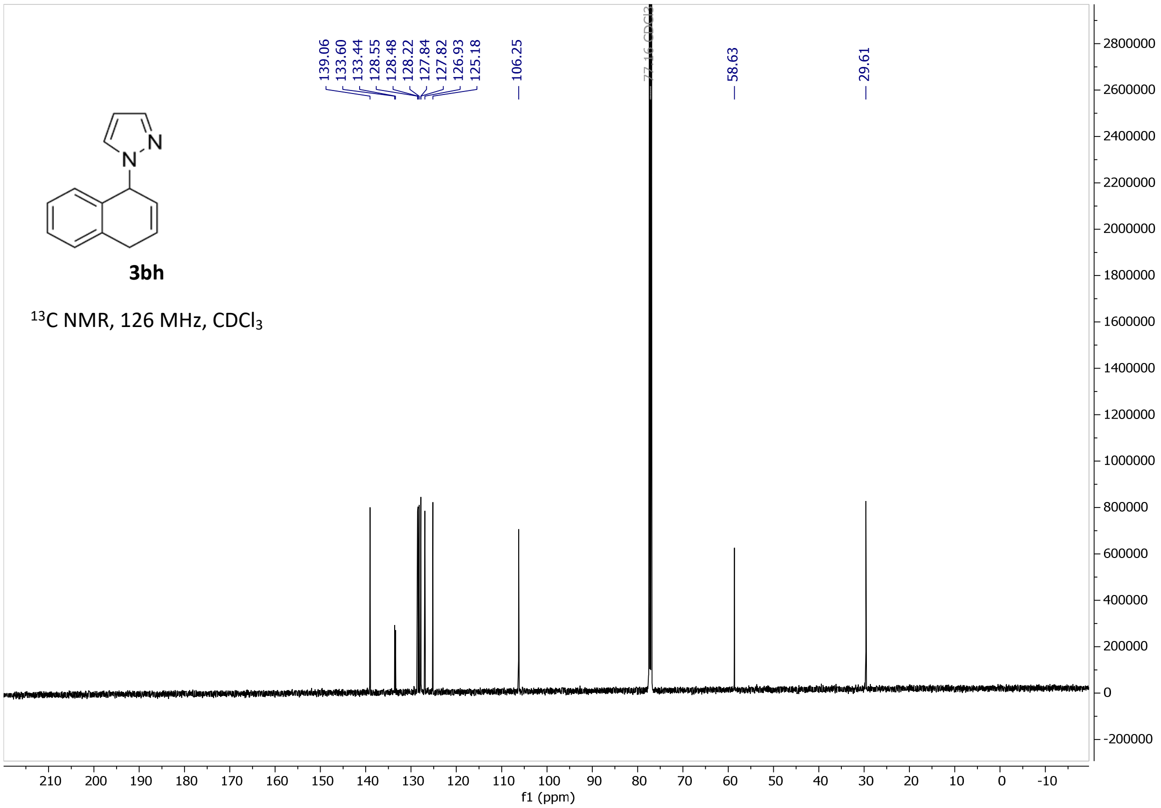

Supplementary Figure 149. ^{13}C NMR spectra of **3bh** (126 MHz, rt, $CDCl_3$).

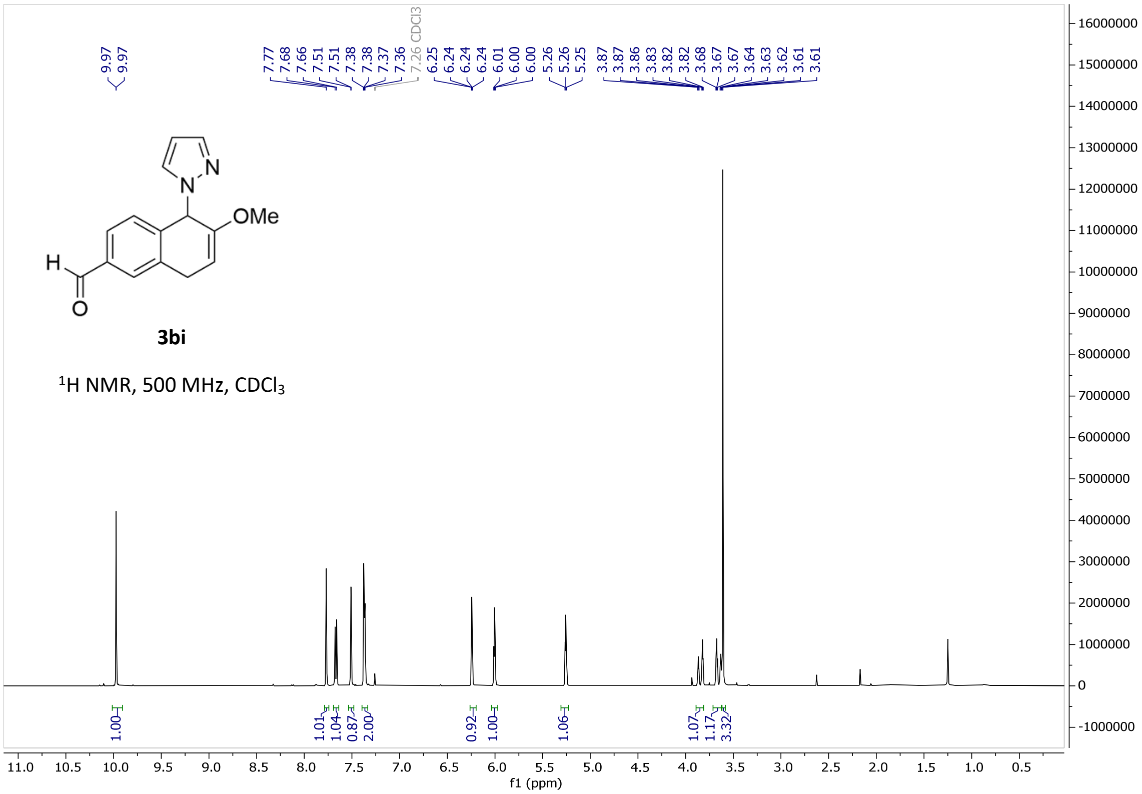

Supplementary Figure 150. ^{1}H NMR spectra of **3bi** (500 MHz, rt, $CDCl_3$).

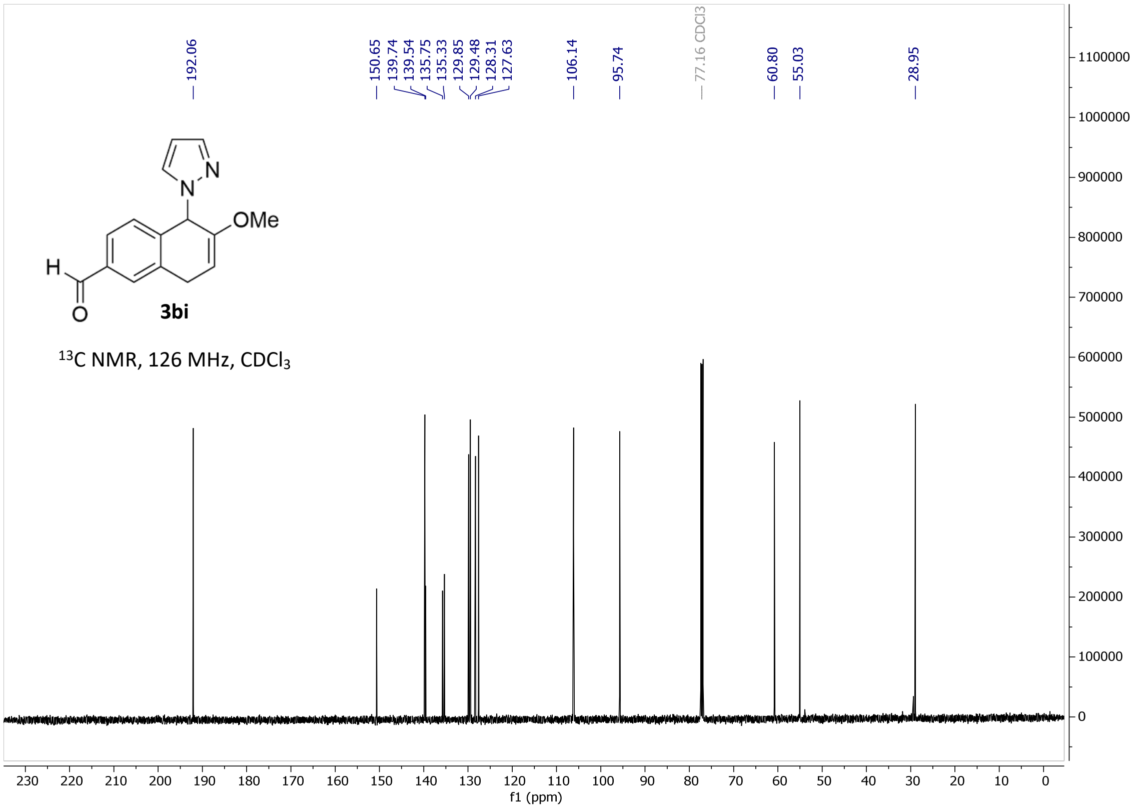

Supplementary Figure 151. ^{13}C NMR spectra of **3bi** (126 MHz, rt, $CDCl_3$).

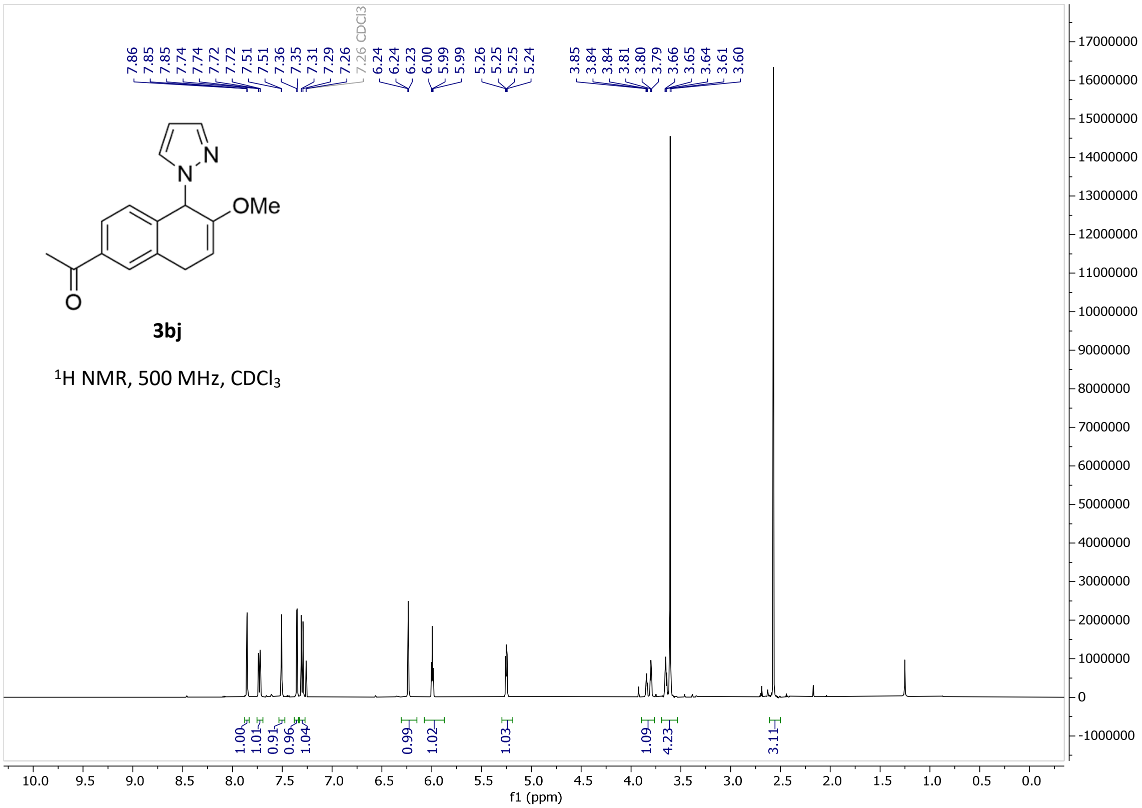

Supplementary Figure 152. ^{1}H NMR spectra of **3bj** (500 MHz, rt, $CDCl_3$).

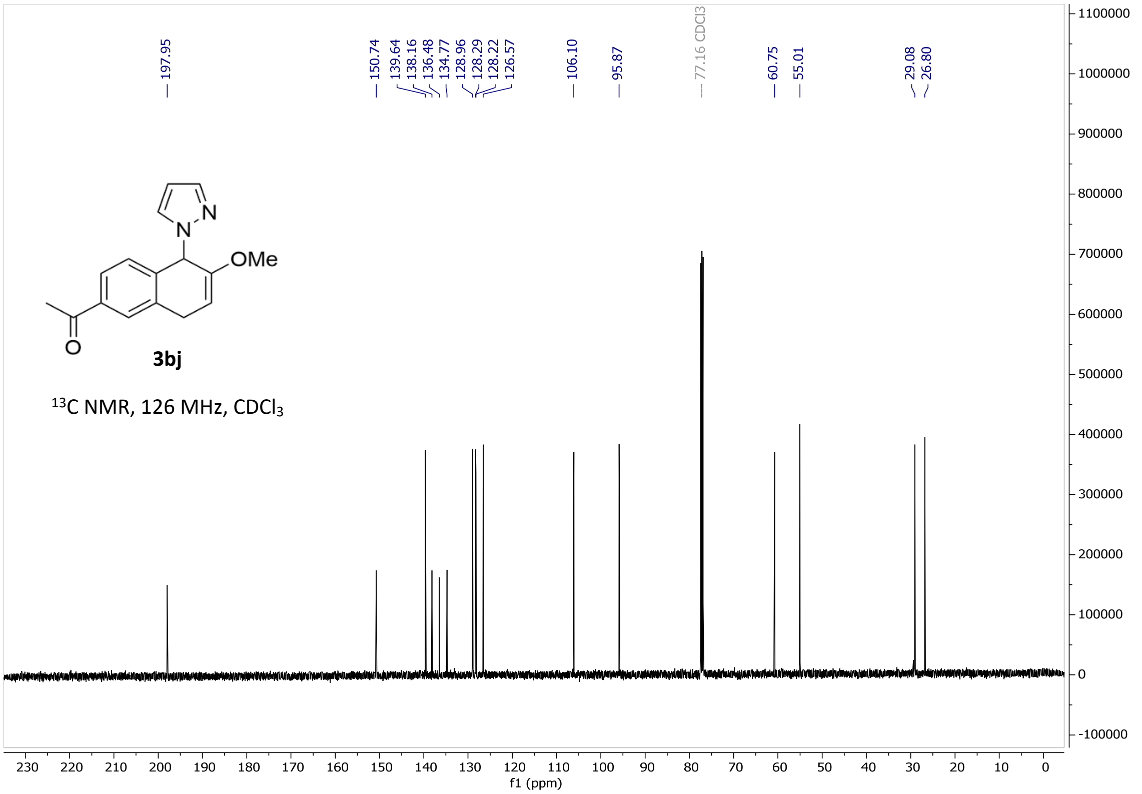

Supplementary Figure 153. ^{13}C NMR spectra of **3bj** (126 MHz, rt, $CDCl_3$).

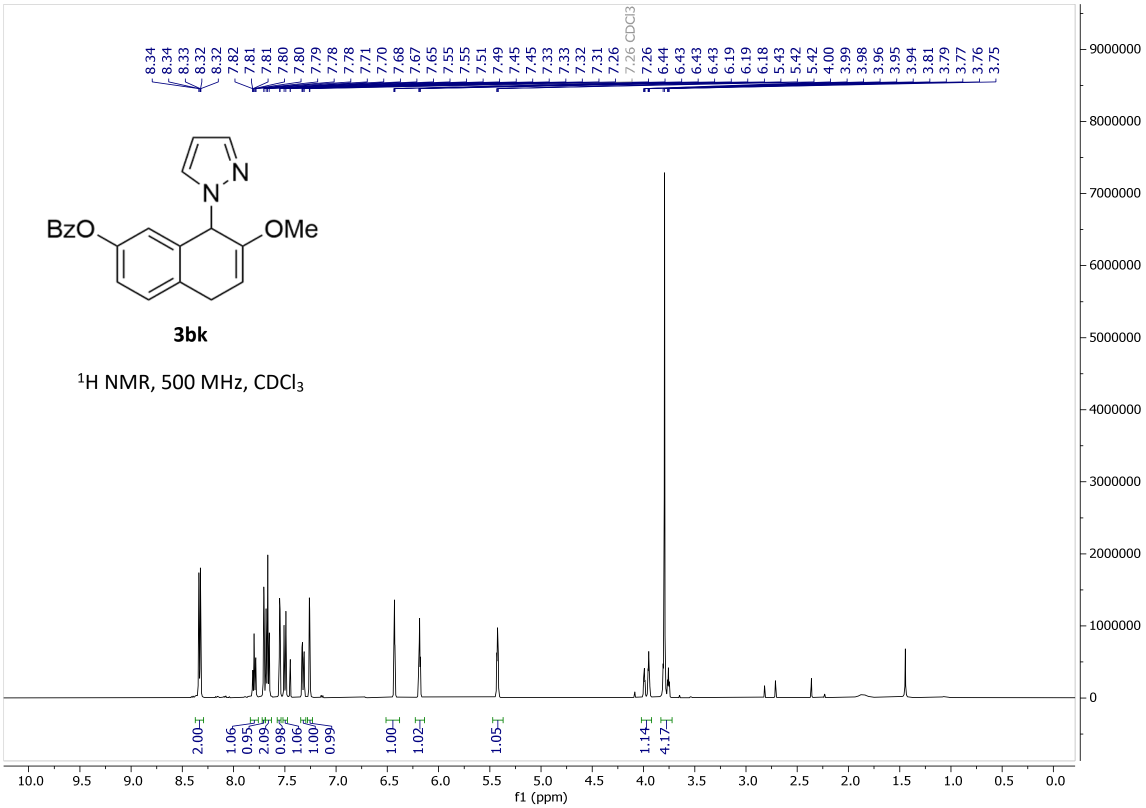

Supplementary Figure 154. ^{1}H NMR spectra of **3bk** (500 MHz, rt, $CDCl_3$).

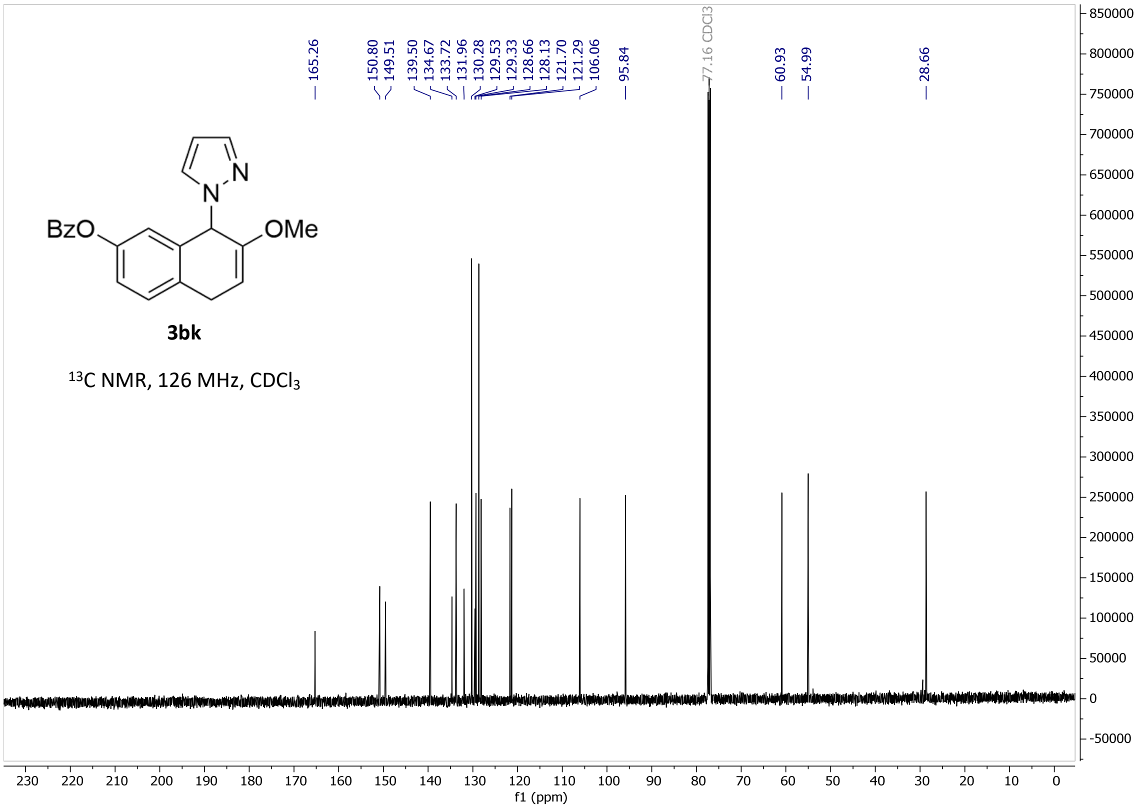

Supplementary Figure 155. ^{13}C NMR spectra of **3bk** (126 MHz, rt, $CDCl_3$).

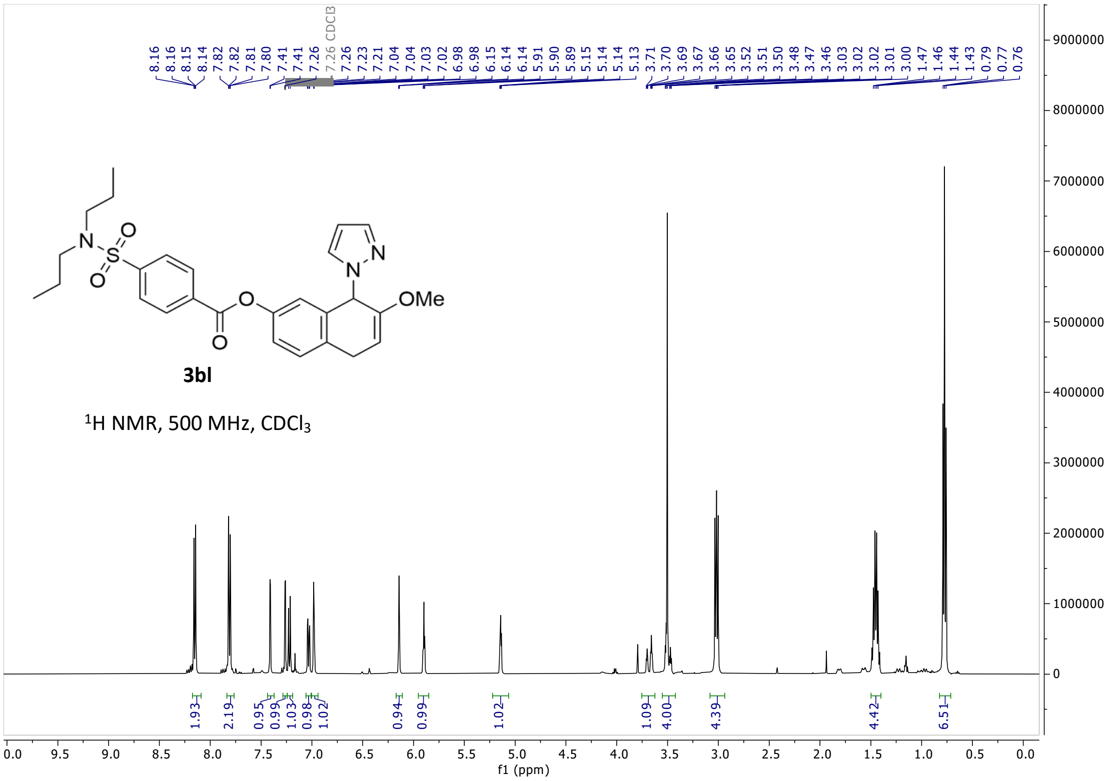

Supplementary Figure 156. ^{1}H NMR spectra of **3bl** (500 MHz, rt, $CDCl_3$).

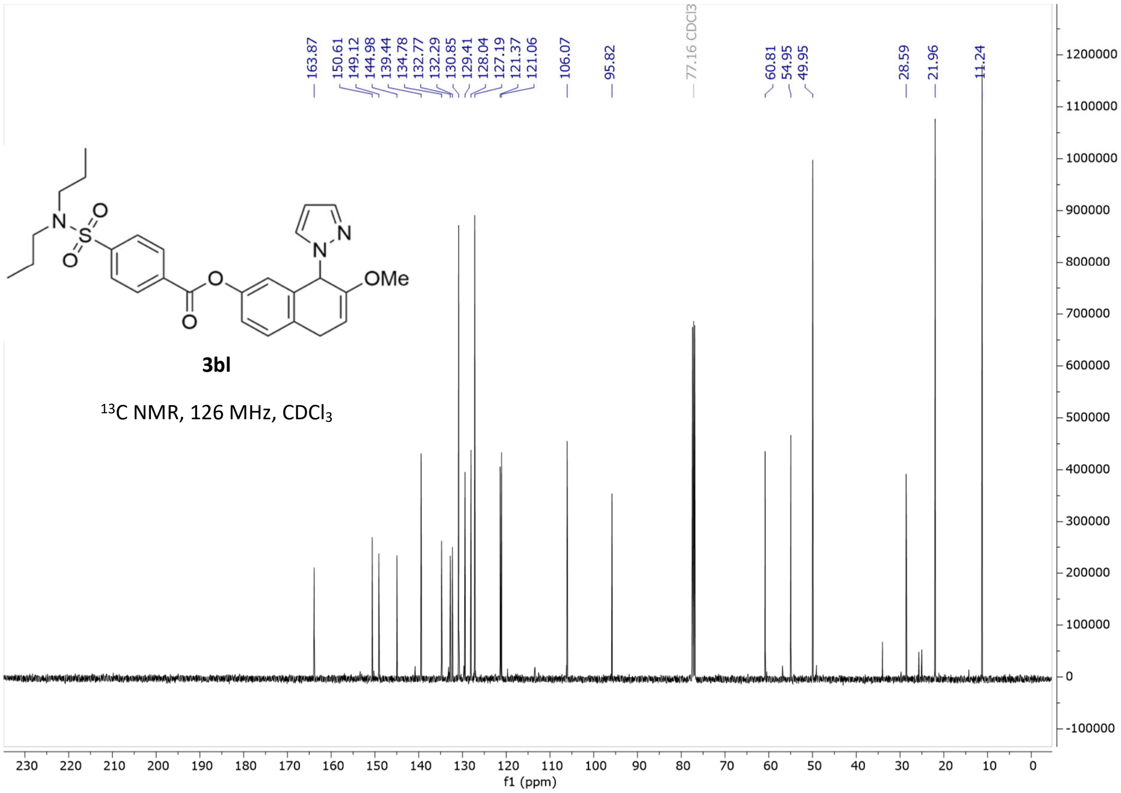

Supplementary Figure 157. ^{13}C NMR spectra of **3bl** (126 MHz, rt, $CDCl_3$).

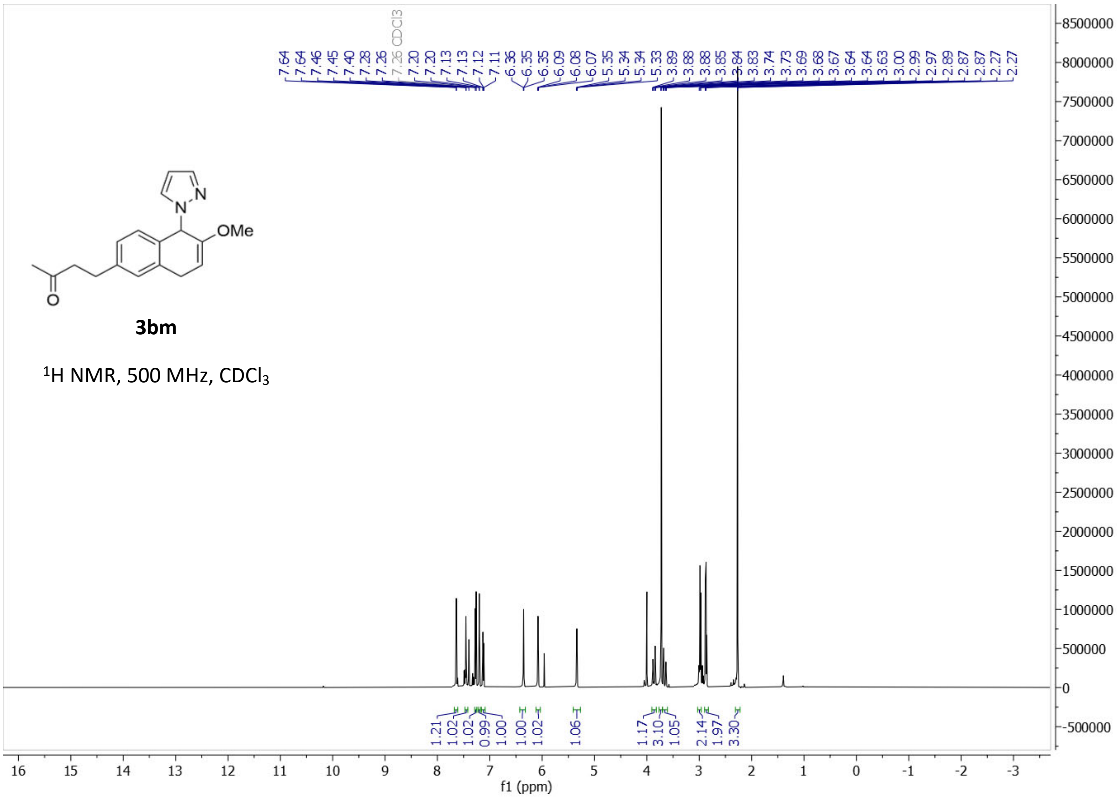

Supplementary Figure 158. ^{1}H NMR spectra of **3bm** (500 MHz, rt, $CDCl_3$).

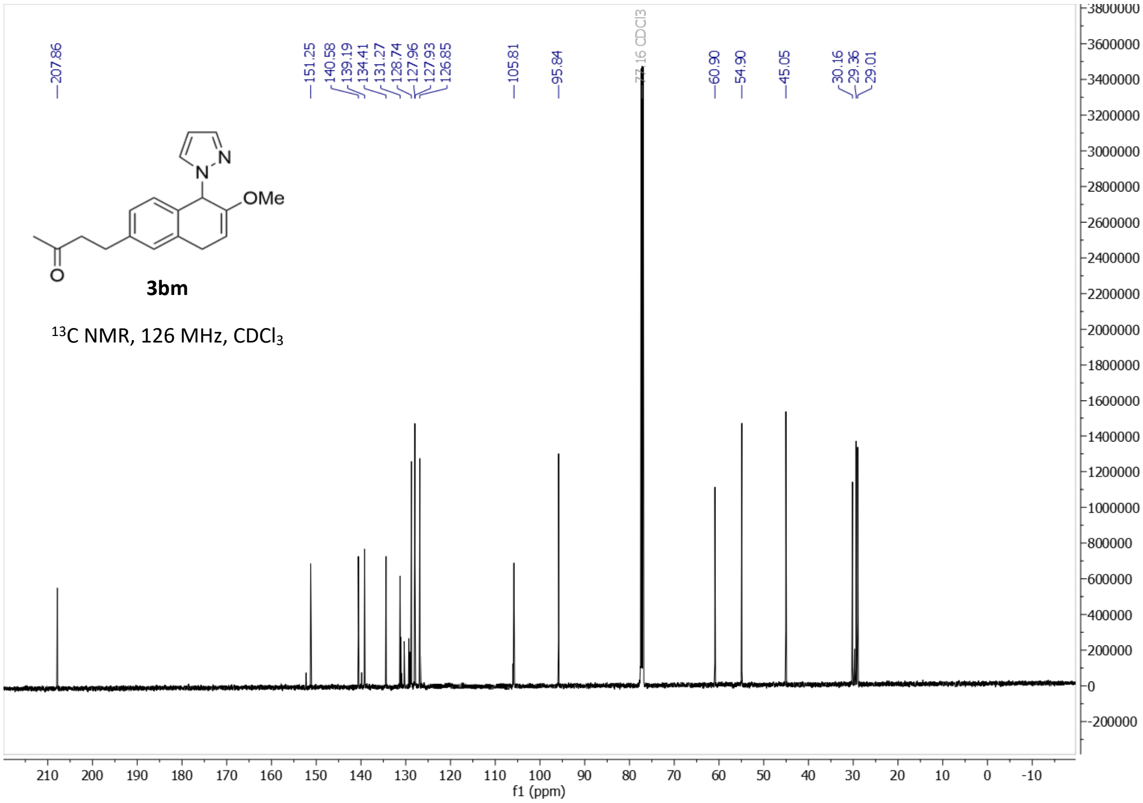

Supplementary Figure 159. ^{13}C NMR spectra of **3bm** (126 MHz, rt, $CDCl_3$).

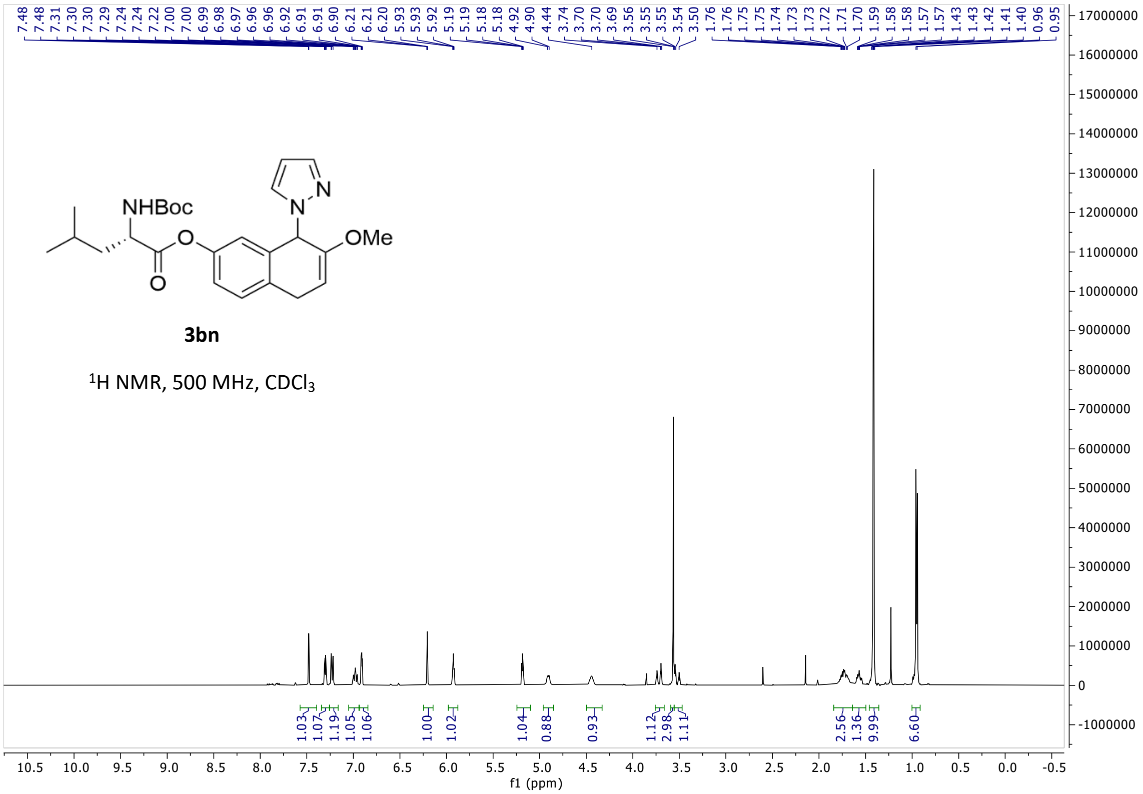

Supplementary Figure 160. ^{1}H NMR spectra of **3bn** (500 MHz, rt, $CDCl_3$).

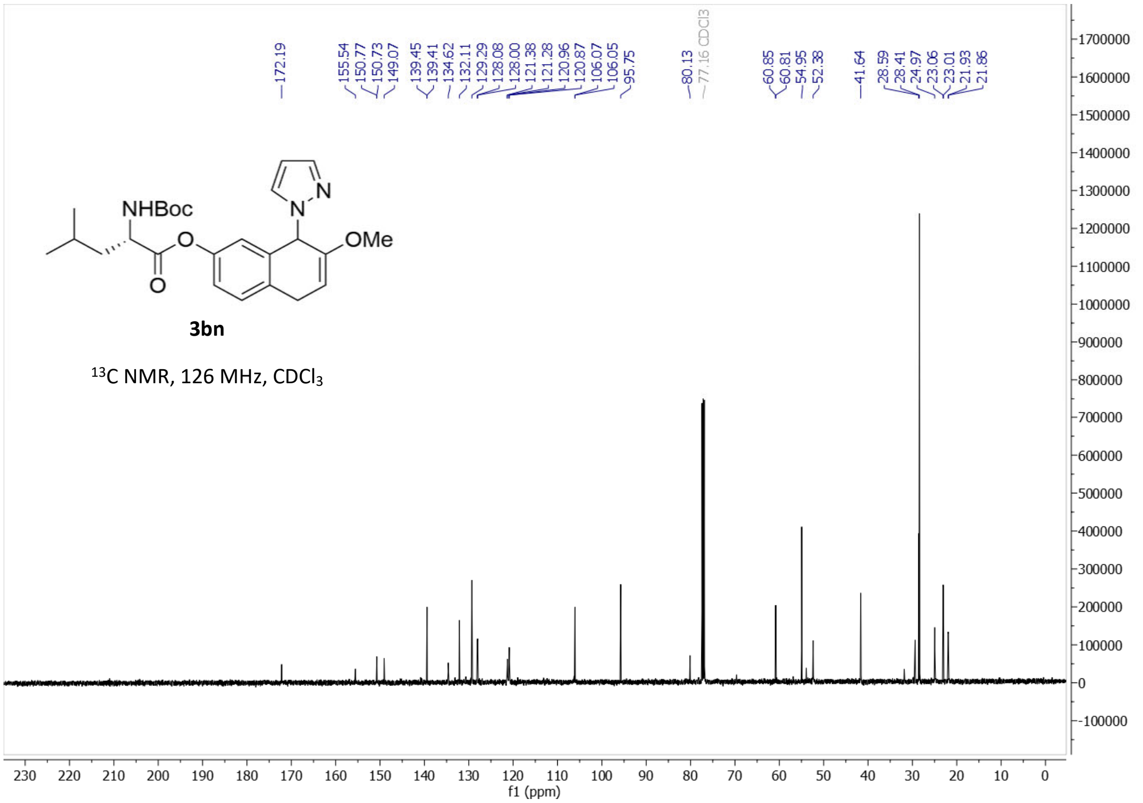

Supplementary Figure 161. ^{13}C NMR spectra of **3bn** (126 MHz, rt, $CDCl_3$).

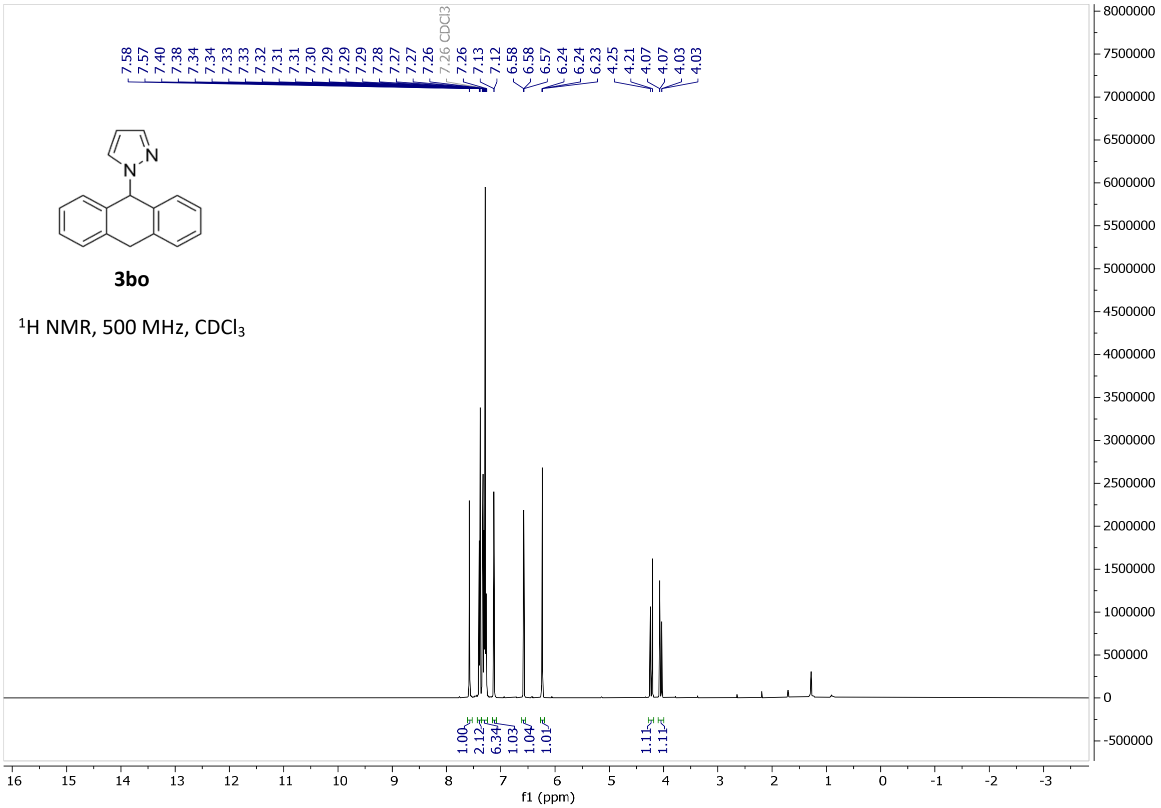

Supplementary Figure 162. ^{1}H NMR spectra of **3bo** (500 MHz, rt, $CDCl_3$).

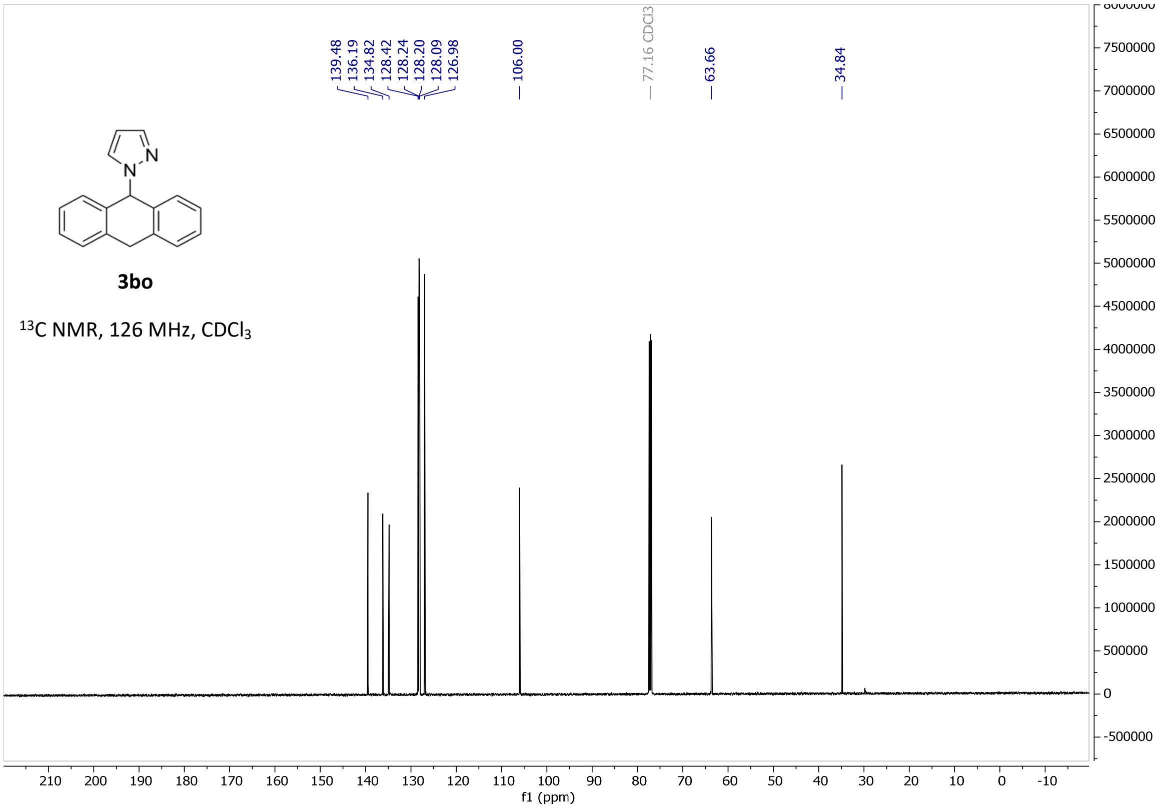

Supplementary Figure 162. ^{13}C NMR spectra of **3bo** (126 MHz, rt, $CDCl_3$).

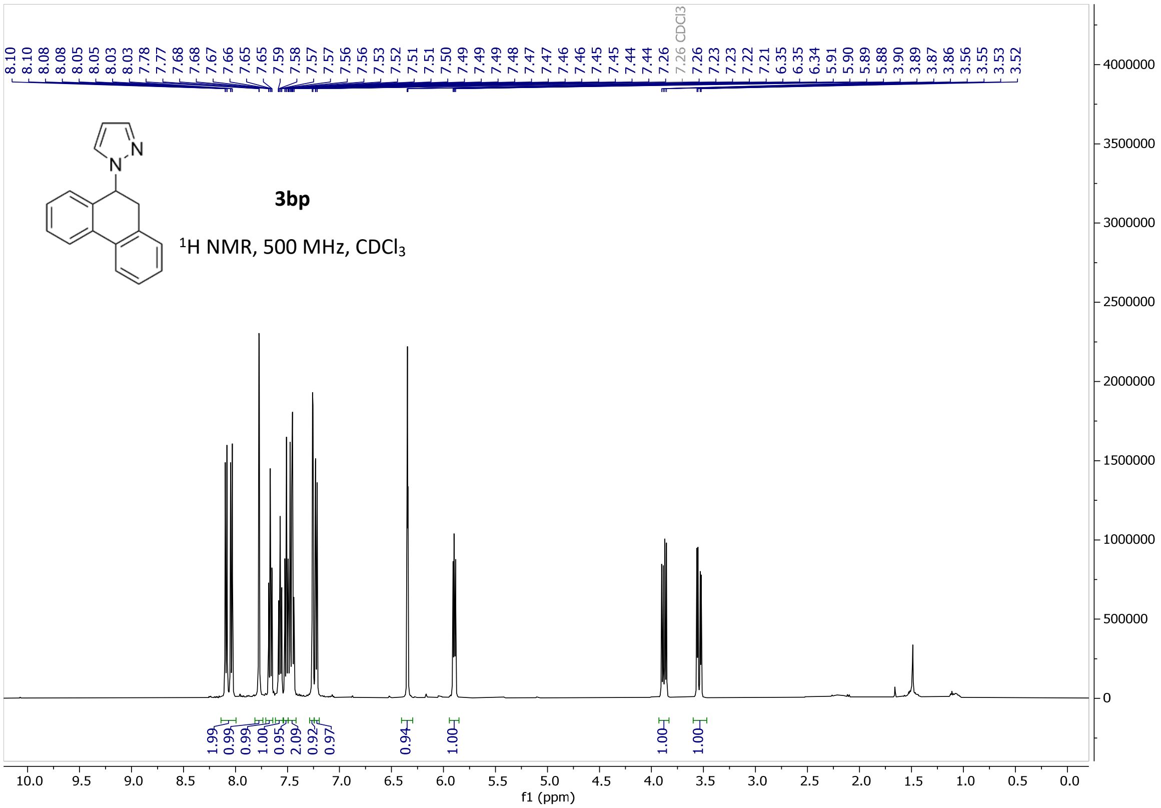

Supplementary Figure 164. ^{1}H NMR spectra of **3bp** (500 MHz, rt, $CDCl_3$).

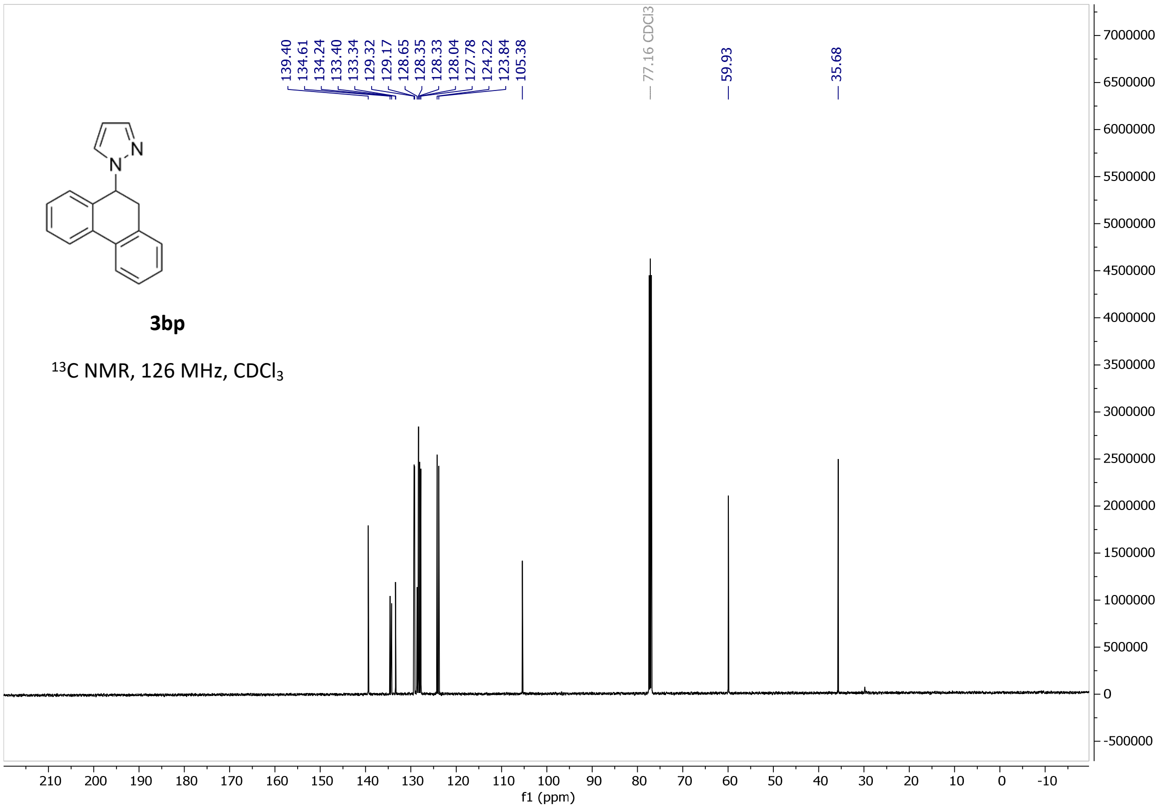

Supplementary Figure 165. ^{13}C NMR spectra of **3bp** (126 MHz, rt, $CDCl_3$).

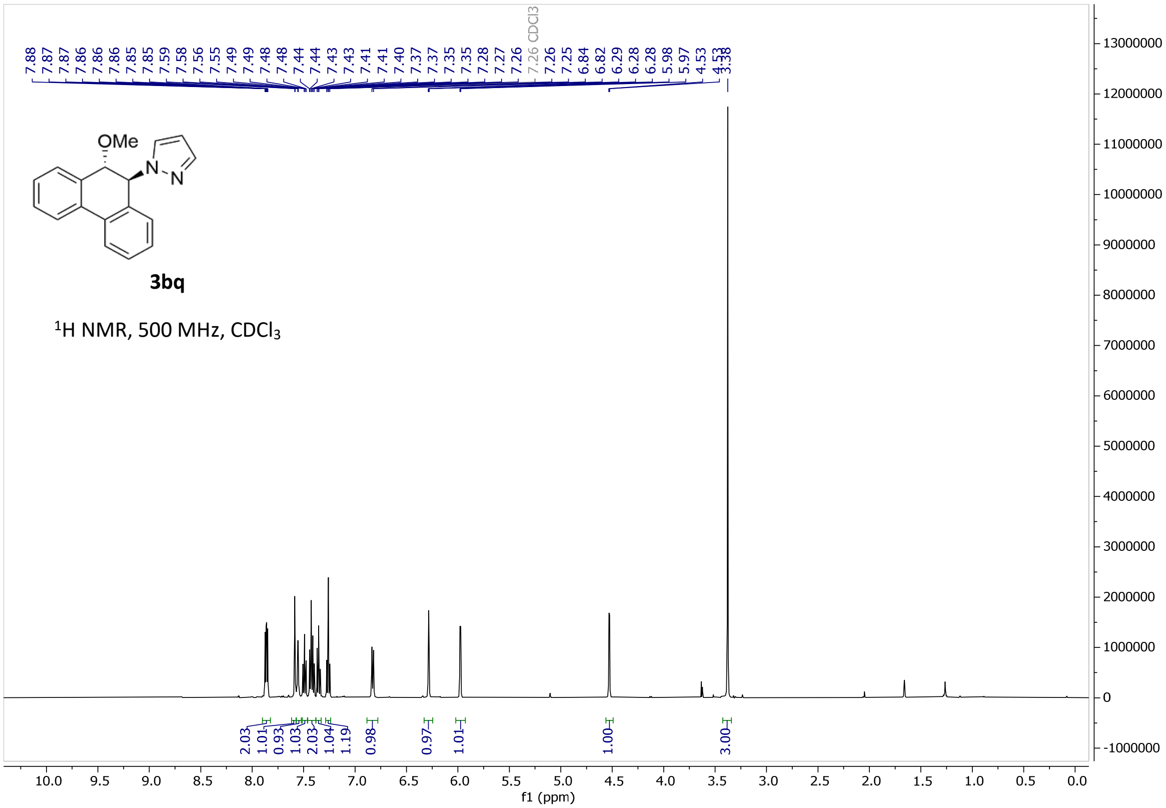

Supplementary Figure 166. ^{1}H NMR spectra of **3bq** (500 MHz, rt, $CDCl_3$).

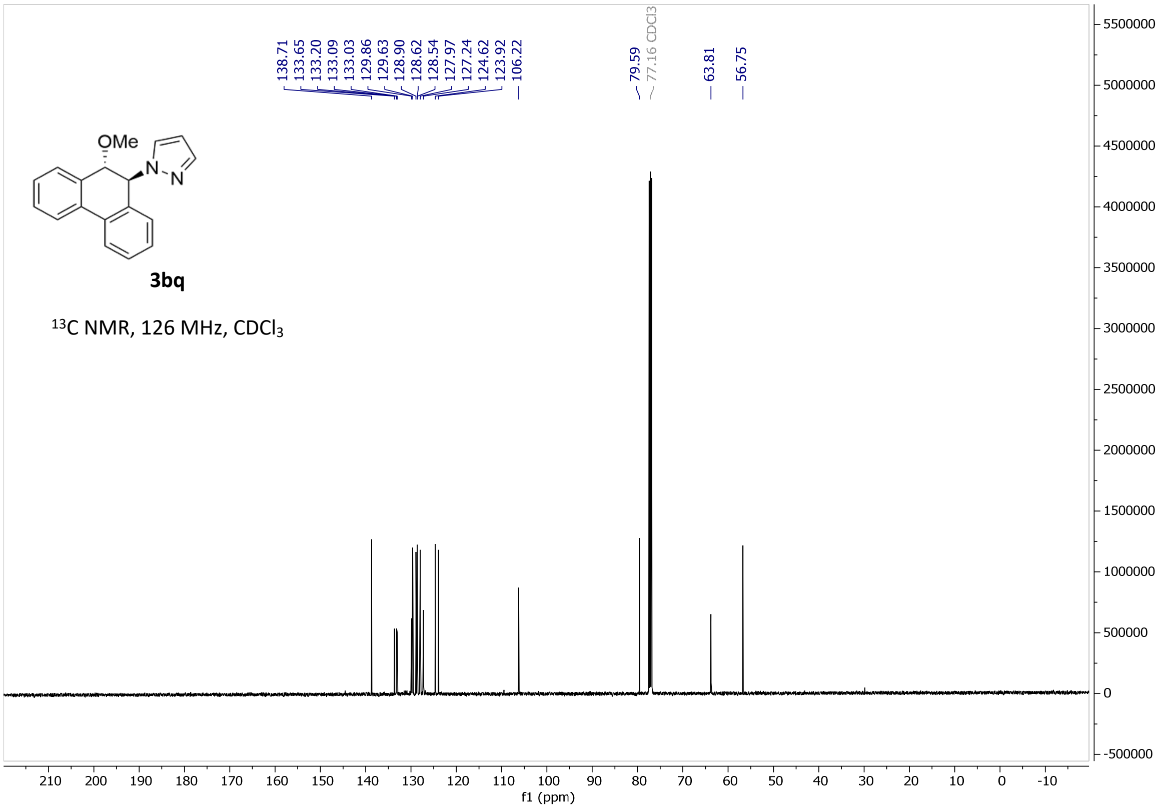

Supplementary Figure 167. ^{13}C NMR spectra of **3bq** (126 MHz, rt, $CDCl_3$).

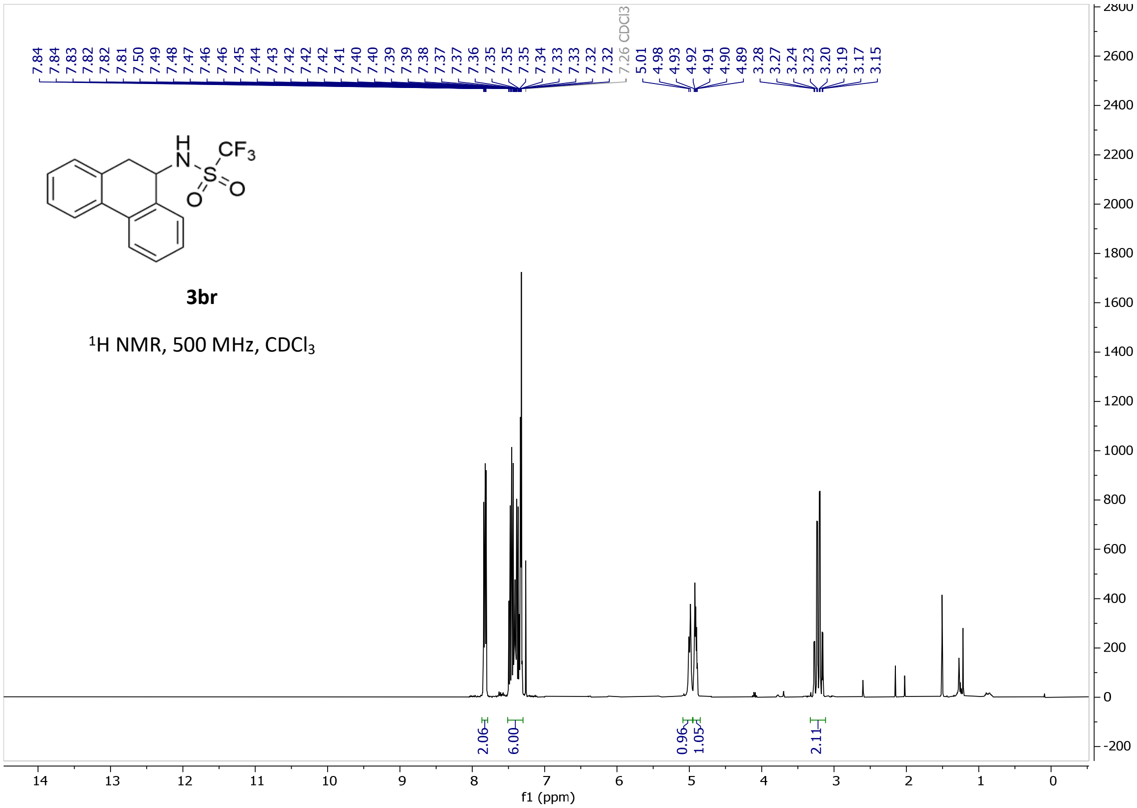

Supplementary Figure 168. ^{1}H NMR spectra of **3br** (500 MHz, rt, $CDCl_3$).

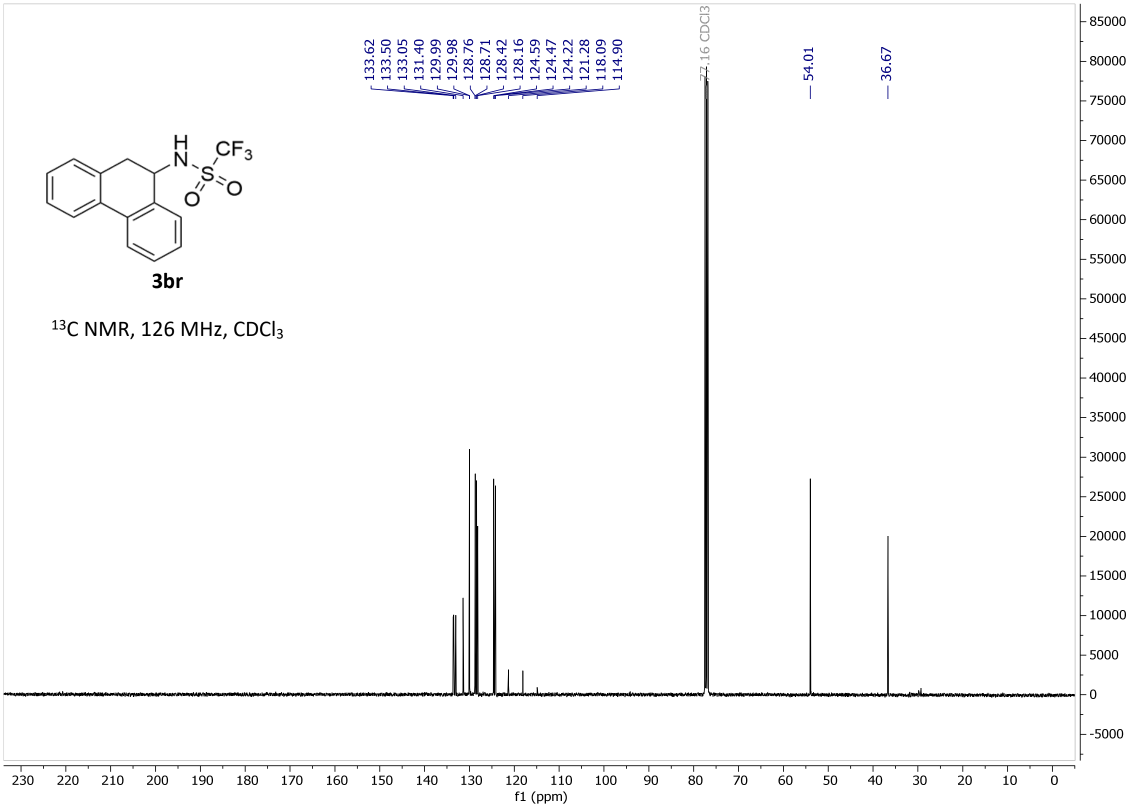

Supplementary Figure 169. ^{13}C NMR spectra of **3br** (126 MHz, rt, $CDCl_3$).

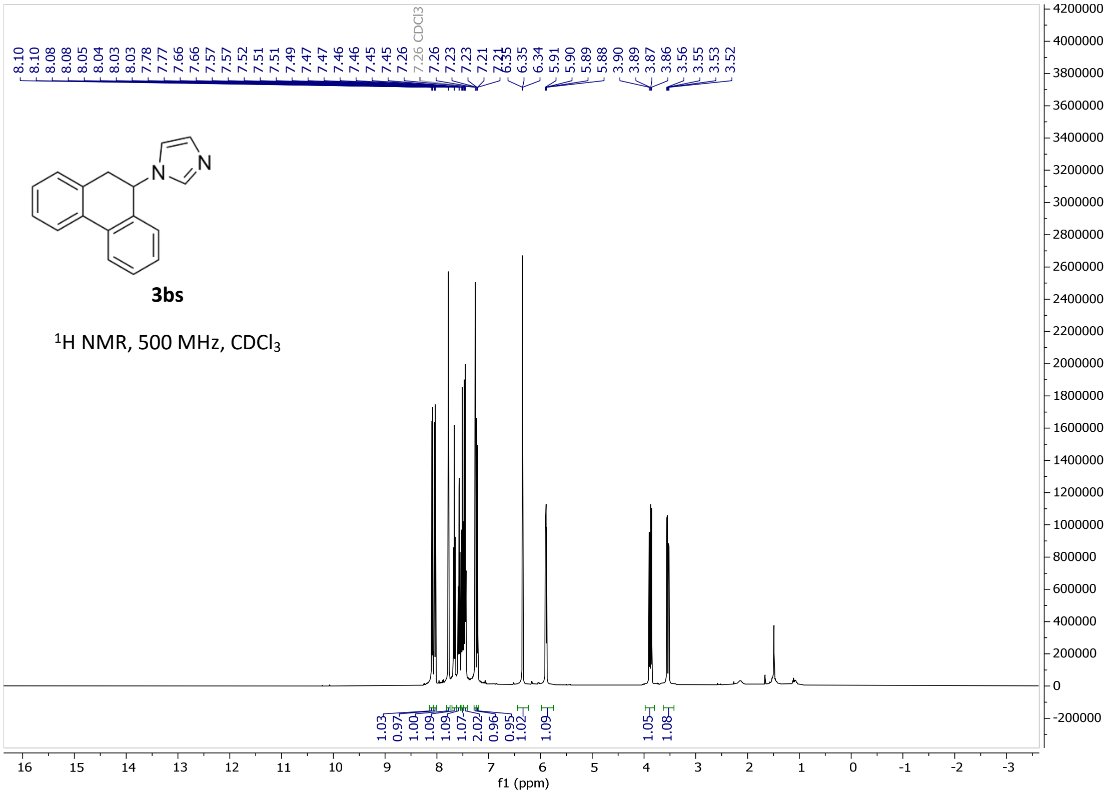

Supplementary Figure 170. ^{1}H NMR spectra of **3bs** (500 MHz, rt, $CDCl_3$).

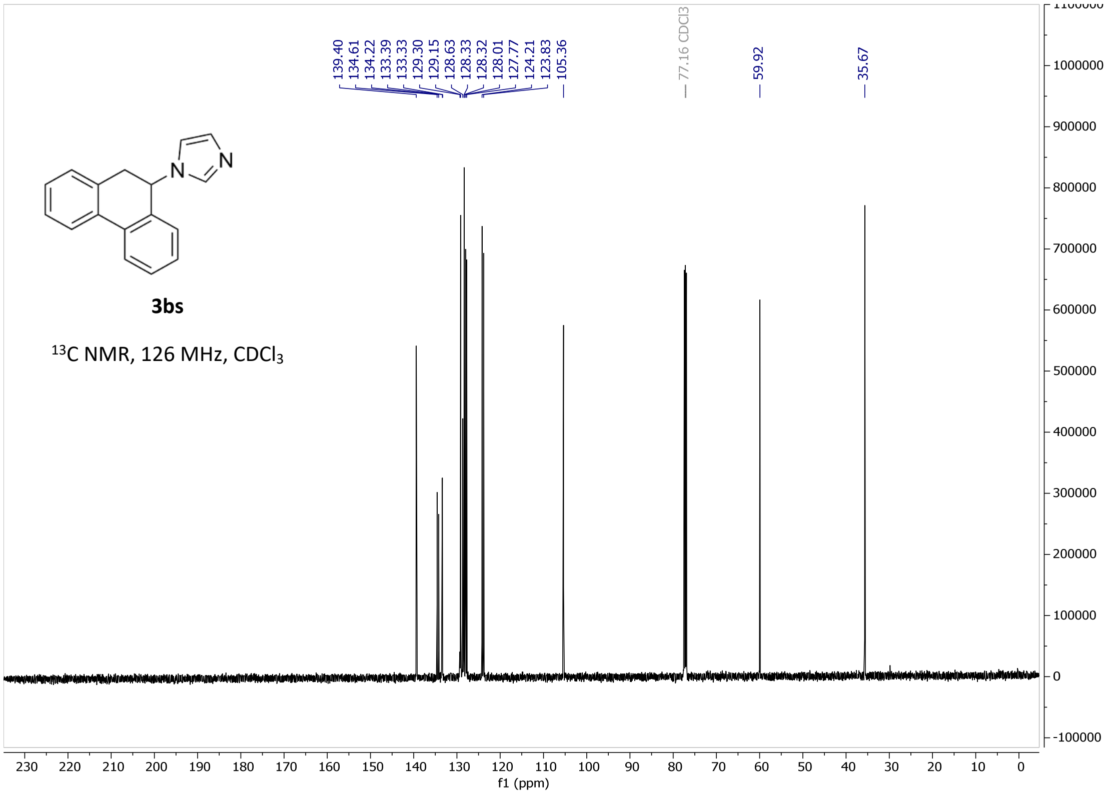

Supplementary Figure 171. ^{13}C NMR spectra of **3bs** (126 MHz, rt, $CDCl_3$).

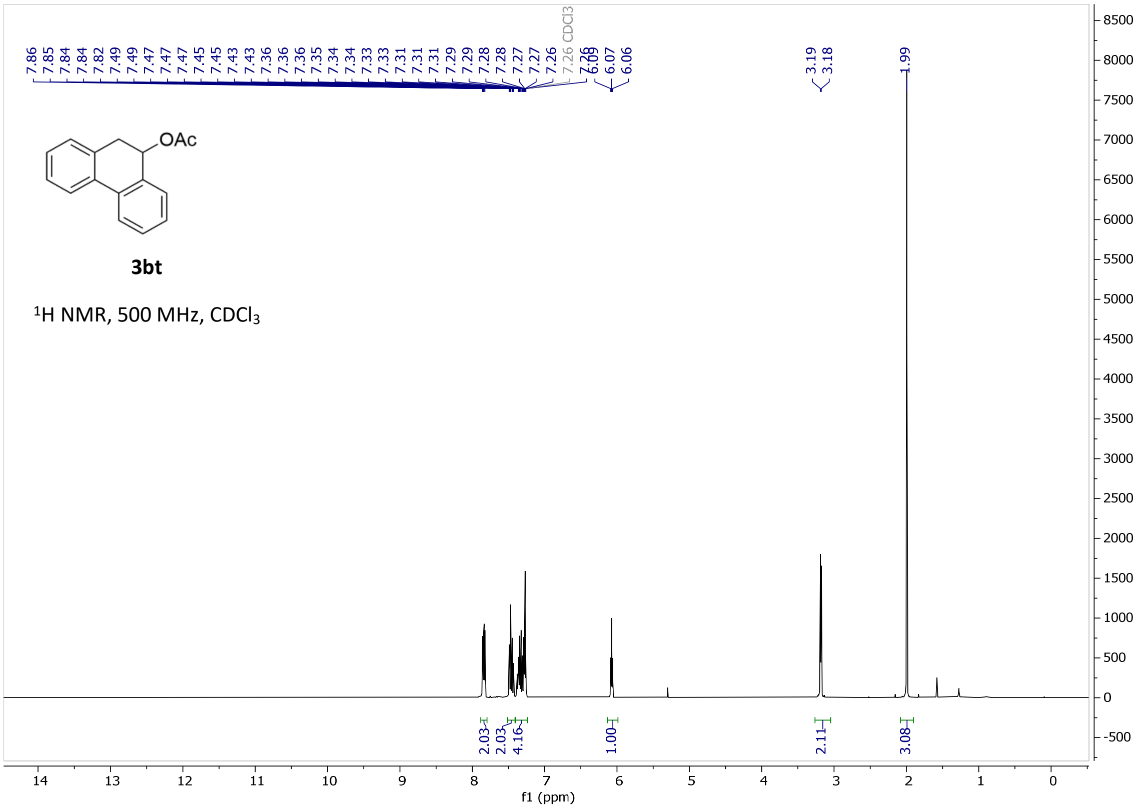

Supplementary Figure 172. ^{1}H NMR spectra of **3bt** (500 MHz, rt, $CDCl_3$).

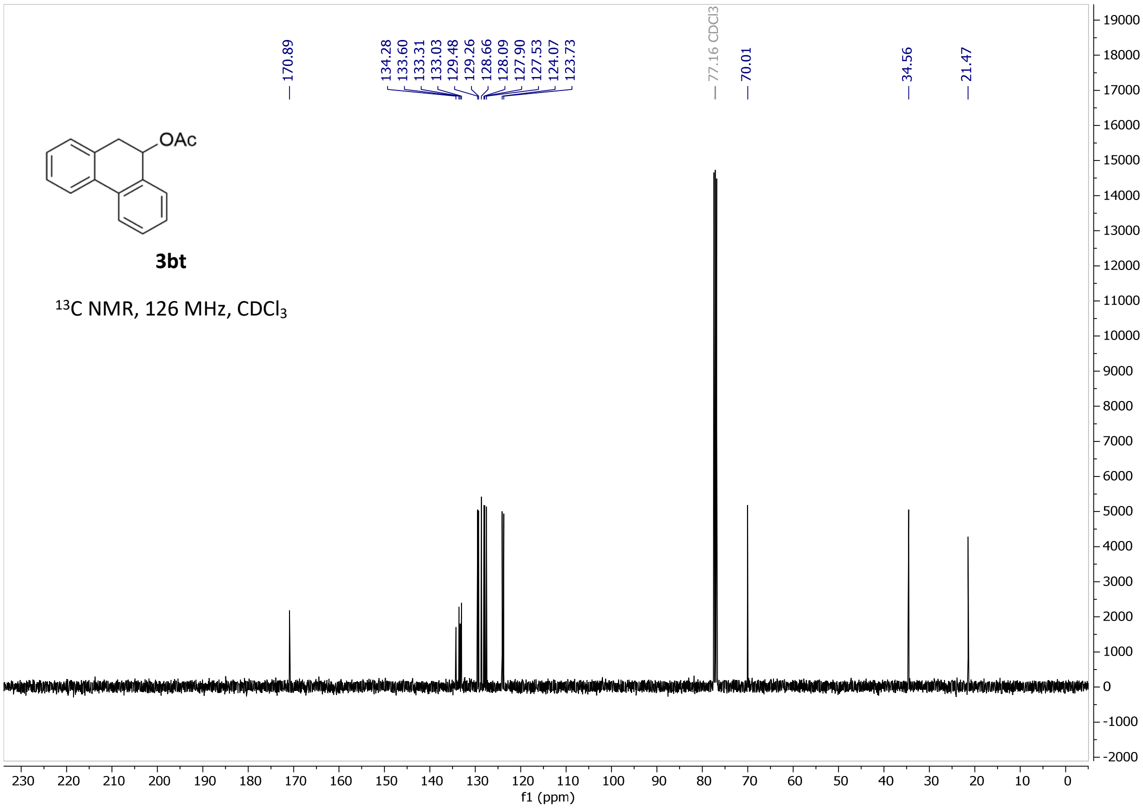

Supplementary Figure 173. ^{13}C NMR spectra of **3bt** (126 MHz, rt, $CDCl_3$).

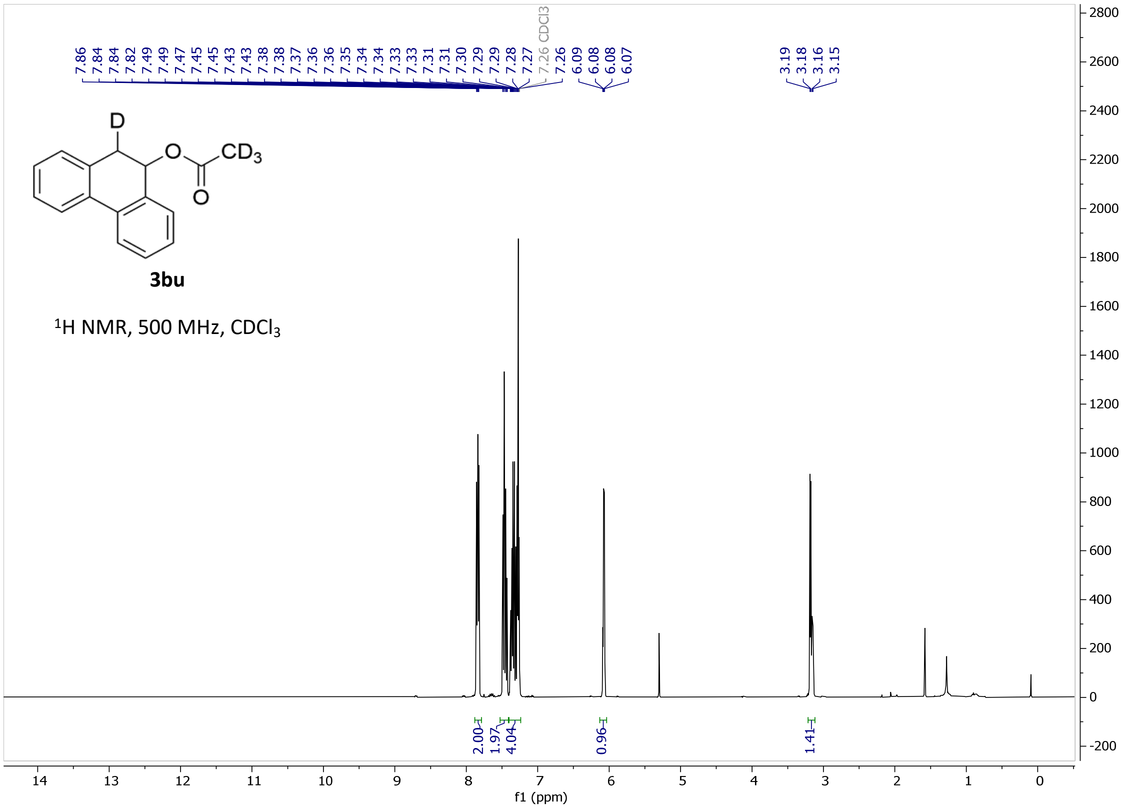

Supplementary Figure 174. ^{1}H NMR spectra of **3bu** (500 MHz, rt, $CDCl_3$).

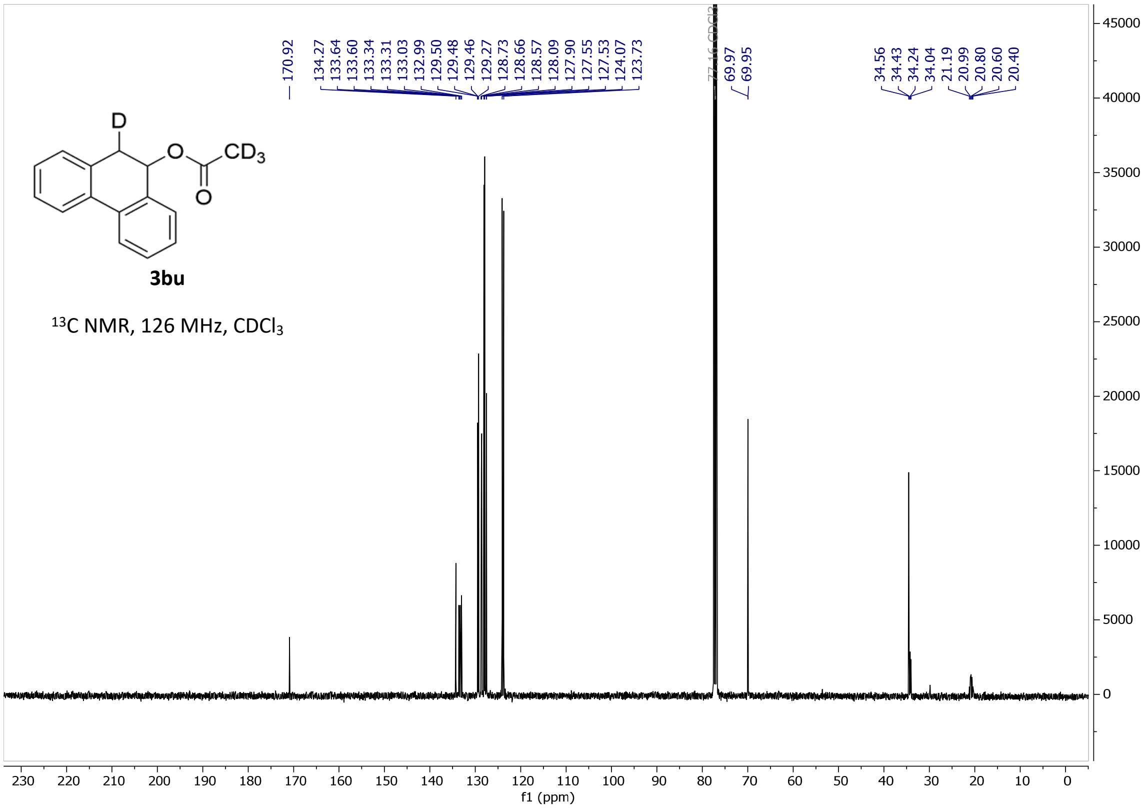

Supplementary Figure 175. ^{13}C NMR spectra of **3bu** (126 MHz, rt, $CDCl_3$).

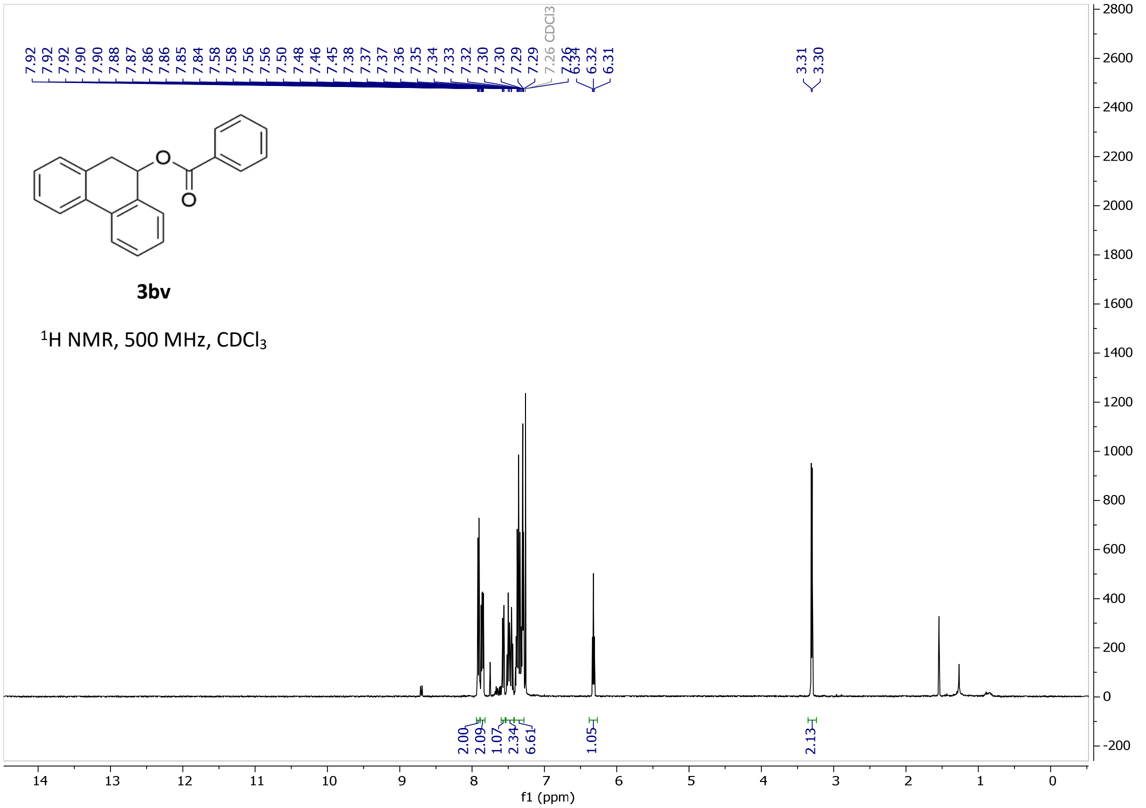

Supplementary Figure 176. ^{1}H NMR spectra of **3bv** (500 MHz, rt, CDCl$_3$).

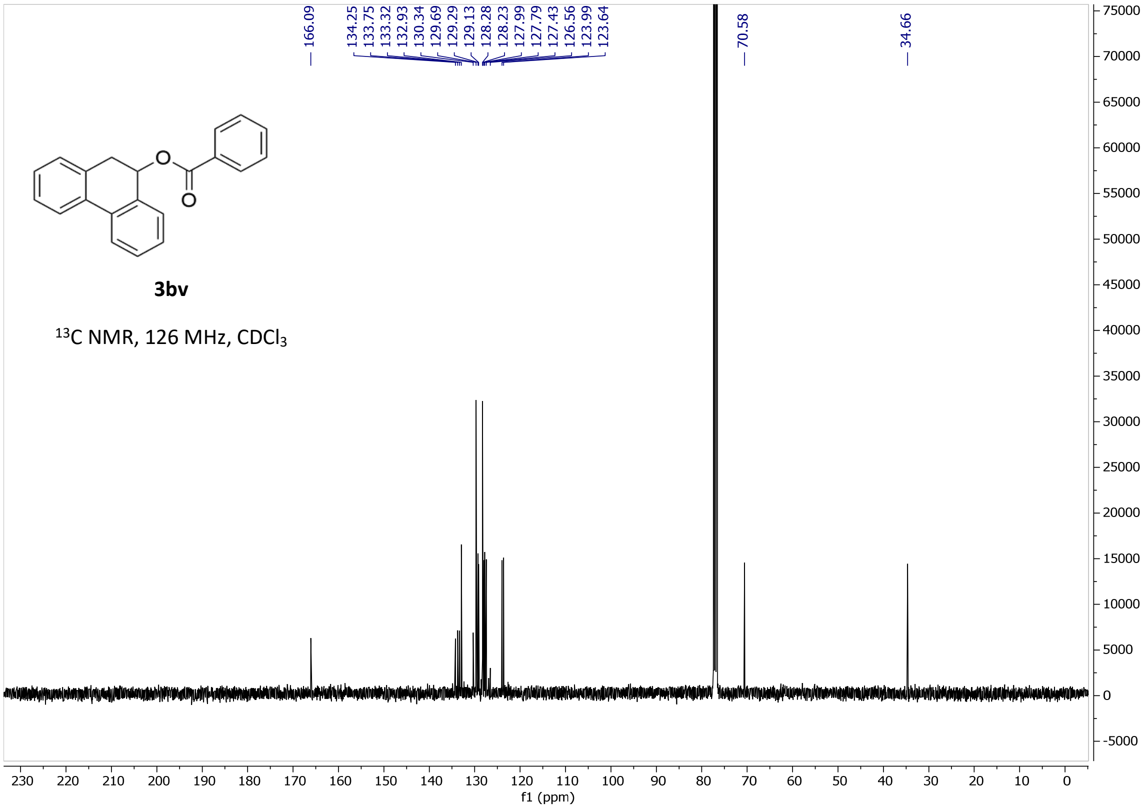

Supplementary Figure 177. ^{13}C NMR spectra of **3bv** (126 MHz, rt, CDCl$_3$).

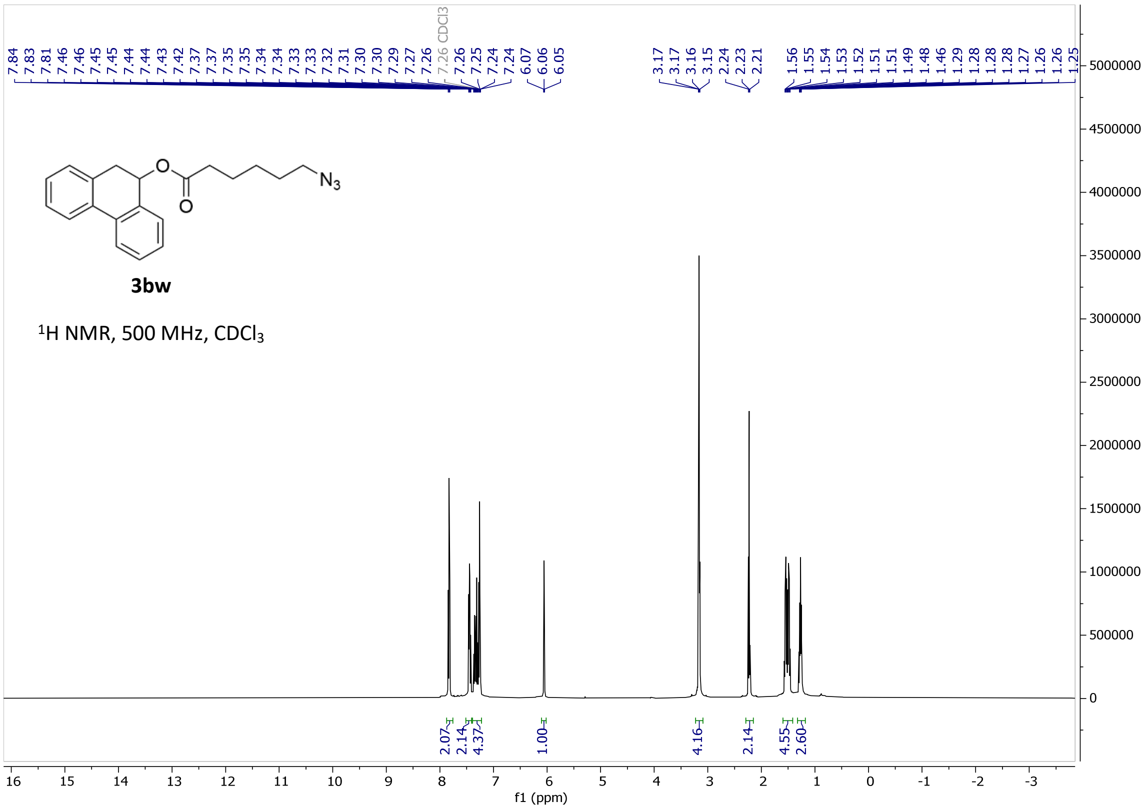

Supplementary Figure 178. ^{1}H NMR spectra of **3bw** (500 MHz, rt, $CDCl_3$).

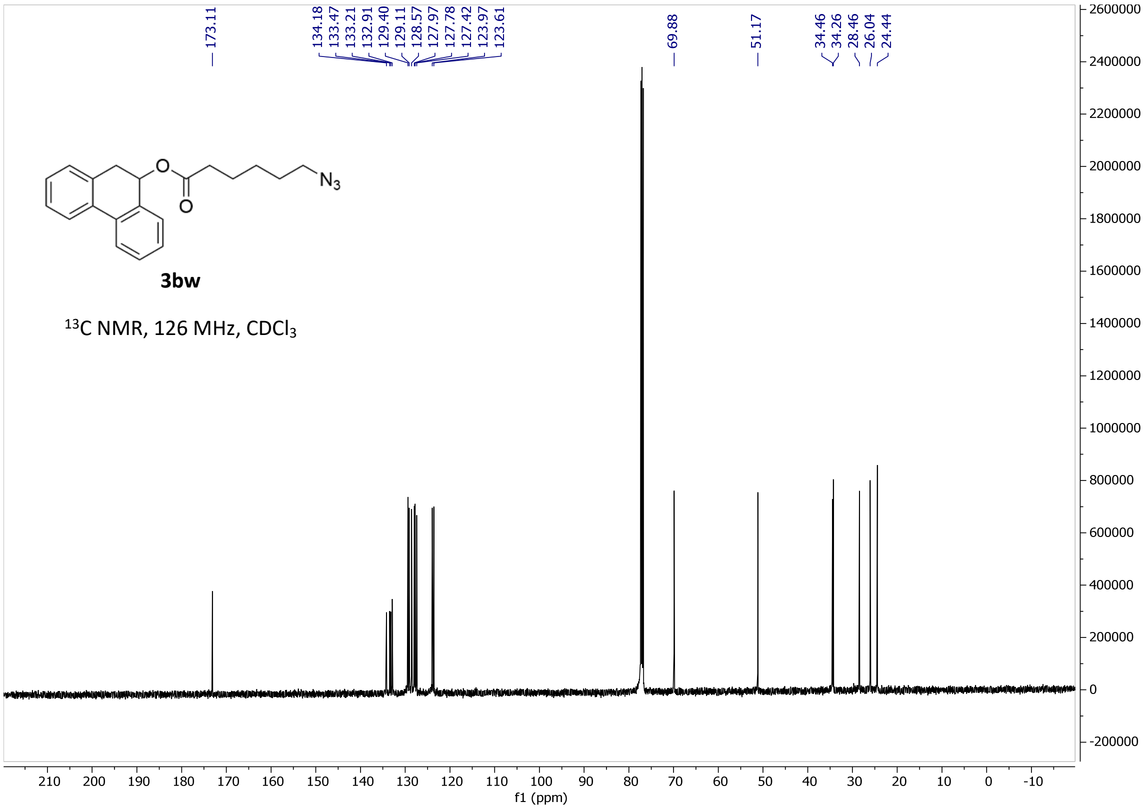

Supplementary Figure 179. ^{13}C NMR spectra of **3bw** (126 MHz, rt, $CDCl_3$).

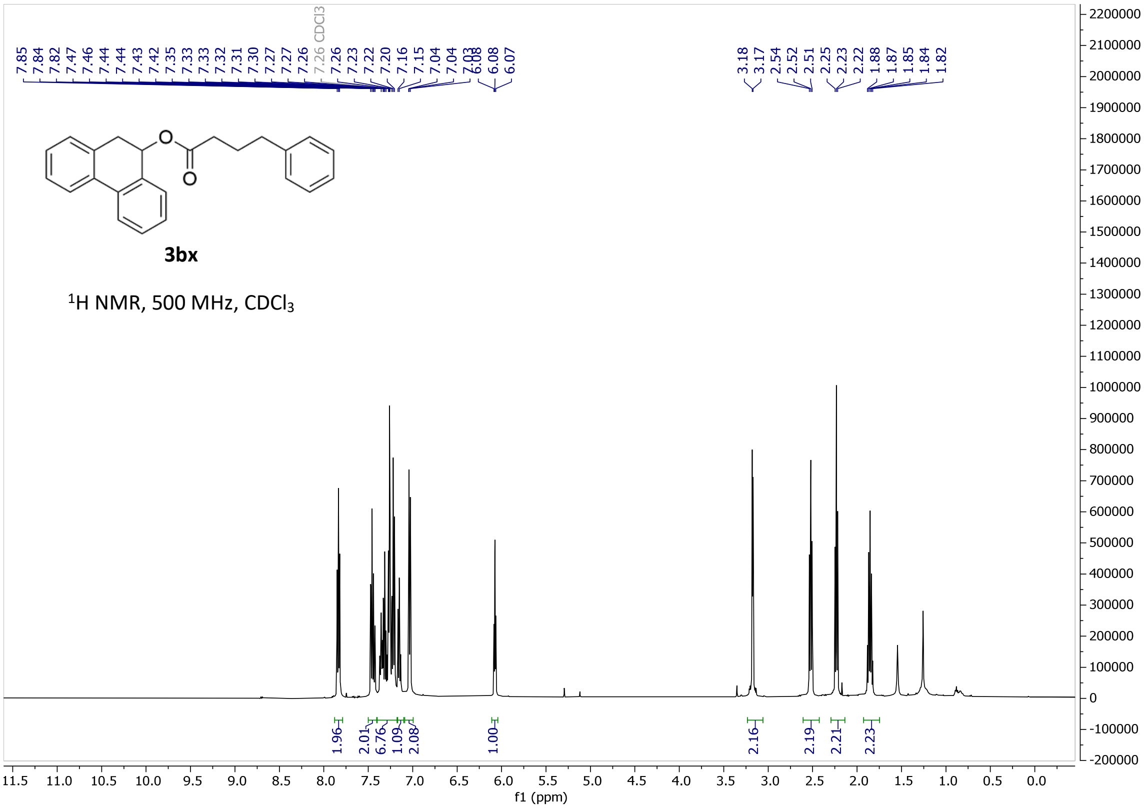

Supplementary Figure 180. ^{1}H NMR spectra of **3bx** (500 MHz, rt, $CDCl_3$).

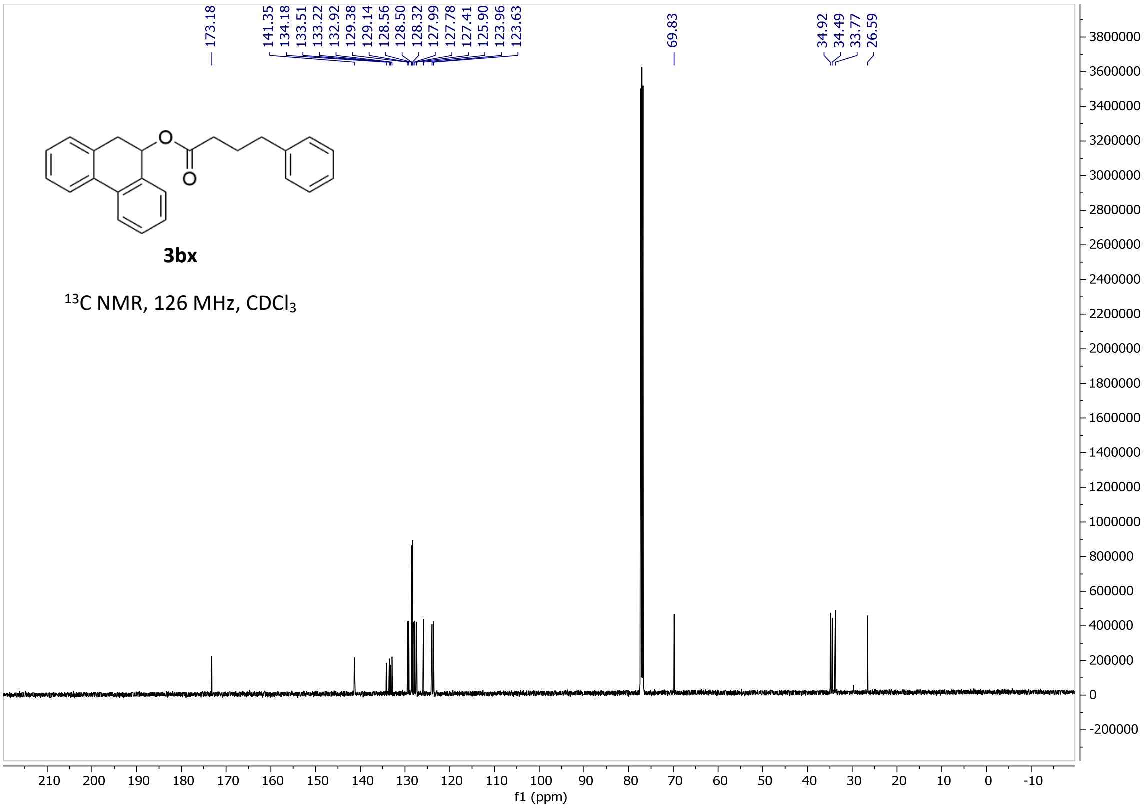

Supplementary Figure 181. ^{13}C NMR spectra of **3bx** (126 MHz, rt, $CDCl_3$).

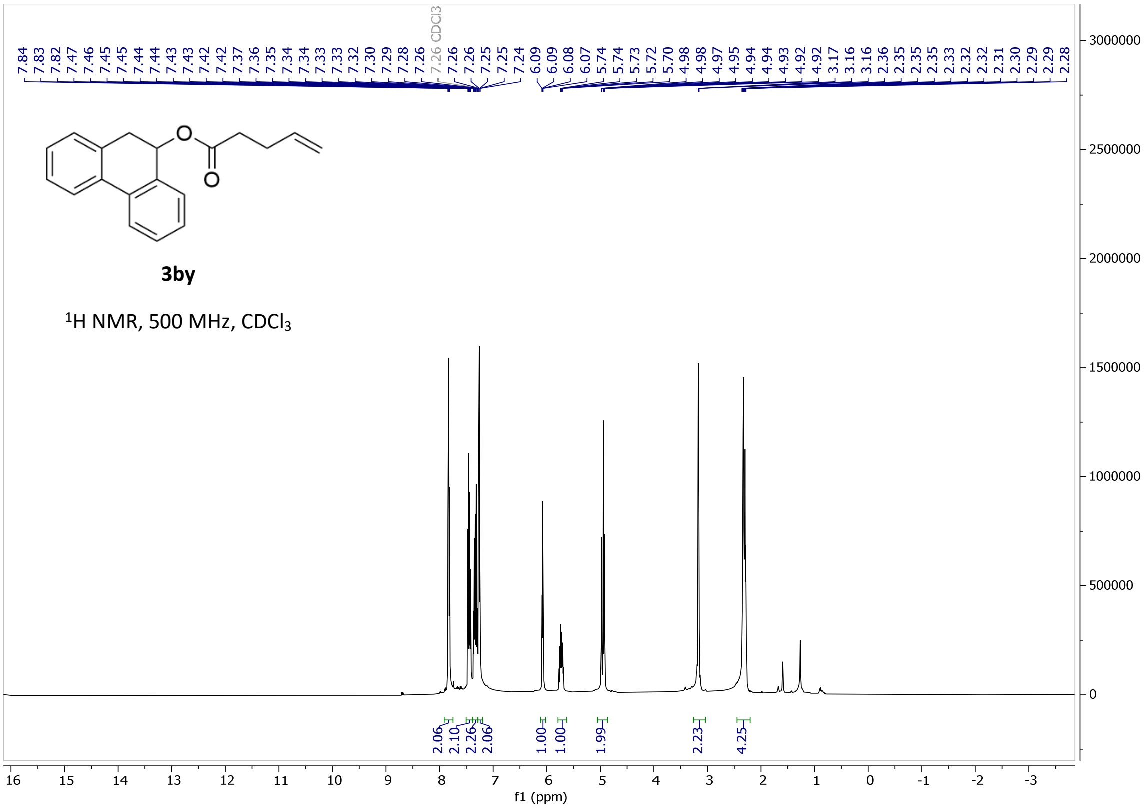

Supplementary Figure 182. ^{1}H NMR spectra of **3by** (500 MHz, rt, $CDCl_3$).

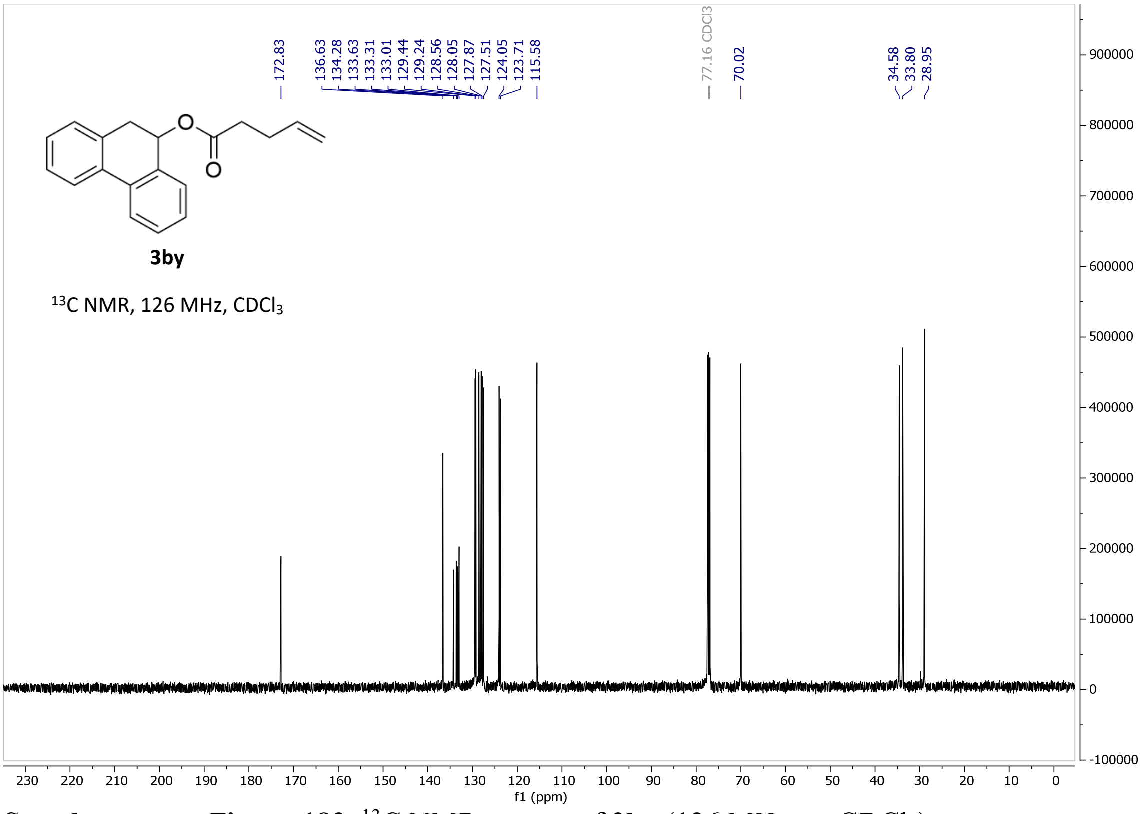

Supplementary Figure 183. ^{13}C NMR spectra of **3by** (126 MHz, rt, $CDCl_3$).

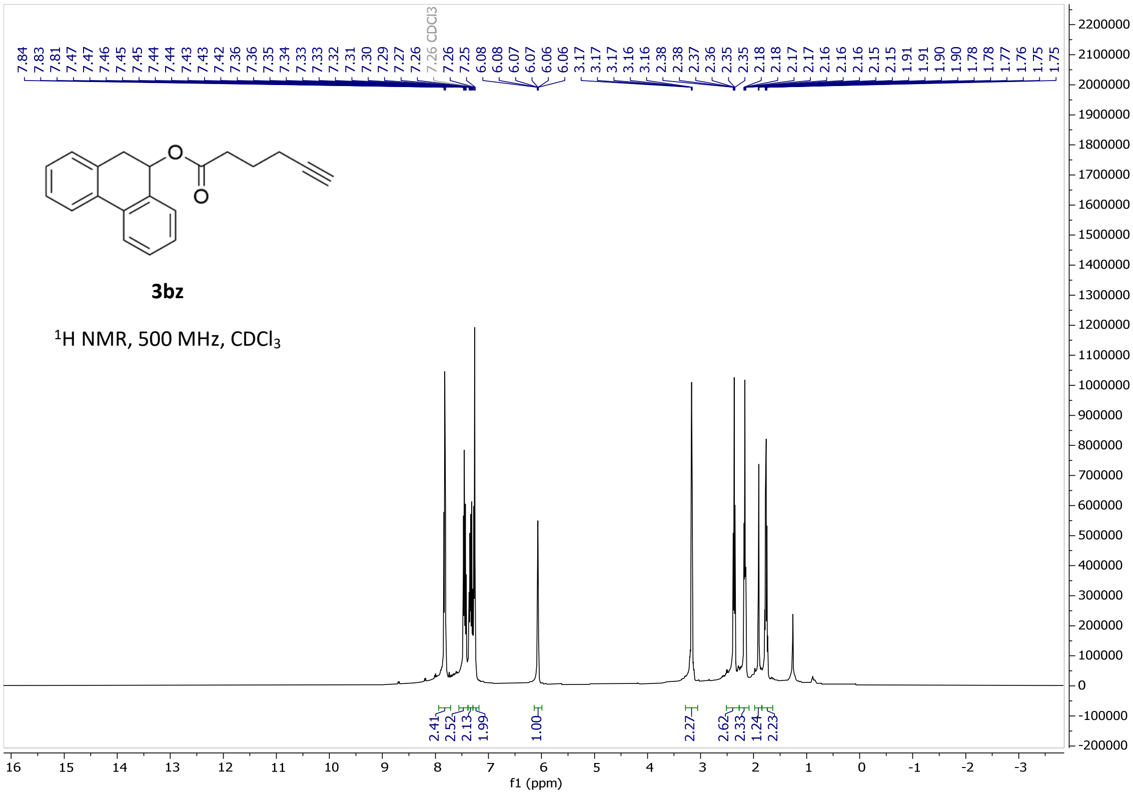

Supplementary Figure 184. ^{1}H NMR spectra of **3bz** (500 MHz, rt, $CDCl_3$).

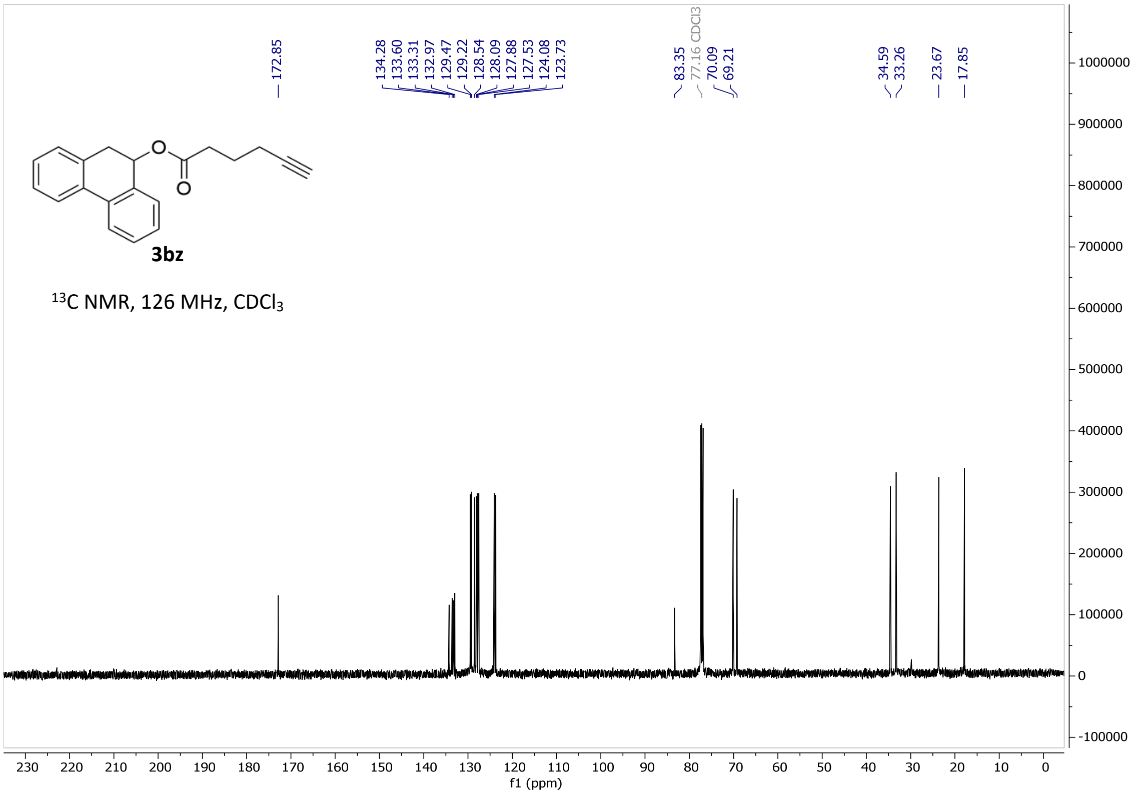

Supplementary Figure 185. ^{13}C NMR spectra of **3bz** (126 MHz, rt, $CDCl_3$).

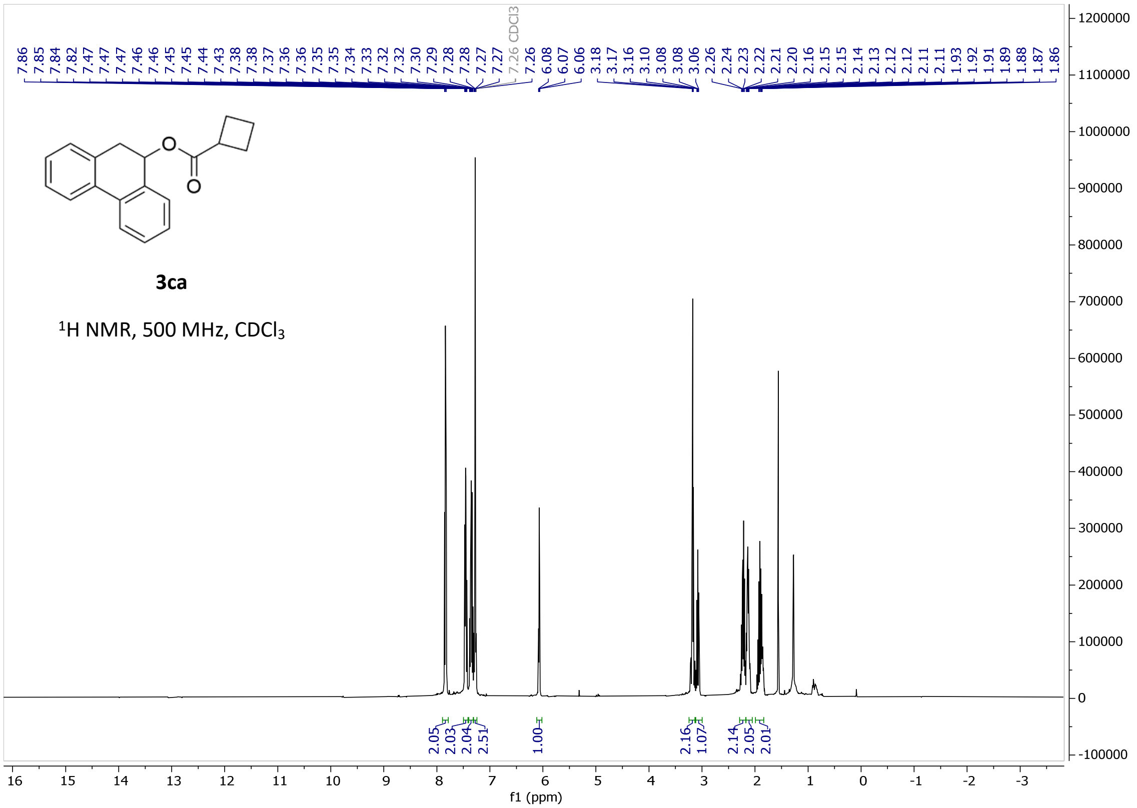

Supplementary Figure 186. ^{1}H NMR spectra of **3ca** (500 MHz, rt, $CDCl_3$).

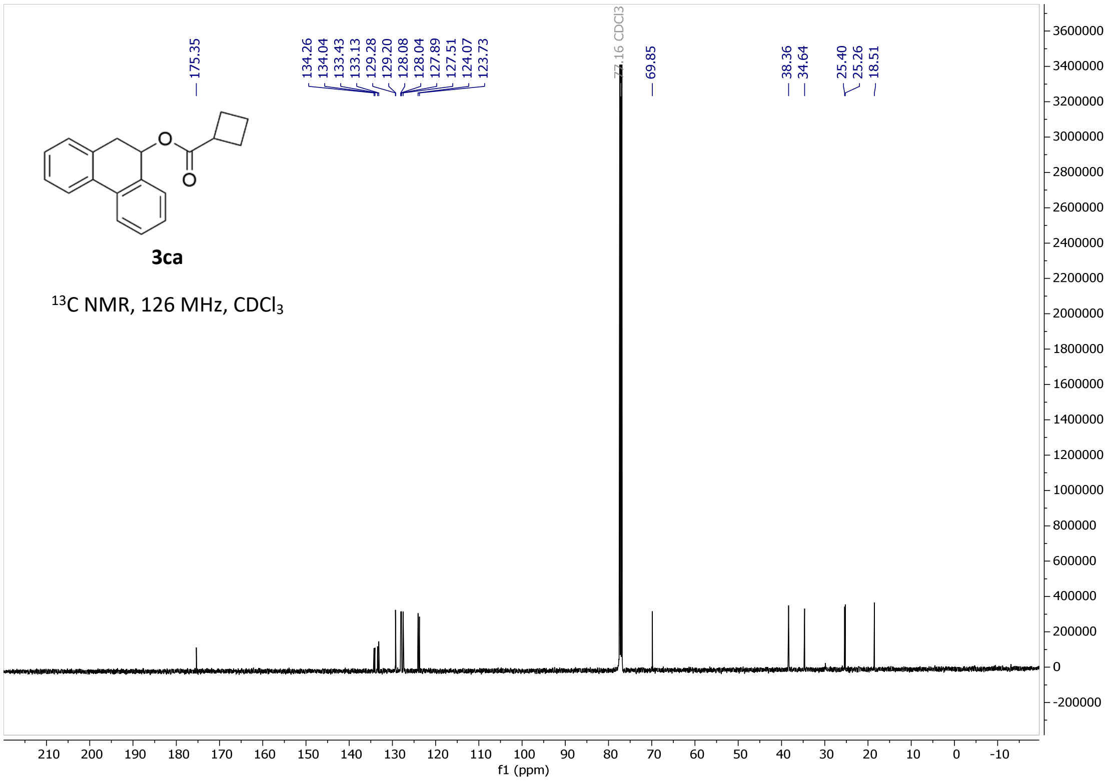

Supplementary Figure 187. ^{13}C NMR spectra of **3ca** (126 MHz, rt, $CDCl_3$).

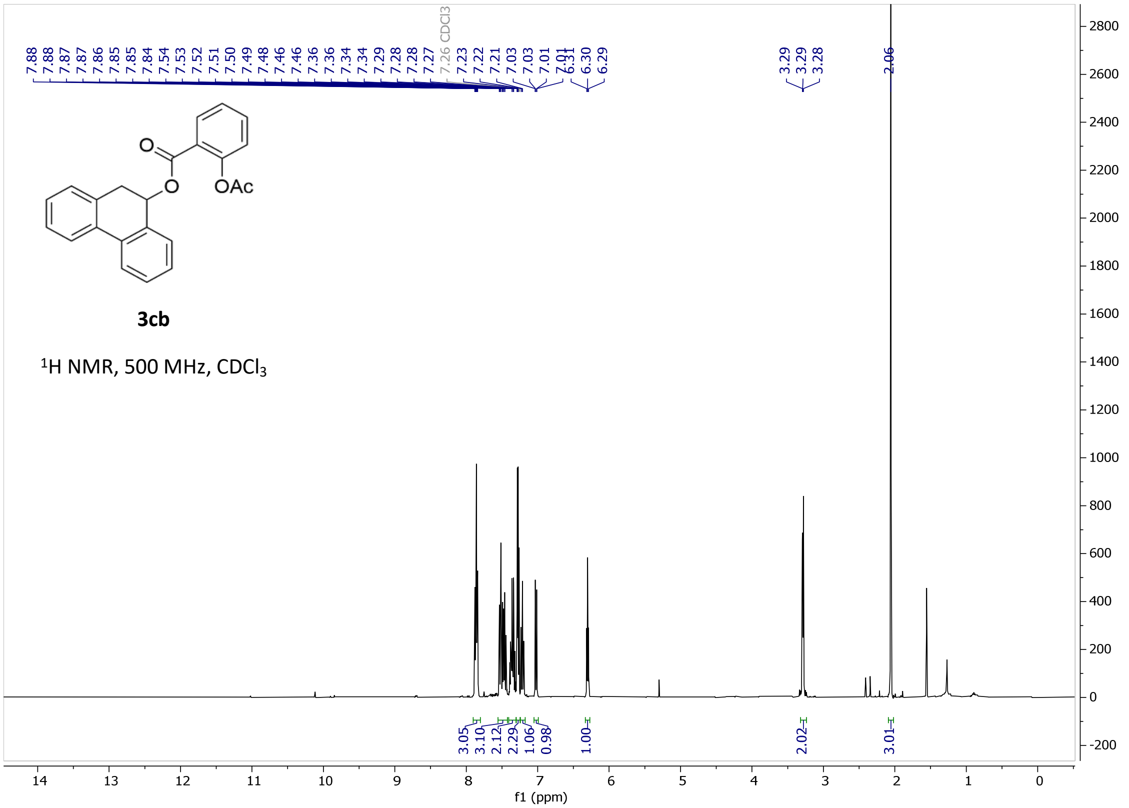

Supplementary Figure 188. ^{1}H NMR spectra of **3cb** (500 MHz, rt, $CDCl_3$).

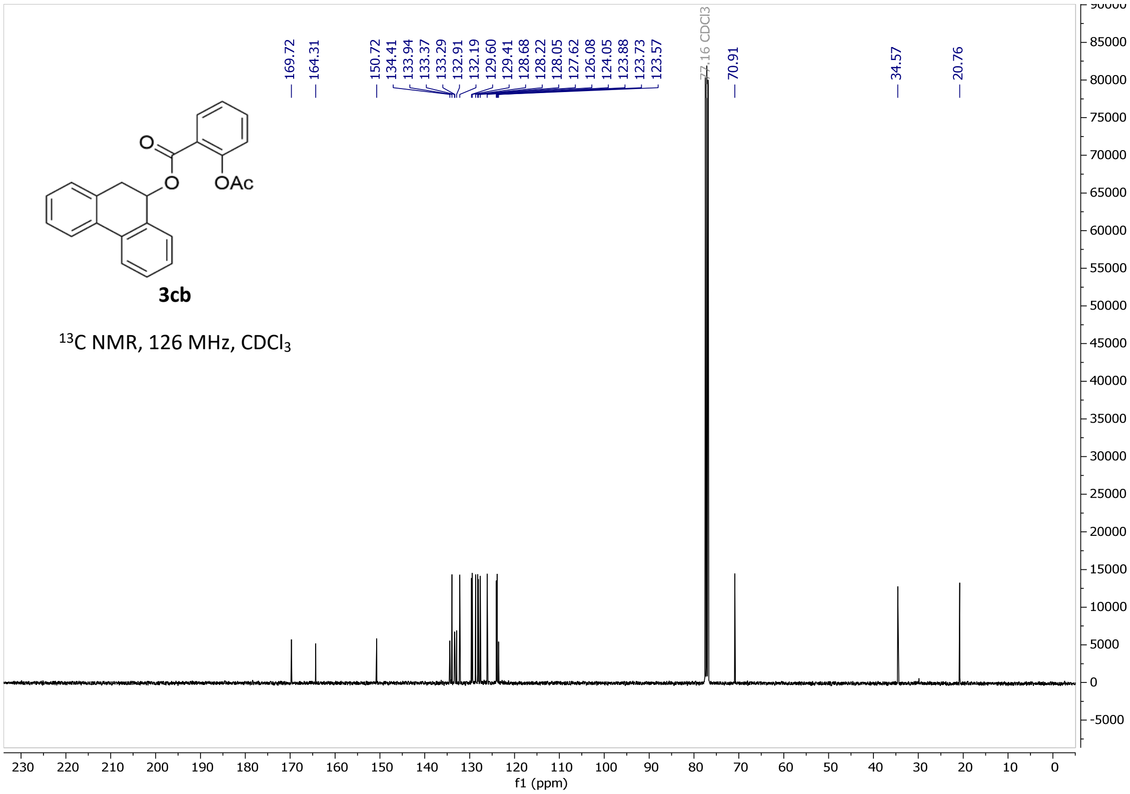

Supplementary Figure 189. ^{13}C NMR spectra of **3cb** (126 MHz, rt, $CDCl_3$).

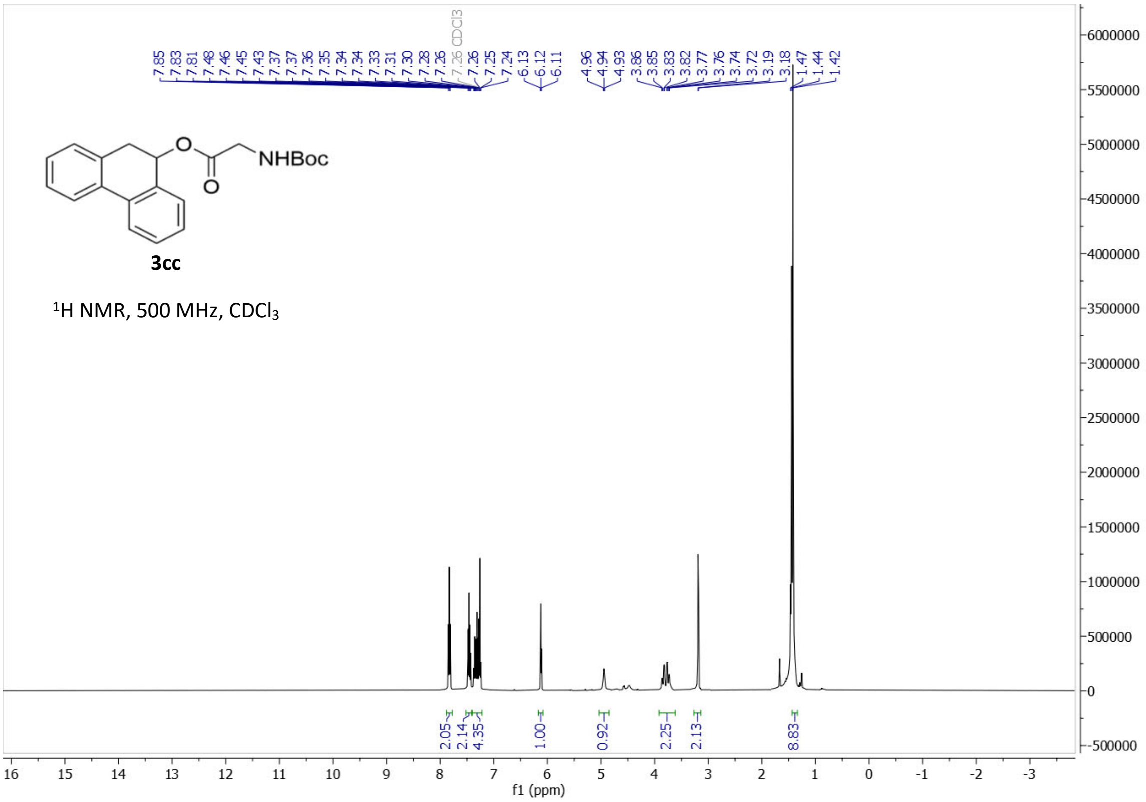

Supplementary Figure 190. ^{1}H NMR spectra of **3cc** (500 MHz, rt, $CDCl_3$).

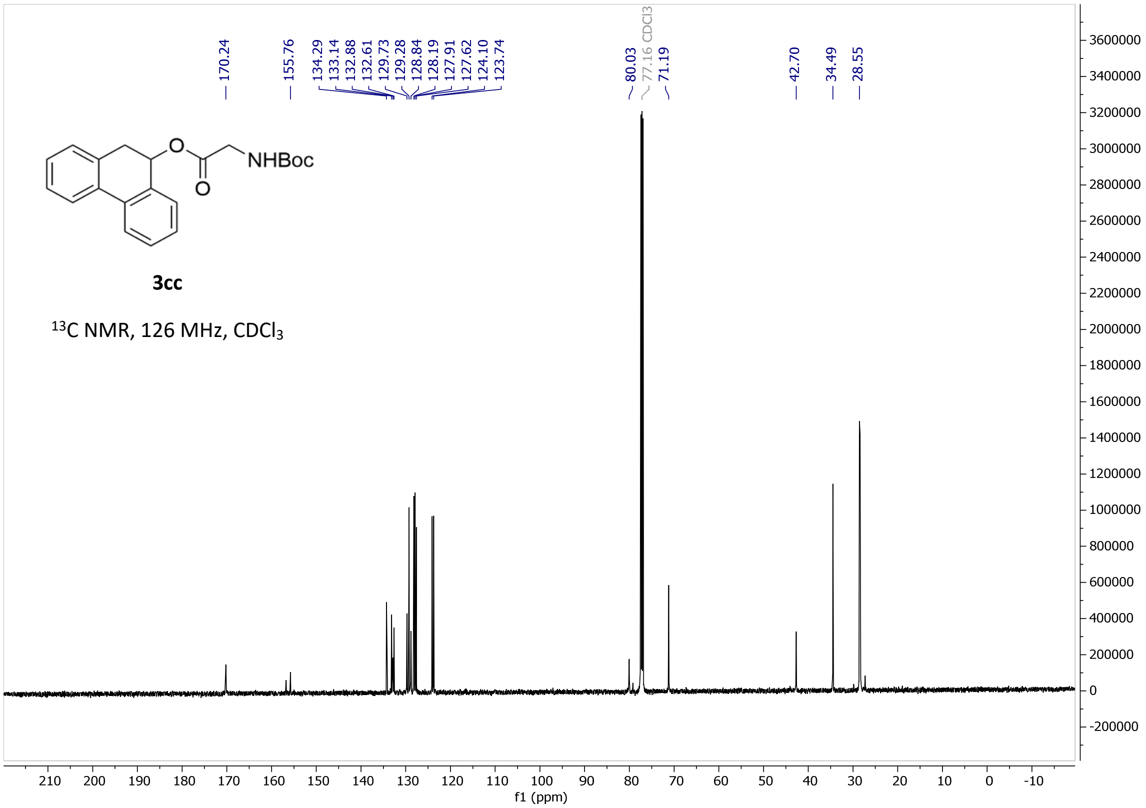

Supplementary Figure 191. ^{13}C NMR spectra of **3cc** (126 MHz, rt, $CDCl_3$).

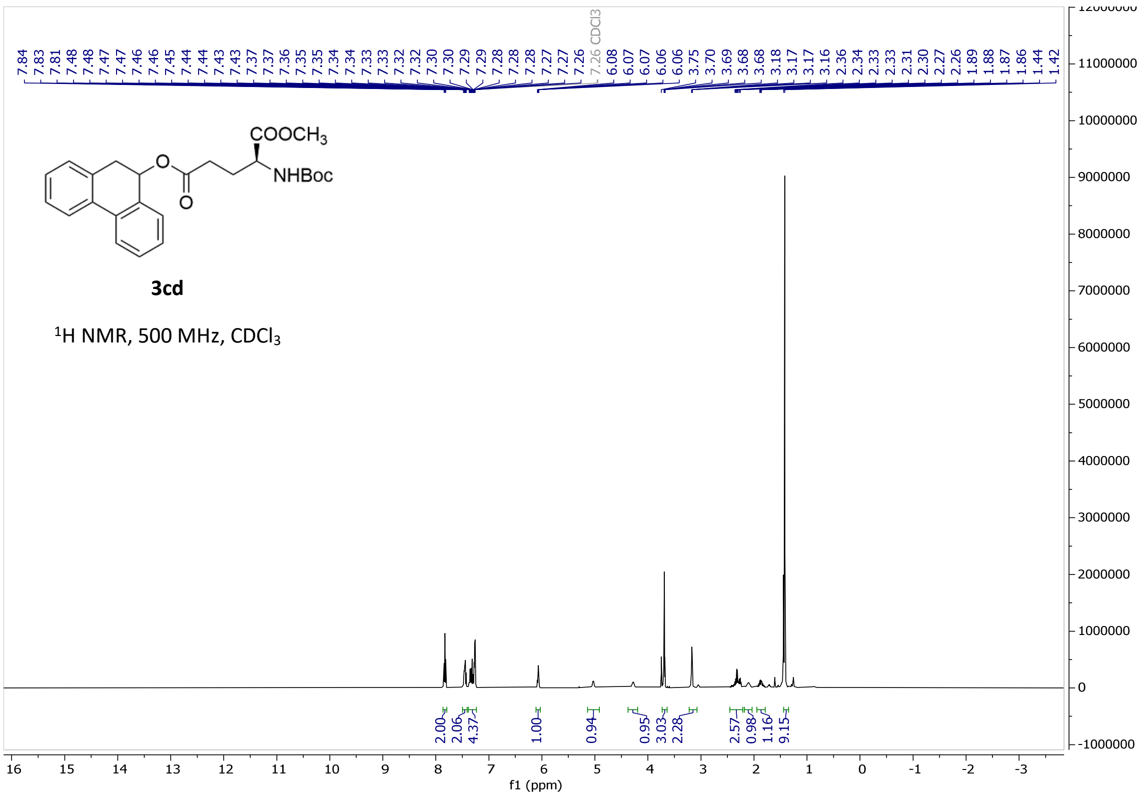

Supplementary Figure 192. ^{1}H NMR spectra of **3cd** (500 MHz, rt, $CDCl_3$).

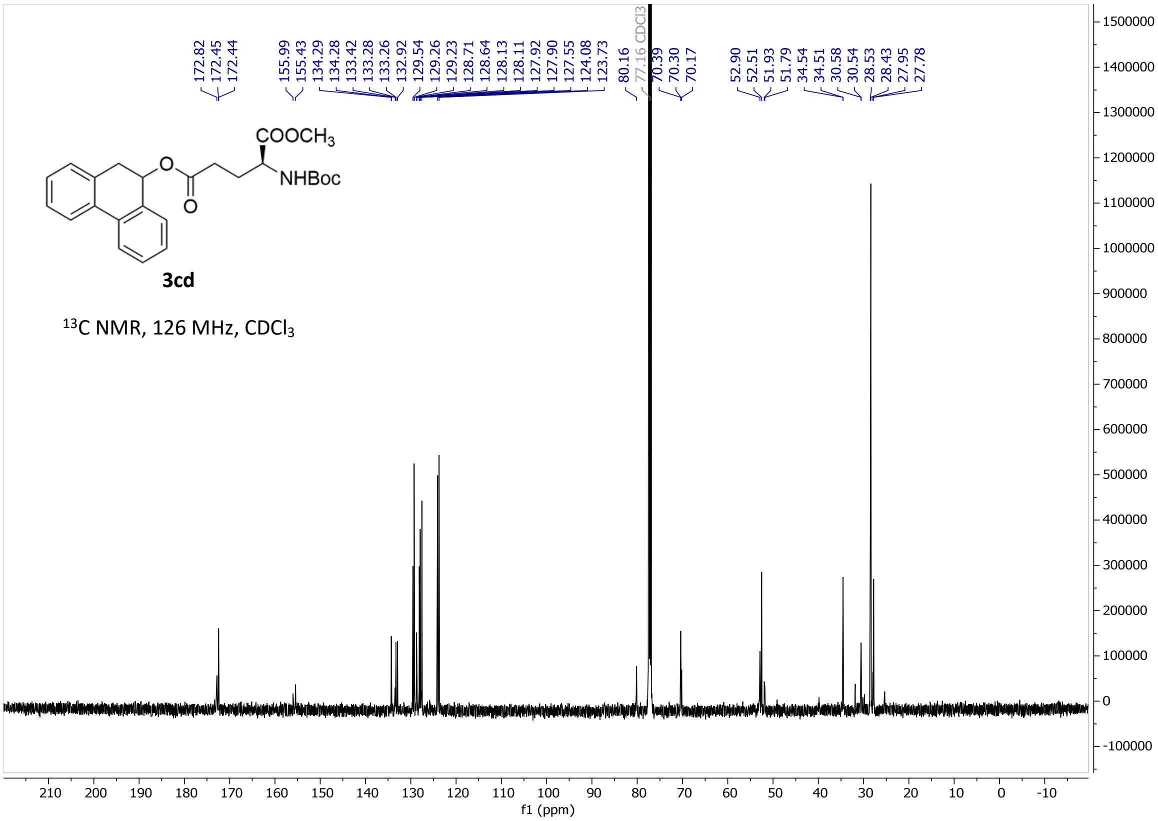

Supplementary Figure 193. ^{13}C NMR spectra of **3cd** (126 MHz, rt, $CDCl_3$).

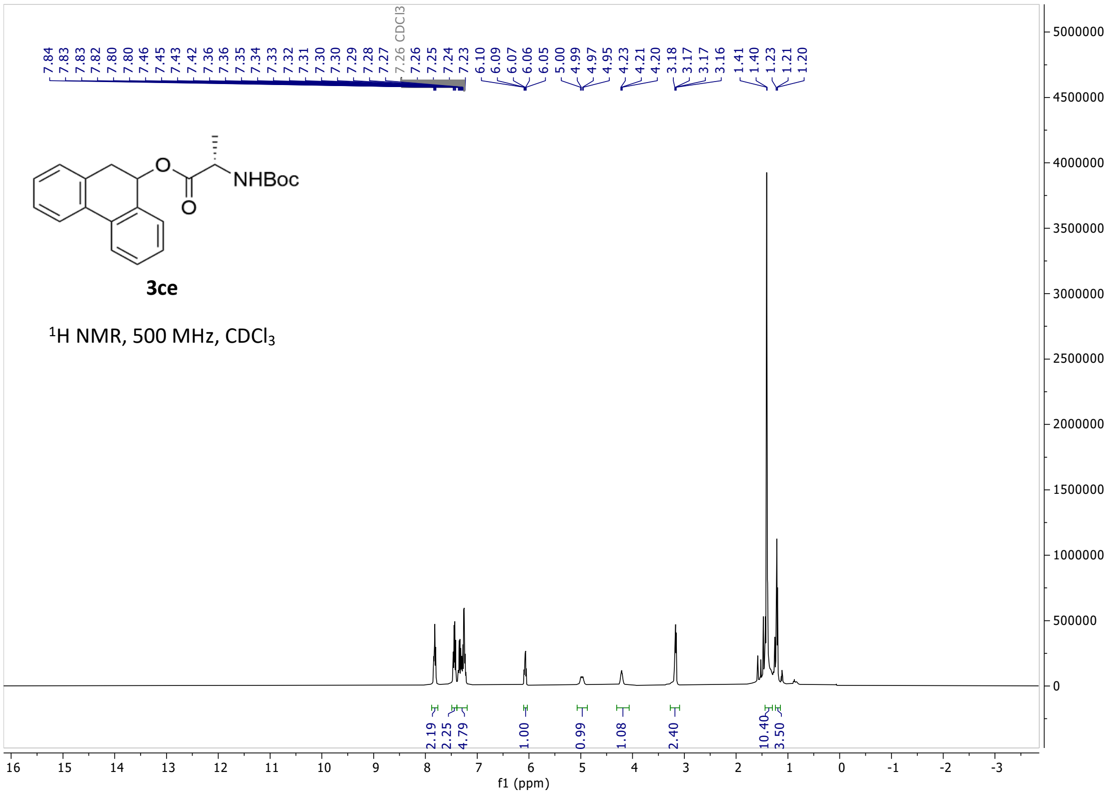

Supplementary Figure 194. ^{1}H NMR spectra of **3ce** (500 MHz, rt, $CDCl_3$).

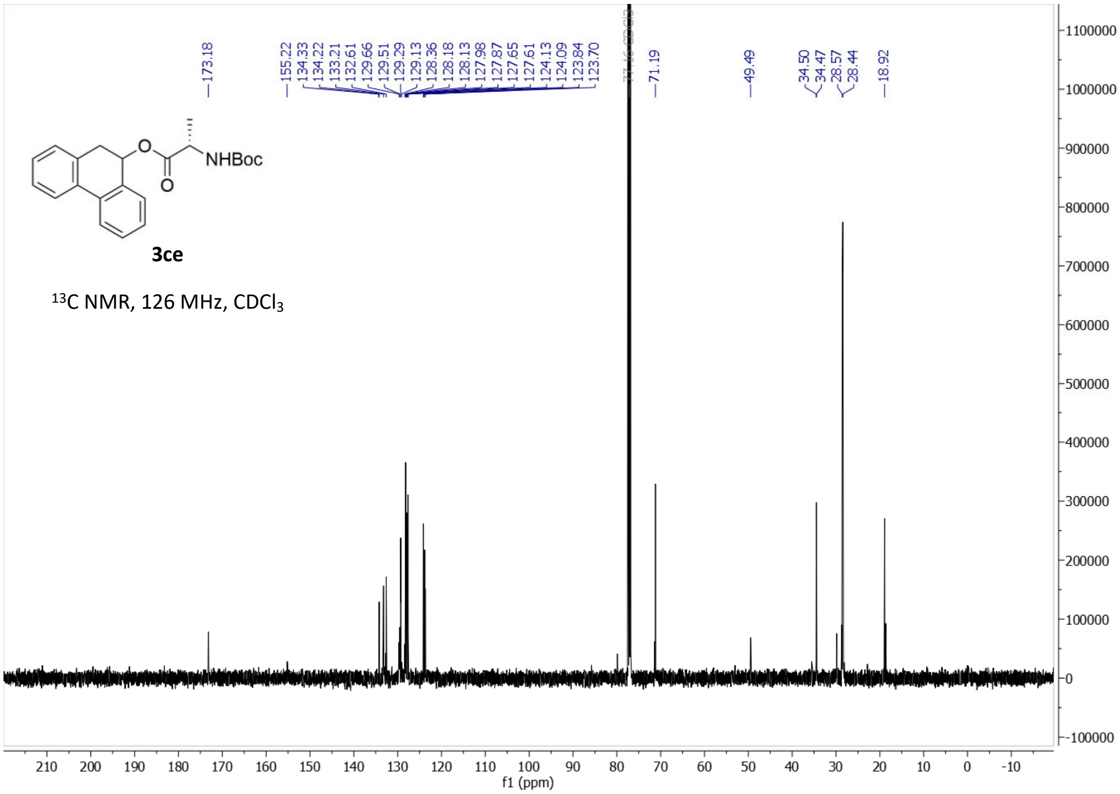

Supplementary Figure 195. ^{13}C NMR spectra of **3ce** (126 MHz, rt, $CDCl_3$).

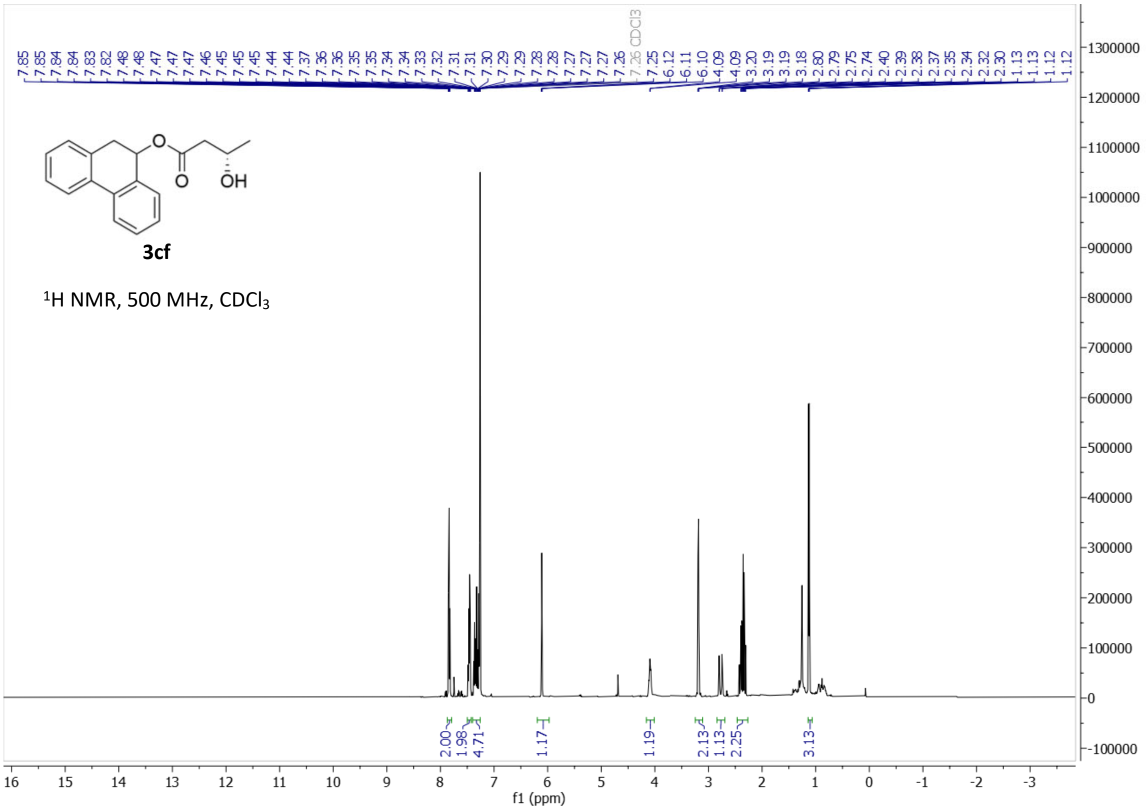

Supplementary Figure 196. ^{1}H NMR spectra of **3cf** (500 MHz, rt, $CDCl_3$).

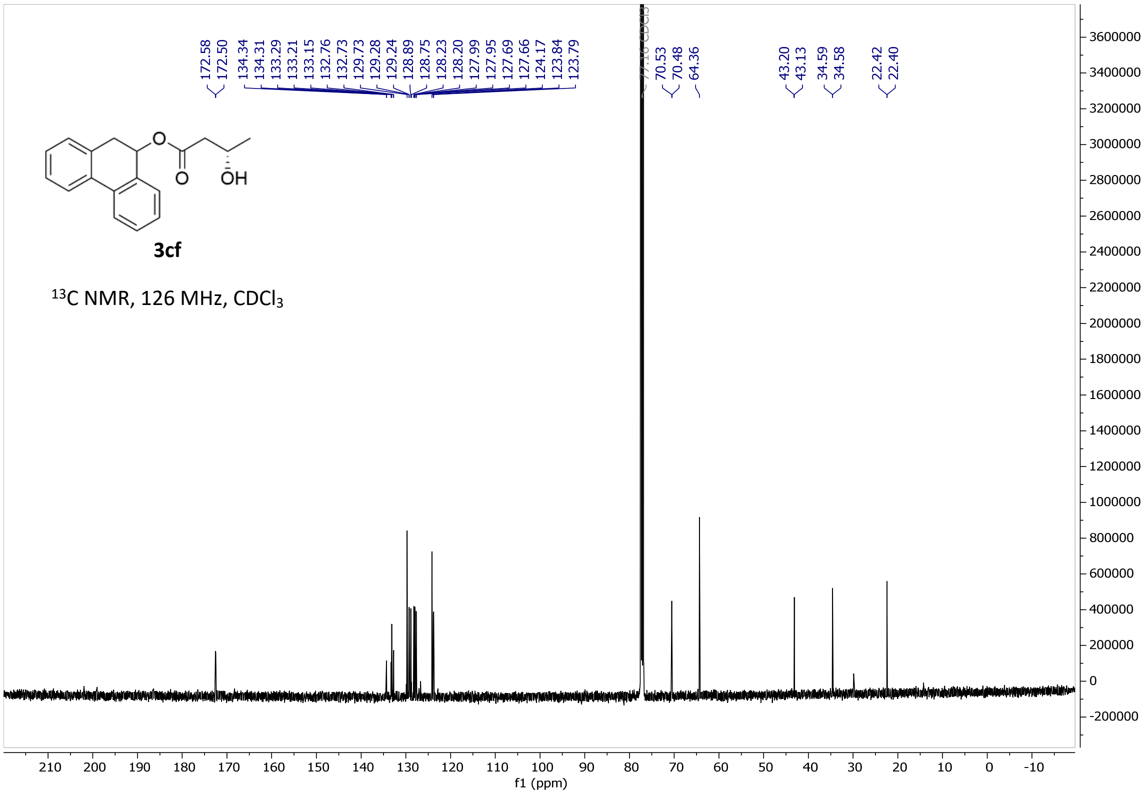

Supplementary Figure 197. ^{13}C NMR spectra of **3cf** (126 MHz, rt, $CDCl_3$).

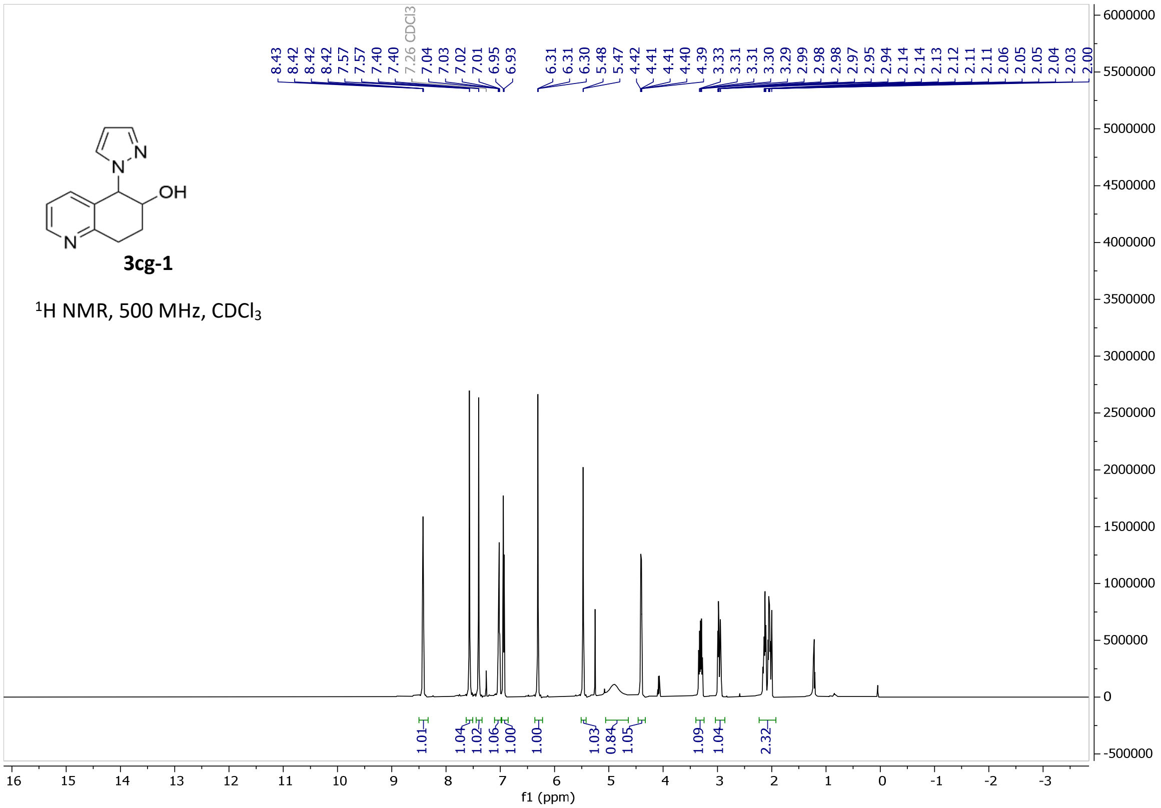

Supplementary Figure 198. ^{1}H NMR spectra of **3cg-1** (500 MHz, rt, $CDCl_3$).

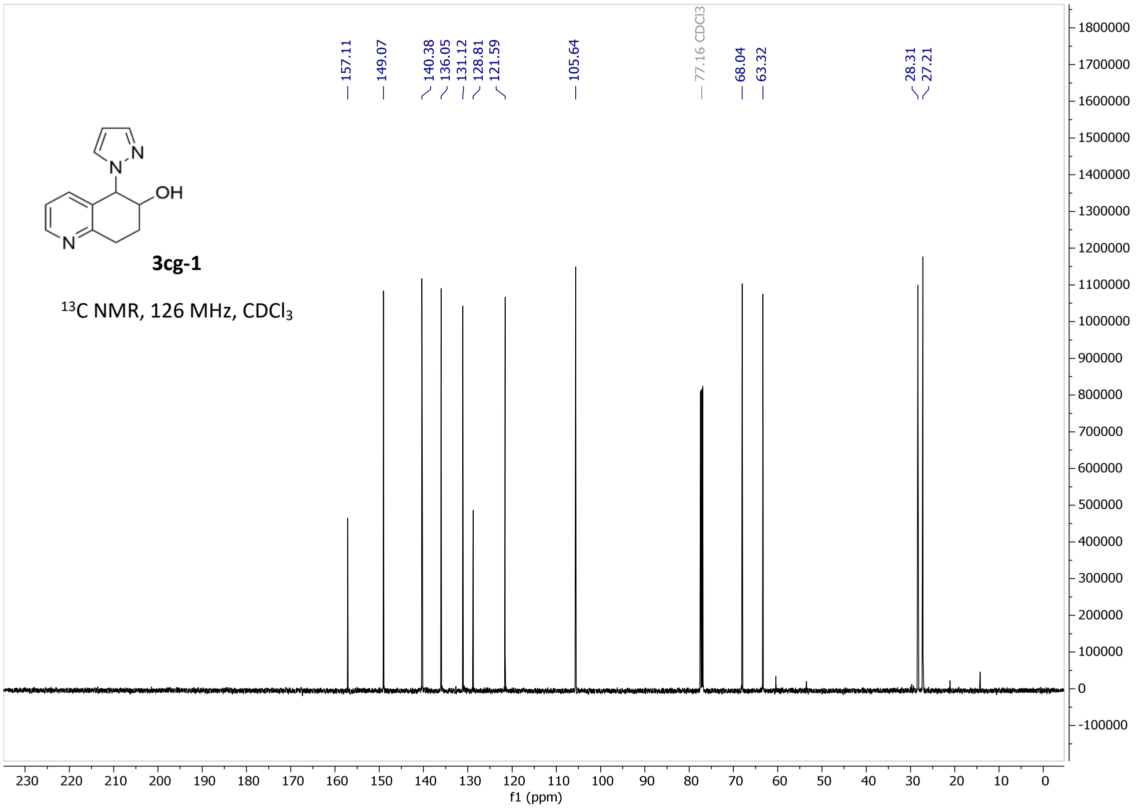

Supplementary Figure 199. ^{13}C NMR spectra of **3cg-1** (126 MHz, rt, $CDCl_3$).

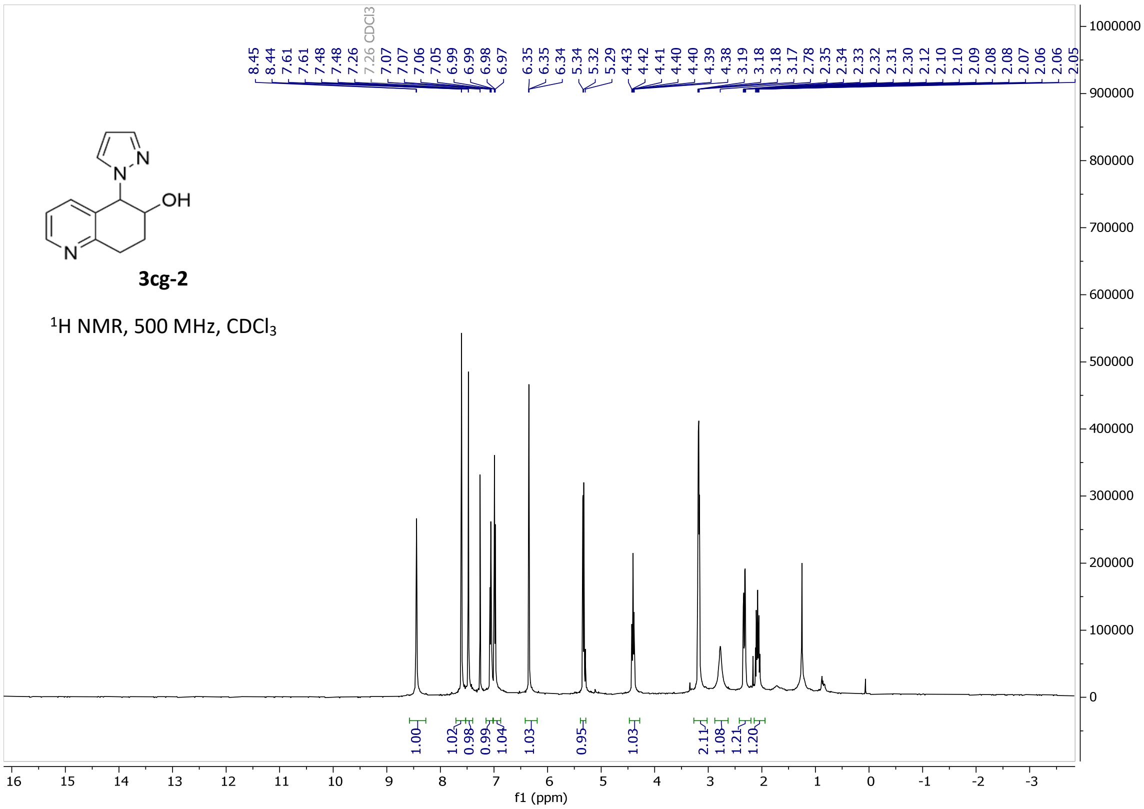

Supplementary Figure 200. ^{1}H NMR spectra of **3cg-2** (500 MHz, rt, CDCl$_3$).

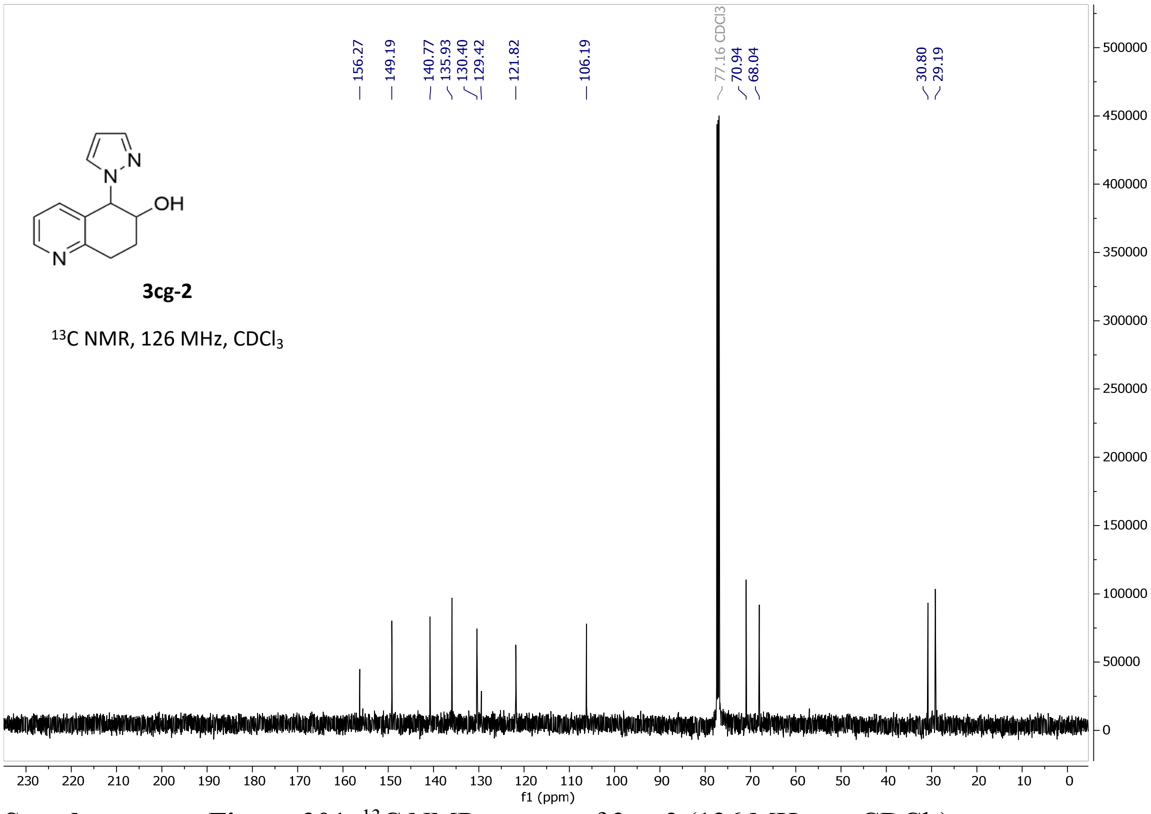

Supplementary Figure 201. ^{13}C NMR spectra of **3cg-2** (126 MHz, rt, CDCl$_3$).

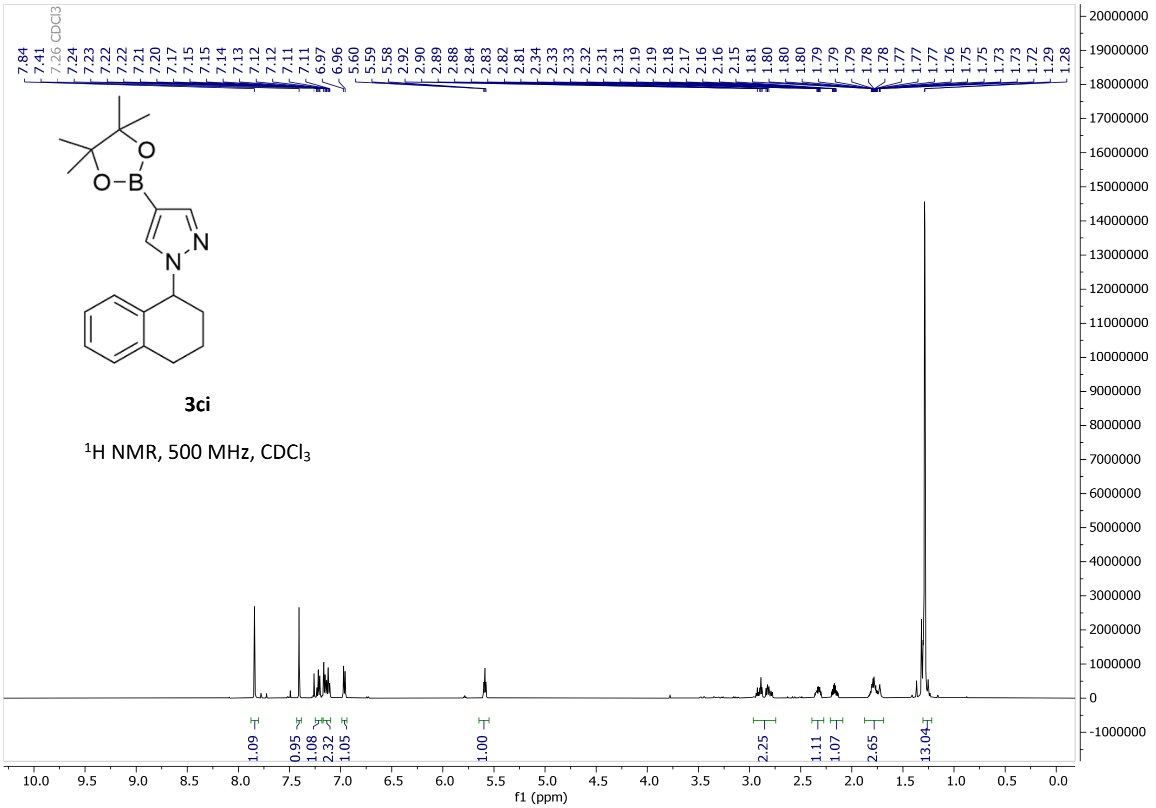

Supplementary Figure 202. ^{1}H NMR spectra of **3ci** (500 MHz, rt, $CDCl_3$).

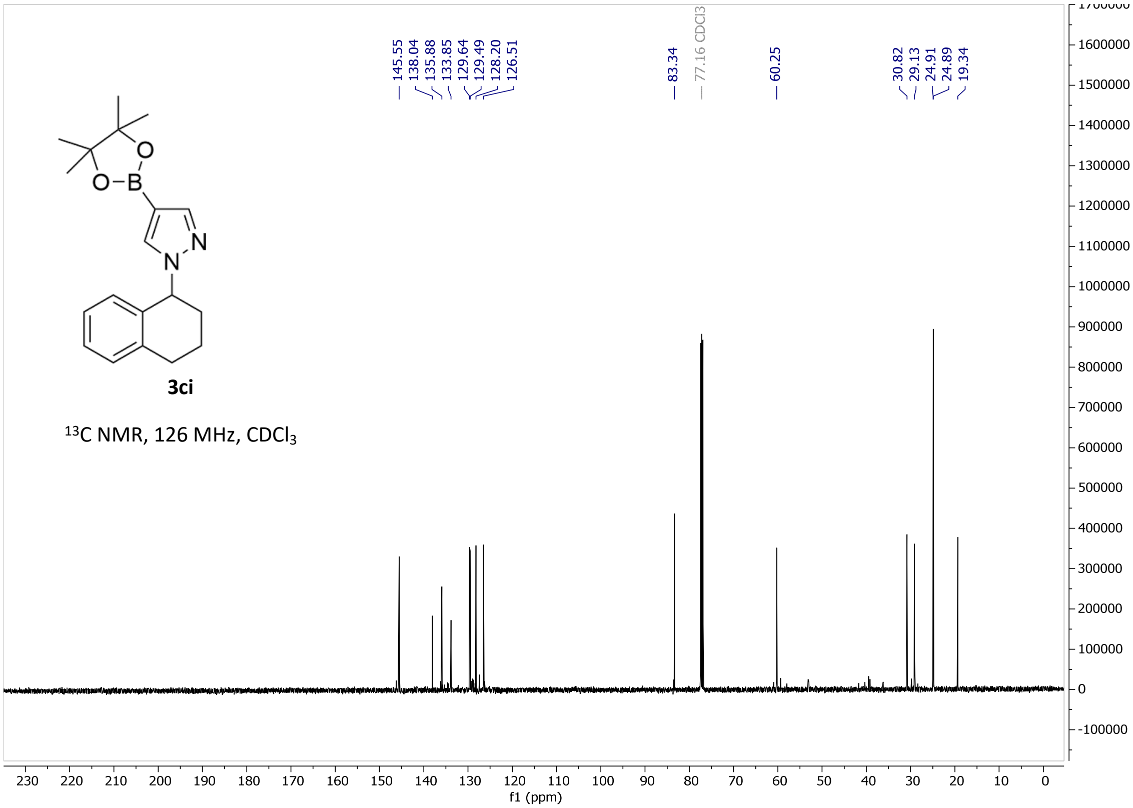

Supplementary Figure 203. ^{13}C NMR spectra of **3ci** (126 MHz, rt, $CDCl_3$).

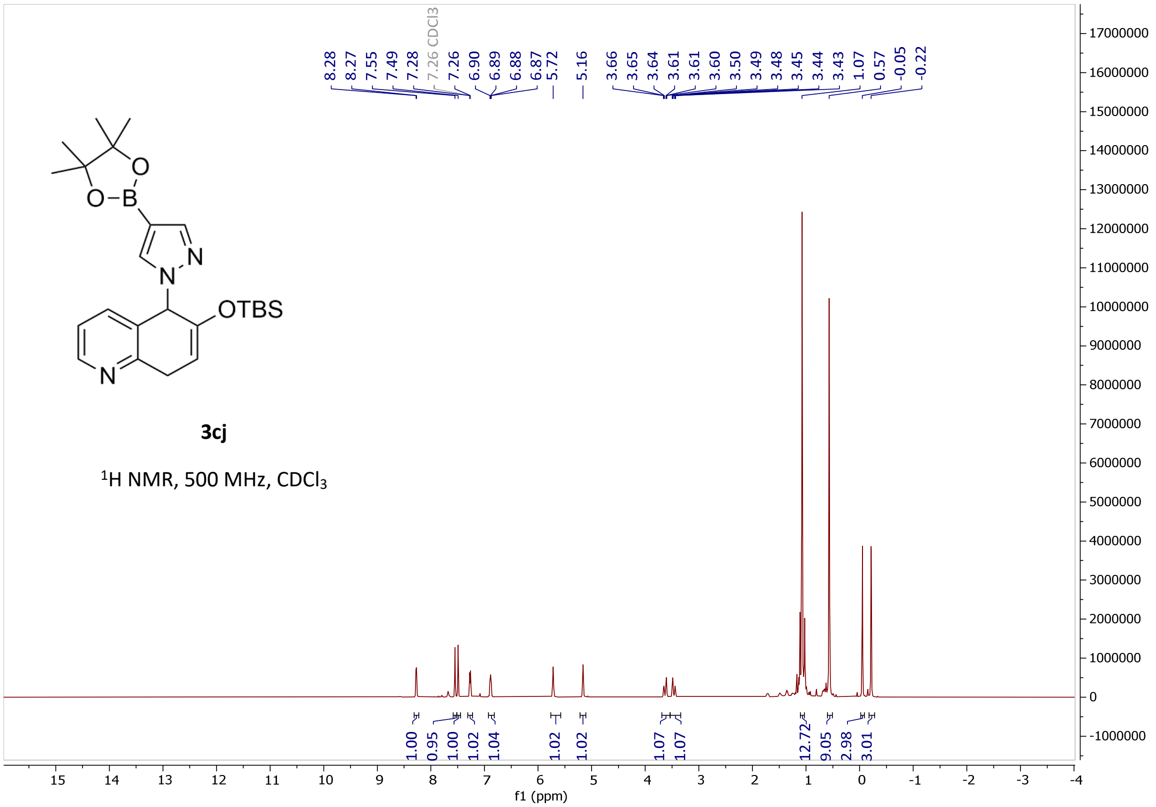

Supplementary Figure 204. ^{1}H NMR spectra of **3cj** (500 MHz, rt, $CDCl_3$).

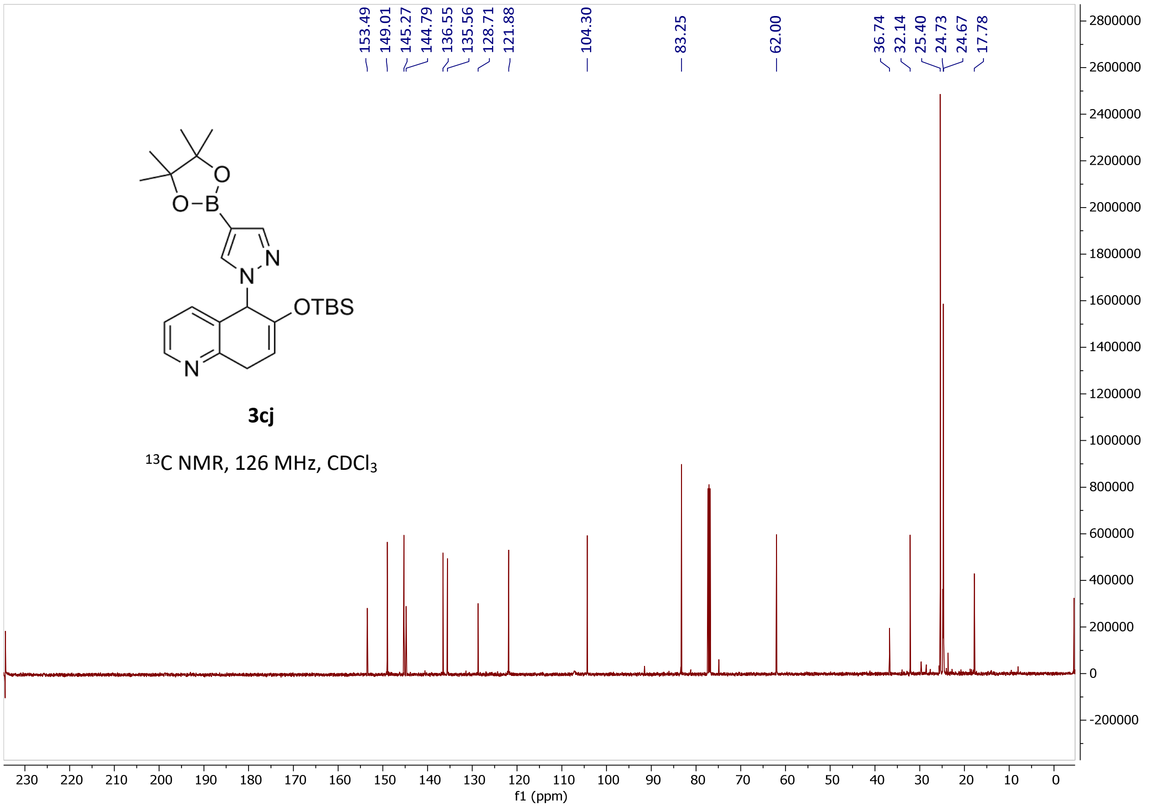

Supplementary Figure 205. ^{13}C NMR spectra of **3cj** (126 MHz, rt, $CDCl_3$).

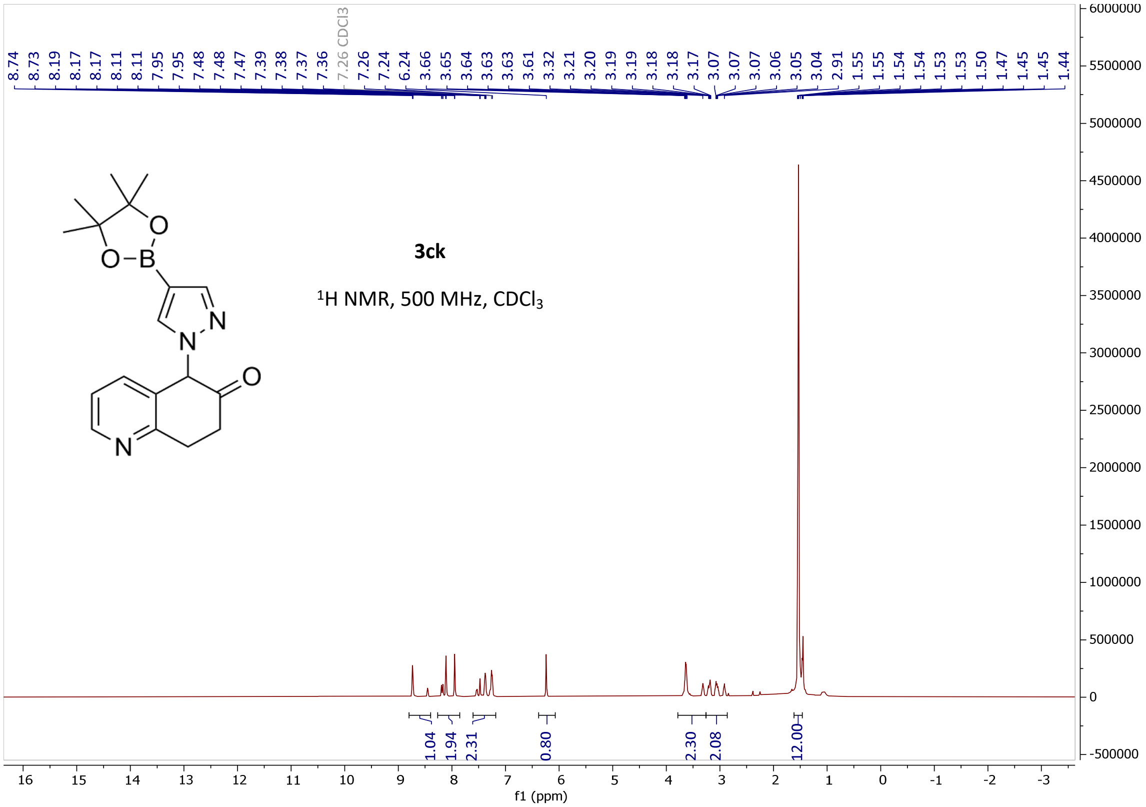

Supplementary Figure 206. ^1H NMR spectra of **3ck** (500 MHz, rt, $CDCl_3$).

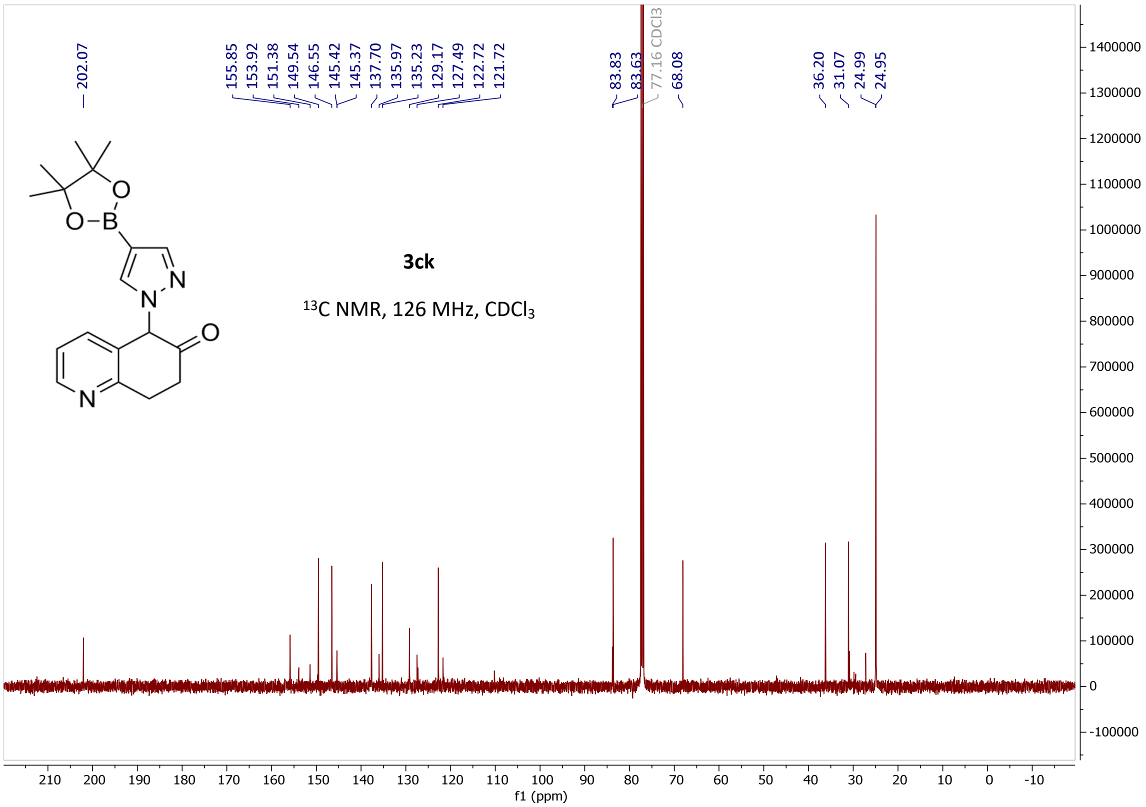

Supplementary Figure 207. ^{13}C NMR spectra of **3ck** (126 MHz, rt, $CDCl_3$).

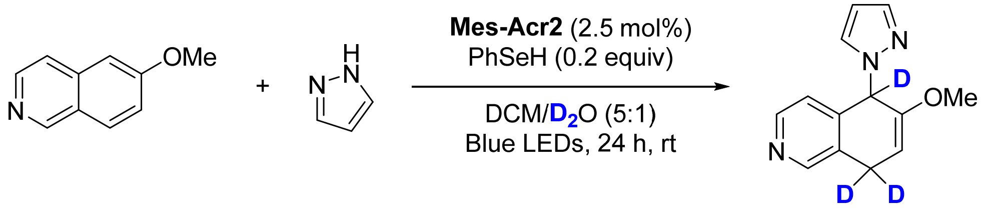

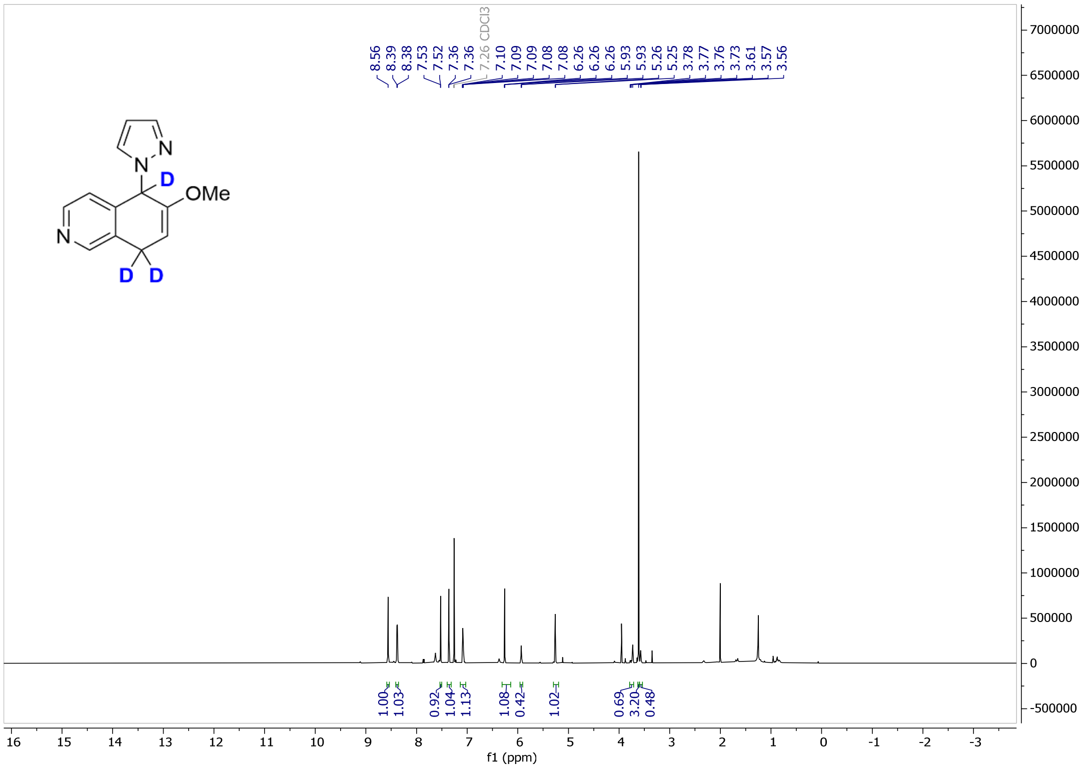

Supplementary Figure 208. ^{1}H NMR spectra of **3cp’** (500 MHz, rt, $CDCl_3$).

3. Supplementary References

(1) Yang, G.-Z.; Zhu, J.-K.; Yin, X.-D.; Yan, Y.-F.; Wang, Y.-L.; Shang, X.-F.; Liu, Y.-Q.; Zhao, Z.-M.; Peng, J.-W.; Liu, H. Design, Synthesis, and Antifungal Evaluation of Novel Quinoline Derivatives Inspired from Natural Quinine Alkaloids. Journal of Agricultural and Food Chemistry 2019, 67, 11340-11353.

(2) Davies, D. T.; Henry, C. J.; Pearson, N. D. Preparation of piperidinylpropylquinolines and related compounds as protein tyrosine kinase inhibitors. WO 2000043383.

(3) Zhang, Q.; Wang, D.; Wang, X.; Ding, K. (2-Pyridyl)acetone-Promoted Cu-Catalyzed O-Arylation of Phenols with Aryl Iodides, Bromides, and Chlorides. The Journal of Organic Chemistry 2009, 74, 7187-7190.

(4) Wang, P.; Du, J.; Liu, H.; Bi, G.; Zhang, G. Small quinolinium-based enzymatic probes via blue-to-red ratiometric fluorescence. Analyst 2016, 141, 1483-1487.

(5) Huang, H.; Strater, Z. M.; Rauch, M.; Shee, J.; Sisto, T. J.; Nuckolls, C.; Lambert, T. H. Electrophotocatalysis with a Trisaminocyclopropenium Radical Dication. Angewandte Chemie International Edition 2019, 58, 13318-13322.

(6) Ji, P.; Zhang, Y.; Gao, F.; Bi, F.; Wang, W. Direct, stereoselective thioglycosylation enabled by an organophotoredox radical strategy. Chemical science 2020, 11, 13079-13084.

(7) Gaussian 16, Revision A.03, Frisch, M. J.; Trucks, G. W.; Schlegel, H. B.; Scuseria, G. E.; Robb, M. A.; Cheeseman, J. R.; Scalmani, G.; Barone, V.; Petersson, G. A.; Nakatsuji, H.; Li, X.; Caricato, M.; Marenich, A. V.; Bloino, J.; Janesko, B. G.; Gomperts, R.; Mennucci, B.; Hratchian, H. P.; Ortiz, J. V.; Izmaylov, A. F.; Sonnenberg, J. L.; Williams-Young, D.; Ding, F.; Lipparini, F.; Egidi, F.; Goings, J.; Peng, B.; Petrone, A.; Henderson, T.; Ranasinghe, D.; Zakrzewski, V. G.; Gao, J.; Rega, N.; Zheng, G.; Liang, W.; Hada, M.; Ehara, M.; Toyota, K.; Fukuda, R.; Hasegawa, J.; Ishida, M.; Nakajima, T.; Honda, Y.; Kitao, O.; Nakai, H.; Vreven, T.; Throssell, K.; Montgomery, Jr., J. A.; Peralta, J. E.; Ogliaro, F.; Bearpark, M. J.; Heyd, J. J.; Brothers, E. N.; Kudin, K. N.; Staroverov, V. N.; Keith, T. A.; Kobayashi, R.; Normand, J.; Raghavachari, K.; Rendell, A. P.; Burant, J. C.; Iyengar, S. S.; Tomasi, J.; Cossi, M.; Millam, J. M.; Klene, M.; Adamo, C.; Cammi, R.; Ochterski, J. W.; Martin, R. L.; Morokuma, K.; Farkas, O.; Foresman, J. B.; and Fox, D. J. Gaussian, Inc., Wallingford CT, 2016.

(8) Chai, J.-D. & Head-Gordon, M. Long-range corrected hybrid density functionals with damped atom–atom dispersion corrections. PCCP 10, 6615-6620 (2008).

(9) Grimme, S. Supramolecular binding thermodynamics by dispersion‐corrected density functional theory. Chem. Eur. J. 18, 9955-9964 (2012).

(10) Marenich, A. V., Cramer, C. J. & Truhlar, D. G. Universal solvation model based on solute electron density and on a continuum model of the solvent defined by the bulk dielectric constant and atomic surface tensions. J. Phy. Chem. B 113, 6378-6396 (2009).

(11) Legault, C. Y. CYLview, 1.0b; Université de Sherbrooke, 2009; http://www.cylview.org.
Spartan ’20, Wavefunction, Inc.

(12) Lu, T.; Chen, F. Multiwfn: A multifunctional wavefunction analyzer. J. Comput. Chem. 2012, 33, 580−592.

(13) Humphrey, W.; Dalke, A.; Schulten, K. VMD: Visual Molecular Dynamics. J. Mol. Graph. 1996, 14, 33−38.
