# Supplementary material for: Selective skeletal editing of polycyclic arenes using organophotoredox dearomative functionalization
Source: Nat Commun. 2022 Aug 5;13:4565. doi: 10.1038/s41467-022-32201-7 (PMC9355940; doi:10.1038/s41467-022-32201-7)
Supplement: Supplementary file 2 — Description of Additional Supplementary Files [file 41467_2022_32201_MOESM2_ESM.docx]

**Description of Additional Supplementary Files**

**File Name: Supplementary Data 1
Description:** Cartesian Coordinates of Calculated Structures.
