# Supplementary material for: Selective skeletal editing of polycyclic arenes using organophotoredox dearomative functionalization
Source: Nat Commun. 2022 Aug 5;13:4565. doi: 10.1038/s41467-022-32201-7 (PMC9355940; doi:10.1038/s41467-022-32201-7)
Supplement: Supplementary file 3 — Supplementary Data 1 [file 41467_2022_32201_MOESM3_ESM.pdf]

Cartesian Coordinates of Calculated Structures

S1

C	3.24608900	0.49136400	-0.00011100
C	0.98036300	0.76316100	-0.00008900
C	0.76201800	-0.65339700	0.00004700
C	1.90840500	-1.49990700	0.00010200
C	3.15465400	-0.91734300	0.00001800
H	0.04997300	2.72384500	-0.00030700
H	4.22884300	0.97340700	-0.00019300
C	-0.16290000	1.65284500	-0.00017200
C	-0.54675600	-1.13504400	0.00012300
H	1.78663800	-2.58542900	0.00020500
H	4.06325300	-1.52054900	0.00004800
C	-1.65672900	-0.22749800	0.00008600
C	-1.44288900	1.18329800	-0.00009700
H	-2.28476800	1.87481800	-0.00018600
N	2.19461500	1.30557700	-0.00017300
H	-0.76716100	-2.20510400	0.00022600
O	-2.82229300	-0.79861200	0.00012100
C	-4.04361500	-0.05016200	0.00011100
H	-4.84470600	-0.79461100	0.00027400
H	-4.10387100	0.56991600	0.90541200
H	-4.10400400	0.56965600	-0.90535900

S2

C	-3.02289600	-1.04662200	0.00003500
C	-0.77551000	-0.61834200	-0.00011300

C	-0.97634100	0.79506000	-0.00001300
C	-2.29527900	1.26578700	0.00019000
C	-3.32805500	0.33794100	0.00026500
H	0.70888200	-2.19524300	-0.00034300
H	-3.83338300	-1.78215000	-0.00002400
C	0.53380500	-1.11770200	-0.00020100
C	0.17207600	1.66845800	-0.00016800
H	-2.50398800	2.33794700	0.00029800
H	-4.37031100	0.66184000	0.00049800
C	1.45119900	1.18888200	-0.00028300
C	1.65340600	-0.22212200	-0.00011100
N	-1.79668700	-1.51338100	-0.00013900
H	0.00198100	2.74803300	-0.00051100
H	2.29699000	1.87525000	-0.00050300
O	2.81285300	-0.80393600	0.00011400
C	4.04149000	-0.06830700	0.00032100
H	4.83510000	-0.82075000	-0.00000300
H	4.10771300	0.55134500	-0.90494500
H	4.10762700	0.55068300	0.90605400

S3

C	-3.22922700	0.43012300	-0.00004700
C	-0.99469700	0.76883500	-0.00000900
C	-0.78587900	-0.64062100	-0.00002200
C	-3.02597900	-0.98514800	-0.00006700
H	-0.06579900	2.73384100	0.00004100
H	-4.24497700	0.83825800	-0.00006500
C	0.14523000	1.66227300	0.00002700

C	0.52295500	-1.13577200	-0.00000600
H	-3.88645400	-1.66141700	-0.00009300
C	1.63382600	-0.22644100	0.00002000
C	1.42235500	1.18908100	0.00004200
H	2.26676600	1.87759300	0.00007200
N	-2.22903300	1.28885300	-0.00002100
H	0.70877500	-2.21179800	-0.00001600
O	2.79588400	-0.79626700	0.00004100
C	4.02504400	-0.05623500	0.00006800
H	4.81884800	-0.80826700	0.00006400
H	4.08723400	0.56214700	-0.90603300
H	4.08721200	0.56211200	0.90619500
N	-1.83103000	-1.51155300	-0.00005300

S4

C	-0.96984400	0.76869300	-0.00000600
C	-0.75766700	-0.64185700	-0.00003600
C	-1.89096500	-1.48222800	-0.00008600
C	-3.16156700	-0.88755300	-0.00010400
H	-0.01415600	2.72520300	0.00007100
C	0.16119300	1.64613000	0.00004600
C	0.56034900	-1.13350700	-0.00001400
H	-1.78629100	-2.56897900	-0.00011000
H	-4.05953900	-1.51300800	-0.00014300
C	1.67078100	-0.22650600	0.00003600
C	1.45184500	1.17336100	0.00006800
H	2.28946200	1.86968800	0.00011000
H	0.77552400	-2.20441900	-0.00003400

O	2.83741600	-0.80075100	0.00005500
C	4.05222200	-0.04578700	0.00010100
H	4.85932600	-0.78383600	0.00009600
H	4.11043100	0.57567300	-0.90489900
H	4.11039400	0.57562100	0.90513800
C	-2.28873900	1.23443800	-0.00002800
N	-3.35093600	0.42686200	-0.00007600
H	-2.49356900	2.31093100	-0.00000600

S5

C	3.35352300	0.40243400	-0.00009800
C	0.95756700	0.79015600	-0.00009800
C	0.74615600	-0.62826300	0.00005200
C	1.87342800	-1.50176900	0.00012500
C	3.15863200	-0.98420500	0.00004900
H	-0.01848600	2.73905800	-0.00031200
H	4.36754600	0.80697500	-0.00015900
C	-0.18657100	1.65931200	-0.00018000
C	-0.55919500	-1.11861100	0.00012800
H	1.71077000	-2.58176100	0.00023900
H	4.01872000	-1.65535400	0.00010300
C	-1.67585000	-0.22604200	0.00007500
C	-1.46980700	1.17886400	-0.00010400
H	-2.31323200	1.86806100	-0.00018800
H	-0.76862100	-2.19052300	0.00024200
O	-2.84185800	-0.80732100	0.00011800
C	-4.05796100	-0.05808400	0.00008700
H	-4.86288700	-0.79869700	0.00020900

H	-4.12112000	0.56373100	0.90457400
H	-4.12120300	0.56351600	-0.90454300
C	2.26143300	1.28321300	-0.00017200
H	2.43524900	2.36153900	-0.00028900

S6

C	-2.96525200	0.32941500	0.00000100
C	-0.56973700	0.79508100	0.00000000
C	-0.31057700	-0.61048800	0.00000000
C	-1.40320000	-1.51410000	0.00000000
C	-2.71890100	-1.04008800	0.00000000
H	0.34712900	2.76581700	0.00000000
H	-3.99201200	0.69997100	0.00000100
C	0.53405900	1.68895800	0.00000000
C	1.02012300	-1.05809200	-0.00000100
H	-1.21013400	-2.58965500	-0.00000100
H	-3.55063700	-1.74642200	0.00000000
C	2.11782100	-0.15426500	-0.00000100
C	1.84573700	1.21717400	-0.00000100
H	2.67199400	1.93062700	-0.00000100
H	1.22131100	-2.13337900	-0.00000100
C	-1.89779600	1.24637800	0.00000000
H	-2.10531400	2.31919600	0.00000000
C	3.51354400	-0.68672500	0.00000100
H	3.68146400	-1.32114800	0.88484700
H	3.68145600	-1.32118000	-0.88482300
H	4.25980400	0.11668800	-0.00001600

S7

C	2.44841500	0.69402700	0.00000000
C	0.00000000	0.71482500	0.00000000
C	-0.00012200	-0.71501100	0.00000000
C	1.23397100	-1.40129200	0.00000000
C	2.44834200	-0.69528400	0.00000000
H	-1.24154300	2.49402200	0.00000000
H	3.39182400	1.24283600	0.00000000
C	-1.23375400	1.40115100	0.00000000
C	-1.23358100	-1.40064100	0.00000000
H	1.24119800	-2.49417600	0.00000000
H	3.39196100	-1.24370400	0.00000000
C	-2.44839100	-0.69401000	0.00000000
C	-2.44851900	0.69521800	0.00000000
H	-3.39157500	1.24453500	0.00000000
H	-1.24235300	-2.49351100	0.00000000
C	1.23362000	1.40089900	0.00000000
H	1.24229500	2.49374400	0.00000000
H	-3.39169900	-1.24304300	0.00000000

S8

C	3.66217900	0.70192200	0.00005100
C	2.46587700	1.40678500	0.00003000
C	1.23291600	0.71768400	-0.00000200
C	1.23291600	-0.71768500	-0.00002300
C	2.46587900	-1.40678400	-0.00003600

C	3.66218100	-0.70192000	0.00001300
C	-0.00000100	1.40351500	-0.00003500
C	0.00000100	-1.40351500	-0.00001400
C	-1.23291500	-0.71768600	-0.00000800
C	-1.23291600	0.71768400	-0.00003900
C	-2.46587700	1.40678400	-0.00003100
H	-2.47175400	2.49888000	-0.00007300
C	-3.66218000	0.70192100	0.00001900
C	-3.66217900	-0.70192100	0.00004900
C	-2.46587700	-1.40678500	0.00001900
H	-0.00000600	2.49697400	-0.00006200
H	4.61043700	1.24225500	0.00007800
H	2.47175100	2.49888000	0.00005200
H	2.47175300	-2.49887900	-0.00007100
H	4.61043400	-1.24226000	0.00000800
H	-0.00000500	-2.49697500	-0.00006400
H	-4.61043500	1.24225900	0.00003900
H	-4.61043400	-1.24225900	0.00008600
H	-2.47175600	-2.49888000	0.00004900

S9

C	-1.89394500	-0.60415000	-0.00010100
C	-1.98911000	0.79653300	-0.00002600
C	-0.75518900	1.41364800	0.00003500
C	0.30943100	0.45920800	0.00002100
S	-0.27376500	-1.19385700	0.00006600
H	-2.72230300	-1.31577800	-0.00014200
H	-2.94583700	1.31910900	-0.00006000

H	-0.55999900	2.48646700	0.00004400
O	1.53995400	0.79994800	0.00004700
C	2.60829400	-0.17012000	-0.00008600
H	3.53495100	0.41002300	-0.00041900
H	2.53869200	-0.78404100	0.90921900
H	2.53821200	-0.78436300	-0.90913400

PC^{+}

C	1.26289900	-3.61299600	0.00038600
C	1.95430000	-2.42616100	0.00020800
C	1.24262300	-1.19991400	0.00014700
C	-0.18428000	-1.21604000	0.00025700
C	-0.85910300	-2.47505300	0.00046200
C	-0.15400800	-3.64660700	0.00052400
C	-0.89708000	0.00035000	0.00015000
C	-0.18462700	1.21691500	-0.00003700
C	1.24228300	1.20119000	-0.00009300
C	1.95359000	2.42765800	-0.00023600
H	3.04218100	2.42664500	-0.00026600
C	1.26184700	3.61429200	-0.00033300
C	-0.15506800	3.64749100	-0.00030200
C	-0.85979300	2.47571300	-0.00016000
H	1.82168800	-4.55098800	0.00042800
H	3.04289100	-2.42482900	0.00011500
H	-1.95014300	-2.47411800	0.00057800
H	-0.67435600	-4.60518500	0.00068200
H	1.82036400	4.55244600	-0.00043800
H	-0.67567100	4.60593000	-0.00038900

H	-1.95081400	2.47429400	-0.00012100
C	-2.38481700	-0.00009400	0.00022100
C	-3.07306300	-0.00359500	-1.22604400
C	-3.07289000	-0.00268900	1.22630200
C	-4.46826400	-0.00905500	-1.19964000
C	-4.46835400	-0.00812700	1.20000300
C	-5.18551500	-0.00935100	0.00031100
H	-5.01267600	-0.01520100	-2.14781000
H	-5.01260500	-0.01355500	2.14823300
N	1.90456000	0.00073800	-0.00001100
C	3.35175900	0.00094700	-0.00008500
C	4.03084100	0.00098000	-1.21593900
C	4.03096000	0.00111500	1.21570100
C	5.42425600	0.00119900	-1.21010900
H	3.47209000	0.00083700	-2.15400100
C	5.42437600	0.00133200	1.20973400
H	3.47230400	0.00107500	2.15382000
C	6.11875900	0.00137600	-0.00022100
H	5.96883700	0.00122900	-2.15582900
H	5.96904900	0.00146500	2.15540100
H	7.21021800	0.00154600	-0.00027600
C	-2.32202200	-0.00532200	-2.53279000
H	-1.67406000	0.88052800	-2.62571600
H	-1.67572900	-0.89255700	-2.62452400
H	-3.01134800	-0.00524300	-3.38675200
C	-2.32196900	-0.00348300	2.53311800
H	-1.67547800	-0.89051300	2.62543100
H	-1.67422900	0.88257600	2.62560100
H	-3.01137300	-0.00307100	3.38701600
C	-6.68996700	0.01753800	-0.00081500

H	-7.05572000	1.05635600	-0.03096600
H	-7.10107900	-0.50141400	-0.87768000
H	-7.10229600	-0.45107500	0.90322300

PC*⁺

C	1.29489300	-3.65371700	0.00086400
C	1.96415300	-2.42097500	0.00048000
C	1.24215600	-1.21893800	0.00032300
C	-0.18750800	-1.23318300	0.00047900
C	-0.82259300	-2.47391800	0.00088100
C	-0.08696600	-3.67913700	0.00108100
C	-0.92653800	0.00044200	0.00018900
C	-0.18779100	1.23418500	-0.00013200
C	1.24184800	1.22022600	-0.00019000
C	1.96359400	2.42231300	-0.00039000
H	3.05299200	2.40411700	-0.00037900
C	1.29408300	3.65492900	-0.00059700
C	-0.08779800	3.68011600	-0.00059400
C	-0.82313700	2.47481700	-0.00035600
H	1.87244100	-4.57851500	0.00098700
H	3.05354600	-2.40255900	0.00029300
H	-1.91408400	-2.49986000	0.00103400
H	-0.62088600	-4.63109300	0.00139500
H	1.87146300	4.57983300	-0.00075200
H	-0.62184000	4.63200300	-0.00075800
H	-1.91461600	2.50039300	-0.00031900
C	-2.41004600	-0.00006200	0.00021200
C	-3.10597700	-0.00409500	-1.22327700

C	-3.10588900	-0.00221500	1.22346800
C	-4.50139300	-0.00958000	-1.19965800
C	-4.50156800	-0.00768300	1.19985300
C	-5.21883500	-0.00932800	0.00020200
H	-5.04614800	-0.01618100	-2.14786800
H	-5.04623500	-0.01279100	2.14808600
N	1.91300400	0.00064500	-0.00000600
C	3.35657700	0.00085800	-0.00012500
C	4.03527700	0.00093900	-1.21707400
C	4.03547600	0.00102700	1.21671300
C	5.42862500	0.00117100	-1.21016500
H	3.47632800	0.00081800	-2.15496200
C	5.42882300	0.00126200	1.20957800
H	3.47668000	0.00098100	2.15469300
C	6.12318500	0.00133400	-0.00035000
H	5.97287300	0.00122400	-2.15608400
H	5.97322500	0.00139200	2.15540800
H	7.21460400	0.00152000	-0.00044000
C	-2.35360000	-0.00630100	-2.52933700
H	-1.70429100	0.87884100	-2.61957300
H	-1.70532300	-0.89245100	-2.61746900
H	-3.04009600	-0.00692500	-3.38598700
C	-2.35373000	-0.00243600	2.52965700
H	-1.70534800	-0.88836800	2.61917900
H	-1.70456500	0.88292800	2.61874800
H	-3.04037100	-0.00192600	3.38619000
C	-6.72363200	0.01768400	-0.00097900
H	-7.09036600	1.05619900	-0.03162400
H	-7.13495500	-0.50184400	-0.87752100
H	-7.13616900	-0.45065600	0.90322400

PC•

C	1.26808200	-3.65122100	0.00077200
C	1.94830900	-2.43565200	0.00047200
C	1.24483900	-1.22453400	0.00029100
C	-0.17993700	-1.23349100	0.00041000
C	-0.83654400	-2.48683300	0.00073300
C	-0.12856700	-3.67806700	0.00090800
C	-0.90007900	-0.00036900	0.00018700
C	-0.18107900	1.23339300	-0.00008000
C	1.24372800	1.22571400	-0.00015900
C	1.94610000	2.43746700	-0.00037800
H	3.03574400	2.43246800	-0.00042600
C	1.26479600	3.65243500	-0.00053800
C	-0.13187200	3.67803300	-0.00048600
C	-0.83877200	2.48615700	-0.00026100
H	1.83840800	-4.58225200	0.00090400
H	3.03794900	-2.42965900	0.00037300
H	-1.92840700	-2.49464700	0.00085000
H	-0.66232900	-4.63040300	0.00115500
H	1.83429700	4.58397100	-0.00070500
H	-0.66646400	4.62990700	-0.00061400
H	-1.93062100	2.49291600	-0.00020200
C	-2.38855400	-0.00112600	0.00022100
C	-3.09048200	-0.00488900	-1.21860600
C	-3.09039200	-0.00334800	1.21879700
C	-4.48701300	-0.00956700	-1.19835400
C	-4.48719500	-0.00800000	1.19851900

C	-5.20468600	-0.00885700	0.00018400
H	-5.03083100	-0.01580300	-2.14796600
H	-5.03095600	-0.01299200	2.14813700
N	1.91646500	0.00090400	-0.00001600
C	3.34557700	0.00157100	-0.00011900
C	4.03987500	0.00173000	-1.21013400
C	4.04005200	0.00209600	1.20979600
C	5.43323100	0.00241200	-1.20833500
H	3.47674400	0.00131400	-2.14536500
C	5.43340700	0.00277500	1.20779300
H	3.47705900	0.00196300	2.14511000
C	6.13062200	0.00293500	-0.00032200
H	5.97708000	0.00253600	-2.15519100
H	5.97739400	0.00318300	2.15457000
H	7.22261300	0.00347200	-0.00040200
C	-2.33574900	-0.00774100	-2.52237800
H	-1.68308400	0.87554600	-2.60169500
H	-1.68431600	-0.89223300	-2.59881300
H	-3.02007700	-0.00866000	-3.38202900
C	-2.33589300	-0.00457100	2.52271200
H	-1.68432800	-0.88886500	2.60030100
H	-1.68339900	0.87892300	2.60115300
H	-3.02037700	-0.00461300	3.38224000
C	-6.71131900	0.02119400	-0.00096600
H	-7.08227700	1.05875700	-0.03180400
H	-7.12363900	-0.50044100	-0.87677400
H	-7.12434700	-0.44859800	0.90322800

L

C	1.15485200	0.26644600	0.00000600
C	1.19868100	-1.13278800	0.00005200
C	0.00000700	-1.83843100	0.00001800
C	-1.19867900	-1.13279700	-0.00005400
C	-1.15486200	0.26643200	-0.00007100
N	-0.00000500	0.93582300	-0.00005300
H	0.00001100	-2.93102000	0.00003100
H	2.15775800	-1.65432800	0.00009200
H	-2.15775000	-1.65434600	-0.00010200
C	-2.40983200	1.09677000	0.00004200
H	-2.42709200	1.75241200	-0.88315300
H	-3.31553700	0.47547300	-0.00187800
H	-2.42893600	1.74938200	0.88546500
C	2.40983400	1.09677200	-0.00000300
H	2.42805100	1.75084200	-0.88435600
H	2.42800100	1.75094400	0.88427500
H	3.31552900	0.47545600	0.00005700

LH^+

C	1.20603800	-0.22681500	0.00000600
C	1.21071100	1.16051000	0.00002400
C	-0.00000500	1.85203200	0.00000600
C	-1.21071400	1.16050400	-0.00002600
C	-1.20603200	-0.22682400	-0.00003400
N	0.00000300	-0.84567500	-0.00002000
H	-0.00000600	2.94407300	0.00001300
H	2.16278100	1.69131600	0.00004400

H	-2.16278800	1.69130400	-0.00004700
C	-2.42877800	-1.08427200	0.00002500
H	-2.45374800	-1.72853600	0.89246300
H	-3.33252400	-0.46496900	-0.00073600
H	-2.45305700	-1.72976600	-0.89153600
C	2.42877900	-1.08427200	-0.00000100
H	2.45336000	-1.72921900	0.89195200
H	2.45343400	-1.72908500	-0.89204900
H	3.33252800	-0.46497600	0.00008100
H	0.00000600	-1.86559500	-0.00003500

Ph_3SiSH

Si	0.01891000	-0.00604400	0.63422900
